# Unrelated Fungal Rust Candidate Effectors Act on Overlapping Plant Functions

**DOI:** 10.3390/microorganisms9050996

**Published:** 2021-05-05

**Authors:** Karen Cristine Goncalves dos Santos, Gervais Pelletier, Armand Séguin, François Guillemette, Jeffrey Hawkes, Isabel Desgagné-Penix, Hugo Germain

**Affiliations:** 1 Department of Chemistry, Biochemistry and Physics, Université du Québec à Trois-Rivières, Trois-Rivières, QC G9A 5H9, Canada; cris.kgs@gmail.com (K.C.G.d.S.); Isabel.Desgagne-Penix@uqtr.ca (I.D.-P.); 2 Plant Biology Research Group, Université du Québec à Trois-Rivières, Trois-Rivières, QC G8Z 1V3, Canada; 3 Natural Resources Canada, Canadian Forest Service, Laurentian Forestry Centre, Quebec City, QC G1V 4C7, Canada; Gervais.Pelletier@canada.ca (G.P.); Armand.Seguin@canada.ca (A.S.); 4 Centre for Research on Aquatic Ecosystem Interactions (RIVE), Université du Québec à Trois-Rivières, Trois-Rivières, QC G8Z 1V3, Canada; Francois.Guillemette3@uqtr.ca; 5 Department of Chemistry—BMC, Analytical Chemistry, Uppsala University, VJ2J+92 Uppsala, Sweden; Jeffrey.Hawkes@kemi.uu.se

**Keywords:** transcriptome, metabolome, plant-microbe interactions, rust fungi, effector biology, *Melampsora larici-populina*

## Abstract

Rust fungi cause epidemics that threaten the production of important plant species, such as wheat and soy. *Melampsora larici-populina* (*Mlp*) causes the poplar rust and encodes at least 1184 candidate effectors (CEs) whose functions are poorly known. In this study, we sequenced the transcriptome and used mass spectrometry to analyze the metabolome of *Arabidopsis* plants constitutively expressing 14 *Mlp* CEs and of a control line to discover alterations leading to plant susceptibility. We found 2299 deregulated genes across the experiment. Genes involved in pattern-triggered immunity, such as FRK1, PR1, RBOHD, and WRKY33, as well as AUX/IAA genes were down-regulated. We further observed that 680 metabolites were deregulated in at least one CE-expressing transgenic line, with “highly unsaturated and phenolic compounds” and “peptides” enriched among down- and up-regulated metabolites. Interestingly, transgenic lines expressing unrelated CEs had correlated patterns of gene and metabolite deregulation, while expression of CEs belonging to the same family deregulated different genes and metabolites. Thus, our results uncouple effector sequence similarity and function. This supports that effector functional investigation in the context of their virulence activity and effect on plant susceptibility requires the investigation of the individual effector and precludes generalization based on sequence similarity.

## 1. Introduction

Plants must defend themselves against different types of pathogens. Their first line of defense consists of passive barriers, such as the cuticle and cell wall, which prevent pathogens from entering the plant tissue and its cells. Upon successful entry of a pathogen, conserved pathogenic motifs, called Microbe-Associated Molecular Patterns (MAMPs), may be detected and activate the Pattern-Triggered Immunity (PTI) [1]. PTI includes the transient accumulation of reactive oxygen species (ROS), callose deposition, alteration of hormone networks and activation of defense genes [2,3]. Finally, microorganisms secrete effectors into their host to modulate the host metabolism in favor of the pathogen. If detected, these effectors will activate the Effector-Triggered Immunity (ETI), leading to plant cell death in order to avoid the pathogen spreading to surrounding cells [4].

Rust fungi are the largest group of fungal plant pathogens, infecting ferns, gymnosperms and angiosperms and causing important losses in food production [5,6]. They are obligate biotrophs, produce two to five types of spores and infect one or two unrelated plant species to complete their life cycle [6]. To guard themselves against the defense mechanism of two different host species and to be able to feed on them, rust fungi deploy a large arsenal of effectors. To better comprehend the interaction between these pathogens and their hosts, and to provide new mechanisms to target in order to improve plant immunity, it is imperative that we understand how these effectors are secreted into host cells, how they evolve and how they act to promote pathogen growth [7,8]. While the precise number of *bona fide* effectors carried by each rust fungi species is unknown, Duplessis and colleagues [9] established that the poplar rust (*Melampsora larici-populina*) genome encodes 1184 small secreted proteins (SSPs) whereas the wheat stripe rust (*Puccinia graminis f. sp. tritici*) genome encodes 1106 SSPs [9], which are considered candidate effectors (CEs). These CEs are grouped within families based on sequence homologies [10,11]. Furthermore, effectors in the same family have been shown to interact with homologous R-proteins [12], however the virulence function of these effectors has seldom been investigated.

Previous studies have proposed different criteria to screen the genome of plant pathogenic fungi for high-priority CEs, including having less than 300 amino acids, high cysteine content, being expressed in infection structures during host infection or being detected in the host tissue during infection [10,13,14]. Once identified, putative effectors must be functionally characterized. In pathogens that are not obligate biotrophs, this can be achieved by silencing or overexpressing the gene encoding the CEs and analyzing the outcome of an infection [15,16]. For rust fungi and other obligate biotrophs, which are not amenable to genetic transformation, this direct investigative approach is not possible. The alternative solution proposed by different research groups is to use heterologous systems, either by transforming model plants to express the CE-encoding gene or by infecting model plants with pathogens able to express these genes [17,18]. This way, it is possible to evaluate if immunity is compromised, as it was shown that effectors expressed in heterologous systems conserve their capacity to alter the plant’s susceptibility to pathogens [19,20,21,22,23,24]. The stable and transient expression of CEs from *M. larici-populina* in *Arabidopsis thaliana* and *Nicotiana benthamiana*, from *Phakopsora pachirhyzi* in *N. benthamiana* and from *Hyaloperonospora arabidopsidis* in *A. thaliana* allowed the study of their subcellular localization in planta, their impact on the growth of different pathogens and the search for host proteins potentially targeted by the CEs [22,25,26,27].

Still, the impact of CEs in the plant may not be easy to detect or the isolated effect of a single CE may be too subtle to affect pathogen growth. In the study of Germain and colleagues, 14 CEs impacted the growth of *H. arabidopsidis* or *Pseudomonas syringae* pv *tomato* [22]. Eleven of the analyzed CEs displayed nucleocytoplasmic localization *in planta*, providing very limited information on possible host targets or helpers of these proteins [22]. Petre and colleagues found seven CEs of wheat yellow rust fungus (out of 16) with a specific accumulation pattern in plant cells (other than nucleocytoplasmic) and discovered specific plant protein interactors for six CEs [28]. Only three of the 16 CEs studied had both the specific accumulation pattern in *N. benthamiana* cells and specific plant protein interactors. Although the pathogen growth readout is informative regarding the impairment of the immune pathway, it is opaque with regard to which pathway has been tampered with or which metabolites are off-balance. Transcriptomic and metabolomic studies of stable transgenic plants expressing CEs have been useful in these cases, since they allow the detection of more subtle changes, unlikely to have a quantifiable impact on pathogen growth on their own [24,29,30].

Here, we studied the transcriptome and metabolome of 14 transgenic *Arabidopsis* plant lines expressing *Mlp* CEs known to cause effector-triggered susceptibility in *Arabidopsis*. We identified 2299 deregulated genes using this approach, including PTI-related genes, such as FRK1, PR1, RBOHD and WRKY33, as well as several AUX/IAA genes and genes involved in specialized metabolism. Four lines expressing CEs from different families showed correlated patterns of gene deregulation, demonstrating that the current grouping based on sequence homology does not reflect the virulence function of these CEs. We also found important down-regulation of highly unsaturated and phenolic compounds and up-regulation of peptides in almost all CE-overexpressing lines. Overall, our results show a lack of correlation between the sequence similarity of the studied CEs and their overall deregulation of genes and metabolites. Taken together, our results demonstrate that CEs that have completely different sequences can alter the expression of the same gene sets, while CEs of the same family can target completely different gene sets. Therefore, it is not possible to estimate the function of a CE, its impact on the transcriptome or on the metabolome of the plant, based solely on its sequence or its similarity to another CE.

## 2. Materials and Methods

### 2.1. Plant Growth conditions

*Arabidopsis thaliana* transgenic plants in Columbia-0 background expressing GFP alone (control) or fused to a candidate effector of the fungus *Melampsora larici-populina* (Mlp37347, Mlp72983, Mlp102036, Mlp106078, Mlp123218, Mlp123227, Mlp123531, Mlp124256, Mlp124266, Mlp124357, Mlp124466, Mlp124497, Mlp124499, Mlp124518) previously obtained in our laboratory [22,30], were grown at 22 °C at 12 h/12 h light/dark cycles. The transgenes were constitutively expressed under Cauliflower Mosaic Virus 35S promoter [31].

### 2.2. RNA Extraction and Transcriptome Analysis

RNA was extracted from pooled aerial tissue of 2-week-old soil-grown plants, using three replicates per genotype, with the Plant Total RNA Mini Kit (Geneaid) using RB buffer following manufacturer’s protocol. The samples were treated with DNAse, then RNA quality was assessed using agarose gel electrophoresis. QC was performed using a 2100 Bioanalyzer (Agilent, Santa Clara, CA, USA) and only samples with an RNA Integrity Number higher than 7 were kept for library preparation. Libraries were generated with the NeoPrep Library Prep System (Illumina, Vancouver, BC, Canada) using the TruSeq Stranded mRNA Library Prep kit (Illumina) and 100 ng of total RNA as per the manufacturer’s recommendations. The libraries were then sequenced with an Illumina HiSeq 4000 Sequencer with paired end reads of 100 nt at the Genome Quebec Innovation Centre (McGill University, Montreal, QC, Canada).

The bioinformatic analyses were done with Compute Canada servers; the parameters used are presented in Table S1. We trimmed the reads using Trimmomatic [32] and we aligned the surviving paired reads to the genome of *A. thaliana* assembly TAIR10 with HISAT2 [33]. Unmapped reads were aligned to the sequences of the CEs, without signal peptide, attached to eGFP. We counted the reads assigned to each transcript with the R v4.0 packages Rsamtools v2.2.3 [34], GenomicAlignments and GenomicFeatures [35]. The general information of the sequencing results and mapping data is presented in Table S2. Before comparing the samples, we used the CustomSelection package [36] to select as reference genes the top 0.5% genes with lowest coefficient of variation of TPM among the 45 samples [37]. We assessed the variation between the replicates and the similarity of the samples with principal component analysis (Figure S1), using the result of the “varianceStabilizingTransformation” function as input to the function “plotPCA” of the DeSeq2 package [38] (with “ntop” equal to the total number of genes in the experiment). Differential expression analysis was performed with DeSeq2 [38], using the un-normalized counts as input. DeSeq2 uses normalization factors (calculated with the reference genes selected above) to normalize the counts and then estimates the dispersion of each gene. The differential expression is computed by fitting the negative binomial model and testing the hypothesis with the Wald test. We considered as deregulated the genes with |log_2_ Fold change| ≥ 2 (*p*-Value ≤ 0.01), when comparing each CE-expressing line to the control line. We used clusterProfiler [39] for GO term enrichment analysis and KEGGprofile v1.24.0 [40] for KEGG enrichment analysis. Sets of deregulated genes were computed using WGCNA [41]. We calculated the similarity of gene deregulation of different transgenic lines with the R package pvclust v2.2-0 [42], using Pearson’s correlation and 5000 bootstrap replications.

### 2.3. Metabolite Extraction and Metabolomics Analysis

Metabolites were extracted from pooled aerial tissue of 2-week-old soil-grown plants, with four replicates per genotype. After pulverizing the tissues with a TissueLyser (30 cycles per second for 45 s repeated 3 times), we added 300 μL of distilled water to it. From the mix of tissue and water, we used 100 μL of tissue slurry for an extraction with 1 mL of 20% methanol and a separate 100 μL for an extraction with 1 mL of 80% methanol. After agitation with the solvent, we pooled the samples of the same genotype and extraction together and filtered them using glass microfiber filters (Whatman GF/F CAT No. 1825-025). We evaporated the extracts with a speed vacuum at room temperature and chamber vacuum of 7.4 torrs and resolubilized them in 2 mL of distilled water. Then, we solid phase extracted 50 µg of dissolved organic carbon (DOC) of each sample, using Agilent PPL cartridges, and eluted it in 1 mL of 100% methanol.

The mass spectrometry was performed in an Orbitrap LTQ-Velos calibrated and tuned to maximize the peak at 369.1 in Suwannee River Fulvic Acid (SRFA) reference material. The extracts were analyzed by direct injection in negative mode at a resolution setting of 100,000, with accumulation time set to a maximum of 500 ms and a target of 1 × 10^6^ ions. Peaks were only considered for formula assignment if their intensity was higher than 10× the median noise baseline. We assigned formulas to masses using an in-house MATLAB script [43] and we allowed assignments with mass error <2 ppm. Briefly, formulas were considered over the ranges C_4-50_H_4-100_O_2-40_N_0-2_ under the conditions O ≤ C; 0.3C ≤ H ≤ 2.2C. For each sample, the intensity of the peaks was normalized so that the sum of the intensities equaled 10,000. The following analyses were performed using R software v4.0. We used the molecular formulas to calculate the modified aromaticity index (AImod) of each metabolite [44] and the compound categories were defined as: condensed aromatic (AImod > 0.66), polyphenolic (0.66 ≥ AImod > 0.5), highly unsaturated and phenolic (AImod < 0.5 and H/C < 1.5), aliphatic (2 ≥ H/C ≥ 1.5, N = 0), peptide (2 ≥ H/C ≥ 1.5, N > 0) or sugar (O/C > 0.9) [45].

The results of the two extractions, with 20% and 80% methanol, were combined and the fold changes (FC) were calculated as: log_2_((0.5 + M_x_^y^)/(0.5 + M_c_^y^)), where M_x_^y^ is the relative abundance of the metabolite y in the CE-sample x and M_c_^y^ is the relative abundance of the metabolite y in the control. For each sample, only metabolites with |FC| > 2 were considered to have relative abundance different to that of the control. Categories enriched among up- and down-regulated genes were found by applying Fisher’s test. We calculated the similarity of metabolite deregulation of different transgenic lines with the R package pvclust v2.2-0 [42], using Pearson’s correlation and 5000 bootstrap replications. Pairwise correlation of metabolite deregulation between specific transgenic lines was calculated with the function cor from the R package stats, using the method “pearson”. We were not able to analyze the extraction with 80% methanol of the transgenic line Mlp123218, thus the results presented for this line are only of the extraction with 20% methanol and they are compared to the results of the Control for the same extraction for consistency.

We searched the molecular formulas, obtained with the in-house script, in KEGG database using the R package KEGGREST 1.24.0 for identification of the metabolites detected. We also used Pathos [46] to search for metabolites with the same *m/z* (settings: negative mode, all organisms, H^+^ as adduct and mass error at 3 ppm).

### 2.4. Sequence Analysis and Integration

Multiple sequence alignment of CE amino acid sequences without signal peptides was performed with the software MEGA X [47] using Muscle [48] default settings. Evolutionary history was inferred using the UPGMA method and 1000 bootstrap replicates. Comparisons of dendrograms from CE sequence alignment, gene and metabolite deregulation correlation were done with the R package dendextend [49] by calculating the cophenetic correlation between two dendrograms. We performed pairwise sequence alignment of the 14 CEs using Needle [48], with default parameters.

## 3. Results

### 3.1. In Planta Expression of Candidate Fungal Effectors Results in Important Deregulation at the Transcriptome Level

*Melampsora larici-populina* CEs have been previously studied in heterologous systems for functional characterization [22,24,27,30,50]. In Table 1, we present features of the 14 CEs studied here. They were selected from the set of small secreted proteins from *M. larici-populina*, characterized for their small size (less than 300 amino acids), the presence of a signal peptide, the absence of a transmembrane domain and no sequence similarity to proteins outside the Pucciniales order [9]. Two families of CEs studied here, CPG5464 (Mlp124256 and Mlp124266, homologous to *M. lini* AvrP4) and CPGH1 (Mlp124497, Mlp124499 and Mlp124518), show evidence of positive selection. In addition, the genes encoding the 14 CEs selected for this study are up-regulated during poplar infection [51]. Mlp37347 is a homolog of the well-studied AvrL567 group from *M. lini* [52,53] and accumulates at the plasmodesmata in *Arabidopsis*. Mlp72983 accumulates in the chloroplast [22] and Mlp124357 is found in the tonoplast and was shown to interact with *Arabidopsis* and poplar Protein Disulfide Isomerase [30]. The other 11 CEs selected here have nucleocytosolic accumulation, the same as the marker protein GFP. Although information about these CEs is scarce, all of them impacted *Arabidopsis* susceptibility to either *Pseudomonas syringae* or to *Hyaloperonospora arabidopsidis* [22].

**Figure 1:**
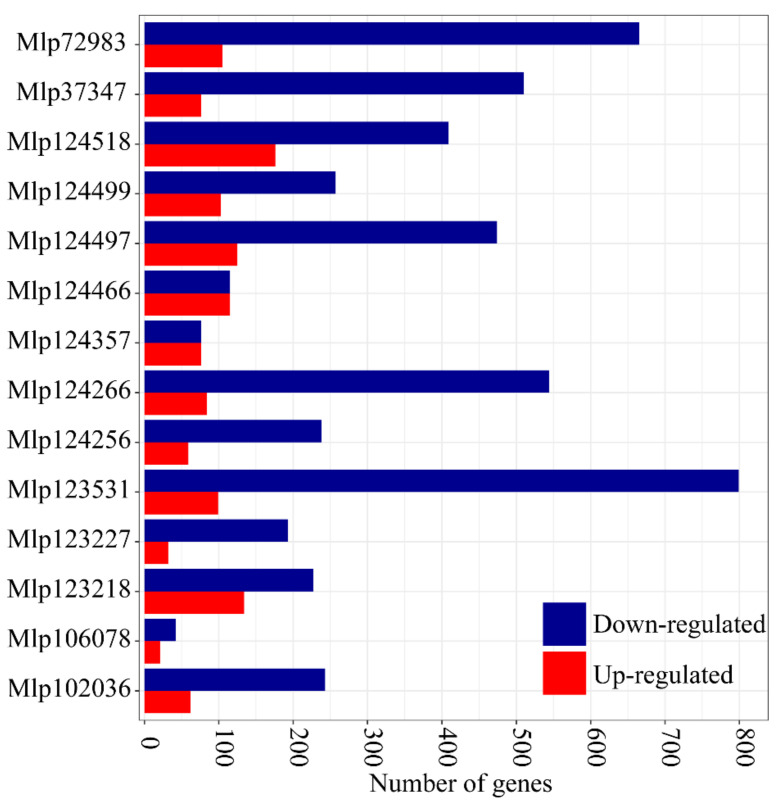
In planta expression of a candidate fungal effector results in important deregulation at the transcriptome level. Blue and red bars indicate the number of down- and up-regulated genes, respectively, in each CE-expressing transgenic line compared to the control line. The underlying data for this figure can be found in dos Santos et al. [37].

To better understand the mechanism through which these 14 CEs impact plants, we studied the transcriptome and the metabolome of transgenic *Arabidopsis* plants constitutively expressing them. In total, we found 2299 differentially expressed genes (DEGs) across the experiment. However, the number of DEGs in each line was variable, from 84 in Mlp106078 to 898 in Mlp123531 (Figure 1), indicating each CE affects the plant transcriptome to a different degree. The list of deregulated genes in each transgenic line is available in Table S3. We further assessed if the level of transgene expression could explain the number of DEGs in each sample and plotted the number of deregulated genes per transgenic line against expression level (in transcript per million) of the CE: GFP fusion transcripts. Linear regression shows a poor relation between the two (R^2^ = 0.1016, Figure S2) suggesting that the number of deregulated genes per line depends more on the identity of the expressed CE than on the strength of its expression

### 3.2. Hierarchical Clustering Based on Gene Expression Groups Effectors Independently of Amino Acid Sequence Homology

**Figure 2:**
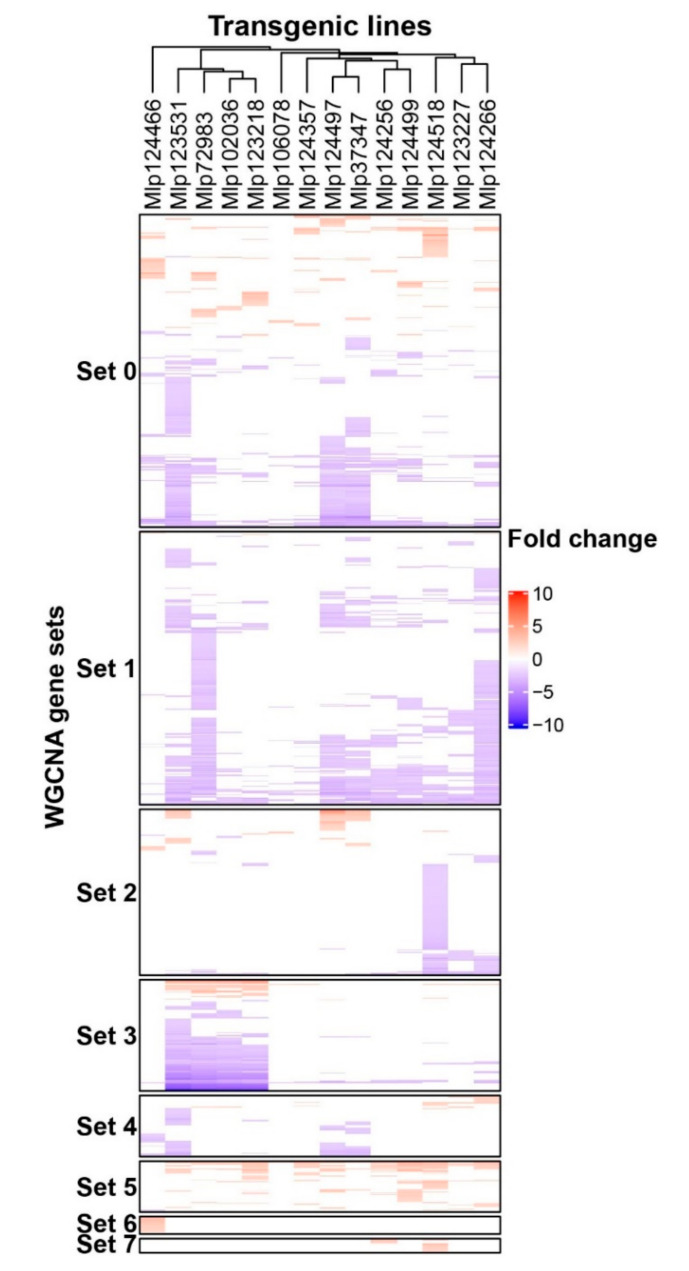
Heatmap of genes deregulated in each CE-expressing transgenic line. Up and down-regulated genes are shown in red and blue, respectively. Transgenic lines are displayed as columns and deregulated genes as lines. Sets of co-expressed genes (Sets 0 to 7) were calculated with WGCNA. Transgenic lines were grouped by correlation of gene deregulation using Pearson’s correlation coefficient. The underlying data for this figure can be found at dos Santos et al. [37].

CEs are typically grouped into families based on their amino acid sequences [10] and it has been shown that R-protein recognize related effectors [12]. Nevertheless, the virulence activity of effectors from the same family has rarely been studied. To search for gene deregulation patterns of related and unrelated CEs, we used WGCNA to cluster the co-expressed DEGs and Pearson’s correlation coefficient to cluster the transgenic lines (Figure 2). We found in total 208 GO terms enriched in the gene sets from WGCNA. A summary is presented in Table 2, and the full list of enriched terms is available in dos Santos et al. [37]. Set 0 clusters 714 genes deregulated across the 14 transgenic lines, 63.17% of which were down-regulated. Functions enriched in this gene set are related to defense, specialized metabolism, stress, and signaling pathways. Set 1 is composed of down-regulated genes enriched in GO terms related to defense responses, and all transgenic lines have down-regulated genes in this set. Of the 379 genes in Set 2, 76.5% were down-regulated and this set has enriched GO terms related to specialized metabolite biosynthesis. In the case of Set 3, 81.8% of the genes were down-regulated, but we did not find enriched GO terms in this gene set. Interestingly, this set is composed of genes with the same pattern of deregulation in four transgenic lines expressing effectors without sequence similarity (Mlp72983, Mlp102036, Mlp123218, and Mlp123531, Table S4) which accumulate in two separate cell compartments (Table 1). Set 4 is related to metabolism and abiotic stress and 77.6% of its genes were down-regulated. Sets 5, 6, and 7 are composed almost exclusively of up-regulated genes (Figure 2). Set 5 genes are deregulated in most transgenic lines and are related to abiotic stress and development. Set 6 is comprised of up-regulated genes almost exclusively found in the transgenic line Mlp124466 and related to transcription, vascular histogenesis, and response to different types of stress. Finally, Set 7 is made of genes related to photosynthesis and deregulated in the lines Mlp124256 and Mlp124518. In the cases of the Sets 0, 2, 3 and 4, there is mix of genes up and down-regulated, thus the enriched GO terms may be either up or down-regulated, or both. Interestingly, the dendrogram at the top of Figure 2. shows that CEs belonging to the same family (Mlp124497, Mlp124499 and Mlp124518; Mlp124256 and Mlp124266) fall in separate clusters despite their similarity at the amino acid level (Table S4).

**Figure 3:**
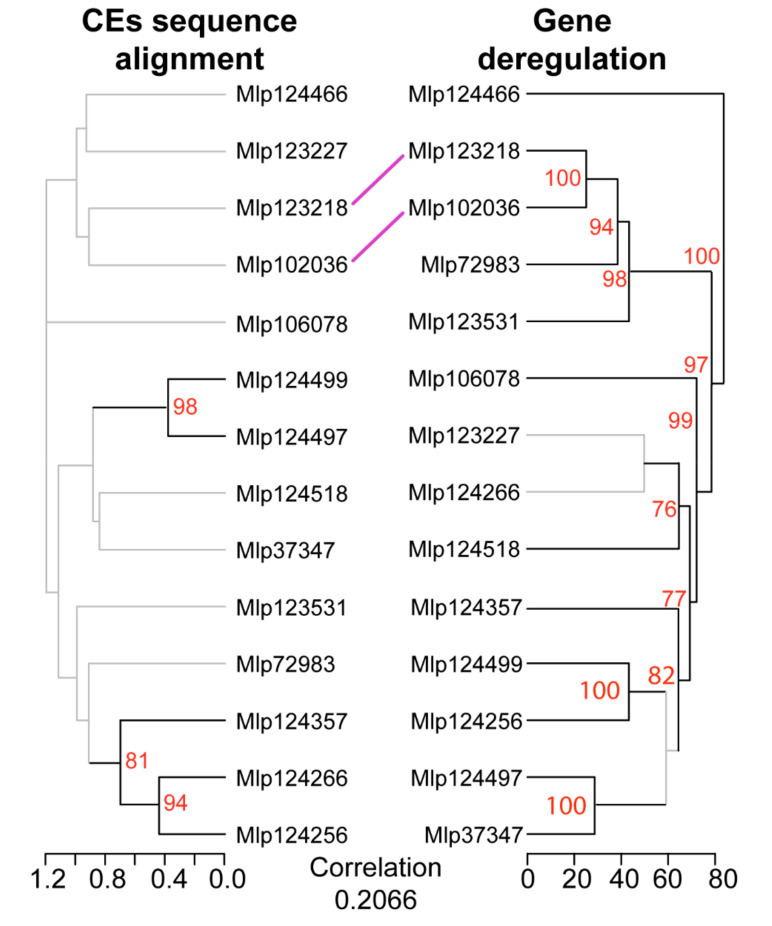
Hierarchical clustering of gene deregulation groups effectors independently of amino acid sequence homology. Comparison between dendrograms based on CE sequence similarity (left, tree computed with UPGMA from Muscle multiple sequence alignment) and on gene deregulation (right, computed with hierarchical clustering from Pearson’s correlation coefficient of gene fold change levels) shows only one cluster shared between the two (central lines) and an overall lack of correlation between the dendrograms (cophenetic correlation in the bottom). Branches with bootstrap support <70% are shown in grey. The underlying data for this figure can be found in the study by dos Santos et al. [37].

To analyze the relation between the sequence of each effector and its influence on the plant transcriptome, we compared the sequence alignment dendrogram to the differential expression dendrogram. After removal of the signal peptide, we aligned the sequences of the studied CEs and compared the resulting dendrogram with the one obtained from the gene deregulation correlation (Figure 3). Pearson’s correlation showed that transgenic lines expressing CEs from different families had correlated patterns of gene deregulation. Only one cluster was present in both dendrograms, Mlp102036 and Mlp123218, however this grouping is not supported in the effector sequence dendrogram (bootstrap value 8%) while it is in the gene deregulation dendrogram (bootstrap 100%). This analysis indicates that the sequence similarity between the CEs is not a good predictor of the impact they have on plant gene expression.

### 3.3. Effectors Converge on Deregulating the Same Metabolic Pathways while Others Display Unique Patterns

**Figure 4:**
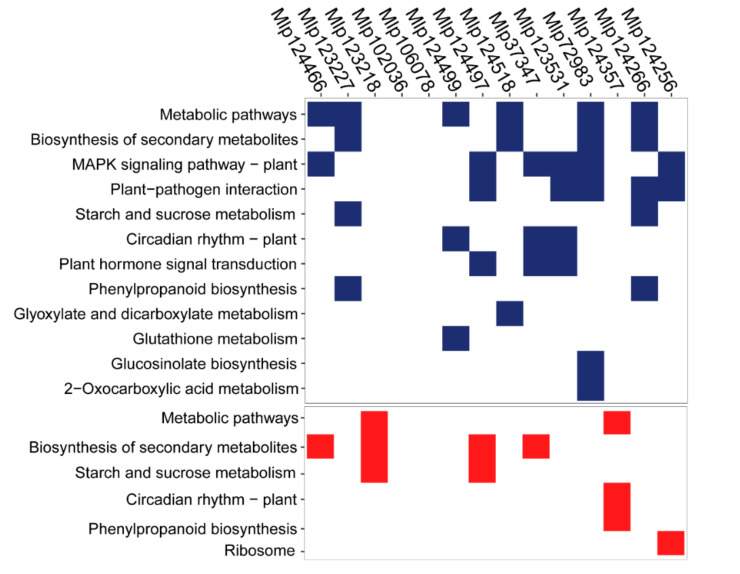
Effectors converge on deregulating the same metabolic pathways while others display unique patterns. KEGG pathways over-represented among the sets of down- (blue) and up- (red) regulated genes in each transgenic line (columns) were calculated with KEGGprofile. Transgenic lines are ordered according to dendrogram of sequence similarity calculated with Muscle and UPGMA. The underlying data for this figure can be found in the study by dos Santos et al. [37].

**Figure 5:**
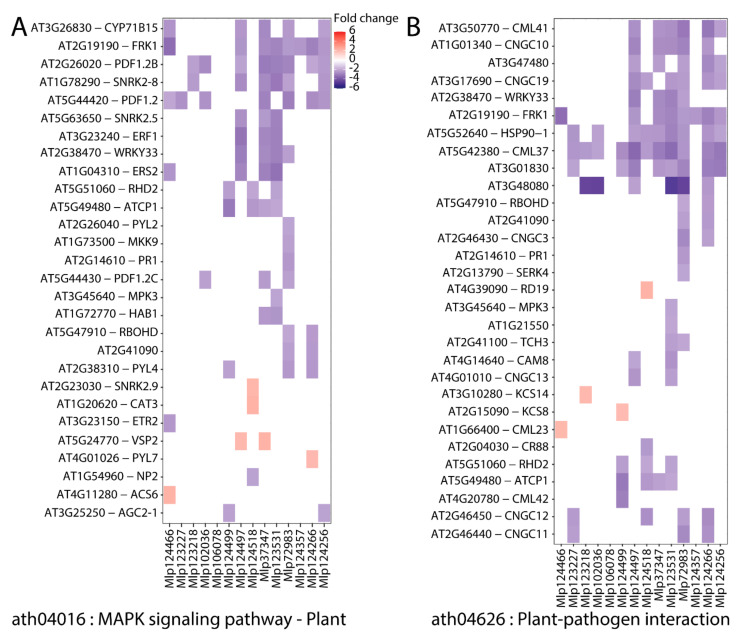
Heatmaps of genes belonging to (**A**) MAPK signaling pathway and (**B**) Plant–pathogen interaction deregulated in this experiment. Up- and down-regulated genes are shown in red and blue, respectively. Transgenic lines are ordered according to dendrogram of sequence similarity calculated with Muscle and UPGMA. The underlying data for this figure can be found at dos Santos et al. [37].

Even though the transcripts affected by related effectors are different, in theory they could fall within the same metabolic pathway and therefore similarly alter the plant. To test this hypothesis, we searched for KEGG pathways over-represented in the up- and down-regulated genes in each transgenic line. “Biosynthesis of secondary metabolites” and “Metabolic pathways” were enriched among gene sets (either up-, red, or down-regulated, blue) of eight transgenic lines, while “MAPK signaling pathway” and “Plant–pathogen interaction” were enriched among the down-regulated genes of six and five transgenic lines, respectively (Figure 4). In “MAPK signaling pathway” (Figure 5A) and “Plant–pathogen interaction” (Figure 5B), we observed the down-regulation of important plant defense-related genes such as WRKY33 (by four effectors: Mlp37347, Mlp72983, Mlp123531 and Mlp124497), PR1 (Mlp72983), PDF1.2a (by seven effectors: Mlp37347, Mlp72983, Mlp102036, Mlp123227, Mlp124256, Mlp124266 and Mlp124466), PDF1.2b (by eight effectors: (Mlp37347, Mlp72983, Mlp102036, Mlp123218, Mlp123531, Mlp124256, Mlp124266 and Mlp124497), PDF1.2c (by three effectors: Mlp37347, Mlp72983 and Mlp102036), MPK3 (Mlp123531), RBOHD (by two effectors: Mlp72983 and Mlp124266), as well as several calmodulin-like protein-encoding genes. Heatmaps of the genes deregulated in each transgenic line divided by KEGG pathways are available in File S1. Taken together, these results suggest that several of these fungal effectors converge on altering the expression of genes whose role is already well established in plant immunity, and with very few exceptions, the effectors downregulated those defense genes.

We also found that “Starch and sucrose metabolism” (File S1 Figure S35) was down-regulated in the transgenic lines Mlp123227 and Mlp124266 (several beta-amylase and beta-glucanase encoding genes as well as Cell-wall invertase 6 and Sucrose-phosphate synthase 4 were down-regulated in both transgenic lines), but up-regulated in the lines Mlp123218 and Mlp124497 (common up-regulation of isoamylase 3, disproportionating enzyme 1 and alpha-glucan phosphorylase 2), whereas several transgenic lines showed impact on specialized metabolism, such as carotenoid biosynthesis (File S1 Figure S6) and glucosinolate metabolism (File S1 Figure S13). This was also visible in the enriched GO terms found on the WGCNA gene sets (Table 2) and dos Santos et al. [37]). The circadian rhythm pathway, although it was only found to be enriched among the down-regulated genes of the lines Mlp124499, Mlp37347 and Mlp123531 and up-regulated genes in the Mlp124357 transgenic line, has genes deregulated in all the transgenic lines studied, with exception of Mlp124466 (File S1 Figure S7). Namely, pseudo-response regulator 3 and 5 were up-regulated while elongated hypocotyl 5 homolog and late elongated hypocotyl were down-regulated in almost all our transgenic lines. The plant–hormone signal transduction pathway is enriched among the down-regulated genes in the transgenic lines Mlp37347, Mlp123531, and Mlp124497. In these lines, we found several (17, 23, and 17, respectively) down-regulated genes related to auxin response. These results show that the CEs studied here can impact a wide range of plant mechanisms, deregulating genes in different pathways. They also show that CEs with similar sequences not only deregulate different genes but also alter different pathways.

### 3.4. Similar to the Transcriptome, the Metabolome Is Deregulated by Several Effectors

**Figure 6:**
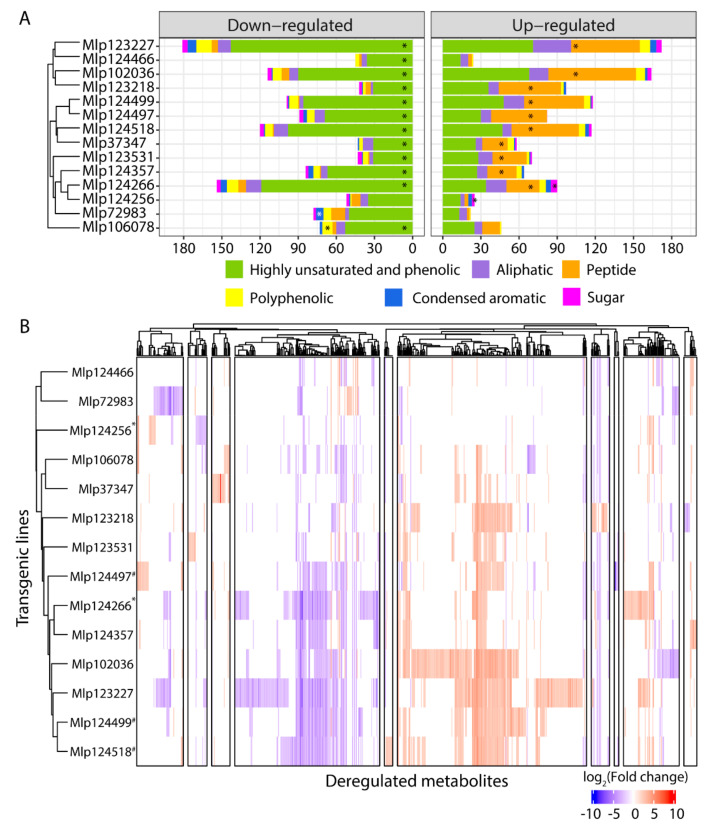
(**A**) Metabolites down-regulated (**left**) are enriched in highly unsaturated and phenolic compounds while peptides are over-represented among those up-regulated (**right**). Samples were analyzed in negative mode and relative abundance of metabolites in samples was compared to that in the control plants. Estimated molecular formulas were separated in six categories: highly unsaturated and phenolic (green), aliphatic (purple), peptide (orange), polyphenolic (yellow), condensed aromatic (blue), and sugar (pink). (**B**) Transgenic lines expressing candidate effectors with no similarity in amino acid sequence have correlated patterns of metabolite deregulation. Both metabolites and transgenic lines were clustered using Pearson’s correlation. * indicates transgenic lines with CEs from the CPG5464 family; # indicates transgenic lines with CEs from the CPGH1 family. The underlying data for this figure can be found in the study by dos Santos et al. [37].

As both primary and specialized metabolisms were affected at the transcriptomic level, and as they can have an important role in the outcome of an infection, we proceeded with an untargeted analysis of the metabolome of these plants. We extracted metabolites with aqueous solutions containing 20% and 80% methanol and used ultra-high resolution mass spectrometry in negative mode. A total of 5192 masses were assigned across the experiment, ranging from 2679 (Mlp123227) to 3151 (Mlp124357) masses in each transgenic line (Table S5, Figure S3A). When separated in biochemical categories, assigned formula belonged mostly to the highly unsaturated and phenolic and the aliphatic categories, while peptides, sugars, condensed aromatics and polyphenolics were less important both in number of formulas and in relative abundance (Figures S3A and S3B, respectively). Compared to the control, we found 680 assigned molecular formulas with a | log_2_-transformed Fold change | > 2 (Figure 6A), ranging from 69 metabolites in the line Mlp124466 (1.95% of the masses detected in this sample and/or in the control) to 353 in the line Mlp123227 (9.68% of the masses detected in this line and/or in the control, Table S5). In almost all transgenic lines, with the exception of Mlp72983 and Mlp124256, there was an over-representation of highly unsaturated and phenolic compounds among the down-regulated metabolites (accumulation level lower than in the control line) whereas up-regulated metabolites (accumulation level higher than in the control line) were enriched in peptides in almost all samples, except Mlp72983, Mlp106078 and Mlp124466 (Figure 6A, Table S6). As done with the transcriptomic data, we assessed whether the variation in the number of metabolites deregulated in each transgenic line could be explained by the level of expression of the transgene. For this, we plotted the number of deregulated metabolites per transgenic line (left *Y*-axis, blue, Figure S4) against the average expression level of the CEs in each transgenic line (*X*-axis, Figure S4). As the number of metabolites detected in each transgenic line varied (Figure S3A), we also plotted the ratio of deregulated metabolites:identified (detected either in the control or in the corresponding sample) metabolites in the right *Y*-axis (red, Figure S4). We found that the variation in transgene expression could explain neither the number (R^2^ = 0.0063, *p*-Value = 0.7872) nor the ratio of deregulated metabolites (R^2^ = 0.0033, *p*-Value = 0.8444), suggesting that the magnitude of the impact on the metabolome depends on the identity of the CE expressed in the plant rather than the strength of the CE expression.

**Figure 7:**
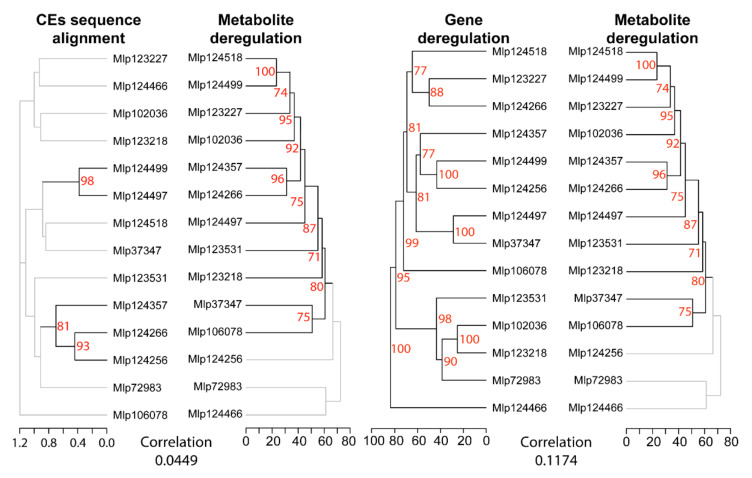
Pearson’s correlation of transgenic lines based on metabolite deregulation groups effectors independently of amino acid sequence homology, and gene deregulation patterns are not correlated to metabolite deregulation patterns in CE-expressing lines. The comparison between the dendrogram obtained from CE sequence alignment (multiple sequence alignment with Muscle of CEs without signal peptide and a tree computed with UPGMA, **left**) and the dendrogram of transgenic lines based on metabolite deregulation (**middle**) shows low correlation (correlation value on the **left**). Similarly, comparison between the dendrogram of transgenic lines based on metabolite deregulation and the one based on gene deregulation (**right**) shows a lack of correlation (correlation value on the **right**). Dendrograms based on correlation of metabolite deregulation or gene deregulation were calculated with Pearson’s correlation coefficient of fold Change levels and bootstrap values were obtained with pvclust. Branches with bootstrap support < 70% are shown in grey. The underlying data for this figure can be found in the study by dos Santos et al. [37].

In order to find shared patterns of metabolite deregulation across the transgenic lines studied, we used Pearson’s correlation to group metabolites with correlated deregulation across the experiment and transgenic lines which deregulated the same metabolites. As observed with the gene deregulation, we found that transgenic lines expressing non sequence similar CEs have correlated patterns of metabolite deregulation (Figure 6B). Within the CPGH1 family (CEs Mlp12497, Mlp124499, Mlp124518), lines Mlp124499 and Mlp124518 are correlated at 0.77 (Pearson’s correlation), but their correlation with the line Mlp124497 is less strong (Mlp12497-Mlp124499: 0.59; Mlp124497-Mlp124518: 0.64). The two AvrP4 homologues, Mlp124256 and Mlp124266, have 46.3% amino acid sequence similarity [35], but the correlation in metabolites deregulation patterns of the transgenic lines expressing these CEs is 0.32. On the other hand, although Mlp124266 and Mlp124357 have 21.2% amino acid sequence similarity (Table S4), multiple sequence alignment groups the AvrP4 homologues with the CE Mlp124357 (Figure 7) and the metabolite deregulation correlation between Mlp124266 and Mlp124357 lines is 0.69.

Remarkably, there was no correlation between the gene and metabolite deregulation dendrograms (cophenetic correlation of 0.1046, Figure 7). When considering the number of genes and metabolites deregulated in each sample, the correlation was also low (Pearson’s correlation = −0.1182). These results suggest these two omics approaches are needed to understand the magnitude of the impact of the CEs in the plant. Nevertheless, the possibility that the metabolic pathways deregulated at the metabolite level are the same as those deregulated in the gene level cannot be discarded.

To associate the metabolomic results with metabolic pathways, we tried to identify the molecular formula assigned in each sample. To do so, we searched for compounds with matching molecular formula or *m/z* values in the KEGG database. From the 5192 *m/z* detected across the experiment, 437 (8.41%) had a single match in KEGG database, while another 548 corresponded to multiple metabolites, and the rest were unmatched. When only considering the 680 deregulated metabolites, 54 (7.07%) matched a single metabolite and 82 (12.06%) matched multiple metabolites [55], leaving 544 unmatched. Taken together these results demonstrate that assigning metabolites identities to *m/z* values remains difficult even for a model plant such as *Arabidopsis*, for these reasons drawing correlation between deregulated transcriptome and deregulated metabolites or pathways cannot be performed with a high level of confidence.

## 4. Discussion

Effector biologists have tackled both the identification and the functional characterization of candidate effectors (CEs) [13,56], as this is a key step towards a better understanding of plant–microbe interactions. In rust fungi, different approaches are used in the functional characterization of these proteins, including analysis of subcellular localization in planta [22,27,28,30,50], infection assays in true host or in a model plant, and induction/repression of plant cell death [22,30,57,58]. The transcriptome or metabolome of the host in responses to the pathogen are frequently evaluated [59,60,61,62,63,64], but the assessment of the role of individual CEs in these processes is not easily measured and seldom analyzed [24,65]. Here, we investigated 14 CEs from *Melampsora larici-populina* by evaluating their individual impact on the transcriptome and metabolome of stable transgenic *Arabidopsis* plants. By studying the impact of several individual CEs, we were able to compare patterns of gene and metabolite deregulation. Unexpectedly, we found that transgenic lines expressing CEs belonging to the same family did not have comparable patterns of gene or metabolite deregulation.

Previous studies in *M. larici-populina* have shown that genes encoding fungal effectors are expressed in waves in the telial host [51] and that members of the same family may be expressed during the infection of different hosts [54]. This reflects the functional diversification of effectors, indicating that the fungus uses different sets of effectors for each stage of the infection, and suggesting that effector families can have different functions and may target different host proteins or the same host protein that diverged in different hosts. The concurrent study of individual *M. larici-populina* CEs allows the comparison of their individual impact in the plant [22]. We found variability in the magnitude of the impact of each CE on the transcriptome (from 84 to 898 DEGs) and the metabolome (from 69 to 363 metabolites deregulated) of the transgenic plants (Figure 1 and Figure 6A), a variability which is not related to the level of expression of the transgenes (Figures S2 and S4). This suggests that the identities of the CEs are orienting the deregulations. By comparing the correlation of gene and metabolite deregulation patterns with the CEs sequence similarity (Figure 3 and Figure 7), we show that CEs belonging to the same family do not deregulate the transcriptome or the metabolome in a same way nor do they deregulate the same metabolic pathways (Figure 4). These results corroborate the infection assays from Germain and colleagues [22]. In their study, *Arabidopsis* plants constitutively expressing *Mlp* CEs were infected with *P. syringae* DC3000 or *H. arabidopsidis* Noco2. Mlp124497, Mlp124499 and Mlp124518 (family CPGH1) and Mlp124256 and Mlp124266 (family CPG5464) [66]; all increased *Arabidopsis* susceptibility to *H. arabidopsidis*. However, only Mlp124266, Mlp124497 and Mlp124499 made *Arabidopsis* more susceptible to *P. syringae*.

It has been suggested that proteins with higher sequence similarity have higher probability of having the same function [67], thus small secreted proteins from many fungal and oomycete plant pathogens [9,10,68,69,70] have been grouped in protein families to guide functional annotation and to help understand effector evolution. Nevertheless, recent studies have hypothesized that effectors from the same family may have different functions in the same host. This is the case for HopAF1 effectors from *P. savastanoi* [71] and GALA effectors from *Ralstonia solanacearum* [72], which impact the plant defense differently. It is also the case for XopD effectors from plant pathogenic bacteria, which show different levels of SUMO protease activity and have different impacts in *Nicotiana* leaves [73]. This hypothesis is also supported by the evolution of the Tin2 effector in Ustilaginaceae. Tin2 from *Ustilago maydis* interacts with *Zea mays* TTK1 protein to stabilize it, leading to accumulation of anthocyanin. However, Tin2 from *Sporosorium reiliannum* interacts with *Zea mays* TTK2 and TTK3, inhibiting their activity [74].

The CEs studied here deregulate diverse biochemical pathways in the plant (Figure 4). In relation to primary metabolism, genes in the “starch and sucrose metabolism” pathway (File S1 Figure S35) were over-represented among up-regulated genes in the transgenic lines expressing the CEs Mlp123218 and Mlp124497, comparable to what is observed in susceptible wheat infected with *Puccinia triticina* [63]. On the other hand, the plants expressing Mlp123227 and Mlp124266 showed an enrichment of this pathway among down-regulated genes and the transgenic lines Mlp72983 and Mlp124266 had several genes down-regulated in this pathway as well (File S1 Figure S35), a pattern seen in resistant wheat infected with *P. triticina* [63]. This difference in the direction of gene deregulation within the same pathway by different CEs may be an indication that deregulated genes have different functions. It can also suggest that these CEs are used in different stages of the infection.

When considering genes related to defense, there is down-regulation of PTI-related genes (FRK1, MPK3, PR1, RBOHD, and WRKY33) and ETI-related genes (ERF1 and PDF1.2A, B and C) in several transgenic lines, especially Mlp37347 (AvrL567 homolog), Mlp72983, and Mlp123531 (Figure 5). Interestingly, of these three lines only Mlp37347 impacted plant susceptibility to bacterial infection, leading to less growth of *P. syringae* [22]. However, the three transgenic lines showed increased susceptibility to the oomycete *H. arabidopsidis* [22], which might be linked to the down-regulation of PTI. On the other hand, Mlp37347, Mlp123531 and Mlp124497 down-regulated several AUX/IAA genes (File S1 Figure S28), which repress auxin responses [75]. Since auxin represses SA-mediated defense responses [76], this could explain the increased susceptibility of these plants to *H. arabidopsidis* and of Mlp124497 to *P. syringae* [22].

For three pathways, the transcriptomic deregulations found in this study differ from previous reports of susceptible plants infected by rust fungi. While genes encoding Glutathione-S-transferase are down-regulated in at least one of 12 transgenic lines studied here (File S1 Figure S14), these genes are up-regulated in apple leaves infected with *Gymnosporangium yamadae* [62]. Moreover, Tremblay and colleagues [61] reported up-regulation of genes in the “photosystem” and “nitrogen metabolism” pathways in susceptible Glycine max infected with *P. pachyrhizi*, whereas genes from these pathways (File S1 Figures S27 and S20, respectively) were down-regulated in our transgenic lines. There are several possible explanations for these differences between previous studies and our own. First, our results may be due to the long-term exposure of our plants to CEs, as they are stable transgenic lines, whereas during the infection rust fungi secrete effectors in waves [51] so these proteins are not constitutively present in the host. It is also possible that results from Tao and colleagues [62] and Tremblay and colleagues [61] included the activation of PTI as well as the combinatory effect of multiple effectors, as they investigated plant response to the fungal infection, not to individual CEs. Our approach was to express CEs from *M. larici-populina* in a plant that cannot be infected by this fungus, and thus should not recognize these proteins nor mount active defense responses against them (ETI). Finally, although there are limitations of the use of heterologous systems, they allow faster functional characterization of CEs [17,77] and they may be indispensable for high-throughput studies of CEs of obligate biotrophic pathogens or other microorganisms not amenable to genetic manipulation [78,79].

Taken together, our results reinforce the hypothesis that the CEs studied here and functionally characterized by Germain and colleagues [22] are bona fide effectors. Nevertheless, future studies interested in CEs evaluated here should analyze more independent transgenic lines. In addition, since our methodology for the metabolomic analysis is semi-quantitative and does not allow the distinction of metabolites with the same *m/z*, follow up studies should use chromatography in tandem with mass spectrometry and should analyze more replicates for the mass spectrometry. Our study also questions the validity of grouping CEs by sequence similarity. The importance of this approach for understanding the evolution of effectors is obvious [9] but basing functional characterization on sequence similarity may be misleading [71,72,74].

## Figures and Tables

**Table 1:** Features of the CEs investigated in this study.

CE	Length (Cysteine)	Family (Members)	Subcellular Localization ^a^	U, P, B, L ^b,c^
Mlp37347	151 (2)	-	Plasmodesmata	E, HE, E, E
Mlp72983	220 (8)	CPG332-CPG333(13)	Chloroplast	E, HE, E, HE
Mlp102036	107 (0)	CPG2528(5)	Nucleocytosolic	E, HE, E, E
Mlp106078	137 (10)	-	Nucleocytosolic	E, HE, E, E
Mlp123218	209 (6)	CPG543(7)	Nucleocytosolic	E, HE, E, E
Mlp123227	124 (3)	CPG1059(2)	Nucleocytosolic	E, HE, E, HE
Mlp123531	102 (8)	CPG4557(3)	Nucleocytosolic	E, HE, E, E
Mlp124256	89 (6)	CPG5464(13)	Nucleocytosolic	N, N, E, E
Mlp124266	92 (7)	CPG5464(13)	Nucleocytosolic	N, N, E, E
Mlp124357	98 (6)	CPG4890	Tonoplast	N, N, E, E
Mlp124466	76 (0)	-	Nucleocytosolic	-
Mlp124497	77 (4)	CPGH1(33)	Nucleocytosolic	N, N, N, N
Mlp124499	72 (3)	CPGH1(33)	Nucleocytosolic	N, N, E, HE
Mlp124518	76 (3)	CPGH1(33)	Nucleocytosolic	N, N, E, E

^a^ Subcellular localization was evaluated in *Arabidopsis* [22]. ^b,c^ U, P, B, L refer to expression on: (U) urediniospores, (P) poplar leaves, (B) basidiospores or (L) larch needles [54], where E, HE, and N indicate that the CE is expressed, highly expressed, or was not detected, respectively, and indicates no data are available.

**Table 2:** Summary of “biological process” GO terms enriched in the WGCNA gene sets.

Set	Genes in the Set	Up-Regulated ^a^	Down-Regulated ^a^	Enriched GO Terms
**Set 0**	714	262	451	Response to water deprivation
				Cold acclimation; Leaf senescence
				Response to fungus, to chitin, to ROS
				Response to salt stress and to hypoxia
				Defense response to fungus Response to toxic substance
				Response to nitrogen compound and to ET
				Isoprenoid, triterpenoid and terpenoid biosynthesis
				Plant-type cell wall loosening
				Phosphorelay signal transduction system
**Set 1**	624	10	615	Response to drug, nitrogen, ROS and ozone
				Response to SA, JA and karrikin
				Response to wounding, to herbivore and insect
				Cellular response to light stimulus and hypoxia
				Cellular response to acid chemical
				Defense response (incompatible interaction)
				Defense response by callose deposition in cell wall
				Defense response by cell wall thickening
				SAR and ISR
				Camalexin, indole phytoalexin and SA biosynthesis
				Sulfur compound biosynthesis
				Toxin and phenol-containing compound biosynthesis
**Set 2**	379	89	290	Response to karrikin, to nutrient levels and to copper ion
				S-glycoside and unsaturated fatty acid biosynthesis
				Chlorophyll biosynthesis
				Tetraterpenoid, terpenoid and carotenoid biosynthesis
				Isoprenoid, glycosyl and xanthophyll metabolism
				Sulfur compound, cofactor and leucine biosynthesis
				Defense response to insect
				De-etiolation; Chloroplast organization
**Set 3**	253	47	207	No GO term enriched
**Set 4**	140	32	109	Response to water deprivation
				Response to salt stress and to starvation
				Cellular amino acid catabolism/metabolism
				ET-activated signaling pathway
				Indole-containing compound metabolism
**Set 5**	116	113	4	Circadian rhythm; Starch catabolism
				Response to cold
				Regulation of reproductive process
				Regulation of post-embryonic development
**Set 6**	40	38	2	Response to hypoxia and to wounding
				Response to drug, to chitin and to salt stress
				Transcription; Phloem or xylem histogenesis
**Set 7**	32	32	0	Photosynthesis; Proton transmembrane transport

^a^ Up- and down-regulated indicate the number of genes in the set that are up- or down-regulated in at least one transgenic line, thus there may be genes that are deregulated in both directions in the set because they are deregulated in opposite directions in different samples.

## Data Availability

Transcriptomic data presented in this study (raw reads and count matrices) are available in NCBI GEO under the accession GSE158410. Metabolomic data (raw and mzXML files along with annotation of metabolites and their relative abundances in each sample) are available at MetaboLights under the accession MTBLS2096. Data underlying figures, full list of enriched GO terms in the WGNCA gene sets, information on the deregulated metabolites and the list of selected reference genes are available at FigShare at 10.6084/m9.figshare.13166501.v3 [37].

## Supplementary Materials

The following are available online at https://www.mdpi.com/article/10.3390/microorganisms9050996/s1, Figure S1. Principal component analysis of the replicates of 14 transgenic lines expressing candidate effectors from Melampsora larici-populina attached to GFP and a control line expressing only GFP (black dots). Replicates of the same transgenic lines are close together, indicating the homogeneity of the sample, with the exception of one replicate of each of the following transgenic lines: Mlp102036 (yellow), Mlp106078 (red), Mlp124256 (sky blue) and Mlp124357 (dark green). The underlying data for this figure can be found at dos Santos et al. [37]. Figure S2. Magnitude of impact of CE on the plant’s transcriptome is independent of its level of expression. Reads not mapped to Arabidopsis genome were aligned to the transgene sequences (CE: GFP fusion) and average expression (in transcripts per million) across replicates of each transgenic line was calculated. Linear regression was performed using the number of genes deregulated in each transgenic line as the dependent variable and the average expression of the CE as the independent variable. The underlying data for this figure can be found in the study by dos Santos et al. [37]. Figure S3. Metabolic composition of samples in number of formulas (A) and relative abundance of compounds (B). Samples were analyzed in negative mode and estimated molecular formulas were separated into six categories: highly unsaturated and phenolic (green), aliphatic (purple), peptide (orange), polyphenolic (yellow), condensed aromatic (blue), and sugar (pink). The underlying data for this figure can be found in the study by dos Santos et al. [37]. Figure S4. Magnitude of impact of CE on the plant’s metabolome is independent of its level of expression, considering either the absolute number of deregulated metabolites (triangles, linear regression results in blue) or the ratio of metabolites deregulated by those identified (circles, linear regression results in red). Reads not mapped to Arabidopsis genome were aligned to the transgene sequences (CE: GFP fusion) and average expression (in transcripts per million) across replicates of each transgenic line was calculated. Two separate linear regressions were performed using the number of metabolites deregulated and the ratio between metabolites deregulated by those detected in each transgenic line as the dependent variables and the average expression of the CE as the independent variable in both cases. The underlying data for this figure can be found in the study by dos Santos et al. [37]. File S1. Heatmaps of deregulated genes separated by KEGG pathway. Pathways with at least 5 genes deregulated across the experiment were selected for display of genes deregulated in each transgenic line. Figure S1. Alanine, aspartate and glutamate metabolism. Figure S2. Alpha-Linolenic acid metabolism. Figure S3. Amino sugar and nucleotide sugar metabolism. Figure S4. Ascorbate and aldarate metabolism. Figure S5. Carbon fixation in photosynthetic organisms. Figure S6. Carotenoid biosynthesis. Figure S7. Circadian rhythm. Figure S8. Cutin, suberin and wax biosynthesis. Figure S9. Cyanoamino acid metabolism. Figure S10. Cysteine and methionine metabolism. Figure S11. Diterpenoid biosynthesis. Figure S12. Endocytosis. Figure S13. Glucosinolate biosynthesis. Figure S14. Glutathione metabolism. Figure S15. Glycerolipid metabolism. Figure S16. Glycerophospholipid metabolism. Figure S17. Glycine, serine and threonine metabolism. Figure S18. Glycolysis/Gluconeogenesis. Figure S19. Glyoxylate and dicarboxylate metabolism. Figure S20. Nitrogen metabolism. Figure S21. Oxidative phosphorylation. Figure S22. Pentose and glucuronate interconversions. Figure S23. Peroxisome. Figure S24. Phenylalanine metabolism. Figure S25. Phenylalanine, tyrosine and tryptophan biosynthesis. Figure S26. Phenylpropanoid biosynthesis. Figure S27. Photosynthesis. Figure S28. Plant hormone signal transduction. Figure S29. Porphyrin and chlorophyll metabolism. Figure S30. Protein processing in endoplasmic reticulum. Figure S31. Purine metabolism. Figure S32. Ribosome. Figure S33. Sesquiterpenoid and triterpenoid biosynthesis. Figure S34. Spliceosome. Figure S35. Starch and sucrose metabolism. Figure S36. Tropane, piperidine and pyridine alkaloid biosynthesis. Figure S37. Tryptophan metabolism. Figure S38. Tyrosine metabolism. Figure S39. Ubiquinone and other terpenoid-quinone biosynthesis. Figure S40. Ubiquitin mediated proteolysis. Figure S41. Valine, leucine and isoleucine degradation. Figure S42. Zeatin biosynthesis. The underlying data for this file can be found in the study by dos Santos et al. [37]. Table S1. Parameters used for bioinformatic analyses. Table S2. Sequencing results and alignment summary. Table S3. List of deregulated genes across the experiment with log2-transformed fold changes (FC) and false discovery rates (FDR) for each transgenic line. Table S4. Percentage of identity and similarity, presented as “ID (SIM)”, calculated with pairwise sequence alignment of CEs using Needle. Table S5. Summary of metabolomic analysis in negative mode of extractions with 20% and 80% methanol combined. Assigned, CHO, CHON and Mean mass refer exclusively to the sample in question, while the number of deregulated formulas considers those m/z detected in the sample or in the Control. Table S6. Metabolites assigned and deregulated in each sample separated by category. Identified metabolites are m/z detected either in the sample or in the control. The percentages were calculated by dividing the number of formulas assigned or deregulated in the sample in each category by the number of formulas identified in that sample and multiplying by 100.

## References

[1] Henry G., Thonart P., Ongena M. (2012). PAMPs, MAMPs, DAMPs and others: An update on the diversity of plant immunity elicitors. Biotechnol. Agron. Soc. Environ..

[2] Luna E., Pastor V., Robert J., Flors V., Mauch-Mani B., Ton J. (2011). Callose deposition: A multifaceted plant defense response. Mol. Plant-Microbe Interact..

[3] Bigeard J., Colcombet J., Hirt H. (2015). Signaling mechanisms in pattern-triggered immunity (PTI). Mol. Plant.

[4] Jones J.D.G., Dangl J.L. (2006). The plant immune system. Nature.

[5] Dean R., Van Kan J.A., Pretorius Z.A., Hammond-Kosack K.E., Di Pietro A., Spanu P.D., Rudd J.J., Dickman M., Kahmann R., Ellis J. (2012). The Top 10 fungal pathogens in molecular plant pathology. Mol. Plant Pathol..

[6] Aime M.C., McTaggart A.R., Mondo S.J., Duplessis S. (2017). Phylogenetics and phylogenomics of rust fungi. Adv. Genet..

[7] Hogenhout S.a., Van der Hoorn R.a.L., Terauchi R., Kamoun S. (2009). Emerging concepts in effector biology of plant-associated organisms. Mol. Plant-Microbe Interact..

[8] Dangl J.L., Horvath D.M., Staskawicz B.J. (2013). Pivoting the plant immune system from dissection to deployment. Science.

[9] Duplessis S., Cuomo C.A., Lin Y.-C., Aerts A., Tisserant E., Veneault-Fourrey C., Joly D.L., Hacquard S., Amselem J., Cantarel B.L. (2011). Obligate biotrophy features unraveled by the genomic analysis of rust fungi. Proc. Natl. Acad. Sci. USA.

[10] Saunders D.G.O., Win J., Cano L.M., Szabo L.J., Kamoun S., Raffaele S. (2012). Using hierarchical clustering of secreted protein families to classify and rank candidate effectors of rust fungi. PLoS ONE.

[11] Enright A.J., Dongen S.V., Ouzounis C.A. (2002). An efficient algorithm for large-scale detection of protein families. Nucleic Acids Res..

[12] Ravensdale M., Nemri A., Thrall P.H., Ellis J.G., Dodds P.N. (2010). Co-evolutionary interactions between host resistance and pathogen effector genes in flax rust disease. Mol. Plant Pathol..

[13] Sperschneider J., Dodds P.N., Gardiner D.M., Manners J.M., Singh K.B., Taylor J.M. (2015). Advances and challenges in computational prediction of effectors from plant pathogenic fungi. PLoS Pathog..

[14] Lorrain C., Hecker A., Duplessis S. (2015). Effector-mining in the poplar rust fungus *Melampsora larici-populina*. Front. Plant Sci..

[15] Lyu X., Shen C., Fu Y., Xie J., Jiang D., Li G., Cheng J. (2016). A small secreted virulence-related protein isessential for the necrotrophic interactions of *Sclerotinia sclerotiorum* with its host plants. PLoS Pathog..

[16] Li Z., Yin Z., Fan Y., Xu M., Kang Z., Huang L. (2015). Candidate effector proteins of the necrotrophic apple canker pathogen *Valsa mali* can suppress BAX-induced PCD. Front. Plant Sci..

[17] Lorrain C., Petre B., Duplessis S. (2018). Show me the way: Rust effector targets in heterologous plant systems. Curr. Opin. Microbiol..

[18] Chaudhari P., Ahmed B., Joly D.L., Germain H. (2014). Effector biology during biotrophic invasion of plant cells. Virulence.

[19] Pitino M., Armstrong C.M., Cano L.M., Duan Y. (2016). Transient expression of *Candidatus* Liberibacter Asiaticus effector induces cell death in *Nicotiana benthamiana*. Front. Plant Sci..

[20] Jamir Y., Guo M., Oh H.S., Petnicki-Ocwieja T., Chen S., Tang X., Dickman M.B., Collmer A., Alfano J.R. (2004). Identification of *Pseudomonas syringae* type III effectors that can suppress programmed cell death in plants and yeast. Plant J..

[21] Houterman P.M., Ma L., van Ooijen G., De Vroomen M.J., Cornelissen B.J.C., Takken F.L.W., Rep M. (2009). The effector protein Avr2 of the xylem-colonizing fungus *Fusarium oxysporum* activates the tomato resistance protein I-2 intracellularly. Plant J..

[22] Germain H., Joly D.L., Mireault C., Plourde M.B., Letanneur C., Stewart D., Morency M.-J., Petre B., Duplessis S., Séguin A. (2018). Infection assays in *Arabidopsis* reveal candidate effectors from the poplar rust fungus that promote susceptibility to bacteria and oomycete pathogens. Mol. Plant Pathol..

[23] Bentem S.D.L.F.v., Vossen J.H., Vries K.J.d., Wees S.v., Tameling W.I.L., Dekker H.L., Koster C.G.d., Haring M.A., Takken F.L.W., Cornelissen B.J.C. (2005). Heat shock protein 90 and its co-chaperone protein phosphatase 5 interact with distinct regions of the tomato I-2 disease resistance protein. Plant J..

[24] Ahmed M.B., Santos K.C.G.d., Petre B., Lorrain C., Duplessis S., Desgagne-Penix I., Germain H. (2018). A rust fungal effector binds plant DNA and modulates transcription. Nat. Sci. Rep..

[25] Kunjeti S.G., Iyer G., Johnson E., Li E., Broglie K.E. (2016). Identification of *Phakopsora pachyrhizi* candidate effectors with virulence activity in a distantly related pathosystem. Front. Plant Sci..

[26] Caillaud M.C., Piquerez S.J.M., Fabro G., Steinbrenner J., Ishaque N., Beynon J., Jones J.D.G. (2012). Subcellular localization of the *Hpa* RxLR effector repertoire identifies a tonoplast-associated protein HaRxL17 that confers enhanced plant susceptibility. Plant J..

[27] Petre B., Saunders D.G.O., Sklenar J., Lorrain C., Win J., Duplessis S., Kamoun S. (2015). Candidate effector proteins of the rust pathogen *Melampsora larici-populina* target diverse plant cell compartments. Mol. Plant-Microbe Interact..

[28] Petre B., Saunders D.G.O., Sklenar J., Lorrain C., Krasileva K.V., Win J., Duplessis S., Kamoun S. (2016). Heterologous expression screens in *Nicotiana benthamiana* identify a candidate effector of the wheat yellow rust pathogen that associates with processing bodies. PLoS ONE.

[29] Plett J.M., Kemppainen M., Kale S.D., Kohler A., Legué V., Brun A., Tyler B.M., Pardo A.G., Martin F. (2011). A secreted effector protein of *Laccaria bicolor* is required for symbiosis development. Curr. Biol..

[30] Madina M.H., Rahman M.S., Huang X., Zhang Y., Zheng H., Germain H. (2020). A poplar rust effector protein associates with protein disulfide isomerase and enhances plant susceptibility. Biology.

[31] Amack S.C., Antunes M.S. (2020). CaMV35S promoter—A plant biology and biotechnology workhorse in the era of synthetic biology. Curr. Plant Biol..

[32] Bolger A.M., Lohse M., Usadel B. (2014). Trimmomatic: A flexible trimmer for Illumina sequence data. Bioinformatics.

[33] Kim D., Paggi J.M., Park C., Bennett C., Salzberg S.L. (2019). Graph-based genome alignment and genotyping with HISAT2 and HISAT-genotype. Nat. Biotechnol..

[34] Morgan M., Pagès H., Obenchain V., Hayden N. Bioconductor—Rsamtools: Binary Alignment (BAM), FASTA, Variant Call (BCF), and Tabix File Import. R Package Version 2.2.3. http://bioconductor.riken.jp/packages/3.10/bioc/html/Rsamtools.html.

[35] Lawrence M., Huber W., Pagès H., Aboyoun P., Carlson M., Gentleman R., Morgan M., Carey V. (2013). Software for Computing and Annotating Genomic Ranges. PLoS Compu. Biol..

[36] Dos Santos K.C.G., Desgagne-Penix I., Germain H. (2020). Custom selected reference genes outperform pre-defined reference genes in transcriptomic analysis. BMC Genom..

[37] Dos Santos K.C.G., Pelletier G., Séguin A., Guillemette F., Hawkes J.A., Desgagné-Penix I., Germain H. FigShare—Supplementary Material: Differential Alteration of Plant Functions by Homologous Fungal Candidate Effectors.

[38] Love M.I., Huber W., Anders S. (2014). Moderated estimation of fold change and dispersion for RNA-seq data with DESeq2. Genome Biol..

[39] Yu G., Wang L., Han Y., He Q. (2012). clusterProfiler: An R package for comparing biological themes among gene clusters. OMICS J. Integr. Biol..

[40] Zhao S., Guo Y., Shyr Y. Bioconductor — KEGGprofile: An Annotation and Visualization Package for Multi-Types and Multi-Groups Expression Data in KEGG Pathway. R Package Version 1.24.0. https://bioconductor.org/packages/3.8/bioc/html/KEGGprofile.html.

[41] Langfelder P., Horvath S. (2008). WGCNA: An R package for weighted correlation network analysis. BMC Bioinform..

[42] Suzuki R., Terada Y., Shimodaira H. Github—Pvclust: Hierarchical Clustering with p-Values via Multiscale Bootstrap Resampling. R Package Version 2.2-0. https://github.com/shimo-lab/pvclust.

[43] Hawkes J.A., Patriarca C., Sjöberg P.J.R., Tranvik L.J., Bergquist J. (2018). Extreme isomeric complexity of dissolved organic matter found across aquatic environments. Limnol. Oceanogr. Lett..

[44] Koch B.P., Dittmar T. (2006). From mass to structure: An aromaticity index for high-resolution mass data of natural organic matter. Rapid Commun. Mass Spectrom..

[45] Kellerman A.M., Guillemette F., Podgorski D.C., Aiken G.R., Butler K.D., Spencer R.G.M. (2018). Unifying concepts linking dissolved organic matter composition to persistence in aquatic ecosystems. Environ. Sci. Technol..

[46] Leader D.P., Burgess K., Creek D., Barrett M.P. (2011). Pathos: A web facility that uses metabolic maps to display experimental changes in metabolites identified by mass spectrometry. Rapid Commun. Mass Spectrom..

[47] Kumar S., Stecher G., Li M., Knyaz C., Tamura K. (2018). MEGA X: Molecular evolutionary genetics analysis across computing platforms. Mol. Biol. Evol..

[48] Madeira F., Park Y.M., Lee J., Buso N., Gur T., Madhusoodanan N., Basutkar P., Tivey A.R.N., Potter S.C., Finn R.D. (2019). The EMBL-EBI search and sequence analysis tools APIs in 2019. Nucleic Acids Res..

[49] Galili T. (2015). dendextend: An R package for visualizing, adjusting, and comparing trees of hierarchical clustering. Bioinformatics.

[50] Gaouar O., Morency M.-J., Letanneur C., Séguin A., Germain H. (2016). The 124202 candidate effector of *Melampsora larici-populina* interacts with membranes in *Nicotiana* and *Arabidopsis*. Can. J. Plant Pathol..

[51] Duplessis S., Hacquard S., Delaruelle C., Tisserant E., Frey P., Martin F., Kohler A. (2011). *Melampsora larici-populina* transcript profiling during germination and timecourse infection of poplar leaves reveals dynamic expression patterns associated with virulence and biotrophy. Mol. Plant-Microbe Interact..

[52] Dodds P.N., Lawrence G.J., Catanzariti A.-M., Ayliffe M.A., Ellis J.G. (2004). The *Melampsora lini* AvrL567 Avirulence genes are expressed in haustoria and their products are recognized inside plant cells. Plant Cell.

[53] Gan P.H.P., Rafiqi M., Ellis J.G., Jones D.A., Hardham A.R., Dodds P.N. (2010). Lipid binding activities of flax rust AvrM and AvrL567 effectors. Plant Signal. Behav..

[54] Lorrain C., Marchal C., Hacquard S., Delaruelle C., Péytowski J., Petre B., Hecker A., Frey P., Duplessis S. (2018). The rust fungus *Melampsora larici-populina* expresses a conserved genetic program and distinct sets of secreted protein genes during infection of its two host plants, larch and poplar. Mol. Plant-Microbe Interact..

[55] Dos Santos K.C.G., Pelletier G., Séguin A., Guillemette F., Hawkes J.A., Desgagné-Penix I., Germain H. Embl Metabolights—MTBLS2096: Differential Alteration of Plant Functions by Homologous Fungal Candidate Effectors. https://www.ebi.ac.uk/metabolights/MTBLS2096/descriptors.

[56] Selin C., Kievit T.R.d., Belmonte M.F., Fernando W.G.D. (2016). Elucidating the role of effectors in plant-fungal interactions: Progress and challenges. Front. Microbiol..

[57] Schmidt S.M. (2009). Identification and Functional Characterization of Powdery Mildew Effectors. Doctoral Dissertation.

[58] Ramachandran S.R., Yin C., Kud J., Tanaka K., Mahoney A.K., Xiao F., Hulbert S.H. (2016). Effectors from wheat rust fungi suppress multiple plant defense responses. Phytopathology.

[59] Zhang H., Yang Y., Wang C., Liu M., Li H., Fu Y., Wang Y., Nie Y., Liu X., Ji W. (2014). Large-scale transcriptome comparison reveals distinct gene activations in wheat responding to stripe rust and powdery mildew. BMC Genom..

[60] Trujillo-Moya C., Ganthaler A., Stöggl W., Kranner I., Schüler S., Ertl R., Schlosser S., George J.-P., Mayr S. (2020). RNA-Seq and secondary metabolite analyses reveal a putative defence-transcriptome in Norway spruce (*Picea abies*) against needle bladder rust (*Chrysomyxa rhododendri*) infection. BMC Genom..

[61] Tremblay A., Hosseini P., Alkharouf N.W., Li S., Matthews B.F. (2010). Transcriptome analysis of a compatible response by *Glycine max* to *Phakopsora pachyrhizi* infection. Plant Sci..

[62] Tao S., Auer L., Morin E., Liang Y.-M., Duplessis S. (2020). Transcriptome analysis of apple leaves infected by the rust fungus *Gymnosporangium yamadae* at two sporulation stages reveals detoxication and secondary metabolite host responses and fungal pathogenesis related genes. Mol. Plant-Microbe Interact..

[63] Chandra S., Singh D., Pathak J., Kumari S., Kumar M., Poddar R., Balyan H.S., Gupta P.K., Prabhu K.V., Mukhopadhyay K. (2016). *De novo* assembled wheat transcriptomes delineate differentially expressed host genes in response to leaf rust infection. PLoS ONE.

[64] Azaiez A., Boyle B., Levée V., Séguin A. (2009). Transcriptome profiling in hybrid poplar following interactions with *Melampsora* rust fungi. Mol. Plant-Microbe Interact..

[65] Meng Q., Gupta R., Kwon S.J., Wang Y., Agrawal G.K., Rakwal R., Park S.R., Kim S.T. (2018). Transcriptomic analysis of *Oryza sativa* leaves reveals key changes in response to *Magnaporthe oryzae* MSP1. Plant Pathol. J..

[66] Hacquard S., Joly D.L., Lin Y.C., Tisserant E., Feau N., Delaruelle C., Legué V., Kohler A., Tanguay P., Petre B. (2012). A comprehensive analysis of genes encoding small secreted proteins identifies candidate effectors in *Melampsora larici-populina* (poplar leaf rust). Mol. Plant-Microbe Interact..

[67] Joshi T., Xu D. (2007). Quantitative assessment of relationship between sequence similarity and function similarity. BMC Genom..

[68] Haas B.J., Kamoun S., Zody M.C., Jiang R.H.Y., Handsaker R.E., Cano L.M., Grabherr M., Kodira C.D., Raffaele S., Torto-Alalibo T. (2009). Genome sequence and analysis of the Irish potato famine pathogen *Phytophthora infestans*. Nature.

[69] Evangelisti E., Gogleva A., Hainaux T., Doumane M., Tulin F., Quan C., Yunusov T., Floch K., Schornack S. (2017). Time-resolved dual transcriptomics reveal early induced *Nicotiana benthamiana* root genes and conserved infection-promoting *Phytophthora palmivora* effectors. BMC Biol..

[70] Anderson J.P., Sperschneider J., Win J., Kidd B., Yoshida K., Hane J., Saunders D.G.O., Singh K.B. (2017). Comparative secretome analysis of *Rhizoctonia solani* isolates with different host ranges reveals unique secretomes and cell death inducing effectors. Nat. Sci. Rep..

[71] Castañeda-Ojeda M.P., López-Solanilla E., Ramos C. (2017). Differential modulation of plant immune responses by diverse members of the *Pseudomonas savastanoi* pv. *savastanoi* HopAF type III effector family. Mol. Plant Pathol..

[72] Remigi P., Anisimova M., Guidot A., Genin S., Peeters N. (2011). Functional diversification of the GALA type III effector family contributes to *Ralstonia solanacearum* adaptation on different plant hosts. New Phytol..

[73] Kim J.-G., Taylor K.W., Mudgett M.B. (2011). Comparative analysis of the XopD type III secretion (T3S) effector family in plant pathogenic bacteria. Mol. Plant Pathol..

[74] Tanaka S., Schweizer G., Rössel N., Thines M., Kahmann R. (2019). Neofunctionalization of the secreted Tin2 effector in the fungal pathogen *Ustilago maydis*. Nat. Microbiol..

[75] Zenser N., Ellsmore A., Leasure C., Callis J. (2001). Auxin modulates the degradation rate of Aux/IAA proteins. Proc. Natl. Acad. Sci. USA.

[76] Iglesias M.J., Terrile M.C., Casalongué C.A. (2011). Auxin and salicylic acid signalings counteract the regulation of adaptive responses to stress. Plant Signal. Behav..

[77] Popa C., Coll N.S., Valls M., Sessa G. (2016). Yeast as a heterologous model system to uncover type III effector function. PLoS Pathog..

[78] Rice D.W., Sheehan K.B., Newton I.L.G. (2017). Large-scale identification of *Wolbachia pipientis* effectors. Genome Biol. Evol..

[79] Cunha M.d., Milho C., Almeida F., Pais S.V., Borges V., Maurício R., Borrego M.J., Gomes J.P., Mota L.J. (2014). Identification of type III secretion substrates of *Chlamydia trachomatis* using *Yersinia enterocolitica* as a heterologous system. BMC Microbiol..

