# Supplementary material for: Unrelated Fungal Rust Candidate Effectors Act on Overlapping Plant Functions
Source: Microorganisms. 2021 May 5;9(5):996. doi: 10.3390/microorganisms9050996 (PMC8148019; doi:10.3390/microorganisms9050996)
Supplement: Supplementary file 1 [file microorganisms-09-00996-s001.zip › Supplementary Table S1.pdf]

Supplementary Table S1. List of deregulated genes accros the experiment with log2-transformed fold changes (FC) and false discovery rates (FDR) for each transgenic line.

	Mlp37347		Mlp72983		Mlp102036		Mlp106078		Mlp123218		Mlp123227		Mlp123531		Mlp124256		Mlp124266		Mlp124357		Mlp124466		Mlp124497		Mlp124499		Mlp124518	
	FC	FDR	FC	FDR	FC	FDR	FC	FDR	FC	FDR	FC	FDR	FC	FDR	FC	FDR	FC	FDR	FC	FDR	FC	FDR	FC	FDR	FC	FDR	FC	FDR
AT1G01010	-2.2162	0.0000	-0.8083	0.0371	-0.8365	0.0493	-1.2397	0.0035	-0.6034	0.2110	-1.0377	0.0101	-2.3179	0.0000	-1.4398	0.0001	-1.2286	0.0007	-1.1965	0.0031	-1.2057	0.0027	-1.9703	0.0000	-1.4753	0.0001	-1.6465	0.0000
AT1G01060	-3.7765	0.0000	-2.2066	0.0003	-1.8490	0.0066	-2.1277	0.0036	-3.2088	0.0000	-1.7683	0.0100	-3.8362	0.0000	-2.9640	0.0000	-2.5530	0.0000	-0.3559	0.8004	0.7685	0.4021	-3.9914	0.0000	-2.2789	0.0003	-2.5244	0.0000
AT1G01110	-1.8546	0.0000	-0.0512	0.9386	-0.1697	0.8079	-0.1963	0.7921	-0.1322	0.8917	0.0601	0.9240	-1.8114	0.0000	-0.1717	0.8778	-0.1284	0.8127	-0.6500	0.2788	-0.3860	0.5498	-2.2581	0.0000	0.0190	0.9895	-0.1191	0.7954
AT1G01340	-2.5921	0.0000	-2.8256	0.0000	-1.4560	0.0055	-1.7422	0.0017	-1.2201	0.0254	-1.1356	0.0347	-2.6118	0.0000	-1.8284	0.0002	-2.3740	0.0000	-1.3760	0.0134	-0.6703	0.3301	-2.8007	0.0000	-1.9558	0.0000	-1.7151	0.0002
AT1G01390	-1.2395	0.0541	-1.7212	0.0076	-0.9613	0.2471	-1.5292	0.0578	-0.4671	0.6918	-2.3820	0.0004	-1.9655	0.0009	-0.4167	0.7768	-2.6588	0.0000	-1.2716	0.1215	-0.0032	1.0000	-1.1046	0.0963	0.4955	0.6684	-1.6351	0.0066
AT1G01490	-1.9456	0.0001	-1.1894	0.0293	-0.9633	0.1360	-1.4177	0.0259	-0.8781	0.1978	-0.6350	0.3007	-2.1986	0.0000	-0.9729	0.1482	-1.0643	0.0472	-0.8125	0.2785	-1.1849	0.0587	-1.8783	0.0001	-1.0574	0.0856	-0.8621	0.0821
AT1G01520	-3.2756	0.0000	-0.7849	0.3623	-2.5791	0.0003	-2.1234	0.0121	-3.4685	0.0000	-3.2841	0.0000	-3.3386	0.0000	-2.8820	0.0000	-2.5285	0.0003	-0.2422	0.8984	-0.6170	0.5796	-1.7423	0.0136	-2.4934	0.0005	-2.4198	0.0003
AT1G01560	-2.5550	0.0000	-2.5162	0.0000	-1.3490	0.0764	-0.6422	0.5181	-0.8558	0.3559	-1.3425	0.0591	-2.1451	0.0003	-2.3037	0.0004	-2.2017	0.0005	-2.3490	0.0005	-1.0761	0.2076	-3.4060	0.0000	-2.1431	0.0010	-1.2031	0.0474
AT1G01680	-3.1356	0.0000	-3.1784	0.0000	-1.6673	0.0442	-1.9326	0.0290	-1.2442	0.1841	-2.1361	0.0060	-2.6175	0.0001	-2.6001	0.0004	-3.5261	0.0000	-2.2588	0.0044	-1.7123	0.0455	-3.4192	0.0000	-2.6062	0.0003	-2.4370	0.0003
AT1G02230	-2.0177	0.0041	-0.9520	0.2621	-0.8763	0.3759	0.0862	0.9569	-0.5724	0.6601	-1.3463	0.0971	-1.7645	0.0099	-1.8078	0.0264	-1.6890	0.0218	-1.1343	0.2726	-0.6833	0.5371	-2.3296	0.0008	-2.0809	0.0064	-1.7341	0.0111
AT1G02300	0.7690	0.0380	1.4712	0.0000	1.8734	0.0000	0.2075	0.7470	2.3873	0.0000	0.3280	0.4749	2.2028	0.0000	0.7938	0.0735	0.2028	0.6550	0.1945	0.8186	-0.2381	0.7122	1.0033	0.0054	1.1594	0.0022	0.3807	0.2991
AT1G02340	-2.5662	0.0001	-0.8589	0.2978	-0.0105	0.9947	-1.2708	0.1747	-0.3284	0.8250	-0.3363	0.7137	-2.9717	0.0000	-0.2106	0.9178	-1.5272	0.0303	-0.8901	0.4137	-0.4689	0.6912	-2.4595	0.0002	0.1603	0.9256	0.2147	0.7761
AT1G02360	-2.0327	0.0000	-2.5994	0.0000	-1.2382	0.0018	-1.8779	0.0000	-1.6978	0.0000	-1.3052	0.0013	-2.3529	0.0000	-1.9202	0.0000	-2.1854	0.0000	-1.8061	0.0000	-0.4515	0.4142	-2.2106	0.0000	-1.8210	0.0000	-1.5370	0.0000
AT1G02400	-2.9829	0.0000	-0.4028	0.3332	0.5603	0.2018	-0.3587	0.4980	-0.3037	0.6116	-0.0179	0.9751	-3.8028	0.0000	-0.5237	0.2954	-0.1203	0.7860	-0.5256	0.2946	-0.1434	0.8309	-3.3911	0.0000	0.3105	0.5995	-0.0843	0.8264
AT1G02405	-1.5840	0.0202	-1.6855	0.0196	-1.1984	0.1675	-0.9279	0.3423	-0.9194	0.3485	-1.5036	NA	-2.0425	0.0014	-1.4489	0.0809	-2.0882	0.0029	-1.6404	0.0509	-1.5577	0.0656	-1.8243	0.0075	-0.7332	0.5014	-0.4137	0.5587
AT1G02450	-1.9634	0.0085	-2.0738	0.0069	-1.0168	0.3219	-0.9616	0.3954	-0.1605	0.9337	-2.0227	0.0140	-1.7047	0.0196	-1.7439	0.0496	-1.8555	0.0157	-1.1707	0.2881	-0.8777	0.4310	-1.7672	0.0207	-1.0544	0.3258	-0.7352	0.3292
AT1G02820	-2.4444	0.0000	-1.3341	0.0366	-1.9178	0.0026	-0.9878	0.2414	-2.1029	0.0006	-2.1428	0.0009	-2.5197	0.0000	-1.8234	0.0047	-2.0888	0.0004	-1.8223	0.0070	-1.2554	0.0950	-1.2953	0.0344	-1.8116	0.0043	-2.1655	0.0002
AT1G02920	-1.6617	0.0014	-2.1203	0.0000	-1.0992	0.0916	-0.5424	0.5195	-1.1688	0.0677	-0.6567	0.3036	-2.1399	0.0000	-1.4637	0.0139	-1.4187	0.0090	-1.0751	0.1257	-1.8197	0.0016	-1.9997	0.0001	-1.3927	0.0177	-0.1007	0.8697
AT1G03010	-3.0969	0.0000	-1.7823	0.0004	-0.9717	0.1242	-1.8845	0.0013	0.2466	0.8169	-1.3979	0.0142	-2.7732	0.0000	-1.0904	0.0772	-2.0511	0.0001	-1.5489	0.0076	-1.8743	0.0008	-3.0540	0.0000	-0.4856	0.5715	-0.9356	0.0577
AT1G03020	-2.3347	0.0008	0.0241	0.9840	-0.3120	0.7862	-0.1070	0.9434	-1.3981	0.1153	-0.2793	NA	-2.6225	0.0001	-0.7506	0.5456	0.3933	0.6366	-0.4898	0.7325	-1.1189	0.2489	-2.3661	0.0007	-0.3858	0.7918	-1.5745	0.0219
AT1G03055	-0.5381	0.4515	-1.4497	0.0275	-1.5518	0.0289	-0.8242	0.3645	-1.1094	0.1670	-1.5613	0.0240	-0.6458	0.3289	-1.6523	0.0174	-2.3832	0.0001	-1.3692	0.0793	-0.5365	0.5851	-0.5736	0.4316	-1.1562	0.1348	-3.1077	0.0000
AT1G03106	-0.1614	0.8738	2.9486	0.0000	0.9460	0.3366	-0.0197	0.9925	-0.0369	0.9888	-0.1529	0.8859	-0.1451	0.8788	-0.0667	0.9798	-0.0099	0.9935	-0.1222	0.9573	3.2178	0.0000	-0.2271	0.8262	-0.0552	0.9801	0.4583	0.5395
AT1G03630	1.1246	0.0332	-0.6338	0.3168	-0.4801	0.5344	-0.0213	0.9886	0.0859	0.9517	-1.5405	0.0068	1.1761	0.0201	0.1394	0.9298	-1.5235	0.0035	-0.6256	0.4708	0.3268	0.7218	1.1620	0.0293	0.1048	0.9365	-2.0701	0.0000
AT1G03790	0.3688	0.6974	3.0824	0.0000	0.9632	0.3427	0.7578	0.5136	0.3109	0.8506	0.1324	0.9053	0.0000	1.0000	0.4086	0.8281	1.6809	0.0279	0.5322	0.7269	2.3011	0.0050	1.1282	0.1600	0.0000	1.0000	1.8958	0.0070
AT1G03800	-1.0468	0.0730	-0.1031	0.9068	-0.0038	0.9979	0.2221	0.8313	-2.0439	0.0005	0.3436	0.6356	-1.5218	0.0046	-0.3800	0.7702	0.1556	0.8358	-0.4980	0.6448	-0.8497	0.2808	-1.3237	0.0216	-0.8395	0.2860	-0.0338	0.9598
AT1G04240	-1.4918	0.0005	-0.3222	0.5860	0.1172	0.8851	-0.8126	0.1844	-0.4194	0.5823	0.1494	0.8113	-2.0901	0.0000	-0.1494	0.9080	0.0999	0.8680	-0.2082	0.8443	0.5947	0.3499	-1.5124	0.0004	0.2948	0.7357	0.1927	0.6901
AT1G04290	-0.2316	0.7258	-2.0755	0.0001	-2.3736	0.0000	-0.1628	0.8708	-2.3902	0.0000	0.0397	0.9620	-2.3588	0.0000	-0.3058	0.8082	-0.7104	0.2110	0.0979	0.9511	0.3909	0.6432	0.4815	0.4185	0.0320	0.9856	-0.3647	0.4998
AT1G04310	-2.9021	0.0000	-1.0784	0.0056	-0.9940	0.0211	-0.4447	0.4483	-0.8506	0.0604	-0.4666	0.3057	-3.2922	0.0000	-0.5920	0.2882	-0.3909	0.3674	-0.9528	0.0380	-2.4136	0.0000	-2.7693	0.0000	0.0097	0.9968	-0.6709	0.0683
AT1G04313	1.3270	0.0869	-0.5841	0.5532	0.2756	0.8318	-0.1254	0.9394	1.5123	0.0990	0.4487	NA	1.4101	0.0557	2.2613	0.0046	-0.5276	0.5550	0.3381	0.8514	0.8013	0.4810	1.4173	0.0694	1.2080	0.2247	-0.0539	0.9513
AT1G04370	-2.3332	0.0013	-1.3737	0.0988	-1.4267	0.1256	-1.0520	0.3348	0.5907	0.6649	-1.5891	NA	-2.1882	0.0019	-1.0678	0.3493	-2.3644	0.0014	-1.3172	0.2043	-1.9146	0.0297	-2.4281	0.0008	-1.7494	0.0398	-1.8852	0.0079
AT1G04490	-1.3601	0.0563	-1.7025	0.0210	-1.4531	0.0868	-1.0416	0.2973	-0.5656	0.6526	-2.0983	0.0069	-1.3036	0.0562	-1.7964	0.0239	-2.2087	0.0019	-1.8190	0.0290	-0.8777	0.3838	-1.4637	0.0415	-1.9504	0.0109	-1.5219	0.0239
AT1G04520	-0.5657	0.3658	-0.9606	0.1250	-1.1591	0.0841	-0.4578	0.6107	-0.1929	0.8804	-1.2491	0.0441	-0.5625	0.3496	-0.2970	0.8348	-1.3683	0.0163	-0.3843	0.7396	0.4588	0.6087	-0.4928	0.4583	-0.6891	0.4111	-2.0386	0.0001
AT1G04530	-1.6088	0.0009	-1.3380	0.0092	-1.2039	0.0364	-1.2829	0.0417	-0.8621	0.1910	-1.3753	0.0121	-1.7029	0.0002	-1.1559	0.0506	-1.6477	0.0009	-0.7377	0.3281	-0.5235	0.4918	-1.6752	0.0005	-0.9699	0.1129	-2.3164	0.0000
AT1G04540	-1.5885	0.0055	-1.7009	0.0038	-1.4895	0.0235	-1.3455	0.0730	-0.9252	0.2381	-1.6504	0.0101	-1.9064	0.0004	-1.2027	0.0943	-1.9817	0.0006	-0.7307	0.4329	-0.1591	0.8991	-2.0168	0.0003	-1.5595	0.0141	-2.0220	0.0003
AT1G04580	0.2926	0.7689	2.2959	0.0017	1.8031	0.0332	0.3819	0.7762	2.1500	0.0060	-0.3323	NA	2.2897	0.0008	-0.2837	0.8947	0.3975	0.6692	0.2874	0.8838	-0.0801	0.9649	1.3224	0.0901	0.4464	0.7712	0.0935	0.9144
AT1G04600	-2.0520	0.0027	-2.1224	0.0025	-1.2516	0.1555	-1.6814	0.0597	-0.0953	0.9617	-1.3275	0.0931	-2.3586	0.0003	-1.3179	0.1454	-2.0104	0.0042	-1.3313	0.1523	-1.0606	0.2692	-2.2007	0.0012	-2.1531	0.0034	-1.7589	0.0084
AT1G04625	0.0000	1.0000	1.4795	0.0547	2.2810	0.0025	0.4435	0.7146	1.9736	0.0105	0.0000	NA	0.9541	0.2001	0.5202	0.7410	0.1361	0.8860	0.0000	1.0000	0.0000	1.0000	0.0176	0.9891	0.1687	0.9225	1.4658	0.0295
AT1G05035	1.0143	0.2012	-0.5217	0.6028	0.4043	0.7377	0.6241	0.6024	1.1511	0.2549	0.2934	0.7748	0.7930	0.3173	2.2067	0.0052	0.0588	0.9569	0.4059	0.8105	0.8021	0.4718	1.2176	0.1219	1.1525	0.2464	0.3883	0.6250
AT1G05090	0.1297	0.8904	0.7166	0.3765	0.2345	0.8373	0.2674	0.8268	2.8332	0.0000	0.6864	0.3908	0.2071	0.8078	0.4138	0.7916	0.4602	0.5581	0.3776	0.7983	0.7277	0.4600	0.4768	0.5599	0.3878	0.7751	0.2158	0.7692
AT1G05200	-0.4591	0.2414	-2.2615	0.0000	-2.1365	0.0000	-0.4866	0.3601	-1.6253	0.0000	-0.9537	0.0165	-2.2563	0.0000	-0.0522	0.9688	-0.9863	0.0070	-0.2764	0.7026	0.1356	0.8506	-0.2607	0.5620	-0.0502	0.9579	-0.7406	0.0320
AT1G05540	-0.7812	0.2138	-1.0355	0.1149	-0.6928	0.3968	-0.8782	0.2960	-0.2603	0.8391	-1.8762	0.0035	-0.0799	0.9192	-0.6193	0.5565	-1.7824	0.0026	-0.6073	0.5573	0.6746	0.4293	-0.5741	0.3997	-0.5223	0.6098	-2.0055	0.0004
AT1G05550	-0.7650	0.3081	1.4147	0.0377	1.0459	0.2119	-0.0371	0.9816	1.1874	0.1482	-0.8810	0.2880	2.3754	0.0000	-0.4546	0.7689	-0.6566	0.4036	-1.1108	0.2695	-0.1037	0.9467	-0.9029	0.2310	-0.4721	0.7222	0.0180	0.9827
AT1G05675	-2.3064	0.0000	-2.7976	0.0000	-1.0960	0.1110	-0.5408	0.5419	-2.1719	0.0001	-1.1393	0.0694	-2.8226	0.0000	-2.3389	0.0000	-2.1447	0.0001	-1.6449	0.0094	-1.1421	0.1070	-3.4523	0.0000	-2.1524	0.0001	-2.1870	0.0000
AT1G05680	0.0586	0.9587	0.0362	0.9772	-0.0280	0.9862	3.1430	0.0001	0.1004	0.9628	-0.2915	0.7777	0.0961	0.9249	-0.4058	0.8325	0.0878	0.9335	0.8120	0.5347	0.2787	0.8526	0.1515	0.8939	-0.9003	0.4332	0.2933	0.7202
AT1G05710	-0.4642	0.4221	-1.8031	0.0003	-2.0686	0.0001	-0.5254	0.5017	-1.5454	0.0042	-1.1987	0.0346	-1.0263	0.0369	-1.0424	0.0992	-1.3874	0.0073	-0.4844	0.6023	0.4011	0.6240	-0.4887	0.4085	-1.5220	0.0052	-1.3616	0.0055
AT1G05880	-3.3140	0.0000	-2.4014	0.0011	-1.1773	0.2229	-0.8054	0.4813	-1.0059	0.3506	-1.8258	0.0260	-2.3659	0.0006	-2.5703	0.0009	-2.5780	0.0004	-1.6743	0.0699	-2.0629	0.0154	-2.9494	0.0000	-2.7379	0.0002	-1.9751	0.0052
AT1G06080	-1.9422	0.0034	-1.8566	0.0072	-1.0995	0.2110	-1.2879	0.1623	-0.7800	0.4569	-2.3827	0.0010	-2.9428	0.0000	-0.5847	0.6717	-2.2832	0.0006	-2.5533	0.0004	-2.2718	0.0019	-2.0975	0.0014	1.0064	0.2784	-0.6323	0.3520
AT1G06100	2.0468	0.0052	0.1735	0.8813	0.9915	0.3297	0.7998	0.4890	0.7891	0.5121	0.2659	0.7986	2.0188	0.0043	0.7833	0.5653	-0.4264	0.6428	0.3282	0.8569	1.3247	0.1805	1.7660	0.0190	0.1683	0.9282	-0.6826	0.3663
AT1G06160	-0.7704	0.3237	-2.0599	0.0036	-1.2765	0.1496	0.4894	0.6769	-0.3370	0.8249	-0.7592	0.3704	-0.5522	0.4881	-0.5007	0.7538	-1.5757	0.0297	-0.8910	0.4308	-0.3981	0.7521	-0.3461	0.7134	-1.3037	0.1354	0.4519	0.5373
AT1G06360	1.5480	0.0003	1.1322	0.0134	0.7215	0.2105	0.7584	0.2215	1.0681	0.0351	0.5470	0.2998	1.4769	0.0003	0.9552	0.0763	0.1848	0.7448	0.7359	0.2427	2.2213	0.0000	1.7117	0.0000	1.0282	0.0413	-0.0303	0.9537
AT1G06460	1.2347	0.0012	0.9864	0.0156	1.2504	0.0026	0.4875	0.4123	1.4426	0.0002	0.2720	0.5887	1.4749	0.0000	0.7620	0.1317	0.6621	0.1133	0.9298	0.0530	-0.1193	0.8867	1.6282	0.0000	2.0194	0.0000	1.3613	0.0002
AT1G06570	-1.5612	0.0099	0.6129	0.4216	0.2521	0.8072	0.3057	0.7797	-1.4473	0.0390	0.9247	0.1906	-2.0026	0.0004	-0.1466	0.9401	1.0953	0.0882	0.5560	0.6294	-1.6627	0.0175	-1.5620	0.0105	-0.4825	0.6677	0.8249	0.1646
AT1G06640	0.0665	0.8851	-2.2545	0.0000	-1.5904	0.0000	-0.2099	0.7014	-1.8318	0.0000	-0.3704	0.3380	-1.6975	0.0000	-0.4733	0.3288	-0.7194	0.0309	-0.1005	0.9105	0.8591	0.0209	-0.1997	0.6303	-0.5355	0.2045	-0.4126	0.1921
AT1G06830	-0.1375	0.8708	-0.9848	0.1502	-1.2600	0.0821	0.2624	0.8136	-0.0767	0.9649	-0.4500	0.5543	0.0141	0.9866	0.2908	0.8548	-1.1202	0.0766	-0.0064	0.9978	0.7968	0.3508	-0.0049	0.9971	-2.1547	0.0005	-2.4366	0.0000
AT1G06980	0.2646	0.6992	0.5487	0.4120	1.1289	0.0745	0.1178	0.9142	0.8945	0.2048	0.4890	0.4674	0.5207	0.3654	0.7103	0.4002	0.2643	0.6963	1.0534	0.1288	1.0153	0.1408	0.3899	0.5571	1.6189	0.0032	2.8223	0.0000
AT1G06983	-2.3898	0.0004	-1.4004	0.0616	-1.3340	0.1179	-0.7390	0.4765	-0.9030	0.3671	-0.2030	NA	-2.0778	0.0012	-1.6776	0.0350	-0.9787	0.1855	-1.1976	0.2082	-0.7596	0.4550	-1.8610	0.0067	-1.5948	0.0463	-0.1266	0.8696
AT1G07000	-1.2319	0.0046	-2.3342	0.0000	-1.2646	0.0091	-1.3638	0.0102	-0.8817	0.1067	-0.9672	0.0501	-1.7463	0.0000	-1.6425	0.0003	-1.8058	0.0000	-1.7510	0.0002	-0.5035	0.4442	-1.8382	0.0000	-1.8712	0.0000	-0.9110	0.0305
AT1G07010	-1.2993	0.0141	-1.4750	0.0063	-1.4629	0.0127	-1.4134	0.0321	-1.3341	0.0253	-1.9868	0.0004	-1.1120	0.0321	-1.3627	0.0233	-2.0694	0.0001	-0.5525	0.5583	0.0593	0.9630	-1.0603	0.0545	-1.4500	0.0112	-2.4622	0.0000
AT1G07050	0.3520	0.7126	-0.4800	0.6351	0.9298	0.3638	-0.1501	0.9231	1.6682	0.0555	0.0206	0.9877	0.8185	0.3030	0.9485	0.4333	0.7588	0.3669	1.9213	0.0276	-0.7130	0.5393	2.5128	0.0004	1.0502	0.3172	0.8404	0.2522
AT1G07160	-2.3928	0.0001	-1.9361	0.0029	-0.6619	0.4623	-0.8938	0.3241	-1.7381	0.0136	-1.2144	0.0936	-2.4556	0.0000	-1.5581	0.0332	-1.2410	0.0623	-2.0018	0.0059	-2.1816	0.0023	-2.3150	0.0002	-2.1805	0.0014	-2.5131	0.0001
AT1G07180	-0.7668	0.0889	-0.9723	0.0353	-0.7926	0.1474	-0.6910	0.2629	-1.1248	0.0196	-1.1876	0.0124	-0.4207	0.3768	-1.3586	0.0033	-1.4839	0.0005	-1.1228	0.0286	-0.2509	0.7468	-0.8485	0.0604	-0.9828	0.0484	-2.3320	0.0000
AT1G07187	-2.2511	0.0001	-1.1416	0.0744	-1.0960	0.1339	-0.7345	0.4012	-2.2695	0.0001	-0.2642	0.7376	-2.4016	0.0000	-1.0701	0.1661	-0.2434	0.7436	-0.1892	0.9069	-1.4069	0.0447	-1.9860	0.0004	-1.4545	0.0257	-1.2645	0.0234
AT1G07367	2.5057	0.0004	0.0485	0.9685	0.0000	1.0000	0.9005	0.4166	0.0000	1.0000	0.0000	1.0000	2.8019	0.0000	0.0712	0.9794	0.6364	0.4487	0.0605	0.9788	0.3610	0.7897	4.4031	0.0000	0.0000	1.0000	0.0000	1.0000
AT1G07373	2.3502	0.0011	0.0499	0.9680	0.0917	0.9503	0.9289	0.4063	0.0000	1.0000	0.1629	0.8828	3.0004	0.0000	0.0734	0.9791	0.6086	0.4796	0.7224	0.5956	1.0316	0.3257	4.2245	0.0000	0.1508	0.9352	0.0000	1.0000
AT1G07390	-2.2583	0.0000	-0.9675	0.0209	-0.3507	0.5534	-1.1666	0.0164	-0.5174	0.3806	-0.9179	0.0397	-2.4484	0.0000	-1.2663	0.0030	-1.2069	0.0027	-0.8707	0.0832	-1.1023	0.0170	-1.7027	0.0000	-1.2271	0.0038	-1.1767	0.0022
AT1G07450	0.4640	0.5013	-0.6008	0.4223	-0.5897	0.5007	0.0875	0.9471	-0.6714	0.4773	-1.2223	0.0775	0.6953	0.2577	-0.5724	0.6177	-1.5108	0.0168	-0.7080	0.4799	0.1834	0.8833	0.5632	0.4137	-0.8295	0.3407	-2.0484	0.0007
AT1G07610	-1.1463	0.0325	0.8854	0.1382	0.0566	0.9578	-0.4543	0.5952	0.9417	0.1676	-0.0731	0.9288	-1.2857	0.0115	-0.5445	0.5787	0.5665	0.3393	0.1258	0.9359	-1.1516	0.0810	-1.1065	0.0429	-1.4300	0.0134	-2.0881	0.0000
AT1G07620	-1.2702	0.0911	-2.4731	0.0005	-1.4382	0.1045	-0.6560	0.5706	-0.6806	0.5833	-1.7284	0.0310	-1.5655	0.0261	-1.4054	0.1302	-2.1440	0.0030	-0.1468	0.9485	-0.1311	0.9373	-1.3338	0.0789	-1.6184	0.0521	-1.8514	0.0071
AT1G08050	-2.1894	0.0000	-1.8780	0.0000	-1.0994	0.0162	-0.9988	0.0560	-0.9251	0.0553	-1.2960	0.0035	-2.0229	0.0000	-2.0842	0.0000	-1.6114	0.0001	-1.8512	0.0000	-1.9737	0.0000	-2.4080	0.0000	-2.0468	0.0000	-1.5162	0.0001
AT1G08090	-0.6577	0.4119	-0.5601	0.5386	-0.3038	0.7957	-0.1028	0.9473	1.2175	0.1789	-1.1997	0.1387	-0.6228	0.4203	0.6225	0.6548	-1.4945	0.0419	0.3308	0.8403	2.4808	0.0007	-0.5348	0.5343	0.1825	0.9159	-0.0329	0.9689
AT1G08115	-0.7619	0.3438	0.4754	0.6187	-0.9065	0.3656	0.4011	0.7459	0.1028	0.9596	0.8002	0.3459	-0.4491	0.5891	-0.0267	0.9929	0.7308	0.3606	0.5463	0.6991	-0.0015	1.0000	0.4093	0.6549	0.3070	0.8466	2.0104	0.0023
AT1G08173	-2.0344	NA	-1.7791	0.0175	-1.2459	0.1815	-1.1530	NA	-0.3737	0.8119	-1.3830	NA	-1.8607	0.0078	-1.4950	NA	-1.9456	NA	-1.1674	NA	-1.0059	0.3307	-1.4575	0.0544	-1.5502	0.0640	-1.6532	NA
AT1G08830	-2.7158	0.0000	-0.8819	0.0077	-0.6306	0.1152	-0.3378	0.5035	0.2019	0.7537	-0.6420	0.0788	-3.0436	0.0000	-0.3021	0.6347	-0.5516	0.1090	-0.3035	0.6172	-0.1003	0.8839	-2.4543	0.0000	-0.6629	0.0869	-0.3946	0.2163
AT1G08860	-1.6473	0.0296	-2.3150	0.0019	-2.8494	0.0001	-0.4163	0.7520	-1.7595	0.0426	-1.6123	NA	-2.2619	0.0013	-2.4401	0.0020	-2.6507	0.0003	-2.2432	0.0079	-1.9802	0.0232	-2.2836	0.0018	-2.9827	0.0000	-2.7309	0.0001
AT1G08890	1.4345	0.0054	2.1308	0.0000	2.5038	0.0000	1.2002	0.0781	2.5353	0.0000	2.1140	0.0001	1.0248	0.0469	2.1637	0.0000	2.7907	0.0000	2.5503	0.0000	0.1750	0.8739	1.6042	0.0016	3.5948	0.0000	3.6316	0.0000
AT1G09080	-2.4181	0.0004	-2.5658	0.0002	-1.2069	0.1840	-1.7635	0.0486	-0.2379	0.8845	-2.5018	0.0010	-2.2120	0.0008	-1.9223	0.0142	-2.7687	0.0001	-0.6422	0.6305	-0.5468	0.6406	-2.0946	0.0025	-1.8028	0.0208	-1.4282	0.0340
AT1G09180	-1.9591	0.0018	-1.1120	0.1197	-1.4366	0.0576	-0.3902	0.7152	-1.0451	0.2189	-1.5554	0.0303	-2.1967	0.0002	-1.6251	0.0253	-1.2083	0.0720	-1.0161	0.2672	-0.8616	0.3406	-0.9723	0.1513	-0.2924	0.8320	-0.7179	0.2544
AT1G09240	-1.8066	0.0001	0.8453	0.1081	1.1166	0.0398	0.0188	0.9888	1.2590	0.0144	0.3976	0.5007	-1.7673	0.0000	0.1495	0.9142	-0.0479	0.9434	0.2790	0.7896	0.0439	0.9703	-2.0930	0.0000	1.1890	0.0220	1.4040	0.0016
AT1G09350	-0.7919	0.2494	1.7419	0.0056	1.4094	0.0528	0.7196	0.4519	3.2254	0.0000	1.4044	0.0418	-1.0786	0.0874	2.3230	0.0002	2.7218	0.0000	2.1466	0.0013	0.1890	0.8874	0.1010	0.9177	4.2621	0.0000	3.4792	0.0000
AT1G09410	-0.3442	0.6297	-1.7595	0.0047	-1.4746	0.0353	-0.9674	0.2417	-2.1708	0.0007	-1.1274	0.1017	-1.9018	0.0006	-0.5788	0.5971	-0.5669	0.3974	-0.3180	0.8089	-0.5435	0.5544	-0.5254	0.4450	-0.4379	0.6941	-0.6848	0.2476
AT1G09420	-0.3982	0.3833	2.3717	0.0000	2.4306	0.0000	0.1374	0.8606	2.1792	0.0000	0.4099	0.4026	1.6248	0.0000	0.1879	0.8598	0.6894	0.1037	0.6543	0.2520	-0.5063	0.3889	-0.2333	0.6545	-0.1094	0.9132	0.6800	0.0771
AT1G09680	-0.2988	0.6381	-1.8688	0.0004	-2.2315	0.0000	-0.9834	0.1629	-1.9983	0.0002	-0.4542	0.4845	-1.2195	0.0134	-0.6346	0.4588	-0.8776	0.1147	-0.4177	0.6764	-0.5771	0.4565	-0.3929	0.5309	-0.3016	0.7758	-0.9059	0.0756
AT1G09820	-0.6086	0.2785	-0.0698	0.9327	0.0390	0.9710	-0.8966	0.2098	0.0938	0.9444	-1.1057	0.0562	-0.2735	0.6551	-0.2888	0.8222	-1.0292	0.0560	-0.4366	0.6574	-0.2411	0.8037	-0.5296	0.3727	-0.3978	0.6770	-2.0298	0.0000
AT1G09910	-0.5446	0.0860	-1.4898	0.0000	-1.2622	0.0000	0.1231	0.8282	-1.3508	0.0000	0.4240	0.2353	-2.0403	0.0000	-0.0252	0.9833	0.5856	0.0659	0.4707	0.2903	-0.2191	0.6743	-0.4586	0.1661	0.3189	0.5258	0.7343	0.0111
AT1G09930	-0.9815	0.2239	-2.1328	0.0048	-0.8826	0.3992	-0.3170	0.8195	-1.3869	0.1461	-0.6855	NA	-2.4588	0.0004	-0.7408	0.5987	-1.6575	0.0321	-1.2468	0.2394	-1.2284	0.2245	-1.6598	0.0297	-1.4948	0.0977	-0.8276	0.2632
AT1G09932	-2.7059	0.0000	-3.0859	0.0000	-2.0409	0.0023	-1.7573	0.0250	-0.7464	0.4452	-2.0259	0.0030	-3.2235	0.0000	-2.4762	0.0001	-3.2802	0.0000	-1.7981	0.0135	-1.9262	0.0063	-3.4212	0.0000	-2.5783	0.0000	-2.9797	0.0000
AT1G09950	0.1158	0.8999	0.7061	0.3697	-0.5886	0.5514	-0.0071	0.9976	-0.3760	0.7805	-0.4014	0.6419	0.2523	0.7510	-0.5073	0.7217	-0.0475	0.9607	-0.1644	0.9308	2.7674	0.0000	-0.3268	0.7069	0.3472	0.8011	-0.1652	0.8244
AT1G10000	0.6480	0.2131	-3.3635	0.0000	-3.2934	0.0000	0.2300	0.7960	-3.3215	0.0000	0.0099	0.9914	-3.7630	0.0000	-0.2242	0.8675	-0.3819	0.5267	-0.3305	0.7618	0.8482	0.1898	-0.0159	0.9857	-0.0678	0.9602	0.1804	0.7457
AT1G10070	-2.0213	0.0039	0.5961	0.5172	0.5693	0.5960	0.6502	0.5692	-0.6264	0.6184	1.5593	0.0498	-2.2138	0.0009	0.5060	0.7555	1.5135	0.0407	1.2071	0.2287	-1.9048	0.0210	-1.7961	0.0121	-0.0855	0.9655	1.2113	0.0767
AT1G10140	-2.2929	0.0000	-0.2804	0.6924	-0.1245	0.8953	0.1773	0.8571	-0.4659	0.5974	0.8424	0.1498	-1.8643	0.0001	-0.0229	0.9924	0.3981	0.5028	-0.0600	0.9712	-2.4456	0.0000	-1.9838	0.0000	0.5181	0.5407	0.9657	0.0463
AT1G10155	-2.1441	0.0012	-1.4361	0.0503	-1.1211	0.2022	-1.2218	0.1908	-0.3048	0.8371	-0.8911	0.2649	-1.4445	0.0282	-1.2347	0.1711	-1.1603	0.1078	0.2374	0.8957	-0.1297	0.9325	-1.9546	0.0036	-1.3309	0.1092	-1.7931	0.0061
AT1G10340	-2.6751	0.0000	-2.4275	0.0000	-1.0764	0.0375	-2.2422	0.0000	-0.8386	0.1428	-0.9481	0.0594	-2.7267	0.0000	-2.5006	0.0000	-2.3932	0.0000	-2.2098	0.0000	-1.8088	0.0001	-3.1284	0.0000	-2.2677	0.0000	-1.0631	0.0129
AT1G10370	-0.0893	0.8951	-0.5094	0.3880	-0.6957	0.2740	-0.0060	0.9973	-1.2344	0.0199	-1.6119	0.0016	-0.0406	0.9516	-1.6219	0.0011	-1.4778	0.0019	-0.8869	0.1687	-0.2766	0.7448	-0.0239	0.9775	-0.4493	0.5812	-2.1185	0.0000
AT1G10540	1.0849	0.0597	1.2859	0.0324	1.2211	0.0736	0.7983	0.3351	1.6438	0.0053	0.4446	0.5674	2.2522	0.0000	0.4087	0.7596	0.2519	0.7483	0.8173	0.3596	1.3671	0.0441	1.3440	0.0162	0.8577	0.2990	0.4553	0.4756
AT1G10585	-2.0028	0.0033	-1.1400	0.1495	-1.0469	0.2541	1.6452	0.0633	-0.8228	0.4390	-0.3606	0.6957	-2.2297	0.0006	-0.9612	0.3734	0.3131	0.7203	0.0782	0.9726	-2.0823	0.0076	-2.1057	0.0019	-2.0484	0.0055	-0.9854	0.1443
AT1G10760	1.1754	0.0049	0.6742	0.1677	1.0833	0.0238	0.5310	0.4067	1.3699	0.0017	0.2430	0.6622	1.4203	0.0003	0.9403	0.0657	0.8295	0.0619	1.0819	0.0322	-0.5007	0.4251	1.6503	0.0000	2.6678	0.0000	1.1476	0.0044
AT1G10770	1.4763	0.0454	0.1123	0.9248	0.4911	0.6744	-0.0551	0.9748	1.3964	0.1296	0.2096	NA	2.2987	0.0005	0.4665	0.7916	0.2847	0.7687	0.5465	0.7198	-0.0445	0.9831	2.7575	0.0000	2.2175	0.0033	0.7717	0.3004
AT1G10990	-0.6494	0.2452	-2.1619	0.0000	-1.3477	0.0205	-0.9723	0.1671	-0.4696	0.5980	-1.2897	0.0245	-1.3925	0.0044	-0.0433	0.9825	-1.7521	0.0007	-0.5816	0.5073	0.1432	0.8976	-0.7738	0.1646	-0.7633	0.2841	-0.8930	0.0725
AT1G11000	-1.2880	0.0000	-0.6481	0.0225	-0.3242	0.3862	-0.5991	0.0859	-0.5830	0.0717	-0.3918	0.2158	-2.3013	0.0000	-0.4190	0.2767	-0.4451	0.1220	-0.7307	0.0204	-0.1774	0.7029	-1.4147	0.0000	-0.2581	0.5734	-0.3593	0.1782
AT1G11070	0.8608	0.1905	2.7975	0.0000	2.7387	0.0000	1.5867	0.0371	1.1037	0.1640	1.4784	0.0290	1.4045	0.0170	0.6918	0.5229	1.6561	0.0072	1.2799	0.1040	-1.0024	0.2506	-0.0835	0.9324	1.6787	0.0107	0.5464	0.3840
AT1G11100	-1.5477	0.0022	-1.9480	0.0002	-1.3288	0.0247	-0.5408	0.4852	-1.8917	0.0005	-0.7136	0.2373	-2.1063	0.0000	-1.4007	0.0147	-0.8892	0.1035	-0.8238	0.2614	-1.1025	0.0893	-1.5699	0.0019	-1.4790	0.0098	-1.7359	0.0008
AT1G11175	0.2404	0.6566	0.8814	0.0584	0.7274	0.1889	0.7698	0.1938	0.4898	0.4655	1.3871	0.0025	0.3200	0.5156	0.4984	0.5026	1.0675	0.0147	1.0574	0.0400	0.3474	0.6219	0.2524	0.6510	0.4652	0.5052	2.0178	0.0000
AT1G11210	-0.4972	0.4997	1.8928	0.0022	2.1142	0.0010	1.1029	0.1998	3.2838	0.0000	1.8074	0.0074	0.6942	0.2960	2.2025	0.0005	1.4701	0.0209	1.8875	0.0065	0.9230	0.2855	0.9512	0.1561	3.7017	0.0000	2.7721	0.0000
AT1G11280	-0.0471	0.9053	-6.2525	0.0000	-6.5660	0.0000	0.0363	0.9526	-6.6025	0.0000	-0.2415	0.4889	-7.3307	0.0000	0.1904	0.7642	-0.1147	0.7472	0.1490	0.8133	0.5629	0.1067	-0.0537	0.8977	0.4122	0.2767	-0.4443	0.1008
AT1G11330	-0.5858	0.4004	-2.2742	0.0001	-1.2362	0.0959	-0.7995	0.3789	-0.4654	0.6818	-1.3809	0.0415	-0.8974	0.1514	-0.4597	0.7312	-1.8056	0.0030	-0.2307	0.8859	0.1520	0.9106	-0.7862	0.2471	-1.0342	0.1902	-1.4867	0.0103
AT1G11362	0.1562	0.8848	0.5075	0.6180	0.1531	0.9144	1.5441	0.1231	0.4458	0.7694	2.7409	0.0005	-0.0578	0.9563	1.5026	0.1140	2.0709	0.0061	2.5352	0.0019	-0.1805	0.9142	0.2343	0.8305	0.5189	0.7218	2.4778	0.0004
AT1G11370	0.0231	0.9845	2.1259	0.0041	2.0004	0.0144	-0.7987	0.4920	2.5312	0.0006	0.1875	NA	2.0981	0.0023	-0.7598	0.5882	0.7329	0.3911	-0.3771	0.8285	-0.5516	0.6591	-0.5220	0.5810	-0.5005	0.7299	-0.1539	0.8557
AT1G11600	2.4263	0.0000	1.5168	0.0000	0.8514	0.0429	1.0224	0.0205	1.6062	0.0000	0.1774	0.7235	2.2206	0.0000	1.2160	0.0012	0.8982	0.0154	0.5904	0.2596	1.6286	0.0000	2.4678	0.0000	1.4396	0.0000	0.4473	0.2196
AT1G12010	0.5092	0.2545	-4.3665	0.0000	-4.1463	0.0000	-0.0775	0.9307	-4.3202	0.0000	0.3750	0.4699	-4.9660	0.0000	0.4276	0.5712	0.3516	0.4643	0.0445	0.9738	-0.0039	1.0000	0.3549	0.4727	0.1276	0.9002	0.3630	0.3920
AT1G12030	3.2935	0.0000	0.0774	0.9481	0.4161	0.7143	0.5869	0.6057	0.7697	0.4938	0.1959	0.8532	3.1827	0.0000	1.1373	0.2437	0.2727	0.7687	0.1706	0.9359	1.2976	0.1559	4.5785	0.0000	0.4748	0.7361	0.0585	0.9453
AT1G12080	-2.2167	0.0002	0.9157	0.2007	0.9272	0.2530	-0.2721	0.8117	0.7960	0.3829	0.3012	0.7145	-1.0767	0.0795	-0.1061	0.9608	0.4831	0.4999	0.4342	0.7324	-1.2591	0.1055	-2.0960	0.0004	0.4667	0.6858	0.8585	0.1500
AT1G12160	-0.8482	0.1755	-2.5899	0.0000	-2.8417	0.0000	-0.5107	0.5857	-2.0551	0.0014	-0.9183	0.1882	-2.9723	0.0000	-0.3327	0.8194	-1.0989	0.0833	0.1429	0.9322	-0.6970	0.4256	-1.0966	0.0765	-0.2264	0.8667	0.0286	0.9677
AT1G12340	1.0140	0.1347	5.5661	0.0000	5.2787	0.0000	0.9409	0.3101	5.3830	0.0000	0.4248	0.6338	4.7276	0.0000	0.7776	0.4804	0.5266	0.5076	0.2624	0.8834	0.8931	0.3478	0.0933	0.9306	1.5037	0.0412	-0.0035	0.9972
AT1G12370	-0.8232	0.1330	-1.3465	0.0133	-1.6381	0.0038	-0.9267	0.1986	-2.4240	0.0000	-1.2240	0.0362	-1.8420	0.0001	-1.3912	0.0175	-1.6034	0.0024	-0.7468	0.3472	-0.6160	0.4230	-0.4849	0.4301	-0.7062	0.3504	-2.1713	0.0000
AT1G12390	-0.8118	0.0077	-3.1804	0.0000	-3.7849	0.0000	-0.1210	0.8316	-3.8106	0.0000	0.2057	0.6000	-3.8517	0.0000	-0.3333	0.5426	0.2275	0.5261	0.2785	0.6279	-0.4463	0.3012	-0.7713	0.0126	-0.3062	0.5558	-0.2193	0.4934
AT1G12400	-0.4184	0.4429	-2.6989	0.0000	-2.6445	0.0000	-0.2473	0.7673	-1.9644	0.0000	-0.4050	0.4950	-2.6155	0.0000	-0.5135	0.5411	-0.4210	0.4394	-0.0811	0.9567	0.1499	0.8785	-0.5400	0.3134	-0.3694	0.6746	0.1756	0.7327
AT1G12520	-2.8132	0.0000	0.1413	0.6575	0.0628	0.8808	-0.1390	0.7297	-0.2741	0.4430	-0.1364	0.6528	-2.9444	0.0000	-0.3502	0.3091	-0.0280	0.9314	-0.5558	0.0496	-0.2202	0.5439	-2.5260	0.0000	-0.4504	0.1134	-0.6714	0.0023
AT1G12610	0.4690	0.6146	2.2747	0.0024	1.0791	0.2853	1.1169	0.3049	-0.0040	1.0000	0.5570	0.5606	0.7010	0.3986	0.1486	0.9538	0.1162	0.9107	0.4468	0.7890	4.5748	0.0000	0.2115	0.8493	0.0726	0.9747	0.5501	0.4792
AT1G12620	-0.4334	0.3250	-1.6961	0.0000	-2.0381	0.0000	-0.6483	0.2489	-1.9802	0.0000	-0.3820	0.4388	-2.3031	0.0000	-0.0526	0.9698	-0.5105	0.2433	-0.0791	0.9494	0.0627	0.9456	-0.2946	0.5470	-0.1597	0.8585	-0.7631	0.0483
AT1G12710	1.0911	0.0086	1.8123	0.0000	1.9090	0.0000	0.8595	0.1249	2.2148	0.0000	1.1840	0.0101	2.2066	0.0000	1.1851	0.0099	1.4675	0.0004	1.6525	0.0002	-0.0316	0.9778	1.2715	0.0018	1.3158	0.0026	1.6910	0.0000
AT1G12800	0.2527	0.6362	-0.3107	0.5854	-0.2571	0.7050	-0.3534	0.6114	-0.3478	0.6557	-0.7275	0.1386	0.1010	0.8585	0.0539	0.9720	-0.7269	0.1097	-0.8929	0.1056	-0.3184	0.6581	0.3381	0.5196	-0.3621	0.6387	-2.1123	0.0000
AT1G12845	1.0368	0.0821	0.8706	0.1947	0.4112	0.6442	0.6732	0.4462	1.7653	0.0033	0.0719	0.9360	0.7991	0.1798	1.1882	0.0977	0.3148	0.6601	0.1933	0.9037	-0.2085	0.8597	1.2554	0.0332	2.1589	0.0002	1.6869	0.0020
AT1G13110	0.5779	0.4215	-2.0489	0.0008	-2.0285	0.0072	-0.2922	0.7978	0.2125	0.8829	-0.5524	0.4785	0.2405	0.7605	0.0590	0.9795	-1.4191	0.0272	0.2479	0.8775	0.5746	0.5563	0.4099	0.6067	-0.3880	0.7562	-0.3364	0.6124
AT1G13470	-1.9661	0.0084	-2.0662	0.0071	-0.5075	0.6643	-1.6192	0.1028	0.7170	0.5774	-1.8557	0.0256	-1.0158	0.1953	-1.9282	0.0238	-2.6370	0.0004	-1.6503	0.0816	-1.6878	0.0676	-2.0963	0.0049	-1.3820	0.1421	-1.7735	0.0132
AT1G13480	-2.1984	0.0008	-3.7088	0.0000	-3.7923	0.0000	-1.4619	0.0978	-3.6110	0.0000	-1.9439	0.0097	-4.1208	0.0000	-2.3082	0.0012	-2.5169	0.0002	-1.1543	0.2191	-2.4482	0.0009	-2.5267	0.0001	-2.1396	0.0029	-2.0916	0.0014
AT1G13550	-2.7243	0.0001	-2.2359	0.0025	-1.0085	0.3090	-1.9460	0.0331	-0.2739	0.8683	-2.1356	NA	-1.7869	0.0110	-2.2648	0.0042	-2.6518	0.0003	-2.2139	0.0078	-1.8333	0.0358	-2.0787	0.0042	-1.9375	0.0163	-2.3362	0.0008
AT1G13608	1.2235	0.0960	-0.2467	0.8079	-0.2321	0.8435	0.2862	0.8256	-0.0461	0.9839	-0.1917	NA	0.9935	0.1751	0.2097	0.9191	-0.2185	NA	-0.0703	0.9754	0.2922	0.8286	2.9772	0.0000	-0.1995	0.9037	0.2694	NA
AT1G13609	3.3711	0.0000	-0.7673	0.4154	-0.7967	0.4637	0.4493	0.7311	-0.8069	0.5046	-0.7503	0.4169	2.3001	0.0012	-0.2288	0.9187	-0.4538	0.6212	0.0011	1.0000	-0.1879	0.9106	5.3372	0.0000	-0.7559	0.5484	-0.6926	0.3603
AT1G13650	-0.2520	0.7866	-1.4144	0.0622	-1.5201	0.0667	-0.1768	0.8999	-1.2039	0.1898	-1.9407	0.0112	-0.0730	0.9395	-1.4291	0.1019	-2.4023	0.0005	-1.7113	0.0416	-0.0022	1.0000	0.3722	0.6870	-1.0122	0.2988	-3.2818	0.0000
AT1G13750	-0.6755	0.2849	-2.8162	0.0000	-1.7415	0.0050	-0.9157	0.2618	-0.4386	0.6812	-1.2109	0.0600	-1.8918	0.0004	-0.5696	0.5994	-1.7960	0.0018	-1.0165	0.2004	-0.4503	0.6340	-0.6951	0.2827	-0.5907	0.5348	-0.9034	0.1036
AT1G13830	-1.3455	0.0625	-1.6930	0.0233	-1.0626	0.2541	-1.3281	0.1671	0.2927	0.8504	-1.5092	0.0579	-0.7805	0.2941	-0.9343	0.4029	-2.0965	0.0034	-0.6673	0.6083	0.0442	0.9816	-1.4492	0.0463	-1.0798	0.2603	0.5528	0.4360
AT1G13930	0.9341	0.0599	0.1745	0.8087	-0.2544	0.7474	0.6161	0.3962	0.3211	0.7336	0.2492	0.6990	0.3239	0.5670	0.9396	0.1302	1.0133	0.0436	1.2372	0.0311	-0.7790	0.2369	1.8327	0.0001	2.1681	0.0000	1.5397	0.0007
AT1G14240	1.7602	0.0114	0.4701	0.6273	0.2304	0.8545	0.9614	0.3553	0.7253	0.5367	1.0120	0.2240	0.6994	0.3627	1.2186	0.2063	0.8005	0.3143	2.0715	0.0086	1.0560	0.2844	1.4598	0.0438	0.8117	0.4715	0.4143	0.5884
AT1G14250	0.7395	0.3407	-2.3919	0.0004	-1.0896	0.2291	0.1633	0.9081	-1.3102	0.1360	-1.9204	0.0116	-0.1791	0.8451	-0.6690	0.6165	-1.7079	0.0160	-0.5796	0.6660	0.5656	0.6166	0.8287	0.2905	0.4672	0.7303	-2.6251	0.0001
AT1G14345	-0.0261	0.9658	-0.7191	0.0995	-1.0100	0.0224	-0.5764	0.3098	-1.4169	0.0003	-1.5999	0.0001	-0.1612	0.7427	-0.9033	0.0517	-1.7545	0.0000	-1.3462	0.0016	0.0208	0.9848	-0.1006	0.8632	-0.6203	0.2373	-2.0984	0.0000
AT1G14370	-1.6840	0.0004	-2.2108	0.0000	-1.1419	0.0465	-1.0636	0.1012	-0.4670	0.5788	-0.9379	0.0930	-2.0259	0.0000	-0.8176	0.2353	-1.7922	0.0002	-1.0094	0.1147	-0.2576	0.7763	-1.8795	0.0001	-0.8118	0.2097	-0.8912	0.0592
AT1G14540	-3.9968	0.0000	-2.9746	0.0000	-1.8019	0.0016	-2.2410	0.0002	-2.1728	0.0000	-2.1107	0.0003	-3.5687	0.0000	-3.1396	0.0000	-3.7164	0.0000	-2.8947	0.0000	-1.9204	0.0009	-4.1035	0.0000	-3.5368	0.0000	-2.9529	0.0000
AT1G14870	-2.1729	0.0000	-2.1125	0.0000	-1.3747	0.0028	-1.0592	0.0552	-2.0097	0.0000	-0.4049	0.4504	-2.5389	0.0000	-1.8508	0.0000	-1.1361	0.0097	-0.7455	0.2183	-1.6178	0.0004	-2.4781	0.0000	-2.2440	0.0000	-0.3161	0.4794
AT1G14880	-1.3518	0.0807	-2.0806	0.0061	-1.2189	0.2093	-1.4785	0.1394	-0.0281	0.9924	-1.7188	0.0379	-0.7563	0.3530	-1.5660	0.0897	-2.0516	0.0064	-0.9315	0.4445	-0.5669	0.6497	-1.1582	0.1505	-1.2357	0.2065	-0.2639	0.7477
AT1G15050	-1.0596	0.1594	2.0909	0.0014	2.1962	0.0015	0.7181	0.4920	0.6307	0.5917	0.9362	0.2420	-0.9490	0.1878	0.4464	0.7795	0.8496	0.2557	0.4322	0.7708	-0.1843	0.9048	-0.4921	0.5733	1.9253	0.0064	0.0679	0.9345
AT1G15125	-1.8086	0.0001	-3.3878	0.0000	-1.6010	0.0012	-0.6675	0.3386	-0.1803	0.8669	-0.9659	0.0705	-1.6396	0.0002	-0.1669	0.9031	-1.7181	0.0002	-0.7227	0.3055	-1.0583	0.0708	-1.4832	0.0014	-1.0425	0.0590	0.5732	0.2208
AT1G15150	1.8159	0.0088	-1.0339	0.2374	-0.5355	0.6357	-0.0007	1.0000	-0.3580	0.8213	-0.8092	NA	1.6092	0.0169	-0.2636	0.8997	-0.8706	0.2896	-1.5228	0.1147	-0.1729	0.9149	2.2917	0.0006	-0.2668	0.8757	-0.8977	0.2194
AT1G15160	-0.8622	0.0773	-0.3709	0.5463	-0.4528	0.5132	-0.6033	0.3921	-0.6540	0.3388	-0.6888	0.2153	-0.9172	0.0470	-0.3825	0.7019	-0.5771	0.2699	-0.3743	0.6882	0.7798	0.2140	-0.7703	0.1270	-0.3299	0.7161	-2.1119	0.0000
AT1G15415	-2.1929	0.0024	-1.4930	0.0627	-1.4976	0.0955	-0.2781	0.8393	-1.6526	0.0578	-0.4191	NA	-2.1075	0.0024	-1.0797	0.3258	-0.9330	0.2472	-0.7060	0.6005	-1.5534	0.0937	-1.6506	0.0281	-2.0662	0.0093	-1.8524	0.0085
AT1G15520	-2.5164	0.0000	-2.4312	0.0000	-1.2609	0.0946	-0.0152	0.9929	-1.9858	0.0019	-1.1849	0.0913	-2.0724	0.0003	-2.4892	0.0001	-2.2583	0.0002	-2.1814	0.0011	-2.8306	0.0000	-2.2731	0.0001	-2.4433	0.0001	-2.0299	0.0005
AT1G15580	-0.7945	0.1893	0.6067	0.3729	0.6088	0.4406	-0.0542	0.9659	0.1373	0.9211	0.5625	0.4076	-0.4413	0.4819	0.0514	0.9799	0.0537	0.9470	-1.1923	0.1108	-0.0086	0.9993	-0.6915	0.2747	0.6666	0.4320	-2.2512	0.0001
AT1G15790	-1.8327	0.0002	-3.4966	0.0000	-2.4012	0.0000	-1.5136	0.0136	-0.8427	0.2124	-1.1793	0.0367	-2.9563	0.0000	-1.5240	0.0055	-1.8799	0.0002	-1.0766	0.0972	-0.9093	0.1764	-1.9731	0.0000	-1.5492	0.0039	-0.8905	0.0682
AT1G15870	0.2063	0.7544	2.2029	0.0000	1.7286	0.0004	0.6135	0.4084	2.3346	0.0000	-0.1699	0.8212	1.8405	0.0000	-0.3229	0.7924	-0.4746	0.4402	-0.0345	0.9826	0.7100	0.3113	0.6659	0.2193	-0.0372	0.9827	-0.8032	0.1403
AT1G15885	-0.3444	0.2713	-5.1923	0.0000	-5.1047	0.0000	-0.4459	0.2764	-5.1397	0.0000	0.0783	0.8532	-5.8245	0.0000	-0.3595	0.4475	0.1596	0.6495	0.1470	0.8226	-0.2915	0.5158	-0.1863	0.6064	-0.4610	0.2496	-0.1225	0.7043

AT1G15890	-0.6350	0.2287	-5.0117	0.0000	-4.9444	0.0000	-0.6278	0.3887	-4.9714	0.0000	0.3350	0.5882	-5.5831	0.0000	-0.5167	0.5584	-0.8866	0.0876	-0.6616	0.3876	-0.1923	0.8422	-0.6864	0.1984	-0.5071	0.5302	-0.0977	0.8608
AT1G15980	-0.1606	0.8094	-0.8917	0.1034	-0.4554	0.5301	-0.6855	0.3404	-0.2909	0.7693	-1.4694	0.0059	-0.0281	0.9678	-0.6056	0.4595	-1.7852	0.0002	-1.2358	0.0348	-0.3717	0.6505	-0.3341	0.5911	-1.0067	0.0856	-3.3871	0.0000
AT1G16090	-2.0214	0.0000	-1.4567	0.0041	-1.3333	0.0175	-1.0027	0.1337	-1.2297	0.0313	-1.8532	0.0006	-1.7801	0.0001	-1.3993	0.0114	-1.7357	0.0005	-1.0822	0.0880	-0.4418	0.5774	-1.5405	0.0016	-1.4170	0.0086	-1.7587	0.0002
AT1G16130	-0.6701	0.1303	-1.7075	0.0000	-0.2448	0.7145	-0.9931	0.0683	-1.0704	0.0247	0.0341	0.9599	-2.0755	0.0000	-1.5769	0.0003	-0.9842	0.0244	-1.1374	0.0213	-0.4609	0.4659	-1.7133	0.0000	-1.7335	0.0000	-0.8607	0.0342
AT1G16260	-1.3429	0.0056	-4.4916	0.0000	-3.1319	0.0000	-1.6158	0.0051	-2.3117	0.0000	-1.4610	0.0063	-3.1477	0.0000	-1.7093	0.0008	-2.0341	0.0000	-1.2400	0.0346	-0.3058	0.7240	-1.6309	0.0006	-1.6129	0.0015	-1.7490	0.0002
AT1G16370	-1.3773	0.0074	-1.6601	0.0017	-1.4899	0.0109	-0.5698	0.4683	-2.9492	0.0000	-0.3494	0.5949	-0.7040	0.1769	-0.8456	0.2413	-0.9670	0.0783	-0.7476	0.3385	0.1779	0.8635	-1.8159	0.0003	-1.0108	0.1165	0.0018	0.9983
AT1G16390	0.2346	0.7580	-3.5704	0.0000	-2.4693	0.0000	-1.2994	0.0844	-2.7258	0.0000	-1.7064	0.0068	-1.3693	0.0138	-1.2944	0.0613	-2.9217	0.0000	-1.1298	0.1390	0.2418	0.8314	-0.1577	0.8536	-1.0023	0.1759	-1.3009	0.0182
AT1G16410	0.8034	0.3404	-2.0486	0.0076	-1.5946	0.0767	0.3179	0.8208	-0.6963	0.5914	-1.8240	0.0281	0.0009	1.0000	0.1960	0.9324	-1.6175	0.0379	-0.2323	0.9120	-0.0273	0.9902	1.3434	0.0903	-0.3841	0.8118	-1.6844	0.0184
AT1G16420	-3.1721	0.0000	-3.8040	0.0000	-2.4050	0.0017	-1.4686	0.1316	-2.5063	0.0007	-2.8319	0.0002	-3.3399	0.0000	-3.2152	0.0000	-3.6610	0.0000	-1.9443	0.0223	-1.7758	0.0411	-3.7031	0.0000	-3.2633	0.0000	-3.3675	0.0000
AT1G16630	0.7396	0.2243	0.1202	0.8922	-0.2749	0.7675	-0.1815	0.8699	0.4643	0.6496	-0.7261	0.2808	0.5306	0.3906	0.6373	0.5195	-0.8078	0.1883	-0.1581	0.9218	0.0648	0.9622	0.7340	0.2403	-0.3958	0.7159	-2.0729	0.0002
AT1G16730	-1.2820	0.0322	-1.5805	0.0098	-1.6722	0.0110	-0.8272	0.3379	-1.9580	0.0014	-1.7445	0.0077	-1.2382	0.0314	-1.8302	0.0042	-2.0345	0.0006	-0.5885	0.5808	-0.1092	0.9353	-0.8725	0.1738	-1.7008	0.0077	-2.1026	0.0002
AT1G16850	-0.7810	0.3333	1.7256	0.0222	1.0229	0.2904	0.4621	0.7068	0.6407	0.6119	1.7090	0.0316	-0.9374	0.2142	0.9876	0.3814	2.2486	0.0016	1.7490	0.0441	0.2444	0.8693	1.8094	0.0122	2.5945	0.0003	2.3218	0.0006
AT1G17020	1.2464	0.0706	0.0188	0.9875	-0.5594	0.5848	0.6043	0.5751	0.8529	0.3955	0.0854	0.9347	0.5824	0.4354	-0.4364	0.7860	0.9103	0.2128	1.3996	0.1036	0.2873	0.8278	2.1263	0.0010	-0.0187	0.9961	1.1498	0.0745
AT1G17050	-0.9820	0.0781	-1.0637	0.0712	-1.0482	0.1152	-0.9328	0.2155	-1.2814	0.0388	-2.0310	0.0004	-0.7572	0.1737	-1.5467	0.0086	-2.1552	0.0000	-0.7248	0.3921	-0.3203	0.7380	-0.9378	0.0998	-1.2342	0.0452	-3.0540	0.0000
AT1G17147	-1.2476	0.0253	-2.4445	0.0000	-1.4921	0.0164	-0.3194	0.7394	-1.4269	0.0218	-0.1901	0.8059	-2.1163	0.0000	-0.8482	0.2951	-0.8916	0.1315	-1.0166	0.1790	-0.9386	0.2086	-1.3343	0.0171	-0.1103	0.9365	-0.0577	0.9288
AT1G17180	0.3063	0.7585	0.0879	0.9427	-0.2617	0.8410	3.3412	0.0000	1.0860	0.3093	-0.4981	0.6058	-0.0450	0.9664	0.1810	0.9402	-0.5598	0.5279	0.3290	0.8584	0.4057	0.7674	0.6469	0.4766	-0.0125	0.9984	0.0580	0.9479
AT1G17220	-0.7015	0.1509	-0.9779	0.0480	-0.6512	0.2928	-1.0106	0.0937	-0.8334	0.1592	-1.1539	0.0226	-0.7393	0.1130	-0.6346	0.3777	-1.3497	0.0033	-0.9015	0.1379	-0.4173	0.5637	-0.5407	0.3013	-0.5098	0.4874	-2.0814	0.0000
AT1G17240	-1.4823	0.0039	-3.0832	0.0000	-2.9170	0.0000	-0.9278	0.1879	-2.9438	0.0000	-1.1792	0.0482	-3.1718	0.0000	-1.1362	0.0693	-0.7469	0.1823	-1.1709	0.0740	-0.5006	0.5282	-1.2161	0.0203	-1.1101	0.0776	-1.0109	0.0480
AT1G17255	0.0775	0.9213	-1.6302	0.0079	-0.8799	0.2466	-0.0264	0.9855	-2.0136	0.0015	-0.3279	0.6567	-1.7314	0.0012	-0.1296	0.9408	-0.6217	0.3309	-0.0314	0.9841	0.2191	0.8389	0.3423	0.6166	0.4190	0.6807	-0.2990	0.6233
AT1G17360	-0.9472	0.0399	-0.6813	0.1978	-0.6899	0.2501	-0.8294	0.1840	-0.8041	0.1739	-0.9592	0.0590	-1.3494	0.0016	-1.0660	0.0453	-1.1864	0.0099	-0.3170	0.7373	0.2024	0.8169	-0.9294	0.0478	-1.1159	0.0277	-2.0329	0.0000
AT1G17380	-1.3838	0.0171	-1.0590	0.1011	-1.3033	0.0569	0.0180	0.9912	-1.6206	0.0100	-0.9421	0.1591	-2.0445	0.0001	-1.3722	0.0434	-1.2549	0.0373	-0.7467	0.4206	0.9088	0.2481	-2.0100	0.0003	-1.8076	0.0031	-1.3968	0.0119
AT1G17460	1.2900	0.0049	1.0116	0.0462	0.8862	0.1369	1.0497	0.0863	1.8481	0.0000	0.7632	0.1707	1.1933	0.0068	1.7727	0.0001	1.1423	0.0183	1.4455	0.0056	0.8145	0.2036	2.0142	0.0000	1.6188	0.0006	1.6594	0.0002
AT1G17600	-0.6130	0.3364	-2.2846	0.0000	-1.4318	0.0285	-1.5367	0.0309	-1.0504	0.1528	-1.7255	0.0060	-0.9995	0.0795	-1.7526	0.0045	-2.6098	0.0000	-1.4456	0.0348	-0.4575	0.6245	-1.2144	0.0391	-1.8129	0.0026	-2.2799	0.0000
AT1G17665	0.6829	0.3572	0.9902	0.1970	1.7941	0.0129	0.3971	0.7289	2.5314	0.0001	0.7913	0.3173	2.1901	0.0003	2.0295	0.0034	1.4690	0.0299	2.4805	0.0003	0.1539	0.9179	1.9006	0.0029	0.8765	0.3656	1.3919	0.0264
AT1G18020	-0.3988	0.6705	-1.6497	0.0368	-1.3447	0.1508	-1.2446	0.2331	0.6435	0.6247	0.0031	0.9982	-2.3457	0.0007	-1.1703	0.2729	-1.0910	0.1715	-1.6091	0.0857	-1.8841	0.0309	-0.8792	0.2955	-1.6156	0.0607	-1.6930	0.0162
AT1G18193	-0.4940	0.5917	3.8034	0.0000	3.6822	0.0000	-0.5629	0.6520	4.8728	0.0000	-0.2066	0.8507	4.2973	0.0000	-0.2855	0.8940	-0.2701	0.7823	-0.3506	0.8454	-0.2842	0.8500	-0.4967	0.6054	0.8161	0.4994	-0.1398	0.8706
AT1G18200	-0.2633	0.6987	-1.4470	0.0099	-0.0525	0.9620	-0.6010	0.4651	0.7496	0.3196	0.1997	0.7861	-0.9585	0.0704	-0.1838	0.9041	-0.8290	0.1519	-0.1107	0.9471	2.1579	0.0000	-0.9794	0.0822	-0.5618	0.5267	0.1240	0.8375
AT1G18300	-1.0789	0.0551	-1.1008	0.0669	-1.4499	0.0187	-0.4421	0.6212	-1.2946	0.0420	-1.0919	0.0785	-1.3696	0.0093	-1.6403	0.0060	-1.6923	0.0021	-1.2768	0.0600	2.1577	0.0001	-1.1529	0.0419	-1.4066	0.0206	-0.8110	0.1287
AT1G18570	-1.4747	0.0128	-2.8005	0.0000	-1.1552	0.1157	-1.2722	0.1045	-0.2563	0.8466	-1.5272	0.0194	-1.6844	0.0028	-0.9385	0.2729	-2.3229	0.0001	-1.5253	0.0305	0.0759	0.9572	-1.7835	0.0022	-1.2071	0.0902	-0.8905	0.1218
AT1G18740	-0.3449	0.5285	-0.2283	0.7267	-0.2106	0.7822	-0.1448	0.8729	-0.4094	0.6102	-0.7553	0.1533	-0.6197	0.1929	-0.7240	0.2729	-0.5670	0.2619	-0.3300	0.7265	2.4413	0.0000	-0.5930	0.2451	-0.4049	0.6169	0.7273	0.0988
AT1G18870	0.5749	0.2336	-0.4559	0.4273	0.0376	0.9688	0.0853	0.9292	0.2727	0.7628	0.3491	0.5412	-0.3972	0.4247	0.5668	0.4366	0.2325	0.6788	-0.1441	0.9088	0.3237	0.6727	0.7753	0.0983	2.6101	0.0000	0.9551	0.0249
AT1G19020	-1.8314	0.0001	-2.1786	0.0000	-1.1298	0.0550	-0.8687	0.2146	-1.2910	0.0208	-0.4814	0.4365	-2.6561	0.0000	-1.8412	0.0003	-1.6629	0.0008	-1.6121	0.0036	-0.6550	0.3633	-2.3657	0.0000	-2.1020	0.0000	-1.4742	0.0019
AT1G19040	0.0000	1.0000	1.1053	0.1939	0.8413	0.4141	1.3605	0.1762	0.0000	1.0000	1.1616	NA	-0.0051	0.9971	0.2015	0.9268	2.1723	0.0030	0.9682	0.4029	3.4188	0.0000	0.0419	0.9735	0.0000	1.0000	0.0000	1.0000
AT1G19050	-0.0476	0.9609	1.8594	0.0033	0.8208	0.3436	1.2603	0.1359	-1.5905	0.0262	1.2844	0.0699	0.5115	0.4750	0.7558	0.4815	2.2909	0.0002	1.4257	0.0699	1.6225	0.0284	0.3083	0.7173	-0.8721	0.3376	0.1040	0.8862
AT1G19080	-0.4463	0.2276	-2.5201	0.0000	-2.2233	0.0000	-0.1831	0.7619	-1.4885	0.0000	0.0333	0.9515	-2.7631	0.0000	0.1135	0.9081	0.0464	0.9218	0.0119	0.9912	0.0682	0.9291	-0.2747	0.5080	-0.0132	0.9917	-0.8677	0.0108
AT1G19150	1.0282	0.0364	-0.7657	0.1711	-0.8709	0.1563	-0.0354	0.9750	-0.7287	0.2808	-1.4691	0.0054	0.9917	0.0370	-0.3707	0.7267	-1.5798	0.0010	-1.2694	0.0263	0.3688	0.6483	0.9375	0.0637	-0.4650	0.5734	-2.3110	0.0000
AT1G19180	-1.6202	0.0000	-1.0758	0.0008	-0.5085	0.2357	-0.1427	0.8154	-1.1641	0.0004	-0.4996	0.1881	-2.2351	0.0000	-1.1707	0.0005	-0.7604	0.0241	-1.2510	0.0003	0.0187	0.9840	-2.2190	0.0000	-1.0919	0.0012	-1.4713	0.0000
AT1G19210	0.1826	0.8614	-0.0280	0.9828	-0.0589	0.9704	0.7558	0.5123	0.2830	0.8667	-0.1701	0.8789	-0.5218	0.5444	0.0539	0.9867	-0.1439	0.8883	-0.1270	0.9578	3.3954	0.0000	-0.5906	0.5206	-0.4586	0.7626	1.0679	0.1378
AT1G19250	-1.9998	0.0073	-1.7612	0.0267	-2.1597	0.0087	-0.1648	0.9148	-1.5401	0.0942	-1.5094	0.0744	-1.2163	0.1113	-2.5272	0.0013	-1.8268	0.0177	-1.0584	0.3611	-1.8834	0.0343	-1.9261	0.0108	-1.8834	0.0239	-0.8811	0.2355
AT1G19320	-1.9436	0.0011	-1.7158	0.0061	-0.3219	0.7426	-2.2223	0.0016	-0.5027	0.6457	-0.9692	0.1691	-1.3021	0.0264	-0.6285	0.5694	-2.7559	0.0000	-2.1624	0.0013	-1.5990	0.0263	-2.1234	0.0003	-1.5628	0.0218	-2.0404	0.0006
AT1G19450	-0.6967	0.1270	-0.4753	0.3747	-0.1497	0.8416	-0.8617	0.1381	-0.3229	0.6917	-1.7425	0.0001	0.1730	0.7505	-1.1239	0.0216	-2.2220	0.0000	-1.4653	0.0020	-0.4862	0.4536	-1.0323	0.0187	-0.9941	0.0452	-2.4657	0.0000
AT1G19490	-1.2773	0.0066	-1.2330	0.0127	-1.4450	0.0053	-1.0457	0.0930	-1.7802	0.0002	-1.1404	0.0320	-2.2352	0.0000	-1.4642	0.0043	-1.0960	0.0263	0.2990	0.7689	0.2932	0.7236	-1.8055	0.0001	-1.0486	0.0594	-1.1342	0.0134
AT1G19510	-0.5460	0.4587	-3.9957	0.0000	-2.6227	0.0000	-1.4681	0.0748	-1.8351	0.0074	-2.2433	0.0012	-2.6156	0.0000	-1.0065	0.2684	-2.5006	0.0001	-1.4243	0.0734	-0.0022	1.0000	-0.1930	0.8331	-1.3020	0.0877	-0.2987	0.6609
AT1G19530	-0.3979	0.6691	1.1696	0.1667	0.4759	0.6799	0.7481	0.5161	0.7416	0.5422	1.6268	0.0473	-0.7602	0.3417	1.2966	0.1933	1.8316	0.0145	2.2387	0.0066	-1.2703	0.1966	-0.4563	0.6305	0.6764	0.5955	1.9977	0.0041
AT1G19540	-0.7335	0.3528	-0.3064	0.7636	-0.1044	0.9406	1.1568	0.2434	-1.3672	0.1191	1.4355	0.0676	-1.7277	0.0104	1.1917	0.2182	1.6315	0.0235	1.5531	0.0773	-0.8857	0.3839	-0.6666	0.4225	0.4773	0.7266	2.0654	0.0018
AT1G19550	-0.5292	0.5617	4.5572	0.0000	4.2347	0.0000	-0.6237	0.6094	4.1346	0.0000	0.0177	0.9898	5.2310	0.0000	-0.0221	0.9947	-0.2713	0.7817	0.0337	0.9876	-0.0831	0.9635	-0.5311	0.5745	0.9415	0.3941	0.4861	0.5363
AT1G19565	-1.1837	0.0695	2.6671	0.0000	2.3218	0.0000	-0.6515	0.4942	1.9360	0.0009	-1.0647	0.1571	2.0947	0.0001	-0.9712	0.2868	-0.6230	0.3868	-0.6488	0.5611	0.1073	0.9387	-0.9795	0.1460	-0.8901	0.3356	-0.7430	0.2456
AT1G19610	-1.3646	0.0219	-0.9821	0.1450	-0.6799	0.4163	-0.1505	0.8991	-1.5194	0.0225	0.0692	0.9391	-2.4046	0.0000	-1.0840	0.1679	-0.0995	0.9021	-0.1094	0.9537	-1.3796	0.0559	-1.2946	0.0330	-0.7098	0.4357	1.6675	0.0026
AT1G19640	-1.1142	0.0618	-2.4781	0.0000	-2.0181	0.0010	0.1394	0.9057	-1.7791	0.0039	-1.3965	0.0313	-1.7023	0.0019	-1.0679	0.1652	-0.5472	0.4097	-0.2730	0.8424	-0.2344	0.8375	-1.0421	0.0881	-1.5340	0.0167	-2.2089	0.0001
AT1G19700	-1.7208	0.0002	-1.8420	0.0001	-1.5216	0.0026	-1.1560	0.0587	-1.4348	0.0044	-0.8389	0.1242	-2.2450	0.0000	-0.9757	0.1030	-1.0902	0.0268	-0.1855	0.8820	-0.2750	0.7482	-1.4698	0.0016	-0.9274	0.1120	-1.4946	0.0010
AT1G19960	-1.2224	0.1014	-2.3748	0.0007	-1.7445	0.0332	-0.7537	0.4979	-0.4063	0.7829	-2.0529	0.0086	-0.9669	0.1938	-0.9852	0.3792	-1.7464	0.0170	-0.4665	0.7624	0.1104	0.9466	-0.3466	0.7189	-0.5437	0.6860	-0.0197	0.9820
AT1G20030	2.1694	0.0000	1.6183	0.0031	1.3444	0.0312	1.5574	0.0193	0.7233	0.3723	1.2715	0.0360	1.9121	0.0001	0.9741	0.1868	1.6640	0.0022	1.5594	0.0123	-0.0044	1.0000	2.9805	0.0000	2.6432	0.0000	1.7991	0.0005
AT1G20120	-2.5955	0.0000	-0.7586	0.1244	-0.8398	0.1290	-0.5876	0.3432	-0.9950	0.0601	-0.7135	0.1677	-1.6200	0.0001	-1.8193	0.0001	-0.6966	0.1401	-0.1374	0.9064	-0.6341	0.3012	-1.8771	0.0000	-1.2623	0.0128	-1.1092	0.0134
AT1G20310	-2.2466	0.0018	-1.7557	0.0241	-1.3319	0.1525	0.4323	0.7311	-1.7724	0.0369	-0.8635	NA	-1.7705	0.0122	-1.9716	0.0167	-1.3001	0.0958	-0.6348	0.6510	-0.2351	0.8778	-1.8276	0.0138	-1.3074	0.1637	-1.5521	0.0277
AT1G20440	-0.0659	0.9423	1.8218	0.0027	1.7133	0.0101	0.6383	0.4990	1.2169	0.1034	1.6943	0.0105	-0.1197	0.8817	0.9397	0.2877	2.2008	0.0002	1.6878	0.0159	-0.4343	0.6745	-0.0948	0.9207	2.3482	0.0001	1.9685	0.0006
AT1G20450	-0.1349	0.8547	1.6643	0.0012	1.2280	0.0419	0.6400	0.4181	1.0471	0.1031	1.8028	0.0012	-0.8315	0.1167	1.1131	0.0815	2.2195	0.0000	1.5060	0.0114	0.3077	0.7414	-0.1024	0.8975	2.2613	0.0000	1.6775	0.0006
AT1G20620	0.3143	0.4811	0.7764	0.0596	0.5234	0.3086	0.7752	0.1254	0.8285	0.0667	0.7532	0.0779	0.3793	0.3605	0.7647	0.1105	0.9951	0.0096	1.0211	0.0207	0.0069	0.9982	0.1673	0.7483	1.2852	0.0009	2.3036	0.0000
AT1G20680	-1.2004	0.1303	-1.5946	0.0470	-1.4203	0.1275	-0.7833	0.5050	-1.6058	0.0727	-1.0011	NA	-2.0333	0.0044	0.1734	0.9437	-0.8239	0.3287	-0.6432	0.6574	-1.0806	0.3039	-0.9073	0.2868	-0.9077	0.4300	0.6597	0.3863
AT1G20823	-0.8072	0.2092	-0.4205	0.5974	-0.5011	0.5752	0.0285	0.9857	-0.5986	0.5448	-1.1947	0.0770	-0.7983	0.1964	-1.1993	0.1130	-0.6945	0.2949	-0.0856	0.9653	2.4997	0.0000	-0.7839	0.2356	-1.2878	0.0668	-0.4581	0.4544
AT1G21050	-2.3389	0.0000	-0.3770	0.4774	-0.2587	0.6949	-0.5908	0.3318	-0.3082	0.6938	-0.0743	0.9065	-2.3733	0.0000	-0.4727	0.5183	-0.4317	0.3598	-0.7185	0.2174	-0.2272	0.7639	-2.2055	0.0000	-0.3660	0.6195	-0.0836	0.8603
AT1G21060	-2.1085	0.0000	-0.9026	0.0679	-0.1399	0.8597	-1.0891	0.0605	-0.2794	0.7574	-0.3280	0.5688	-1.9979	0.0000	-0.4557	0.5918	-1.2114	0.0080	-1.3108	0.0111	-0.8549	0.1476	-2.0854	0.0000	-0.2743	0.7652	-1.1187	0.0093
AT1G21100	-0.7440	0.0957	-1.2733	0.0033	-0.5716	0.3295	-0.5979	0.3417	-1.5202	0.0005	0.2367	0.6739	-2.4343	0.0000	-0.5063	0.4910	-0.4421	0.3633	0.0786	0.9542	-0.5515	0.3734	-1.1639	0.0064	-0.0143	0.9942	1.1011	0.0065
AT1G21110	-1.8986	0.0000	-1.4301	0.0002	0.2716	0.6477	-1.5620	0.0003	-0.1655	0.8479	-0.4827	0.2968	-0.9216	0.0132	-1.8055	0.0000	-1.5671	0.0000	-1.4553	0.0005	-1.9099	0.0000	-2.4064	0.0000	-1.5799	0.0001	-1.3050	0.0005
AT1G21120	-2.9438	0.0000	-2.0475	0.0001	-0.9938	0.1525	-1.0449	0.1637	-1.3398	0.0328	-1.2178	0.0475	-1.8622	0.0003	-2.6336	0.0000	-2.7770	0.0000	-2.2327	0.0001	-2.0879	0.0003	-3.9728	0.0000	-1.9580	0.0004	-2.4022	0.0000
AT1G21130	-0.2766	0.4142	-2.0640	0.0000	-1.1040	0.0002	-0.3498	0.4368	-1.5637	0.0000	0.4885	0.1498	-1.7689	0.0000	-0.2282	0.7218	-0.2427	0.4794	-0.4747	0.2650	-0.9728	0.0024	-0.4777	0.1307	0.0070	0.9972	0.3321	0.2544
AT1G21140	-2.0363	0.0019	-1.1474	0.1282	-1.1110	0.1983	-1.1482	0.2176	-0.1454	0.9313	-1.5240	0.0414	-1.6778	0.0085	-0.8711	0.4171	-2.0274	0.0026	-0.2139	0.9071	-0.1365	0.9270	-2.2612	0.0005	-0.8344	0.4061	-1.2078	0.0603
AT1G21240	-1.3047	0.0966	-2.6487	0.0003	-2.0281	0.0155	-0.2431	0.8698	-1.1676	0.2595	-1.1601	0.1839	-2.0176	0.0050	-1.9188	0.0248	-2.1421	0.0046	-1.0524	0.3656	-1.1528	0.2670	-1.5911	0.0405	-1.8386	0.0291	-1.6300	0.0229
AT1G21250	-1.2359	0.1137	-2.8046	0.0001	-1.7417	0.0439	-1.2462	0.2359	-0.9233	0.4123	-1.8964	0.0206	-1.7659	0.0141	-1.5290	0.1013	-2.4590	0.0008	-0.8695	0.4892	-0.3734	0.7875	-1.4815	0.0560	-1.5184	0.0884	-1.7509	0.0134
AT1G21270	-0.4146	0.6367	-2.2554	0.0011	-0.6821	0.5074	-1.3956	0.1383	0.0832	0.9683	-1.4281	0.0700	-0.7510	0.3222	-0.7209	0.5806	-2.3385	0.0007	-0.8362	0.4752	-0.3239	0.8087	-0.8217	0.3042	-1.0548	0.2731	-1.5323	0.0218
AT1G21310	-0.5531	0.3133	-0.7328	0.2038	-1.3984	0.0100	-1.3984	0.0211	0.5800	0.4452	-1.2696	0.0194	-0.1740	0.7828	-0.9304	0.1488	-1.8212	0.0002	-2.0795	0.0000	-1.9621	0.0001	0.1826	0.7909	-1.1848	0.0332	-0.2264	0.6715
AT1G21320	-1.6672	0.0269	-1.9145	0.0134	-1.2685	0.1876	-0.6501	0.5876	-0.3827	0.8093	-1.4530	NA	-1.3802	0.0620	-0.2498	0.9080	-2.0561	0.0064	-1.7456	0.0576	-1.2228	0.2271	-1.8303	0.0151	-1.2960	0.1761	-0.9372	0.2016
AT1G21400	-1.3801	0.0404	0.3234	0.7327	0.2195	0.8489	0.6801	0.5116	-1.6930	0.0219	1.1808	0.1178	-2.0889	0.0008	0.4553	0.7661	1.2183	0.0806	0.9691	0.3331	-1.8319	0.0158	-1.0994	0.1203	0.1743	0.9156	1.0560	0.0980
AT1G21550	-1.4593	0.0022	-0.4848	0.4229	-0.5826	0.3886	-0.0651	0.9511	-0.7101	0.2986	-0.2711	0.6693	-2.0240	0.0000	-1.6088	0.0018	-0.4954	0.3666	-0.3752	0.6974	-0.7209	0.2860	-1.5667	0.0009	-0.7450	0.2593	-0.5865	0.2212
AT1G21680	0.6277	0.3243	1.6667	0.0037	1.6298	0.0092	1.6468	0.0181	0.5280	0.5921	2.1044	0.0005	0.6499	0.2871	1.2377	0.0745	2.3128	0.0000	1.7437	0.0065	-0.5585	0.5282	0.6005	0.3654	1.2565	0.0574	2.1680	0.0000
AT1G22120	-0.0480	0.9665	2.5557	0.0003	3.1027	0.0000	0.2802	0.8427	2.7851	0.0001	0.0000	NA	2.8166	0.0000	-0.0979	0.9699	0.1703	0.8649	0.1055	0.9659	0.3498	0.8042	-0.0381	0.9772	0.4294	0.7791	1.6336	0.0210
AT1G22360	1.5284	0.0004	-2.5909	0.0000	-1.7358	0.0001	0.1147	0.8996	-1.1626	0.0208	-0.1307	0.8418	-0.0523	0.9320	0.3161	0.7596	-0.4419	0.3894	0.0955	0.9459	0.4678	0.4946	1.1524	0.0105	-0.3039	0.7303	-0.0002	1.0000
AT1G22370	1.9568	0.0000	-1.4313	0.0037	-0.4254	0.5544	0.9291	0.1462	0.6539	0.3440	0.8797	0.1039	0.9327	0.0458	0.7840	0.2400	1.2832	0.0074	1.2510	0.0244	0.3992	0.6064	2.1649	0.0000	1.7608	0.0002	1.8031	0.0000
AT1G22400	-1.1026	0.0496	-3.2065	0.0000	-2.1898	0.0001	-0.3645	0.6958	-2.2329	0.0000	-1.0327	0.0974	-2.5838	0.0000	-1.6819	0.0043	-1.2073	0.0337	-1.2856	0.0567	-1.7129	0.0048	-1.4442	0.0084	-3.0476	0.0000	-1.9868	0.0001
AT1G22440	1.6578	0.0107	1.7086	0.0116	1.9214	0.0069	-0.0865	0.9550	2.5034	0.0001	0.9031	0.2524	1.9218	0.0016	1.0620	0.2605	0.8416	0.2530	1.3999	0.0962	1.2921	0.1313	0.8752	0.2333	1.1317	0.1901	0.4163	0.5631
AT1G22490	0.7305	0.2205	0.7695	0.2390	0.3862	0.6536	1.6213	0.0140	0.7007	0.3991	1.9945	0.0006	0.7084	0.2163	1.3711	0.0284	1.8085	0.0008	2.1467	0.0002	-0.1529	0.9021	1.0029	0.0822	1.9669	0.0003	1.7567	0.0007
AT1G22550	1.8370	0.0000	0.7126	0.0047	1.1767	0.0000	0.6595	0.0316	0.7252	0.0063	0.6347	0.0230	2.7064	0.0000	0.3087	0.4636	0.2259	0.4455	0.3496	0.3711	0.6642	0.0227	1.7513	0.0000	0.0432	0.9474	0.6914	0.0043
AT1G22570	2.6787	0.0000	0.2146	0.6763	1.0818	0.0071	0.5120	0.3470	1.0784	0.0061	1.1635	0.0035	2.3159	0.0000	1.0206	0.0125	0.7075	0.0695	0.7544	0.1176	0.7351	0.1230	2.4585	0.0000	1.0966	0.0050	1.3451	0.0001
AT1G22630	-0.7014	0.2365	-2.8748	0.0000	-2.7661	0.0000	-0.9193	0.2314	-1.7894	0.0014	-1.2883	0.0330	-2.2067	0.0000	-0.6638	0.4699	-1.7296	0.0014	-0.5886	0.5391	-0.1778	0.8762	-0.3637	0.5998	-0.5983	0.4946	-2.2424	0.0000
AT1G22650	-0.6263	0.1719	-2.2117	0.0000	-1.6569	0.0001	-0.6136	0.3283	-1.3478	0.0027	-1.1332	0.0168	-2.5425	0.0000	-0.7840	0.1704	-1.5258	0.0003	-1.0800	0.0361	0.3006	0.6831	-1.0858	0.0120	-0.6832	0.2348	-1.0502	0.0107
AT1G22770	1.6201	0.0021	2.0236	0.0001	1.8520	0.0010	1.4316	0.0331	1.7339	0.0018	1.4920	0.0117	1.8399	0.0003	1.8288	0.0012	2.1804	0.0000	2.2393	0.0001	-1.6099	0.0085	1.8718	0.0003	2.2254	0.0000	2.3028	0.0000
AT1G22890	-1.1780	0.0215	-1.4458	0.0052	-1.3002	0.0241	-0.5462	0.4905	-2.2113	0.0000	0.8802	0.1311	-2.5058	0.0000	-0.8339	0.2466	0.0600	0.9334	-0.1909	0.8878	-0.9996	0.1287	-1.2527	0.0148	-0.6313	0.4135	0.4375	0.3974
AT1G22990	-0.0732	0.9486	0.6255	0.5204	-0.0768	0.9603	0.8582	0.4588	0.4728	0.7516	0.7914	0.3866	-0.2597	0.7890	0.6477	0.6744	1.2686	0.1129	0.8164	0.5358	0.1295	0.9402	2.7128	0.0001	1.1329	0.2718	2.4370	0.0006
AT1G23020	0.1271	0.7836	-2.6662	0.0000	-2.0005	0.0000	-0.4507	0.3829	-1.8832	0.0000	-0.0363	0.9472	-0.6379	0.0636	-0.0330	0.9795	-0.4777	0.2045	-0.3468	0.5787	-0.8966	0.0274	0.5832	0.1126	-0.6088	0.1690	-0.1166	0.7604
AT1G23060	-2.4524	0.0000	-2.4717	0.0000	-1.5136	0.0361	-1.6294	0.0378	-0.2895	0.8316	-2.1965	0.0012	-2.9186	0.0000	0.0165	0.9948	-1.6598	0.0092	-0.9977	0.2705	-0.9160	0.2948	-2.2101	0.0002	0.4327	0.7179	-0.0570	0.9383
AT1G23120	-0.5981	0.3255	2.1827	0.0000	2.0741	0.0001	-0.2689	0.7816	1.3161	0.0264	0.3013	0.6717	0.4984	0.3888	-0.0173	0.9941	0.4714	0.4461	0.7208	0.3859	-0.3860	0.6836	-0.3529	0.6097	0.2168	0.8595	1.1137	0.0279
AT1G23130	0.1247	0.8633	-2.9011	0.0000	-2.3516	0.0000	-0.5531	0.4875	-0.3940	0.6800	-0.9974	0.0854	-1.1969	0.0159	0.0859	0.9625	-1.0188	0.0575	0.2596	0.8272	0.2764	0.7674	0.1705	0.8165	-0.2230	0.8447	-1.0745	0.0277
AT1G23140	-0.5889	0.4185	-2.4372	0.0001	-2.3862	0.0003	-0.3388	0.7631	-0.8226	0.3857	-0.2591	0.7657	-1.7840	0.0031	-0.0914	0.9688	-1.0605	0.1194	-0.0002	1.0000	-0.1625	0.9075	-0.6055	0.4179	-0.3287	0.8080	-0.7877	0.2111
AT1G23340	-0.8888	0.0771	-0.1916	0.7889	-0.3379	0.6518	-0.6188	0.3954	-0.0988	0.9368	-0.9215	0.0978	-1.1321	0.0155	-0.0522	0.9762	-0.6958	0.1870	-0.8866	0.1851	-0.1386	0.8949	-0.9547	0.0594	0.2850	0.7735	-2.1903	0.0000
AT1G23510	-2.5839	NA	-2.5129	0.0004	-2.2563	0.0037	-2.2068	NA	-1.6462	0.0596	-2.1871	NA	-2.7150	0.0001	-2.2118	NA	-2.4354	NA	-2.7157	NA	-2.2351	0.0055	-2.3774	0.0008	-1.5543	0.0740	-1.9239	NA
AT1G23560	-1.3520	0.0254	-0.5418	0.4691	-0.8976	0.2598	-1.2899	0.1012	-0.5575	0.5817	-1.4123	0.0418	-0.4925	0.4402	-1.3466	0.0620	-1.0283	0.1104	-0.7251	0.4566	-2.0075	0.0043	-1.4318	0.0189	-1.7144	0.0122	-2.4066	0.0001
AT1G23720	0.0712	0.9499	1.8480	0.0181	1.9422	0.0212	-0.0555	0.9747	-0.0013	1.0000	-0.1444	0.8982	-0.3963	0.6617	2.3388	0.0034	0.1347	0.8950	0.6253	0.6672	1.6899	0.0658	-0.2039	0.8545	1.1530	0.2564	0.1824	0.8281
AT1G23740	-0.1225	0.8569	-1.7461	0.0002	-1.8534	0.0001	-0.5093	0.4953	-2.1216	0.0000	-1.6412	0.0014	-1.0218	0.0296	-1.0717	0.0635	-1.6575	0.0005	-0.7264	0.3102	-0.2641	0.7627	-0.0933	0.8981	-0.9026	0.1299	-2.5411	0.0000
AT1G23935	0.3860	0.5281	-3.4836	0.0000	-3.9118	0.0000	0.2398	0.7983	-3.6438	0.0000	-0.4998	0.4379	-4.4140	0.0000	0.2442	0.8583	-0.0924	0.8956	-0.2841	0.8091	0.1108	0.9224	0.5087	0.3964	-0.5172	0.5555	-1.7668	0.0006
AT1G23950	-0.3014	0.4129	-5.5642	0.0000	-4.9550	0.0000	-0.1316	0.8255	-4.4746	0.0000	-0.4902	0.1916	-6.2301	0.0000	-0.0855	0.9299	-0.6589	0.0507	-0.1270	0.8781	0.1612	0.7863	-0.1317	0.7665	-0.1383	0.8473	-0.4182	0.1878
AT1G23960	-0.2655	0.4849	-6.8919	0.0000	-6.9087	0.0000	0.2710	0.6045	-6.8422	0.0000	0.6670	0.0652	-7.3383	0.0000	0.1303	0.8838	0.6103	0.0716	0.5932	0.1770	-0.0683	0.9242	-0.2382	0.5544	0.0136	0.9909	1.0458	0.0006
AT1G23965	-2.4616	0.0003	-2.3474	0.0007	-2.4157	0.0011	-1.4880	0.1012	-2.1839	0.0031	-0.2673	NA	-2.7391	0.0000	-0.7852	0.5081	-1.0238	0.1683	-1.4741	0.0969	-1.4334	0.1045	-2.3093	0.0007	-0.5368	0.6726	-1.7012	0.0114
AT1G24140	-1.9066	0.0033	-2.4890	0.0001	-1.7377	0.0195	-0.9422	0.3300	-1.1110	0.2069	-1.7813	0.0144	-1.9853	0.0014	-2.6068	0.0001	-2.5577	0.0001	-1.7782	0.0213	0.0476	0.9785	-2.5321	0.0001	-1.9187	0.0066	0.0594	0.9392
AT1G24145	-2.7223	0.0000	-5.2767	0.0000	-4.3660	0.0000	-2.4053	0.0004	-3.1400	0.0000	-1.9880	0.0024	-5.1491	0.0000	-2.3950	0.0001	-3.3410	0.0000	-2.3388	0.0003	-0.9536	0.2549	-2.8037	0.0000	-1.9330	0.0024	-1.2794	0.0279
AT1G24148	-0.9346	0.2196	-2.3779	0.0005	-1.2692	0.1533	-1.1527	0.2470	0.0818	0.9691	-1.6935	0.0297	-1.0274	0.1550	-0.7118	0.5889	-2.3082	0.0009	-0.1385	0.9504	0.1037	0.9491	-0.6953	0.3999	-0.4300	0.7618	-0.3439	0.6488
AT1G24260	-0.0895	0.9117	1.8355	0.0005	1.7565	0.0022	0.3414	0.7228	2.3101	0.0000	0.2273	0.7720	1.6473	0.0011	0.3829	0.7665	-0.0071	0.9941	-0.3240	0.8017	-0.8397	0.3171	-0.0494	0.9585	0.7987	0.3097	0.3890	0.5123
AT1G24430	0.9698	0.2205	1.0595	0.2194	-0.0809	0.9576	0.5189	0.6741	0.5713	0.6792	1.6195	NA	0.2846	0.7588	1.2173	0.2315	1.3036	0.0924	2.3157	0.0034	1.1992	0.2284	1.4082	0.0625	1.4949	0.0873	2.1490	0.0018
AT1G24470	-1.1204	0.0162	-1.6066	0.0009	-2.2112	0.0000	-1.0478	0.0828	-1.2773	0.0164	-1.2168	0.0248	-1.5528	0.0003	-1.5757	0.0018	-1.3871	0.0049	-0.9415	0.1256	-0.3921	0.5967	-1.0565	0.0255	-0.8827	0.1382	-0.5212	0.2670
AT1G24530	0.2908	0.6383	-2.2224	0.0000	-0.9656	0.1156	-0.8533	0.2190	-0.4624	0.5879	-1.4487	0.0077	-0.3206	0.5812	-0.2528	0.8446	-2.1547	0.0000	-1.1275	0.0678	-0.0554	0.9629	-0.0145	0.9872	-0.5440	0.4946	-0.8940	0.0605
AT1G24800	-0.3529	0.6612	-2.4940	0.0002	-2.4395	0.0007	-0.0020	0.9997	-1.9872	0.0070	0.3840	0.6430	-2.9568	0.0000	0.0113	0.9962	0.6550	0.3576	0.3532	0.8037	0.0401	0.9819	-0.7475	0.3103	-0.1769	0.9132	1.3071	0.0277
AT1G24880	-1.5109	0.0494	-1.9680	0.0104	-1.9447	0.0199	0.0518	0.9765	-1.6384	0.0660	-0.7466	NA	-2.1978	0.0020	-0.3022	0.8847	-0.6867	0.4280	-0.8718	0.4938	-1.2116	0.2358	-1.1373	0.1638	-0.6155	0.6553	-0.3161	0.7006
AT1G24996	-0.2378	0.8164	-2.7228	0.0002	-2.7213	0.0004	-1.4528	0.1535	-2.6677	0.0004	-0.2110	0.8471	-2.7404	0.0001	-0.5354	0.7546	-0.5301	0.5531	-0.0420	0.9841	-0.0890	0.9608	-1.4794	0.0589	-0.2499	0.8879	-0.7360	0.3277
AT1G25422	0.7286	0.3859	1.3557	0.0963	1.2207	0.2009	1.7251	0.0637	1.6766	0.0494	1.2114	NA	0.4346	0.6185	1.7308	0.0405	0.9114	0.2662	1.3027	0.1993	0.2604	0.8658	1.7745	0.0149	2.6898	0.0002	0.6128	0.4234
AT1G25510	0.9241	0.1247	-0.5777	0.4251	-0.8489	0.2730	-0.3640	0.7124	0.1998	0.8805	-1.4400	0.0261	0.8284	0.1594	0.2420	0.8772	-1.3126	0.0294	-0.5891	0.5693	0.6413	0.4558	0.8702	0.1601	-0.0457	0.9799	-2.0864	0.0002
AT1G25988	-0.5416	0.5511	3.6938	0.0000	3.7105	0.0000	-0.0948	0.9553	4.0142	0.0000	0.1939	0.8605	2.4838	0.0001	0.1122	0.9676	-0.0767	0.9432	-0.1701	0.9399	-0.3247	0.8243	-0.3481	0.7337	0.5278	0.7143	0.2942	0.7211
AT1G26200	-1.4353	0.0247	-1.3457	0.0499	-0.0573	0.9650	-1.7787	0.0239	0.9567	0.2671	-1.5788	0.0260	-0.3304	0.6623	-1.0986	0.2045	-2.1637	0.0007	-1.1396	0.1925	-1.0887	0.2010	-1.3647	0.0360	-1.5965	0.0253	-0.9134	0.1401
AT1G26380	-2.2754	0.0001	-2.1546	0.0002	-0.8481	0.2880	-0.1255	0.9184	-1.7687	0.0049	-0.6166	0.3936	-2.0193	0.0003	-2.3073	0.0001	-1.8826	0.0015	-1.6203	0.0190	-2.2888	0.0002	-2.5455	0.0000	-2.1386	0.0004	-1.2587	0.0263
AT1G26390	-1.0712	0.1252	-1.9717	0.0032	-2.2004	0.0016	-1.5834	0.0637	-1.5834	0.0387	-0.8422	0.2856	-1.7444	0.0059	-2.6536	0.0001	-1.1353	0.1077	-1.2269	0.1752	-2.4050	0.0007	-1.0116	0.1592	-1.8197	0.0112	-0.9107	0.1598
AT1G26410	-0.6963	0.3882	-1.3549	0.0849	-1.8693	0.0201	-0.6863	0.5429	-1.7747	0.0283	-0.8884	0.2939	-1.2402	0.0804	-2.5194	0.0007	-2.0539	0.0044	-1.8506	0.0288	-1.7029	0.0483	-1.0615	0.1688	-1.6192	0.0491	-0.5105	0.4872
AT1G26420	-1.6473	0.0032	-2.2804	0.0000	-1.0829	0.1272	-0.5681	0.5251	-1.8216	0.0021	-0.4400	0.5409	-2.1519	0.0000	-2.0709	0.0004	-1.5904	0.0057	-1.5300	0.0213	-2.0348	0.0008	-2.1140	0.0001	-1.7587	0.0034	-0.6931	0.2169
AT1G26530	0.1361	0.8761	2.4694	0.0000	2.0610	0.0004	0.3733	0.7191	2.5326	0.0000	0.3768	0.6453	2.2156	0.0000	0.9695	0.2437	0.7145	0.2923	0.3987	0.7649	0.6302	0.5007	1.1538	0.0580	0.5502	0.6150	0.2263	0.7481
AT1G26560	-1.2458	0.0377	-2.2937	0.0001	-1.2831	0.0679	-0.9197	0.2755	-1.3311	0.0556	-1.7640	0.0061	-1.7274	0.0019	-1.3901	0.0446	-2.1839	0.0002	-0.8543	0.3415	0.1302	0.9208	-1.1269	0.0677	-0.7504	0.3941	-2.3824	0.0000
AT1G26730	0.4275	0.5603	-1.2199	0.0680	-0.4316	0.6449	-0.4877	0.6230	0.2484	0.8556	-1.4759	0.0302	0.1066	0.8950	-0.4674	0.7245	-2.4336	0.0001	-0.6481	0.5471	0.1353	0.9205	-0.1649	0.8548	-0.8572	0.3248	-1.3152	0.0248
AT1G26761	0.0121	0.9902	-0.2209	0.7919	-0.2143	0.8255	-0.5950	0.4988	-0.4862	0.6302	-1.4145	0.0236	0.3917	0.5501	-0.6259	0.5337	-1.6279	0.0042	-0.7439	0.4080	-0.1152	0.9276	0.1855	0.8189	0.1707	0.9011	-2.7770	0.0000
AT1G26790	-3.4979	0.0000	-3.4242	0.0000	-3.1513	0.0000	-3.1838	0.0001	-3.3583	0.0000	-3.1364	0.0001	-3.9087	0.0000	-3.0839	0.0000	-3.4804	0.0000	0.1916	0.9295	1.1061	0.2883	-3.3105	0.0000	-3.4601	0.0000	-3.3752	0.0000
AT1G26945	-2.6953	0.0000	-0.2259	0.7437	-0.0030	0.9982	-0.2330	0.7878	-0.0551	0.9693	0.4093	0.4963	-2.4420	0.0000	0.3469	0.7498	-0.1260	0.8451	-0.8848	0.1768	-0.5304	0.4618	-2.2668	0.0000	0.4815	0.5494	-0.2236	0.6661
AT1G27110	-0.2484	0.7409	-1.4064	0.0307	-2.0710	0.0021	-0.4533	0.6329	-1.2049	0.1110	-0.9096	0.1988	-1.6184	0.0041	-0.7424	0.4404	-0.8082	0.2168	-0.4952	0.6627	-0.1160	0.9293	-0.1288	0.8810	-0.1717	0.9047	-0.8967	0.1323
AT1G27130	-0.6004	0.1729	-2.5559	0.0000	-1.6029	0.0001	0.1643	0.8309	-1.4927	0.0003	-0.4884	0.3218	-1.6922	0.0000	-0.3384	0.6979	-0.8433	0.0500	-0.5194	0.4365	-0.0604	0.9496	-0.6410	0.1495	-0.5442	0.3692	-0.4229	0.3040
AT1G27180	-0.4017	0.4857	-5.0171	0.0000	-5.0547	0.0000	-0.8066	0.2470	-3.6410	0.0000	-0.6390	0.2734	-5.6303	0.0000	-0.4621	0.6297	-0.8300	0.1140	-0.6405	0.4144	-0.0360	0.9778	-0.5417	0.3370	-0.4631	0.5838	-0.9213	0.0522
AT1G27480	-0.3822	0.5639	-1.3424	0.0190	-0.9289	0.1879	-0.8671	0.2651	-0.7817	0.3159	-1.8396	0.0018	-0.2109	0.7599	-0.7499	0.3817	-2.1139	0.0001	-0.5272	0.6009	0.5086	0.5518	-0.0767	0.9294	-0.5686	0.5287	-2.7064	0.0000
AT1G27540	-0.1131	0.7016	-2.2049	0.0000	-1.7325	0.0000	-0.0634	0.8905	-1.9534	0.0000	0.1561	0.5994	-2.2907	0.0000	0.1467	0.7888	0.1611	0.5583	0.1360	0.7901	-0.0876	0.8504	-0.0821	0.7991	0.0994	0.8475	-0.1289	0.6065
AT1G27565	0.0000	1.0000	2.7196	0.0001	5.1932	0.0000	0.0537	0.9754	0.9836	0.3705	0.0000	1.0000	-0.1042	0.9185	-0.1306	0.9600	0.2026	0.8392	0.5176	0.7403	0.1814	0.9126	-0.0544	0.9663	5.3506	0.0000	0.0000	1.0000
AT1G27570	-0.5525	0.5354	6.3899	0.0000	6.6348	0.0000	-0.4672	0.7119	5.5559	0.0000	-0.1786	0.8715	5.8235	0.0000	-0.6092	0.6980	-0.2606	0.7891	-0.3641	0.8357	-0.2796	0.8518	-0.3621	0.7166	1.3759	0.1147	0.1235	0.8857
AT1G27670	-0.6496	0.3740	0.6694	0.3981	1.3154	0.0851	0.5267	0.6098	0.1001	0.9548	1.0777	0.1390	-0.9509	0.1488	0.4509	0.7563	0.9083	0.1860	0.4182	0.7603	0.3587	0.7555	-0.3332	0.6971	0.8253	0.3811	2.7102	0.0000
AT1G27730	-0.9096	0.1346	-0.4020	0.6028	-0.4804	0.5787	0.2233	0.8360	-0.9741	0.2024	-0.4617	0.5238	-1.2440	0.0265	-1.3677	0.0420	-0.5618	0.3917	-1.0171	0.2022	2.0809	0.0006	-1.0569	0.0807	-1.0032	0.1737	0.4699	0.4227
AT1G27980	-2.0170	0.0000	-1.0809	0.0002	-0.4466	0.2630	-0.8365	0.0240	-0.2135	0.7089	-0.5567	0.1031	-1.8668	0.0000	-0.8296	0.0125	-0.6665	0.0320	-0.3444	0.4977	-0.7622	0.0318	-1.9427	0.0000	-1.2945	0.0000	-0.5989	0.0371
AT1G28190	-2.4410	0.0000	-1.9206	0.0002	-1.5489	0.0085	-0.5959	0.4734	-1.1696	0.0680	-0.7480	0.2354	-2.4181	0.0000	-1.4440	0.0159	-1.4119	0.0096	-0.8760	0.2557	-1.2022	0.0690	-2.0561	0.0001	-1.5206	0.0082	-1.3360	0.0091
AT1G28230	2.3307	0.0002	0.1832	0.8554	0.9031	0.3119	1.1642	0.2020	2.1165	0.0017	0.5128	0.5395	2.2644	0.0002	1.6359	0.0294	0.9029	0.2076	1.5753	0.0483	1.4713	0.0679	2.5621	0.0000	1.9218	0.0056	2.7368	0.0000
AT1G28370	-1.1098	0.1189	-1.4162	0.0541	-1.3455	0.1063	-0.0225	0.9906	-0.5878	0.6254	-1.2907	0.0937	-1.3013	0.0519	-1.0612	0.2796	-2.1119	0.0019	-1.1159	0.2495	1.4068	0.0973	-1.3891	0.0478	-1.1151	0.2114	0.1412	0.8543
AT1G28480	-2.3394	0.0006	-2.7376	0.0001	-1.9451	0.0129	0.0748	0.9624	-1.2544	0.1722	-1.3173	0.1002	-2.5616	0.0001	-2.1108	0.0057	-1.7849	0.0132	-1.0999	0.2862	-0.5601	0.6290	-2.8907	0.0000	-1.6431	0.0413	-0.9676	0.1597
AT1G28660	-0.3720	0.6314	-1.9804	0.0013	-0.5597	0.5456	-1.5351	0.0526	-0.1725	0.9082	-2.0018	0.0028	-0.5839	0.3938	-0.9094	0.3317	-2.6586	0.0000	-1.1498	0.1744	0.6168	0.5190	-0.6477	0.3704	-1.0489	0.1950	-0.9588	0.1112
AT1G28670	0.0596	0.9486	-1.2671	0.0571	-0.2822	0.7820	-0.9633	0.2721	0.2726	0.8406	-1.7377	0.0099	0.0856	0.9185	-0.3904	0.7893	-2.3528	0.0001	-0.5958	0.5970	0.7948	0.3677	0.0627	0.9514	-0.2675	0.8441	-1.1390	0.0523
AT1G29195	-2.5196	0.0000	-0.4116	0.4870	-0.5731	0.3762	-0.5661	0.4179	0.4249	0.5866	-1.5021	0.0048	-1.4437	0.0009	-0.6537	0.3616	-0.6949	0.1696	-0.1423	0.9116	-0.3145	0.6956	-2.2079	0.0000	-1.3823	0.0078	-1.3366	0.0043
AT1G29357	-2.1547	0.0009	0.0529	0.9600	0.0531	0.9687	0.1064	0.9384	0.3001	0.8277	-0.0951	0.9230	-1.1108	0.0828	-0.3998	0.7935	0.3860	0.6162	0.2366	0.8878	-0.3362	0.7769	-1.4592	0.0295	-0.6776	0.5297	0.2129	0.7634
AT1G29410	-0.0447	0.9208	-3.5293	0.0000	-3.0224	0.0000	0.3122	0.5143	-2.9092	0.0000	0.2910	0.4529	-3.0444	0.0000	0.1637	0.8366	0.3364	0.3342	0.3061	0.5839	0.3408	0.4631	0.2023	0.6041	0.1832	0.7758	-0.1525	0.6604
AT1G29465	-2.3496	0.0000	-0.0951	0.8929	-0.3096	0.6571	-0.2592	0.7424	-0.1438	0.8927	-0.0532	0.9389	-1.7573	0.0000	-0.3903	0.6711	-0.1458	0.8058	-0.5193	0.4974	0.1651	0.8548	-1.9198	0.0000	-0.7291	0.2281	0.4619	0.3021
AT1G29530	-0.8620	0.1640	-1.2415	0.0473	-1.0911	0.1344	-0.7855	0.3594	-0.4800	0.6489	-1.7204	0.0068	-0.6814	0.2695	-0.5339	0.6433	-1.7131	0.0033	-0.5768	0.5849	-0.1865	0.8782	-0.6575	0.3231	-0.6117	0.5231	-2.5088	0.0000
AT1G29640	-2.2358	0.0008	-0.5175	0.5600	-1.0119	0.2627	-0.8434	0.4076	-0.9574	0.3257	-0.5773	0.4968	-2.7336	0.0000	-1.3810	0.1069	-0.4681	0.5623	-0.1429	0.9459	-0.7120	0.4890	-1.0685	0.1425	-0.7501	0.4894	0.5326	0.4385
AT1G29720	-1.7219	0.0095	-2.7473	0.0000	-1.6268	0.0337	-1.6560	0.0512	-1.1838	0.1741	-2.1069	0.0034	-1.5936	0.0134	-1.4636	0.0702	-2.8203	0.0000	-0.9245	0.3713	-0.2213	0.8732	-1.6443	0.0146	-1.7258	0.0185	-3.2262	0.0000
AT1G30080	-0.5101	0.4986	-0.3143	0.7317	0.0344	0.9796	-0.6104	0.5490	-0.0084	1.0000	-1.0040	0.1954	-2.0464	0.0010	-0.2713	0.8755	-0.6087	0.4156	0.1380	0.9430	-0.6895	0.4865	-0.9444	0.1810	0.0111	0.9984	-0.1808	0.8033
AT1G30135	-0.0156	0.9903	-0.4373	0.6670	-0.0886	0.9524	1.2630	0.2166	-0.6196	0.6433	-0.0714	0.9517	-0.6352	0.4402	-0.6864	0.6369	-0.3050	0.7476	0.3543	0.8379	2.2582	0.0052	-0.7641	0.3746	-0.6943	0.5859	0.0805	0.9258
AT1G30190	-1.4874	0.0237	-1.7217	0.0114	-0.7454	0.4185	-1.0497	0.2565	-0.9933	0.2712	-0.5662	0.4876	-2.0249	0.0009	-1.5255	0.0481	-1.4044	0.0396	-1.2827	0.1379	0.6301	0.5269	-0.6278	0.4113	-1.3663	0.0807	-2.1163	0.0009
AT1G30250	0.7216	0.2716	1.8800	0.0015	1.3215	0.0617	1.1961	0.1350	0.3315	0.7910	1.3821	0.0369	1.2113	0.0391	1.0833	0.1747	2.5251	0.0000	1.6078	0.0207	1.4278	0.0466	0.8717	0.1815	1.2247	0.0862	2.4753	0.0000
AT1G30370	-2.0459	0.0028	-2.1743	0.0019	-1.7467	0.0285	-0.9282	0.3642	-1.5181	0.0683	-1.3093	0.0987	-2.1452	0.0010	-1.7621	0.0267	-2.0869	0.0030	-1.0749	0.2907	-0.6274	0.5646	-2.9568	0.0000	-1.5901	0.0480	-1.1597	0.0848

AT1G30420	-1.6935	0.0003	-2.2978	0.0000	-1.0258	0.0803	-0.9750	0.1350	-0.5522	0.4690	-1.1478	0.0336	-1.7360	0.0001	-1.0580	0.0739	-1.8306	0.0001	-1.2032	0.0400	-0.8518	0.1903	-1.7661	0.0001	-1.4514	0.0048	-1.6037	0.0005
AT1G30700	-0.5253	0.3788	-1.4786	0.0060	-1.1453	0.0685	-0.6624	0.4018	-1.6453	0.0029	-0.4715	0.4731	-1.3616	0.0067	-1.5679	0.0062	0.2860	0.6596	0.1018	0.9511	-1.8844	0.0008	-1.0900	0.0452	-2.1994	0.0000	-0.0752	0.9016
AT1G30730	-1.1640	0.0618	-0.5700	0.4573	0.4112	0.6607	-0.4714	0.6356	-1.1102	0.1542	0.7829	0.2733	-1.5766	0.0064	-0.7534	0.4536	-0.0152	0.9878	-0.5567	0.6255	-2.3739	0.0002	-1.6958	0.0047	-0.6800	0.4799	0.7478	0.2085
AT1G30750	-0.5067	0.5799	0.2193	0.8473	1.0296	0.3139	-0.2094	0.8897	-0.2528	0.8851	-0.4926	0.6085	-0.9082	0.2549	-0.1275	0.9614	-0.3734	0.6910	-0.2846	0.8863	1.5905	0.0913	-0.4654	0.6317	-0.4368	0.7773	2.5980	0.0002
AT1G30755	-1.7912	0.0000	-1.6785	0.0000	-0.6790	0.1160	-0.7472	0.1045	-0.8797	0.0254	-0.3594	0.3989	-1.9711	0.0000	-1.4398	0.0000	-1.1583	0.0008	-1.0989	0.0048	-0.9458	0.0194	-2.1304	0.0000	-1.5371	0.0000	-1.2990	0.0001
AT1G30900	-2.0688	0.0012	-2.4639	0.0001	-1.3434	0.0914	-1.7807	0.0285	-0.2505	0.8660	-1.6580	0.0217	-1.7468	0.0052	-1.8311	0.0113	-2.4703	0.0001	-1.0003	0.3011	-1.1648	0.1815	-2.0515	0.0013	-1.8464	0.0085	-1.9213	0.0020
AT1G31580	-1.3946	0.0643	-2.4711	0.0005	-1.4463	0.1077	-1.6292	0.0891	-0.5302	0.7033	-2.0914	0.0089	-1.2393	0.0930	-1.5666	0.0804	-2.5302	0.0004	-1.0945	0.3185	0.0766	0.9655	-1.2395	0.1118	-1.1196	0.2606	0.0716	0.9339
AT1G31690	2.0485	0.0027	-0.0518	0.9647	0.4596	0.6747	0.7352	0.4988	1.0714	0.2661	-0.4871	0.5874	2.3788	0.0002	1.2086	0.2059	-0.2084	0.8225	-0.2955	0.8675	0.6252	0.5717	2.5555	0.0001	0.6859	0.5616	-1.4897	0.0275
AT1G31800	0.1973	0.6943	-0.5053	0.2840	-0.3073	0.6046	-0.2208	0.7491	-0.1371	0.8802	-0.8569	0.0507	0.3481	0.4247	-0.3521	0.6548	-0.9967	0.0127	-0.5820	0.3244	-0.1468	0.8518	0.1504	0.7824	-0.8948	0.0469	-2.3575	0.0000
AT1G32080	0.3961	0.5098	-0.8305	0.1504	-0.3256	0.6820	-0.2020	0.8308	-0.2523	0.8165	-1.3432	0.0155	0.7161	0.1710	-0.1059	0.9504	-1.5289	0.0024	-1.1017	0.0845	0.0549	0.9641	0.2499	0.7136	-1.1553	0.0462	-2.7920	0.0000
AT1G32220	0.7261	0.0620	-0.6127	0.1590	-0.1408	0.8292	-0.1766	0.7994	0.1531	0.8578	-1.0703	0.0101	0.9006	0.0135	-0.0954	0.9360	-1.2725	0.0007	-1.1672	0.0061	-0.2644	0.6858	0.6786	0.0890	-0.4890	0.3865	-2.0495	0.0000
AT1G32350	-2.0399	0.0040	-2.2835	0.0014	-2.1670	0.0054	1.3081	0.1867	-1.8863	0.0182	-0.9643	0.2551	-1.7474	0.0117	-2.3971	0.0015	-1.4120	0.0613	-1.1253	0.2848	-2.4723	0.0015	-1.5426	0.0374	-2.4602	0.0008	-0.1885	0.8152
AT1G32560	-0.7352	0.3911	2.2435	0.0028	0.3729	0.7628	-0.2531	0.8637	-0.1598	0.9339	-0.6312	NA	-0.8932	0.2641	0.0049	0.9994	0.4718	0.6056	0.3452	0.8479	1.3023	0.1951	-0.4494	0.6448	0.6971	0.5919	0.4545	0.5662
AT1G32780	-0.4054	0.5264	-1.2846	0.0260	-1.1605	0.0760	-0.7512	0.3414	-1.5131	0.0113	-1.7487	0.0037	-0.7214	0.1942	-1.2798	0.0450	-1.9794	0.0003	-1.5707	0.0126	-0.3055	0.7522	-0.3261	0.6368	-1.2574	0.0457	-2.5728	0.0000
AT1G32950	-1.7670	0.0060	-1.7193	0.0110	-0.9548	0.2657	-0.7778	0.4208	-1.0310	0.2390	-1.5997	0.0283	-1.8608	0.0022	-1.5566	0.0395	-1.5469	0.0217	-0.6776	0.5494	-0.8252	0.3742	-2.2454	0.0004	-2.0901	0.0027	-0.8786	0.1659
AT1G32960	-2.7050	0.0000	-2.5475	0.0001	-1.7499	0.0249	-0.1898	0.8905	-1.1684	0.1999	-1.3198	0.0905	-2.7158	0.0000	-2.6162	0.0002	-2.4588	0.0003	-1.1575	0.2338	-1.9760	0.0118	-2.5816	0.0001	-2.2150	0.0020	-1.4209	0.0305
AT1G33030	-2.0871	0.0013	-1.6720	0.0160	-1.4557	0.0661	0.1699	0.8978	-0.6092	0.5940	-0.1577	0.8715	-2.1783	0.0004	-1.4937	0.0607	-1.1984	0.0879	-0.3488	0.8195	-1.7609	0.0239	-2.0068	0.0021	-1.4914	0.0530	0.4529	0.5083
AT1G33102	-1.8361	0.0001	-0.5366	0.3503	-1.8276	0.0005	-1.1532	0.0570	-1.4327	0.0066	-1.5254	0.0059	-2.0497	0.0000	-1.8552	0.0002	-0.8440	0.0970	-0.5614	0.4706	0.0881	0.9327	-1.0793	0.0249	-1.6763	0.0015	-1.1427	0.0165
AT1G33110	-1.9643	0.0000	-1.1724	0.0080	-1.4724	0.0010	-0.7157	0.2415	-2.4450	0.0000	-1.4252	0.0021	-2.4154	0.0000	-1.8904	0.0000	-1.2489	0.0039	-0.6635	0.2967	-0.5980	0.3297	-1.7761	0.0000	-1.6595	0.0001	-1.3896	0.0007
AT1G33440	1.1439	0.0202	1.9856	0.0000	0.8508	0.1817	1.7828	0.0013	1.2529	0.0228	0.1708	0.8110	1.1205	0.0177	1.5416	0.0031	2.1354	0.0000	1.9602	0.0001	1.4434	0.0090	1.4999	0.0016	1.8728	0.0001	1.2867	0.0063
AT1G33720	-1.4531	0.0001	-1.0515	0.0066	-0.3711	0.5026	-1.6028	0.0001	-0.1964	0.8069	-0.4334	0.3410	-1.9202	0.0000	-1.4782	0.0001	-0.8959	0.0217	-1.4363	0.0004	-1.0967	0.0109	-2.1457	0.0000	-0.8847	0.0408	-1.1498	0.0015
AT1G33760	-0.0532	0.9586	-0.7104	0.4047	-1.0445	0.2494	-0.3374	0.7820	-0.8751	0.3921	-1.1036	0.1652	-0.3550	0.6645	-1.1901	0.2037	-1.4396	0.0451	-1.9367	0.0156	2.3379	0.0011	-0.2199	0.8216	-1.3238	0.1192	-1.5006	0.0235
AT1G33950	-2.4405	0.0008	-1.9531	0.0118	-1.4386	0.1233	-2.2702	0.0121	-0.4508	0.7658	-1.4023	0.1000	-1.6541	0.0234	-2.1392	0.0094	-2.2353	0.0029	-1.7837	0.0521	-1.3951	0.1576	-2.0185	0.0069	-1.0771	0.3076	-0.5448	0.4821
AT1G33960	-3.3946	0.0000	-3.5189	0.0000	-1.9041	0.0132	-2.5239	0.0014	-1.3523	0.1191	-2.0092	0.0080	-2.8859	0.0000	-2.7466	0.0001	-3.0997	0.0000	-2.2976	0.0026	-2.0821	0.0072	-3.3731	0.0000	-2.2791	0.0014	-2.4419	0.0002
AT1G34060	-1.3894	0.0653	-1.4456	0.0712	-0.5592	0.6170	-1.1206	0.2868	0.4030	0.7918	-1.2387	0.1404	-1.1579	0.1199	-0.2857	0.8886	-2.0747	0.0047	-0.0492	0.9826	0.2489	0.8687	-1.2230	0.1173	-0.0540	0.9816	-0.4520	0.5566
AT1G34420	-1.8538	0.0003	-1.9271	0.0002	-1.4076	0.0199	-1.1660	0.0978	-0.6591	0.4151	-1.2549	0.0359	-2.0454	0.0000	-1.2695	0.0434	-1.5713	0.0037	-1.1778	0.0814	-0.0827	0.9463	-2.1379	0.0000	-1.6800	0.0031	-0.8688	0.0942
AT1G34750	-1.1397	0.0553	-2.3507	0.0000	-1.1314	0.1150	-1.7519	0.0123	-0.4100	0.7111	-1.4668	0.0219	-1.4542	0.0090	-1.3510	0.0484	-2.1951	0.0001	-0.9465	0.2562	-0.4782	0.6130	-1.4858	0.0108	-1.5344	0.0153	-1.3890	0.0120
AT1G35210	-1.8536	0.0072	-2.1286	0.0023	-1.4719	0.0788	-1.0291	0.3068	-0.9746	0.3297	-1.5843	0.0411	-1.8389	0.0056	-1.7836	0.0240	-2.4438	0.0004	-1.5337	0.0802	0.0163	0.9964	-1.9211	0.0054	-1.6250	0.0408	-0.5755	0.4166
AT1G35230	-2.0708	0.0001	-2.5053	0.0000	-1.8762	0.0012	-1.9048	0.0031	-1.5825	0.0078	-1.7597	0.0033	-1.9877	0.0001	-2.7781	0.0000	-3.1196	0.0000	-2.9235	0.0000	-1.5427	0.0154	-2.4374	0.0000	-2.4786	0.0000	-0.9012	0.0917
AT1G35290	1.2933	0.0770	-0.1898	0.8643	0.6174	0.5651	0.9185	0.3869	0.5197	0.7026	0.0988	0.9301	1.0993	0.1260	0.4951	0.7638	-0.0609	0.9539	0.4483	0.7733	2.4649	0.0010	0.9799	0.2091	0.3716	0.8095	-0.1222	0.8844
AT1G35350	-0.3278	0.5624	-2.1490	0.0000	-0.6898	0.2675	-1.0582	0.0824	-0.9331	0.1062	-0.9051	0.0867	-1.4099	0.0013	-1.1624	0.0302	-1.6325	0.0004	-0.7642	0.2538	0.0366	0.9765	-0.9917	0.0393	-1.5697	0.0011	-1.3646	0.0021
AT1G35710	-2.0782	0.0013	-3.4351	0.0000	-1.8798	0.0098	-2.1564	0.0058	-0.7217	0.4959	-1.7506	0.0161	-2.5360	0.0000	-1.9287	0.0076	-2.8399	0.0000	-1.9178	0.0112	-0.7065	0.4815	-2.4424	0.0001	-2.0448	0.0031	-2.2566	0.0003
AT1G36622	-2.1929	0.0007	-2.5918	0.0001	-1.9069	0.0096	-0.9209	0.3454	-1.6037	0.0368	-1.4893	0.0461	-2.6569	0.0000	-2.5911	0.0001	-2.3921	0.0003	-1.9030	0.0131	-1.7317	0.0273	-1.8163	0.0061	-2.2122	0.0014	-1.4157	0.0264
AT1G37130	-3.9529	0.0000	-0.2873	0.6374	-0.7046	0.2293	-0.2036	0.8060	-2.0878	0.0000	-0.2936	0.6095	-4.5338	0.0000	-1.5020	0.0013	0.1136	0.8509	-0.3202	0.7299	0.2320	0.7788	-4.1808	0.0000	-2.9255	0.0000	-2.3245	0.0000
AT1G42980	-1.4194	0.0336	-1.0466	0.1676	-1.2638	0.1272	0.2996	0.7965	-1.5813	0.0414	-0.4285	0.6092	-2.3830	0.0001	-1.4700	0.0649	-0.3885	0.6248	-0.1935	0.9153	0.5428	0.5978	-2.4432	0.0002	-1.9578	0.0072	-0.9232	0.1551
AT1G43160	-2.0827	0.0050	-0.5635	0.5717	-0.4472	0.7089	0.0721	0.9665	-1.5942	0.0794	-0.5407	0.5726	-1.9818	0.0058	-0.8886	0.4923	0.3340	0.7268	-0.0620	0.9792	-1.9435	0.0276	-1.4507	0.0652	-1.8955	0.0228	-0.7967	0.2874
AT1G43910	-1.2490	0.1072	-2.1695	0.0036	-1.1520	0.2362	-0.6924	0.5582	0.0320	0.9915	-2.1185	0.0089	-1.2215	0.1034	-1.1652	0.2770	-2.5005	0.0006	-0.4501	0.7843	-0.4389	0.7400	-1.7187	0.0226	-2.1788	0.0054	-2.1093	0.0027
AT1G44000	-0.5428	0.1664	-0.6570	0.1108	-0.9470	0.0224	-0.6926	0.1688	-1.1639	0.0023	-1.0985	0.0061	-0.4411	0.2575	-1.0742	0.0073	-1.3439	0.0002	-0.9164	0.0376	0.0938	0.9083	-0.6145	0.1172	-1.0330	0.0087	-2.5967	0.0000
AT1G44100	-0.6341	0.3537	-1.4130	0.0279	-0.8321	0.3129	-0.7906	0.3829	0.1778	0.9026	-2.0575	0.0016	-0.3203	0.6624	-0.3788	0.7947	-1.9397	0.0013	0.2047	0.9023	1.0580	0.1909	-0.4274	0.5757	-0.9613	0.2367	-1.1178	0.0544
AT1G44130	-2.2077	0.0007	-2.5959	0.0001	-1.9522	0.0084	-1.7802	0.0331	-1.7338	0.0213	-1.9702	0.0081	-2.2355	0.0003	-2.3757	0.0007	-2.7427	0.0000	-1.9118	0.0137	-1.8741	0.0159	-2.4971	0.0001	-2.1053	0.0031	-1.6535	0.0105
AT1G44575	0.7011	0.1145	-0.3947	0.4624	-0.2740	0.6800	-0.1136	0.8936	-0.0295	0.9836	-0.8167	0.0878	0.9913	0.0154	0.0447	0.9773	-0.8782	0.0461	-0.6791	0.2680	0.1997	0.8037	0.6721	0.1404	-0.7007	0.2067	-2.0250	0.0000
AT1G44830	-2.8807	0.0000	-1.0762	0.1556	-0.7912	0.3927	-1.2154	0.1811	0.1239	0.9427	-1.8049	0.0138	-2.2370	0.0003	-0.8959	0.3907	-1.8872	0.0049	-1.8781	0.0143	-1.0073	0.2711	-2.9737	0.0000	-0.8583	0.3816	-0.4500	0.5128
AT1G46554	0.2444	0.7453	1.5619	0.0074	1.2777	0.0582	1.2983	0.0811	0.7761	0.3543	1.4597	0.0202	0.8416	0.1512	1.1470	0.1130	1.8992	0.0007	1.7207	0.0073	-0.1512	0.9055	0.8412	0.1775	1.5309	0.0135	2.5427	0.0000
AT1G47370	-0.8403	0.1909	-1.4447	0.0283	-1.6013	0.0247	-1.1445	0.1665	-0.7986	0.3724	-2.2142	0.0016	-0.5581	0.3846	-1.3814	0.0592	-1.4553	0.0245	-1.2846	0.1071	-0.0839	0.9515	-0.3933	0.5997	-1.5428	0.0301	-2.2482	0.0003
AT1G47395	3.2570	0.0000	0.0034	0.9986	-0.7148	0.5079	1.7560	0.0590	0.0099	1.0000	-0.4618	0.6268	3.8115	0.0000	0.5787	0.7129	0.2908	0.7579	1.2056	0.2446	1.1903	0.2278	5.2373	0.0000	-0.2579	0.8806	-0.6619	0.3752
AT1G47400	2.8696	0.0000	-0.0321	0.9797	-0.6078	0.5906	1.7481	0.0680	-0.3063	0.8558	-0.3270	0.7497	3.0987	0.0000	0.3576	0.8575	0.6469	0.4528	1.2154	0.2541	0.9190	0.3970	4.7335	0.0000	-0.5506	0.6999	-0.1492	0.8615
AT1G47510	-2.5112	0.0000	0.2035	0.8333	0.0136	0.9919	-0.4336	0.6902	-1.4187	0.0654	-0.2901	0.7396	-2.4507	0.0000	-1.4958	0.0502	0.3101	0.6997	0.1286	0.9494	-2.3926	0.0005	-2.3558	0.0001	-1.6909	0.0176	-0.8297	0.1904
AT1G47890	-1.1434	0.1311	-2.5036	0.0004	-1.6548	0.0496	-1.0142	0.3343	-0.5510	0.6798	-1.6401	0.0412	-1.8024	0.0088	-1.8240	0.0262	-2.0904	0.0040	-1.2677	0.2040	-1.1933	0.2206	-1.5204	0.0402	-1.6296	0.0496	-1.0323	0.1402
AT1G48120	-1.1764	0.0100	-1.5615	0.0005	-0.7744	0.1914	-0.7933	0.2162	-0.5851	0.3939	-1.4441	0.0038	-1.0857	0.0140	-0.6699	0.3324	-2.1458	0.0000	-0.7129	0.2885	-0.1409	0.8846	-1.2532	0.0060	0.0808	0.9447	-1.5207	0.0005
AT1G48745	-2.0891	0.0003	-1.1823	0.0742	-0.9165	0.2505	-1.4970	0.0512	-0.6809	0.4706	-1.7681	0.0079	-2.1814	0.0001	-1.4688	0.0359	-1.3641	0.0291	-0.0546	0.9779	0.6673	0.4570	-1.5999	0.0079	-1.8302	0.0043	-0.9041	0.1229
AT1G49000	-2.3923	0.0000	-2.2188	0.0000	-0.8271	0.1731	-1.4836	0.0094	-1.2436	0.0186	-1.1055	0.0367	-2.4570	0.0000	-2.0142	0.0000	-2.2552	0.0000	-1.7855	0.0004	-1.4275	0.0076	-2.8399	0.0000	-1.7896	0.0002	-0.7031	0.1279
AT1G49210	-0.3708	0.5642	-0.4277	0.5383	0.0747	0.9436	-0.1311	0.9028	1.1671	0.0595	0.4233	0.5251	-0.7243	0.1874	0.5418	0.5789	-0.3993	0.5252	-0.7178	0.3952	-2.0445	0.0005	-0.3742	0.5745	0.7252	0.3376	-0.5055	0.3511
AT1G49500	-1.9971	0.0016	1.0936	0.1385	0.8338	0.3515	0.7907	0.4235	-0.9487	0.3022	1.5133	0.0356	-2.0545	0.0007	0.1493	0.9438	1.3874	0.0397	0.6878	0.5508	-1.4643	0.0663	-1.7481	0.0069	0.6558	0.5469	1.4182	0.0218
AT1G49570	-0.1799	0.8624	2.4890	0.0005	0.3379	0.7827	-0.0926	0.9554	1.6304	0.0637	-0.2245	NA	-0.2415	0.8015	1.5932	0.0773	1.5942	NA	0.2679	0.8920	0.6474	0.5822	-0.0213	0.9876	0.0023	1.0000	0.0915	NA
AT1G49720	0.9259	0.0902	1.6150	0.0020	1.6110	0.0041	1.0670	0.1278	2.2891	0.0000	1.5973	0.0050	1.3221	0.0085	1.8402	0.0007	1.9002	0.0002	1.7630	0.0021	-0.7858	0.2908	1.6415	0.0013	2.5477	0.0000	1.9683	0.0001
AT1G49750	-0.4913	0.4308	-2.1984	0.0000	-1.3039	0.0347	-1.3943	0.0388	-0.2688	0.8164	-1.7792	0.0020	-0.6655	0.2409	-0.5749	0.5551	-2.2523	0.0000	-1.6821	0.0051	-0.0963	0.9379	-0.6970	0.2453	-0.7054	0.3730	-1.0837	0.0344
AT1G50110	-0.7015	0.2634	-1.0802	0.0904	-1.0512	0.1487	-0.3924	0.6846	0.9474	0.2120	-1.7579	0.0056	-0.5817	0.3495	0.1117	0.9544	-1.6708	0.0041	-1.2604	0.0828	-0.4823	0.6035	-0.6950	0.2826	-0.6688	0.4566	-2.1363	0.0001
AT1G50180	-1.5400	0.0226	-2.2586	0.0006	-1.7839	0.0176	-1.6551	0.0531	-1.6118	0.0369	-2.7815	0.0001	-1.9650	0.0019	-1.7259	0.0234	-2.3462	0.0004	-0.5209	0.6975	-0.1114	0.9430	-1.6648	0.0139	-2.0041	0.0048	-2.7967	0.0000
AT1G50420	-1.8720	0.0000	-1.2572	0.0000	-0.8775	0.0103	-1.0499	0.0037	-0.4544	0.2980	-0.8019	0.0193	-2.1169	0.0000	-0.4402	0.3531	-1.0461	0.0007	-0.1632	0.8194	-0.0291	0.9698	-1.8957	0.0000	-0.2771	0.6109	-0.5659	0.0579
AT1G50745	-1.1350	0.0979	-0.2323	0.8104	-0.3412	0.7487	0.0261	0.9886	-0.9853	0.2877	-0.5058	0.5438	-0.9693	0.1439	-1.1703	0.1901	-1.2079	0.0871	-1.2021	0.1892	2.8537	0.0000	-1.4066	0.0392	-1.1725	0.1713	-0.9280	0.1551
AT1G50750	-0.5398	0.4045	-0.1846	0.8280	-0.4750	0.5814	-0.0207	0.9899	-0.7145	0.4150	-0.4101	0.5781	-1.1527	0.0382	-1.5690	0.0181	-0.5242	0.4299	-1.4447	0.0469	3.1176	0.0000	-1.3857	0.0196	-0.7368	0.4032	-1.1239	0.0527
AT1G51090	0.1839	0.8481	1.9157	0.0058	0.9370	0.3139	0.3426	0.7804	0.6441	0.5844	0.4869	0.5778	0.5873	0.4443	1.2507	0.1690	2.0056	0.0033	2.1017	0.0060	0.6597	0.5357	1.8419	0.0067	2.5765	0.0002	2.8465	0.0000
AT1G51110	-0.0941	0.8826	-0.4969	0.3667	-0.3768	0.5737	-0.4758	0.4918	0.0229	0.9889	-0.8696	0.0847	-0.0091	0.9884	-0.3594	0.7073	-1.0509	0.0220	-0.9234	0.1099	-0.5607	0.3871	-0.0013	0.9996	-0.6185	0.3330	-2.4151	0.0000
AT1G51270	-1.2842	0.0034	-2.2915	0.0000	-0.8735	0.1143	-1.9074	0.0001	-0.5705	0.3939	-1.0320	0.0380	-1.5593	0.0002	-1.7010	0.0002	-2.4078	0.0000	-1.6489	0.0005	-1.4236	0.0039	-1.8337	0.0000	-2.0932	0.0000	-2.3437	0.0000
AT1G51610	1.2632	0.0014	0.5878	0.2215	1.3164	0.0021	0.7072	0.2154	2.0408	0.0000	0.5837	0.2233	1.6388	0.0000	1.4188	0.0007	0.8343	0.0501	1.0364	0.0327	0.1602	0.8442	1.8487	0.0000	1.6624	0.0000	0.8149	0.0369
AT1G51620	-1.5252	0.0013	-2.5485	0.0000	-1.3396	0.0134	-1.2334	0.0431	-0.6367	0.3705	-1.0256	0.0609	-2.0903	0.0000	-2.3387	0.0000	-2.2961	0.0000	-1.8491	0.0004	-1.3912	0.0129	-2.1190	0.0000	-2.1788	0.0000	-2.5773	0.0000
AT1G51670	-1.4646	0.0245	-1.3416	0.0573	-2.0055	0.0051	0.3032	0.7894	-1.5720	0.0369	-0.6066	0.4466	-1.8601	0.0022	-0.8657	0.3922	-0.7477	0.2961	-0.6612	0.5629	0.0789	0.9587	-1.4426	0.0289	-0.7952	0.4176	-1.4166	0.0246
AT1G51790	-1.3722	0.0153	-2.4479	0.0000	-0.9131	0.2160	-1.7881	0.0083	-0.8505	0.2806	-1.7574	0.0042	-1.8517	0.0005	-1.9423	0.0010	-2.9592	0.0000	-1.6812	0.0087	-1.3701	0.0433	-1.7617	0.0014	-2.2927	0.0000	-2.1758	0.0000
AT1G51805	0.2022	0.8047	-2.0217	0.0006	-0.8136	0.3234	-0.9447	0.2746	-0.4994	0.6489	-1.5005	0.0245	-0.2557	0.7347	-0.8756	0.3385	-1.9880	0.0009	0.0135	0.9945	0.2627	0.8230	0.0258	0.9811	-1.0040	0.2042	-1.5430	0.0071
AT1G51820	-1.7041	0.0064	-3.1167	0.0000	-2.0316	0.0024	-0.9160	0.3190	-2.2319	0.0005	-2.5110	0.0002	-2.7110	0.0000	-2.9763	0.0000	-3.2275	0.0000	-1.9034	0.0076	-1.9584	0.0053	-2.6595	0.0000	-3.3937	0.0000	-2.9576	0.0000
AT1G51850	-1.2332	0.0552	-3.0074	0.0000	-1.4443	0.0494	-1.9813	0.0084	-1.0729	0.1964	-2.3592	0.0004	-1.7585	0.0032	-2.0576	0.0019	-2.8944	0.0000	-1.2007	0.1528	-1.4139	0.0670	-1.7993	0.0037	-2.7306	0.0000	-2.4417	0.0000
AT1G51860	-2.0065	0.0009	-2.3963	0.0001	-1.7217	0.0123	-1.7212	0.0248	-1.0270	0.2139	-1.5950	0.0198	-2.0378	0.0004	-2.2754	0.0004	-2.6025	0.0000	-0.8888	0.3467	-1.7996	0.0108	-1.6721	0.0066	-2.3390	0.0002	-1.6568	0.0052
AT1G51890	-2.6264	0.0000	-3.1253	0.0000	-1.5732	0.0144	-1.6177	0.0239	-1.3830	0.0380	-1.1883	0.0677	-2.5947	0.0000	-2.5378	0.0000	-2.6631	0.0000	-1.7491	0.0073	-2.0860	0.0007	-2.7484	0.0000	-2.2729	0.0001	-1.7499	0.0015
AT1G51900	-0.1536	0.8749	-0.0291	0.9798	0.0462	0.9745	0.4164	0.7263	-0.2770	0.8565	0.0776	NA	-0.2844	0.7416	0.6682	0.6136	-0.0738	0.9394	-0.1739	0.9297	-0.2322	0.8678	-0.4870	0.5698	-0.0458	0.9839	2.1432	0.0010
AT1G51920	-2.3424	0.0005	-2.4001	0.0005	-1.9691	0.0111	-1.3376	0.1499	-1.0597	0.2643	-1.0381	NA	-2.6012	0.0001	-1.9937	0.0091	-2.4456	0.0005	-2.3248	0.0028	-1.9701	0.0144	-2.4259	0.0003	-1.6051	0.0452	-0.2096	0.7818
AT1G52030	-0.6331	0.3301	-2.5991	0.0000	-2.2692	0.0001	-0.1355	0.9094	-1.2932	0.0618	-0.6671	0.3408	-0.3685	0.5895	-0.3694	0.7899	-0.5021	0.4557	-0.2770	0.8402	-0.7626	0.3633	-0.5131	0.4631	-0.8564	0.2901	-1.2502	0.0250
AT1G52240	-0.8118	0.1440	-0.5260	0.4237	-0.7756	0.2718	-0.3175	0.7241	-0.7208	0.3476	-1.2038	0.0457	-0.7271	0.1751	-0.4961	0.6254	-1.1034	0.0476	-1.4471	0.0207	0.7571	0.2923	-0.6787	0.2435	-0.1350	0.9174	-2.1920	0.0000
AT1G52290	0.4579	0.5785	-2.0664	0.0023	-0.8411	0.3700	-1.0869	0.2597	-0.3476	0.8101	-1.5399	0.0410	-0.2225	0.8002	-0.3442	0.8457	-2.4265	0.0003	-0.7704	0.5039	0.4290	0.7180	0.0479	0.9671	-0.9689	0.3098	-1.6171	0.0121
AT1G52565	-3.5863	0.0000	-1.7278	0.0020	-1.4758	0.0187	-1.5555	0.0244	-1.2208	0.0671	-1.6697	0.0064	-3.6099	0.0000	-1.8145	0.0022	-2.3056	0.0000	-1.2880	0.0629	-0.4978	0.5719	-3.5174	0.0000	-1.7386	0.0031	-1.7064	0.0014
AT1G52690	0.4861	0.5820	1.8393	0.0144	0.5450	0.6233	0.4130	0.7459	0.5527	0.6826	0.2355	0.8188	0.0114	0.9913	0.1178	0.9640	1.3967	0.0660	0.8308	0.4990	1.2016	0.2206	3.2615	0.0000	0.4097	0.7858	0.5639	0.4472
AT1G52830	-3.1573	0.0000	-0.8764	0.2391	-0.1823	0.8702	-1.2073	0.1582	-0.1133	0.9459	-0.2538	0.7685	-2.8315	0.0000	-0.0302	0.9921	-0.9641	0.1539	-1.2791	0.1199	-0.2462	0.8431	-2.6125	0.0000	0.6438	0.5352	-0.4817	0.4560
AT1G52855	-2.1705	0.0002	0.2565	0.7683	0.0595	0.9617	-0.1324	0.9135	-0.5331	0.6139	0.4881	0.5147	-1.7115	0.0027	-1.0890	0.1784	-0.0467	0.9575	-0.1648	0.9231	-1.4679	0.0447	-1.4505	0.0179	0.3089	0.8087	0.5275	0.3830
AT1G52890	-1.7727	0.0039	0.3119	0.7260	0.2207	0.8379	0.8673	0.3433	0.0519	0.9802	1.1303	0.1111	-1.5185	0.0116	-0.1425	0.9439	0.7302	0.2917	0.5568	0.6411	-2.7221	0.0000	-1.2228	0.0604	0.8876	0.3137	1.1687	0.0495
AT1G53035	0.1652	0.8554	0.1904	0.8447	1.6149	0.0247	-0.1227	0.9291	2.4310	0.0001	0.0027	0.9982	1.2011	0.0560	1.4651	0.0514	-0.0669	0.9424	0.6053	0.6131	-0.3672	0.7544	1.3030	0.0477	1.7739	0.0092	1.3544	0.0259
AT1G53080	-1.9427	0.0044	-1.6221	0.0269	-0.2741	0.8094	-2.0968	0.0122	-0.8454	0.4099	-1.6924	NA	-1.9236	0.0029	-1.5288	0.0638	-1.1115	0.1294	-1.3886	0.1219	-0.6314	0.5522	-2.2295	0.0010	-1.0295	0.2756	-0.6293	0.3615
AT1G53090	-2.1854	0.0000	-0.9452	0.0312	-0.8779	0.0765	-1.0896	0.0338	-1.2876	0.0029	-1.4948	0.0007	-1.7843	0.0000	-1.3007	0.0031	-1.2452	0.0026	-0.0927	0.9402	0.3206	0.6385	-1.5090	0.0001	-1.5557	0.0002	-1.5404	0.0001
AT1G53100	-0.0642	0.9548	0.7068	0.4551	0.9299	0.3692	0.2403	0.8704	2.1464	0.0073	0.4165	NA	0.5167	0.5525	0.5144	0.7661	-0.1741	0.8626	0.4352	0.7947	0.1302	0.9392	1.7512	0.0211	1.7073	0.0462	0.5279	0.4936
AT1G53180	-1.6935	0.0001	-0.3851	0.4840	0.0009	0.9996	-0.6068	0.3348	0.3076	0.7039	-0.5015	0.3367	-2.1959	0.0000	0.2316	0.8321	-0.4482	0.3607	-0.3989	0.6220	-0.6195	0.3099	-1.1880	0.0059	0.4620	0.5026	0.1814	0.6990
AT1G53490	0.7756	0.1184	1.2511	0.0099	1.6030	0.0010	1.4215	0.0117	2.1128	0.0000	1.3053	0.0119	0.5967	0.2259	2.0327	0.0000	1.2370	0.0099	1.3024	0.0165	1.1390	0.0448	0.8495	0.0878	1.8352	0.0001	1.0789	0.0179
AT1G53620	-2.1440	0.0036	-2.3456	0.0015	-1.6606	0.0616	-2.0826	0.0248	-1.5488	0.0908	-2.0611	NA	-2.2834	0.0013	-2.1865	0.0073	-2.4545	NA	-2.0352	0.0192	-1.9609	0.0246	-1.9703	0.0086	-1.9349	0.0180	-1.6647	NA
AT1G53625	-3.5404	0.0000	-2.7832	0.0000	-1.8570	0.0024	-1.9114	0.0047	-2.1003	0.0003	-1.4440	0.0235	-3.6792	0.0000	-2.7340	0.0000	-2.5317	0.0000	-2.4041	0.0001	-3.1766	0.0000	-3.3187	0.0000	-3.0792	0.0000	-2.0614	0.0002
AT1G53633	0.0032	0.9985	0.7846	0.3291	0.8097	0.3784	0.3007	0.8060	1.0277	0.2528	1.0237	0.1819	-0.3685	0.6448	0.9190	0.3625	1.1533	0.0967	0.8312	0.4352	0.1962	0.8934	-0.0088	0.9947	0.9198	0.3323	2.7459	0.0000
AT1G53640	-0.3799	0.6801	-0.5457	0.5607	-0.5196	0.6344	-0.2882	0.8325	0.0851	0.9689	-0.1998	0.8474	-0.5806	0.4852	0.0667	0.9800	-0.2542	0.7849	-0.5501	0.7003	0.3105	0.8278	-0.6412	0.4593	0.0191	0.9964	5.8946	0.0000
AT1G53700	-2.1547	0.0003	-1.3424	0.0444	-0.1716	0.8769	-1.1443	0.1786	-0.1429	0.9267	-1.4628	0.0347	-1.7883	0.0022	-0.3879	0.7919	-1.8926	0.0025	-1.1924	0.1489	-0.7070	0.4425	-1.7054	0.0057	-0.5298	0.6334	-1.2808	0.0315
AT1G53830	-2.0262	0.0003	0.3136	0.6855	1.0506	0.1278	-0.0975	0.9357	-0.1147	0.9367	0.6413	0.3373	-1.8968	0.0004	-0.5299	0.6299	0.2102	0.7714	0.0627	0.9726	-1.3281	0.0591	-2.3212	0.0000	0.9187	0.2065	0.4998	0.3766
AT1G53870	-0.4019	0.6695	0.1133	0.9236	0.0831	0.9559	0.3317	0.8110	2.9798	0.0000	0.5639	0.5508	-0.4540	0.6072	0.1347	0.9588	0.1006	0.9222	0.0847	0.9726	-0.2923	0.8428	-0.3172	0.7576	0.8423	0.4756	1.8710	0.0083
AT1G53875	-0.9422	0.2326	0.1135	0.9223	0.0870	0.9530	-0.3245	0.8117	2.0487	0.0100	-0.0687	NA	-0.9252	0.2287	-0.4323	0.8100	-0.2428	0.7995	-0.1736	0.9359	-0.6223	0.5937	-0.3517	0.7218	-0.0657	0.9767	0.1704	0.8364
AT1G53880	-0.6157	0.4821	-0.1012	0.9333	0.6312	0.5736	-0.3935	0.7674	3.4457	0.0000	-0.3730	0.7076	-0.6806	0.4109	-0.3861	0.8420	-0.4148	0.6508	-0.4113	0.8082	-0.2616	0.8635	-0.5598	0.5449	-0.1927	0.9170	0.2039	0.8067
AT1G53885	0.2177	0.8323	0.7736	0.4054	0.4906	0.6735	1.0639	0.3296	3.5531	0.0000	0.9584	0.2762	0.2067	0.8330	0.6004	0.7067	1.1102	0.1659	0.8927	0.4709	-0.0125	0.9988	0.5052	0.5930	1.0491	0.3213	1.6578	0.0193
AT1G54870	-0.1740	0.8549	2.3855	0.0003	0.5652	0.5794	-0.1715	0.8997	-0.1407	0.9350	0.0228	NA	-0.2169	0.8066	0.4026	0.8100	0.6828	0.3753	0.3739	0.8084	1.9506	0.0123	0.2094	0.8321	-0.1195	0.9437	0.3228	0.6537
AT1G54960	0.2421	0.7210	-0.6561	0.3094	-0.8425	0.2287	-0.0141	0.9926	-0.2175	0.8551	-1.0807	0.0769	0.2942	0.6378	-0.2450	0.8621	-1.2276	0.0282	-0.6352	0.4741	-0.5506	0.4986	0.4206	0.5108	-0.7184	0.3579	-2.0856	0.0001
AT1G55110	-2.3266	0.0000	-0.7943	0.0669	-0.7084	0.1593	-0.4821	0.4230	-1.0105	0.0228	0.0346	0.9563	-2.4864	0.0000	-0.5849	0.3253	-0.0710	0.8956	0.0994	0.9312	-0.6364	0.2394	-1.9779	0.0000	-0.4221	0.5153	-0.1314	0.7642
AT1G55480	-0.2513	0.6681	-0.6409	0.2424	-0.3930	0.5723	-0.8044	0.2141	-0.2033	0.8435	-1.0165	0.0492	-0.0204	0.9753	-0.6075	0.4163	-1.4217	0.0020	-0.9721	0.1026	-0.4132	0.5731	-0.3372	0.5600	-1.1715	0.0220	-2.8467	0.0000
AT1G55910	-1.5979	0.0124	-1.7797	0.0065	-1.2862	0.1012	-1.3999	0.0970	-0.9263	0.3088	-2.2472	0.0010	-1.3135	0.0368	-1.8963	0.0065	-2.7191	0.0000	-0.5418	0.6625	0.0811	0.9579	-1.4873	0.0225	-1.5343	0.0341	-1.0408	0.0923
AT1G55920	-2.3352	0.0000	-0.4858	0.3723	-0.2850	0.6801	-0.6173	0.3394	-0.4505	0.5348	-0.4610	0.3894	-2.2236	0.0000	-0.8231	0.1552	-0.5893	0.2208	-0.3640	0.6706	-0.6658	0.2756	-1.6929	0.0000	-1.4766	0.0011	-0.8723	0.0381
AT1G55960	-3.6243	0.0000	-2.3732	0.0000	-2.5064	0.0000	-2.5969	0.0000	-3.1505	0.0000	-2.5306	0.0000	-3.7403	0.0000	-3.3789	0.0000	-2.8723	0.0000	-1.8816	0.0000	-1.3393	0.0018	-3.4978	0.0000	-2.8322	0.0000	-3.2163	0.0000
AT1G56050	1.0441	0.0386	-0.5040	0.4201	-0.8055	0.2160	0.0052	0.9976	-0.4145	0.6468	-0.7727	0.1823	1.2177	0.0108	0.3891	0.7198	-1.0790	0.0376	-0.6857	0.3769	0.1332	0.9034	1.0494	0.0405	-0.6669	0.3600	-2.0418	0.0000
AT1G56060	-3.7314	0.0000	-2.3062	0.0001	-1.8456	0.0057	-1.5393	0.0486	-2.5802	0.0000	-1.2978	0.0601	-2.9228	0.0000	-3.0723	0.0000	-3.1965	0.0000	-2.3400	0.0004	-2.5164	0.0001	-4.1150	0.0000	-3.0827	0.0000	-2.4916	0.0000
AT1G56120	-1.7350	0.0080	-2.5350	0.0001	-1.8341	0.0119	-1.5374	0.0720	-0.4192	0.7473	-2.6296	0.0002	-1.9193	0.0021	-1.1810	0.1828	-2.5563	0.0001	-0.4878	0.7179	0.0012	1.0000	-1.4022	0.0390	-1.5012	0.0473	-1.7185	0.0062
AT1G56250	-2.0116	0.0048	-2.7343	0.0001	-1.7244	0.0391	-1.4262	0.1400	-2.4353	0.0010	-1.8544	0.0200	-2.1303	0.0018	-1.6835	0.0480	-1.8631	0.0114	-1.5039	0.1071	0.2140	0.8894	-1.9619	0.0062	-2.7041	0.0002	-2.5348	0.0002
AT1G56300	2.8079	0.0000	2.7922	0.0000	3.0667	0.0000	2.3579	0.0018	3.6422	0.0000	2.8634	0.0000	3.1331	0.0000	2.8699	0.0000	3.1744	0.0000	3.4344	0.0000	-0.3786	0.7522	3.3504	0.0000	3.9451	0.0000	3.1947	0.0000
AT1G56430	1.1458	0.0852	-0.4955	0.5589	-0.5474	0.5725	0.9715	0.2938	0.2361	0.8728	-0.6152	0.4423	1.7033	0.0053	0.6142	0.6217	-0.3204	0.6913	0.6387	0.5838	1.5440	0.0441	2.0596	0.0009	-0.2986	0.8336	-0.7809	0.2218
AT1G56510	-0.6488	0.3472	-2.0290	0.0008	-1.2409	0.0974	-1.3102	0.1056	-1.0931	0.1713	-1.3999	0.0402	-1.4697	0.0130	-1.6093	0.0197	-2.4044	0.0001	-0.6388	0.5590	0.6502	0.4845	-1.1542	0.0728	-1.3623	0.0548	-2.2417	0.0001
AT1G56520	-1.8112	0.0003	-1.6944	0.0009	-0.9308	0.1541	-1.5601	0.0123	-0.8717	0.2037	-1.1289	0.0506	-1.6315	0.0007	-1.1600	0.0608	-2.0651	0.0000	-0.9777	0.1581	-0.0477	0.9705	-1.7917	0.0003	-1.2144	0.0386	-1.8299	0.0002
AT1G56550	-1.2623	0.0092	-1.0276	0.0525	-0.7093	0.2759	-2.2145	0.0000	-0.6565	0.3550	-1.3626	0.0133	-1.8434	0.0000	-2.0179	0.0001	-1.6935	0.0007	-1.3418	0.0195	-1.8753	0.0005	-1.4919	0.0019	-0.1648	0.8840	-0.4508	0.3602
AT1G56660	-2.1003	0.0011	-1.5433	0.0273	-1.1693	0.1650	-1.7821	0.0299	-1.5153	0.0504	-0.4857	0.5635	-2.8163	0.0000	-1.3277	0.1130	-1.1601	0.0978	-1.3496	0.1170	0.1664	0.9101	-2.0737	0.0013	-1.5460	0.0398	-1.6325	0.0094
AT1G57560	-2.2427	0.0004	-2.2170	0.0007	-1.1305	0.1783	-1.2135	0.1753	-0.6823	0.5186	-1.7312	0.0173	-2.1615	0.0004	-1.5247	0.0485	-2.8306	0.0000	-1.7530	0.0228	-0.9703	0.2869	-2.1309	0.0008	-1.7170	0.0179	-1.7150	0.0066
AT1G57590	0.2608	0.7942	-0.6204	0.5172	-0.1928	0.8875	0.0253	0.9906	0.5088	0.7233	-0.8607	0.3318	0.1098	0.9131	-0.5124	0.7645	-0.9487	0.2428	-0.1765	0.9359	-0.1329	0.9379	2.2323	0.0022	0.6071	0.6555	-0.9726	0.1778
AT1G57630	-3.1683	0.0000	-3.1659	0.0000	-1.7726	0.0188	-1.1598	0.2187	-1.1140	0.2160	-1.8305	0.0133	-2.9531	0.0000	-2.9353	0.0000	-2.8943	0.0000	-2.3436	0.0013	-2.3293	0.0013	-3.6790	0.0000	-2.6682	0.0001	-1.9037	0.0029
AT1G57650	-3.3284	0.0000	-2.2455	0.0003	-1.3179	0.0745	-1.9662	0.0081	-1.7057	0.0122	-1.0885	NA	-2.7408	0.0000	-2.6609	0.0000	-2.6221	0.0000	-2.2394	0.0011	-2.2032	0.0014	-3.2328	0.0000	-2.5534	0.0001	-1.5797	0.0089
AT1G57750	0.3787	0.6333	0.0834	0.9346	0.0755	0.9524	0.2607	0.8255	0.4601	0.7020	0.0000	NA	0.6665	0.3391	0.6954	0.5411	0.0000	1.0000	0.8536	0.3958	2.0555	0.0036	0.4497	0.5745	0.1032	0.9478	0.0000	1.0000
AT1G57800	0.1099	0.9011	0.3879	0.6439	0.2068	0.8454	0.4588	0.6560	-0.0365	0.9861	-0.1293	NA	-0.2092	0.7942	0.1819	0.9216	0.0980	0.9062	0.0769	0.9696	-0.1433	0.9148	-0.1774	0.8442	0.0074	0.9992	2.5338	0.0000
AT1G58170	-1.7123	0.0116	-0.9641	0.2277	-1.6333	0.0388	-0.7383	0.4803	-0.3413	0.8133	-2.2507	0.0026	-1.0385	0.1304	-1.1476	0.2235	-1.8980	0.0063	-0.1187	0.9560	-0.2505	0.8530	-1.3566	0.0543	-1.5803	0.0439	-2.3672	0.0003
AT1G58420	-2.0314	0.0025	-1.7282	0.0155	-0.9503	0.3027	-1.1764	0.2166	0.2851	0.8506	-1.0689	0.1776	-1.8373	0.0046	-1.1844	0.2039	-1.5074	0.0339	-0.9084	0.3989	0.7395	0.4654	-2.1826	0.0011	-0.9704	0.3157	-0.6506	0.3422
AT1G58520	-1.8907	0.0001	-1.0383	0.0640	-1.3832	0.0159	-0.9696	0.1681	-1.8631	0.0003	-1.9851	0.0003	-2.0357	0.0000	-1.9121	0.0003	-1.4505	0.0050	-0.0819	0.9611	0.4601	0.5706	-1.0589	0.0464	-0.7642	0.2810	-1.8862	0.0001
AT1G58602	-1.6678	0.0036	-2.4288	0.0000	-1.2057	0.0879	-1.3759	0.0679	-0.7823	0.3610	-1.0457	0.1161	-1.6330	0.0032	-0.9400	0.2584	-1.9090	0.0009	-0.6313	0.5375	-0.1382	0.9149	-1.5580	0.0073	-1.4192	0.0298	-1.8110	0.0010
AT1G58848	-1.1860	0.0880	-1.0867	0.1561	-0.8575	0.3516	-0.7154	0.4905	2.0386	0.0035	-0.6952	0.3925	-1.2055	0.0706	-0.6913	0.5761	-0.9737	0.1775	-0.2889	0.8611	0.3160	0.8033	-1.2166	0.0841	-0.5652	0.6394	-0.8508	0.1954
AT1G59124	-1.1888	0.1132	-0.3398	0.7407	-0.7776	0.4500	-0.1629	0.9107	3.1202	0.0000	-0.7355	0.4002	-0.1492	0.8744	-0.3122	0.8726	-0.4649	0.5898	0.1829	0.9295	0.3967	0.7582	-0.9714	0.2189	0.1722	0.9233	-0.6043	0.4065
AT1G59850	-1.1952	0.1085	-0.8163	0.3491	-0.7328	0.4782	-0.7047	0.5248	-0.9620	0.3579	-1.1459	NA	-0.7326	0.3325	-0.5777	0.6980	-1.0012	0.1979	-1.2367	0.2183	0.4369	0.7208	-0.7387	0.3673	-0.5170	0.7037	-2.1211	0.0022
AT1G59930	0.6048	0.4859	0.5073	0.6125	1.4348	0.1123	0.0679	0.9674	1.5605	0.0761	0.8454	NA	0.9470	0.2139	0.7311	0.6010	0.1131	0.9126	0.4215	0.8004	0.6097	0.6148	0.7099	0.4149	1.1947	0.2214	3.1558	0.0000
AT1G60270	-0.8214	0.1960	-1.9181	0.0011	-1.7414	0.0069	-0.6993	0.4377	-1.7037	0.0073	-2.0888	0.0010	-1.3270	0.0205	-1.6692	0.0104	-2.0508	0.0005	-0.6537	0.5225	-0.0520	0.9723	-0.5464	0.4369	-0.9769	0.2087	-2.3145	0.0000
AT1G60590	-1.0478	0.1445	-0.8278	0.3132	-1.7583	0.0215	-0.4230	0.7161	-1.2051	0.1724	-1.6892	0.0245	-1.0828	0.1151	-1.0858	0.2632	-1.4305	0.0426	-0.0255	0.9904	0.9242	0.3371	0.2987	0.7477	0.2734	0.8582	-2.2104	0.0006
AT1G61120	-1.5757	0.0335	-2.5179	0.0004	-1.6896	0.0477	0.1205	0.9399	-1.6185	0.0622	-1.2044	0.1529	-1.4839	0.0388	-2.1590	0.0066	-2.0787	0.0047	-0.4561	0.7737	-0.8522	0.4310	-1.7742	0.0163	-0.9498	0.3802	-1.2454	0.0767
AT1G61255	-1.6377	0.0112	-0.1010	0.9204	0.2118	0.8474	-1.1047	0.2199	-0.4559	0.7087	-0.8788	0.2482	-2.1386	0.0004	-1.0006	0.2844	-0.6125	0.4042	-0.2447	0.8837	-0.0392	0.9822	-0.7913	0.2710	0.2253	0.8790	-0.5441	0.4070
AT1G61360	-1.9681	0.0000	-1.8746	0.0000	-0.4951	0.4209	-0.9278	0.1050	-0.9187	0.0802	-0.5369	0.2991	-1.8595	0.0000	-1.2247	0.0105	-1.6691	0.0001	-1.0326	0.0521	-1.0847	0.0355	-2.2676	0.0000	-1.1855	0.0119	-1.1703	0.0048
AT1G61380	-1.6883	0.0003	-2.0481	0.0000	-0.7259	0.2605	-1.3395	0.0255	-0.3036	0.7531	-1.5524	0.0030	-1.6965	0.0002	-1.1649	0.0395	-2.5126	0.0000	-1.2483	0.0302	-1.2008	0.0373	-1.7590	0.0001	-1.3245	0.0116	-1.8831	0.0000
AT1G61550	-4.9786	0.0000	-4.8806	0.0000	-4.4941	0.0000	-4.2362	0.0000	-4.0509	0.0000	-4.6338	0.0000	-5.0888	0.0000	-4.8760	0.0000	-4.6312	0.0000	-3.9448	0.0000	-4.7687	0.0000	-5.5993	0.0000	-5.0739	0.0000	-4.9725	0.0000
AT1G61590	0.9932	0.1966	0.9783	0.2512	1.4768	0.0874	0.7149	0.5275	0.5839	0.6593	0.4669	0.6180	-0.0240	0.9811	1.2411	0.2031	0.8866	0.2653	1.5007	0.1008	2.2891	0.0031	0.7626	0.3589	0.8109	0.4811	2.9047	0.0000
AT1G61610	-1.8837	0.0019	-1.4840	0.0230	-2.1274	0.0012	-0.3149	0.7738	-1.2062	0.1185	-1.5623	0.0234	-1.9941	0.0005	-1.6415	0.0181	-1.4639	0.0221	-0.5243	0.6570	-1.2782	0.1037	-1.3369	0.0346	-1.5483	0.0259	-1.7607	0.0032
AT1G61860	-0.6260	0.3850	0.3523	0.6791	0.2807	0.7855	0.0088	0.9967	0.9748	0.2320	-0.0043	0.9972	0.1801	0.8189	0.3033	0.8511	-0.4280	0.5801	0.3728	0.7846	2.0151	0.0015	-0.3581	0.6607	-0.0175	0.9961	-0.0606	0.9364
AT1G61890	-0.8674	0.0342	0.5585	0.2414	0.3063	0.6191	0.6482	0.2579	-0.8623	0.0771	0.5092	0.2873	-0.7228	0.0727	-0.1420	0.9029	1.0903	0.0073	0.8687	0.0889	2.2563	0.0000	-0.7843	0.0630	-0.1828	0.8385	0.4934	0.2140
AT1G62180	0.3729	0.6679	-1.5935	0.0285	-1.2116	0.1664	0.2652	0.8370	-0.1908	0.9082	-1.8807	0.0126	-0.0929	0.9215	-0.3154	0.8636	-2.2245	0.0011	0.3394	0.8327	0.6870	0.5158	0.0586	0.9598	-1.5134	0.0576	-1.9362	0.0030
AT1G62262	2.0319	0.0055	-0.0717	0.9537	-0.2676	0.8324	0.2800	0.8424	1.1166	0.2814	-0.2005	NA	1.6218	0.0247	1.1723	0.2750	-0.0407	NA	0.8516	0.4996	0.5489	0.6603	1.1100	0.1702	0.5103	0.7218	0.8415	NA
AT1G62420	-2.0823	0.0014	-0.5242	0.5466	-0.2489	0.8277	-0.3120	0.7968	-1.1408	0.1964	1.7729	0.0143	-1.1250	0.0926	-0.7874	0.4900	-0.6751	0.3719	-1.3909	0.1075	-3.1286	0.0000	-1.1584	0.1005	-1.8550	0.0106	-0.1911	0.7964
AT1G62440	-1.3868	0.0338	-0.5131	0.5414	-0.2799	0.7960	-0.3535	0.7571	-0.7016	0.4958	-0.0139	0.9908	-2.5040	0.0000	-0.7078	0.5367	-0.4328	0.5759	-1.3944	0.0882	-2.7789	0.0000	-2.2750	0.0002	-0.2328	0.8763	-0.1724	0.8112
AT1G62570	-0.3966	0.6120	1.3405	0.0484	0.5378	0.5708	0.7761	0.4172	-0.0180	0.9951	0.5731	0.4669	-0.5355	0.4508	0.9019	0.3506	1.7845	0.0048	1.5485	0.0415	-0.3603	0.7518	1.0690	0.1114	2.3196	0.0002	1.9079	0.0014
AT1G62760	-1.9035	0.0004	-1.0510	0.0847	-0.3408	0.6957	-0.6589	0.4311	-1.3604	0.0313	-0.4282	0.5385	-2.1686	0.0000	-1.2429	0.0603	-0.4050	0.5325	-0.6917	0.4380	-1.8839	0.0018	-2.2078	0.0000	-1.3694	0.0277	-0.6146	0.2620
AT1G62770	-0.4409	0.5518	0.4182	0.6130	0.9131	0.2620	0.3098	0.7785	0.5623	0.5957	1.0083	0.1529	-1.4569	0.0141	0.9836	0.2607	0.9834	0.1324	1.2762	0.1060	-1.0366	0.2125	-0.8276	0.2234	1.4018	0.0452	2.1631	0.0002
AT1G62780	0.2114	0.7846	-0.6128	0.3939	-0.6129	0.4624	-0.5601	0.5424	-0.0083	0.9998	-0.9421	0.1573	0.3698	0.5858	0.3048	0.8381	-1.2610	0.0351	-0.7535	0.4134	-0.4362	0.6517	0.1859	0.8236	-0.8212	0.3149	-2.2528	0.0000
AT1G62975	-0.9299	0.1075	-0.2930	0.7031	-0.0304	0.9794	-0.2544	0.7987	-0.9442	0.2032	-0.0659	0.9381	-2.1740	0.0000	-0.6739	0.4670	0.0224	0.9792	0.0915	0.9584	-0.3555	0.7120	-0.7168	0.2412	-0.1664	0.9011	0.1880	0.7554
AT1G63820	-1.2165	0.1060	-0.9902	0.2471	-0.5057	0.6473	-0.1699	0.9068	-0.1140	0.9534	-1.5644	NA	-1.0029	0.1732	-0.1602	0.9452	-2.0571	0.0052	-0.7115	0.5857	0.4252	0.7358	-0.7850	0.3404	-0.4911	0.7255	-0.9253	0.1941
AT1G63840	-2.3748	0.0000	-1.1887	0.0192	-1.3104	0.0154	-0.6444	0.3680	-1.2408	0.0222	-1.1193	0.0381	-2.3300	0.0000	-1.4591	0.0054	-1.1685	0.0191	-0.2358	0.8356	-0.0488	0.9669	-1.7996	0.0001	-1.2937	0.0145	-0.4118	0.4025
AT1G64110	-0.1064	0.9230	2.9635	0.0000	1.0274	0.3142	0.1500	0.9243	0.7063	0.5846	0.3007	0.7713	-0.1710	0.8641	0.5831	0.7217	1.2295	0.1247	1.4209	0.1583	2.6420	0.0009	4.0906	0.0000	1.2608	0.1974	1.4844	0.0381

AT1G64360	0.0754	0.9438	-2.1421	0.0029	-1.5575	0.0680	-0.8285	0.4526	0.9600	0.3610	-1.3987	0.0841	0.1990	0.8327	0.0748	0.9774	-1.7390	0.0178	0.5476	0.7043	0.3936	0.7627	0.1939	0.8557	-0.2988	0.8518	-0.7635	0.2839
AT1G64500	-3.0532	0.0000	-1.9833	0.0002	-2.0894	0.0001	-1.7881	0.0052	-3.0628	0.0000	-1.9112	0.0010	-3.1944	0.0000	-2.2321	0.0000	-1.8255	0.0007	-0.4813	0.6448	0.1360	0.9106	-2.4429	0.0000	-2.6195	0.0000	-1.6446	0.0014
AT1G64590	0.3919	0.6746	0.5478	0.5729	-0.0474	0.9756	-0.0946	0.9540	-0.0437	0.9861	0.0155	NA	0.7920	0.3208	0.7277	0.6032	-0.0282	NA	1.1953	0.2607	2.3067	0.0047	0.9314	0.2599	1.0841	0.2875	0.4609	NA
AT1G64660	-3.2334	0.0000	0.3220	0.7107	0.2690	0.7948	0.2505	0.8269	-0.0747	0.9674	0.4304	0.5836	-3.2285	0.0000	0.2190	0.9020	0.8751	0.1887	0.3746	0.7833	-1.6767	0.0179	-2.4432	0.0000	-0.0751	0.9643	1.5008	0.0103
AT1G64780	-1.4828	0.0174	-1.5457	0.0174	-1.9069	0.0043	-0.5694	0.5682	-2.2528	0.0003	-1.8656	0.0060	-1.6661	0.0050	-1.5597	0.0286	-1.2121	0.0631	0.0625	0.9760	0.9013	0.3002	-0.8308	0.2285	-1.9677	0.0024	-1.2400	0.0370
AT1G64860	-0.9359	0.0387	-0.4913	0.3721	-0.6171	0.3063	-0.7869	0.2045	-0.8107	0.1573	-0.8043	0.1130	-1.0034	0.0203	-0.6764	0.3048	-1.0607	0.0204	-0.6293	0.3578	-0.0642	0.9506	-1.1329	0.0113	-0.4226	0.5792	-2.0904	0.0000
AT1G64890	1.6880	0.0002	1.6921	0.0002	1.8170	0.0001	1.4677	0.0094	2.1350	0.0000	1.3934	0.0073	2.2850	0.0000	1.7575	0.0002	1.8831	0.0000	1.7218	0.0006	-0.0015	1.0000	2.0176	0.0000	2.6465	0.0000	2.6323	0.0000
AT1G65230	0.0511	0.9050	-0.4677	0.1774	-0.2669	0.5503	-0.6369	0.1074	-0.4799	0.2381	-1.1150	0.0005	0.1315	0.7209	-0.8963	0.0058	-1.3383	0.0000	-1.0984	0.0006	-0.2024	0.6971	-0.0315	0.9494	-0.6599	0.0589	-2.1483	0.0000
AT1G65250	-1.1947	0.0405	-1.5031	0.0114	-0.6162	0.4556	-1.3592	0.0663	-0.2404	0.8509	-1.0777	0.0988	-1.2738	0.0219	-1.0724	0.1544	-2.0438	0.0003	-1.0656	0.1715	-0.5840	0.5058	-1.4561	0.0116	-0.8644	0.2720	-1.2825	0.0195
AT1G65310	-1.7809	0.0020	-0.7195	0.3134	1.0108	0.1797	-1.2309	0.1168	0.3539	0.7654	-0.4363	0.5603	-1.1964	0.0385	-0.0997	0.9614	-1.4663	0.0152	-1.3454	0.0667	0.3038	0.7790	-2.0706	0.0003	0.7570	0.3861	0.3050	0.6275
AT1G65330	0.8009	0.3352	2.1353	0.0043	1.7206	0.0462	1.1039	0.3041	0.7714	0.5245	1.7557	0.0325	1.6419	0.0226	1.7456	0.0446	2.2958	0.0018	1.9815	0.0217	-0.3686	0.7926	1.0096	0.2182	1.8089	0.0292	2.2589	0.0013
AT1G65390	0.2184	0.7879	-2.0570	0.0005	-0.8047	0.3331	-1.0814	0.1997	-0.8062	0.3675	-0.8407	0.2386	-0.4091	0.5641	-0.8412	0.3752	-2.2364	0.0002	-0.6539	0.5407	0.3054	0.7863	-0.4621	0.5386	-1.9622	0.0018	-2.6470	0.0000
AT1G65480	-1.2195	0.1215	-2.2567	0.0027	-1.6218	0.0698	0.9086	0.4230	0.1266	0.9501	-2.1908	0.0074	-0.4688	0.5948	-0.4135	0.8282	-2.4483	0.0010	-1.7813	0.0521	-1.8407	0.0392	-1.4542	0.0631	-2.3075	0.0033	-1.8877	0.0081
AT1G65481	-1.6766	0.0195	-2.6298	0.0002	-1.6645	0.0463	-1.9936	0.0246	-0.4326	0.7618	-2.2527	0.0039	-2.3809	0.0004	-1.4610	0.1035	-2.7531	0.0001	-1.5236	0.0960	-1.8876	0.0246	-1.7644	0.0145	-2.1743	0.0042	-1.7713	0.0098
AT1G65483	-1.6158	0.0288	-2.5688	0.0003	-2.1239	0.0085	-1.8625	0.0442	0.2876	0.8593	-1.6064	NA	-1.5965	0.0241	-2.2962	0.0035	-2.9153	0.0001	-2.0467	0.0162	-1.5495	0.0926	-1.3707	0.0730	-2.1060	0.0075	-1.0988	0.1222
AT1G65486	-0.9411	0.1483	-2.6436	0.0000	-1.7030	0.0128	-0.9892	0.2574	-1.0255	0.2103	-1.9623	0.0033	-1.3498	0.0236	-1.9071	0.0040	-2.1940	0.0003	-1.0443	0.2269	0.6244	0.5045	-0.8747	0.1927	-1.5273	0.0267	-0.7817	0.1958
AT1G65490	-0.5420	0.4318	-1.7664	0.0031	-1.8827	0.0029	-0.7996	0.3633	-1.7638	0.0052	-1.0120	0.1388	-1.2532	0.0312	-2.6879	0.0000	-1.7420	0.0035	-1.2825	0.0897	0.8832	0.2860	-0.9957	0.1168	-2.5624	0.0000	-1.7254	0.0023
AT1G65510	1.1234	0.0895	0.3901	0.6619	0.8464	0.3367	0.5550	0.5932	0.8024	0.4077	1.2197	0.0931	0.5979	0.3940	0.2243	0.9041	0.5411	0.4726	0.5992	0.6212	-0.0081	1.0000	0.5813	0.4489	0.1689	0.9177	2.8379	0.0000
AT1G65610	-1.7255	0.0015	-1.7238	0.0021	-1.0714	0.1207	-1.2844	0.0753	-1.3101	0.0427	-1.1680	0.0625	-1.2496	0.0196	-2.0860	0.0003	-2.2879	0.0000	-1.8958	0.0021	-2.2460	0.0001	-2.5130	0.0000	-2.6224	0.0000	-1.4958	0.0053
AT1G65790	-0.8445	0.2722	-2.3301	0.0007	-0.2756	0.8184	-1.6274	0.0737	0.9313	0.3663	-1.1442	0.1571	-1.3040	0.0613	-1.0617	0.3064	-2.0202	0.0041	-1.1011	0.2811	-0.9200	0.3620	-1.4685	0.0424	-0.6161	0.6197	-0.7801	0.2614
AT1G65800	-1.3094	0.0361	-1.8288	0.0031	-0.5493	0.5502	-1.6606	0.0297	-0.0974	0.9542	-1.3718	0.0458	-1.4269	0.0166	-1.1188	0.1733	-2.2841	0.0002	-1.0127	0.2501	-1.0155	0.2233	-1.2290	0.0550	-0.8437	0.3406	-1.7634	0.0025
AT1G65870	-2.7442	0.0001	-2.7099	0.0002	-2.5260	0.0012	-1.8852	0.0469	-2.5497	0.0008	-2.9740	0.0002	-2.5025	0.0003	-2.5920	0.0009	-2.9205	0.0001	-1.3299	0.1999	-0.7174	0.5390	-2.1690	0.0033	-2.7747	0.0002	-2.0701	0.0036
AT1G66060	0.1836	0.8599	1.0093	0.2451	2.5389	0.0006	1.4688	0.1299	2.3677	0.0014	1.0882	0.2041	1.5011	0.0338	1.3598	0.1545	0.9139	0.2594	0.7853	0.5444	0.1454	0.9311	0.6715	0.4432	2.8070	0.0001	3.2081	0.0000
AT1G66090	-2.2967	0.0000	-2.8494	0.0000	-1.5943	0.0129	-1.2915	0.0884	-1.9819	0.0007	-1.7132	0.0067	-2.5842	0.0000	-2.5447	0.0000	-2.8894	0.0000	-1.8595	0.0037	0.3520	0.7315	-2.8831	0.0000	-2.5448	0.0000	-2.2577	0.0000
AT1G66270	-0.0055	0.9973	2.0908	0.0035	0.8256	0.4105	0.0963	0.9511	0.2140	0.8987	0.0000	1.0000	0.0529	0.9578	1.7009	0.0405	0.3323	0.7100	0.7850	0.5246	2.4497	0.0014	0.7192	0.3878	1.0542	0.2822	0.1588	0.8430
AT1G66280	1.2201	0.1005	2.5386	0.0002	1.1968	0.1953	0.4394	0.7239	2.1006	0.0050	0.6781	0.4488	0.9388	0.2056	2.3964	0.0011	1.7294	0.0176	2.7523	0.0002	2.8575	0.0001	0.7452	0.3709	1.7699	0.0253	0.7604	0.2908
AT1G66330	-1.4500	0.0000	-0.9342	0.0074	-1.1542	0.0012	-1.2081	0.0020	-0.8794	0.0208	-1.1133	0.0023	-1.4396	0.0000	-1.2820	0.0002	-1.3937	0.0000	-0.7098	0.1080	-0.7287	0.0877	-1.2857	0.0001	-0.9480	0.0097	-2.1019	0.0000
AT1G66400	-0.3285	0.6900	0.0053	0.9976	-0.3261	0.7584	0.3860	0.7281	0.1662	0.9160	-0.2311	0.7986	0.0932	0.9144	-0.2650	0.8810	-0.3762	0.6353	0.4096	0.7682	2.1376	0.0018	0.0102	0.9935	0.0034	1.0000	0.2914	0.6772
AT1G66465	-2.2898	0.0003	-2.3659	0.0003	-1.3022	0.1106	-1.7291	0.0369	-0.1222	0.9440	-1.2644	0.0928	-1.7506	0.0055	-1.7720	0.0167	-2.5768	0.0001	-1.6635	0.0364	-0.7221	0.4672	-2.4903	0.0001	-1.8856	0.0081	-0.5856	0.3816
AT1G66500	-0.6875	0.1972	-0.4787	0.4443	-0.5687	0.4196	0.5915	0.4281	-0.0972	0.9401	-0.2279	0.7350	-1.0101	0.0371	-0.3787	0.7262	-0.1439	0.8261	0.0631	0.9687	2.4456	0.0000	-0.7313	0.1756	-0.8676	0.1783	-0.4683	0.3516
AT1G66540	-2.3589	0.0001	-1.2999	0.0628	-2.0051	0.0035	-1.7063	0.0325	-1.7543	0.0121	-3.0665	0.0000	-1.9013	0.0017	-3.0104	0.0000	-3.4043	0.0000	-1.5418	0.0483	-1.7577	0.0176	-1.2633	0.0584	-1.4415	0.0515	-3.4379	0.0000
AT1G66600	-2.3691	0.0009	-2.4225	0.0009	-2.3411	0.0028	0.1631	0.9127	-1.6467	0.0579	-1.5381	0.0623	-1.3768	0.0579	-2.4707	0.0014	-2.2258	0.0025	-1.2275	0.2382	-1.3449	0.1646	-2.5464	0.0003	-2.6104	0.0005	-1.8225	0.0094
AT1G66700	-1.9433	0.0036	-1.7977	0.0104	-1.4577	0.0731	0.1034	0.9454	-1.0350	0.2700	-0.9883	0.2126	-1.7984	0.0054	-2.0842	0.0044	-1.9170	0.0052	-1.0994	0.2606	-1.2987	0.1415	-1.7180	0.0119	-1.6647	0.0291	-0.5523	0.4245
AT1G66725	-2.7412	0.0000	-2.1531	0.0005	-2.0825	0.0019	-1.8336	0.0183	-2.1386	0.0010	-2.4184	0.0004	-2.7346	0.0000	-2.6341	0.0000	-2.1467	0.0007	-0.4131	0.7631	-0.7078	0.4570	-2.4287	0.0001	-2.6282	0.0000	-2.0390	0.0007
AT1G66840	-1.9875	0.0009	-1.3029	0.0517	-1.2295	0.1058	-1.7270	0.0241	-1.3132	0.0793	-1.6900	0.0128	-1.6569	0.0050	-1.0699	0.2077	-1.7171	0.0060	-0.3449	0.8055	-0.3549	0.7499	-1.2656	0.0486	-1.3258	0.0681	-2.4860	0.0000
AT1G66880	-2.0307	0.0000	-2.1100	0.0000	-1.0002	0.0706	-1.5600	0.0038	-0.3496	0.6865	-1.0896	0.0339	-2.1769	0.0000	-1.6438	0.0006	-2.0564	0.0000	-1.2872	0.0163	-0.8796	0.1452	-2.2790	0.0000	-1.1072	0.0343	-1.2121	0.0059
AT1G66920	-1.4167	0.0183	-2.2302	0.0001	-0.7274	0.3838	-1.3126	0.0953	0.0108	0.9984	-1.2473	0.0635	-1.7245	0.0023	-1.1888	0.1192	-2.5052	0.0000	-0.7932	0.4038	-1.0738	0.1767	-1.4551	0.0162	-1.1687	0.1102	-1.3278	0.0201
AT1G66960	-2.2170	0.0026	-2.7642	0.0001	-1.9760	0.0188	-2.0380	0.0288	0.1057	0.9610	-1.9813	0.0162	-2.5389	0.0003	-2.3603	0.0032	-2.6640	0.0003	-1.6278	0.0870	-1.0259	0.3383	-2.1126	0.0044	-2.2213	0.0051	-0.9560	0.1946
AT1G67000	-1.4604	0.0377	-1.8633	0.0096	-1.4748	0.0776	-1.5669	0.0828	-0.7859	0.4651	-1.4690	0.0604	-1.5034	0.0246	-1.3077	0.1470	-2.2152	0.0016	-0.9355	0.3871	-0.9974	0.3033	-1.8813	0.0065	-1.1045	0.2331	-1.1459	0.0880
AT1G67030	0.9738	0.2205	0.2612	0.8131	-0.0507	0.9743	-0.1122	0.9458	-0.0134	0.9984	0.4169	0.6686	0.2587	0.7833	0.3249	0.8715	0.3338	0.7218	1.5436	0.1004	2.7829	0.0002	0.7376	0.3917	1.1925	0.2204	2.6687	0.0001
AT1G67105	1.5961	0.0213	0.0498	0.9673	0.0995	0.9447	0.4728	0.6922	1.3395	0.1269	0.2851	0.7743	0.3664	0.6648	1.5076	0.0730	0.0786	0.9392	1.1885	0.2260	2.7650	0.0001	1.1962	0.1043	0.9821	0.3317	2.2288	0.0006
AT1G67265	-0.6954	0.3343	0.3772	0.6694	0.8504	0.3330	0.3836	0.7307	0.6266	0.5624	1.0520	0.1550	-0.6904	0.3196	0.3126	0.8552	0.5556	0.4542	0.4758	0.7177	0.0187	0.9937	-0.5214	0.5076	1.3389	0.0780	2.2999	0.0001
AT1G67660	1.9477	0.0000	1.6085	0.0000	1.2914	0.0006	1.2416	0.0034	1.6045	0.0000	0.9947	0.0139	2.1603	0.0000	1.5328	0.0000	1.4950	0.0000	1.1228	0.0062	0.0462	0.9588	2.3998	0.0000	1.7945	0.0000	0.6526	0.0686
AT1G67810	-2.1793	0.0002	-2.2665	0.0002	-1.4817	0.0383	-0.4060	0.6994	-0.6190	0.5478	-1.2009	0.0864	-2.3005	0.0001	-1.5865	0.0239	-2.1880	0.0003	-1.2883	0.1076	-1.0067	0.2313	-2.0835	0.0005	-1.5470	0.0247	-0.5469	0.3823
AT1G67865	-2.0681	0.0000	-1.6682	0.0003	-2.2718	0.0000	-0.9578	0.1297	-0.1767	0.8702	-0.5535	0.3285	-1.9233	0.0000	-0.6114	0.4265	-0.8544	0.0871	-0.2122	0.8518	-1.6627	0.0010	-1.4035	0.0025	-0.6682	0.3197	-1.5433	0.0005
AT1G67980	-1.6443	0.0104	-1.1000	0.1317	-0.8347	0.3448	-0.7744	0.4284	-0.8870	0.3417	0.7765	0.3075	-2.4343	0.0000	-0.5130	0.7101	-0.0999	0.9095	0.0990	0.9624	-2.3481	0.0007	-1.8701	0.0033	-0.7954	0.4157	1.0488	0.0884
AT1G68050	0.9139	0.2653	1.9306	0.0126	1.7536	0.0433	1.6469	0.0935	2.1233	0.0081	1.7578	0.0339	1.6565	0.0228	2.1836	0.0073	2.6173	0.0004	2.7458	0.0005	0.0195	0.9949	2.0843	0.0049	2.6268	0.0005	2.5805	0.0002
AT1G68238	-2.6663	0.0000	-0.6208	0.4755	-1.7115	0.0296	0.2706	0.8311	-1.1217	0.2231	-1.8609	0.0142	-2.6107	0.0000	-0.6678	0.6077	-1.5940	0.0248	-0.9386	0.3775	0.4379	0.7124	-2.5640	0.0001	-2.1245	0.0035	-2.7228	0.0000
AT1G68250	-0.2990	0.7549	3.5635	0.0000	0.9344	0.3504	-0.1739	0.9068	-0.2472	0.8827	0.0457	0.9697	-0.1930	0.8413	-0.0317	0.9924	-0.1670	0.8642	-0.2036	0.9214	4.1057	0.0000	0.4741	0.6106	-0.2207	0.8999	-0.0993	0.9052
AT1G68440	-1.2568	0.0169	-1.1391	0.0434	-1.1998	0.0513	-0.9807	0.1736	-2.6880	0.0000	-0.7880	0.1974	-2.2613	0.0000	-1.2688	0.0376	-0.6331	0.2760	-0.6503	0.4499	-0.3847	0.6650	-1.6245	0.0015	-1.9709	0.0002	-0.8900	0.0781
AT1G68500	0.3140	0.6305	1.1120	0.0466	0.8561	0.2066	0.6930	0.3697	0.7857	0.2796	1.1234	0.0541	0.4295	0.4629	0.9774	0.1507	2.0076	0.0001	1.6727	0.0033	-0.5821	0.4747	1.0794	0.0438	0.9770	0.1320	1.0895	0.0287
AT1G68590	-0.0228	0.9697	-0.4233	0.3859	-0.5128	0.3422	-0.5029	0.3948	-0.1415	0.8759	-0.6335	0.1630	0.0525	0.9227	0.0119	0.9945	-1.0663	0.0073	-0.8480	0.0894	-0.5650	0.3059	-0.2646	0.5969	-0.7634	0.1126	-2.0372	0.0000
AT1G68620	-2.8916	0.0000	-1.1874	0.0252	-0.8182	0.2106	-0.5041	0.5222	-1.6846	0.0011	-0.5759	0.3396	-3.2061	0.0000	-1.6354	0.0020	-0.5965	0.2841	-0.5092	0.5715	-0.6992	0.3257	-2.0702	0.0000	-0.9180	0.1456	0.3895	0.4479
AT1G68850	1.6109	0.0172	0.3729	0.7022	0.3216	0.7820	0.2746	0.8316	0.8299	0.4309	1.1262	0.1560	0.8817	0.2134	1.2876	0.1459	0.1460	0.8789	2.4962	0.0004	1.6000	0.0516	1.5658	0.0226	0.7596	0.4957	0.9973	0.1389
AT1G69260	-2.1907	0.0012	1.5882	0.0312	0.7034	0.4872	0.8542	0.4172	-1.0835	0.2574	0.9011	0.2726	-2.0798	0.0015	-0.5560	0.7101	1.4625	0.0428	0.7114	0.5683	-0.7903	0.4472	-0.7882	0.3231	0.3050	0.8438	0.1938	0.8031
AT1G69490	-4.9068	0.0000	-1.4799	0.0004	-0.7960	0.1387	-1.2904	0.0123	-0.3194	0.6914	-0.2261	0.6918	-4.2826	0.0000	-1.1559	0.0153	-1.5408	0.0002	-1.4126	0.0027	-1.9059	0.0000	-4.3291	0.0000	-0.1470	0.8831	-0.9568	0.0190
AT1G69520	-0.8661	0.2347	-2.2849	0.0005	-1.0829	0.2160	-1.2399	0.1791	0.0302	0.9911	-1.8571	0.0119	-0.8301	0.2400	-0.6775	0.5907	-2.3414	0.0004	-0.4910	0.7210	-0.2870	0.8271	-0.9537	0.1946	-1.7963	0.0142	-1.9794	0.0020
AT1G69523	-0.0279	0.9711	-0.7962	0.1700	-0.6171	0.3771	-0.4620	0.5636	-0.0881	0.9480	-1.6445	0.0030	0.2014	0.7466	-0.5530	0.5352	-2.0889	0.0000	-0.7378	0.3316	0.3841	0.6433	0.2870	0.6582	-0.1057	0.9332	-2.8314	0.0000
AT1G69526	-0.9935	0.0734	-0.5555	0.4067	-0.7067	0.3380	-0.8504	0.2651	-0.9214	0.1972	-1.6074	0.0081	-0.6049	0.2850	-0.9356	0.2031	-1.7441	0.0016	-0.7336	0.3812	0.1620	0.8853	-0.7468	0.2032	-0.5177	0.5720	-2.7117	0.0000
AT1G69570	-2.1014	0.0000	-0.3966	0.4733	-0.0031	0.9977	-0.6494	0.2994	-0.4686	0.5038	-0.2929	0.6023	-2.2157	0.0000	-0.5269	0.4699	-0.3746	0.4585	-0.6096	0.3668	0.1600	0.8518	-2.0147	0.0000	-1.4001	0.0043	0.0525	0.9170
AT1G69720	-1.3573	0.0757	-2.4178	0.0009	-1.1523	0.2340	-1.6594	0.0857	0.0216	0.9946	-1.7337	0.0342	-1.3070	0.0770	-1.2217	0.2386	-2.3535	0.0013	-1.3160	0.1974	-1.2655	0.2012	-1.2584	0.1091	-1.3402	0.1495	-1.8786	0.0075
AT1G69730	-1.4490	0.0420	-2.2516	0.0011	-1.2736	0.1496	-1.0747	0.2850	-0.7788	0.4822	-1.9608	0.0104	-1.5575	0.0220	-1.2230	0.2000	-2.3877	0.0005	-0.2730	0.8810	0.0171	0.9958	-1.4905	0.0387	-1.6380	0.0394	-1.9778	0.0029
AT1G69880	1.9338	0.0084	0.5581	0.5713	-0.6224	0.5812	1.1870	0.2615	1.8540	0.0264	0.2676	0.7982	1.7857	0.0122	0.7601	0.5838	1.0088	0.2134	1.0768	0.3414	0.0341	0.9867	2.4693	0.0005	-0.3154	0.8505	0.6469	0.3926
AT1G69930	-1.6908	0.0138	-1.5824	0.0307	-1.2634	0.1452	0.0427	0.9790	-1.6831	0.0331	-0.6764	0.4232	-1.7550	0.0074	-1.8034	0.0203	-0.5396	0.4991	-0.8398	0.4550	-1.4176	0.1054	-1.5698	0.0250	-2.1911	0.0026	-0.5478	0.4346
AT1G70030	0.1217	0.9099	0.8452	0.3588	0.5676	0.6203	0.8973	0.4307	0.6529	0.6235	1.1330	NA	0.8654	0.2757	1.0058	0.3961	0.3095	0.7472	0.7234	0.6006	0.7264	0.5327	0.9405	0.2613	0.5377	0.7082	2.3209	0.0010
AT1G70140	-1.5607	0.0089	-1.8497	0.0021	-0.6452	0.4559	-1.7860	0.0142	-0.8110	0.3557	-1.7023	0.0100	-1.8814	0.0008	-1.6114	0.0163	-2.2319	0.0002	-1.0725	0.1958	-1.1057	0.1621	-2.0263	0.0005	-1.8911	0.0026	-2.3291	0.0000
AT1G70170	-1.9453	0.0082	-1.4526	0.0737	-1.4950	0.0987	-1.0870	0.3070	-1.9017	0.0218	-0.5641	NA	-2.4800	0.0003	-2.0015	0.0153	-1.4259	0.0667	-1.2241	0.2450	-1.1285	0.2685	-1.4601	0.0574	-1.4973	0.0935	0.0254	0.9773
AT1G70220	1.3378	0.0804	0.3059	0.7799	0.5378	0.6400	0.6219	0.6070	1.5662	0.0795	0.9928	NA	1.3268	0.0691	1.3953	0.1471	0.4076	NA	1.1706	0.2789	0.1839	0.9119	1.4124	0.0669	2.3312	0.0022	1.2311	NA
AT1G70250	-1.5117	0.0042	-2.0489	0.0001	-1.4949	0.0118	-1.6100	0.0130	-1.0492	0.1178	-1.9214	0.0008	-1.8497	0.0002	-1.4161	0.0188	-2.4718	0.0000	-1.4205	0.0241	-0.4725	0.5805	-1.5656	0.0030	-1.7561	0.0015	-2.3626	0.0000
AT1G70580	-0.4274	0.4200	-0.9574	0.0547	-0.7336	0.2212	-0.6116	0.3697	-0.4480	0.5633	-1.5077	0.0023	-0.1076	0.8601	-1.2379	0.0157	-1.5935	0.0004	-0.7748	0.2335	-0.3478	0.6512	-0.1831	0.7735	-0.4754	0.5321	-2.1516	0.0000
AT1G70610	-0.8995	0.0014	-0.3445	0.3268	-0.2501	0.5626	-0.6966	0.0597	-0.6246	0.0708	-0.8753	0.0054	-0.4388	0.1360	-1.2520	0.0000	-0.9883	0.0005	-0.7689	0.0227	-0.5213	0.1726	-0.9377	0.0008	-0.7178	0.0258	-2.2655	0.0000
AT1G70640	-0.2990	0.7650	0.4783	0.6399	0.6297	0.5770	0.4382	0.7377	1.0865	0.3056	0.4441	0.6509	-0.6839	0.4125	0.6599	0.6648	0.8634	0.3016	0.4449	0.7897	-0.1406	0.9348	0.9458	0.2592	2.5961	0.0006	1.9077	0.0072
AT1G70820	0.7852	0.1186	0.5280	0.3724	0.7620	0.2229	0.3654	0.6444	0.7663	0.2383	-0.0444	0.9538	0.5574	0.2747	0.4075	0.6794	0.1214	0.8510	0.1991	0.8702	-2.2877	0.0000	1.0553	0.0314	1.6982	0.0004	-0.3342	0.5016
AT1G70850	1.5348	0.0235	1.2709	0.0878	1.0786	0.2193	0.9813	0.3155	0.3362	0.8165	1.1243	0.1471	-0.0029	0.9987	2.0648	0.0042	1.7622	0.0098	2.7069	0.0001	2.0976	0.0048	0.9795	0.1831	2.2675	0.0010	3.0984	0.0000
AT1G70920	-1.4963	0.0032	-0.2997	0.6571	-0.9556	0.1395	-0.2876	0.7311	-0.8619	0.2036	-0.5614	0.3613	-2.3430	0.0000	-0.9819	0.1272	-0.2976	0.6232	-0.5712	0.4990	-0.6153	0.4050	-0.9016	0.0857	-0.8812	0.1915	-1.5832	0.0029
AT1G70985	-0.5582	0.4880	-1.7234	0.0169	-1.5074	0.0666	-0.7476	0.4812	-0.5952	0.6252	-1.8729	0.0141	-0.4329	0.5892	-1.1237	0.2464	-2.4075	0.0005	-0.9554	0.3673	0.8365	0.3996	-0.3691	0.6834	-0.8819	0.3900	-1.0316	0.1232
AT1G71030	-1.4291	0.0001	-0.1974	0.7248	0.1016	0.8879	-0.3211	0.6159	-0.7261	0.1489	0.2663	0.5966	-1.8073	0.0000	-0.4498	0.5139	-0.0799	0.8818	-0.5436	0.3719	-2.7026	0.0000	-1.3573	0.0003	0.3398	0.6286	0.3127	0.4361
AT1G71040	-1.5309	0.0000	-1.7643	0.0000	-0.9711	0.0018	-1.3392	0.0000	-0.6828	0.0484	-1.2668	0.0000	-2.2082	0.0000	-1.2168	0.0000	-1.5976	0.0000	-1.1538	0.0002	-0.7284	0.0385	-1.7271	0.0000	-1.0159	0.0008	-0.9529	0.0007
AT1G71390	-2.2906	0.0017	-1.9879	0.0099	-1.3540	0.1519	-1.6514	0.0917	-0.6064	0.6557	-2.1569	NA	-1.8746	0.0088	-2.3730	0.0029	-2.2631	0.0024	-2.3110	0.0058	-1.2393	0.2208	-2.3085	0.0016	-1.9667	0.0161	-1.6977	0.0171
AT1G71400	0.1319	0.8292	-2.2864	0.0000	-1.0344	0.0531	-1.3771	0.0121	-1.3755	0.0045	-1.2496	0.0135	-0.8836	0.0446	-0.6150	0.3839	-1.9381	0.0000	-0.4675	0.5588	0.2307	0.7787	0.0241	0.9755	-1.0967	0.0342	-1.2641	0.0042
AT1G71520	-2.0225	0.0041	-1.6793	0.0266	-0.9947	0.3029	0.6001	0.6017	-1.9368	0.0136	-1.4169	0.0798	-1.9701	0.0037	-1.5706	0.0693	-1.1336	0.1382	-1.2320	0.2183	-1.6840	0.0523	-2.9807	0.0000	-2.9090	0.0000	-1.9689	0.0041
AT1G71890	-1.0011	0.0571	-1.1470	0.0410	-0.9168	0.1673	-0.7056	0.3444	-0.3483	0.7228	-1.6636	0.0054	-1.0087	0.0416	-1.7137	0.0023	-1.3810	0.0112	-1.8560	0.0018	-0.6448	0.3902	-1.0981	0.0382	-1.3449	0.0244	-2.5459	0.0000
AT1G71920	-1.0878	0.1618	1.1986	0.1452	1.5017	0.0856	-1.0181	0.3417	2.4964	0.0006	-0.6250	0.4949	1.0630	0.1520	-0.4952	0.7687	-1.0890	0.1671	-0.8427	0.4955	-0.9804	0.3500	-0.7618	0.3683	0.1526	0.9336	-0.4121	0.5952
AT1G72000	-1.7587	0.0173	-2.3370	0.0015	-1.4758	0.1033	-1.4321	0.1500	0.1539	0.9343	-2.0330	NA	-1.6468	0.0208	-1.2318	0.2302	-2.4313	0.0009	-2.0556	0.0164	-1.1891	0.2365	-1.6510	0.0285	-1.1839	0.2281	-0.3860	0.6226
AT1G72070	-1.5806	0.0335	-1.7879	0.0207	-1.4643	0.1045	-0.2748	0.8412	-1.8888	0.0219	1.8813	0.0172	-2.2785	0.0009	-0.8241	0.5214	0.3382	0.7114	-0.0296	0.9893	-1.2915	0.1876	-1.4245	0.0624	-0.2323	0.8935	1.0155	0.1490
AT1G72100	0.1659	0.8513	2.4545	0.0001	0.3194	0.7555	0.2709	0.8188	0.0000	1.0000	0.1441	NA	-0.0112	0.9900	0.0076	0.9972	0.2278	0.7787	0.0000	1.0000	1.7436	0.0176	0.2884	0.7374	0.2628	0.8490	0.0000	1.0000
AT1G72240	-2.0076	0.0059	-1.5237	0.0571	-1.5459	0.0824	-1.2585	0.2227	-0.9178	0.4077	-1.7380	0.0339	-1.9684	0.0050	-2.0670	0.0112	-2.2557	0.0023	-1.4562	0.1343	0.3142	0.8268	-1.7411	0.0199	-1.7456	0.0373	-1.0361	0.1492
AT1G72416	3.1050	0.0000	-0.8599	0.3365	0.0850	0.9539	0.3477	0.7943	0.9247	0.4004	-0.0947	0.9334	2.4358	0.0003	0.9229	0.4429	-0.6107	0.4734	0.9047	0.4503	1.3007	0.1815	2.8826	0.0000	-0.4015	0.7954	0.3007	0.7075
AT1G72430	-0.3267	0.4601	-1.3564	0.0002	-1.3471	0.0005	-1.4238	0.0008	-1.6162	0.0000	-2.0366	0.0000	-0.8722	0.0171	-1.9490	0.0000	-1.5146	0.0000	-1.5212	0.0001	0.2617	0.6886	-0.7260	0.0662	-2.1945	0.0000	-1.3474	0.0002
AT1G72510	-0.4081	0.5286	-2.3543	0.0000	-2.0844	0.0001	-0.4937	0.5698	-1.6776	0.0031	-0.7819	0.2170	-1.4085	0.0065	-0.6622	0.4676	-0.7761	0.1858	0.4470	0.6704	0.9978	0.1607	-0.1078	0.8961	-0.3752	0.7198	0.3482	0.5392
AT1G72520	-0.6597	0.3428	-1.5472	0.0166	-0.7693	0.3703	-0.1970	0.8719	-1.0845	0.1825	-1.3874	0.0449	-0.9330	0.1417	-2.1936	0.0006	-2.4229	0.0001	-1.2974	0.1044	1.1598	0.1521	-1.7923	0.0033	-2.0087	0.0017	-1.6647	0.0048
AT1G72770	-2.3139	0.0000	0.2376	0.7285	-0.3758	0.6044	-0.3914	0.6163	-1.6803	0.0005	-0.0664	0.9287	-2.3941	0.0000	-0.8450	0.1944	0.5785	0.2751	0.3214	0.7529	-0.5386	0.4530	-1.8447	0.0000	-1.0659	0.0544	-0.7014	0.1318
AT1G72890	-1.1809	0.0180	-2.8204	0.0000	-2.1054	0.0000	-1.1081	0.0896	-1.5265	0.0037	-1.6065	0.0030	-2.3566	0.0000	-0.9661	0.1321	-1.9914	0.0001	-0.8025	0.2651	-0.4203	0.6008	-0.7376	0.1731	-0.9699	0.1127	-1.6614	0.0005
AT1G72900	-1.6059	0.0052	-2.8699	0.0000	-2.6747	0.0000	-0.3058	0.7685	-2.4235	0.0000	-1.3380	0.0384	-3.1623	0.0000	-1.4670	0.0271	-1.2926	0.0318	-1.1398	0.1387	-0.5876	0.5110	-1.6504	0.0041	-1.7948	0.0032	-1.8204	0.0010
AT1G72910	-1.7182	0.0222	-2.4475	0.0009	-1.7330	0.0464	-1.3863	0.1774	-0.8670	0.4553	-2.1945	0.0070	-1.9381	0.0067	-1.5904	0.0833	-2.4278	0.0010	-0.7093	0.6111	0.8287	0.4602	-1.5625	0.0431	-1.6884	0.0493	-1.6237	0.0225
AT1G72920	-1.3411	0.0508	-2.0279	0.0027	-1.4310	0.0758	-0.7377	0.4777	-0.7861	0.4485	-1.5551	0.0374	-1.1524	0.0870	-1.4673	0.0718	-2.0192	0.0027	-0.6538	0.5956	1.4547	0.0797	-0.9984	0.1713	-1.5557	0.0430	-1.4296	0.0258
AT1G72930	-1.0852	0.1528	-2.6376	0.0001	-1.7818	0.0283	-1.2216	0.2223	-0.7585	0.5108	-1.9144	0.0143	-1.5803	0.0228	-1.5953	0.0624	-2.5743	0.0002	-0.8805	0.4519	-0.1159	0.9441	-1.2792	0.0895	-1.5304	0.0672	-1.8678	0.0059
AT1G72950	-1.2114	0.0565	-1.3434	0.0462	-0.5432	0.5550	-1.1867	0.1590	-0.0426	0.9836	-1.2755	0.0724	-0.7273	0.2593	-1.6763	0.0169	-2.3122	0.0003	-1.2142	0.1420	0.4392	0.6708	-1.5139	0.0162	-1.3011	0.0838	-1.9308	0.0017
AT1G73110	-0.5994	0.1658	-1.2054	0.0031	-0.9399	0.0468	-0.8226	0.1288	-0.7173	0.1757	-0.9746	0.0302	-0.7631	0.0577	-0.5311	0.4172	-1.2924	0.0014	-0.8305	0.1156	-0.4027	0.5195	-0.6336	0.1473	-0.7697	0.1248	-2.2959	0.0000
AT1G73120	-0.7198	0.3416	3.1038	0.0000	2.7713	0.0000	0.2880	0.8176	2.6332	0.0000	-0.4699	0.5887	0.2569	0.7588	1.2174	0.1633	-0.4428	0.5876	-0.2491	0.8888	0.7924	0.4183	-0.3169	0.7268	0.9516	0.3082	-0.1801	0.8128
AT1G73330	1.0111	0.0571	-1.5947	0.0023	-1.3798	0.0187	-0.0493	0.9665	0.6723	0.3842	0.0420	0.9603	-0.6800	0.2110	1.2849	0.0307	0.4252	0.4820	2.4823	0.0000	2.2749	0.0000	0.8213	0.1411	1.4794	0.0073	2.3916	0.0000
AT1G73480	-1.4624	0.0024	0.4256	0.5021	-0.1913	0.8216	0.3242	0.6991	-1.6985	0.0007	0.6965	0.2275	-2.1664	0.0000	-0.0868	0.9599	0.8458	0.1064	0.0974	0.9503	-1.3802	0.0145	-1.1915	0.0169	0.9545	0.1138	0.7747	0.1037
AT1G73490	-0.6787	0.0649	-6.4497	0.0000	-6.5852	0.0000	-0.4444	0.4095	-6.3253	0.0000	-0.4260	0.3181	-6.6288	0.0000	-0.0232	0.9885	-0.5221	0.1759	-0.3861	0.5377	-0.0270	0.9775	-0.4667	0.2403	-0.5547	0.2517	-0.9410	0.0058
AT1G73500	-0.2026	0.7754	-2.1265	0.0000	-1.6127	0.0048	0.0312	0.9808	-1.5028	0.0086	-0.2090	0.7753	-1.2955	0.0113	-0.3871	0.7460	-0.3709	0.5581	-0.4967	0.6189	0.7759	0.2955	-0.4739	0.4580	-0.3995	0.6906	1.1978	0.0175
AT1G73510	0.0463	0.9647	-0.3349	0.7267	-0.2960	0.7924	0.5058	0.6556	-0.4191	0.7528	-0.1560	0.8749	-0.4945	0.5277	-0.1898	0.9250	-0.0722	0.9400	0.0459	0.9826	3.3520	0.0000	-0.2979	0.7485	-0.2621	0.8639	0.2634	0.7207
AT1G73540	-0.6382	0.3159	-1.2464	0.0423	-1.6757	0.0072	0.0441	0.9734	-1.1990	0.0840	-0.9192	0.1644	-1.2399	0.0260	-0.9961	0.2029	-1.3023	0.0276	-0.8901	0.2879	2.0259	0.0008	-0.6661	0.3062	-0.8802	0.2574	-0.5515	0.3365
AT1G73670	-0.7465	0.0046	-1.3340	0.0000	-1.0878	0.0000	-0.7477	0.0239	-0.9259	0.0007	-0.6471	0.0298	-2.0667	0.0000	-0.8283	0.0039	-0.5798	0.0372	-0.4215	0.2788	-0.4424	0.2230	-1.0174	0.0001	-0.4972	0.1370	-1.0808	0.0000
AT1G73805	-1.8941	0.0062	-2.9616	0.0000	-1.2819	0.1462	-1.8448	0.0338	-0.5498	0.6706	-1.7944	0.0197	-1.8833	0.0047	-1.9525	0.0114	-2.6205	0.0001	-1.4424	0.1120	-0.2334	0.8721	-2.1861	0.0013	-1.8726	0.0136	-1.5328	0.0213
AT1G73810	-1.3574	0.0257	-2.0163	0.0008	-0.8925	0.2662	-0.3230	0.7625	0.1198	0.9384	-0.8773	0.2136	-1.6455	0.0040	-1.4776	0.0346	-1.4462	0.0202	-1.3662	0.0699	-1.5618	0.0284	-1.4460	0.0180	-0.7300	0.4296	0.1556	0.8170
AT1G73860	0.2632	0.7768	-0.4541	0.6368	0.9078	0.3381	-0.7246	0.5123	0.9341	0.3550	-1.1975	NA	0.5356	0.4937	-1.0965	0.2946	-2.0561	0.0051	-0.5825	0.6733	-0.7787	0.4691	-0.7571	0.3549	-1.3389	0.1393	-1.5206	0.0286
AT1G73870	-1.8631	0.0016	-1.4107	0.0281	-1.4284	0.0423	-1.6774	0.0253	-1.8721	0.0032	-1.8941	0.0038	-2.0116	0.0004	-1.6989	0.0112	-2.5495	0.0000	-0.8261	0.3844	-0.4389	0.6709	-1.9088	0.0012	-1.2117	0.0985	-2.5428	0.0000
AT1G73965	-1.7659	0.0121	-1.6919	0.0240	-1.1370	0.2134	-0.0998	0.9472	-1.7864	0.0267	-1.6351	NA	-1.8903	0.0044	-1.1536	0.2350	-1.1795	0.1162	-1.1124	0.2727	-0.3851	0.7559	-2.3340	0.0007	-0.7251	0.5289	-0.2007	0.7940
AT1G74010	-0.9328	0.0900	-1.8126	0.0007	-1.3233	0.0320	-0.3659	0.6781	-1.0437	0.1183	-1.2966	0.0311	-2.1420	0.0000	-1.0869	0.1038	-0.7845	0.1719	0.0828	0.9611	-0.9330	0.1908	-1.0899	0.0471	-1.7956	0.0016	-0.3398	0.5419
AT1G74070	-0.0441	0.9496	-0.6589	0.2255	-0.9647	0.0833	-0.3721	0.6208	-0.8823	0.1294	-0.9148	0.0791	0.0325	0.9600	-0.5361	0.5013	-1.0792	0.0233	-0.6533	0.3567	-0.0325	0.9788	0.1928	0.7621	-0.3878	0.6419	-2.2168	0.0000
AT1G74080	-2.6747	0.0002	-2.0759	0.0063	-1.7431	0.0441	-0.3744	0.7781	-1.7752	0.0388	-0.9603	NA	-2.3836	0.0006	-2.2992	0.0040	-1.3179	0.0946	-2.0607	0.0169	-1.8252	0.0401	-2.2720	0.0018	-1.6270	0.0608	-0.9057	0.2167
AT1G74100	-1.0540	0.1077	-2.0502	0.0009	-0.8207	0.3394	-0.7471	0.4343	0.0961	0.9563	-1.8099	0.0081	-1.2390	0.0449	-0.6314	0.5918	-1.9774	0.0015	-0.9946	0.2740	0.0089	0.9997	-0.9844	0.1449	-1.2418	0.1015	-1.1185	0.0624
AT1G74110	-0.4352	0.6414	1.2327	0.1480	2.1892	0.0067	0.1567	0.9194	0.9148	0.4217	0.3658	NA	-0.7770	0.3392	0.6124	0.7000	0.5874	0.5020	0.4684	0.7733	-0.2189	0.8911	-0.6102	0.5025	1.9739	0.0149	0.8677	0.2404
AT1G74130	0.3194	0.7446	3.6212	0.0000	3.7169	0.0000	-0.0759	0.9645	4.1796	0.0000	0.0000	1.0000	3.8808	0.0000	0.7754	0.5672	0.1830	0.8570	0.0000	1.0000	0.0000	1.0000	0.1142	0.9226	0.4389	0.7761	0.3185	0.6967
AT1G74210	-0.4489	0.5465	-2.3070	0.0001	-1.2027	0.1170	-0.6756	0.4812	-0.0538	0.9790	-1.3289	0.0557	-0.9919	0.1143	-0.3464	0.8262	-1.5619	0.0136	-0.3349	0.8129	-0.1324	0.9227	-0.4576	0.5526	-0.5021	0.6583	-1.4070	0.0173
AT1G74360	-1.8902	0.0001	-1.9794	0.0000	-1.2040	0.0349	-0.5235	0.4970	-1.5120	0.0038	-1.1319	0.0408	-2.2946	0.0000	-1.9613	0.0001	-1.3599	0.0068	-1.1432	0.0632	-1.1840	0.0480	-2.1003	0.0000	-1.9891	0.0001	-1.4562	0.0021
AT1G74450	-0.0772	0.9033	-0.1909	0.7683	-0.3316	0.6271	-0.1374	0.8760	-0.4406	0.5547	-0.1026	0.8766	-0.5717	0.2185	-0.3314	0.7355	-0.2164	0.6974	0.0920	0.9474	2.4612	0.0000	-0.1998	0.7420	-0.6754	0.2683	-0.3610	0.4284
AT1G74710	-1.7112	0.0027	-1.6291	0.0060	-1.0327	0.1632	-1.0117	0.2138	-0.6174	0.5114	-1.6751	0.0085	-1.5507	0.0052	-1.6482	0.0099	-2.4804	0.0000	-1.4979	0.0299	-1.4120	0.0424	-1.7722	0.0018	-1.5594	0.0135	-2.1092	0.0001
AT1G74930	0.4686	0.4999	0.0872	0.9266	0.1296	0.9069	1.7291	0.0145	0.1383	0.9265	0.8911	0.1920	0.6057	0.3412	0.2294	0.8896	0.2407	0.7516	0.2889	0.8350	3.0220	0.0000	0.9021	0.1544	0.8132	0.3355	1.4029	0.0119
AT1G75000	-1.1595	0.0219	-1.8849	0.0001	-1.9168	0.0002	-0.8315	0.2412	-2.2431	0.0000	-1.8419	0.0009	-1.7320	0.0002	-1.6321	0.0024	-1.7281	0.0007	-1.2872	0.0332	0.0744	0.9485	-1.4317	0.0041	-1.8309	0.0005	-1.1697	0.0164
AT1G75100	-1.6974	0.0000	-1.0856	0.0029	-0.9944	0.0134	-1.0519	0.0177	-1.5661	0.0000	-0.8092	0.0458	-1.8347	0.0000	-1.4220	0.0001	-1.3605	0.0001	-1.1980	0.0027	-0.3691	0.5074	-1.6941	0.0000	-1.4545	0.0000	-2.0520	0.0000
AT1G75250	0.1931	0.7848	-2.0612	0.0001	-1.1130	0.0906	-0.9355	0.2113	-0.6854	0.3929	-1.1540	0.0590	-0.2923	0.6447	-1.0048	0.1546	-1.7959	0.0011	-1.1875	0.0815	0.3865	0.6661	0.6420	0.2793	0.2391	0.8394	-0.3062	0.5913
AT1G75260	-0.8406	0.1189	-0.7687	0.2018	-1.1409	0.0652	-1.3266	0.0421	-0.3979	0.6759	-1.2999	0.0255	-1.1044	0.0264	-0.4915	0.6186	-0.9225	0.0929	-0.8591	0.2414	-0.8021	0.2590	-0.9490	0.0786	-0.5767	0.4835	-2.1264	0.0000
AT1G75450	-3.3589	0.0000	-0.8452	0.1566	-0.0575	0.9564	-1.6192	0.0096	-0.2294	0.8427	-1.0798	0.0650	-2.8121	0.0000	-0.5292	0.5869	-2.2922	0.0000	-1.5595	0.0079	0.2040	0.8431	-3.6075	0.0000	0.0793	0.9523	-1.3134	0.0082
AT1G75490	-2.6805	0.0000	-0.2221	0.8009	-0.2202	0.8290	0.3360	0.7466	-2.0713	0.0005	0.4850	0.5134	-3.8235	0.0000	-0.5954	0.5929	0.3294	0.6516	-0.5017	0.6612	-1.8614	0.0045	-2.6568	0.0000	0.0260	0.9905	-0.0187	0.9792
AT1G75590	-2.8117	0.0000	-0.1769	0.8270	-0.5174	0.5149	-0.5108	0.5522	-0.7142	0.3720	-0.0788	0.9240	-2.6103	0.0000	-0.5020	0.6361	-0.3108	0.6389	-0.5162	0.6075	-1.7912	0.0031	-2.8707	0.0000	-0.4428	0.6553	0.0544	0.9316
AT1G76240	-1.2830	0.0266	-1.7269	0.0027	-0.8874	0.2390	-1.4826	0.0397	-1.6048	0.0096	-2.5340	0.0000	-2.0158	0.0001	-1.5581	0.0146	-2.1734	0.0001	-1.0734	0.1658	0.2926	0.7819	-0.7848	0.2140	-1.0367	0.1521	-1.0800	0.0491
AT1G76520	0.2215	0.7326	-2.1025	0.0000	-0.7239	0.2781	-0.3386	0.6879	-0.2825	0.7837	-1.0809	0.0523	-0.2417	0.6924	-0.2642	0.8370	-1.7032	0.0005	-0.5770	0.4938	0.3463	0.6865	0.2778	0.6710	-0.8631	0.1775	-0.5973	0.2240
AT1G76590	-0.0569	0.9436	0.4988	0.4637	0.6966	0.3439	1.1955	0.0881	0.7521	0.3294	2.0622	0.0003	-0.7838	0.1562	1.2190	0.0562	1.7904	0.0007	1.7111	0.0039	-1.2806	0.0499	0.4666	0.4701	1.5750	0.0054	2.7135	0.0000
AT1G76610	-0.3215	0.6682	-0.4774	0.5446	-0.0424	0.9732	0.7428	0.3916	-0.6470	0.5104	0.6421	0.3651	-2.0936	0.0003	0.7667	0.4077	0.9792	0.1128	0.3713	0.7704	0.4192	0.6762	-0.0219	0.9838	1.3097	0.0483	1.9324	0.0003
AT1G76680	-0.2443	0.7681	-1.9971	0.0011	-0.6684	0.4559	0.2665	0.8192	-0.6645	0.5071	-0.9088	0.2105	-0.9226	0.1487	-0.7202	0.5043	-1.7902	0.0041	-0.3100	0.8308	0.2691	0.8237	-0.5633	0.4495	-1.4253	0.0452	-2.1052	0.0003
AT1G76790	3.2698	0.0000	1.5162	0.0122	0.9782	0.2013	2.3662	0.0002	1.6582	0.0082	1.3360	0.0416	2.2703	0.0000	2.0490	0.0007	2.0292	0.0004	2.4375	0.0000	2.3887	0.0001	3.3885	0.0000	2.9790	0.0000	2.1909	0.0001
AT1G76970	-0.8336	0.0267	-2.0539	0.0000	-1.5165	0.0000	-0.6443	0.2163	-0.9066	0.0343	-0.7870	0.0607	-1.8884	0.0000	-1.2166	0.0020	-1.3192	0.0004	-0.9082	0.0448	-0.7859	0.0942	-1.2281	0.0007	-1.4402	0.0001	-0.7326	0.0408
AT1G77090	0.2392	0.6663	-0.7722	0.1187	-0.6856	0.2343	-0.3778	0.5952	-0.1341	0.8975	-1.2882	0.0074	0.5024	0.2828	-0.5296	0.4771	-1.5261	0.0004	-0.6488	0.3277	0.0698	0.9456	0.4089	0.4333	-0.3697	0.6418	-2.0537	0.0000
AT1G77145	-1.2139	0.0486	-1.3790	0.0337	-1.1366	0.1357	-0.4298	0.6634	-0.9600	0.2426	0.4749	0.5231	-2.0425	0.0003	0.0524	0.9807	0.0127	0.9899	0.6726	0.4996	-0.4937	0.6130	-1.1807	0.0595	0.4967	0.6418	0.8620	0.1318
AT1G77530	1.9387	0.0082	0.4252	0.6747	0.4489	0.7005	0.2871	0.8355	0.6073	0.6496	0.3741	NA	0.6168	0.4599	0.9961	0.3951	0.1562	0.8756	2.0479	0.0165	1.3074	0.1839	1.6315	0.0313	0.6733	0.5978	0.8690	0.2299
AT1G77760	-4.4666	0.0000	0.6198	0.2880	0.0968	0.9157	0.0197	0.9886	-0.4344	0.6088	-0.4161	0.4937	-3.2112	0.0000	-0.8073	0.2340	0.5168	0.3438	0.4500	0.6214	-0.7605	0.2518	-4.2491	0.0000	-2.4473	0.0000	-1.8367	0.0001
AT1G77855	-1.0847	0.0934	-1.6418	0.0121	-1.5492	0.0326	-1.2235	0.1439	-1.1252	0.1650	-1.8304	0.0093	-1.8125	0.0020	-1.0308	0.2366	-2.0518	0.0013	-0.9943	0.2705	-0.0314	0.9854	-0.9717	0.1456	-0.4966	0.6640	-1.1262	0.0636
AT1G77870	-1.5874	0.0234	-1.0061	0.2163	-1.3352	0.1240	-0.5835	0.6001	-0.6298	0.5989	-1.3884	0.0794	-1.6430	0.0136	-0.3636	0.8370	-1.4991	0.0394	-0.8005	0.4931	-0.6399	0.5548	-2.3490	0.0005	-0.7696	0.4873	-0.3606	0.6255
AT1G78070	-2.0721	0.0000	0.7029	0.1908	0.1509	0.8520	-0.0455	0.9655	-0.0720	0.9553	0.4610	0.4171	-2.4407	0.0000	-0.1337	0.9223	1.1179	0.0181	0.7900	0.2229	1.4455	0.0046	-0.9967	0.0362	0.9054	0.1072	0.3613	0.4456
AT1G78110	-1.8850	0.0002	-0.6002	0.3496	-0.3994	0.6173	-0.9509	0.1879	-0.0941	0.9462	-0.3847	0.5633	-2.1443	0.0000	-0.4199	0.7084	-0.6090	0.2956	-0.3926	0.7124	-0.8378	0.2407	-1.8764	0.0002	-0.6453	0.4170	-0.5815	0.2623
AT1G78230	-2.0358	0.0000	-0.7544	0.0809	-1.3267	0.0012	-1.3197	0.0039	-1.5587	0.0000	-1.9984	0.0000	-2.1942	0.0000	-0.9902	0.0268	-1.1397	0.0038	-0.7139	0.1847	0.7758	0.1215	-1.5945	0.0000	-1.4644	0.0002	-1.5302	0.0000
AT1G78290	-2.6419	0.0000	-2.0988	0.0000	-1.7808	0.0011	-1.9865	0.0008	-2.2271	0.0000	-1.5179	0.0082	-3.1288	0.0000	-2.3505	0.0000	-1.9490	0.0002	-0.3819	0.7236	0.0490	0.9702	-2.6252	0.0000	-1.7933	0.0008	-0.9889	0.0488
AT1G78410	-2.0822	0.0025	-3.1929	0.0000	-2.5349	0.0004	-0.7486	0.4914	-1.8582	0.0166	-1.3413	0.0920	-1.8084	0.0070	-1.8096	0.0229	-2.5830	0.0002	-1.5582	0.0774	-1.0512	0.2807	-2.2354	0.0011	-1.9866	0.0083	-0.8544	0.2166
AT1G78440	0.5339	0.4840	1.5334	0.0219	0.6760	0.4770	0.6745	0.5034	-1.2111	0.1912	1.1909	0.1061	-0.3610	0.6468	0.4875	0.7342	1.3076	0.0514	0.4099	0.7744	-0.5163	0.6544	1.1364	0.0916	2.4887	0.0001	0.1648	0.8293
AT1G78600	1.1469	0.0048	1.5811	0.0001	1.6026	0.0001	1.2476	0.0121	1.7061	0.0000	1.1010	0.0150	1.0047	0.0113	1.1143	0.0152	1.5482	0.0001	0.9985	0.0462	-1.2390	0.0090	1.6923	0.0000	2.0485	0.0000	1.2042	0.0021
AT1G78970	-2.5393	0.0000	-1.5380	0.0000	-0.6388	0.1486	-0.7838	0.0865	-1.0036	0.0076	0.1351	0.7791	-3.3021	0.0000	0.1023	0.9218	-0.2458	0.5543	-0.7338	0.1033	-0.5510	0.2497	-2.3466	0.0000	0.1518	0.8446	0.1332	0.7275
AT1G79270	-2.5676	0.0000	-0.5228	0.3931	-1.1429	0.0460	-1.1884	0.0597	-1.9593	0.0000	-0.9876	0.0744	-2.6881	0.0000	-1.4503	0.0067	-0.5946	0.2747	-0.2360	0.8391	-0.4315	0.5822	-1.8250	0.0001	-0.5539	0.4782	-0.9890	0.0350
AT1G79310	0.9346	0.2259	-0.7963	0.3895	-1.1036	0.2671	0.6233	0.5900	-0.4053	0.7948	-0.4111	NA	-0.3320	0.7101	0.3742	0.8420	0.2642	0.7816	1.5505	0.0815	2.2708	0.0030	0.3399	0.7281	0.7079	0.5656	0.4660	0.5456

AT1G79440	1.2614	0.0203	1.6708	0.0019	1.6210	0.0055	1.4957	0.0253	1.5220	0.0090	2.0900	0.0002	1.2733	0.0149	1.8153	0.0013	2.3138	0.0000	2.2464	0.0001	-0.3860	0.6782	1.6266	0.0021	2.3600	0.0000	2.3249	0.0000
AT1G79680	-2.0772	0.0003	-2.5092	0.0000	-1.0301	0.1711	-0.3147	0.7631	-1.4006	0.0398	-0.5418	0.4587	-2.2909	0.0000	-2.1882	0.0003	-1.6888	0.0045	-1.4758	0.0369	-1.8231	0.0056	-2.3393	0.0000	-1.4768	0.0248	-0.8050	0.1617
AT1G79770	-0.5301	0.5189	-2.0270	0.0040	-1.2260	0.1650	-0.6294	0.5691	-0.7413	0.5043	-1.7025	0.0274	-0.6077	0.4273	-0.8116	0.4949	-2.4280	0.0005	0.0195	0.9931	0.2442	0.8620	-0.3342	0.7185	-2.0118	0.0070	-1.5941	0.0165
AT1G80130	-2.1790	0.0029	-1.4601	0.0745	-0.7276	0.5057	-1.6414	0.0938	0.3881	0.8069	-1.9556	0.0169	-2.0298	0.0043	0.0121	0.9963	-2.3178	0.0018	0.2638	0.8959	0.0979	0.9556	-1.8709	0.0128	-0.6741	0.6066	-1.0031	0.1686
AT1G80160	-0.5954	0.5043	1.1730	0.1779	0.6774	0.5459	0.9817	0.3823	-0.3932	0.8059	1.3785	0.1068	-0.6424	0.4469	0.8779	0.4995	2.0599	0.0066	1.9006	0.0342	-0.6390	0.5981	-0.1187	0.9199	-0.0477	0.9853	1.7723	0.0132
AT1G80190	-0.9147	0.0358	-1.7192	0.0000	-2.2154	0.0000	0.1370	0.8704	-1.5882	0.0002	0.9041	0.0579	-1.6537	0.0000	-0.1829	0.8755	0.7008	0.1231	-0.0040	0.9983	-1.1859	0.0171	-0.8938	0.0439	-0.0712	0.9474	0.4499	0.2903
AT1G80240	-1.0757	0.1392	0.0411	0.9710	0.9641	0.2785	-0.1562	0.9098	-0.7067	0.5308	-0.5446	0.5313	-2.0705	0.0016	-0.3108	0.8637	-0.1888	0.8358	-0.9872	0.3509	0.1486	0.9208	-1.5038	0.0351	0.2687	0.8609	1.1199	0.0810
AT1G80555	-0.8279	0.1850	2.7118	0.0000	2.6769	0.0000	-0.2903	0.7703	2.9517	0.0000	-0.7076	0.3339	2.7149	0.0000	-0.4857	0.6764	-1.1513	0.0800	-0.1817	0.9086	-0.0949	0.9431	-0.6741	0.3036	-0.8428	0.3405	-0.3883	0.5392
AT1G80760	-1.1631	0.0235	-1.4229	0.0063	-1.7331	0.0011	-0.8316	0.2508	-0.8201	0.2352	-2.2495	0.0000	-1.3984	0.0039	-1.1915	0.0476	-1.4456	0.0049	-0.8982	0.2052	0.0171	0.9915	-1.2854	0.0122	-0.5926	0.4575	-0.7096	0.1538
AT1G80820	-1.0841	0.0827	-1.8674	0.0022	-1.4028	0.0476	-0.5463	0.5700	-2.0007	0.0015	-1.7256	0.0101	-1.5235	0.0082	-1.7912	0.0068	-1.7680	0.0039	-1.0813	0.1945	-0.8608	0.3104	-1.2888	0.0380	-1.5580	0.0206	-1.8450	0.0016
AT1G80840	-2.0170	0.0001	-1.8663	0.0003	-1.4670	0.0118	-0.0229	0.9879	-2.0584	0.0001	-0.9455	0.1159	-2.6945	0.0000	-2.0874	0.0001	-1.4153	0.0079	-1.5411	0.0102	0.6717	0.3766	-2.6222	0.0000	-2.1393	0.0000	-1.3270	0.0081
AT1G80870	-0.6394	0.3060	0.0289	0.9762	-0.2101	0.8285	-0.8151	0.3203	0.1450	0.9161	-0.9891	0.1254	-0.1314	0.8602	0.1077	0.9553	-0.9778	0.1026	-0.3548	0.7714	-0.3288	0.7435	-0.3591	0.6193	-0.2383	0.8490	-2.0901	0.0001
AT1G80920	-1.0120	0.1202	0.4782	0.5562	0.3157	0.7558	-0.1052	0.9383	0.0426	0.9836	0.9411	0.1906	-1.3234	0.0287	0.0160	0.9948	0.6352	0.3658	0.2418	0.8810	-2.5781	0.0000	-1.1520	0.0764	0.3087	0.8186	0.4501	0.4816
AT1G80960	-0.2542	0.4515	-1.7841	0.0000	-1.7599	0.0000	-0.4516	0.2796	-1.6791	0.0000	0.1197	0.7651	-2.1131	0.0000	-0.5008	0.2162	-0.2828	0.4016	-0.3072	0.5553	-0.7333	0.0412	-0.6143	0.0419	0.2851	0.5602	-0.2784	0.3564
AT1G80970	-0.8593	0.1103	-3.9334	0.0000	-3.8668	0.0000	-0.5051	0.5295	-3.8935	0.0000	-0.3800	0.5635	-4.4736	0.0000	-0.0866	0.9610	-0.3928	0.5146	-0.2033	0.8770	0.1200	0.9148	-0.6046	0.2958	-0.2716	0.8048	-0.5405	0.3011
AT1G80980	-0.5109	0.3926	-3.2569	0.0000	-3.2531	0.0000	-0.4922	0.5559	-1.8490	0.0006	-0.2996	0.6636	-4.4052	0.0000	0.1461	0.9258	-0.4802	0.4270	-0.0720	0.9665	-0.1340	0.9082	-0.1890	0.7969	0.0939	0.9437	-0.6390	0.2191
AT2G01200	-0.8480	0.2835	1.3614	0.0633	0.8107	0.4010	-0.0999	0.9484	1.3050	0.1232	0.8736	NA	0.0616	0.9487	1.1798	0.1967	0.4127	0.6288	0.8785	0.4227	-0.2479	0.8658	-0.4759	0.5969	2.2110	0.0011	0.5961	0.4038
AT2G01520	3.0815	0.0000	1.4159	0.0599	0.7195	0.4766	0.4050	0.7391	1.2274	0.1748	1.5255	0.0486	1.9458	0.0029	2.2383	0.0020	1.2880	0.0776	3.6749	0.0000	1.7566	0.0311	2.3481	0.0004	2.1684	0.0025	1.9595	0.0028
AT2G01530	0.6302	0.4733	2.0525	0.0070	1.3541	0.1514	0.5359	0.6676	0.8867	0.4413	1.0952	0.2102	0.5879	0.4898	1.5351	0.1003	1.6976	0.0275	2.2401	0.0075	0.6204	0.6110	0.3847	0.7014	1.3656	0.1453	1.5731	0.0271
AT2G02930	-2.2098	0.0001	-1.3879	0.0236	-1.0324	0.1602	-0.9120	0.2652	-0.9374	0.2291	-0.2497	0.7503	-2.6997	0.0000	-1.0053	0.2020	-0.6892	0.2807	-0.4037	0.7343	-1.5131	0.0275	-2.2901	0.0000	-1.3048	0.0532	1.3090	0.0152
AT2G02990	-2.7243	0.0000	-1.0099	0.1886	-1.4622	0.0628	-0.7554	0.4560	-1.5153	0.0512	-1.5667	0.0334	-3.2505	0.0000	-1.9521	0.0069	-1.5665	0.0218	-0.7066	0.5437	-2.6798	0.0001	-2.4588	0.0001	-1.1141	0.1963	-0.7866	0.2279
AT2G03505	-2.0398	0.0034	-1.5972	0.0345	-1.4052	0.1061	0.2379	0.8629	-1.0589	0.2830	-1.0176	0.2183	-1.4075	0.0426	-1.1409	0.2566	-1.7232	0.0180	-0.7012	0.5839	-0.8586	0.4064	-1.3314	0.0720	-1.6790	0.0373	-1.1846	0.0829
AT2G03530	-0.7563	0.2097	-2.1576	0.0001	-0.5629	0.4889	-1.1574	0.1196	-0.0499	0.9784	-1.3267	0.0320	-1.0731	0.0518	-0.6987	0.4476	-2.2396	0.0000	-0.8932	0.2691	-0.0584	0.9655	-0.8233	0.1757	-0.3715	0.7352	-1.0893	0.0411
AT2G03880	1.3081	0.0736	1.1932	0.1421	0.3539	0.7689	1.1914	0.2333	1.2346	0.1898	0.8189	NA	1.8020	0.0067	1.0741	0.3093	1.0982	0.1546	1.2554	0.2047	1.7614	0.0355	2.2094	0.0011	1.0847	0.2727	-0.0814	0.9257
AT2G04030	0.4521	0.4337	-0.2657	0.7036	-0.7795	0.2316	-0.0618	0.9557	-0.4996	0.5456	-0.9056	0.1100	0.5451	0.3088	-0.0163	0.9940	-0.8665	0.1010	-0.1623	0.9069	-0.0052	1.0000	0.5694	0.3176	-0.8069	0.2209	-2.2752	0.0000
AT2G04039	-0.4326	0.4507	-1.1621	0.0253	-0.8720	0.1639	-0.6010	0.4196	-0.4162	0.6389	-1.3850	0.0102	-0.3823	0.4948	-0.9385	0.1420	-1.9131	0.0001	-1.0161	0.1114	-0.0163	0.9917	-0.5171	0.3654	-1.3540	0.0108	-2.7759	0.0000
AT2G04050	-0.2444	0.7953	-0.6696	0.4508	-0.8637	0.3767	2.4153	0.0027	-0.0555	0.9811	-1.3375	0.0955	-0.2580	0.7718	-0.6515	0.6374	-0.5966	0.4636	0.4074	0.7943	-0.6507	0.5549	-0.4189	0.6439	-1.4539	0.0853	0.7203	0.3011
AT2G04070	-0.9231	0.2628	-1.7929	0.0236	-1.5413	0.0919	2.3249	0.0099	-0.5144	0.7240	-1.6624	0.0478	-0.7185	0.3860	-1.2347	0.2444	-2.0193	0.0079	-0.9053	0.4687	-0.6394	0.5981	-1.2280	0.1284	-1.3621	0.1509	-0.6830	0.3681
AT2G04450	-3.2110	0.0000	-2.7958	0.0000	-2.3242	0.0000	-2.4231	0.0000	-0.4781	0.5788	-2.6822	0.0000	-2.2618	0.0000	-2.5675	0.0000	-3.1541	0.0000	-2.3395	0.0000	-1.8146	0.0007	-3.4853	0.0000	-2.4232	0.0000	-2.6976	0.0000
AT2G04495	-1.8314	0.0148	-2.5682	0.0005	-1.4397	0.1240	-1.9117	0.0442	-0.7450	0.5547	-2.0588	0.0124	-2.2442	0.0016	-1.9595	0.0208	-2.6153	0.0004	-1.0067	0.3970	-1.1081	0.2912	-2.2782	0.0019	-2.0584	0.0111	-1.5160	0.0344
AT2G04790	-1.1458	0.0145	-1.3545	0.0047	-1.3524	0.0093	-1.1740	0.0486	-0.9583	0.0962	-1.6429	0.0013	-0.8126	0.0815	-1.1807	0.0282	-2.0650	0.0000	-1.0012	0.0954	-0.2338	0.7881	-0.8945	0.0675	-1.4190	0.0047	-3.3667	0.0000
AT2G04795	-0.4723	0.4781	-1.6714	0.0037	-2.1093	0.0003	-1.3970	0.0548	-0.7682	0.3555	-1.3478	0.0336	-1.0206	0.0711	-1.0900	0.1396	-1.5559	0.0072	0.1962	0.8984	0.0887	0.9460	-0.3932	0.5822	-2.1947	0.0001	-1.9572	0.0003
AT2G05230	-0.0285	0.9802	0.0732	0.9537	0.1701	0.9037	-0.1592	0.9184	3.5106	0.0000	-0.3909	0.6969	-0.4417	0.6220	0.4047	0.8344	-0.3512	0.7116	-0.1480	0.9504	-0.8295	0.4631	0.0323	0.9810	-0.1373	0.9423	0.1094	0.9001
AT2G05440	1.8245	0.0153	0.8168	0.3813	0.1489	0.9168	0.6966	0.5624	0.3583	0.8275	1.7377	0.0374	1.0592	0.1744	1.7971	0.0405	1.4685	0.0623	2.4789	0.0028	-0.0046	1.0000	1.7178	0.0253	0.8423	0.4821	0.8858	0.2331
AT2G05940	-0.7979	0.2207	-1.5968	0.0099	-0.9489	0.2273	-1.0002	0.2399	-0.5229	0.6247	-1.6847	0.0105	-1.1756	0.0477	-1.0927	0.1736	-2.1375	0.0003	-0.6201	0.5643	0.5920	0.5237	-0.9895	0.1247	-1.1892	0.1035	-1.6931	0.0030
AT2G06355	-2.2783	0.0001	-2.8750	0.0000	-1.9285	0.0025	-1.8242	0.0116	-0.7935	0.3637	-1.4376	0.0310	-1.7035	0.0024	-1.2742	0.0810	-2.0307	0.0007	-2.6894	0.0000	-1.5259	0.0311	-2.3970	0.0000	-2.2522	0.0002	-2.1310	0.0002
AT2G06995	2.0334	0.0003	-0.5960	0.4781	-0.2365	0.8326	2.6552	0.0000	0.7697	0.4051	3.0926	0.0000	-0.8999	0.1722	2.5593	0.0000	2.6635	0.0000	2.4564	0.0000	2.5979	0.0000	2.0214	0.0003	0.6427	0.5359	2.1117	0.0002
AT2G07042	-0.3628	0.2974	-4.1292	0.0000	-4.4900	0.0000	-0.3803	0.4281	-4.6643	0.0000	-0.1213	0.7854	-5.2285	0.0000	-0.5032	0.2729	-0.2319	0.5418	-0.3053	0.6058	-0.1956	0.7307	-0.7914	0.0145	-0.6961	0.0784	-0.5121	0.1142
AT2G07751	-0.7801	0.3417	-0.2397	0.8280	-0.1668	0.9029	0.9023	0.4238	0.2342	0.8929	0.2187	NA	-0.7601	0.3436	-0.0820	0.9753	-0.0005	1.0000	2.4313	0.0030	-0.1664	0.9191	-0.6315	0.4759	0.0358	0.9889	0.4371	0.5732
AT2G11810	1.3053	0.0812	-0.7244	0.4345	0.8783	0.3862	0.5782	0.6263	2.0272	0.0089	-0.3634	0.7135	1.6717	0.0163	1.5395	0.0818	-0.6351	0.4566	0.3554	0.8357	0.6733	0.5549	1.8550	0.0102	0.6604	0.6045	0.7582	0.2962
AT2G12462	-0.6758	0.3649	-1.0078	0.1893	0.1173	0.9262	-1.0987	0.2393	0.3555	0.7985	-1.4451	0.0506	-0.7274	0.3044	-0.0091	0.9968	-2.0578	0.0018	-0.9977	0.3101	-0.0034	1.0000	-0.6074	0.4406	0.6799	0.5328	-1.6916	0.0073
AT2G13570	-1.2357	0.0816	-0.3267	0.7356	-0.2898	0.7993	-1.1189	0.2470	-0.4411	0.7399	-0.6029	NA	-0.9242	0.1837	-0.8577	0.4421	-1.0656	0.1505	-0.9992	0.3372	-1.0924	0.2480	-1.3458	0.0597	-1.0132	0.2927	-2.0067	0.0030
AT2G13790	-1.0990	0.0085	-2.0437	0.0000	-1.0756	0.0237	-1.1126	0.0334	-0.2164	0.8094	-0.9117	0.0526	-1.6146	0.0000	-1.4524	0.0009	-1.7252	0.0000	-1.6297	0.0002	-0.5627	0.3515	-1.5094	0.0002	-1.5766	0.0002	-1.5732	0.0001
AT2G13810	-1.7794	0.0138	-2.6997	0.0001	-1.6881	0.0450	-0.3130	0.8176	-1.1760	0.2283	-1.6444	0.0413	-0.6652	0.4061	-2.4510	0.0012	-2.3782	0.0009	-2.0651	0.0129	-1.8070	0.0354	-2.1336	0.0027	-2.3493	0.0017	-1.9710	0.0042
AT2G14247	3.1744	0.0000	-0.2259	0.8416	-0.1444	0.9191	1.2559	0.2276	-0.0730	0.9755	-0.1099	0.9239	3.9179	0.0000	-0.0147	0.9957	-0.0769	0.9426	-0.4710	0.7721	-0.1823	0.9127	4.9814	0.0000	-0.5978	0.6658	-0.4959	0.5274
AT2G14610	-0.4772	0.6053	-2.2984	0.0021	-1.4381	0.1224	-1.3405	0.1967	0.0834	0.9703	-1.2530	0.1464	-0.3149	0.7369	-1.8415	0.0332	-1.7695	0.0217	-1.4316	0.1531	-1.3180	0.1875	-0.6010	0.5129	-1.1024	0.2887	-0.4113	0.6062
AT2G15020	-3.7057	0.0000	-1.7020	0.0279	-2.0102	0.0130	-2.7551	0.0008	-2.8776	0.0001	-3.6245	0.0000	-3.4858	0.0000	-3.0457	0.0000	-2.6122	0.0003	-0.0417	0.9841	-0.1940	0.9041	-1.8311	0.0129	-2.6162	0.0004	-2.7712	0.0001
AT2G15042	-1.7267	0.0061	-2.8089	0.0000	-1.6241	0.0240	-2.2015	0.0029	-0.9427	0.2914	-1.5313	0.0298	-1.5907	0.0092	-1.0373	0.2496	-2.0541	0.0011	-1.6756	0.0249	-1.5645	0.0388	-1.9922	0.0013	-2.0480	0.0019	-1.8695	0.0020
AT2G15090	1.9313	0.0000	0.7958	0.0410	1.3609	0.0002	1.1236	0.0099	1.4852	0.0000	0.9314	0.0199	1.6121	0.0000	1.2555	0.0009	0.6286	0.1040	0.4343	0.4747	0.6831	0.1513	1.8445	0.0000	2.0151	0.0000	1.2585	0.0002
AT2G15220	-1.5550	0.0110	-1.1709	0.0840	-1.2222	0.1053	-1.8391	0.0138	-1.6733	0.0134	-0.7501	0.3042	-2.1046	0.0002	-1.7691	0.0089	-1.4883	0.0191	-0.9744	0.2725	-2.0349	0.0029	-2.1680	0.0003	-1.4565	0.0384	-1.6072	0.0068
AT2G15390	-1.6297	0.0136	-2.9275	0.0000	-1.0364	0.2317	-1.0820	0.2495	-0.4358	0.7370	-1.0350	0.1772	-1.8565	0.0031	-1.3088	0.1214	-2.6283	0.0000	-1.3271	0.1257	-0.9424	0.3113	-2.0804	0.0012	-1.5002	0.0485	-1.2670	0.0450
AT2G15480	-1.8169	0.0007	-1.8120	0.0009	-0.7873	0.2913	0.7447	0.3619	-0.5510	0.5522	-0.5600	0.4102	-2.2333	0.0000	-0.8822	0.2679	-1.1508	0.0462	-0.4331	0.6959	-0.5637	0.5067	-1.6721	0.0021	-1.5378	0.0101	-1.0580	0.0460
AT2G15760	-1.4254	0.0514	-2.2982	0.0013	-1.3190	0.1439	-0.3865	0.7610	-0.2896	0.8572	-1.5510	0.0536	-1.3444	0.0574	-0.5759	0.7059	-2.2976	0.0014	-0.5081	0.7306	-0.2016	0.8965	-1.3866	0.0634	-1.2869	0.1563	-1.2573	0.0682
AT2G15880	0.4541	0.6171	1.1729	0.1634	0.8329	0.4228	1.5397	0.1137	-0.1414	0.9416	1.4919	0.0697	-0.1926	0.8428	1.1972	0.2471	2.1777	0.0029	1.8335	0.0365	-1.1709	0.2413	0.0790	0.9484	0.4569	0.7561	1.6714	0.0164
AT2G15890	0.9003	0.1897	1.5208	0.0237	1.5850	0.0296	1.4175	0.0903	1.6462	0.0208	2.0671	0.0025	0.9420	0.1516	1.4437	0.0585	2.1679	0.0006	2.1236	0.0025	-1.9055	0.0077	0.9507	0.1718	1.8735	0.0056	2.2141	0.0002
AT2G15960	-0.1211	0.8972	1.1892	0.1033	0.8731	0.3283	0.6855	0.5011	0.5794	0.6143	2.0626	0.0033	-0.2532	0.7597	0.9602	0.3297	1.8172	0.0057	1.2912	0.1342	-1.7286	0.0231	0.0709	0.9481	0.9594	0.2904	1.5762	0.0112
AT2G15970	0.7549	0.2347	2.0799	0.0002	1.7455	0.0057	1.3022	0.0888	1.0389	0.1722	1.4385	0.0259	0.7745	0.2050	1.4397	0.0325	2.3343	0.0000	2.1056	0.0007	-0.4647	0.6291	1.2944	0.0305	2.9186	0.0000	3.1340	0.0000
AT2G16660	2.0779	0.0000	0.3947	0.4598	0.3455	0.5854	1.1854	0.0219	0.5636	0.3618	0.2548	0.6422	2.0869	0.0000	0.7056	0.2294	0.4635	0.3268	0.4321	0.5668	-0.6350	0.2794	1.2893	0.0016	-0.4718	0.4838	-0.5372	0.1963
AT2G16720	-2.4287	0.0000	-0.6191	0.3845	-0.1492	0.8871	-1.2171	0.1106	0.4016	0.7124	-0.9945	0.1332	-2.1653	0.0000	-0.4587	0.7099	-1.5101	0.0104	-0.8216	0.3503	0.1663	0.8929	-1.6535	0.0037	0.3051	0.8022	-0.8784	0.1169
AT2G17660	-0.9053	0.2481	0.8294	0.3226	0.0268	0.9860	0.3900	0.7483	-0.5953	0.6496	-0.4426	NA	-0.3891	0.6411	-1.1019	0.3012	-0.0133	0.9914	-0.2381	0.9037	2.9570	0.0000	-0.9973	0.2088	-0.4818	0.7352	-0.6355	0.3901
AT2G17740	-0.8007	0.1760	-2.7110	0.0000	-0.7812	0.2966	-1.2900	0.0737	-0.7439	0.3609	-1.0832	0.0859	-1.1825	0.0284	-1.9370	0.0009	-2.3730	0.0000	-1.2212	0.0814	-2.4855	0.0000	-0.9455	0.1082	-1.3006	0.0410	-0.5244	0.3477
AT2G17840	0.2829	0.6132	1.1634	0.0129	1.0342	0.0507	0.5580	0.4159	1.1545	0.0222	1.3059	0.0082	0.2722	0.6122	1.0071	0.0638	1.5964	0.0003	1.2073	0.0220	1.3873	0.0056	0.4592	0.3857	2.3572	0.0000	2.2145	0.0000
AT2G17880	-1.0908	0.0958	-0.0690	0.9470	0.1708	0.8801	0.0964	0.9453	-0.7803	0.4109	0.4411	0.5821	-1.5014	0.0133	-0.2937	0.8608	0.0600	0.9474	-0.3641	0.7957	-2.3036	0.0005	-1.3846	0.0315	0.3432	0.7966	0.7630	0.2173
AT2G18120	-0.9855	0.1755	-0.8066	0.3320	-0.4917	0.6384	-1.0373	0.2910	-0.6648	0.5623	-1.3401	0.0860	-0.6158	0.4107	-0.5494	0.7057	-2.0091	0.0040	0.1383	0.9483	0.6749	0.5157	-0.2466	0.7948	-0.2264	0.8879	-1.7448	0.0085
AT2G18150	2.9379	0.0000	-0.0862	0.9396	-0.5982	0.5737	0.9891	0.3298	1.4136	0.0952	0.7801	0.3528	1.3572	0.0463	1.2448	0.1808	0.2360	0.7970	2.4802	0.0008	0.8787	0.3839	2.3544	0.0004	1.2312	0.1633	0.4786	0.5115
AT2G18193	-1.7147	0.0102	-0.2683	0.7846	-0.5288	0.6004	2.0998	0.0082	-0.9183	0.3448	-0.6444	0.4361	-1.5952	0.0134	-1.1249	0.2283	-0.7282	0.3334	-0.0026	1.0000	-1.5214	0.0648	-1.8583	0.0052	-1.7287	0.0198	-1.4434	0.0245
AT2G18328	0.2708	0.7548	-1.8495	0.0056	-1.1320	0.1797	-0.3267	0.7820	0.4372	0.7327	-1.2715	0.0870	0.0042	0.9976	0.0043	0.9994	-2.0023	0.0023	-0.1280	0.9509	0.8782	0.3508	0.1019	0.9211	-0.4014	0.7624	-1.5700	0.0121
AT2G18370	1.7445	0.0033	0.7867	0.2885	-0.1614	0.8898	-0.0425	0.9768	0.8936	0.3044	0.8552	0.2395	0.4140	0.5610	1.1270	0.1632	0.9437	0.1529	2.5028	0.0001	1.8531	0.0060	1.0828	0.0925	1.5222	0.0877	2.5550	0.0000
AT2G18540	-0.5000	0.5728	2.2444	0.0022	0.1193	0.9338	-0.1023	0.9502	-0.3862	0.8018	-0.1094	NA	-0.6367	0.4369	0.4063	0.8278	0.3841	0.6726	-0.1504	0.9478	0.8230	0.4534	-0.0538	0.9660	0.2244	0.8994	1.0373	0.1453
AT2G18660	-2.0248	0.0032	-2.2778	0.0010	-1.5531	0.0613	-1.4749	0.1130	-0.1132	0.9529	-1.4076	0.0743	-1.6581	0.0142	-2.0719	0.0065	-2.3329	0.0008	-1.4326	0.1163	-1.1837	0.2100	-2.0281	0.0033	-1.3659	0.1105	-0.7043	0.3143
AT2G18680	-2.1591	0.0003	-1.3374	0.0439	-1.1202	0.1508	-0.0066	0.9976	-1.0514	0.1973	-0.4090	0.6036	-2.4117	0.0000	-1.7429	0.0107	-1.2701	0.0489	-1.0511	0.2245	-0.2510	0.8355	-1.9386	0.0013	-1.3366	0.0652	0.2654	0.6904
AT2G18690	-2.2047	0.0000	-1.9074	0.0001	-0.8374	0.2012	-1.1191	0.0910	-1.3368	0.0165	-0.6466	0.2808	-2.2584	0.0000	-2.0904	0.0000	-1.9739	0.0001	-1.7399	0.0015	-1.8631	0.0005	-2.9757	0.0000	-1.6686	0.0013	-0.1916	0.7291
AT2G18735	-0.1168	0.8719	0.4626	0.4755	0.1174	0.9017	0.4440	0.5873	0.1393	0.9103	0.7443	0.2096	0.5814	0.2799	0.5087	0.5952	0.7745	0.1547	0.8267	0.2551	0.8571	0.2093	0.3348	0.6050	-0.2286	0.8421	2.1705	0.0000
AT2G19190	-2.6256	0.0000	-2.2731	0.0000	-0.6385	0.3761	-1.7646	0.0036	-0.4642	0.6100	-1.4590	0.0105	-2.8047	0.0000	-2.3413	0.0000	-2.8925	0.0000	-2.3523	0.0000	-3.3813	0.0000	-2.3948	0.0000	-1.1725	0.0503	-1.3730	0.0056
AT2G19350	0.0804	0.9068	0.7712	0.1572	0.4749	0.4963	0.4545	0.5490	0.4638	0.5654	0.7720	0.1605	0.3162	0.5694	0.4765	0.5971	0.7581	0.1366	0.6633	0.3670	0.2597	0.7654	0.3744	0.5225	0.6535	0.3415	2.2599	0.0000
AT2G19450	1.5828	0.0001	1.2724	0.0034	1.7895	0.0000	1.5908	0.0011	2.1224	0.0000	1.5858	0.0005	2.1680	0.0000	1.7308	0.0001	1.9773	0.0000	2.1439	0.0000	0.0216	0.9858	1.8793	0.0000	1.6945	0.0001	1.6933	0.0000
AT2G19650	-1.2670	0.0485	-1.4778	0.0260	-1.3499	0.0725	-1.9378	0.0104	-1.3065	0.0871	-2.5616	0.0001	-1.5404	0.0108	-0.9957	0.2710	-2.4017	0.0001	0.1150	0.9542	0.5529	0.5799	-1.0590	0.1134	-1.4042	0.0530	-1.9230	0.0013
AT2G19660	-2.0819	0.0000	-0.1660	0.8217	0.4831	0.4964	-0.4996	0.5173	-0.6182	0.4108	-0.3803	0.5472	-1.8637	0.0001	-0.6245	0.4418	-0.6648	0.2233	-1.0386	0.1111	0.0605	0.9587	-1.7414	0.0004	-0.0457	0.9756	0.8719	0.0612
AT2G19810	0.0454	0.9564	0.7245	0.2678	0.6448	0.4020	0.9372	0.2216	-0.3009	0.7930	1.2084	0.0478	-0.1724	0.8091	0.7809	0.3512	1.1266	0.0479	0.8664	0.2756	-0.4695	0.5943	-0.0249	0.9803	1.4890	0.0116	2.5003	0.0000
AT2G19900	0.4183	0.6439	1.4133	0.0749	0.4443	0.6979	0.1268	0.9352	0.9885	0.3467	0.1304	0.9047	0.2696	0.7697	0.0821	0.9746	0.6754	0.4145	0.4558	0.7714	1.7070	0.0510	3.3739	0.0000	0.0002	1.0000	0.4334	0.5672
AT2G19970	-0.5562	0.3945	-1.3148	0.0378	-2.2216	0.0005	-1.4856	0.0458	-1.3395	0.0561	-0.7404	0.2884	-0.8658	0.1360	-1.2437	0.0842	-1.3932	0.0240	-0.5885	0.5694	-1.8612	0.0062	-1.3292	0.0259	-0.7831	0.3606	-0.7182	0.2172
AT2G20142	-1.6580	0.0114	-2.5159	0.0001	-0.8559	0.3427	-1.2901	0.1452	-0.3280	0.8164	-1.4619	0.0468	-2.0096	0.0012	-1.9204	0.0074	-2.4360	0.0002	-1.7766	0.0205	-1.3080	0.1214	-2.3879	0.0001	-1.9242	0.0059	-0.9074	0.1571
AT2G20150	-2.9047	0.0000	-1.9577	0.0098	-1.5495	0.0792	-1.5505	0.1079	-1.1952	0.2231	-2.7841	NA	-3.1145	0.0000	-3.0399	0.0000	-2.6400	0.0003	-2.1126	0.0123	-0.6155	0.5986	-2.8745	0.0000	-2.8024	0.0001	-2.2945	0.0010
AT2G20562	-1.7060	0.0152	-2.1443	0.0024	-1.8134	0.0228	-0.5055	0.6640	-1.2340	0.1800	-1.8434	0.0176	-1.1979	0.0887	-1.2433	0.1908	-2.4076	0.0006	-1.4673	0.1060	-0.4818	0.6909	-1.2872	0.0811	-2.0607	0.0061	-0.8963	0.1942
AT2G20750	-0.6105	0.4704	-1.2014	0.1434	-0.5890	0.5873	-0.7720	0.4905	-0.0268	0.9924	-1.9750	0.0128	-0.6677	0.4020	0.2610	0.8999	-2.1315	0.0033	-0.8029	0.5192	1.2366	0.1994	-0.0077	0.9958	0.9425	0.3741	0.0803	0.9242
AT2G21130	2.3145	0.0000	2.5973	0.0000	2.1829	0.0000	2.0702	0.0006	3.4220	0.0000	1.9358	0.0008	2.9328	0.0000	2.9179	0.0000	2.8154	0.0000	2.9812	0.0000	0.4872	0.5880	3.4747	0.0000	4.0788	0.0000	2.8572	0.0000
AT2G21260	0.8948	0.2568	0.5044	0.6089	-0.2532	0.8443	1.0303	0.3281	2.6116	0.0002	-0.1212	NA	1.2756	0.0729	0.4247	0.8142	0.6223	0.4635	0.4889	0.7544	0.3519	0.8013	1.5998	0.0284	0.0121	0.9984	-0.0367	0.9675
AT2G21490	-0.5142	0.5743	2.3123	0.0020	0.1815	0.8976	-0.5972	0.6278	0.3023	0.8583	0.2415	NA	-0.5376	0.5372	0.0197	0.9948	-0.3552	0.7073	-0.1681	0.9409	1.6825	0.0682	-0.2335	0.8313	-0.4518	0.7674	0.0559	0.9494
AT2G21560	-2.4321	0.0000	-1.1316	0.0729	-1.4382	0.0283	-1.3400	0.0725	-0.8097	0.3268	-1.2197	0.0586	-2.3749	0.0000	-1.1016	0.1397	-1.1508	0.0558	0.3583	0.7740	-0.4321	0.6544	-1.8455	0.0009	-1.1397	0.1029	-1.2873	0.0190
AT2G21650	0.6613	0.3756	-1.7992	0.0077	-0.8146	0.3730	0.0762	0.9584	1.0587	0.2341	-0.4667	0.5784	0.6226	0.3902	0.7363	0.5258	-1.1158	0.1119	0.8563	0.4150	2.1913	0.0020	0.4132	0.6260	0.6947	0.5209	-0.5003	0.4603
AT2G21660	2.5174	0.0000	2.5261	0.0000	2.5479	0.0000	2.3787	0.0001	3.8047	0.0000	2.5089	0.0000	3.4792	0.0000	3.6349	0.0000	3.2278	0.0000	3.6597	0.0000	0.7581	0.3384	3.4899	0.0000	3.9483	0.0000	3.9669	0.0000
AT2G21910	-4.2871	0.0000	-1.2328	0.0998	0.6483	0.5075	-2.3316	0.0030	0.6489	0.5650	-1.8378	0.0133	-3.9047	0.0000	-1.3303	0.1210	-2.8865	0.0000	-2.9453	0.0000	-4.0769	0.0000	-4.2970	0.0000	-0.5157	0.6819	-1.2704	0.0490
AT2G22190	0.9434	0.1816	0.1501	0.8842	0.4771	0.6384	0.5529	0.6057	2.0658	0.0025	-0.1252	0.9007	1.0914	0.1013	1.8359	0.0115	0.1268	0.8885	1.2830	0.1420	1.8017	0.0173	1.4188	0.0361	1.2378	0.1298	-0.1427	0.8499
AT2G22450	0.7035	0.2205	1.8881	0.0002	1.6733	0.0029	1.3029	0.0533	1.6788	0.0021	2.0520	0.0002	0.9879	0.0603	1.5500	0.0071	2.2131	0.0000	1.8009	0.0018	-0.8855	0.2208	1.0463	0.0578	1.9591	0.0002	1.8443	0.0002
AT2G23000	0.8722	0.2598	-0.8857	0.3015	-0.5318	0.6244	0.3517	0.7822	-0.6322	0.6114	-1.2501	0.1239	0.5110	0.5313	-0.3835	0.8333	-1.3948	0.0608	-0.0789	0.9726	0.5492	0.6406	1.3997	0.0574	-1.1485	0.2226	-2.0465	0.0025
AT2G23030	-0.1300	0.9017	0.8606	0.3320	0.2171	0.8675	0.2823	0.8368	0.6622	0.6024	0.9869	0.2491	-0.5247	0.5348	1.1277	0.2844	1.5888	0.0338	1.6399	0.0677	-0.6179	0.6083	-0.2250	0.8334	1.3874	0.1201	2.2006	0.0013
AT2G23170	-2.0469	0.0000	0.6341	0.2457	0.8841	0.1220	-0.0685	0.9472	0.6757	0.2982	0.1401	0.8333	-1.8561	0.0000	0.2051	0.8690	0.4666	0.3722	-0.5969	0.4187	-1.6045	0.0011	-1.9239	0.0000	-0.0025	1.0000	-0.4768	0.2992
AT2G23590	-0.4186	0.5785	-0.4524	0.5853	-0.4077	0.6762	-0.7425	0.4343	-0.4561	0.6989	-1.6406	0.0210	0.6269	0.3450	-0.9805	0.2801	-2.2243	0.0006	-1.2284	0.1422	0.3248	0.7744	-0.2581	0.7625	-1.3022	0.0882	-2.3825	0.0001
AT2G23600	0.3861	0.5820	-1.7610	0.0022	-1.0161	0.1661	-0.6848	0.4362	-0.1619	0.9067	-1.0593	0.1056	0.7126	0.2405	-0.4285	0.7368	-1.8918	0.0009	-0.4274	0.7170	0.5456	0.5440	0.3775	0.6082	-1.5821	0.0107	-2.0968	0.0001
AT2G23680	-0.4358	0.5893	-2.3113	0.0003	-1.0055	0.2507	-0.5175	0.6334	-0.1211	0.9450	-1.0843	0.1555	-0.8458	0.2224	-1.0078	0.3021	-1.7031	0.0115	-0.6004	0.6323	0.0124	0.9981	-0.7214	0.3440	-1.8332	0.0103	-0.4068	0.5586
AT2G23910	1.6213	0.0238	1.2046	0.1419	0.8053	0.4388	1.1321	0.2699	1.4745	0.0927	1.1381	0.1758	0.7925	0.3022	1.5038	0.0887	1.2809	0.0936	1.8452	0.0287	1.3706	0.1442	2.5597	0.0001	1.2772	0.1658	0.9407	0.1896
AT2G24190	-0.8248	0.2469	-0.6572	0.4231	-0.4022	0.6981	-0.7591	0.4498	-0.3671	0.7863	-1.1538	0.1268	-0.3624	0.6415	-0.9141	0.3690	-2.0671	0.0020	-0.4300	0.7591	-0.0087	0.9999	-0.5385	0.4976	-0.9469	0.3062	-2.1342	0.0009
AT2G24240	0.8129	0.1026	0.0149	0.9861	0.5717	0.3930	0.4448	0.5596	0.3240	0.7274	1.1686	0.0265	0.7978	0.0966	0.3690	0.7226	0.4174	0.4502	0.2541	0.8183	-0.7723	0.2418	0.7531	0.1425	1.0994	0.0436	2.0214	0.0000
AT2G24600	-0.1665	0.8521	-2.3159	0.0002	-0.8918	0.2974	-0.9537	0.2977	-0.7348	0.4555	-1.0329	0.1578	-1.1694	0.0639	-1.0191	0.2607	-1.9441	0.0021	-0.5734	0.6337	1.0256	0.2337	-1.1922	0.0729	-1.3852	0.0604	-1.8057	0.0027
AT2G24850	-1.9482	0.0092	-2.8227	0.0001	-1.9391	0.0223	-1.2640	0.2339	-1.2300	0.2243	-2.4668	0.0022	-1.0738	0.1678	-2.4901	0.0016	-2.7947	0.0001	-1.3692	0.1852	-1.2234	0.2317	-2.0345	0.0065	-1.6288	0.0637	-1.6692	0.0198
AT2G25090	-2.2979	0.0001	-0.6601	0.3825	-0.6888	0.4285	-0.5219	0.5943	-1.8230	0.0068	0.1085	0.9061	-1.7252	0.0029	-0.8891	0.3352	0.2026	0.7975	-0.0097	0.9965	-0.9847	0.2419	-1.7669	0.0039	-0.6257	0.5405	0.2428	0.7144
AT2G25297	-2.4710	0.0002	-2.5418	0.0001	-1.5877	0.0453	-0.8844	0.3822	-0.8819	0.3829	-0.8840	0.2709	-2.7208	0.0000	-1.4620	0.0784	-1.9081	0.0056	-1.8565	0.0192	-1.1405	0.2143	-2.6715	0.0000	-2.1066	0.0034	-1.1004	0.0941
AT2G25440	-1.8835	0.0092	-3.2027	0.0000	-2.4650	0.0012	-1.3879	0.1609	-1.2649	0.1857	-2.0764	0.0094	-1.9151	0.0057	-2.3974	0.0018	-2.2203	0.0023	-1.0896	0.3160	-1.0659	0.2938	-1.3325	0.0811	-2.3030	0.0025	-1.6796	0.0156
AT2G25460	-2.2969	0.0000	-0.3193	0.6641	-0.7661	0.2879	-0.5181	0.5427	-1.6387	0.0040	-0.1911	0.7985	-2.6499	0.0000	-1.1220	0.0920	0.0084	0.9928	-0.5430	0.5716	0.8889	0.2161	-2.4079	0.0000	-1.4375	0.0146	-0.7348	0.1617
AT2G25470	-1.6047	0.0186	-2.1908	0.0013	-1.4723	0.0706	-1.1131	0.2465	-1.5265	0.0581	-2.3045	0.0019	-2.2327	0.0004	-2.1316	0.0036	-2.8020	0.0000	-0.5949	0.6453	-0.9718	0.3077	-1.6350	0.0175	-2.0297	0.0054	-2.0273	0.0020
AT2G25510	-1.3095	0.0703	-2.5984	0.0001	-1.7733	0.0255	-1.8088	0.0400	-0.4673	0.7324	-2.2882	0.0023	-1.0144	0.1603	-1.8011	0.0232	-2.7319	0.0001	-1.5596	0.0759	-0.1691	0.9137	-1.2260	0.0985	-1.3575	0.1133	-0.3519	0.6393
AT2G25680	-1.4005	0.0080	-1.3474	0.0152	-1.5565	0.0076	-1.5096	0.0213	-1.4477	0.0130	-2.0521	0.0003	-1.2572	0.0141	-1.4814	0.0120	-1.8322	0.0005	-0.9743	0.1810	-0.4745	0.5720	-0.5837	0.3389	-1.2766	0.0347	-2.3297	0.0000
AT2G25735	-0.1798	0.8414	-2.3225	0.0002	-0.6885	0.4595	-0.1374	0.9176	-0.9822	0.2716	-0.4610	0.5748	-0.4150	0.5836	-0.6568	0.5860	-1.5477	0.0198	-0.9811	0.3051	0.0901	0.9519	-0.3035	0.7281	-1.6410	0.0218	-0.4394	0.5124
AT2G25980	0.0000	1.0000	2.2163	0.0021	0.6415	0.5487	0.2243	0.8744	0.2239	0.8942	0.0000	NA	-0.0663	0.9474	1.5594	0.0760	0.6425	0.4363	1.2122	0.2338	2.4665	0.0015	0.0753	0.9496	0.7786	0.5008	0.7678	0.2791
AT2G26010	-2.7604	0.0000	-2.6399	0.0001	-2.3265	0.0020	-0.8767	0.4172	-1.1593	0.2284	-1.4865	0.0635	-2.6351	0.0001	-2.0691	0.0080	-1.6672	0.0235	-0.9614	0.3917	-3.1736	0.0000	-1.1992	0.1148	-0.8779	0.4186	0.4028	0.5944
AT2G26020	-3.0660	0.0000	-2.7896	0.0001	-2.6320	0.0007	-0.6425	0.5986	-2.1335	0.0082	-1.7728	0.0333	-2.9174	0.0000	-2.2428	0.0058	-2.0145	0.0080	-1.2035	0.2696	-1.9022	0.0323	-2.0811	0.0052	-1.5635	0.0802	-0.1078	0.9015
AT2G26040	-0.1850	0.8525	-2.0292	0.0053	-1.3993	0.1117	-0.6539	0.5636	-0.3322	0.8296	-1.5735	0.0491	-0.2884	0.7447	-0.1618	0.9439	-1.7925	0.0143	-0.6653	0.6166	0.3890	0.7613	-0.1291	0.9060	-0.8442	0.4427	-1.6198	0.0178
AT2G26150	-1.9580	0.0084	-1.6886	0.0343	-1.7208	0.0493	0.7316	0.5377	-0.9320	0.4089	-1.8681	0.0239	-2.1197	0.0028	-1.5320	0.1029	-1.4478	0.0651	1.0745	0.3467	0.2959	0.8420	-1.5172	0.0507	-1.4440	0.1161	-2.2115	0.0019
AT2G26170	-0.5177	0.4644	-1.9610	0.0010	-1.3719	0.0537	-0.9712	0.2591	-0.6633	0.4905	-1.6657	0.0122	-0.7677	0.2257	-0.7725	0.4344	-1.7438	0.0041	0.1726	0.9192	0.7500	0.3935	-0.6551	0.3474	-2.2289	0.0002	-1.1861	0.0401
AT2G26355	-0.5195	0.3851	0.4385	0.5167	0.0631	0.9524	-0.1953	0.8454	0.4517	0.6280	0.3747	0.5748	-0.1752	0.7960	0.1222	0.9422	0.4115	0.5015	0.7334	0.3627	0.6581	0.3867	-0.2724	0.6973	0.4445	0.6378	2.1300	0.0000
AT2G26390	-1.5165	0.0386	-2.1446	0.0036	-1.0951	0.2513	-1.3650	0.1654	-0.3668	0.8093	-1.9005	NA	-1.6545	0.0170	-2.1554	0.0065	-2.3155	0.0015	-1.8459	0.0338	-0.5201	0.6686	-2.2922	0.0012	-1.5670	0.0664	-0.9186	0.1967
AT2G26400	-2.0364	0.0057	-2.8550	0.0001	-1.8965	0.0246	-1.7183	0.0764	0.3049	0.8558	-2.3626	0.0033	-1.5427	0.0349	-1.2577	0.2257	-2.5900	0.0004	-1.1608	0.2876	-0.6903	0.5549	-1.7412	0.0216	-1.7140	0.0446	-1.2930	0.0710
AT2G26440	-0.5469	0.4750	-2.0570	0.0015	-0.3859	0.7098	-1.3111	0.1330	0.4481	0.7218	-1.8347	0.0101	-0.8680	0.2024	-0.4809	0.7406	-2.3404	0.0003	-0.9285	0.3525	-0.9065	0.3268	-0.7986	0.2775	-1.0130	0.2500	-1.3460	0.0301
AT2G26480	-1.5436	0.0436	-1.8082	0.0218	-1.1700	0.2345	-0.9264	0.4141	-0.7306	0.5633	-1.8216	NA	-1.5492	0.0351	-2.2684	0.0050	-2.0039	0.0082	-1.4613	0.1420	-1.4889	0.1226	-2.0676	0.0055	-0.8617	0.4659	-1.7127	0.0163
AT2G26530	-1.5651	0.0000	-1.0702	0.0041	-0.8145	0.0629	-0.2475	0.6917	-1.3342	0.0003	-0.3753	0.4066	-2.1003	0.0000	-1.0112	0.0135	-0.7924	0.0388	-0.7135	0.1457	0.4738	0.3722	-1.6688	0.0000	-1.1855	0.0020	-0.3980	0.2782
AT2G26560	-0.5830	0.2274	-1.9411	0.0000	-0.4685	0.4675	-0.4054	0.5700	-0.3785	0.6361	0.4597	0.3965	-1.0078	0.0196	-0.5171	0.5020	-0.5918	0.2233	-0.8535	0.1508	-2.5128	0.0000	-1.1376	0.0109	-0.6938	0.2480	0.2145	0.6536
AT2G26750	-0.9376	0.2131	-1.4501	0.0587	-2.3630	0.0018	-0.8584	0.4155	0.1658	0.9239	-2.2492	0.0038	-1.1624	0.0961	-1.3293	0.1459	-1.7734	0.0149	-1.2501	0.1988	-1.8815	0.0237	-1.5723	0.0287	-0.5684	0.6572	-0.4595	0.5302
AT2G27420	-1.8542	0.0102	-2.1821	0.0026	-2.0491	0.0101	-1.3636	0.1704	-2.0961	0.0067	-2.3501	0.0024	-2.0376	0.0031	-2.0894	0.0082	-2.4280	0.0007	-0.2696	0.8888	-0.2089	0.8944	-1.1300	0.1494	-2.1363	0.0052	-2.2979	0.0008
AT2G27550	0.6325	0.4725	0.8898	0.3305	0.2282	0.8626	0.3456	0.8029	1.1915	0.2422	-0.1531	NA	1.1708	0.1240	0.6476	0.6733	0.5091	0.5716	0.5987	0.6873	0.1214	0.9443	2.7204	0.0001	0.5468	0.7026	0.1007	0.9071
AT2G27660	-2.4934	0.0001	-3.1340	0.0000	-1.7535	0.0157	-2.3211	0.0019	-1.6077	0.0296	-2.5443	0.0002	-3.1482	0.0000	-3.2022	0.0000	-3.4419	0.0000	-2.1352	0.0030	-1.8864	0.0107	-3.0894	0.0000	-2.7274	0.0000	-2.3658	0.0001
AT2G28040	-1.9600	0.0000	0.4679	0.4207	0.4879	0.4686	-0.1013	0.9159	-0.2197	0.8325	-0.0133	0.9875	-0.9076	0.0509	-0.2116	0.8675	-0.5163	0.3430	-1.5810	0.0053	0.1828	0.8445	-2.0270	0.0000	-0.5765	0.4448	-0.4121	0.4074
AT2G28190	-3.7885	0.0000	0.1582	0.7521	0.2230	0.6819	0.2084	0.7286	0.0592	0.9498	0.0196	0.9728	-3.8449	0.0000	-0.1966	0.8212	0.1453	0.7429	-0.2544	0.7202	-0.0639	0.9358	-3.1616	0.0000	-0.1158	0.8874	-0.1916	0.6029
AT2G28400	-2.5994	0.0000	-0.1790	0.8009	-0.3678	0.6130	-0.6700	0.3378	-0.5430	0.4664	0.3169	0.6040	-3.2270	0.0000	-0.6201	0.4228	-0.1741	0.7790	-0.6173	0.4191	-0.4361	0.5615	-2.7976	0.0000	0.2952	0.7579	0.7305	0.1130
AT2G28570	-1.7290	0.0000	-1.6355	0.0001	-1.2976	0.0062	-1.2487	0.0188	-1.2042	0.0110	-1.0855	0.0249	-2.3581	0.0000	-1.5640	0.0005	-1.4509	0.0009	-1.1752	0.0205	-0.5975	0.3318	-1.7226	0.0000	-1.8466	0.0000	-0.9108	0.0291
AT2G28620	0.6695	0.2506	0.1265	0.8792	-0.1613	0.8642	0.2985	0.7519	0.5520	0.5277	-0.4583	0.4968	0.4295	0.4786	0.7329	0.3821	-0.4845	0.4340	-0.0740	0.9665	0.2330	0.8230	0.6668	0.2665	0.0179	0.9942	-2.0560	0.0001
AT2G28900	1.7554	0.0000	1.8032	0.0000	1.8952	0.0000	1.2186	0.0200	2.9618	0.0000	2.0128	0.0000	2.1340	0.0000	2.6457	0.0000	2.4363	0.0000	2.1578	0.0000	-0.0049	1.0000	2.2855	0.0000	3.5561	0.0000	2.9383	0.0000
AT2G28960	1.2061	0.1203	1.6351	0.0379	1.6798	0.0524	0.7019	0.5539	0.6662	0.6113	0.2598	NA	0.0587	0.9555	1.1561	0.2812	0.3945	0.6703	1.2956	0.2096	3.1945	0.0000	0.1260	0.9144	1.3963	0.1297	1.2782	0.0745
AT2G28970	2.1783	0.0012	0.7385	0.4207	1.0370	0.2885	0.6108	0.6027	1.7096	0.0355	0.7090	NA	1.6919	0.0107	1.5028	0.0825	0.4638	0.6071	1.2132	0.2328	1.3973	0.1339	1.9008	0.0059	2.2167	0.0024	1.7999	0.0084
AT2G29090	-2.0022	0.0007	0.4373	0.5815	-0.6691	0.4357	-0.0955	0.9425	-0.2906	0.8227	-1.8066	0.0068	-1.6209	0.0046	-0.7947	0.4086	-0.7152	0.2841	-0.7966	0.4038	-1.2806	0.0916	-0.3825	0.6188	0.5550	0.5900	-0.0185	0.9797
AT2G29110	-2.2114	0.0001	-2.9275	0.0000	-1.8955	0.0028	-2.3315	0.0005	-1.4304	0.0356	-1.1112	0.0989	-2.2237	0.0000	-2.9130	0.0000	-3.1282	0.0000	-2.2060	0.0005	-1.5761	0.0228	-2.9600	0.0000	-2.4493	0.0000	-2.1117	0.0002
AT2G29120	-1.7994	0.0052	-2.4297	0.0001	-1.2608	0.1185	-1.7786	0.0280	-0.5223	0.6631	-2.1285	0.0023	-1.9736	0.0013	-1.7786	0.0140	-3.3436	0.0000	-0.9593	0.3304	-0.9895	0.2727	-2.2253	0.0004	-1.5435	0.0367	-2.3401	0.0001
AT2G29130	-1.3352	0.0634	-2.1756	0.0021	-1.3087	0.1366	-1.2487	0.1966	-0.5738	0.6504	-1.6409	0.0360	-1.2143	0.0812	-1.1282	0.2564	-2.0576	0.0038	-1.2578	0.1925	-0.4615	0.7035	-1.4077	0.0524	-0.6510	0.5884	-0.5152	0.4748
AT2G29170	0.1391	0.8814	-0.7715	0.3387	-0.5707	0.5629	0.6949	0.4900	-0.6005	0.5998	-1.9163	0.0101	0.1572	0.8550	-0.9589	0.3369	-2.0944	0.0020	-1.2645	0.1554	-0.9202	0.3281	0.8992	0.2063	0.4832	0.6962	-1.7704	0.0063
AT2G29330	0.1862	0.8602	0.3068	0.7805	0.1894	0.8912	0.0955	0.9552	0.5582	0.6925	-0.4198	NA	0.0547	0.9582	0.9651	0.4290	0.9492	0.2495	1.1282	0.3122	1.1977	0.2412	-0.2747	0.7939	0.6785	0.6061	2.3328	0.0009
AT2G29350	-0.5707	0.4853	-1.9004	0.0075	-2.0265	0.0074	0.3785	0.7596	-0.5190	0.6910	-1.3901	0.0756	-1.0023	0.1620	-2.4376	0.0007	-1.1552	0.1188	-0.2702	0.8821	-1.4655	0.0930	-0.0123	0.9926	-1.9664	0.0081	-0.9007	0.1859
AT2G29460	-2.7073	0.0000	-1.9718	0.0030	-1.5664	0.0414	0.4879	0.6576	-1.6827	0.0234	-0.2912	0.7473	-2.7227	0.0000	-1.7694	0.0167	-1.0387	0.1434	-0.7611	0.4977	-2.3792	0.0007	-2.3748	0.0002	-2.2219	0.0011	-1.4281	0.0237
AT2G29470	-3.3016	0.0000	-2.8409	0.0001	-2.2154	0.0049	-0.6942	0.5494	-3.1753	0.0000	-0.9538	0.2678	-3.0083	0.0000	-2.8031	0.0002	-1.4625	0.0553	-1.5165	0.1084	-3.8344	0.0000	-3.0157	0.0000	-3.1278	0.0000	-1.8605	0.0075
AT2G29620	0.9260	0.2360	1.8585	0.0115	0.8697	0.3920	0.8708	0.4274	0.5504	0.6870	2.3643	0.0019	0.7590	0.3263	0.4758	0.7801	1.8148	0.0125	1.0529	0.3358	0.1760	0.9144	0.9377	0.2416	1.4454	0.0959	0.8820	0.2210
AT2G30020	0.0375	0.9658	-0.0171	0.9865	-0.4031	0.6442	0.4258	0.6486	0.0203	0.9927	-0.5453	0.4371	-0.2625	0.7056	-0.1199	0.9497	-0.6717	0.2857	-0.3179	0.8026	2.6512	0.0000	-0.1497	0.8586	-0.5256	0.5919	0.2535	0.6795
AT2G30140	-2.6238	0.0000	-0.8330	0.0427	-0.8158	0.0758	-0.8280	0.1012	-1.0906	0.0080	-0.2962	0.5413	-2.8932	0.0000	-1.4868	0.0001	-0.6083	0.1378	-0.7056	0.1770	-1.7549	0.0000	-2.4938	0.0000	-1.7416	0.0000	-0.9781	0.0073
AT2G30210	3.0960	0.0000	0.7392	0.3679	0.9618	0.2797	1.0175	0.2845	1.0345	0.2554	1.7132	0.0182	1.6092	0.0111	1.4327	0.0745	0.8578	0.2398	2.1496	0.0031	1.8088	0.0171	2.3698	0.0002	2.0229	0.0034	1.8877	0.0026
AT2G30320	-0.0070	0.9944	-0.4740	0.5031	-0.4035	0.6336	-0.5278	0.5472	0.0623	0.9697	-0.6780	0.3018	0.1008	0.8900	-0.0986	0.9591	-1.1766	0.0404	-0.4942	0.6382	-0.3980	0.6713	0.0368	0.9693	-0.3814	0.7199	-2.3546	0.0000
AT2G30750	-0.8399	0.2199	-1.4371	0.0328	-0.9564	0.2533	-0.1882	0.8808	-1.3248	0.0840	-0.7027	0.3562	-1.5588	0.0103	-2.1262	0.0013	-1.1246	0.0929	-1.0193	0.2643	-2.3776	0.0004	-1.2321	0.0617	-1.3836	0.0602	-0.8409	0.1736

AT2G30770	-2.7523	0.0001	-2.2338	0.0023	-1.6540	0.0548	-0.7138	0.5396	-1.9926	0.0124	-0.7814	0.3787	-1.3482	0.0643	-2.8070	0.0001	-1.5407	0.0430	-2.9758	0.0001	-2.7435	0.0004	-2.1522	0.0028	-1.8004	0.0278	-1.5705	0.0243
AT2G30890	-0.7462	0.1875	-1.0724	0.0644	-1.0611	0.1039	-0.9680	0.1840	-0.0079	0.9995	-1.9553	0.0008	-0.7354	0.1760	-0.1474	0.9252	-1.9370	0.0003	-0.6834	0.4196	0.0855	0.9441	-0.6825	0.2453	-1.0238	0.1188	-2.1494	0.0000
AT2G31020	-1.6076	0.0014	-2.2690	0.0000	-1.0075	0.1145	-1.5010	0.0175	-1.2467	0.0357	-0.0249	0.9775	-2.0226	0.0000	-1.9120	0.0003	-1.3716	0.0090	-1.4486	0.0152	-1.8650	0.0008	-1.9019	0.0001	-2.3432	0.0000	-1.6655	0.0007
AT2G31360	1.9123	0.0000	1.3870	0.0001	1.3560	0.0003	1.1224	0.0120	2.0900	0.0000	1.1465	0.0042	1.9007	0.0000	2.0010	0.0000	1.5038	0.0000	1.6160	0.0000	-0.1540	0.8344	2.2240	0.0000	1.8520	0.0000	1.5679	0.0000
AT2G31380	-1.8739	0.0001	-1.2848	0.0131	-1.4017	0.0111	-1.0012	0.1350	-1.8637	0.0002	-1.5976	0.0030	-1.7346	0.0002	-1.5798	0.0030	-1.6437	0.0009	-0.5125	0.5672	0.5552	0.4587	-1.6478	0.0006	-2.2197	0.0000	-1.2996	0.0063
AT2G31585	-0.7828	0.1350	0.7307	0.2034	0.6054	0.3759	0.2834	0.7429	0.6241	0.3978	0.5290	0.3756	-0.1574	0.8057	-0.2101	0.8759	0.6990	0.1894	0.7253	0.3279	0.6818	0.3289	-0.5512	0.3323	0.3498	0.7120	2.1026	0.0000
AT2G31790	0.1078	0.9068	-1.4029	0.0411	-1.3700	0.0745	0.1321	0.9208	-0.4391	0.7230	-1.6455	0.0198	0.0355	0.9687	-0.3027	0.8591	-1.6589	0.0109	-0.5014	0.6959	0.3049	0.8018	0.5502	0.4768	-0.8905	0.3318	-2.4813	0.0000
AT2G31880	-1.2085	0.0031	-1.7494	0.0000	-0.9965	0.0388	-1.2412	0.0140	-0.6899	0.2166	-0.6100	0.2110	-2.0415	0.0000	-1.7773	0.0000	-1.4581	0.0004	-1.1537	0.0169	-0.2631	0.7198	-2.0441	0.0000	-1.6239	0.0001	-0.8829	0.0264
AT2G31900	-2.0943	0.0000	-1.3673	0.0034	-0.4372	0.5068	-0.8117	0.1907	-0.4430	0.5568	-1.3218	0.0104	-2.0950	0.0000	-0.9888	0.0725	-1.0213	0.0321	-0.5419	0.4674	-0.7505	0.2235	-2.1151	0.0000	-0.5099	0.4791	-1.4100	0.0019
AT2G31980	-2.5066	0.0000	0.2438	0.7529	-0.1062	0.9169	-0.8629	0.2624	0.1143	0.9337	0.0753	0.9276	-1.9932	0.0001	-0.7220	0.4028	-0.5971	0.3265	-0.9202	0.2292	-0.5029	0.5539	-2.0414	0.0001	-0.4576	0.6401	-0.4361	0.4330
AT2G32030	-1.6765	0.0100	-2.4869	0.0001	-1.5477	0.0415	-0.3102	0.7931	-1.4279	0.0675	-1.4636	0.0457	-1.8275	0.0032	-1.8014	0.0131	-2.3718	0.0003	-1.2432	0.1549	-0.0865	0.9548	-1.9601	0.0023	-1.7566	0.0140	-1.1694	0.0630
AT2G32160	-1.9696	0.0010	-2.2685	0.0001	-1.4624	0.0414	-1.7097	0.0253	-0.5605	0.6029	-1.4249	0.0380	-1.6326	0.0057	-1.4782	0.0404	-2.3953	0.0001	-1.5689	0.0335	-1.3458	0.0801	-2.1295	0.0003	-1.5930	0.0192	-2.2262	0.0001
AT2G32190	-2.1072	0.0007	-0.7446	0.3394	-1.0648	0.1982	-0.3234	0.7773	-1.4034	0.0658	-0.6969	0.3660	-1.7152	0.0048	-1.7005	0.0177	-1.1330	0.0945	-1.0173	0.2716	-1.6009	0.0354	-1.8123	0.0042	-2.1650	0.0011	-0.9176	0.1405
AT2G32200	-2.2942	0.0000	-1.7985	0.0021	-1.2036	0.0856	-1.1143	0.1504	-2.1910	0.0002	-1.3840	0.0327	-1.7964	0.0008	-2.1921	0.0003	-2.5965	0.0000	-2.0798	0.0011	-0.1099	0.9322	-2.2687	0.0000	-2.3518	0.0001	-1.8286	0.0012
AT2G32210	-2.3126	0.0002	-1.2141	0.0879	-1.4688	0.0524	-0.6486	0.5184	-1.9605	0.0040	-1.1392	0.1220	-1.8804	0.0019	-2.1192	0.0019	-1.7175	0.0088	-1.3051	0.1199	-0.1850	0.8938	-1.8433	0.0037	-2.6030	0.0001	-0.9822	0.1156
AT2G32280	1.5549	0.0041	1.1464	0.0603	0.6845	0.3920	0.9660	0.2166	1.6296	0.0057	0.3473	0.6494	2.1087	0.0000	1.4946	0.0153	0.7528	0.2281	1.1819	0.1000	0.9524	0.2134	1.9996	0.0001	1.5766	0.0082	0.6220	0.2831
AT2G32290	-0.6214	0.4819	-1.7329	0.0295	-0.8007	0.4602	-1.0213	0.3565	-0.2170	0.9030	-2.2365	0.0062	-0.2864	0.7636	-1.2810	0.2173	-2.2824	0.0023	-0.1303	0.9566	-0.0776	0.9661	-0.3568	0.7255	-0.8279	0.4915	-2.0521	0.0040
AT2G32300	0.4167	0.6378	0.6437	0.4769	2.0288	0.0069	0.3908	0.7552	-0.0845	0.9691	-0.8675	0.3269	-0.5254	0.5224	0.4537	0.7884	-0.1277	0.8973	-0.0496	0.9824	2.2222	0.0033	0.6814	0.4124	0.0443	0.9859	2.3398	0.0003
AT2G32530	-2.0837	0.0000	-1.2117	0.0342	-1.6993	0.0029	-1.6642	0.0099	-1.7380	0.0017	-2.4770	0.0000	-2.1392	0.0000	-2.3754	0.0000	-1.9132	0.0003	-0.9660	0.1924	-0.2894	0.7669	-1.2709	0.0195	-2.7184	0.0000	-1.7171	0.0008
AT2G32540	-2.3089	0.0000	-1.2474	0.0001	-0.9821	0.0052	-1.3787	0.0001	-1.4181	0.0000	-1.5872	0.0000	-2.0916	0.0000	-2.0612	0.0000	-1.8440	0.0000	-1.3835	0.0000	-0.3565	0.4657	-2.1806	0.0000	-1.8312	0.0000	-1.6061	0.0000
AT2G32640	0.0736	0.9113	-0.8160	0.1164	-0.3846	0.5799	-0.4173	0.5732	-0.3437	0.6918	-1.1992	0.0187	0.3372	0.5237	-0.5378	0.4999	-1.3471	0.0038	-0.8429	0.1838	0.1347	0.8922	0.2610	0.6652	-0.8326	0.1551	-2.2846	0.0000
AT2G32660	-1.3955	0.0144	-0.9303	0.1520	-0.1261	0.9026	-0.4858	0.5858	0.2762	0.8137	-0.5090	0.4710	-2.1231	0.0001	-1.0713	0.1459	-0.6283	0.3205	-0.1080	0.9504	-0.9082	0.2447	-1.2075	0.0383	0.3309	0.7669	1.2502	0.0149
AT2G32680	-2.2030	0.0021	-2.6810	0.0001	-1.6418	0.0579	-1.5190	0.1205	-0.4052	0.7917	-1.4709	0.0747	-2.2891	0.0009	-2.0218	0.0128	-2.5127	0.0005	-1.6379	0.0746	-1.1736	0.2410	-2.5381	0.0003	-1.9262	0.0161	-1.6203	0.0203
AT2G32990	-0.7890	0.3204	-1.5945	0.0362	-0.7244	0.4855	-0.6230	0.5879	-0.3604	0.8136	-1.8689	0.0171	-0.7077	0.3640	-0.9247	0.4245	-2.2454	0.0016	-0.6725	0.6136	0.7346	0.4993	-0.7747	0.3463	-0.4555	0.7487	-1.0149	0.1434
AT2G33020	-2.7473	0.0000	-0.5643	0.3623	-0.5814	0.4158	-0.5829	0.4444	-0.8488	0.2124	-1.0338	0.0798	-2.5748	0.0000	-0.9848	0.1306	-0.5368	0.3464	-0.8297	0.2547	-0.6651	0.3633	-2.6056	0.0000	-0.6506	0.3941	-0.4564	0.3781
AT2G33080	-1.6492	0.0298	-1.6542	0.0388	-1.5311	0.0927	-1.2779	0.2265	-0.9735	0.3849	-1.1107	NA	-2.0681	0.0038	-1.6996	0.0574	-2.1706	0.0035	-1.2837	0.2231	-1.5364	0.1059	-0.1819	0.8725	-0.7793	0.5301	-1.2468	0.0832
AT2G33160	0.1286	0.8998	0.4834	0.6142	0.1298	0.9233	0.3564	0.7805	0.1282	0.9456	0.7806	NA	0.0262	0.9794	-0.0251	0.9932	3.2297	0.0000	1.3185	0.1794	0.3205	0.8131	0.0000	1.0000	0.5066	0.7089	2.3936	0.0004
AT2G33175	-0.3798	0.6334	-0.3353	0.7119	-1.4553	0.0694	-0.7548	0.4456	0.7475	0.4372	-1.1226	0.1453	0.0432	0.9605	-1.5567	0.0462	-0.8652	0.2297	-0.5214	0.6818	-0.6501	0.5186	-1.1758	0.0881	-2.3912	0.0008	-2.2825	0.0005
AT2G33250	-1.4418	0.0005	-1.0571	0.0190	-1.2877	0.0057	-1.3176	0.0112	-1.3891	0.0017	-1.1457	0.0154	-1.3350	0.0009	-1.4141	0.0018	-1.7105	0.0000	-0.8707	0.1215	-0.3646	0.5994	-1.4290	0.0006	-1.4378	0.0012	-2.1591	0.0000
AT2G33330	0.6027	0.4034	-1.1949	0.0866	-0.5259	0.5794	-0.4299	0.6866	0.1547	0.9220	-1.0600	0.1445	0.5798	0.4058	0.6578	0.5688	-1.4083	0.0310	-0.0924	0.9645	0.7131	0.4485	0.5835	0.4372	-0.5994	0.5785	-2.0004	0.0008
AT2G33380	-2.0860	0.0003	1.1948	0.0654	0.0497	0.9686	0.0987	0.9396	-0.8606	0.3077	0.4072	0.5922	-2.4727	0.0000	-0.7650	0.4303	1.3148	0.0324	0.8978	0.3104	-1.0064	0.2092	-1.1013	0.0779	0.4040	0.7276	-0.1535	0.8177
AT2G33710	-1.1888	0.0098	-2.0363	0.0000	-1.0253	0.0648	-0.3657	0.6204	-2.3460	0.0000	-0.4580	0.4202	-1.5767	0.0002	-1.9864	0.0000	-1.2874	0.0073	-1.5248	0.0038	-1.3555	0.0123	-1.6227	0.0003	-2.1640	0.0000	-1.6021	0.0006
AT2G33760	1.6578	0.0129	1.4953	0.0393	0.8966	0.3482	0.6111	0.5825	1.1308	0.2212	0.4300	NA	1.6990	0.0070	0.7295	0.5620	1.0745	0.1485	1.1938	0.2113	1.0849	0.2522	2.0742	0.0013	1.6052	0.0381	0.7716	0.2743
AT2G33830	0.2186	0.8256	1.2945	0.1096	1.1473	0.2237	1.1969	0.2417	0.5667	0.6713	1.6052	0.0463	0.3819	0.6622	1.2062	0.2318	1.8081	0.0141	1.8245	0.0343	-1.0704	0.2876	0.4087	0.6660	1.6878	0.0398	2.4667	0.0003
AT2G34500	-1.7482	0.0088	-1.2320	0.0995	-1.0452	0.2356	1.3535	0.1299	-1.5997	0.0391	-0.3540	0.6918	-1.9256	0.0024	-1.4468	0.0776	-0.2343	0.7885	-0.9843	0.3322	-1.1170	0.2195	-2.0559	0.0017	-1.6391	0.0298	0.4219	0.5484
AT2G34510	2.8843	0.0000	0.2837	0.6027	0.5912	0.2758	0.7070	0.2114	0.8474	0.0843	0.2433	0.6457	2.1143	0.0000	0.8725	0.0772	0.0238	0.9693	0.0835	0.9478	1.8107	0.0000	2.2510	0.0000	0.3024	0.6950	-0.1642	0.7130
AT2G34530	-0.7219	0.2153	-0.4496	0.5190	-1.1376	0.0883	-0.5218	0.5412	0.4954	0.5869	-0.5860	0.3753	-0.7647	0.1642	-0.0717	0.9693	-0.2104	0.7619	-0.2889	0.8145	-1.1742	0.0896	-0.5148	0.4167	-0.5233	0.5720	-2.0299	0.0002
AT2G34640	0.2478	0.7160	-0.3536	0.6212	-0.8057	0.2486	-0.4912	0.5603	-0.0539	0.9737	-0.6661	0.2884	0.0415	0.9555	0.2930	0.8276	-0.8630	0.1257	-0.5320	0.5751	-0.1995	0.8502	0.5867	0.3337	-0.5396	0.5396	-2.1030	0.0000
AT2G34940	-2.4997	0.0002	-2.6757	0.0001	-1.6995	0.0314	-1.8921	0.0267	-0.6618	0.5705	-1.9851	0.0088	-2.3027	0.0003	-2.1604	0.0034	-3.4309	0.0000	-1.9376	0.0149	-1.9795	0.0119	-2.8538	0.0000	-1.9160	0.0100	-2.4306	0.0002
AT2G35290	2.2260	0.0001	0.9083	0.1918	0.6430	0.4604	0.8788	0.3080	1.6858	0.0076	0.6251	0.3996	2.3691	0.0000	1.1865	0.1143	0.6823	0.3119	0.6683	0.5199	2.9427	0.0000	1.7390	0.0025	0.8849	0.2898	1.3692	0.0161
AT2G35300	-0.3459	0.7133	2.5717	0.0003	0.7858	0.4580	-0.1950	0.8942	-0.1592	0.9318	0.1410	NA	-0.4250	0.6274	0.4169	0.8210	0.5937	0.4872	0.4286	0.7937	2.4384	0.0023	1.4541	0.0584	0.4775	0.7410	0.2448	0.7604
AT2G35637	0.3105	0.7523	0.8588	0.3476	-0.4892	0.6692	0.2010	0.8933	-0.2572	0.8805	0.5494	NA	0.0673	0.9488	0.1847	0.9375	0.3354	NA	0.9416	0.4360	2.2857	0.0054	0.1670	0.8820	0.0184	0.9969	0.4351	NA
AT2G35940	-2.0348	0.0000	-1.0125	0.0173	-1.1207	0.0131	-1.2211	0.0130	-0.9311	0.0518	-1.0801	0.0159	-2.1942	0.0000	-1.1927	0.0073	-0.9975	0.0170	-0.1837	0.8512	-0.2410	0.7409	-1.9336	0.0000	-1.1307	0.0099	-1.3506	0.0005
AT2G35945	-2.3147	0.0013	-1.5621	0.0521	-1.4409	0.1179	-1.9798	0.0322	-1.5392	0.0891	-1.2685	NA	-2.0773	0.0034	-2.0006	0.0154	-1.8075	0.0170	-1.4593	0.1386	-1.3167	0.1846	-2.5120	0.0004	-1.9014	0.0188	-1.8378	0.0085
AT2G35960	-1.2121	0.0368	-1.4116	0.0181	-1.1038	0.1192	-1.0712	0.1715	-0.8078	0.3248	-2.0755	0.0007	-0.8175	0.1656	-0.8324	0.3349	-2.2069	0.0001	-0.7633	0.3982	0.1760	0.8845	-0.8862	0.1506	0.0501	0.9777	-0.8656	0.1182
AT2G35980	-3.4707	0.0000	-2.4997	0.0000	-1.7096	0.0055	-1.9240	0.0037	-2.7698	0.0000	-1.3074	0.0385	-2.7313	0.0000	-3.0219	0.0000	-2.5050	0.0000	-2.4684	0.0000	-3.0779	0.0000	-2.9807	0.0000	-2.8150	0.0000	-1.4169	0.0090
AT2G36080	-1.7199	0.0001	0.3052	0.6286	0.1322	0.8740	0.0469	0.9646	-0.5177	0.4844	0.1466	0.8259	-2.1630	0.0000	-0.1300	0.9263	0.2903	0.6079	-0.3336	0.7295	-1.3942	0.0081	-1.4837	0.0011	0.3294	0.7126	0.2524	0.6116
AT2G36100	-0.4120	0.6394	0.3809	0.6949	1.5420	0.0542	-0.2789	0.8312	0.1526	0.9320	-1.0765	0.2040	-1.1177	0.1195	0.0281	0.9924	-0.6451	0.4350	-0.0248	0.9911	2.1590	0.0037	-0.0409	0.9732	0.3601	0.8103	2.2730	0.0004
AT2G36220	-1.8505	0.0015	-0.1854	0.8366	0.3540	0.7077	-0.1514	0.8996	-0.1596	0.9126	0.3578	0.6436	-2.3752	0.0000	-0.5084	0.6775	-0.0727	0.9303	0.0157	0.9936	0.1383	0.9169	-1.5352	0.0103	0.1822	0.8996	1.2304	0.0293
AT2G36270	-0.4088	0.5768	1.7486	0.0029	0.9320	0.2284	0.9206	0.2753	1.5520	0.0166	0.8997	0.1923	-0.2826	0.6992	1.4273	0.0359	1.9605	0.0007	1.6096	0.0180	0.2374	0.8420	0.3530	0.6461	2.2011	0.0002	1.8559	0.0008
AT2G36630	-2.3652	0.0000	-1.5112	0.0001	-1.5247	0.0002	-1.1793	0.0171	-1.9882	0.0000	-1.5987	0.0002	-2.6127	0.0000	-2.1463	0.0000	-1.6934	0.0000	-0.8735	0.0892	-0.7965	0.1269	-2.1797	0.0000	-1.6538	0.0000	-2.3382	0.0000
AT2G36640	-0.2949	0.7604	2.9850	0.0000	0.7410	0.4880	0.5036	0.6846	-0.2341	0.8915	0.3986	NA	-0.3750	0.6737	-0.1200	0.9635	1.1742	0.1345	0.7260	0.5865	2.1097	0.0116	0.5502	0.5464	0.4668	0.7487	0.5334	0.4786
AT2G36690	-1.7332	0.0019	-2.0209	0.0003	-0.2499	0.7902	-0.9482	0.2339	-1.2435	0.0674	-2.1601	0.0005	-1.6987	0.0014	-1.4325	0.0277	-1.5134	0.0093	-2.1850	0.0004	-1.5103	0.0239	-1.3475	0.0190	-0.2875	0.8123	-1.7621	0.0014
AT2G36780	-0.5530	0.4408	-0.0184	0.9866	-0.1313	0.9097	0.4387	0.6684	-0.3290	0.8010	-0.1634	0.8564	-0.9802	0.1174	-0.1695	0.9275	0.7144	0.2961	0.5626	0.6281	-0.2359	0.8480	-0.6819	0.3370	-2.1663	0.0007	-0.8279	0.1722
AT2G36800	-1.5164	0.0120	-1.3022	0.0453	-1.1406	0.1322	-0.1343	0.9135	-0.8882	0.2981	-1.7028	0.0102	-1.4162	0.0155	-1.5564	0.0233	-1.5645	0.0110	-0.3213	0.8183	0.1627	0.9026	-1.0138	0.1165	-2.0317	0.0011	-1.9077	0.0009
AT2G36885	-0.1926	0.7661	-0.1407	0.8488	-0.2748	0.7262	-0.1607	0.8665	-0.1383	0.9055	-1.0299	0.0611	0.4021	0.4629	0.1469	0.9192	-1.0668	0.0359	-0.9881	0.1217	-0.2297	0.8042	-0.6942	0.1943	-0.9428	0.1178	-2.7603	0.0000
AT2G36970	-0.6386	0.4689	-1.9057	0.0144	-0.9354	0.3684	-0.2493	0.8659	0.0723	0.9758	-1.6250	0.0528	-0.1483	0.8817	-0.0905	0.9735	-2.0949	0.0055	0.0642	0.9788	0.5301	0.6811	-0.2725	0.7961	-1.5495	0.0833	-1.4082	0.0496
AT2G37040	-1.1079	0.0175	-1.2440	0.0093	-1.0064	0.0699	-0.6175	0.3720	-0.9477	0.0964	-1.5093	0.0025	-0.9931	0.0289	-1.1711	0.0275	-1.6412	0.0003	-0.6675	0.3482	-0.4308	0.5551	-0.7829	0.1146	-1.3796	0.0050	-2.0761	0.0000
AT2G37130	-0.0251	0.9665	-0.2293	0.6683	0.3978	0.4809	-0.1543	0.8316	0.8084	0.0849	0.8466	0.0501	-0.4828	0.2369	0.5911	0.3004	0.5283	0.2107	1.0613	0.0177	0.3473	0.5711	0.1530	0.7757	1.5665	0.0000	2.6290	0.0000
AT2G37240	-0.4032	0.5691	-1.2083	0.0560	-0.7906	0.3194	-1.1583	0.1383	-0.2775	0.8273	-1.6107	0.0117	-0.2125	0.7745	-0.7533	0.4267	-1.8111	0.0018	-0.4843	0.6687	0.0013	1.0000	-0.2757	0.7268	-0.9967	0.1861	-2.3077	0.0000
AT2G37430	-2.1517	0.0004	-3.0976	0.0000	-2.1378	0.0012	-0.9514	0.2840	-1.7994	0.0076	-1.3096	0.0623	-2.7425	0.0000	-2.1775	0.0009	-2.0587	0.0011	-1.8115	0.0117	-0.6058	0.5277	-1.8411	0.0027	-2.7130	0.0000	-2.0551	0.0007
AT2G37460	-0.4134	0.5891	-2.1595	0.0004	-1.6377	0.0200	-0.7896	0.4018	0.0259	0.9917	-1.6992	0.0135	-0.8406	0.1983	-0.1957	0.9163	-2.0510	0.0010	-0.2948	0.8432	0.2314	0.8537	-0.5958	0.4225	-0.6501	0.5301	-1.8101	0.0024
AT2G37580	1.0230	0.1320	0.7924	0.3132	0.8133	0.3646	0.2813	0.8178	1.3974	0.0687	-0.0315	0.9777	1.5837	0.0096	0.6655	0.5805	0.9184	0.1911	0.3033	0.8461	0.0230	0.9909	1.9976	0.0013	2.1222	0.0012	1.8621	0.0022
AT2G37710	-1.1258	0.0761	-1.8287	0.0030	-0.8100	0.3353	-1.2881	0.1141	-0.3834	0.7532	-1.7982	0.0071	-1.2785	0.0335	-1.1854	0.1341	-2.1391	0.0004	-0.3882	0.7708	-0.1825	0.8895	-1.2352	0.0526	-1.0164	0.2061	-0.7820	0.1940
AT2G37970	-1.5266	0.0122	-1.7161	0.0058	-1.9203	0.0032	-0.5170	0.6014	-2.2733	0.0002	-1.7137	0.0105	-2.3927	0.0000	-2.0074	0.0018	-1.8729	0.0022	-1.0092	0.2469	-0.6368	0.4935	-1.5549	0.0113	-1.8853	0.0033	-1.5005	0.0100
AT2G37980	-2.1873	0.0000	-1.2974	0.0012	-0.7551	0.1259	-0.6229	0.2598	-0.8992	0.0553	-0.0996	0.8620	-2.0076	0.0000	-1.0965	0.0125	-1.0731	0.0087	-1.0650	0.0225	-1.4781	0.0008	-2.1192	0.0000	-1.2598	0.0034	-0.5608	0.1508
AT2G38240	-2.2120	0.0004	-3.1309	0.0000	-1.6633	0.0246	0.3361	0.7736	-0.7815	0.4344	-1.3312	0.0699	-1.6837	0.0070	-1.7060	0.0205	-0.5463	0.4716	-0.0678	0.9751	-2.2647	0.0013	-2.3253	0.0002	-2.8384	0.0000	-1.1336	0.0710
AT2G38310	1.5232	0.0123	-2.3194	0.0001	-1.1361	0.1383	-0.5166	0.6021	-0.4919	0.6601	-1.5307	0.0231	1.0429	0.0895	-0.5083	0.6909	-2.3320	0.0001	-1.1573	0.1577	0.8529	0.3236	1.0593	0.1016	-2.1064	0.0007	-1.1179	0.0559
AT2G38380	2.8682	0.0000	0.4107	0.6089	-0.0103	0.9940	0.8976	0.2950	1.0136	0.1968	1.7413	0.0069	1.3408	0.0194	1.2967	0.0709	1.1209	0.0726	2.2477	0.0003	1.3719	0.0564	1.9438	0.0007	1.4168	0.0343	1.5159	0.0072
AT2G38390	2.5664	0.0000	0.2931	0.7208	0.0662	0.9548	0.5305	0.5619	1.1459	0.1032	1.3188	0.0373	0.8834	0.1267	1.4844	0.0202	1.1790	0.0472	2.5840	0.0000	1.7754	0.0046	1.5097	0.0075	1.9012	0.0011	2.5252	0.0000
AT2G38465	0.1954	0.8262	1.2910	0.0661	1.3484	0.0821	0.7548	0.4457	2.1168	0.0012	1.7462	0.0131	0.8006	0.2364	1.5887	0.0312	1.8858	0.0033	2.3809	0.0004	-0.3342	0.7838	0.9308	0.1859	1.7226	0.0134	2.3638	0.0001
AT2G38470	-2.6583	0.0000	-2.1480	0.0000	-1.4753	0.0049	-1.2676	0.0376	-1.9073	0.0001	-1.0180	0.0622	-2.8903	0.0000	-1.9448	0.0001	-1.9260	0.0000	-1.5039	0.0058	-0.0583	0.9602	-2.9423	0.0000	-1.8445	0.0001	-1.6212	0.0004
AT2G38490	-1.7604	0.0022	-0.1612	0.8481	-0.0991	0.9260	-0.7116	0.3976	-0.5302	0.5850	-0.6816	0.3227	-2.2075	0.0000	-0.7740	0.3811	-0.1837	0.8025	-0.5277	0.6166	-0.5157	0.5634	-2.1936	0.0001	-0.3776	0.7352	-0.3543	0.5557
AT2G38780	-0.8314	0.0644	-0.7538	0.1268	-0.5225	0.3929	-0.9682	0.0905	-0.3094	0.7106	-1.0369	0.0325	-0.7939	0.0677	-0.6802	0.2838	-1.2570	0.0042	-0.9410	0.0897	-0.8652	0.1236	-0.6367	0.1813	-0.8895	0.0898	-2.1501	0.0000
AT2G38860	-1.2109	0.0668	-2.5085	0.0000	-1.3081	0.0930	-0.8688	0.3578	-0.1383	0.9325	-1.7005	0.0155	-1.3998	0.0248	-1.0369	0.2549	-2.0687	0.0011	-0.8688	0.3872	-0.4740	0.6603	-1.2217	0.0684	-1.5529	0.0306	-1.0168	0.0999
AT2G39030	-1.1684	0.0950	-2.2941	0.0006	-1.0763	0.2201	1.0976	0.2448	-1.4265	0.0802	-0.4992	0.5587	-1.7143	0.0076	-1.9031	0.0106	0.0146	0.9897	0.5739	0.6544	-1.6535	0.0407	-0.9101	0.2190	-0.1550	0.9264	0.0551	0.9441
AT2G39250	-1.6497	0.0015	-0.3927	0.5785	-0.7056	0.3337	-0.4179	0.6325	-1.1963	0.0577	-1.0915	0.0689	-2.2265	0.0000	-0.9580	0.1811	-0.9320	0.0995	-0.2021	0.8859	0.3660	0.6909	-1.0273	0.0651	-0.3935	0.6976	-1.5510	0.0023
AT2G39350	-1.9426	0.0000	0.5600	0.2326	0.6448	0.2119	-0.4555	0.4575	-0.6766	0.2021	-0.2620	0.6090	-1.9389	0.0000	-1.0909	0.0141	-0.0052	0.9938	-0.0762	0.9514	-1.4518	0.0007	-2.1142	0.0000	-0.1425	0.8777	-0.4783	0.2254
AT2G39400	-1.1494	0.0087	-1.1172	0.0148	-0.0636	0.9424	-0.5687	0.3897	-0.5350	0.4274	0.5702	0.2741	-1.1224	0.0079	-0.3725	0.6837	-0.3880	0.4463	-0.6119	0.3703	-2.0106	0.0000	-1.2846	0.0031	-0.4915	0.4851	-0.1329	0.7860
AT2G39518	-3.0022	0.0000	-2.3050	0.0000	-1.4114	0.0034	-1.1310	0.0459	-1.8299	0.0000	-1.2834	0.0098	-2.8835	0.0000	-2.2059	0.0000	-1.5238	0.0007	-1.7139	0.0004	-2.5645	0.0000	-2.7984	0.0000	-2.5378	0.0000	-0.7700	0.0756
AT2G39530	-3.3674	0.0000	-2.3697	0.0003	-2.1572	0.0023	-1.7426	0.0330	-2.4276	0.0003	-2.5897	NA	-3.8124	0.0000	-3.9609	0.0000	-3.3072	0.0000	-2.6731	0.0002	-2.7590	0.0001	-4.1383	0.0000	-3.3394	0.0000	-2.6257	0.0000
AT2G39560	-0.0901	0.9045	-0.6710	0.2970	-1.1689	0.0703	-0.3998	0.6459	-1.2124	0.0578	-1.0545	0.0862	-0.0964	0.8890	-0.6475	0.4678	-1.3023	0.0201	-0.1674	0.9088	0.4545	0.5864	-0.1215	0.8773	-0.9927	0.1433	-2.0936	0.0001
AT2G39855	-2.0287	0.0031	0.3819	0.6902	-0.7203	0.4748	0.2004	0.8834	-1.4471	0.0891	-0.3974	0.6614	-2.9928	0.0000	-0.6765	0.6081	0.3304	0.7027	0.0193	0.9933	-1.9629	0.0151	-1.4235	0.0482	-0.0325	0.9892	-0.7186	0.3007
AT2G39900	1.9110	0.0000	1.3117	0.0012	1.1258	0.0133	1.1574	0.0217	1.6876	0.0000	0.9637	0.0348	2.1642	0.0000	1.5020	0.0003	1.4481	0.0003	1.3373	0.0031	-0.0603	0.9505	2.0196	0.0000	1.6481	0.0000	1.5725	0.0000
AT2G39920	0.9331	0.1648	2.0939	0.0006	2.5337	0.0000	1.8075	0.0183	3.2545	0.0000	2.4112	0.0003	1.8257	0.0020	2.5128	0.0001	2.6307	0.0000	2.5242	0.0001	-0.1967	0.8845	1.6431	0.0088	3.0159	0.0000	2.6022	0.0000
AT2G39980	-1.8031	0.0000	-0.6102	0.2507	-0.0575	0.9487	-0.6767	0.2951	-0.6038	0.3565	-0.1581	0.7999	-1.8940	0.0000	-0.8825	0.1229	-0.5758	0.2411	-0.7132	0.2716	-1.4317	0.0037	-2.0024	0.0000	0.0042	0.9999	-0.0770	0.8812
AT2G40000	-1.7862	0.0003	-0.5917	0.3448	-0.4066	0.5985	-0.3437	0.6918	-1.3310	0.0191	0.4092	0.5254	-2.5809	0.0000	-0.8895	0.2034	-0.0939	0.8932	-0.5324	0.5591	-0.2526	0.7924	-2.1431	0.0000	-0.4613	0.6069	0.0283	0.9628
AT2G40080	1.5575	0.0249	2.2017	0.0012	2.1278	0.0038	1.8684	0.0285	3.4917	0.0000	1.8977	0.0121	2.2798	0.0003	2.8551	0.0000	3.0561	0.0000	3.5613	0.0000	1.3706	0.1237	3.2325	0.0000	3.4838	0.0000	2.8432	0.0000
AT2G40113	-1.8274	0.0097	-0.5667	0.5322	-0.2021	0.8700	0.0162	0.9932	-0.5112	0.6989	-1.2190	0.1354	-2.1061	0.0016	-0.4364	0.7922	0.2493	0.7814	-0.0914	0.9672	-0.4085	0.7431	-1.0140	0.1844	-1.4314	0.0966	0.9358	0.1625
AT2G40150	-0.1939	0.7270	-0.8126	0.0905	-0.7638	0.1671	-0.3657	0.5986	-0.4399	0.5356	-0.6428	0.2045	-0.2543	0.6149	-0.4688	0.5419	-1.1819	0.0076	-0.4625	0.5418	0.1753	0.8355	-0.3110	0.5609	-0.6055	0.3285	-2.0675	0.0000
AT2G40180	-1.2318	0.0504	-1.3819	0.0391	-0.6816	0.4364	-0.2457	0.8262	-1.0038	0.2291	-0.3644	0.6477	-1.7983	0.0020	-0.7408	0.4706	-1.0180	0.1247	-1.0005	0.2605	-0.9947	0.2400	-1.5670	0.0122	-0.5238	0.6356	-2.0654	0.0009
AT2G40520	-0.2636	0.6317	-1.5469	0.0003	-1.4449	0.0020	-1.1676	0.0338	-0.8395	0.1338	-1.3746	0.0043	-0.8986	0.0377	-1.4507	0.0019	-1.8504	0.0000	-1.0558	0.0509	-0.5289	0.4164	-0.3678	0.4919	-1.4070	0.0024	-2.3469	0.0000
AT2G40610	-3.1201	0.0000	-1.4324	0.0125	-0.2021	0.8326	-1.2498	0.0841	-0.2858	0.8092	-1.0996	0.0786	-2.5272	0.0000	-0.6427	0.5042	-1.8019	0.0010	-1.4683	0.0238	0.1872	0.8698	-1.8354	0.0006	-0.2795	0.8156	0.1966	0.7469
AT2G40740	-2.0740	0.0008	-2.3606	0.0002	-1.6309	0.0232	-1.7326	0.0261	-0.7882	0.3978	-1.3560	0.0567	-2.2602	0.0001	-1.9206	0.0050	-1.6786	0.0096	-1.5064	0.0509	-1.3644	0.0829	-1.9776	0.0014	-1.7756	0.0105	-0.8466	0.1686
AT2G40750	-1.1733	0.1412	-2.3847	0.0014	-1.0961	0.2768	-1.0128	0.3627	0.0899	0.9679	-2.2182	0.0068	-0.7945	0.3303	-0.9493	0.4429	-2.2326	0.0030	-0.7519	0.5810	-0.5405	0.6735	-0.9533	0.2581	-1.4811	0.1053	-1.1746	0.1062
AT2G41070	-2.2234	0.0000	-0.0107	0.9864	-0.4876	0.3150	-0.6765	0.1640	-0.1928	0.7963	-0.4849	0.2509	-2.1849	0.0000	-0.5311	0.3167	-0.2350	0.5832	-0.4374	0.4537	-0.5496	0.2654	-2.1909	0.0000	-0.7863	0.0620	-1.0989	0.0017
AT2G41090	-0.4836	0.5916	-2.1632	0.0035	-1.1123	0.2544	-0.7940	0.4875	-0.0761	0.9732	-1.5714	0.0565	-0.5763	0.4926	-0.6506	0.6636	-2.2549	0.0021	-0.2427	0.9054	0.1711	0.9169	-0.6774	0.4417	-1.1285	0.2593	-1.6216	0.0206
AT2G41100	-1.1004	0.0346	-2.0061	0.0000	-1.1257	0.0651	-1.2053	0.0696	-1.1686	0.0527	-0.5118	0.4176	-2.1640	0.0000	-1.4951	0.0075	-1.4468	0.0050	-1.1597	0.0701	-0.4530	0.5798	-1.7865	0.0003	-1.9512	0.0001	-1.6274	0.0008
AT2G41105	-1.3704	0.0788	-1.5509	0.0570	-1.8898	0.0257	-2.0749	0.0252	-2.0686	0.0104	-1.1161	NA	-2.2753	0.0013	-2.0724	0.0123	-1.8736	NA	-2.2408	0.0075	-2.0059	0.0201	-1.8074	0.0174	-2.1387	0.0067	-2.2353	NA
AT2G41120	-0.7888	0.0492	-1.2071	0.0025	-0.9370	0.0418	-0.9062	0.0765	-0.9323	0.0425	-1.6899	0.0001	-0.6285	0.1097	-1.6258	0.0001	-1.8510	0.0000	-1.1894	0.0081	-0.1578	0.8382	-1.1801	0.0023	-1.1344	0.0083	-2.0173	0.0000
AT2G41150	-1.9412	0.0000	0.3357	0.4829	0.0635	0.9311	-0.2865	0.6460	-0.6373	0.2139	0.1383	0.7930	-2.1633	0.0000	-0.6268	0.2425	0.0982	0.8468	-0.1539	0.8744	-0.1588	0.8322	-1.8732	0.0000	-0.5164	0.3577	-0.6312	0.0939
AT2G41170	-1.6218	0.0000	-0.3529	0.4697	0.2046	0.7429	-0.9689	0.0460	-0.7064	0.1523	-0.2505	0.6111	-2.0144	0.0000	-0.8448	0.0688	-0.8782	0.0271	-0.7618	0.1324	-0.5686	0.2889	-2.2535	0.0000	-0.2704	0.7139	-1.5241	0.0000
AT2G41190	-0.0044	0.9978	1.7442	0.0093	1.1030	0.1906	0.6993	0.4905	0.1723	0.9150	0.8390	0.2783	-0.2939	0.7165	0.6086	0.6358	1.5033	0.0254	1.1404	0.2102	0.1433	0.9210	2.0081	0.0016	1.3180	0.0931	1.6569	0.0075
AT2G41230	-2.4208	0.0003	-1.3557	0.0737	-0.8999	0.3414	-1.4755	0.1050	0.8566	0.4051	-2.2363	0.0030	-1.9108	0.0035	-0.1774	0.9329	-2.7976	0.0000	-2.1084	0.0071	-2.0758	0.0078	-1.7375	0.0124	-1.1883	0.1829	-1.7155	0.0097
AT2G41240	3.6543	0.0000	0.3066	0.7812	0.2535	0.8460	0.4584	0.7241	-0.1342	0.9469	-0.2113	0.8464	3.3600	0.0000	0.4087	0.8323	0.3062	0.7515	-0.3174	0.8669	-0.1176	0.9460	6.0993	0.0000	0.2223	0.9039	-0.1583	0.8526
AT2G41250	-2.5754	0.0000	-2.1965	0.0000	-2.3154	0.0000	-1.6615	0.0005	-2.2950	0.0000	-1.7396	0.0001	-3.0152	0.0000	-2.0322	0.0000	-2.1335	0.0000	-0.9819	0.0635	-0.1337	0.8825	-2.3533	0.0000	-2.5990	0.0000	-2.1250	0.0000
AT2G41260	-0.4885	0.5941	1.9866	0.0101	0.8082	0.4549	0.8988	0.4324	0.3682	0.8201	1.0304	0.2427	-0.5835	0.4948	0.1248	0.9629	1.9616	0.0099	0.9921	0.4052	2.3784	0.0040	-0.4059	0.6823	-0.3727	0.8178	1.6176	0.0234
AT2G41380	-1.3410	0.0414	-1.4515	0.0353	-1.2132	0.1343	-0.1779	0.8905	0.2297	0.8769	-1.8685	0.0084	-1.3368	0.0346	-1.0032	0.2859	-2.1210	0.0010	-0.7518	0.4900	-0.9509	0.2916	-1.3382	0.0451	-1.0329	0.2303	-0.8646	0.1709
AT2G41480	-0.6196	0.4482	-1.5434	0.0435	-1.2884	0.1510	-1.1025	0.2746	-0.4280	0.7611	-2.4751	0.0014	-1.0576	0.1400	-1.4106	0.1175	-2.2993	0.0014	-1.9883	0.0164	0.0146	0.9972	-1.1804	0.1166	-1.8409	0.0200	-1.5018	0.0279
AT2G41680	-0.1913	0.6774	-0.4312	0.3283	-0.4099	0.4257	-0.5226	0.3224	-0.6397	0.1763	-0.7022	0.0863	-0.2193	0.6092	-0.1695	0.8608	-0.7220	0.0564	-0.8936	0.0401	-0.4209	0.4331	-0.2821	0.5267	-0.7393	0.0879	-2.0313	0.0000
AT2G41880	-1.4961	0.0010	-1.6991	0.0002	-1.2066	0.0227	-0.7584	0.2548	-1.0318	0.0642	-0.6882	0.2085	-2.0644	0.0000	-1.0937	0.0477	-1.2819	0.0068	-0.7610	0.2592	-0.4575	0.5288	-1.4651	0.0013	-1.3528	0.0068	-1.4607	0.0010
AT2G42005	1.8558	0.0069	1.1489	0.1628	0.3487	0.7751	0.5002	0.6800	0.5269	0.7057	0.0102	NA	2.4313	0.0001	0.6249	0.6709	0.5252	0.5477	0.1173	0.9611	1.3862	0.1345	1.3136	0.0754	-0.4083	0.7972	-0.3106	0.7054
AT2G42350	-1.5107	0.0073	-1.6900	0.0037	-0.9480	0.1952	-0.8500	0.2920	-0.5711	0.5364	-1.0224	0.1169	-2.0443	0.0001	-1.5853	0.0121	-1.8154	0.0018	-1.1408	0.1235	-0.3312	0.7396	-1.6924	0.0025	-1.3432	0.0410	0.2142	0.7249
AT2G42530	1.4842	0.0206	2.2475	0.0003	1.8664	0.0071	0.5106	0.6255	2.9903	0.0000	1.3588	0.0573	1.5199	0.0136	2.8218	0.0000	2.6755	0.0000	2.8500	0.0000	-0.1985	0.8839	2.5074	0.0000	5.2206	0.0000	3.2539	0.0000
AT2G42540	-0.6187	0.3974	0.2600	0.7816	0.0067	0.9965	-1.3770	0.1024	0.5696	0.6114	-0.9431	0.2061	-0.4862	0.5064	0.4068	0.7896	0.4744	0.5290	0.7396	0.4939	-1.1522	0.1738	1.5941	0.0132	3.7772	0.0000	1.5014	0.0134
AT2G42560	0.0853	0.9375	2.5798	0.0003	0.6436	0.5574	0.7968	0.4838	0.4254	0.7764	0.7235	0.4231	-0.1133	0.9094	0.7389	0.5926	2.1113	0.0041	1.2043	0.2525	1.1618	0.2476	0.6455	0.4651	-0.0497	0.9837	0.7152	0.3295
AT2G42950	-1.0898	0.0015	-1.2985	0.0002	-1.2307	0.0009	-0.9378	0.0322	-0.8100	0.0504	-1.4686	0.0001	-1.5084	0.0000	-1.3798	0.0001	-1.2630	0.0003	-0.8811	0.0375	-0.7959	0.0670	-1.2473	0.0002	-1.1814	0.0014	-2.0793	0.0000
AT2G42975	-1.4122	0.0000	-0.6491	0.0151	-0.8305	0.0023	-0.9669	0.0008	-1.2823	0.0000	-1.3523	0.0000	-1.4171	0.0000	-1.8171	0.0000	-1.7446	0.0000	-1.2333	0.0000	-0.3272	0.3771	-1.4555	0.0000	-0.9154	0.0005	-2.2042	0.0000
AT2G43000	-1.6033	0.0175	-2.1131	0.0017	-1.7894	0.0181	-0.7841	0.4462	-0.6264	0.5878	-1.0641	0.1726	-1.8569	0.0037	-1.7805	0.0188	-2.3570	0.0004	-1.1567	0.2203	-0.6857	0.5051	-1.8642	0.0053	-1.2148	0.1543	-0.7772	0.2430
AT2G43050	-1.2778	0.0261	-0.6391	0.3607	0.1985	0.8390	-1.0758	0.1671	-0.1273	0.9291	-1.1117	0.0858	-2.3373	0.0000	-0.9238	0.2518	-0.7762	0.2108	-0.4905	0.6521	1.5785	0.0142	-1.6176	0.0041	-0.0351	0.9857	2.1541	0.0000
AT2G43060	-2.0604	0.0000	-1.3206	0.0158	-0.2417	0.7824	-1.3941	0.0330	0.0081	0.9994	-0.6329	0.3129	-1.9125	0.0001	-0.3573	0.7689	-1.3735	0.0101	-0.7119	0.3886	-0.4231	0.6233	-1.9075	0.0001	-0.4428	0.6409	-0.0445	0.9429
AT2G43140	-0.8838	0.2349	-2.6696	0.0001	-1.5872	0.0472	-2.0280	0.0149	0.0175	0.9960	-1.6856	0.0262	-1.0198	0.1465	-0.4080	0.8092	-2.5102	0.0002	-0.7141	0.5580	-0.1051	0.9467	-0.7248	0.3614	-2.0187	0.0056	-1.3265	0.0429
AT2G43535	1.4386	0.0028	1.1183	0.0331	0.8985	0.1505	1.0035	0.1268	1.3703	0.0100	1.2120	0.0262	1.1200	0.0186	1.1651	0.0435	1.5765	0.0012	2.2290	0.0000	0.5067	0.5053	1.5530	0.0011	1.9268	0.0001	1.3871	0.0030
AT2G43550	1.7279	0.0000	0.0215	0.9769	0.0818	0.9208	0.9626	0.0834	0.5328	0.4108	-0.5365	0.3044	1.6283	0.0000	0.8260	0.1334	0.4585	0.3437	0.2453	0.7993	0.4725	0.4639	2.3366	0.0000	1.3883	0.0016	-0.0318	0.9503
AT2G43570	-1.4634	0.0331	-1.5914	0.0263	-1.9648	0.0080	-1.1017	0.2523	-1.2305	0.1588	-1.3154	0.0862	-1.3919	0.0363	-2.4678	0.0004	-1.4459	0.0400	-1.5655	0.0620	-1.5361	0.0643	-0.6232	0.4414	-1.7921	0.0153	-0.3346	0.6458
AT2G43620	-2.2108	0.0001	-0.5735	0.4377	-0.3738	0.6857	-0.8135	0.3454	-1.2928	0.0642	-1.0429	0.1204	-2.3283	0.0000	-1.2830	0.0718	-0.4682	0.4941	-0.7699	0.4107	-1.8253	0.0051	-1.4907	0.0116	-0.1973	0.8867	-0.0086	0.9911
AT2G44290	-0.4340	0.6025	-2.2047	0.0010	-1.3719	0.0971	-1.5274	0.0841	-0.2892	0.8485	-2.0807	0.0046	-0.6131	0.4149	-1.4502	0.0805	-2.8088	0.0000	-1.3207	0.1414	0.2630	0.8448	-0.7618	0.3271	-1.3982	0.0834	-0.7768	0.2461
AT2G44370	-1.2666	0.0441	-2.9158	0.0000	-1.4101	0.0528	-1.6574	0.0313	-1.4805	0.0381	-1.7168	0.0120	-1.5492	0.0087	-1.7941	0.0081	-1.8692	0.0027	-1.6405	0.0242	-1.7289	0.0154	-1.3078	0.0399	-2.2150	0.0005	-1.3806	0.0199
AT2G44380	-1.8438	0.0000	-2.2126	0.0000	-1.2741	0.0118	-2.3532	0.0000	-1.2470	0.0121	-1.6072	0.0013	-2.3157	0.0000	-2.5581	0.0000	-2.5689	0.0000	-2.0212	0.0000	-2.0885	0.0000	-2.0054	0.0000	-1.3858	0.0044	-0.8818	0.0438
AT2G44840	-0.7743	0.3046	0.0674	0.9527	-0.7603	0.4352	0.8492	0.4006	-1.0944	0.2397	-0.6169	0.4689	-0.8151	0.2555	-1.1154	0.2486	-0.5610	0.4798	-0.1938	0.9192	2.9257	0.0000	-0.9510	0.2036	-0.9801	0.3118	0.0805	0.9189
AT2G44990	-0.0086	0.9946	1.5557	0.0277	2.8057	0.0000	0.6588	0.5417	1.5026	0.0574	1.2584	0.1033	0.6032	0.4169	1.2622	0.1486	0.9218	0.2167	0.9585	0.3583	0.5245	0.6512	-0.0695	0.9526	1.5558	0.0427	2.8275	0.0000
AT2G45220	-1.3094	0.0302	-2.1763	0.0002	-1.8350	0.0041	-0.8557	0.3270	-1.7835	0.0047	-0.5413	0.4670	-2.0799	0.0002	-2.2099	0.0003	-0.4611	0.5102	-0.6371	0.5456	-2.8261	0.0000	-1.4602	0.0154	-2.1561	0.0003	-0.1928	0.7700
AT2G45360	-0.2653	0.7812	0.8843	0.2988	0.4834	0.6604	1.3757	0.1419	0.1431	0.9384	2.1174	0.0051	0.1344	0.8854	0.8455	0.4788	2.0366	0.0035	1.7575	0.0346	-0.5567	0.6448	0.4635	0.6070	0.4492	0.7533	1.4545	0.0299
AT2G45430	0.4588	0.6220	2.0406	0.0069	1.6519	0.0603	0.5926	0.6284	0.7425	0.5547	0.4688	NA	0.1728	0.8623	1.9706	0.0170	1.2521	0.1154	1.7789	0.0488	2.3702	0.0035	0.3105	0.7653	0.8212	0.4964	1.1620	0.1090
AT2G45560	0.9820	0.0271	1.1919	0.0082	1.1303	0.0228	0.5871	0.3692	1.1142	0.0240	-0.2780	0.6283	1.1923	0.0044	0.6674	0.3067	0.3522	0.4947	0.6742	0.3005	-0.3557	0.6253	1.8792	0.0000	2.1317	0.0000	0.8580	0.0416
AT2G45760	-2.4443	0.0004	-1.7849	0.0164	-1.4554	0.0934	-1.1734	0.2444	-1.6748	0.0427	-1.3876	0.0856	-2.0721	0.0021	-2.8911	0.0001	-2.1453	0.0027	-2.0481	0.0124	-2.3914	0.0021	-3.1951	0.0000	-2.3861	0.0012	-2.4902	0.0002
AT2G46270	-2.3615	0.0000	0.1545	0.8268	-0.1291	0.8785	-0.2190	0.7993	-0.8165	0.1871	0.2426	0.6984	-2.9308	0.0000	-0.6679	0.3555	0.3172	0.5736	0.0590	0.9696	-0.6282	0.3521	-2.0449	0.0000	-0.1387	0.9043	-0.1030	0.8471
AT2G46400	-2.4112	0.0007	-2.5529	0.0004	-1.4387	0.1143	-1.0969	0.3040	-0.9010	0.4218	-2.0538	0.0110	-2.0920	0.0027	-2.3702	0.0023	-2.9970	0.0000	-1.8144	0.2686	-0.0799	0.9641	-2.5899	0.0002	-2.1499	0.0059	-1.7119	0.0146
AT2G46430	-1.0116	0.1719	-2.6643	0.0001	-0.9081	0.3394	-0.8991	0.3859	-0.0345	0.9895	-1.5780	0.0406	-0.9464	0.1893	-1.1275	0.2510	-2.1366	0.0020	-0.4675	0.7501	-0.2686	0.8451	-1.2260	0.0941	-1.4858	0.0672	-1.1572	0.0827
AT2G46440	-0.7578	0.3688	-2.6632	0.0002	-1.3005	0.1716	-1.2607	0.2286	-0.0899	0.9674	-2.1726	0.0074	-1.1216	0.1416	-1.1978	0.2594	-2.5033	0.0007	-0.4980	0.7539	-0.1284	0.9401	-1.0266	0.2106	-1.4078	0.1261	-1.5957	0.0243
AT2G46450	-0.6900	0.3303	-2.2093	0.0003	-1.8394	0.0078	-1.2318	0.1506	-0.8233	0.3832	-2.2448	0.0009	-0.8472	0.2007	-0.9877	0.2841	-2.6073	0.0000	-0.0860	0.9665	0.3678	0.7459	-0.7154	0.3252	-1.9454	0.0034	-2.5210	0.0000
AT2G46830	-2.7618	0.0000	-1.5754	0.0076	-1.6382	0.0097	-1.4568	0.0458	-3.0832	0.0000	-1.1841	0.0676	-3.2359	0.0000	-2.2272	0.0001	-1.4055	0.0174	-0.1130	0.9504	0.4446	0.6435	-2.6221	0.0000	-2.3236	0.0000	-1.9393	0.0004
AT2G46940	-2.3580	0.0002	-1.5548	0.0262	-1.3934	0.0798	-1.2097	0.1796	-0.8698	0.3682	-1.2969	0.0834	-2.7344	0.0000	-1.4477	0.0692	-1.7431	0.0099	-0.0727	0.9726	-0.2943	0.8146	-1.9496	0.0027	-1.4047	0.0723	-1.3188	0.0378
AT2G46970	-3.7016	0.0000	-1.3011	0.0882	-0.0412	0.9780	-2.1763	0.0083	0.5400	0.6719	-1.4981	0.0519	-3.4557	0.0000	0.0373	0.9901	-2.7100	0.0001	-1.1408	0.2464	-0.2148	0.8833	-4.0220	0.0000	-0.6750	0.5628	-1.7666	0.0071
AT2G46990	-1.4378	0.0268	-0.8153	0.2864	-0.3918	0.6932	-0.9467	0.2971	-1.0072	0.2528	-0.8460	NA	-2.3485	0.0001	0.0775	0.9715	-0.6524	0.3658	-0.7406	0.4875	-0.7453	0.4296	-1.0420	0.1236	-0.2011	0.8927	-1.1778	0.0636
AT2G47130	-1.5711	0.0222	-2.6997	0.0000	-1.6916	0.0302	-0.9776	0.3274	-0.3297	0.8237	-2.0196	0.0068	-1.5854	0.0164	-1.8007	0.0188	-2.6479	0.0001	-1.0183	0.3206	-0.7141	0.4924	-1.6998	0.0135	-1.5404	0.0502	-0.6159	0.3715
AT2G47140	-2.5032	0.0000	-1.1646	0.0546	-1.3846	0.0325	-0.2263	0.8231	-1.3037	0.0470	-2.1645	0.0006	-2.5230	0.0000	-1.6366	0.0078	-0.8659	0.1467	-0.9761	0.2055	-1.2267	0.0796	-2.4129	0.0000	-1.5268	0.0144	-2.1075	0.0002
AT2G47190	-2.5270	0.0000	-0.4299	0.5524	-0.7034	0.3575	-0.3468	0.7133	0.1222	0.9304	0.0530	0.9521	-2.0849	0.0000	-0.9455	0.2178	-0.2532	0.7166	-0.3317	0.7858	-1.7500	0.0053	-1.5230	0.0060	-0.0988	0.9437	1.2067	0.0203
AT2G47270	-3.7775	0.0000	-0.1473	0.8754	-0.6221	0.4825	-0.9657	0.2643	-0.8325	0.3467	-0.1306	0.8859	-3.6529	0.0000	-1.1739	0.1359	-0.5038	0.4760	-0.8848	0.3384	-2.8453	0.0000	-3.4750	0.0000	-0.6483	0.5148	-0.1671	0.8058
AT2G47750	0.7671	0.2281	-0.9608	0.1550	-0.9142	0.2386	-0.3281	0.7520	-0.1135	0.9416	-1.1825	0.0762	0.7230	0.2426	0.0320	0.9901	-1.6202	0.0068	-0.4446	0.7099	0.4309	0.6644	0.5289	0.4522	-0.4619	0.6732	-2.0479	0.0003
AT2G47800	-1.2813	0.0075	-1.8397	0.0001	-1.5563	0.0023	-0.5931	0.4185	-1.0845	0.0578	-1.7236	0.0008	-1.3102	0.0044	-1.3210	0.0139	-1.9053	0.0001	-1.4342	0.0088	-0.0755	0.9461	-1.4298	0.0026	-2.1096	0.0000	-2.0678	0.0000
AT2G47870	2.1373	0.0024	-0.3292	0.7636	0.8439	0.4226	0.6840	0.5616	1.9830	0.0119	0.5057	NA	2.8861	0.0000	1.4334	0.1242	0.2750	0.7779	1.0446	0.3565	0.9875	0.3527	2.7951	0.0000	2.0958	0.0066	0.7973	0.2838
AT2G47890	0.9589	0.0481	1.2885	0.0080	1.1580	0.0336	1.5704	0.0047	1.4554	0.0034	1.3699	0.0083	1.4737	0.0009	1.4888	0.0032	1.7937	0.0001	1.7569	0.0005	-0.3167	0.7066	1.5667	0.0006	2.1318	0.0000	2.1239	0.0000

AT2G48140	1.7194	0.0074	0.4928	0.5768	0.6864	0.4766	0.3104	0.7986	0.5497	0.6496	0.7757	0.3318	0.8108	0.2370	0.9642	0.3297	0.7152	0.3394	1.9554	0.0073	2.2962	0.0008	1.3469	0.0453	0.8632	0.3802	2.5046	0.0000
AT3G01060	-0.7380	0.1965	-1.1948	0.0346	-1.0277	0.1179	-0.6713	0.4005	-0.7101	0.3623	-1.5642	0.0068	-0.6970	0.2092	-0.8580	0.2558	-1.9394	0.0002	-1.0397	0.1386	0.3122	0.7419	-0.6622	0.2667	-0.8560	0.2243	-2.5823	0.0000
AT3G01080	-0.5466	0.5138	-2.7275	0.0001	-1.6296	0.0485	-1.8557	0.0353	-0.5232	0.6926	-1.6627	0.0342	-0.7831	0.2987	-1.6453	0.0482	-2.2367	0.0016	-1.0064	0.3509	-0.7264	0.4995	-0.9260	0.2368	-1.5582	0.0576	-1.2603	0.0633
AT3G01290	-0.2738	0.6959	-2.0769	0.0001	-0.7906	0.2800	-0.9324	0.2239	-0.9015	0.2173	-0.4697	0.4929	-1.1350	0.0341	-1.2752	0.0476	-1.5558	0.0045	-1.0542	0.1462	-0.9107	0.2178	-1.0033	0.0809	-2.1439	0.0001	-1.2002	0.0210
AT3G01420	-0.3801	0.6844	-1.0926	0.2031	-2.1634	0.0068	-1.9153	0.0371	-1.7360	0.0410	-2.0457	0.0112	-0.9094	0.2403	-1.8880	0.0233	-0.5723	0.5055	-0.2663	0.8923	-0.7914	0.4758	-0.0779	0.9492	-1.8576	0.0223	-1.2891	0.0677
AT3G01440	0.3185	0.7213	-1.2279	0.1135	-0.5723	0.5813	-0.5883	0.5993	-0.1654	0.9243	-1.9634	0.0097	0.5844	0.4509	0.0381	0.9901	-2.0551	0.0030	-0.1482	0.9450	0.6119	0.5769	0.5469	0.5184	-0.5566	0.6611	-2.7097	0.0000
AT3G01550	-0.0528	0.9584	-1.7428	0.0119	-1.3922	0.0863	-0.8696	0.3859	-1.0093	0.2792	-2.2871	0.0015	-0.0696	0.9401	-0.8904	0.4061	-2.7655	0.0000	-1.0972	0.2540	0.1018	0.9472	-0.1902	0.8480	-0.7313	0.4988	-2.5488	0.0001
AT3G01570	-0.0940	0.9297	1.3010	0.1041	-0.1722	0.8995	-0.1291	0.9344	-0.4448	0.7614	-0.3801	0.6973	0.2545	0.7816	-0.4945	0.7694	-0.1692	0.8637	-0.2556	0.8968	2.4132	0.0016	0.3015	0.7623	0.0613	0.9786	-0.1382	0.8692
AT3G01600	1.2391	0.0450	0.8955	0.2228	0.0606	0.9649	1.2787	0.1105	0.3472	0.7988	0.3227	0.7143	0.8694	0.1616	0.4578	0.7459	0.9274	0.1710	1.3910	0.0666	7.0593	0.0000	0.6770	0.3438	0.4591	0.7126	0.5817	0.3781
AT3G01830	-2.3703	0.0009	-2.7863	0.0001	-1.9291	0.0197	-0.8493	0.4534	-1.4701	0.1067	-2.0804	0.0100	-2.2381	0.0012	-3.0608	0.0000	-2.9942	0.0000	-1.8104	0.0415	-0.5868	0.6276	-2.9713	0.0000	-2.0646	0.0090	-1.9742	0.0049
AT3G01970	-2.1715	0.0000	-1.4187	0.0011	-1.1691	0.0169	-1.1250	0.0406	-0.4434	0.5371	-0.5983	0.2442	-2.1339	0.0000	-1.5661	0.0005	-1.0995	0.0140	-0.6417	0.3293	-2.4979	0.0000	-2.1809	0.0000	-0.9710	0.0554	0.1591	0.7386
AT3G02140	-0.2432	0.7465	-0.7980	0.2401	-0.9230	0.2173	-0.7751	0.3601	-1.4049	0.0318	-0.3930	0.5923	-1.2587	0.0242	-1.0256	0.1850	-0.2075	0.7810	-0.2857	0.8308	-0.1055	0.9361	-0.4891	0.4812	-2.2186	0.0001	-1.3153	0.0162
AT3G02380	-2.0815	0.0001	-2.0397	0.0002	-1.8131	0.0023	-1.5644	0.0249	-2.1262	0.0001	-1.9042	0.0016	-1.8051	0.0006	-1.8667	0.0016	-2.1352	0.0001	-0.3958	0.7359	0.7311	0.3679	-2.0892	0.0001	-2.3262	0.0000	-2.2254	0.0000
AT3G02480	1.1867	0.1361	1.7076	0.0330	0.5263	0.6506	0.4172	0.7539	1.0186	0.3550	0.4488	0.6474	1.2802	0.0912	0.5234	0.7631	1.0594	0.1940	1.1657	0.2912	1.8594	0.0373	5.5369	0.0000	0.1750	0.9262	0.4073	0.6117
AT3G02670	-1.9835	NA	-2.0168	0.0076	-1.0900	0.2784	-1.5243	NA	-1.6043	0.0728	-1.9220	NA	-2.1398	0.0026	-2.1078	NA	-2.2280	NA	-2.0302	NA	-0.8410	0.4558	-1.9660	0.0083	-1.1563	0.2539	-0.7380	NA
AT3G02705	-2.1933	0.0026	-2.8916	0.0000	-1.8334	0.0310	-1.4279	0.1539	-1.0903	0.2948	-1.4379	NA	-2.2375	0.0013	-2.1656	0.0075	-2.8333	0.0001	-1.5825	0.0946	-2.3181	0.0048	-1.6748	0.0268	-1.8173	0.0291	-1.0800	0.1339
AT3G02800	-1.6889	0.0026	-2.2698	0.0000	-1.4887	0.0215	0.0963	0.9383	-0.9585	0.2074	-0.7949	0.2366	-1.8752	0.0004	-1.4166	0.0316	-1.2217	0.0403	-0.9439	0.2463	-0.4103	0.6720	-2.4417	0.0000	-1.8583	0.0020	-2.0688	0.0002
AT3G02830	-1.3525	0.0002	-1.2229	0.0012	-1.1704	0.0041	-1.1101	0.0171	-1.5617	0.0000	-1.6790	0.0000	-1.3532	0.0001	-1.6248	0.0000	-1.5595	0.0000	-0.9633	0.0343	-0.1000	0.9051	-1.1575	0.0019	-1.3767	0.0003	-2.2061	0.0000
AT3G02840	-1.9415	0.0048	-1.9639	0.0059	-1.2664	0.1506	-0.1268	0.9321	-1.3361	0.1281	-1.2986	0.1016	-2.0803	0.0015	-2.2662	0.0022	-2.1483	0.0022	-1.7028	0.0439	0.8226	0.4194	-2.6072	0.0001	-2.0336	0.0065	-1.0147	0.1343
AT3G02870	0.3236	0.6123	-0.5974	0.3460	-0.1121	0.9081	-0.3522	0.6871	0.2268	0.8435	-1.0104	0.0847	0.4689	0.4131	0.0529	0.9781	-1.2745	0.0163	-0.6376	0.4522	0.0434	0.9745	0.4056	0.5233	0.1014	0.9374	-2.4031	0.0000
AT3G02885	2.8115	0.0000	-0.1368	0.8936	-0.0902	0.9445	1.0949	0.2054	1.3085	0.0825	1.1027	0.1257	1.4703	0.0126	0.4470	0.7572	-0.1171	0.8956	1.5597	0.0346	1.1962	0.1412	2.1696	0.0002	1.0596	0.1964	1.1079	0.0684
AT3G03170	0.8802	0.1681	2.0182	0.0004	1.0888	0.1520	1.0099	0.2286	1.5299	0.0209	0.9224	0.1924	0.6214	0.3348	0.8809	0.3299	1.7424	0.0033	1.5588	0.0249	0.2641	0.8279	1.5681	0.0075	1.9636	0.0012	2.4238	0.0000
AT3G03460	-1.4581	0.0070	-0.4552	0.5144	-0.8721	0.2193	-0.9010	0.2403	-0.3144	0.7758	-0.9060	0.1504	-1.4200	0.0060	-0.3135	0.8151	-0.9447	0.1031	-0.3057	0.8004	-0.3578	0.7048	-1.0421	0.0661	-0.5066	0.5886	-2.0927	0.0001
AT3G03770	0.0266	0.9787	-1.5471	0.0218	-1.4932	0.0457	-0.2989	0.7994	-0.4564	0.7092	-1.7322	0.0140	-0.0508	0.9552	-0.3587	0.8262	-1.7389	0.0074	-0.4611	0.7279	0.7053	0.4636	0.2649	0.7655	-0.4239	0.7372	-2.4741	0.0000
AT3G03780	1.6973	0.0093	-0.8675	0.2698	-0.6832	0.4743	1.0100	0.2844	0.1541	0.9263	-1.3679	0.0634	1.3834	0.0314	0.2064	0.9159	-1.4857	0.0285	-0.7898	0.4696	0.4196	0.7160	2.0881	0.0011	0.0709	0.9707	-1.2973	0.0385
AT3G04010	-1.3203	0.0237	0.3090	0.7007	0.8641	0.2552	0.3536	0.7213	-0.3328	0.7784	0.6603	0.3377	-1.2585	0.0250	-0.5021	0.6691	0.7706	0.2177	0.2576	0.8520	-2.1649	0.0005	-1.4730	0.0116	-0.4819	0.6467	0.3018	0.6236
AT3G04070	-1.6156	0.0012	-0.0276	0.9745	0.5298	0.4709	-0.1156	0.9109	1.1961	0.0403	0.7820	0.1828	-1.5139	0.0016	-0.4401	0.6718	0.1493	0.8241	0.1203	0.9359	-2.3693	0.0000	-1.0210	0.0540	0.6958	0.3392	0.7048	0.1535
AT3G04140	-0.9521	0.1587	-0.8803	0.2415	-0.8855	0.3022	-1.0975	0.2165	-0.8138	0.3908	-2.0612	0.0026	-0.7472	0.2674	-1.0070	0.2708	-1.9939	0.0017	0.2930	0.8468	0.3776	0.7379	-0.5525	0.4695	-0.6588	0.5299	-1.0104	0.0995
AT3G04200	-1.3012	0.0974	-2.0099	0.0089	-0.8792	0.4078	0.1682	0.9129	-0.3682	0.8213	-1.6223	NA	-2.0028	0.0053	-0.4212	0.8262	-1.5290	0.0507	-1.2028	0.2696	0.8718	0.4345	-1.0668	0.1965	-1.3909	0.1370	-1.7120	0.0158
AT3G04210	-0.6306	0.4618	-2.6084	0.0002	-1.2355	0.1906	-0.8322	0.4606	-0.7073	0.5685	-1.8410	0.0223	-1.2558	0.0879	-1.1329	0.2884	-2.5275	0.0004	-0.5581	0.7055	0.0572	0.9766	-1.0880	0.1719	-1.7884	0.0288	-1.7023	0.0143
AT3G04220	-1.6340	0.0207	-2.6619	0.0001	-1.4224	0.0976	-0.5797	0.6110	-1.5731	0.0586	-1.7128	0.0283	-2.0835	0.0017	-2.9323	0.0000	-2.7903	0.0001	-1.6076	0.0661	-1.4114	0.1169	-2.4282	0.0003	-2.6503	0.0002	-2.1824	0.0012
AT3G04300	-2.3372	0.0001	-2.4321	0.0001	-1.1336	0.1339	-1.3533	0.0844	-0.6549	0.4905	-1.3286	0.0517	-2.1073	0.0002	-1.7091	0.0107	-2.4754	0.0001	-1.4738	0.0453	-0.6619	0.4607	-2.1105	0.0003	-2.2102	0.0005	-1.4234	0.0153
AT3G04570	1.3749	0.0601	0.9673	0.2583	0.6091	0.5758	0.7562	0.4985	0.4843	0.7324	0.8937	0.2996	0.5412	0.5080	1.6730	0.0446	1.0318	0.1852	2.3560	0.0024	1.3096	0.1653	1.2020	0.1134	1.7159	0.0326	1.8884	0.0054
AT3G04720	-1.3296	0.0060	-2.2008	0.0000	-1.5053	0.0041	-0.5577	0.4591	-0.7766	0.2439	-0.5803	0.3208	-1.3367	0.0041	-1.4449	0.0066	-1.2288	0.0138	-0.5890	0.4683	-1.0608	0.0812	-1.2886	0.0083	-1.4393	0.0055	0.1194	0.8285
AT3G05370	-0.6130	0.2714	-1.6007	0.0017	-0.8047	0.2264	-0.6466	0.3970	-0.7062	0.3365	-1.6501	0.0028	-0.6470	0.2236	-0.3421	0.7716	-1.7356	0.0006	-0.5437	0.5441	0.5041	0.5186	-0.8195	0.1329	-0.8932	0.1706	-2.2109	0.0000
AT3G05640	-0.4093	0.6099	2.2564	0.0003	1.6853	0.0208	1.3578	0.1156	0.2349	0.8751	1.6273	0.0228	-0.7484	0.2803	0.6841	0.5642	2.4346	0.0001	1.8113	0.0155	-0.7538	0.4340	-0.1295	0.8960	1.9599	0.0040	1.3255	0.0319
AT3G05880	0.2747	0.7363	1.6809	0.0087	1.5895	0.0243	0.9273	0.3057	1.3537	0.0698	1.4768	0.0326	0.4371	0.5465	1.9529	0.0033	1.9101	0.0021	1.7895	0.0123	-0.5446	0.5865	0.9436	0.1639	2.2299	0.0004	3.7271	0.0000
AT3G05945	-1.0230	0.0406	-6.6653	0.0000	-6.5246	0.0000	-0.5287	0.4875	-6.7335	0.0000	0.2725	0.6724	-7.1970	0.0000	-0.4786	0.6101	-0.0717	0.9158	-0.0557	0.9726	0.1460	0.8894	-1.1772	0.0179	0.0660	0.9598	0.6923	0.1463
AT3G06145	-0.1487	0.8543	-1.4781	0.0143	-1.4541	0.0283	-0.6301	0.4803	-1.0726	0.1480	-1.7577	0.0059	0.0459	0.9548	-0.8000	0.3744	-1.8738	0.0013	-0.4521	0.6944	0.6275	0.4657	-0.0259	0.9803	-1.2895	0.0554	-2.1840	0.0001
AT3G06355	0.9007	0.2445	0.1216	0.9136	-0.1812	0.8901	2.2890	0.0064	-0.4731	0.7330	2.4318	0.0013	-1.0034	0.1724	1.7394	0.0338	2.7162	0.0001	2.6479	0.0004	0.9828	0.3301	0.7524	0.3614	1.1810	0.2038	2.7386	0.0000
AT3G06465	0.1346	0.8684	0.1961	0.8177	0.0000	1.0000	0.0000	1.0000	0.1773	0.8946	0.0000	NA	0.0112	0.9889	0.0000	1.0000	0.0000	1.0000	0.1594	0.9218	0.0000	1.0000	0.2773	0.7200	0.0000	1.0000	3.4444	0.0000
AT3G06520	-2.0798	0.0001	-0.6249	0.3647	-1.3268	0.0436	-1.3009	0.0748	-1.2405	0.0651	-1.7562	0.0050	-1.8667	0.0004	-0.9751	0.2031	-0.8938	0.1374	-0.1543	0.9222	0.1792	0.8772	-1.7641	0.0014	-1.3173	0.0425	-1.6930	0.0020
AT3G06655	0.5652	0.5287	-0.7600	0.4203	-0.7343	0.5049	1.7692	0.0659	-0.7445	0.5552	0.7007	0.4526	-0.9057	0.2566	0.8366	0.5283	0.3718	0.6924	0.3498	0.8456	1.3670	0.1655	0.3838	0.7027	2.8765	0.0001	2.2115	0.0018
AT3G07195	-1.2307	0.0620	-2.7432	0.0000	-1.5775	0.0320	-1.1101	0.2159	-0.9206	0.3127	-1.8284	0.0096	-1.7720	0.0036	-1.3375	0.0930	-2.5866	0.0000	-1.6210	0.0346	-0.2514	0.8431	-1.5891	0.0140	-2.1261	0.0014	-1.9312	0.0016
AT3G07315	-1.6559	0.0214	-1.5688	0.0405	-1.1798	0.2042	-2.1678	0.0119	-1.2555	0.1811	-1.1753	0.1544	-1.6523	0.0174	-1.1330	0.2729	-1.8975	0.0087	-1.5118	0.0997	-2.1063	0.0086	-1.7512	0.0153	-1.2606	0.1675	-1.8878	0.0053
AT3G07650	0.6934	0.2534	1.2849	0.0288	1.1056	0.1046	1.2412	0.0872	1.4907	0.0140	1.3601	0.0266	1.3118	0.0142	1.6061	0.0079	2.3396	0.0000	2.6556	0.0000	0.6343	0.4455	0.8133	0.1800	0.9444	0.1908	1.5478	0.0033
AT3G07675	-0.4342	0.5888	-2.6104	0.0001	-2.5608	0.0004	-0.1288	0.9254	-2.5807	0.0002	0.0559	0.9578	-3.0474	0.0000	0.2632	0.8829	-0.0355	0.9717	-0.5235	0.6938	-0.7703	0.4410	0.1145	0.9098	0.4770	0.7018	0.6094	0.3546
AT3G08435	-0.2931	0.7649	1.5699	0.0488	0.8239	0.4342	1.0013	0.3585	-0.2540	0.8813	1.1223	0.1907	-0.3431	0.7060	0.2515	0.9064	2.2959	0.0018	1.6836	0.0674	-0.2446	0.8722	-0.2096	0.8476	1.8289	0.0265	0.8958	0.2173
AT3G08660	-2.0714	0.0000	-1.3146	0.0116	-0.5838	0.4083	-1.3120	0.0366	-0.2528	0.8133	-1.1770	0.0356	-1.7060	0.0003	-0.4378	0.6672	-1.5938	0.0016	-0.5064	0.5724	-0.0792	0.9456	-1.7790	0.0002	-0.5663	0.4765	-0.5695	0.2522
AT3G08720	-1.5336	0.0001	-1.1928	0.0045	-0.5496	0.3327	-0.6082	0.3106	-0.3497	0.6401	-0.5943	0.2207	-2.1066	0.0000	-1.1367	0.0135	-0.9405	0.0282	-0.4250	0.5668	0.4584	0.4598	-1.8900	0.0000	-0.9264	0.0534	-0.6148	0.1272
AT3G08860	2.1220	0.0010	0.9304	0.2447	0.8358	0.3735	1.6826	0.0456	1.2435	0.1481	0.9458	0.2351	2.1984	0.0003	1.3899	0.0943	1.0423	0.1502	1.9875	0.0079	0.6593	0.5329	3.1382	0.0000	1.6133	0.0323	2.3488	0.0002
AT3G08870	-2.1441	0.0002	-1.6125	0.0098	-0.8599	0.2905	-1.7168	0.0213	-0.4621	0.6812	-2.1946	0.0008	-2.0697	0.0002	-1.5465	0.0246	-2.0134	0.0008	-0.7703	0.4295	-0.6087	0.5127	-1.9201	0.0011	-1.1352	0.1315	-1.6045	0.0056
AT3G08970	-1.8363	0.0013	-1.3957	0.0243	-0.9891	0.1901	-0.5675	0.5376	-0.6240	0.5048	-1.3984	0.0327	-1.6861	0.0022	-1.3275	0.0574	-2.0588	0.0005	-0.2148	0.8888	-0.3995	0.6911	-1.1676	0.0540	-1.1947	0.0907	-2.0152	0.0004
AT3G09010	-1.3594	0.0469	-2.7853	0.0000	-1.5608	0.0455	-0.4314	0.7056	-0.9569	0.3169	-2.1442	0.0030	-1.3075	0.0480	-1.5595	0.0485	-2.5616	0.0001	-1.1525	0.2198	-0.8975	0.3504	-1.5110	0.0273	-2.0659	0.0033	-2.0668	0.0012
AT3G09050	-0.6826	0.3041	-0.8102	0.2556	-0.4609	0.6137	-1.0779	0.1930	-0.2166	0.8749	-0.9824	0.1555	-0.5855	0.3718	-0.4097	0.7673	-1.3400	0.0308	-0.7652	0.4279	-0.9536	0.2446	-0.7556	0.2599	-0.7245	0.4303	-2.1357	0.0002
AT3G09405	-1.6807	0.0039	-2.5140	0.0000	-1.2177	0.0906	-0.1113	0.9294	-0.7284	0.4149	-1.0898	0.1062	-1.6084	0.0041	-1.1988	0.1076	-1.9677	0.0009	-1.2423	0.1031	-1.0155	0.2005	-1.8893	0.0011	-1.2309	0.0815	-1.3180	0.0203
AT3G09450	-3.7937	0.0000	-1.9053	0.0100	-2.1585	0.0054	-2.7845	0.0005	-2.7374	0.0001	-3.6382	0.0000	-4.0792	0.0000	-3.0063	0.0000	-3.1484	0.0000	-0.6068	0.6621	0.1877	0.9054	-2.6489	0.0001	-2.9452	0.0000	-2.8417	0.0000
AT3G09490	-1.4087	0.0094	-2.0261	0.0001	-1.2230	0.0583	-1.5095	0.0254	-0.4575	0.6407	-0.9982	0.1091	-1.9129	0.0002	-1.7154	0.0034	-1.5405	0.0054	-0.8922	0.2533	-1.1403	0.0982	-1.9094	0.0003	-1.5710	0.0075	-1.8637	0.0004
AT3G09520	-1.2548	0.0496	-2.3101	0.0002	-1.5154	0.0362	-0.5351	0.5957	-1.4548	0.0469	-1.9386	0.0048	-1.4600	0.0154	-2.0358	0.0022	-2.1197	0.0007	-0.5868	0.6148	0.6996	0.4504	-1.0643	0.1087	-2.1444	0.0010	-2.1685	0.0003
AT3G09600	-3.2851	0.0000	-2.2836	0.0000	-2.0999	0.0001	-1.6318	0.0124	-2.8927	0.0000	-1.6518	0.0050	-3.6042	0.0000	-2.4343	0.0000	-2.1196	0.0001	-0.6860	0.4346	0.5118	0.5460	-3.0193	0.0000	-2.3748	0.0000	-1.8132	0.0004
AT3G09940	-1.7761	0.0130	-3.3273	0.0000	-1.7806	0.0296	-1.2951	0.1908	-1.2754	0.1721	-1.9582	0.0128	-1.3057	0.0677	-1.9202	0.0164	-3.1010	0.0000	-0.5535	0.7001	-0.8262	0.4379	-1.9782	0.0054	-1.9015	0.0148	-1.8751	0.0060
AT3G10113	-0.3232	0.7402	0.9152	0.3126	0.8722	0.4081	0.6052	0.6203	2.7991	0.0001	0.7266	NA	-0.1205	0.9050	0.5961	0.7108	0.8795	0.2894	0.5344	0.7295	0.0426	0.9840	-0.0398	0.9761	0.4846	0.7429	0.8626	0.2420
AT3G10280	-0.0542	NA	-0.1041	0.9275	0.8333	0.4191	0.6860	NA	2.0792	0.0081	0.4036	NA	-0.1768	0.8549	0.2946	NA	0.3402	NA	0.2746	NA	0.1921	0.9035	0.1502	0.8918	0.6899	0.5771	1.2586	NA
AT3G10600	0.2280	0.7690	-1.2350	0.0749	-1.3400	0.0821	-0.2477	0.8231	-1.5841	0.0314	-0.9291	0.2090	0.1723	0.8189	-1.3182	0.0860	-0.5318	0.4542	-1.4560	0.0671	-0.1196	0.9297	0.0545	0.9562	-2.0623	0.0033	-1.7930	0.0048
AT3G10815	-3.5339	0.0000	-2.0418	0.0005	-1.8403	0.0041	-1.4333	0.0570	-1.6181	0.0124	-1.6394	0.0125	-3.6668	0.0000	-2.6283	0.0000	-1.9112	0.0014	-1.5278	0.0292	-1.7964	0.0072	-3.4246	0.0000	-1.9022	0.0024	-1.1853	0.0368
AT3G10840	-0.2901	0.6795	-0.9831	0.1140	-0.9985	0.1543	-0.8822	0.2629	-0.4347	0.6708	-1.7498	0.0035	-0.2124	0.7614	-0.3322	0.8069	-1.8701	0.0007	-0.8849	0.2698	0.0864	0.9460	-0.0604	0.9476	-0.2768	0.8174	-2.3699	0.0000
AT3G10910	-2.4090	0.0000	-0.0103	0.9902	0.4135	0.5417	-1.3148	0.0235	0.0697	0.9566	-1.6386	0.0018	-1.6255	0.0002	-0.6764	0.3332	-0.4001	0.4557	-0.3817	0.6704	0.3890	0.5967	-1.6921	0.0002	-0.2656	0.7820	-0.4081	0.3897
AT3G10930	-0.2295	0.8115	-0.6875	0.4451	-0.4863	0.6597	0.5100	0.6652	-0.4074	0.7806	-0.2732	0.7813	-0.2170	0.8144	-0.4076	0.8194	-0.7382	0.3622	-0.4075	0.7992	2.0505	0.0102	0.0102	0.9940	-0.6736	0.5830	1.7778	0.0081
AT3G11010	-1.4111	0.0544	-2.4840	0.0004	-1.0740	0.2586	-1.7281	0.0597	0.0382	0.9881	-2.1092	0.0068	-1.6387	0.0182	-1.2778	0.1860	-2.4519	0.0005	-1.1741	0.2525	-0.5191	0.6700	-1.7402	0.0161	-1.6100	0.0507	-1.5268	0.0251
AT3G11020	0.2812	0.7709	1.5709	0.0411	1.7077	0.0395	1.0124	0.3369	1.9354	0.0133	1.7273	0.0299	0.5948	0.4634	1.2097	0.2276	2.3778	0.0008	1.8515	0.0289	0.2498	0.8676	1.0490	0.1816	1.6257	0.0492	1.0629	0.1307
AT3G11080	-1.6866	0.0027	-2.1321	0.0001	-1.0706	0.1360	-1.2431	0.0982	-0.5001	0.6187	-1.3777	0.0310	-1.9080	0.0003	-1.3809	0.0381	-1.6840	0.0036	-1.0042	0.2059	-0.4419	0.6405	-1.2163	0.0386	-1.2397	0.0650	-1.0005	0.0700
AT3G11090	-1.0381	0.0695	-0.7313	0.2699	-1.2649	0.0530	-0.7636	0.3479	-0.8461	0.2703	-2.1149	0.0004	-0.6907	0.2344	-1.7045	0.0044	-1.9595	0.0004	-0.6514	0.4847	-0.0457	0.9748	-0.4865	0.4641	-0.6148	0.4875	-1.0743	0.0436
AT3G11110	-1.0164	0.0049	-1.3984	0.0001	-1.5579	0.0000	-1.4034	0.0007	-1.7467	0.0000	-2.0799	0.0000	-1.2132	0.0003	-1.9865	0.0000	-1.9696	0.0000	-1.0202	0.0164	-0.4396	0.4143	-0.9604	0.0086	-1.6306	0.0000	-1.7108	0.0000
AT3G11170	-0.2438	0.5450	-0.7817	0.0273	-0.5975	0.1599	-0.6781	0.1299	-0.8538	0.0226	-0.7087	0.0588	-0.1483	0.7207	-0.8600	0.0234	-1.0138	0.0024	-0.6673	0.1293	0.0829	0.9117	-0.3633	0.3477	-1.1437	0.0008	-2.0461	0.0000
AT3G11260	1.2875	0.0859	1.6113	0.0363	0.5515	0.6277	1.3958	0.1519	2.0293	0.0083	0.8198	NA	0.5673	0.4918	1.3458	0.1562	1.0095	0.2064	0.8674	0.4813	0.4847	0.7086	1.5493	0.0355	1.4471	0.1010	-0.3289	0.6872
AT3G11340	-3.6765	0.0000	-3.1172	0.0000	-2.3790	0.0002	-1.3166	0.1141	-2.4819	0.0001	-2.5460	0.0001	-3.0452	0.0000	-3.4880	0.0000	-2.7752	0.0000	-2.6376	0.0000	-1.9854	0.0042	-4.2480	0.0000	-3.7177	0.0000	-2.4980	0.0000
AT3G11402	0.3243	0.6731	-2.9933	0.0000	-1.6487	0.0164	-1.7064	0.0246	-0.9281	0.2734	-2.3820	0.0003	-0.6434	0.3275	-1.1682	0.1411	-2.5360	0.0000	-1.5359	0.0369	0.0477	0.9765	-0.2779	0.7370	-2.0957	0.0010	-1.4690	0.0126
AT3G11773	-1.7383	0.0070	-1.3992	0.0457	-0.8701	0.3226	-1.3810	0.1053	0.4078	0.7429	-1.0525	0.1613	-2.0367	0.0008	-0.2642	0.8794	-1.4620	0.0310	-1.4346	0.0803	-1.8974	0.0119	-2.0755	0.0012	-0.3679	0.7815	-1.4789	0.0190
AT3G11840	-1.2772	0.0090	-2.0289	0.0000	-1.0716	0.0681	-0.7383	0.2977	-0.6099	0.4116	-0.7950	0.1625	-1.6231	0.0004	-1.3144	0.0170	-1.3732	0.0059	-0.7940	0.2631	-0.5272	0.4781	-1.8186	0.0001	-1.6693	0.0011	-1.1770	0.0126
AT3G12220	-1.7444	0.0204	-2.2308	0.0029	-1.2079	0.2160	-1.5577	0.1181	0.0884	0.9686	-1.7105	0.0397	-1.6339	0.0252	-0.8502	0.5196	-2.0180	0.0076	-0.8170	0.5318	-0.3900	0.7779	-1.6121	0.0363	-1.7936	0.0335	-1.6442	0.0210
AT3G12320	-2.2150	0.0000	-1.4223	0.0061	-1.3671	0.0160	-1.1445	0.0855	-2.1997	0.0000	-0.8922	0.1254	-2.5301	0.0000	-1.7100	0.0013	-1.3815	0.0072	-0.5848	0.4981	-0.0324	0.9816	-2.3640	0.0000	-2.1654	0.0000	-0.9412	0.0533
AT3G12580	-1.5329	0.0428	-1.6768	0.0339	-1.6305	0.0644	0.8326	0.4682	-1.0901	0.2980	-2.1030	0.0097	-1.9346	0.0064	-1.4550	0.1265	-1.5895	0.0392	0.6827	0.6258	0.4263	0.7495	-0.4608	0.6308	-1.3216	0.1614	-1.2828	0.0721
AT3G12700	2.1487	0.0000	-0.0614	0.9340	0.2401	0.7471	0.6359	0.3354	0.6723	0.2893	0.0764	0.9134	1.6969	0.0000	0.8341	0.1616	-0.3502	0.5185	0.1646	0.8930	0.9083	0.1150	1.9727	0.0000	1.1147	0.0267	0.1725	0.7322
AT3G12710	-2.4628	0.0000	-0.7439	0.0329	-0.2394	0.6338	-1.0285	0.0085	-0.0165	0.9892	-1.0186	0.0042	-2.1949	0.0000	-0.6032	0.1640	-1.3128	0.0000	-0.8994	0.0180	-0.2529	0.6418	-2.1568	0.0000	-0.2279	0.7175	-0.0989	0.7854
AT3G12910	-2.4280	0.0004	-1.7824	0.0164	-0.7429	0.4657	-1.1783	0.2360	-0.6544	0.5894	-0.6007	0.4967	-1.5722	0.0212	-1.3737	0.1310	-0.7745	0.3277	-1.0769	0.3019	-0.6856	0.5304	-2.5158	0.0002	-1.4238	0.0980	-1.1389	0.0973
AT3G13100	-2.1269	0.0001	-2.2473	0.0000	-1.3320	0.0406	-0.9656	0.2176	-0.6725	0.4327	-1.5689	0.0112	-2.2940	0.0000	-1.6568	0.0067	-2.3608	0.0000	-1.5556	0.0167	-0.7215	0.3722	-2.0777	0.0001	-1.9311	0.0008	-1.8617	0.0005
AT3G13432	-1.1934	NA	-1.2497	0.1432	-1.1915	0.2237	-1.0231	NA	-1.1979	0.2398	-1.4474	NA	-1.5129	0.0413	-1.8504	NA	-1.7314	NA	-0.5114	NA	-1.3586	0.1690	-2.1223	0.0038	-1.2756	0.1866	-0.2875	NA
AT3G13433	-1.3678	0.0796	-2.1449	0.0046	-1.6420	0.0661	-1.5950	0.1095	-0.6358	0.6401	-0.7999	NA	-1.4369	0.0541	-1.6719	0.0647	-1.4960	0.0568	-1.4869	0.1337	-1.6264	0.0814	-1.5001	0.0555	-1.3511	0.1552	-0.8298	0.2659
AT3G13470	1.5585	0.0037	-0.1700	0.8375	-1.0297	0.1343	0.6802	0.4116	-0.3058	0.7885	-0.8529	0.1801	1.3162	0.0126	0.6003	0.5389	-0.8187	0.1655	-0.5549	0.5730	0.4695	0.5943	1.4915	0.0060	-0.8747	0.2318	-2.0086	0.0001
AT3G13610	-2.5334	0.0004	-2.2402	0.0024	-1.8851	0.0237	-0.8022	0.4812	-0.9706	0.3717	-1.4245	0.0873	-1.0870	0.1495	-2.2158	0.0054	-2.4066	0.0010	-2.2526	0.0066	-1.1381	0.2608	-2.3883	0.0008	-1.8870	0.0203	-1.3730	0.0514
AT3G13672	2.7398	0.0000	0.0043	0.9983	-0.7903	0.4158	0.4397	0.6961	0.0542	0.9807	0.4990	0.5576	2.2558	0.0002	0.0902	0.9693	0.2236	0.7996	1.1775	0.1955	1.6365	0.0355	2.7892	0.0000	-0.1110	0.9478	1.3382	0.0338
AT3G13784	1.2129	0.1230	0.0727	0.9535	-0.1177	0.9361	0.5702	0.6443	1.3638	0.1557	-0.0701	0.9527	1.1597	0.1288	0.5890	0.7166	-0.0963	0.9258	0.9152	0.4566	0.6982	0.5506	3.6557	0.0000	0.0307	0.9911	0.1569	0.8528
AT3G13850	-1.3844	0.0620	-1.0642	0.2077	-0.2882	0.8117	-1.6361	0.0828	0.2634	0.8699	-1.2616	NA	-1.5931	0.0220	-0.8015	0.5246	-2.1365	0.0036	-1.0361	0.3467	-1.3925	0.1412	-1.7907	0.0145	-0.6778	0.5838	-1.1302	0.1090
AT3G13900	-1.2020	0.0742	-1.4468	0.0425	-0.8579	0.3381	-1.2127	0.1742	-0.5117	0.6669	-1.0898	NA	-2.0524	0.0009	-1.0126	0.2831	-1.6343	0.0180	-1.0957	0.2395	-1.0636	0.2358	-1.8598	0.0048	-1.5649	0.0424	-1.4405	0.0256
AT3G13950	-2.3785	0.0008	-2.5776	0.0003	-2.0480	0.0115	-0.5425	0.6564	-1.2771	0.1853	-1.7205	0.0343	-2.3124	0.0008	-2.3889	0.0020	-2.3763	0.0011	-1.3989	0.1538	-1.9674	0.0207	-2.0732	0.0042	-2.0128	0.0110	-0.7973	0.2734
AT3G14210	-0.5841	0.4629	-1.7766	0.0112	-0.9757	0.2847	-0.9283	0.3535	0.4459	0.7377	-1.5618	0.0381	-0.6768	0.3625	0.1678	0.9378	-2.0100	0.0031	-0.6310	0.6213	-0.1538	0.9193	-0.4082	0.6421	0.1385	0.9353	-1.4915	0.0210
AT3G14570	-2.0624	0.0000	-0.8768	0.0586	-0.9080	0.0764	-0.3991	0.5563	-0.9555	0.0582	-0.7813	0.1083	-2.2268	0.0000	-0.6828	0.2632	-0.4956	0.2961	-0.7277	0.2245	-0.7736	0.1705	-1.6644	0.0000	-0.8266	0.1147	-1.4593	0.0003
AT3G14620	-1.9873	0.0004	-1.7401	0.0027	-1.4186	0.0321	-0.4191	0.6626	-1.1747	0.0973	-0.8549	0.2045	-2.6043	0.0000	-1.7715	0.0043	-1.6085	0.0057	-0.7755	0.3950	-1.3567	0.0523	-2.0219	0.0003	-1.6547	0.0072	-1.6008	0.0035
AT3G14770	-2.3786	0.0000	-0.5605	0.2395	-0.5351	0.3310	-0.9074	0.0841	-2.6113	0.0000	-0.3405	0.5000	-2.0394	0.0000	-1.2589	0.0034	-0.2384	0.6278	-0.2789	0.7404	-0.1756	0.8224	-2.3934	0.0000	-2.5869	0.0000	-0.3296	0.4225
AT3G14780	-2.6397	0.0000	-1.5599	0.0024	-1.8062	0.0008	-1.7012	0.0044	-1.1083	0.0665	-1.3966	0.0137	-3.1377	0.0000	-1.3531	0.0167	-1.3893	0.0074	-1.2219	0.0472	-2.0888	0.0001	-2.8489	0.0000	-1.8911	0.0004	-2.1272	0.0000
AT3G14810	-1.4172	0.0039	0.3110	0.6484	-0.2341	0.7778	-0.0779	0.9441	-1.7216	0.0007	0.2932	0.6532	-2.0478	0.0000	-0.6649	0.4055	0.7092	0.1895	0.0990	0.9503	-0.8930	0.1790	-1.2857	0.0104	-0.1635	0.8903	-0.3657	0.4792
AT3G14900	0.2916	0.6983	-0.4648	0.5449	-0.9620	0.2087	-0.7387	0.4029	-0.3956	0.7295	-1.1331	0.0889	0.2056	0.7846	-0.1101	0.9565	-1.5371	0.0102	-0.8725	0.3240	-0.3659	0.7240	0.7049	0.2879	-0.5409	0.5959	-2.2683	0.0000
AT3G15354	-1.9832	0.0006	-1.6559	0.0064	-1.6191	0.0144	-1.2877	0.1012	-1.7738	0.0049	-1.9136	0.0028	-1.8296	0.0011	-1.5446	0.0217	-2.1483	0.0002	-0.1834	0.9129	0.3508	0.7417	-1.5484	0.0094	-1.9516	0.0015	-2.6158	0.0000
AT3G15356	-1.8159	0.0001	-2.1003	0.0000	-1.0356	0.0812	-0.7772	0.2703	-1.8498	0.0001	-0.5283	0.3765	-2.4291	0.0000	-1.2477	0.0262	-1.3650	0.0060	-1.1055	0.0730	-1.1957	0.0424	-2.0607	0.0000	-2.0053	0.0000	-0.8920	0.0589
AT3G15440	-0.1481	0.8901	1.8303	0.0188	1.5235	0.0930	1.5887	0.1063	0.6118	0.6527	2.2137	0.0063	-0.3193	0.7318	1.3034	0.2003	2.4539	0.0009	2.2933	0.0060	-0.5796	0.6421	0.1846	0.8690	1.2805	0.1837	2.2465	0.0015
AT3G15500	-1.8524	0.0013	0.1796	0.8399	0.3601	0.7002	0.7059	0.4311	-0.5816	0.5568	1.6207	0.0118	-1.6965	0.0024	0.1165	0.9535	1.2448	0.0415	0.8642	0.3306	-2.5052	0.0000	-1.7198	0.0031	0.3358	0.7833	1.1468	0.0409
AT3G15536	-1.4186	0.0678	-2.3085	0.0021	-1.9334	0.0228	-1.5515	0.1228	0.1447	0.9418	-1.1994	0.1679	-2.0869	0.0035	-1.5899	0.0866	-2.0856	0.0059	-0.6228	0.6704	-0.5664	0.6541	-1.4499	0.0655	-1.7794	0.0367	-0.5230	0.5030
AT3G15540	-2.9547	0.0000	-0.6060	0.3576	0.0274	0.9802	-1.1635	0.0991	0.1347	0.9184	-0.5459	0.4053	-2.6064	0.0000	0.2597	0.8556	-1.2617	0.0220	-1.9527	0.0006	-0.8511	0.2448	-2.7591	0.0000	0.4784	0.6113	-0.8528	0.0990
AT3G15650	0.9397	0.1803	0.2338	0.8152	-0.2961	0.7954	0.7731	0.4429	0.3307	0.8184	-0.4330	0.6249	1.5300	0.0143	0.5956	0.6539	-0.2505	0.7768	0.7165	0.5358	2.0021	0.0054	1.5174	0.0216	-0.1651	0.9227	-0.1980	0.7954
AT3G15670	0.1512	0.8866	3.4778	0.0000	0.3988	0.7414	0.2529	0.8621	0.2161	0.9027	0.1884	0.8627	0.4467	0.6136	0.3550	0.8583	0.3754	0.6846	-0.1471	0.9502	3.2976	0.0000	2.4311	0.0007	0.0063	0.9999	-0.1628	0.8459
AT3G15720	0.6050	0.4682	0.5422	0.5644	0.4368	0.7005	0.7936	0.4697	0.7762	0.4951	0.2390	0.8136	1.0726	0.1375	0.4938	0.7638	0.1840	0.8492	0.7742	0.5331	0.6285	0.5805	2.3482	0.0005	0.5745	0.6623	-0.2067	0.7980
AT3G15760	-2.5705	0.0000	-0.8166	0.0692	-0.9710	0.0415	-1.3240	0.0065	-0.8210	0.1061	-0.7867	0.0917	-2.6264	0.0000	-1.4826	0.0005	-1.1809	0.0043	-1.4039	0.0018	-0.6451	0.2513	-2.1472	0.0000	-1.0868	0.0156	-0.2494	0.5641
AT3G16175	-1.4719	0.0224	-0.8473	0.2662	-1.0959	0.1846	-1.1251	0.2056	-0.9525	0.2873	-1.4922	0.0384	-1.2647	0.0430	-1.2756	0.1172	-1.3027	0.0528	-0.6576	0.5633	-0.6177	0.5329	-0.9907	0.1491	-1.0285	0.2295	-2.1652	0.0006
AT3G16250	0.2998	0.6534	-1.1348	0.0480	-0.9443	0.1638	-0.4934	0.5616	-0.2358	0.8416	-1.6132	0.0055	0.3431	0.5826	-0.1915	0.9010	-1.8982	0.0003	-1.2667	0.0521	-0.0834	0.9460	0.2497	0.7290	-0.3920	0.6988	-2.8693	0.0000
AT3G16360	1.3593	0.0735	-1.0318	0.2387	0.3136	0.8013	-0.3419	0.8009	2.0452	0.0099	-0.8923	0.3122	1.1830	0.1125	1.4087	0.1388	-0.7704	0.3579	1.7647	0.0482	2.0029	0.0177	1.7744	0.0168	1.2005	0.2161	1.2538	0.0761
AT3G16430	1.2344	0.0147	0.3661	0.5967	0.4222	0.5873	-0.0572	0.9606	-0.5145	0.5667	0.0428	0.9599	0.0903	0.8945	0.4907	0.6218	0.2426	0.7116	1.2561	0.0396	2.1773	0.0000	0.8158	0.1364	0.3672	0.7128	1.2512	0.0102
AT3G16530	-1.9698	0.0002	-2.9812	0.0000	-1.2408	0.0593	-0.4487	0.6212	-1.6709	0.0044	-1.0653	0.0912	-2.5261	0.0000	-1.5207	0.0138	-2.3404	0.0000	-1.3522	0.0451	-0.8576	0.2629	-2.3663	0.0000	-2.1124	0.0001	-1.5413	0.0035
AT3G16800	-1.9327	0.0003	-1.0613	0.0823	-0.6031	0.4495	-1.0060	0.1883	-0.5227	0.5825	-0.5964	0.3742	-2.2134	0.0000	-0.0756	0.9693	-1.2687	0.0265	-0.0460	0.9789	0.7972	0.3039	-1.7447	0.0012	-0.3421	0.7591	-0.9784	0.0659
AT3G16860	-0.4232	0.3870	-0.1284	0.8431	-0.3573	0.5832	-0.2636	0.7222	-0.5169	0.4389	-0.5967	0.2483	-1.1664	0.0045	-0.6075	0.3650	-0.6701	0.1543	-0.5561	0.4261	2.4151	0.0000	-1.1359	0.0095	-0.9729	0.0604	-0.0746	0.8814
AT3G17170	-0.2286	0.7889	-0.5528	0.4995	-0.9999	0.2306	-0.7837	0.4172	0.0497	0.9812	-0.9552	0.1976	-0.3524	0.6454	0.1116	0.9600	-1.2487	0.0614	-0.6076	0.6087	-0.4573	0.6740	0.0873	0.9310	-0.5760	0.6064	-2.3699	0.0001
AT3G17330	-0.5837	0.3780	-1.7853	0.0022	-0.9229	0.2258	-1.0985	0.1691	-0.4551	0.6734	-2.0238	0.0012	-0.9011	0.1298	-0.6419	0.5357	-2.3472	0.0000	-0.6408	0.5274	0.6018	0.4986	-0.5537	0.4231	-0.4098	0.7143	-1.9953	0.0003
AT3G17510	-1.9429	0.0000	0.0406	0.9498	-0.1527	0.8185	-0.2423	0.7170	-1.7022	0.0000	0.5063	0.2688	-2.1950	0.0000	-1.0729	0.0125	0.3051	0.5014	-0.3454	0.6434	-0.5132	0.3587	-1.7761	0.0000	-1.0711	0.0104	-1.2404	0.0007
AT3G17520	0.5210	0.5392	1.7614	0.0168	0.2167	0.8613	0.0640	0.9676	0.0656	0.9773	-0.0512	0.9649	-0.1381	0.8830	-0.0494	0.9874	0.7903	0.3170	0.2162	0.9132	1.1859	0.2140	2.6017	0.0001	0.3964	0.7880	0.1026	0.8999
AT3G17609	-3.8827	0.0000	-2.5769	0.0000	-2.8603	0.0000	-2.0034	0.0064	-2.9527	0.0000	-2.5495	0.0001	-3.9289	0.0000	-2.7283	0.0000	-2.7089	0.0000	-0.8871	0.3503	-0.4382	0.6810	-3.2588	0.0000	-3.5206	0.0000	-3.7078	0.0000
AT3G17690	-1.8113	0.0000	-2.2195	0.0000	-1.5804	0.0013	-1.2165	0.0310	-1.0355	0.0549	-1.7241	0.0008	-2.3256	0.0000	-2.1649	0.0000	-2.4471	0.0000	-1.5451	0.0027	-0.3137	0.6831	-2.6604	0.0000	-1.8579	0.0001	-2.1833	0.0000
AT3G17790	-1.1780	0.0818	0.3791	0.6724	0.1898	0.8700	0.0397	0.9799	0.8222	0.4004	-0.1074	0.9142	-0.6421	0.3682	-0.4970	0.7274	-0.5587	0.4595	-0.1963	0.9149	-0.2298	0.8635	0.2576	0.7753	0.4330	0.7355	2.4625	0.0001
AT3G18000	2.6388	0.0000	0.1900	0.8025	0.0297	0.9783	0.5472	0.4883	1.4466	0.0079	-0.5871	0.3396	2.4502	0.0000	1.1348	0.0628	-0.5039	0.3875	-0.2479	0.8364	0.1314	0.9074	2.4921	0.0000	1.1772	0.0424	-0.6141	0.2242
AT3G18250	-1.5993	0.0029	-2.6022	0.0000	-1.8792	0.0010	-1.6269	0.0141	-0.4213	0.6771	-0.7949	0.2127	-1.9876	0.0001	-2.0374	0.0003	-1.2385	0.0285	-1.3379	0.0431	-2.6852	0.0000	-1.3878	0.0114	-1.5555	0.0082	-0.1087	0.8612
AT3G18750	-0.6416	0.2469	-0.9800	0.0805	-1.0103	0.1058	-0.9139	0.1935	-0.8602	0.2031	-1.2161	0.0313	-0.8466	0.0987	-0.9491	0.1559	-1.4544	0.0045	-0.5603	0.5259	0.1012	0.9296	-0.5975	0.3016	-0.7389	0.2990	-2.3879	0.0000
AT3G19030	-1.3955	0.0048	-0.1745	0.8159	-0.6796	0.3196	0.1835	0.8489	-1.4648	0.0062	0.2002	0.7712	-2.2870	0.0000	-0.6092	0.4710	-0.1163	0.8637	-0.2728	0.8117	1.1138	0.0700	-1.6340	0.0008	-1.9579	0.0001	-1.1850	0.0133
AT3G19150	-0.8736	0.1026	-1.1189	0.0425	-0.8093	0.2278	-0.9144	0.1986	-0.0520	0.9739	-0.7714	0.1972	-0.7648	0.1442	-0.5735	0.5256	-1.4383	0.0057	-0.5070	0.5863	-0.2200	0.8259	-0.9881	0.0651	-1.4672	0.0079	-2.2177	0.0000
AT3G19450	-1.2491	0.0097	-1.2935	0.0096	-1.1531	0.0402	-0.9692	0.1364	-0.9736	0.1039	-1.5648	0.0027	-1.2601	0.0066	-1.2145	0.0289	-2.0440	0.0000	-0.9677	0.1292	-0.2998	0.7270	-1.0732	0.0309	-1.0899	0.0513	-2.5031	0.0000
AT3G19480	0.3283	0.5205	-0.8737	0.0616	-0.5891	0.3120	-0.6075	0.3333	-0.7950	0.1482	-1.2134	0.0101	0.3905	0.4103	-0.8455	0.1236	-1.4677	0.0005	-1.2716	0.0090	-0.3807	0.5766	0.3274	0.5361	-0.5883	0.3410	-2.7952	0.0000
AT3G19580	-1.9781	0.0000	-0.2217	0.6588	-0.3964	0.4518	-0.1063	0.8830	-0.4755	0.3835	-0.1822	0.7105	-1.9899	0.0000	-0.8540	0.0485	0.0086	0.9887	-0.5222	0.3519	0.3310	0.5633	-2.0658	0.0000	-0.3208	0.6223	0.4178	0.2462
AT3G19710	1.1375	0.1341	-2.3188	0.0011	-1.1919	0.2003	0.2320	0.8698	-0.1488	0.9362	-1.6786	0.0349	0.7458	0.3397	0.0168	0.9956	-1.4377	0.0556	-0.5090	0.7324	-0.1755	0.9125	1.2703	0.0944	-0.4240	0.7735	-1.4664	0.0322
AT3G19920	-0.0124	0.9924	2.0592	0.0066	0.7280	0.5026	0.9710	0.3815	0.1003	0.9620	0.0264	NA	0.0029	0.9991	0.7103	0.6217	1.2308	0.1202	0.2035	0.9231	2.3165	0.0048	0.4843	0.6104	0.3311	0.8397	0.8178	0.2647
AT3G20250	-1.8902	0.0000	-0.4958	0.1301	-0.4163	0.2840	-0.7243	0.0480	-0.3785	0.3773	-0.1976	0.5932	-2.0246	0.0000	-0.7209	0.0298	-0.1460	0.6855	0.0434	0.9634	-0.6405	0.0732	-1.5934	0.0000	-0.1148	0.8613	0.1935	0.5207
AT3G20395	-2.4828	0.0000	-1.5749	0.0120	-0.4014	0.6709	-1.4750	0.0570	-0.1463	0.9232	-1.8990	0.0038	-2.3656	0.0000	-0.7203	0.4847	-2.1689	0.0003	-2.4576	0.0001	-3.3130	0.0000	-2.2161	0.0001	-0.3156	0.8069	-1.6129	0.0053
AT3G21080	-2.4009	0.0000	-3.3546	0.0000	-1.6705	0.0040	-2.4521	0.0000	-0.8742	0.2291	-1.6483	0.0052	-3.7993	0.0000	-2.5548	0.0000	-2.7113	0.0000	-1.1979	0.0773	-1.6430	0.0065	-2.7202	0.0000	-2.4034	0.0000	-0.9015	0.0831
AT3G21150	-3.0372	0.0000	-1.4162	0.0781	-2.2190	0.0048	-1.9576	0.0312	-2.3586	0.0017	-2.8532	0.0002	-3.2628	0.0000	-2.9259	0.0001	-2.5159	0.0005	0.1139	0.9620	0.3033	0.8334	-2.8451	0.0000	-2.9314	0.0001	-2.2928	0.0009
AT3G21230	-1.0880	0.1003	-2.2338	0.0003	-1.2598	0.1058	-0.9057	0.3290	-0.2164	0.8827	-2.5458	0.0002	-0.8656	0.1890	-0.9481	0.3163	-2.5731	0.0000	-0.6929	0.5306	0.0486	0.9773	-0.9694	0.1578	-1.5387	0.0310	-1.9411	0.0013
AT3G21320	-3.0076	0.0000	-0.2186	0.8254	1.0283	0.2329	-0.7396	0.4687	0.9772	0.2895	0.1902	0.8428	-2.7884	0.0000	0.3624	0.8296	-0.5905	0.4385	-1.6185	0.0438	-1.2412	0.1529	-2.9084	0.0000	0.4364	0.7366	-0.3286	0.6434
AT3G21330	-2.0439	0.0011	0.5309	0.5268	1.2259	0.1280	-0.1215	0.9302	0.9472	0.2986	0.4953	0.5452	-2.1452	0.0004	0.7271	0.5232	-0.5282	0.4833	-0.8570	0.4037	0.3166	0.7950	-2.2374	0.0003	1.0370	0.2272	-0.2519	0.7219
AT3G21520	-2.5743	0.0002	-2.6200	0.0002	-1.6161	0.0586	-1.6519	0.0787	-1.7798	0.0308	-1.9765	NA	-2.4849	0.0002	-2.1999	0.0049	-1.9339	0.0086	-1.3529	0.1640	-1.2148	0.2116	-2.8437	0.0000	-1.9264	0.0150	-1.2477	0.0729
AT3G21530	-0.5996	0.3423	-2.2241	0.0000	-1.2760	0.0558	-1.2430	0.0937	-1.2532	0.0622	-1.2405	0.0505	-0.9905	0.0785	-0.9271	0.2431	-2.0764	0.0002	-0.2116	0.8871	0.3974	0.6800	-0.5183	0.4404	-1.3416	0.0372	-2.0008	0.0002
AT3G21670	-2.7232	0.0000	-0.1089	0.9044	-0.9500	0.2061	-0.9862	0.2244	-2.4553	0.0000	-1.5568	0.0141	-2.7541	0.0000	-1.5170	0.0197	-0.4080	0.5531	-0.0281	0.9860	0.2724	0.8040	-1.2614	0.0336	-1.1856	0.0862	-1.1788	0.0324
AT3G21720	0.8682	0.2978	4.7894	0.0000	1.0624	0.2952	-0.2411	0.8708	1.2461	0.2155	-0.3865	0.7006	0.6174	0.4688	0.2954	0.8877	0.3013	0.7561	-0.1059	0.9664	4.8030	0.0000	0.6091	0.5086	-0.1385	0.9416	0.7749	0.3016
AT3G22060	-1.5778	0.0001	-1.7647	0.0000	-1.1766	0.0120	-1.3322	0.0085	-2.2090	0.0000	-0.8199	0.0873	-2.3557	0.0000	-1.9167	0.0000	-1.5235	0.0003	-1.5692	0.0005	-1.2392	0.0097	-1.8106	0.0000	-1.8700	0.0000	-0.9785	0.0153
AT3G22121	-1.2304	0.1052	-1.3849	0.0841	-2.1402	0.0069	-2.1949	0.0123	-1.1097	0.2692	-1.7021	0.0348	-1.8986	0.0062	-1.0594	0.3338	-2.1834	0.0027	-2.2585	0.0056	-2.4731	0.0016	-1.3227	0.0837	-1.3473	0.1370	-1.6758	0.0156
AT3G22150	-0.8327	0.0899	-0.7876	0.1427	-0.7699	0.2105	-0.7672	0.2526	-0.8940	0.1355	-1.2296	0.0180	-0.7501	0.1174	-0.7457	0.2734	-1.1349	0.0194	-0.4558	0.6007	-0.1930	0.8358	-0.5086	0.3510	-0.5238	0.4874	-2.1670	0.0000
AT3G22160	-2.1447	0.0000	-1.8678	0.0000	-1.3166	0.0067	-0.5042	0.4591	-0.8720	0.1159	-1.0537	0.0334	-1.9303	0.0000	-1.3535	0.0048	-1.7103	0.0001	-1.5423	0.0015	-1.4030	0.0046	-1.9198	0.0000	-1.3197	0.0054	-0.6290	0.1471
AT3G22600	-1.5677	0.0100	-1.9314	0.0015	-1.6583	0.0149	-1.4696	0.0616	-1.2951	0.0798	-1.2158	0.0782	-1.6553	0.0044	-2.3843	0.0001	-1.0634	0.1015	-1.3882	0.0684	-1.3289	0.0808	-1.3456	0.0318	-1.7965	0.0057	0.3297	0.6124
AT3G22740	1.2352	0.0230	-1.5904	0.0054	-2.0326	0.0005	0.3014	0.7537	-0.9662	0.1856	-1.0807	0.0879	0.6990	0.2168	-0.2930	0.8350	-0.1146	0.8781	-0.0335	0.9836	-0.2426	0.8201	1.8825	0.0003	-0.1035	0.9400	0.2332	0.6965

AT3G22800	2.2256	0.0011	0.0392	0.9745	0.0023	0.9995	-0.0438	0.9799	-0.1632	0.9290	-0.9625	0.2683	1.2482	0.0757	0.7691	0.5471	-0.0335	0.9766	0.4003	0.8045	0.8925	0.3892	1.3143	0.0758	-0.2669	0.8742	-0.6193	0.4075
AT3G23010	-1.5808	0.0031	-2.9647	0.0000	-1.6187	0.0064	-1.9946	0.0013	-0.6522	0.4314	-1.8957	0.0013	-1.7398	0.0006	-1.5018	0.0128	-2.3712	0.0000	-1.8872	0.0015	-0.7337	0.3452	-1.9115	0.0003	-1.3226	0.0310	-1.0935	0.0359
AT3G23030	-1.6979	0.0024	-0.6040	0.3973	-0.0804	0.9439	-0.9109	0.2638	0.0152	0.9953	0.2658	0.7318	-2.1803	0.0000	-0.1395	0.9393	-0.6678	0.2944	-0.7263	0.4326	-0.5323	0.5537	-1.7863	0.0013	-0.0318	0.9873	-0.6536	0.2482
AT3G23050	-2.0632	0.0000	-0.9133	0.0462	-0.0595	0.9441	-1.1344	0.0322	-0.1729	0.8576	-0.3570	0.5038	-2.1607	0.0000	-0.6676	0.2796	-0.9291	0.0354	-1.1155	0.0261	-0.9366	0.0757	-2.0135	0.0000	-0.5378	0.3994	-0.3428	0.4311
AT3G23120	-2.0615	0.0055	-2.7452	0.0002	-1.3651	0.1512	-1.7215	0.0793	-0.2944	0.8626	-2.3669	0.0035	-1.9791	0.0059	-1.5990	0.0841	-2.6834	0.0003	-1.3804	0.1800	-0.9775	0.3678	-2.1982	0.0030	-1.7651	0.0390	-1.8255	0.0107
AT3G23150	-1.7518	0.0001	-0.6957	0.1998	-0.2207	0.7734	-0.8886	0.1599	0.4515	0.5631	-1.1672	0.0234	-1.4926	0.0006	-0.0565	0.9732	-1.4983	0.0012	-1.4010	0.0076	-2.3182	0.0000	-1.6735	0.0002	-0.2025	0.8441	-1.1738	0.0082
AT3G23230	-1.8061	0.0073	-1.7771	0.0118	-0.8608	0.3536	0.8725	0.3778	-2.4689	0.0004	-0.4998	0.5595	-2.4237	0.0001	-1.4100	0.0935	-1.4115	0.0463	-1.5049	0.0778	0.1915	0.8946	-1.9826	0.0031	-1.7877	0.0175	-1.1695	0.0744
AT3G23240	-2.7587	0.0000	-0.8570	0.1698	-1.3615	0.0316	0.0844	0.9422	-0.5201	0.5677	-1.1767	0.0585	-2.8817	0.0000	-1.0870	0.1134	-1.0987	0.0558	-1.1420	0.1050	-1.1530	0.0953	-3.2237	0.0000	-1.1360	0.0853	-0.9678	0.0692
AT3G23250	-2.0356	0.0017	-3.2185	0.0000	-1.9718	0.0066	-1.0412	0.2741	-1.9041	0.0079	-2.1209	0.0033	-2.8695	0.0000	-2.9110	0.0000	-2.6545	0.0001	-1.2792	0.1489	-0.2874	0.8237	-2.7796	0.0000	-2.3537	0.0005	-2.1940	0.0005
AT3G23510	-2.0251	0.0000	-2.5664	0.0000	-2.3918	0.0000	-2.0443	0.0004	-1.9028	0.0002	-2.6095	0.0000	-1.8438	0.0001	-3.2903	0.0000	-3.2025	0.0000	-3.4465	0.0000	-2.0834	0.0001	-2.1664	0.0000	-2.9877	0.0000	-3.8042	0.0000
AT3G23550	-0.9439	0.1906	-3.1619	0.0000	-1.6719	0.0288	0.1428	0.9180	-1.6405	0.0320	-0.9716	0.2153	-1.1890	0.0767	-1.2644	0.1496	-2.0639	0.0020	-0.6110	0.6309	-1.0079	0.2796	-1.0153	0.1631	-2.3239	0.0006	-1.7836	0.0052
AT3G23800	-2.6821	0.0000	-0.2117	0.8087	-0.0664	0.9550	-1.4835	0.0570	0.4637	0.6584	-0.0169	0.9873	-2.7525	0.0000	0.4664	0.6993	-0.9676	0.1396	-1.1510	0.1622	-1.3098	0.0937	-2.6758	0.0000	0.4708	0.6566	0.3803	0.5340
AT3G23870	2.0439	0.0003	0.8585	0.2273	1.2835	0.0718	0.9559	0.2597	2.5733	0.0000	0.9121	0.1976	2.4487	0.0000	2.0136	0.0009	1.1047	0.0814	1.2369	0.1084	0.2457	0.8420	2.3618	0.0000	1.8272	0.0031	2.0410	0.0002
AT3G23930	-2.0021	0.0043	-1.5832	0.0374	-1.1158	0.2300	-1.2061	0.2266	-0.3617	0.8111	-1.1756	0.1520	-1.7497	0.0103	-0.4499	0.7899	-1.5496	0.0361	0.0553	0.9800	0.0503	0.9786	-1.4525	0.0484	-0.9843	0.3337	-2.2056	0.0012
AT3G24020	-1.8883	0.0042	-0.5503	0.5012	-0.0069	0.9964	-1.1734	0.1840	-1.1773	0.1713	-1.8353	NA	-2.0695	0.0008	-0.9953	0.2844	-0.8988	0.2078	-1.0474	0.2686	0.6145	0.5092	-1.9943	0.0025	-1.1463	0.1841	-1.2436	0.0557
AT3G24300	0.5513	0.4744	0.3905	0.6702	-0.0125	0.9933	0.2011	0.8782	0.5232	0.6702	-0.6265	0.4736	0.7656	0.2633	0.2850	0.8750	0.1075	0.9077	0.4786	0.7265	2.5841	0.0001	1.1623	0.0882	0.7234	0.5006	0.9730	0.1314
AT3G24460	-1.9080	0.0003	-2.7966	0.0000	-2.6345	0.0000	-1.9761	0.0019	-2.1918	0.0001	-2.7956	0.0000	-1.9696	0.0001	-2.3281	0.0000	-2.9138	0.0000	-1.5997	0.0116	-0.6153	0.4574	-1.1418	0.0457	-3.0707	0.0000	-3.1540	0.0000
AT3G24518	-2.1614	0.0001	0.4319	0.5318	0.3458	0.6797	-0.1642	0.8772	-1.0038	0.1690	0.4785	0.4741	-2.4784	0.0000	0.0504	0.9795	0.7797	0.1711	0.1493	0.9221	-0.1869	0.8672	-2.0726	0.0002	0.3505	0.7410	0.7165	0.1696
AT3G24900	-2.0940	0.0026	-1.6240	0.0312	-1.3631	0.1210	-1.0324	0.3120	0.2097	0.8999	-1.4039	0.0799	-1.6463	0.0156	-1.4891	0.0884	-1.9044	0.0084	-0.8449	0.4708	-0.1834	0.9055	-1.8800	0.0078	-1.0812	0.2617	-1.0850	0.1142
AT3G25010	-2.0027	0.0010	-2.3386	0.0001	-1.2251	0.1121	-1.9125	0.0112	-0.0947	0.9566	-1.4440	0.0374	-1.9662	0.0008	-1.7826	0.0093	-2.2851	0.0002	-1.1116	0.1986	-0.7536	0.4125	-2.0515	0.0007	-1.6645	0.0144	-1.3574	0.0227
AT3G25180	-2.5789	0.0002	-2.4379	0.0005	-1.6452	0.0493	-0.6123	0.5938	-0.5513	0.6759	-1.3004	0.1104	-1.5153	0.0289	-2.3750	0.0017	-1.9022	0.0090	-0.8073	0.5052	-2.2330	0.0052	-2.1449	0.0022	-0.8748	0.4196	-1.5308	0.0252
AT3G25250	-1.5751	0.0321	-1.7969	0.0179	-1.3967	0.1210	0.4433	0.7228	-1.4055	0.1227	-1.2209	0.1441	-1.8232	0.0087	-2.0601	0.0099	-1.7719	0.0174	-0.7505	0.5622	-0.2479	0.8676	-1.7560	0.0167	-2.0987	0.0068	-0.9622	0.1751
AT3G25510	-2.5573	0.0000	-2.1739	0.0004	-0.9782	0.2283	-1.8824	0.0123	-0.4201	0.7265	-1.5052	0.0298	-2.6018	0.0000	-1.9201	0.0042	-2.7260	0.0000	-1.1878	0.1531	-0.9089	0.2955	-2.9713	0.0000	-1.7032	0.0119	-2.0488	0.0006
AT3G25610	-1.4305	0.0156	-2.5754	0.0000	-0.8763	0.2660	-0.6943	0.4440	-0.5794	0.5610	-1.3505	0.0403	-1.6263	0.0038	-1.3601	0.0527	-2.3174	0.0001	-0.8485	0.3482	-0.4023	0.6949	-1.7187	0.0031	-1.8157	0.0034	-1.4785	0.0087
AT3G25780	-1.5520	0.0158	-2.0248	0.0015	-1.1043	0.1812	-0.1949	0.8780	-1.1161	0.1870	-1.5064	0.0347	-1.4628	0.0187	-1.7652	0.0132	-2.1915	0.0006	-1.3232	0.1130	0.4697	0.6647	-2.2955	0.0002	-1.7915	0.0096	-1.5305	0.0125
AT3G25882	-1.7608	0.0132	-2.3812	0.0007	-1.5219	0.0743	-1.0880	0.2874	-0.2454	0.8815	-2.1430	0.0058	-1.7913	0.0086	-1.4211	0.1160	-2.1543	0.0025	-0.8586	0.4670	-0.9784	0.3333	-1.6335	0.0242	-1.7792	0.0246	-1.1337	0.0989
AT3G26050	0.4482	0.5165	-0.4116	0.6038	-0.4122	0.6514	-0.0440	0.9742	0.4273	0.6985	-0.6721	0.3500	0.3852	0.5690	0.4798	0.6951	-0.5511	0.4185	-0.4878	0.6704	-0.0714	0.9594	0.5454	0.4283	-0.1807	0.8997	-2.1185	0.0004
AT3G26200	-1.2806	0.0706	-2.6012	0.0001	-1.7620	0.0228	-0.5462	0.6266	-0.5679	0.6483	-1.2848	0.0991	-1.0438	0.1368	-0.6597	0.6183	-2.0380	0.0030	-0.5000	0.7233	-1.1320	0.2233	-1.1910	0.1014	-1.4990	0.0602	-1.7821	0.0063
AT3G26210	-1.7066	0.0013	-1.6906	0.0018	-0.4608	0.5729	-1.2928	0.0650	-0.0754	0.9615	-0.3854	0.5783	-2.1707	0.0000	-1.4322	0.0191	-1.6577	0.0023	-0.9717	0.1957	-1.1872	0.0790	-2.0104	0.0001	-1.2568	0.0431	-0.2638	0.6518
AT3G26320	-1.6263	0.0127	-2.8236	0.0000	-1.5092	0.0489	-1.4664	0.0865	-1.0498	0.2360	-1.7940	0.0131	-1.7499	0.0049	-1.4978	0.0545	-2.5367	0.0001	-0.6268	0.6038	-0.8599	0.3607	-1.2404	0.0697	-1.4564	0.0551	-2.1333	0.0007
AT3G26440	-1.6657	0.0087	-2.0253	0.0014	-1.5114	0.0415	-1.2791	0.1350	-0.6963	0.4944	-1.1533	0.1138	-2.0838	0.0005	-1.9663	0.0042	-1.9223	0.0027	-0.7684	0.4683	-1.2188	0.1431	-1.3951	0.0332	-1.1725	0.1432	-1.0393	0.0920
AT3G26460	2.1150	0.0002	0.1249	0.8965	0.5825	0.5131	0.0392	0.9783	1.0395	0.1893	0.9531	0.1730	1.0780	0.0691	0.7914	0.4134	0.0844	0.9209	1.8574	0.0051	1.1107	0.1607	1.3706	0.0239	0.9221	0.2623	0.2136	0.7533
AT3G26470	-1.6337	0.0007	-2.2103	0.0000	-1.6140	0.0023	-0.5792	0.4388	-0.6928	0.3268	-0.9156	0.1043	-2.2218	0.0000	-1.8806	0.0002	-1.8291	0.0002	-1.4909	0.0078	-1.1265	0.0635	-2.0223	0.0000	-1.5850	0.0024	-1.5193	0.0015
AT3G26480	2.0083	0.0024	0.9413	0.2585	0.7210	0.4838	1.2865	0.1716	0.5278	0.6926	0.1767	NA	1.4582	0.0272	0.7216	0.5774	0.7186	0.3717	1.1331	0.2566	1.1556	0.2216	2.3065	0.0004	0.8152	0.4647	0.1481	0.8587
AT3G26500	-1.7592	0.0012	-2.4775	0.0000	-0.9742	0.1716	-1.0964	0.1431	-0.7573	0.3515	-0.8884	0.1680	-1.8758	0.0003	-1.9110	0.0010	-2.2202	0.0000	-1.2514	0.0727	-1.3706	0.0389	-2.0289	0.0001	-1.9211	0.0007	-1.2467	0.0190
AT3G26570	-0.6551	0.1549	-1.2630	0.0037	-1.0157	0.0429	-1.0258	0.0660	-1.4350	0.0012	-1.7360	0.0001	-0.6805	0.1227	-1.0784	0.0298	-2.0707	0.0000	-1.4434	0.0024	0.1483	0.8676	-0.6094	0.1996	-1.3357	0.0031	-2.4867	0.0000
AT3G26590	-1.4828	0.0117	-2.2926	0.0001	-1.5346	0.0209	-1.3620	0.0760	-0.9240	0.2533	-1.9019	0.0028	-1.6870	0.0025	-1.4069	0.0409	-2.5342	0.0000	-0.8182	0.3698	-0.3895	0.7048	-1.2114	0.0473	-1.4920	0.0225	-1.4554	0.0097
AT3G26740	-0.0198	0.9829	1.1913	0.0714	0.9942	0.2038	0.9803	0.2526	1.0777	0.1692	1.5884	0.0163	0.2344	0.7591	0.9368	0.2866	1.5133	0.0139	1.3774	0.0661	-4.2422	0.0001	0.1804	0.8382	1.5534	0.0190	2.3227	0.0000
AT3G26742	-2.1909	0.0010	-0.3592	0.6897	-1.4418	0.0759	-1.2093	0.1804	-1.9789	0.0074	-0.6664	NA	-2.3056	0.0002	-0.7689	0.4891	-0.5771	0.4459	0.0855	0.9665	-2.2294	0.0037	-1.4971	0.0279	0.6062	0.5730	-0.4140	0.5509
AT3G26830	-2.6937	0.0001	-2.2933	0.0012	-1.2490	0.1745	0.8153	0.4612	-1.4981	0.0855	-0.6599	0.4598	-1.9204	0.0050	-2.1665	0.0051	-1.7966	0.0142	-1.9900	0.0164	-2.4376	0.0016	-2.3765	0.0006	-1.7121	0.0347	-1.1849	0.0865
AT3G26922	-0.5308	0.2428	-2.6608	0.0000	-3.8373	0.0000	-0.2530	0.7241	-2.9314	0.0000	-0.6409	0.2017	-3.8928	0.0000	0.2933	0.7537	-0.3270	0.5079	0.1710	0.8706	0.5024	0.4027	0.1204	0.8414	0.6156	0.2751	-0.5260	0.2171
AT3G27025	-1.3850	0.0349	-2.6507	0.0001	-2.8315	0.0000	-0.6807	0.4905	-2.3474	0.0006	-1.4428	0.0516	-3.0392	0.0000	-0.4988	0.7181	-1.4317	0.0362	-1.1676	0.1925	-0.0617	0.9685	-1.0038	0.1460	-0.8362	0.3865	-1.0711	0.0918
AT3G27170	-2.5268	0.0000	-1.1884	0.0459	-0.9831	0.1631	-1.3996	0.0458	-1.6549	0.0047	-0.9514	0.1342	-2.4794	0.0000	-1.2309	0.0648	-1.6166	0.0036	-0.5007	0.6337	0.2873	0.7784	-2.5091	0.0000	-1.9653	0.0005	-2.0046	0.0001
AT3G27210	0.5624	0.1770	1.1930	0.0020	1.1982	0.0039	0.4147	0.4944	2.2901	0.0000	0.9343	0.0310	0.9352	0.0119	1.1042	0.0094	0.9532	0.0169	1.0838	0.0155	1.5936	0.0001	0.9266	0.0183	1.6333	0.0000	1.8202	0.0000
AT3G27250	-0.3504	0.6840	2.1603	0.0010	1.2629	0.1323	1.1242	0.2344	0.2900	0.8471	0.9213	0.2440	-0.5032	0.5155	0.1638	0.9396	1.3147	0.0612	0.8627	0.4246	1.3324	0.1214	1.5572	0.0220	2.3171	0.0006	1.1397	0.0778
AT3G27350	-1.8564	0.0002	-1.4031	0.0083	-1.4650	0.0102	-0.8463	0.2498	-1.5949	0.0033	-0.4346	0.5033	-2.4001	0.0000	-0.6967	0.3972	-0.5515	0.3450	-0.3083	0.7883	-0.8017	0.2606	-1.6685	0.0009	-0.8608	0.2051	-0.9952	0.0445
AT3G27360	-0.0166	0.9802	-2.7775	0.0000	-3.3429	0.0000	-0.0166	0.9898	-2.9342	0.0000	-0.0090	0.9911	-3.0248	0.0000	0.0830	0.9565	-0.3312	0.5298	-0.1489	0.9033	-0.1565	0.8635	0.7417	0.1126	0.3989	0.6069	-1.1287	0.0100
AT3G27440	-0.5977	0.4963	0.6158	0.5135	0.7875	0.4533	0.5878	0.6193	0.1525	0.9362	0.6313	NA	-0.4480	0.6062	0.5552	0.7272	0.4310	0.6324	0.8370	0.4968	0.0863	0.9615	-0.1016	0.9308	1.2512	0.1796	2.3577	0.0004
AT3G27500	-0.1610	0.8633	-2.9508	0.0000	-2.5147	0.0004	-0.9707	0.3193	-2.3638	0.0008	-0.8586	0.2845	-3.3690	0.0000	0.0990	0.9677	-1.4107	0.0466	-0.8936	0.4009	0.1294	0.9313	-0.1894	0.8466	-0.4152	0.7575	0.0513	0.9480
AT3G27540	-1.3197	0.0153	-1.4054	0.0132	-1.7019	0.0038	-1.0037	0.1787	-1.8029	0.0014	-1.5220	0.0117	-2.0787	0.0000	-1.2692	0.0482	-1.4447	0.0094	-0.5554	0.5672	0.3927	0.6674	-0.6709	0.2704	-1.3073	0.0351	-1.3674	0.0091
AT3G27610	-0.3170	0.3563	-2.5939	0.0000	-2.2273	0.0000	-0.2944	0.5450	-1.6854	0.0000	0.2199	0.5717	-2.1900	0.0000	-0.1946	0.7890	-0.0429	0.9194	-0.0193	0.9830	-0.4448	0.3033	-0.4222	0.2090	-0.4939	0.2318	-0.3545	0.2486
AT3G27620	-0.8780	0.2913	-2.1829	0.0037	-2.1619	0.0081	-0.6258	0.6093	-2.1701	0.0065	-1.0413	NA	-2.3973	0.0007	-0.2302	0.9183	-1.8269	0.0173	-0.6197	0.6724	-0.3489	0.8081	-0.5768	0.5353	-0.6243	0.6492	-1.2917	0.0732
AT3G27940	-1.4480	0.0103	-3.9160	0.0000	-3.8556	0.0000	-1.3144	0.0730	-3.8798	0.0000	0.0426	0.9623	-4.4238	0.0000	-0.5809	0.5688	-0.6981	0.2607	-0.8050	0.3483	-0.6733	0.4164	-1.4396	0.0115	-0.1499	0.9141	0.0101	0.9885
AT3G27990	-0.3618	0.3637	-5.9029	0.0000	-5.8198	0.0000	-0.1332	0.8412	-5.5234	0.0000	-0.2246	0.6229	-6.4400	0.0000	-0.3098	0.6663	0.0371	0.9411	-0.0927	0.9272	0.1741	0.7919	-0.1873	0.6880	-0.1370	0.8666	0.1145	0.7697
AT3G28007	0.2718	0.7151	1.3254	0.0293	0.3255	0.7265	1.0213	0.2006	0.0653	0.9698	1.0872	0.0961	-0.2424	0.7368	0.7851	0.3865	2.2111	0.0001	1.7289	0.0073	-0.4240	0.6693	1.0018	0.0994	0.8829	0.2599	1.3084	0.0168
AT3G28070	-0.5695	0.4873	-1.6559	0.0241	-0.9032	0.3450	-0.7504	0.4875	-0.1924	0.9091	-2.3068	0.0020	-0.1720	0.8522	-0.5445	0.7199	-2.5593	0.0002	-0.2896	0.8702	0.7356	0.4859	-0.1430	0.8927	-0.2822	0.8582	-1.8721	0.0047
AT3G28130	0.0044	0.9970	-2.5108	0.0000	-2.1046	0.0001	0.0099	0.9944	-1.6976	0.0023	-0.8120	0.1897	-2.1620	0.0000	0.1811	0.9058	-0.7385	0.2012	0.0809	0.9629	0.5254	0.5183	0.6034	0.3160	1.0045	0.1213	0.0849	0.8894
AT3G28140	-0.2432	0.5711	-2.1142	0.0000	-1.9640	0.0000	-0.1874	0.7670	-1.5432	0.0000	-0.2955	0.5133	-1.4246	0.0000	-0.4010	0.5232	-0.2800	0.5041	-0.0041	0.9974	-0.3031	0.5999	-0.2127	0.6415	-0.1142	0.8934	0.6976	0.0381
AT3G28170	-1.1993	0.1273	-1.9404	0.0118	-1.9055	0.0236	-0.8968	0.4287	-1.9195	0.0209	-0.5642	NA	-2.2591	0.0013	-0.0489	0.9883	-0.2696	0.7804	-0.0054	0.9992	0.4851	0.7060	-0.5653	0.5377	-0.1401	0.9400	-0.7101	0.3443
AT3G28210	-2.2053	0.0008	-2.2681	0.0007	-1.5131	0.0590	-0.0992	0.9478	-2.0532	0.0040	-0.9408	0.2372	-3.0749	0.0000	-1.6282	0.0392	-0.9046	0.2214	-0.9868	0.3372	-1.4573	0.0846	-2.5626	0.0001	-3.0431	0.0000	-1.9330	0.0027
AT3G28220	-0.5629	0.2780	-2.3206	0.0000	-1.7122	0.0004	-0.3293	0.6778	-1.7949	0.0001	-0.5908	0.2924	-2.1503	0.0000	-1.0783	0.0548	-0.2811	0.6257	-0.3457	0.7207	-0.4229	0.5710	0.1568	0.8164	0.5569	0.4454	-0.7785	0.0869
AT3G28270	-1.0630	0.1819	-3.1405	0.0000	-2.8814	0.0001	-1.5901	0.1048	-2.6534	0.0004	-2.7934	0.0004	-2.5126	0.0003	-1.5544	0.0915	-2.6741	0.0002	0.6732	0.6333	1.0454	0.3192	0.6842	0.4411	-1.6104	0.0632	-2.4098	0.0006
AT3G28290	-0.5263	0.5646	-0.8347	0.3693	-0.8097	0.4556	0.8219	0.4794	-0.8195	0.4958	1.7445	0.0351	-1.0909	0.1597	0.6562	0.6674	1.7480	0.0230	1.4776	0.1337	0.7163	0.5403	1.1676	0.1477	1.1279	0.2722	2.0489	0.0038
AT3G28310	-0.6950	0.4185	3.6292	0.0000	4.1132	0.0000	0.3003	0.8245	2.3639	0.0008	0.9988	0.2428	1.1695	0.0982	-0.3184	0.8750	0.2530	0.7938	1.0529	0.3325	-0.6250	0.6072	-0.3374	0.7380	0.8879	0.4246	0.8524	0.2397
AT3G28320	0.0000	1.0000	7.0553	0.0000	7.5699	0.0000	0.0000	1.0000	5.7782	0.0000	0.0000	1.0000	4.2475	0.0000	0.0000	1.0000	0.0000	1.0000	0.0000	1.0000	0.1252	0.9427	0.0000	1.0000	0.8953	0.4245	0.5371	0.4872
AT3G28345	-0.0594	0.9520	-1.9138	0.0037	-2.5636	0.0001	-0.4919	0.6490	-1.3529	0.0893	-1.1888	0.1104	-1.2042	0.0635	0.2022	0.9172	-1.3072	0.0558	0.6575	0.5743	0.4971	0.6473	-0.0353	0.9755	-0.9581	0.2913	-1.4380	0.0211
AT3G28370	0.7452	0.1679	1.5654	0.0018	1.4089	0.0114	0.4897	0.5451	2.0278	0.0000	1.2181	0.0298	1.5150	0.0013	1.3171	0.0203	1.3313	0.0093	1.1859	0.0560	-0.3565	0.6913	1.1304	0.0283	1.6429	0.0016	0.8087	0.1008
AT3G28510	-1.6908	0.0256	-2.3882	0.0013	-1.5203	0.0970	-1.3883	0.1787	-1.0492	0.3323	-1.8322	0.0271	-2.1957	0.0020	-1.6504	0.0689	-2.4843	0.0008	-0.7992	0.5476	-0.7304	0.5312	-1.7994	0.0180	-0.9311	0.4123	-0.4791	0.5428
AT3G28540	-2.2291	0.0019	-3.0447	0.0000	-2.7199	0.0003	-2.1101	0.0193	-1.6785	0.0523	-2.3525	0.0030	-2.9673	0.0000	-2.1221	0.0083	-3.2400	0.0000	-0.8559	0.4920	-1.4045	0.1431	-1.7618	0.0183	-1.4256	0.1141	-2.2511	0.0012
AT3G28580	-2.7013	0.0001	-2.7943	0.0001	-1.5074	0.0927	-1.4761	0.1350	-0.8359	0.4692	-2.1018	0.0092	-2.4066	0.0005	-2.1072	0.0091	-3.1714	0.0000	-1.3167	0.1957	-1.3656	0.1596	-2.8413	0.0000	-2.0048	0.0119	-2.1342	0.0023
AT3G28740	0.1280	0.8912	1.6440	0.0153	2.0622	0.0029	1.5234	0.0715	1.2419	0.1322	1.0431	0.1669	0.4614	0.5420	0.8747	0.3998	2.0428	0.0016	1.7592	0.0205	0.9971	0.2683	0.2796	0.7564	-0.0826	0.9627	1.4183	0.0224
AT3G28770	-0.8754	0.2046	-2.3067	0.0003	-2.2041	0.0012	0.1504	0.9078	-2.2002	0.0010	0.4980	0.5351	-2.1164	0.0004	-0.3447	0.8333	0.6192	0.3926	-0.0536	0.9788	-1.2344	0.1409	-0.9230	0.1869	-0.5940	0.5920	0.4463	0.4986
AT3G28840	0.2957	0.7457	-2.7560	0.0001	-2.2998	0.0026	-0.3906	0.7519	-2.3421	0.0016	-1.0058	0.2255	-2.9949	0.0000	-0.4587	0.7801	-1.1293	0.1363	-0.6146	0.6482	0.8796	0.3823	0.4273	0.6339	-0.5917	0.6427	-0.6575	0.3559
AT3G28857	-2.3123	0.0001	-1.0082	0.1549	0.0138	0.9912	-0.9629	0.2741	0.2509	0.8538	-0.2404	0.7763	-1.6048	0.0063	-0.0526	0.9815	-1.2380	0.0586	-1.0070	0.2572	0.3140	0.7785	-2.1449	0.0004	-0.2873	0.8313	0.3398	0.5996
AT3G28890	-1.7935	0.0130	-2.4606	0.0005	-1.1253	0.2357	-1.1186	0.2803	-0.0750	0.9729	-1.1307	0.1779	-1.8076	0.0091	-1.2940	0.1842	-2.2858	0.0016	-0.8493	0.4849	-0.8598	0.4204	-1.5493	0.0374	-1.3067	0.1533	-0.4980	0.5070
AT3G28930	-1.8069	0.0034	-3.0268	0.0000	-2.1291	0.0012	-0.7579	0.4295	-1.3991	0.0604	-1.2236	0.0857	-2.3665	0.0000	-0.8941	0.3560	-2.1820	0.0004	-1.2028	0.1531	-1.4806	0.0520	-1.7814	0.0042	-1.2390	0.1055	-2.1120	0.0004
AT3G28940	-1.1370	0.0184	-2.8133	0.0000	-2.3835	0.0000	-0.9011	0.1715	-1.4383	0.0046	-0.6881	0.2210	-2.6173	0.0000	-0.4716	0.6071	-1.2106	0.0135	-0.2834	0.7913	-0.3578	0.6583	-1.1722	0.0157	-0.8027	0.2013	-0.9639	0.0355
AT3G28956	-0.1025	0.8371	-1.6639	0.0000	-1.4587	0.0003	-0.1400	0.8350	-1.0040	0.0181	0.3036	0.5030	-2.1968	0.0000	-0.0128	0.9932	0.4092	0.3050	0.3337	0.6172	0.1225	0.8693	-0.0168	0.9791	-0.3511	0.5838	0.2657	0.4816
AT3G29190	-1.7484	0.0202	-0.2761	0.8038	-1.3933	0.1386	0.4913	0.6957	-1.4017	0.1422	0.4236	NA	-2.0661	0.0037	-0.0745	0.9788	1.1844	0.1330	0.6731	0.6314	-1.2262	0.2278	-1.5762	0.0415	-0.9023	0.4327	0.4208	0.5943
AT3G29250	-0.8591	0.2871	-2.0073	0.0082	-1.5364	0.0855	-0.7407	0.5185	-1.2320	0.2091	-1.0508	NA	-1.9202	0.0062	-1.1220	0.3000	-0.8401	0.3053	-0.2956	0.8751	-0.2407	0.8741	-1.1189	0.1587	-1.1246	0.2628	-0.3590	0.6491
AT3G29370	-2.7890	0.0000	-0.6312	0.3743	-0.4266	0.6284	-0.2364	0.8254	-0.6517	0.4695	0.0897	0.9186	-2.4432	0.0000	-0.2587	0.8674	-0.0764	0.9235	-0.9027	0.2822	-1.5396	0.0221	-2.2598	0.0000	0.1931	0.8866	0.7503	0.1803
AT3G29575	-2.4069	0.0001	-0.2574	0.7821	-0.6176	0.5062	0.7318	0.4532	-2.0723	0.0015	1.2618	0.0771	-3.4371	0.0000	0.1635	0.9330	1.3972	0.0331	0.7112	0.5134	-1.1836	0.1555	-1.7265	0.0061	1.1190	0.1658	1.4165	0.0188
AT3G29590	1.5952	0.0370	-0.4946	0.6278	-0.5379	0.6431	0.9432	0.4058	0.2402	0.8928	-0.7854	0.3901	1.3373	0.0758	0.3866	0.8439	-0.5432	0.5427	0.8839	0.4858	1.2914	0.2005	2.3039	0.0017	-0.4580	0.7642	-0.8950	0.2270
AT3G29639	-1.6660	0.0184	-1.6679	0.0265	-1.9646	0.0134	-0.5063	0.6574	-1.4732	0.0874	0.6025	NA	-2.5089	0.0001	-0.9365	0.3947	-0.9740	0.2018	-0.8080	0.4920	-0.5742	0.6119	-0.9098	0.2373	-0.3247	0.8314	0.0628	0.9386
AT3G29644	-0.0916	0.8826	-4.7497	0.0000	-4.5452	0.0000	0.4208	0.5411	-4.5823	0.0000	0.4824	0.3577	-5.3544	0.0000	0.2797	0.7857	0.5570	0.2421	0.2319	0.8172	0.0661	0.9474	0.0738	0.9135	0.2246	0.8101	1.1846	0.0039
AT3G29670	0.0893	0.8882	-2.8547	0.0000	-2.8033	0.0000	0.4080	0.5670	-3.3221	0.0000	0.4072	0.4629	-2.4658	0.0000	0.0128	0.9947	0.4460	0.3781	-0.0910	0.9484	0.2942	0.7048	-0.1340	0.8371	0.3918	0.6195	1.2573	0.0028
AT3G29760	-1.0712	0.0205	-2.6380	0.0000	-1.9486	0.0000	-0.7711	0.2350	-1.6278	0.0005	-0.8186	0.1202	-2.1008	0.0000	-0.4860	0.5625	-0.9824	0.0402	-0.4672	0.5715	-0.5793	0.3865	-0.8258	0.0889	-0.1851	0.8585	-1.3250	0.0025
AT3G29770	-0.5523	0.1336	-2.2527	0.0000	-1.8771	0.0000	-0.5259	0.2965	-1.6157	0.0000	-0.4572	0.2724	-1.9864	0.0000	-0.3423	0.6061	-0.9073	0.0120	-0.4096	0.4863	0.2667	0.6436	-0.5319	0.1582	-1.1332	0.0024	-0.9208	0.0070
AT3G30180	-2.1149	0.0000	-0.7516	0.0846	-0.2812	0.6440	-1.3659	0.0029	0.0858	0.9321	-1.3119	0.0020	-1.6845	0.0000	0.1169	0.9213	-1.1055	0.0055	-0.3920	0.5847	0.1595	0.8376	-1.8422	0.0000	-0.9683	0.0287	-0.7658	0.0426
AT3G30305	0.2629	0.7919	-1.7779	0.0239	-1.7453	0.0441	-0.3947	0.7673	-1.7583	0.0418	-0.1100	NA	-2.0797	0.0036	-0.0953	0.9713	-1.3167	0.0974	-0.0403	0.9845	-0.4035	0.7674	0.3535	0.7249	-0.8006	0.5141	-0.4132	0.6047
AT3G30720	-1.3891	0.0064	-4.2152	0.0000	-4.0182	0.0000	-1.3755	0.0320	-4.0578	0.0000	-0.2233	0.7477	-4.6658	0.0000	-0.4047	0.7099	-0.6395	0.2568	-1.0005	0.1421	-0.7843	0.2685	-1.4374	0.0049	1.1312	0.0474	-0.3396	0.5262
AT3G32920	-0.9699	0.2113	-2.1914	0.0029	-2.1634	0.0067	-0.6992	0.5374	-2.1757	0.0051	-0.4621	NA	-2.7604	0.0000	-0.1724	0.9395	-2.3236	0.0014	0.2621	0.8888	-0.0544	0.9773	-1.2978	0.0881	-0.2412	0.8866	0.2325	0.7678
AT3G32980	1.1966	0.0151	0.0747	0.9243	-0.2482	0.7613	0.6146	0.4100	-0.1721	0.8810	1.0964	0.0466	0.2296	0.7056	0.8730	0.1914	1.0940	0.0321	1.8263	0.0005	0.9538	0.1369	0.5168	0.3685	1.0329	0.0773	2.0342	0.0000
AT3G42800	-2.0854	0.0000	-1.3505	0.0091	-0.8247	0.2023	-0.5424	0.4794	-1.4657	0.0066	0.0557	0.9420	-2.8088	0.0000	-0.1193	0.9398	-0.2235	0.7215	-0.6761	0.3844	-0.1070	0.9217	-1.7652	0.0003	0.7109	0.2997	0.7181	0.1319
AT3G43190	1.7750	0.0068	1.7033	0.0134	1.3053	0.1136	0.8425	0.4019	2.4610	0.0002	0.4682	0.5895	2.1686	0.0004	0.9571	0.3498	0.9312	0.2021	2.0525	0.0056	-0.2480	0.8587	1.6645	0.0126	0.5554	0.6548	0.2938	0.6937
AT3G43210	-0.8375	0.0138	-2.0128	0.0000	-2.2757	0.0000	-0.2195	0.7035	-2.0227	0.0000	-0.3866	0.3471	-1.6955	0.0000	-0.3877	0.5149	-0.2936	0.4559	-0.2116	0.7739	-0.6731	0.1277	-0.5587	0.1242	-0.4015	0.4498	-1.0888	0.0010
AT3G43250	-2.1827	0.0027	-2.4318	0.0008	-1.8139	0.0332	0.3294	0.8149	-2.4210	0.0013	-0.9724	NA	-2.6121	0.0002	-2.0629	0.0121	-0.9641	0.2405	-1.0640	0.3549	-1.9422	0.0247	-2.5067	0.0004	-1.7535	0.0369	-1.9697	0.0048
AT3G43430	-1.5568	0.0023	0.5005	0.4415	0.3547	0.6569	-0.5350	0.5083	-0.0613	0.9683	-0.2825	0.6802	-1.1357	0.0242	-0.2820	0.8322	-0.4300	0.4764	-0.6785	0.4126	-1.1111	0.0889	-2.0767	0.0000	-0.3416	0.7380	0.5138	0.3199
AT3G43740	-0.3255	0.3825	-6.2921	0.0000	-6.0892	0.0000	-0.1437	0.8141	-6.1285	0.0000	-0.1492	0.7384	-6.8447	0.0000	-0.1566	0.8583	-0.3821	0.2976	-0.0186	0.9841	0.3390	0.4962	-0.2191	0.5985	-0.2118	0.7461	-0.1849	0.5988
AT3G44350	-2.1939	0.0025	-2.1671	0.0037	-1.3705	0.1403	-0.8140	0.4768	-0.0684	0.9773	-1.3237	0.1171	-2.2231	0.0014	-1.2008	0.2518	-2.3130	0.0018	-1.7301	0.0576	-0.4837	0.7070	-2.5887	0.0003	-1.6099	0.0622	-0.9918	0.1698
AT3G44450	-0.9016	0.2589	-1.6620	0.0324	-1.6198	0.0610	-0.5738	0.6318	-1.0016	0.3448	-2.3715	0.0025	-1.0881	0.1466	-0.5830	0.7102	-2.2348	0.0022	0.0354	0.9860	-0.3466	0.8026	0.3527	0.7204	-1.0533	0.3037	-1.8145	0.0090
AT3G44480	-1.1114	0.0001	-3.0034	0.0000	-2.6424	0.0000	-0.7987	0.0359	-2.6438	0.0000	-1.1533	0.0004	-3.3211	0.0000	-1.2009	0.0001	-1.3778	0.0000	-0.6162	0.1217	-0.1709	0.7561	-1.1967	0.0000	-0.6493	0.0707	-1.3078	0.0000
AT3G44490	-0.9436	0.2449	2.4991	0.0002	2.5471	0.0003	-1.0488	0.3353	3.0581	0.0000	-1.0156	0.2467	2.5017	0.0001	-1.1306	0.3059	-1.0829	0.1794	-0.7536	0.5742	-0.1665	0.9197	-0.7976	0.3536	-0.2462	0.8885	-0.4815	0.5387
AT3G44590	0.6987	0.1243	-1.8928	0.0000	-2.4265	0.0000	0.5468	0.4005	-1.3408	0.0040	-0.0348	0.9607	-0.5922	0.1893	0.5129	0.4885	-0.1863	0.7377	0.1490	0.9004	0.8673	0.1164	0.9693	0.0279	0.0323	0.9816	-0.3149	0.4879
AT3G44610	-1.5157	0.0000	-1.6412	0.0000	-1.5078	0.0000	-0.7492	0.0411	-1.5107	0.0000	-0.7374	0.0230	-2.4177	0.0000	-0.4477	0.2934	-0.8652	0.0033	-0.8947	0.0065	-0.2525	0.5882	-1.5616	0.0000	0.1179	0.8578	0.0251	0.9419
AT3G44630	-1.6001	0.0013	-3.7007	0.0000	-3.1918	0.0000	-1.2884	0.0465	-3.1776	0.0000	-1.2148	0.0321	-4.1973	0.0000	-1.6646	0.0020	-2.0879	0.0000	-0.8195	0.2692	0.1466	0.8949	-1.9134	0.0001	-1.2813	0.0242	-2.0974	0.0000
AT3G44660	0.0000	1.0000	2.3162	0.0006	1.7460	0.0243	0.0000	1.0000	2.1531	0.0026	0.0000	NA	1.6224	0.0149	0.0000	1.0000	0.1324	0.8848	0.0000	1.0000	0.0000	1.0000	0.0286	0.9813	0.0000	1.0000	0.0000	1.0000
AT3G44670	-1.4496	0.0001	-3.7355	0.0000	-3.1410	0.0000	-1.2252	0.0084	-3.1082	0.0000	-1.3240	0.0015	-4.3441	0.0000	-1.2793	0.0018	-1.6168	0.0000	-0.6670	0.2218	-0.2146	0.7609	-1.3604	0.0003	-0.8165	0.0761	-1.6587	0.0000
AT3G44713	-0.0170	0.9812	-1.6399	0.0023	-1.4189	0.0174	0.1353	0.8857	-1.2108	0.0471	0.3472	0.5707	-2.3569	0.0000	0.1405	0.9213	0.4726	0.3810	0.3451	0.7225	0.4839	0.5045	0.0411	0.9588	0.3872	0.6595	-0.1959	0.7216
AT3G44720	-1.4893	0.0052	-2.3994	0.0000	-2.6329	0.0000	-0.5958	0.4767	-2.3882	0.0000	-1.6746	0.0042	-2.0867	0.0000	-1.7993	0.0015	-1.9364	0.0003	-1.4374	0.0229	0.3982	0.6616	-1.3623	0.0122	-1.4884	0.0106	-0.7708	0.1425
AT3G44798	-0.6393	0.1671	-4.9499	0.0000	-4.8753	0.0000	0.1773	0.8247	-4.9052	0.0000	0.7877	0.1059	-5.5537	0.0000	-0.1451	0.9080	0.5135	0.2799	0.4897	0.5009	-0.3795	0.5851	-0.5516	0.2541	-0.1620	0.8714	0.7622	0.0643
AT3G44830	-2.2975	0.0016	-2.3025	0.0019	-1.6956	0.0538	1.2367	0.2465	-1.5999	0.0761	-1.6883	NA	-2.0174	0.0048	-2.1675	0.0078	-1.9966	0.0080	-1.9404	0.0281	-1.5338	0.1067	-2.1708	0.0032	-2.1073	0.0079	-1.5306	0.0312
AT3G44860	-1.5544	0.0081	-2.7964	0.0000	-1.4881	0.0271	0.3003	0.7776	-1.5092	0.0227	-1.1679	0.0804	-2.0791	0.0002	-1.9050	0.0023	-1.7979	0.0024	-1.3630	0.0632	-1.6729	0.0134	-1.4031	0.0194	-0.7385	0.4078	-1.3996	0.0131
AT3G44960	0.3768	0.5748	-1.0294	0.1104	-1.5104	0.0228	0.1913	0.8596	-0.5362	0.5817	-0.2724	0.7224	-0.9736	0.0830	0.7223	0.4171	-0.1329	0.8617	0.2156	0.8826	0.1907	0.8680	0.3393	0.6343	-0.3742	0.7380	-2.0565	0.0004
AT3G44990	0.7078	0.4121	-0.4315	0.6772	-0.3870	0.7532	0.5119	0.6869	-0.2616	0.8805	-0.5841	0.5385	0.3213	0.7313	0.3855	0.8439	-1.0700	0.1880	0.0977	0.9681	1.2885	0.2002	2.1036	0.0046	0.4545	0.7660	-0.8777	0.2367
AT3G45000	0.2282	0.8194	2.4493	0.0007	2.3628	0.0024	0.1191	0.9410	2.4975	0.0009	0.2009	NA	2.1527	0.0020	0.4904	0.7754	0.1508	0.8789	0.2727	0.8884	0.1359	0.9348	0.4154	0.6662	0.1872	0.9173	0.2592	0.7438
AT3G45060	-0.8530	0.1471	-0.9216	0.1453	-1.7413	0.0037	0.1160	0.9192	-1.5604	0.0098	-0.3651	0.6081	-2.4353	0.0000	-1.5886	0.0095	-0.1041	0.8906	-0.1628	0.9170	-1.1944	0.0866	-1.2698	0.0251	-2.7275	0.0000	-1.6436	0.0021
AT3G45130	-1.9906	0.0029	-2.4588	0.0002	-1.6711	0.0326	-0.7169	0.4905	-0.7406	0.4886	-1.4006	0.0676	-1.3952	0.0335	-2.0670	0.0050	-0.6692	0.3820	-0.6573	0.5931	-2.0111	0.0096	-1.7353	0.0108	-2.2210	0.0020	-0.5030	0.4691
AT3G45440	-0.8069	0.2045	2.1192	0.0000	2.2288	0.0000	-0.1031	0.9328	2.4285	0.0000	-0.3357	0.6686	2.1743	0.0000	-0.2620	0.8657	-0.2315	0.7601	-0.6183	0.5591	0.6199	0.4636	-0.1306	0.8791	0.1031	0.9443	-0.9022	0.1380
AT3G45443	-0.1370	0.6548	-3.2963	0.0000	-3.2959	0.0000	0.1805	0.6512	-2.6271	0.0000	0.5739	0.0329	-3.0501	0.0000	0.2659	0.5352	0.5291	0.0347	0.4095	0.2274	0.0646	0.9054	0.1760	0.5576	-0.1108	0.8431	0.0680	0.8092
AT3G45638	-0.7209	0.2747	-0.8156	0.2651	-1.1781	0.1342	0.3864	0.6940	-1.1546	0.1496	0.4867	0.5100	-2.1542	0.0002	0.2386	0.8816	0.4463	0.5161	0.5260	0.6333	0.8300	0.3069	-0.0357	0.9718	0.2329	0.8636	1.6629	0.0022
AT3G45640	-1.6519	0.0000	-1.8409	0.0000	-1.2834	0.0000	-1.0350	0.0014	-1.0664	0.0002	-0.9707	0.0015	-2.0313	0.0000	-1.2079	0.0000	-1.3896	0.0000	-1.0670	0.0004	0.1419	0.7896	-1.8502	0.0000	-1.0546	0.0002	-0.9854	0.0002
AT3G45660	-0.4309	0.6359	-0.2043	0.8544	0.0762	0.9593	1.1083	0.2756	0.6209	0.6279	-0.2240	0.8328	-0.1627	0.8642	-0.2229	0.9183	-0.5500	0.5259	0.2098	0.9185	3.2908	0.0000	0.0488	0.9687	-0.2226	0.9007	-0.5348	0.4843
AT3G45730	-1.6612	0.0094	-1.2126	0.0889	-0.7499	0.4091	0.0923	0.9484	-1.8203	0.0086	1.0341	0.1635	-3.0875	0.0000	-0.0475	0.9864	0.5753	0.4376	0.3206	0.8308	-0.9861	0.2669	-1.8074	0.0045	-0.4857	0.6898	1.4770	0.0157
AT3G45860	-1.8613	0.0040	-3.6571	0.0000	-2.0594	0.0033	-2.3986	0.0013	-0.6590	0.5473	-1.2256	0.1009	-2.5303	0.0000	-1.2037	0.1689	-2.8861	0.0000	-1.6329	0.0388	-0.6237	0.5441	-2.1760	0.0006	-1.9775	0.0043	-1.8772	0.0026
AT3G45960	1.0557	0.1132	0.6444	0.4284	0.3305	0.7589	1.4145	0.0867	-0.2292	0.8827	2.0532	0.0024	-0.5950	0.4132	1.6873	0.0162	1.2201	0.0697	1.4376	0.0687	0.3276	0.7897	0.5213	0.5080	1.2803	0.0986	1.3102	0.0330
AT3G45970	2.1636	0.0000	0.9169	0.1358	0.9946	0.1455	0.4764	0.5861	0.2033	0.8699	1.2374	0.0398	0.6475	0.2600	0.4920	0.6512	1.1198	0.0470	1.0031	0.1738	0.2600	0.8012	0.9360	0.1035	1.0866	0.0962	1.4612	0.0045
AT3G46030	-0.0116	0.9791	-2.2536	0.0000	-2.4205	0.0000	-0.4233	0.3089	-1.6934	0.0000	-0.1234	0.7536	-1.9559	0.0000	-0.0385	0.9693	-0.3271	0.3074	-0.1528	0.8179	-0.6582	0.0603	0.0538	0.9023	-0.4255	0.2842	-0.8834	0.0012
AT3G46080	-2.5592	0.0001	-2.5103	0.0001	-2.3616	0.0007	-0.1378	0.9201	-2.4650	0.0002	-1.4270	0.0567	-2.5857	0.0000	-2.5300	0.0002	-1.9119	0.0045	-1.1752	0.2044	-1.8447	0.0167	-2.7647	0.0000	-2.6911	0.0001	-1.7786	0.0054
AT3G46090	-1.6293	0.0252	-2.8288	0.0000	-1.7581	0.0340	-1.0029	0.3446	-1.1200	0.2593	-2.1313	0.0068	-1.8671	0.0068	-2.4373	0.0012	-3.1493	0.0000	-2.2086	0.0065	-0.4041	0.7561	-1.8298	0.0117	-2.3140	0.0020	-2.1034	0.0022
AT3G46110	-1.0797	0.0725	-2.1059	0.0002	-1.0605	0.1496	-1.4457	0.0510	-1.0137	0.1855	-1.6459	0.0101	-1.4709	0.0083	-1.2700	0.0726	-2.0038	0.0005	-0.8755	0.3132	-0.8141	0.3230	-1.4110	0.0165	-1.6865	0.0067	-2.0550	0.0002
AT3G46130	-1.4659	0.0382	-2.6254	0.0001	-2.3755	0.0010	-1.4881	0.1050	-1.3216	0.1335	-2.3752	0.0015	-1.9437	0.0032	-0.6574	0.6279	-2.4492	0.0004	-0.2149	0.9120	-0.0462	0.9805	-1.0640	0.1575	-1.0307	0.2839	-2.4160	0.0002
AT3G46280	-2.3793	0.0000	-2.7868	0.0000	-1.5925	0.0011	-1.7485	0.0010	-3.0926	0.0000	-2.7774	0.0000	-4.1504	0.0000	-2.6999	0.0000	-2.4773	0.0000	-2.6482	0.0000	-3.2420	0.0000	-2.8138	0.0000	-2.5891	0.0000	-1.8535	0.0000
AT3G46600	-1.5765	0.0000	-1.4554	0.0000	-0.8177	0.0175	-0.6108	0.1388	-0.9655	0.0026	-0.4459	0.2153	-2.0161	0.0000	-0.9397	0.0045	-0.9078	0.0032	-0.6811	0.0784	0.2383	0.6410	-1.7433	0.0000	-0.8802	0.0074	-0.9881	0.0007
AT3G46640	1.6342	0.0014	1.4906	0.0052	1.6628	0.0029	1.2393	0.0681	1.8187	0.0006	1.4573	0.0116	1.7327	0.0004	1.8956	0.0004	2.5406	0.0000	2.4885	0.0000	-0.4375	0.6142	1.6477	0.0013	0.9996	0.1248	1.9535	0.0001
AT3G46690	-1.6599	0.0023	-1.1529	0.0559	-0.7065	0.3545	-0.3508	0.7104	-1.6779	0.0045	0.7847	0.2225	-2.3178	0.0000	-0.6302	0.5167	0.3085	0.6495	0.0592	0.9735	-2.0474	0.0006	-1.8395	0.0006	-1.1589	0.0804	0.6480	0.2333
AT3G46845	-0.3039	0.5161	-5.5108	0.0000	-5.3282	0.0000	-0.0757	0.9277	-5.4601	0.0000	0.1736	0.7462	-5.9043	0.0000	-0.4523	0.5114	-0.1091	0.8360	0.2264	0.7963	1.1224	0.0111	-0.0103	0.9885	-0.2486	0.7539	0.4485	0.2484
AT3G46870	-0.6864	0.2954	-1.3085	0.0410	-1.5805	0.0187	-0.5425	0.5681	-1.1210	0.1397	-1.8888	0.0039	-0.7775	0.2083	-0.8494	0.3505	-1.6110	0.0081	-0.3774	0.7689	-0.5033	0.5993	-0.4885	0.4981	-0.6949	0.4543	-2.4505	0.0000
AT3G46880	-1.5607	0.0287	-0.9730	0.2421	-1.7531	0.0321	0.1319	0.9281	-1.3151	0.1460	-1.1005	NA	-1.9611	0.0033	-0.4706	0.7695	-1.5341	0.0381	-2.0466	0.0128	-0.0395	0.9840	-1.6910	0.0183	-1.2580	0.1642	-2.0292	0.0031
AT3G46970	1.8843	0.0004	0.2918	0.7096	1.2784	0.0485	0.5199	0.5573	2.1617	0.0001	-0.2929	0.6922	1.5193	0.0040	1.6019	0.0081	0.0563	0.9437	0.5979	0.5408	-0.1326	0.9144	2.5092	0.0000	2.6059	0.0000	-0.0263	0.9688
AT3G46980	-0.0255	0.9337	-2.0805	0.0000	-2.1756	0.0000	-0.0661	0.8736	-1.9837	0.0000	-0.2658	0.2955	-1.9063	0.0000	0.0153	0.9865	-0.1471	0.5615	-0.1832	0.6510	-0.4407	0.1030	0.1066	0.7029	-0.0361	0.9468	-0.2173	0.3103
AT3G47010	-0.7371	0.0286	-3.9923	0.0000	-4.2138	0.0000	-0.3413	0.5112	-3.5596	0.0000	-0.0030	0.9965	-4.6661	0.0000	-0.4365	0.4165	0.0207	0.9671	-0.3584	0.5407	-0.9441	0.0142	-0.9294	0.0049	-0.8193	0.0298	-0.7409	0.0212
AT3G47030	-0.1871	0.8594	-1.8904	0.0144	-1.8595	0.0280	-0.6122	0.6168	-1.8719	0.0253	-0.9817	NA	-2.1732	0.0022	-0.3848	0.8439	-0.6421	NA	-0.5655	0.7116	-1.0672	0.3128	0.0365	0.9783	-0.3801	0.8146	-1.4762	NA
AT3G47070	-1.1667	0.0026	-1.2376	0.0017	-1.1382	0.0087	-1.0543	0.0323	-0.8242	0.0870	-1.2338	0.0039	-0.9395	0.0141	-0.7290	0.1652	-1.4132	0.0003	-1.2238	0.0058	-0.4426	0.4562	-0.9332	0.0204	-1.2595	0.0022	-2.5521	0.0000
AT3G47090	-0.6482	0.3696	-1.8508	0.0042	-1.0784	0.1908	-0.5865	0.5661	-0.2291	0.8760	-1.2801	0.0754	-0.9921	0.1271	-1.1555	0.1762	-2.0553	0.0012	-0.5776	0.6333	-0.1958	0.8848	-1.0109	0.1412	-1.6885	0.0156	-1.6909	0.0055
AT3G47130	-0.6159	NA	-1.8189	0.0183	-1.7912	0.0340	-0.8928	NA	-1.8023	0.0319	-0.2609	NA	-2.0731	0.0036	-0.5630	NA	-0.9873	NA	-1.0517	NA	-0.4739	0.7199	-1.3876	0.0782	-0.3696	0.8219	-1.0252	NA
AT3G47160	1.0872	0.0232	1.3383	0.0056	1.8580	0.0001	1.0745	0.0837	2.3219	0.0000	1.8108	0.0003	1.3026	0.0040	1.6299	0.0010	1.6485	0.0004	1.1999	0.0348	-1.2560	0.0254	1.2557	0.0082	1.8014	0.0001	1.8516	0.0000
AT3G47220	-1.6103	0.0214	-2.0931	0.0033	-2.1903	0.0040	-1.7272	0.0517	-1.2151	0.1841	-1.5039	0.0566	-2.1238	0.0012	0.0477	0.9874	-1.6273	0.0250	-1.3030	0.1661	-0.7271	0.4885	-2.0571	0.0029	-0.5743	0.6477	-0.2261	0.7665
AT3G47250	-1.3186	0.0000	-9.3018	0.0000	-8.9687	0.0000	-0.7659	0.0014	-9.0124	0.0000	-0.2226	0.3854	-9.7560	0.0000	-1.0031	0.0000	-0.4380	0.0461	-0.3211	0.3040	-0.3722	0.1862	-1.4473	0.0000	-0.9849	0.0000	-0.7315	0.0002
AT3G47290	-0.6321	0.1545	-7.4392	0.0000	-7.2635	0.0000	-0.6910	0.2421	-6.8794	0.0000	-0.7826	0.0994	-7.4213	0.0000	-0.3179	0.7276	-1.1661	0.0056	-0.4933	0.4818	-0.0524	0.9584	-0.5441	0.2419	-0.5302	0.3957	-1.4751	0.0002
AT3G47295	-1.2733	0.0134	0.0890	0.9127	-0.6916	0.3258	0.4789	0.5584	-1.0091	0.1181	0.6411	0.2935	-2.0968	0.0000	-0.2029	0.8870	1.0813	0.0424	0.4212	0.6738	-0.2824	0.7629	-1.1991	0.0223	0.0881	0.9467	0.7523	0.1309
AT3G47347	-0.8989	0.2324	-2.5251	0.0003	-2.3369	0.0020	0.4484	0.6992	-2.3485	0.0014	-0.2509	0.7955	-2.6701	0.0000	0.3869	0.8225	0.0079	0.9947	0.2175	0.9089	0.7328	0.4780	-0.3508	0.7032	-0.6319	0.6048	1.3927	0.0318
AT3G47348	-0.9716	0.1402	-3.8800	0.0000	-3.4514	0.0000	-0.0429	0.9763	-3.8517	0.0000	-0.2397	0.7795	-4.3631	0.0000	0.2297	0.8972	-0.3978	0.5972	-0.0832	0.9665	0.6539	0.4853	-0.3551	0.6607	-0.4947	0.6670	0.5654	0.3651

AT3G47350	0.4136	0.2751	-7.4931	0.0000	-7.4857	0.0000	0.0018	0.9991	-7.7635	0.0000	-0.2235	0.6144	-8.0501	0.0000	0.0034	0.9979	-0.3452	0.3799	0.0864	0.9316	0.8142	0.0507	0.3527	0.3816	-0.0646	0.9407	0.5538	0.0959
AT3G47360	0.1929	0.6566	-5.1324	0.0000	-5.1764	0.0000	-0.1087	0.8743	-4.9730	0.0000	0.1150	0.8164	-5.9069	0.0000	-0.3453	0.6028	-0.0941	0.8414	0.1316	0.8859	-0.1144	0.8759	0.2195	0.6204	-0.5944	0.2011	-0.4768	0.1720
AT3G47430	-2.0569	0.0000	-1.8933	0.0001	-2.1605	0.0000	-1.4559	0.0219	-2.2652	0.0000	-2.0555	0.0002	-1.8101	0.0002	-2.2339	0.0000	-2.0744	0.0000	-0.6695	0.4163	0.0324	0.9818	-1.2826	0.0135	-1.7994	0.0006	-1.9082	0.0001
AT3G47480	-2.1955	0.0023	-2.1710	0.0034	-1.2510	0.1882	-1.3380	0.1883	-0.4621	0.7522	-1.7640	0.0307	-1.9401	0.0058	-2.0585	0.0113	-2.6722	0.0002	-1.1326	0.2966	-1.4804	0.1156	-2.2282	0.0020	-1.8261	0.0261	-1.3268	0.0602
AT3G47500	-1.6562	0.0000	-1.6355	0.0000	-1.6487	0.0000	-0.7733	0.1654	-1.8710	0.0000	-0.6785	0.1484	-2.7173	0.0000	-0.6888	0.2245	-0.5352	0.2297	-0.1509	0.8888	-0.2668	0.7074	-1.3211	0.0008	-0.7077	0.1757	-0.8537	0.0279
AT3G47540	-2.2656	0.0000	-2.9967	0.0000	-2.1481	0.0003	-1.0334	0.2074	-1.1980	0.0962	-2.0568	0.0011	-1.6873	0.0024	-1.7055	0.0079	-2.3380	0.0000	-1.6474	0.0153	-1.4235	0.0433	-2.3265	0.0000	-1.9259	0.0016	-1.9672	0.0004
AT3G47675	-0.6475	0.0951	-5.5811	0.0000	-5.4998	0.0000	0.1089	0.8819	-5.5324	0.0000	0.5128	0.2372	-6.2156	0.0000	0.2803	0.7281	0.2501	0.5734	0.3008	0.6764	-0.1399	0.8511	-0.2393	0.6140	0.0948	0.9190	0.2845	0.4558
AT3G47680	-0.9873	0.0154	-7.1350	0.0000	-7.0590	0.0000	-0.7595	0.1742	-6.8870	0.0000	-0.4706	0.3363	-7.7767	0.0000	-0.2658	0.7816	-0.6411	0.1423	-0.1884	0.8468	-0.5602	0.3340	-0.8266	0.0505	-0.3605	0.6166	-0.5997	0.1303
AT3G47965	-0.5921	0.2606	-3.6068	0.0000	-3.6352	0.0000	0.4230	0.5775	-3.5397	0.0000	0.6805	0.2194	-4.4370	0.0000	0.1943	0.8816	0.7461	0.1397	1.0589	0.0699	0.2306	0.7986	-0.3846	0.5109	0.6875	0.2990	1.4766	0.0009
AT3G48020	-0.7869	0.2816	0.0461	0.9670	-0.4961	0.6265	-0.1190	0.9329	0.1319	0.9384	-0.2121	0.8228	-0.4166	0.5898	-0.7215	0.5444	0.3132	0.7053	0.0061	0.9983	-0.1073	0.9441	-0.2315	0.8037	-0.1347	0.9356	2.8477	0.0000
AT3G48080	-1.8483	0.0099	-4.4325	0.0000	-4.4321	0.0000	-1.8933	0.0349	-4.3773	0.0000	-1.6711	0.0364	-4.6963	0.0000	-1.6853	0.0468	-2.3055	0.0013	-1.1987	0.2415	-1.2666	0.1874	-2.1217	0.0027	-1.5867	0.0574	-1.4564	0.0338
AT3G48115	-0.6771	0.2302	-0.0481	0.9553	-0.1761	0.8450	0.2880	0.7533	-0.7392	0.3210	0.3058	0.6517	-0.7190	0.1817	-0.3045	0.8127	0.3253	0.6056	0.4390	0.6616	0.8179	0.2490	-0.5141	0.4001	0.2550	0.8219	2.1182	0.0000
AT3G48200	-0.7601	0.0690	-0.7749	0.0827	-0.6419	0.2250	-0.5946	0.3109	-0.9978	0.0301	-1.3389	0.0020	-0.7497	0.0627	-0.9328	0.0519	-1.2380	0.0022	-0.5112	0.4360	0.2728	0.6983	-0.6228	0.1551	-0.6200	0.2605	-2.0697	0.0000
AT3G48240	-1.9360	0.0044	-0.3491	0.7148	-0.6861	0.4889	-0.4659	0.6814	-1.4352	0.0868	-0.8464	0.2994	-2.0348	0.0016	-0.9419	0.3748	-0.4632	0.5686	-0.1876	0.9222	-0.4745	0.6843	-0.9233	0.2171	0.3973	0.7728	0.2084	0.7801
AT3G48260	-1.8760	0.0004	-0.7016	0.2784	0.3485	0.6763	-0.4091	0.6439	-1.0732	0.1125	0.5940	0.3541	-3.3711	0.0000	0.2480	0.8614	-0.2415	0.7225	-0.3840	0.7292	0.1736	0.8762	-1.9631	0.0002	0.9416	0.1631	0.5753	0.2776
AT3G48280	-0.2054	0.8287	-0.6733	0.4397	-0.6054	0.5585	-0.6749	0.5387	-0.6113	0.6157	-2.0972	0.0060	-0.6839	0.3648	-0.7406	0.5532	-2.2395	0.0013	-0.8959	0.4191	0.1762	0.9083	-0.1588	0.8786	-1.3433	0.1156	-2.1028	0.0016
AT3G48290	-0.5335	0.5505	-2.3877	0.0012	-2.3627	0.0029	-0.4816	0.7030	-2.3725	0.0021	-1.1639	0.1778	-2.6476	0.0001	0.2774	0.8959	-1.3772	0.0796	-0.7854	0.5508	0.3147	0.8279	0.1947	0.8596	-0.3875	0.8069	-1.0217	0.1598
AT3G48310	-2.0995	0.0000	0.2101	0.6641	0.1806	0.7543	-0.8547	0.0585	-0.6994	0.1103	-0.6710	0.0936	-1.5736	0.0000	-1.3865	0.0001	-0.7006	0.0577	-0.6911	0.1383	-1.0806	0.0059	-1.7715	0.0000	-1.1679	0.0013	-1.2856	0.0001
AT3G48390	-0.5311	0.5473	1.5257	0.0546	1.5201	0.0863	1.6328	0.0887	-0.1859	0.9180	2.0973	0.0088	-0.4886	0.5694	1.1482	0.2794	2.2024	0.0026	1.5549	0.0973	-0.6661	0.5636	-0.5495	0.5461	0.5049	0.7218	1.4482	0.0381
AT3G48420	-0.1136	0.8513	-0.1812	0.7738	0.0580	0.9460	-0.4480	0.5052	0.0779	0.9459	-0.6277	0.2123	0.2852	0.5720	-0.3332	0.7220	-0.8248	0.0683	-0.9373	0.0852	-0.4587	0.4819	-0.2260	0.6940	-0.9326	0.0637	-2.5580	0.0000
AT3G48510	0.2695	0.7866	2.1679	0.0032	0.1808	0.8974	0.8474	0.4561	0.5551	0.6918	0.5017	NA	0.5566	0.5103	-0.0128	0.9963	1.0272	0.2028	0.3966	0.8183	0.1173	0.9460	1.6563	0.0270	1.3081	0.1657	0.7653	0.3045
AT3G48630	-2.4748	0.0004	-2.1838	0.0029	-1.0755	0.2685	-1.4789	0.1297	-0.0105	0.9998	-1.4140	0.0870	-1.5126	0.0343	-2.6614	0.0004	-2.7640	0.0001	-1.5104	0.1104	-1.2744	0.1909	-2.7692	0.0001	-2.3094	0.0025	-1.1285	0.1104
AT3G48640	-2.7507	0.0001	-2.9663	0.0000	-1.8685	0.0235	-0.9361	0.3929	-1.1750	0.2343	-2.0121	0.0120	-2.3533	0.0006	-2.4597	0.0013	-3.0468	0.0000	-2.2375	0.0064	-1.1904	0.2300	-2.9293	0.0000	-2.6965	0.0002	-1.8732	0.0070
AT3G48850	-1.6151	0.0225	-1.1615	0.1474	-0.7828	0.4349	1.4759	0.1105	-0.7757	0.4865	-1.0635	0.1936	-1.4458	0.0356	-1.6319	0.0499	-1.3395	0.0704	-1.2053	0.2220	-1.5451	0.0774	-1.4919	0.0396	-2.1665	0.0036	-0.6184	0.3845
AT3G48920	1.2272	0.1198	0.6348	0.5123	0.5434	0.6384	0.7192	0.5476	2.0177	0.0137	0.0101	NA	0.8274	0.3051	0.7457	0.6007	0.6856	0.4281	1.6450	0.0816	1.3407	0.1787	1.9599	0.0088	1.3197	0.1687	1.7797	0.0126
AT3G49055	-1.4604	0.0450	-1.7512	0.0204	-1.0061	0.2961	-1.1765	0.2438	-0.8431	0.4417	-1.4072	0.0837	-2.5131	0.0002	-1.2640	0.1908	-1.3585	0.0719	-0.7277	0.5683	0.1147	0.9441	-1.7015	0.0191	-0.9472	0.3661	-1.6010	0.0202
AT3G49160	-4.1691	0.0000	-1.4625	0.0001	-0.9787	0.0223	-1.0446	0.0259	-1.5459	0.0000	-0.6395	0.1430	-5.7293	0.0000	-0.9170	0.0373	-0.8723	0.0259	-0.6806	0.1931	-0.4402	0.4333	-5.1261	0.0000	-0.7212	0.1241	-1.2316	0.0006
AT3G49230	0.1332	0.9001	2.5199	0.0005	2.0527	0.0120	-0.1151	0.9442	2.7000	0.0002	0.0000	NA	3.1705	0.0000	0.2356	0.9141	0.0000	1.0000	0.0000	1.0000	0.5125	0.6884	0.3858	0.6971	0.4058	0.7941	1.2189	0.0877
AT3G49601	-1.5210	0.0055	-1.0978	0.0710	-1.2036	0.0690	-0.8753	0.2699	-1.2091	0.0679	0.1079	0.8980	-2.0945	0.0000	-0.4916	0.6622	-0.2830	0.6821	-0.5492	0.5869	-1.4508	0.0251	-1.4243	0.0105	-0.5205	0.5853	-0.9004	0.0924
AT3G49620	-1.9833	0.0075	-0.3412	0.7527	0.0694	0.9649	0.9695	0.3869	-1.6688	0.0595	1.4466	0.0873	-2.0211	0.0046	0.4126	0.8291	1.6622	0.0318	1.3489	0.1921	-1.7146	0.0603	-1.9057	0.0113	-1.1816	0.2392	0.4190	0.5988
AT3G49970	-2.1068	0.0001	-0.1512	0.8527	0.5199	0.5017	-1.3635	0.0517	0.5519	0.5197	-1.0567	0.0966	-0.8845	0.1007	-1.3775	0.0302	-1.6734	0.0036	-1.0777	0.1404	-1.3077	0.0549	-2.6283	0.0000	0.3787	0.7110	-0.7021	0.1988
AT3G49980	-1.8275	0.0121	-1.4003	0.0818	-0.8703	0.3904	-1.3392	0.1784	-1.1944	0.2219	-0.7218	NA	-2.0511	0.0029	-0.4328	0.8038	-1.3425	0.0817	-1.2298	0.2308	-1.7551	0.0469	-1.5395	0.0400	-0.2911	0.8582	-0.7228	0.3207
AT3G50010	1.4686	0.0240	-2.0329	0.0041	-2.1215	0.0052	-0.6367	0.5493	-1.3542	0.1143	-0.1145	0.9103	-1.1681	0.0805	0.3229	0.8532	-0.5391	0.4933	0.4969	0.7089	0.5399	0.6147	0.9466	0.1819	-0.2541	0.8695	0.1496	0.8438
AT3G50120	0.5043	0.5543	0.5946	0.5160	2.0000	0.0084	0.3203	0.8069	1.5918	0.0518	0.6863	0.4352	0.7262	0.3374	1.1906	0.2184	1.3513	0.0661	1.5689	0.0715	-0.0532	0.9778	1.0159	0.1837	2.4263	0.0005	2.8440	0.0000
AT3G50140	0.3496	0.6545	-2.3956	0.0002	-0.8726	0.3094	-1.1060	0.2098	-0.6036	0.5740	-0.9970	0.1774	-0.5789	0.4005	-0.3671	0.8127	-1.6358	0.0128	-1.4508	0.0681	-0.2506	0.8402	-0.2781	0.7453	-1.0005	0.2428	0.1351	0.8500
AT3G50280	-1.3917	0.0638	-1.8171	0.0175	-2.0353	0.0122	-0.4150	0.7448	-1.2338	0.2031	-2.1537	0.0073	-1.5549	0.0286	-1.6902	0.0506	-1.6227	0.0323	-0.2483	0.9011	0.2180	0.8878	-1.3713	0.0727	-1.3124	0.1567	-1.5566	0.0260
AT3G50390	-0.8949	0.2397	-1.7801	0.0186	-1.9490	0.0160	-0.2023	0.8818	-2.1849	0.0043	-0.8325	NA	-1.8580	0.0061	-0.3919	0.8210	-0.6148	0.4509	-0.8322	0.4834	-0.2644	0.8495	-0.6401	0.4393	0.3147	0.8357	-0.1895	0.8097
AT3G50470	-0.5481	0.4571	-2.6109	0.0000	-1.3538	0.0740	-1.0914	0.2165	-0.5725	0.6024	-0.6816	0.3728	-0.9907	0.1225	-1.2949	0.1022	-2.2050	0.0004	-1.3577	0.0913	-1.0782	0.2039	-1.0244	0.1301	-1.6887	0.0144	-1.3167	0.0293
AT3G50480	-2.0147	0.0007	-7.8681	0.0000	-7.8395	0.0000	-1.8722	0.0117	-6.9747	0.0000	-1.2383	0.0739	-7.6031	0.0000	-2.0894	0.0011	-2.4400	0.0000	-1.5513	0.0350	-1.5428	0.0343	-2.6768	0.0000	-2.5866	0.0000	-2.0172	0.0005
AT3G50700	1.0297	0.0089	1.7044	0.0000	2.1833	0.0000	1.3034	0.0051	2.1464	0.0000	1.8887	0.0000	1.4027	0.0001	1.6492	0.0000	2.1218	0.0000	1.3722	0.0014	0.1623	0.8371	1.2090	0.0018	1.7032	0.0000	1.8155	0.0000
AT3G50770	-2.4787	0.0002	-3.1256	0.0000	-1.2324	0.1693	-1.9406	0.0253	-0.4840	0.7206	-1.8371	0.0172	-2.5507	0.0001	-2.1529	0.0043	-3.1442	0.0000	-0.8561	0.4605	-1.1740	0.2159	-2.5262	0.0002	-1.7111	0.0297	-1.7990	0.0071
AT3G50800	-1.9244	0.0003	-0.8648	0.1689	-0.9269	0.1883	-0.9186	0.2316	-0.4555	0.6440	-1.8369	0.0019	-2.3872	0.0000	-1.2913	0.0438	-1.4568	0.0088	-1.9795	0.0008	1.3184	0.0410	-1.8087	0.0006	-0.3620	0.7357	-1.1149	0.0332
AT3G50840	-1.0711	0.0275	-1.8474	0.0001	-1.4670	0.0048	-0.2668	0.7529	-2.0826	0.0000	0.7545	0.1732	-2.4524	0.0000	-0.2856	0.8098	0.1256	0.8455	-0.1081	0.9400	-0.4420	0.5580	-0.3442	0.5672	0.9746	0.0889	0.4353	0.3681
AT3G50900	-1.6282	0.0028	-1.9979	0.0002	-1.2153	0.0658	-0.7699	0.3443	-0.9222	0.2124	-0.5271	0.4423	-2.0619	0.0001	-0.5116	0.6408	-0.9761	0.0970	-0.5013	0.6333	-0.3995	0.6719	-1.6838	0.0019	-0.8039	0.3006	-0.8304	0.1231
AT3G50930	-2.2627	0.0001	-2.1763	0.0003	-1.6584	0.0161	-0.4519	0.6603	-1.3890	0.0568	-1.6815	0.0131	-2.6850	0.0000	-2.2041	0.0005	-2.2621	0.0002	-1.3606	0.0804	0.1145	0.9354	-2.7411	0.0000	-2.2189	0.0004	-1.7673	0.0026
AT3G50970	-0.0286	0.9794	0.9087	0.2958	0.3820	0.7446	0.7400	0.5107	0.2292	0.8929	1.3427	0.1000	-0.8625	0.2586	1.5644	0.0733	2.4936	0.0004	2.3402	0.0032	0.1070	0.9485	2.1451	0.0023	2.9695	0.0000	2.4144	0.0004
AT3G50980	-0.0586	0.9580	2.4974	0.0006	0.9797	0.3324	-0.2881	0.8337	0.8382	0.4683	0.1685	NA	0.7434	0.3582	0.2296	0.9167	0.9379	0.2474	0.7068	0.6063	1.3227	0.1776	1.4782	0.0551	0.1437	0.9373	0.4555	0.5538
AT3G51330	-2.3609	0.0000	-1.6087	0.0000	-1.5730	0.0000	-1.6360	0.0000	-1.0944	0.0054	-1.2172	0.0023	-1.8563	0.0000	-2.0960	0.0000	-1.6702	0.0000	-1.5079	0.0001	-1.7765	0.0000	-2.2677	0.0000	-1.5146	0.0000	-0.8777	0.0132
AT3G51340	-1.3108	0.0002	-3.5483	0.0000	-3.0541	0.0000	-1.6956	0.0000	-2.3086	0.0000	-1.7015	0.0000	-3.3263	0.0000	-1.4509	0.0001	-1.7738	0.0000	-1.8609	0.0000	-1.5482	0.0000	-1.5807	0.0000	-1.4336	0.0001	-1.8714	0.0000
AT3G51350	-1.2984	0.0292	-2.6189	0.0000	-2.5475	0.0001	-0.5545	0.5417	-2.5664	0.0000	-0.9240	0.1831	-3.1012	0.0000	-0.3202	0.8241	-0.6490	0.3225	-0.4692	0.6764	-0.2399	0.8323	-0.9857	0.1103	-0.6677	0.4746	-0.7152	0.2247
AT3G51400	1.4333	0.0199	1.0176	0.1522	1.6150	0.0191	1.2059	0.1464	2.2688	0.0002	1.2662	0.0701	2.2313	0.0001	2.0115	0.0017	1.4657	0.0210	1.4086	0.0658	-0.2511	0.8454	1.4649	0.0183	1.8495	0.0042	3.5956	0.0000
AT3G51540	-1.6262	0.0201	-0.8275	0.3278	-0.4386	0.6876	-0.0807	0.9578	-1.2653	0.1588	0.3566	0.6984	-2.7397	0.0000	-0.4876	0.7586	0.1046	0.9124	-0.1110	0.9608	-1.5921	0.0626	-1.7643	0.0119	-0.6114	0.6190	0.5872	0.4019
AT3G51860	-1.1596	0.0676	-0.9481	0.1843	-1.4520	0.0421	-0.5915	0.5458	-0.8207	0.3643	-0.9047	0.2080	-2.0683	0.0003	-1.7850	0.0080	-0.5601	0.4286	-0.3615	0.7917	-1.2897	0.0959	-0.0437	0.9670	-1.5883	0.0193	-0.3309	0.6137
AT3G51890	-1.7025	0.0000	-0.8365	0.0107	-0.4316	0.3175	-1.0433	0.0052	-0.1676	0.8021	-0.4030	0.2856	-1.7184	0.0000	-1.1262	0.0006	-0.8191	0.0113	-0.7737	0.0441	-1.2214	0.0002	-2.0054	0.0000	-0.9596	0.0042	-0.7710	0.0111
AT3G51895	-1.1125	0.0884	-2.0881	0.0007	-1.7850	0.0097	-0.7971	0.3988	-0.4785	0.6830	-2.3335	0.0005	-1.5416	0.0107	-0.9120	0.3370	-2.2203	0.0003	-0.7768	0.4499	-0.1280	0.9276	-0.8098	0.2478	-0.9288	0.2849	-1.5431	0.0099
AT3G51910	-1.2920	0.0438	-2.0823	0.0008	-1.2468	0.1063	-1.6053	0.0429	-1.2255	0.1191	-0.6974	0.3573	-1.7132	0.0042	-0.8174	0.4222	-1.6467	0.0099	-0.3475	0.8075	-0.0537	0.9735	-1.1882	0.0711	-1.2489	0.1013	-0.6813	0.2751
AT3G52180	1.6774	0.0000	0.6408	0.1838	0.7331	0.1661	0.8651	0.1172	2.1341	0.0000	0.3816	0.4618	1.7346	0.0000	1.5292	0.0003	0.7375	0.0934	0.9914	0.0500	0.1536	0.8544	1.9419	0.0000	1.9733	0.0000	0.1697	0.7110
AT3G52310	0.2286	0.7650	1.8280	0.0012	1.4898	0.0204	1.0952	0.1598	0.4067	0.7112	1.0833	0.0962	-0.1075	0.8880	0.6275	0.5411	2.0987	0.0002	1.8551	0.0032	0.0970	0.9423	-0.3008	0.6968	1.8675	0.0015	1.5101	0.0055
AT3G52360	-1.9970	0.0008	-2.1348	0.0003	-2.1566	0.0006	-1.7776	0.0175	-1.5054	0.0307	-2.1517	0.0010	-2.2376	0.0001	-1.5073	0.0329	-2.4022	0.0001	-0.8122	0.4025	-0.3294	0.7674	-1.6203	0.0080	-1.6605	0.0123	-2.5419	0.0000
AT3G52400	-0.7987	0.1085	-2.0013	0.0000	-0.9084	0.1247	-0.8858	0.1742	-0.6278	0.3712	-0.5691	0.3157	-0.8436	0.0759	-1.6037	0.0012	-1.9843	0.0000	-1.4605	0.0061	-0.3084	0.7103	-1.3899	0.0029	-1.4858	0.0027	-0.6137	0.1879
AT3G52430	-2.0474	0.0005	-2.8526	0.0000	-1.5602	0.0228	-2.0335	0.0042	-0.7713	0.3978	-1.6881	0.0113	-1.9865	0.0005	-2.2557	0.0003	-2.8739	0.0000	-1.8280	0.0075	-0.9890	0.2311	-2.1954	0.0001	-1.9572	0.0018	-1.8990	0.0010
AT3G52450	-0.3995	0.6014	-2.3376	0.0001	-0.5165	0.5811	-0.9002	0.3213	-0.6814	0.4930	-1.5307	0.0269	-0.7401	0.2620	-0.9121	0.3314	-2.5962	0.0000	-1.2850	0.1129	1.1532	0.1575	-0.5662	0.4472	-2.0003	0.0021	-1.3743	0.0212
AT3G52526	-0.8913	0.0808	-2.2081	0.0000	-2.1047	0.0001	-0.6379	0.3794	-1.6277	0.0033	-0.1119	0.8777	-3.1135	0.0000	-0.2527	0.8399	0.0399	0.9549	-0.3773	0.6998	-0.3507	0.6757	-1.0345	0.0433	0.0229	0.9892	0.1621	0.7637
AT3G52535	-0.9694	0.0357	-2.4716	0.0000	-2.7730	0.0000	-0.5081	0.4575	-1.6663	0.0008	-0.2771	0.6389	-2.6125	0.0000	-0.8433	0.1585	-0.1634	0.7791	-0.1691	0.8849	0.4263	0.5290	-0.2210	0.7077	-0.6968	0.2751	-0.1979	0.6911
AT3G52680	0.1687	0.7876	-3.7709	0.0000	-3.8556	0.0000	0.2905	0.7180	-3.7341	0.0000	-0.0021	0.9982	-4.1319	0.0000	0.4162	0.6593	-0.4318	0.4387	0.1014	0.9450	0.5955	0.3795	0.1739	0.7899	0.0512	0.9708	-0.6028	0.2165
AT3G52720	-0.5944	0.3885	-1.6641	0.0071	-1.8158	0.0055	-1.1003	0.1877	-1.1304	0.1428	-1.9022	0.0035	-1.2455	0.0359	-1.1238	0.1588	-1.9577	0.0011	-1.1771	0.1421	0.3264	0.7668	-0.4684	0.5298	-1.6231	0.0135	-2.5053	0.0000
AT3G52740	-1.6313	0.0132	-1.3743	0.0547	-1.7134	0.0215	-0.9974	0.2938	-1.4156	0.0747	-2.1718	0.0023	-1.5339	0.0159	-1.8299	0.0123	-2.4434	0.0002	-0.8963	0.3844	-0.9930	0.2774	-0.1926	0.8420	-1.1814	0.1582	-2.0524	0.0011
AT3G52748	-1.9297	0.0034	-2.7606	0.0000	-1.8619	0.0130	-1.0746	0.2521	-1.5041	0.0576	-1.3868	0.0666	-1.9275	0.0021	-2.4074	0.0006	-2.4421	0.0003	-1.8929	0.0150	-2.2997	0.0019	-1.6359	0.0150	-2.1473	0.0026	-1.3902	0.0309
AT3G52900	-0.4620	0.5201	-0.8975	0.2031	-1.0805	0.1586	-0.8165	0.3584	0.1013	0.9502	-0.8046	0.2577	-0.7462	0.2393	0.0932	0.9662	-0.9930	0.1233	-0.4452	0.7185	-0.6281	0.4943	-0.2097	0.8058	-0.5292	0.6203	-2.0255	0.0004
AT3G53040	-0.7138	0.4082	2.7242	0.0002	0.2004	0.8835	-0.1777	0.9080	0.1620	0.9327	-0.6091	NA	-0.3550	0.7015	-0.0517	0.9877	0.4977	0.5827	0.1893	0.9311	1.8816	0.0340	0.0581	0.9644	-0.3380	0.8396	0.1868	0.8252
AT3G53150	-2.8932	0.0000	-2.8212	0.0000	-2.6434	0.0002	-1.9117	0.0251	-2.0760	0.0048	-2.2941	0.0023	-3.1551	0.0000	-2.7030	0.0001	-2.2894	0.0010	-2.0733	0.0081	-2.3600	0.0018	-3.4324	0.0000	-2.6632	0.0002	-1.6135	0.0148
AT3G53160	-1.9558	0.0003	-0.0593	0.9475	0.0044	0.9974	-0.2881	0.7672	-1.0210	0.1503	0.3627	0.6029	-2.4074	0.0000	-0.4214	0.7226	0.2990	0.6556	-0.5635	0.5659	-0.0255	0.9861	-1.8538	0.0006	-0.8781	0.2371	-1.1029	0.0396
AT3G53460	2.2395	0.0000	0.8618	0.0065	0.2977	0.5146	1.0043	0.0062	0.9790	0.0024	0.7927	0.0204	2.2504	0.0000	1.6394	0.0000	1.0449	0.0007	0.8082	0.0274	0.4942	0.2447	2.3605	0.0000	0.7284	0.0391	0.6126	0.0387
AT3G53650	0.4895	0.3800	-4.3225	0.0000	-4.2402	0.0000	-0.3390	0.6863	-4.4157	0.0000	-0.6800	0.2541	-4.8814	0.0000	-0.3597	0.7497	-0.8870	0.0991	-0.4486	0.6378	-0.7603	0.2776	0.4673	0.4216	-0.3735	0.6955	-1.9478	0.0001
AT3G53710	-0.7764	0.0442	-2.1257	0.0000	-1.9597	0.0000	-0.5650	0.3023	-1.8246	0.0000	-0.0893	0.8733	-2.4432	0.0000	-0.3008	0.7122	-0.2710	0.5464	-0.2015	0.8179	-0.5787	0.2680	-0.8223	0.0344	-0.3683	0.5665	-0.8418	0.0196
AT3G53830	-0.4160	0.4777	-0.9523	0.0846	-1.0240	0.0927	-0.3100	0.7180	-1.0274	0.0931	-1.4267	0.0097	-1.0936	0.0243	-0.8697	0.2031	-1.8783	0.0001	-1.1463	0.0655	-0.9153	0.1653	-0.4840	0.4115	-0.4638	0.5912	-2.0416	0.0000
AT3G53980	1.1647	0.1134	0.2531	0.8095	-0.1247	0.9267	-0.0892	0.9553	1.3826	0.1131	-0.3720	0.6925	1.0784	0.1319	0.6574	0.6333	0.0669	0.9477	1.0966	0.2850	1.8350	0.0248	2.7420	0.0000	0.2828	0.8590	1.1549	0.0879
AT3G54150	-2.0129	0.0014	-2.8985	0.0000	-1.4690	0.0531	-0.3096	0.7918	-1.5569	0.0361	-1.0999	0.1387	-2.0298	0.0008	-1.6559	0.0240	-1.9952	0.0020	-1.2681	0.1379	-1.4042	0.0808	-2.6890	0.0000	-2.0461	0.0024	-1.2501	0.0430
AT3G54420	-1.9934	0.0009	-1.7891	0.0045	-1.7694	0.0097	-0.5536	0.5739	-1.2212	0.1119	-2.1359	0.0016	-2.4269	0.0000	-1.6681	0.0157	-1.9326	0.0019	-1.2098	0.1383	-0.7245	0.4253	-1.8559	0.0022	-2.2158	0.0005	-1.2245	0.0391
AT3G54500	-2.4038	0.0000	-1.2869	0.0038	-1.0140	0.0496	-1.1002	0.0501	-2.4215	0.0000	-0.7721	0.1264	-2.4140	0.0000	-1.8600	0.0000	-1.1842	0.0080	-0.9875	0.0743	-0.0067	0.9993	-2.5390	0.0000	-1.9479	0.0000	-1.5904	0.0001
AT3G54510	-3.7083	0.0000	-0.3369	0.6663	0.2231	0.8184	-0.6013	0.4952	-1.6458	0.0069	-0.1262	0.8826	-3.4496	0.0000	-1.4771	0.0215	-0.5828	0.3656	-1.3575	0.0509	-1.0538	0.1596	-3.5719	0.0000	-0.8238	0.3006	-0.1421	0.8238
AT3G54580	0.1967	0.8486	1.9332	0.0112	1.5408	0.0836	0.2597	0.8558	1.0669	0.3072	0.1169	0.9170	0.3022	0.7442	2.5664	0.0008	0.2833	0.7645	0.6483	0.6453	2.3099	0.0047	-0.0251	0.9850	1.1857	0.2254	0.5264	0.4865
AT3G54590	0.4348	0.6394	2.3495	0.0014	1.7246	0.0460	0.0000	1.0000	1.1465	0.2621	1.0540	NA	0.8893	0.2593	2.0174	0.0143	0.1471	0.8837	0.5073	0.7444	2.3607	0.0038	0.1458	0.8973	0.5090	0.7218	0.2497	0.7568
AT3G54770	-2.4870	0.0001	0.5619	0.4878	0.8602	0.3159	-0.5344	0.6096	1.1058	0.1784	-0.0576	0.9554	-1.3714	0.0302	0.3479	0.8302	-1.1510	0.1035	-1.8184	0.0212	-0.9181	0.3264	-1.9005	0.0041	0.1402	0.9307	-0.0409	0.9574
AT3G54830	-2.2237	0.0000	1.2211	0.0017	1.3425	0.0009	1.0903	0.0241	-0.1581	0.8593	0.4768	0.3095	-1.9813	0.0000	0.8199	0.0916	1.3391	0.0005	0.8814	0.0720	0.6842	0.1924	-2.3856	0.0000	-0.5205	0.3758	0.2384	0.5673
AT3G55090	-0.2809	0.5652	-2.1061	0.0000	-2.0382	0.0000	-0.2121	0.7674	-2.6455	0.0000	-0.5068	0.3099	-1.6761	0.0000	-0.6608	0.2589	-0.6102	0.1759	-0.5937	0.3467	-0.0193	0.9860	-0.3889	0.4152	-0.3605	0.6260	-1.5213	0.0004
AT3G55150	-3.0927	0.0000	-2.3343	0.0000	-1.0008	0.1601	-2.5395	0.0000	-1.8054	0.0023	-2.5333	0.0000	-2.7473	0.0000	-2.8066	0.0000	-2.7338	0.0000	-2.2791	0.0002	-1.7113	0.0073	-3.2908	0.0000	-2.6985	0.0000	-2.6691	0.0000
AT3G55230	-0.6880	0.3673	-0.9124	0.2601	0.7466	0.4315	-0.1018	0.9458	-0.4387	0.7406	-2.0867	0.0064	-0.8752	0.2124	-0.5368	0.7112	-1.5975	0.0252	-0.9332	0.3775	1.4598	0.0752	-0.9148	0.2210	-1.3850	0.0963	1.5285	0.0155
AT3G55240	-2.1659	0.0000	-1.2897	0.0299	-0.6379	0.4219	-1.2573	0.0860	0.1954	0.8804	-0.7403	0.2620	-2.7868	0.0000	0.0817	0.9688	-1.3820	0.0157	-1.2949	0.0615	-1.2164	0.0808	-2.6070	0.0000	0.0711	0.9625	-1.4982	0.0049
AT3G55500	0.2473	0.7738	1.9815	0.0020	0.9835	0.2471	1.5349	0.0633	-0.6012	0.5878	1.4937	0.0360	0.2221	0.7889	0.9967	0.2877	2.7113	0.0000	1.8627	0.0111	-0.1691	0.9050	-0.1840	0.8467	2.1525	0.0011	2.3707	0.0001
AT3G55630	-0.3230	0.6108	-1.2773	0.0176	-1.6015	0.0037	-0.5498	0.4908	-1.2911	0.0265	-1.6883	0.0023	-0.5975	0.2747	-0.9360	0.1730	-1.7909	0.0004	-0.6688	0.4159	0.3520	0.6916	0.0821	0.9188	-0.9158	0.1626	-2.6322	0.0000
AT3G55646	-0.5968	0.2135	-1.1269	0.0164	-1.3929	0.0043	-0.8843	0.1391	-2.6582	0.0000	-0.7793	0.1307	-1.9387	0.0000	-1.1503	0.0239	-1.4215	0.0018	-1.6959	0.0005	-0.7832	0.1909	-0.6175	0.2053	-1.8608	0.0001	-2.4153	0.0000
AT3G55734	-1.5021	0.0508	-1.8419	0.0179	-0.7187	0.5157	-1.7158	0.0797	-0.8312	0.4865	-0.5825	NA	-2.3209	0.0010	-1.4802	0.1236	-1.0467	NA	-1.6171	0.0889	-0.8397	0.4562	-1.2339	0.1262	-0.8311	0.4893	-0.6793	NA
AT3G55760	0.4750	0.3770	0.3406	0.5903	0.9559	0.0979	0.0000	1.0000	1.7486	0.0002	-0.0122	0.9887	0.3543	0.5145	1.4794	0.0033	0.3978	0.4701	0.5585	0.4834	-0.1357	0.8952	0.7128	0.1655	2.7176	0.0000	1.1638	0.0097
AT3G55950	-1.3440	0.0222	-2.1912	0.0001	-1.2543	0.0733	-1.1556	0.1400	-1.2592	0.0729	-1.7953	0.0048	-1.5452	0.0056	-1.7895	0.0044	-2.3912	0.0000	-1.4055	0.0483	-0.1460	0.9105	-1.7505	0.0022	-1.5777	0.0131	-1.4513	0.0093
AT3G55970	-2.3003	0.0002	-2.5317	0.0000	-2.1810	0.0010	0.0050	0.9984	-2.3607	0.0002	-1.0955	0.1343	-2.9963	0.0000	-2.4134	0.0002	-0.3164	0.6910	0.0565	0.9782	-1.9763	0.0053	-1.3895	0.0337	-1.7123	0.0136	0.0476	0.9494
AT3G56290	-1.5934	0.0070	-1.7722	0.0031	-1.8478	0.0038	-1.2101	0.1296	-1.9138	0.0019	-2.0405	0.0014	-1.1714	0.0468	-1.9260	0.0023	-2.4013	0.0000	-0.6662	0.5175	-0.0619	0.9661	-0.9887	0.1217	-1.6111	0.0128	-3.5863	0.0000
AT3G56300	-0.1183	0.8581	-0.5950	0.3006	-0.6537	0.3135	-0.8393	0.2043	-0.4450	0.5855	-1.1807	0.0295	-0.4813	0.3426	-0.2199	0.8608	-1.4477	0.0033	-0.6552	0.3752	0.1525	0.8763	-0.1078	0.8784	-0.3834	0.6617	-2.0874	0.0000
AT3G56330	1.7362	0.0028	-0.0077	0.9951	-0.5236	0.5645	0.4393	0.6586	0.5502	0.5949	-0.3911	0.6159	1.7992	0.0013	0.7468	0.4488	-0.4771	0.5011	0.0347	0.9841	0.5812	0.5323	2.1079	0.0002	0.8448	0.3199	-0.9309	0.1190
AT3G56400	-1.7502	0.0164	-2.8413	0.0000	-1.5656	0.0721	-1.1737	0.2579	-0.6198	0.6361	-2.0448	0.0102	-1.5372	0.0310	-1.5830	0.0739	-2.4415	0.0007	-1.4515	0.1292	-0.2095	0.8944	-1.6279	0.0289	-1.3530	0.1357	-0.9136	0.2021
AT3G56790	-2.3381	0.0006	-1.8024	0.0137	-0.2901	0.8018	-0.3851	0.7469	-0.8645	0.4061	-0.7073	0.4043	-2.3360	0.0003	-1.0118	0.3296	-1.4242	0.0519	-1.6242	0.0614	1.5490	0.0551	-1.8710	0.0073	-1.4293	0.0908	-2.0120	0.0031
AT3G56880	0.3093	0.5371	0.3704	0.4883	0.3727	0.5503	0.6182	0.3068	-0.2006	0.8257	0.2644	0.6231	-0.2354	0.6381	-0.1059	0.9358	0.1536	0.7774	-0.0338	0.9796	2.0056	0.0000	0.0718	0.9124	0.5737	0.3392	1.3080	0.0009
AT3G56970	1.4737	0.0249	-0.0503	0.9636	-0.0104	0.9944	0.0837	0.9541	0.0002	1.0000	-0.0413	0.9691	1.5745	0.0124	-0.0045	0.9992	0.0317	0.9749	-0.0157	0.9943	0.0522	0.9752	3.2949	0.0000	-0.0428	0.9843	-0.0348	0.9636
AT3G56980	3.6096	0.0000	0.0550	0.9647	0.0000	1.0000	0.5432	0.6617	0.0000	1.0000	0.0000	1.0000	3.8561	0.0000	0.1789	0.9404	0.1314	0.8970	0.2276	0.9138	0.1157	0.9463	6.2808	0.0000	0.0000	1.0000	0.2383	0.7710
AT3G57020	-2.1143	0.0000	-1.2136	0.0280	-1.5582	0.0058	-1.3362	0.0421	-3.0368	0.0000	-1.8138	0.0012	-2.7181	0.0000	-2.5418	0.0000	-1.5043	0.0041	-0.7134	0.3801	-0.7185	0.3321	-1.6286	0.0013	-1.3787	0.0153	-1.9193	0.0001
AT3G57180	0.0832	0.9104	-0.5719	0.3706	-0.8093	0.2326	-0.6227	0.4290	-0.3777	0.6997	-0.8892	0.1348	-0.0824	0.9059	-0.0579	0.9751	-1.1182	0.0376	-0.8066	0.2865	-0.2574	0.7896	0.2763	0.6864	-0.4388	0.6379	-2.0503	0.0000
AT3G57240	-1.3214	0.0917	-2.0278	0.0086	-0.5022	0.6689	-1.3614	0.1900	1.4394	0.1290	-1.6116	0.0555	-1.2458	0.1017	-0.6875	0.6473	-2.1118	0.0052	-1.5953	0.0978	-1.3928	0.1598	-1.4482	0.0656	-0.8598	0.4688	-0.9367	0.2054
AT3G57260	-1.5120	0.0491	-2.3233	0.0019	-1.5715	0.0830	-1.3705	0.1862	-0.0595	0.9811	-1.4945	0.0775	-1.5969	0.0297	-1.7524	0.0478	-2.1637	0.0041	-0.9332	0.4488	-0.9438	0.3878	-1.6104	0.0373	-1.4618	0.1114	-0.6073	0.4289
AT3G57460	-2.4642	0.0001	-3.5049	0.0000	-1.9623	0.0070	-1.3220	0.1403	-0.9422	0.3266	-2.2202	0.0020	-2.7233	0.0000	-2.1625	0.0022	-2.9814	0.0000	-1.9401	0.0112	-1.2741	0.1439	-2.8240	0.0000	-2.4342	0.0003	-2.4768	0.0001
AT3G58070	-0.4044	0.5922	-0.8866	0.2294	-1.5129	0.0395	-0.6732	0.4830	-0.7880	0.4014	-1.0204	0.1615	0.2019	0.7984	-0.3259	0.8382	-1.7137	0.0082	-0.4287	0.7405	0.0934	0.9474	-0.2128	0.8078	-0.4142	0.7355	-2.1072	0.0007
AT3G58550	1.9099	0.0023	0.5645	0.5024	-0.1986	0.8665	-0.2262	0.8613	0.9364	0.3115	0.6192	0.4475	0.7619	0.2654	1.1730	0.1742	0.8308	0.2469	2.4983	0.0002	2.1136	0.0024	1.4311	0.0296	1.4997	0.0421	2.4134	0.0001
AT3G58570	1.3601	0.0000	1.2120	0.0002	1.4770	0.0000	1.1133	0.0039	2.0924	0.0000	0.9687	0.0080	1.8744	0.0000	1.7819	0.0000	1.3140	0.0000	1.4695	0.0000	0.4039	0.4151	1.6772	0.0000	1.6467	0.0000	1.2561	0.0001
AT3G59140	-0.1326	0.8855	-1.0532	0.1537	-0.9102	0.2955	-0.1110	0.9370	-0.0594	0.9773	-2.0043	0.0041	-0.2521	0.7572	-0.0181	0.9947	-1.6733	0.0108	0.0360	0.9841	0.4465	0.6886	0.2186	0.8119	-1.0401	0.2252	-2.0681	0.0007
AT3G59220	-2.8658	0.0000	-1.7847	0.0103	-1.2374	0.1444	-1.5906	0.0671	-1.0379	0.2619	-3.1928	0.0000	-2.6494	0.0000	-1.6873	0.0286	-2.3955	0.0003	-1.0138	0.3106	-1.5303	0.0637	-1.8516	0.0055	-1.2788	0.1245	-1.9784	0.0022
AT3G59250	-0.1152	0.9104	-1.3743	0.0854	-0.4337	0.7033	-0.6586	0.5668	-0.6974	0.5665	-1.9496	0.0150	0.8675	0.2478	-0.5302	0.7392	-2.1254	0.0037	-0.5167	0.7269	0.4588	0.7130	0.0796	0.9461	-0.5020	0.7166	-1.7675	0.0110
AT3G59880	-1.0014	0.0980	-2.0243	0.0004	-1.0947	0.1335	-1.3057	0.0861	-0.8097	0.3340	-1.1003	0.0967	-1.4755	0.0079	-1.4268	0.0342	-1.8587	0.0015	-0.8378	0.3449	-0.4777	0.6133	-1.3428	0.0233	-1.5115	0.0189	-0.4588	0.4407
AT3G59900	-2.1920	0.0001	-1.4869	0.0134	-0.4346	0.6247	-1.3360	0.0765	0.2276	0.8617	-1.5847	0.0128	-2.3360	0.0000	-0.4120	0.7553	-2.3279	0.0000	-2.7422	0.0000	-1.6796	0.0108	-2.0533	0.0002	-0.3720	0.7467	-0.5903	0.3078
AT3G59930	-0.2147	0.8363	1.8501	0.0176	1.0705	0.2866	1.6356	0.0958	-0.1922	0.9162	1.6378	0.0496	0.1257	0.9004	0.6446	0.6737	2.2049	0.0032	2.3355	0.0051	-0.1809	0.9132	0.2438	0.8205	-0.1055	0.9567	1.0248	0.1593
AT3G60120	-2.8762	0.0000	-1.6896	0.0305	-1.1972	0.2091	-0.7623	0.5000	-1.5046	0.0942	-1.0937	NA	-1.9083	0.0062	-0.8152	0.5250	-1.1491	0.1434	-1.7490	0.0521	-2.3371	0.0042	-2.7120	0.0001	-1.6874	0.0457	-0.5983	0.4224
AT3G60415	-3.4206	0.0000	-2.6962	0.0000	-2.0453	0.0003	-1.7373	0.0096	-1.3219	0.0408	-2.1305	0.0003	-3.0738	0.0000	-3.0238	0.0000	-2.6964	0.0000	-2.4700	0.0000	-1.8181	0.0029	-3.7689	0.0000	-2.6705	0.0000	-2.7486	0.0000
AT3G60520	-2.4911	0.0000	0.3303	0.4081	0.1554	0.7684	-0.2509	0.6344	0.7614	0.0370	-0.1879	0.6575	-2.3168	0.0000	-0.0660	0.9510	0.0613	0.8895	-0.2580	0.6861	-1.1970	0.0007	-1.7040	0.0000	0.8021	0.0228	-0.2001	0.5595
AT3G60550	-1.2790	0.0714	-1.6000	0.0307	-0.8617	0.3643	-0.1756	0.8969	-0.3721	0.7944	-2.0067	0.0099	-1.2626	0.0615	-1.1839	0.2099	-1.7603	0.0142	-2.4744	0.0013	0.1716	0.9089	-1.4511	0.0414	-0.6925	0.5478	-0.2962	0.6914
AT3G60950	-0.0134	0.9902	0.7558	0.3168	1.5352	0.0308	-0.0102	0.9955	2.8464	0.0000	-0.0100	0.9932	0.7857	0.2302	-0.0125	0.9957	-0.0110	0.9919	-0.0121	0.9951	-0.0111	0.9982	-0.0136	0.9911	0.0228	0.9929	0.0045	0.9958
AT3G60980	0.2172	0.5804	2.2223	0.0000	2.2020	0.0000	0.6219	0.1540	1.5969	0.0000	0.8493	0.0164	1.5230	0.0000	0.4720	0.3320	0.7972	0.0155	0.7229	0.0727	-0.2845	0.5860	0.1239	0.7827	0.9362	0.0062	0.7619	0.0133
AT3G61028	-0.8404	0.2866	2.1621	0.0019	2.3171	0.0016	1.1939	0.2265	0.8365	0.4404	2.2270	0.0031	0.1698	0.8557	0.8132	0.5073	1.4326	0.0506	1.2903	0.1790	-0.6063	0.6007	-0.6150	0.4745	0.6318	0.6120	1.6420	0.0141
AT3G61160	0.4420	0.4887	0.6577	0.3178	1.4700	0.0139	0.6759	0.4074	0.7385	0.3498	1.3933	0.0189	0.1752	0.8030	0.6288	0.5026	1.3854	0.0115	1.2283	0.0674	0.6621	0.4047	0.5020	0.4368	1.7181	0.0021	3.2567	0.0000
AT3G61220	-1.2950	0.0621	-1.4287	0.0495	-1.8208	0.0153	-0.9922	0.3098	-1.3398	0.1094	-2.7364	0.0001	-1.1998	0.0752	-1.9581	0.0078	-2.7700	0.0000	-0.3045	0.8507	-0.1771	0.9050	-0.5201	0.5320	-1.2955	0.1184	-2.9102	0.0000
AT3G61280	-1.2462	0.0957	-2.6191	0.0002	-1.2275	0.1840	-1.7477	0.0570	-0.0833	0.9691	-1.4476	0.0727	-1.4477	0.0404	-1.5501	0.0763	-2.5581	0.0003	-0.8694	0.4650	-1.2815	0.1787	-1.4114	0.0591	-1.3044	0.1481	-1.2618	0.0669
AT3G61370	-0.5815	0.2376	-0.8008	0.1194	-0.8332	0.1496	-0.7587	0.2370	-0.0851	0.9427	-0.6679	0.2121	-0.6013	0.2006	-0.4805	0.5602	-0.8419	0.0816	-0.6197	0.3837	-0.8038	0.1878	-0.3192	0.5738	-0.5819	0.3941	-2.0435	0.0000
AT3G61390	-3.2934	0.0000	-1.9040	0.0114	-2.0559	0.0106	-2.0369	0.0235	-2.0638	0.0085	-0.8662	0.3157	-3.8529	0.0000	-2.5275	0.0009	-1.9190	0.0094	-1.2256	0.2296	-2.8085	0.0002	-2.9513	0.0000	-2.8520	0.0001	-1.6770	0.0158
AT3G61890	-1.6957	0.0036	-0.0570	0.9538	-0.3964	0.6680	-0.0836	0.9495	-1.3410	0.0548	1.0466	0.1221	-2.3187	0.0000	-0.3789	0.7888	0.5524	0.4163	0.4599	0.6982	-1.0512	0.1830	-0.9771	0.1240	0.0437	0.9816	0.3194	0.6131
AT3G62040	1.0616	0.1513	-0.2785	0.7892	-0.3982	0.7262	-0.1182	0.9387	0.8886	0.3951	-1.0889	0.1906	0.3386	0.6920	0.8452	0.4737	-1.1181	0.1443	0.8708	0.4445	2.2084	0.0038	0.8572	0.2751	0.0473	0.9839	-1.1114	0.1097
AT3G62100	-2.2941	0.0000	0.0032	0.9985	-0.3877	0.6707	-0.6583	0.4642	-0.0654	0.9699	0.1439	0.8668	-1.9391	0.0004	0.1465	0.9358	-0.6000	0.3655	-1.3807	0.0560	-1.7227	0.0103	-2.1332	0.0002	0.7168	0.4148	-1.9796	0.0005
AT3G62260	-1.7593	0.0000	-0.5468	0.2006	-0.6821	0.1383	-0.4327	0.4366	-1.2221	0.0012	-0.7028	0.0920	-2.1460	0.0000	-1.3099	0.0006	-0.7316	0.0568	-0.2813	0.7001	0.5864	0.2335	-1.9731	0.0000	-0.8834	0.0338	-0.9304	0.0084
AT3G62410	-0.3074	0.6166	-1.3312	0.0089	-1.0657	0.0718	-0.9589	0.1531	-0.4296	0.6261	-1.5868	0.0029	0.1538	0.8119	-1.0034	0.1082	-1.7944	0.0002	-0.8728	0.2047	0.0786	0.9456	-0.0441	0.9581	-0.6945	0.3255	-2.1135	0.0000
AT3G62540	0.0898	0.9333	0.0464	0.9702	0.0678	0.9650	0.3701	0.7794	2.9423	0.0000	0.3061	0.7625	0.0511	0.9604	0.3596	0.8542	0.8634	0.2894	0.5730	0.6969	0.0959	0.9563	-0.4733	0.6185	0.0324	0.9905	0.2296	0.7794
AT3G62550	0.9628	0.1603	2.1143	0.0008	2.0598	0.0024	1.9477	0.0121	2.5044	0.0001	2.7913	0.0000	1.4657	0.0188	1.8641	0.0079	2.7898	0.0000	2.4233	0.0004	-1.3910	0.0825	1.4790	0.0240	2.8418	0.0000	2.3458	0.0001
AT3G63110	-0.6294	0.3037	-0.5498	0.4271	-1.0073	0.1495	-0.3329	0.7269	-0.1899	0.8820	-1.0856	0.0825	-0.1548	0.8304	0.0676	0.9731	-0.9424	0.1089	-0.1461	0.9266	0.4602	0.6064	-0.4249	0.5322	-1.9616	0.0005	-2.3191	0.0000
AT3G63160	1.9833	0.0000	1.0768	0.0353	1.4050	0.0070	0.8697	0.1893	2.0369	0.0000	1.3017	0.0133	2.1213	0.0000	1.7860	0.0003	1.2666	0.0092	1.0519	0.0819	-0.2615	0.7649	2.3058	0.0000	2.4736	0.0000	1.1317	0.0132
AT3G63380	-2.1313	0.0004	-2.4234	0.0001	-1.2006	0.1256	-0.0372	0.9807	-0.7765	0.4150	-1.4678	0.0355	-1.6016	0.0078	-1.9515	0.0038	-2.4723	0.0001	-1.4035	0.0753	-1.6750	0.0219	-2.1386	0.0004	-2.1843	0.0006	-1.5081	0.0118
AT4G00240	-1.0595	0.0302	-1.5073	0.0020	-0.7294	0.2556	-1.1574	0.0633	-0.3145	0.7385	-1.3422	0.0126	-0.9882	0.0359	-0.9878	0.1040	-2.0700	0.0000	-1.6539	0.0021	-1.1048	0.0637	-0.9051	0.0734	-1.2115	0.0263	-2.0113	0.0000
AT4G00490	-0.5809	0.2758	-1.2638	0.0118	-0.5795	0.3943	-0.8686	0.1957	-0.1546	0.8929	-1.4902	0.0046	-0.3670	0.5086	-0.5493	0.5206	-1.5857	0.0010	-0.9824	0.1208	-0.3213	0.7039	-0.6723	0.2079	-0.8765	0.1527	-2.3414	0.0000
AT4G00700	-1.8797	0.0061	-2.5290	0.0002	-1.9988	0.0085	-1.9353	0.0240	-0.5428	0.6718	-1.6966	0.0270	-1.8054	0.0064	-2.2864	0.0017	-2.4834	0.0003	-1.6147	0.0591	-1.1852	0.2027	-1.6734	0.0170	-2.0401	0.0055	-0.8871	0.1922
AT4G00955	-1.3316	0.0441	-2.3133	0.0003	-1.1872	0.1474	-1.4615	0.0845	-0.8293	0.3923	-1.6570	0.0209	-1.5439	0.0136	-1.6699	0.0233	-2.7300	0.0000	-0.4319	0.7556	-0.3766	0.7451	-1.5173	0.0214	-2.1698	0.0012	-1.5988	0.0099
AT4G00960	-1.4335	0.0327	-1.7540	0.0111	-1.4293	0.0730	-1.8188	0.0278	-1.5633	0.0433	-1.5751	0.0340	-1.8429	0.0033	-1.6225	0.0351	-2.0963	0.0018	-0.8414	0.4296	-0.3780	0.7493	-1.6370	0.0145	-1.5911	0.0360	-1.7679	0.0059
AT4G00970	-0.6065	0.1713	-2.2793	0.0000	-0.8192	0.1093	-1.1373	0.0273	-1.0606	0.0226	-0.6796	0.1564	-1.3669	0.0004	-1.2786	0.0042	-1.9065	0.0000	-1.1491	0.0169	-0.1557	0.8512	-1.4582	0.0002	-0.9384	0.0501	-0.9566	0.0159
AT4G01010	-1.7733	0.0000	-1.9551	0.0000	-0.7070	0.2037	-1.1202	0.0366	-0.4504	0.5162	-0.8303	0.0875	-2.1476	0.0000	-1.4734	0.0010	-1.5259	0.0003	-0.9766	0.0674	-0.6616	0.2623	-2.4065	0.0000	-1.2344	0.0072	-0.9931	0.0154
AT4G01023	-0.2019	0.8481	1.5504	0.0574	1.2331	0.2069	1.5261	0.1284	0.1418	0.9427	1.9441	0.0184	-0.4256	0.6371	0.9752	0.4248	2.3650	0.0015	1.5277	0.1188	-0.0060	1.0000	-0.3084	0.7677	0.3970	0.8048	0.6866	0.3653
AT4G01026	0.1659	0.8104	1.0485	0.0574	0.7376	0.2799	1.3730	0.0291	-0.0436	0.9795	1.7330	0.0014	0.0421	0.9535	1.0104	0.1186	2.0517	0.0000	1.3520	0.0226	-0.6104	0.4230	-0.0587	0.9443	0.4432	0.6269	0.8593	0.0799
AT4G01130	2.1422	0.0000	2.1121	0.0000	2.0217	0.0000	1.7387	0.0004	2.3220	0.0000	1.7502	0.0002	2.0885	0.0000	2.1967	0.0000	2.3571	0.0000	1.9244	0.0000	0.2470	0.7650	2.2665	0.0000	2.6769	0.0000	2.1283	0.0000
AT4G01360	-2.2396	0.0000	-0.2796	0.7255	-0.9158	0.2114	0.0311	0.9819	-1.6098	0.0088	-0.7711	0.2451	-2.3194	0.0000	-1.7744	0.0036	-0.7936	0.1931	-0.6094	0.5327	0.5783	0.4929	-2.4339	0.0000	-2.0333	0.0006	-1.8213	0.0010
AT4G01590	-0.5190	0.3340	-2.0083	0.0000	-1.9840	0.0000	-0.5695	0.4377	-1.4293	0.0050	-0.3653	0.5494	-2.1336	0.0000	0.0106	0.9957	-0.6479	0.2167	-0.3130	0.7630	-0.1947	0.8375	-0.3852	0.5122	-0.5171	0.5083	-1.1040	0.0161
AT4G01593	-0.1718	0.7699	-3.4807	0.0000	-3.4050	0.0000	0.3390	0.6311	-3.4353	0.0000	0.2048	0.7365	-4.0483	0.0000	-0.0900	0.9517	-0.1444	0.8093	0.2192	0.8308	0.6058	0.3254	0.4582	0.3555	0.2631	0.7739	0.2323	0.6327

AT4G01630	2.1763	0.0027	0.9746	0.2749	0.1337	0.9253	0.7407	0.5286	1.2446	0.2092	1.5716	NA	1.0599	0.1676	0.5992	0.7077	0.4988	0.5760	1.3025	0.2089	1.3754	0.1607	1.1831	0.1397	0.8232	0.4881	0.2542	0.7536
AT4G01680	-2.1350	0.0004	-1.5303	0.0192	-0.8607	0.3082	-1.6042	0.0411	-0.9873	0.2453	-2.3764	0.0004	-2.5349	0.0000	-1.1485	0.1644	-2.5388	0.0000	-1.1383	0.1831	-0.4306	0.6890	-2.3882	0.0001	-1.0824	0.1765	-0.4804	0.4525
AT4G01700	-0.9214	0.1999	-2.0294	0.0024	-0.9474	0.2915	-0.9909	0.3050	-0.1625	0.9229	-1.8212	0.0129	-0.8554	0.2214	-1.1239	0.2266	-2.3086	0.0005	-0.5272	0.6923	-0.2939	0.8206	-1.2243	0.0811	-1.3859	0.0810	-1.6278	0.0105
AT4G01720	-0.8044	0.0561	-2.0337	0.0000	-0.6746	0.2064	-1.8088	0.0000	-0.3681	0.6087	-1.1157	0.0135	-1.2055	0.0019	-1.2848	0.0034	-2.0366	0.0000	-1.5082	0.0006	-0.8423	0.1067	-1.3643	0.0006	-1.2917	0.0026	-1.1966	0.0022
AT4G01740	0.2329	0.6510	-4.7058	0.0000	-4.6307	0.0000	-1.3187	0.0109	-4.6608	0.0000	-1.3218	0.0065	-5.2839	0.0000	-0.8682	0.1030	-1.5902	0.0003	-0.8139	0.1531	0.3045	0.6627	-0.1612	0.7788	-0.7645	0.1637	-1.1017	0.0093
AT4G01750	0.0523	0.9567	-2.8630	0.0000	-1.1439	0.1582	-1.3084	0.1253	-0.3890	0.7611	-1.1078	0.1301	-0.2126	0.7970	-0.7681	0.4788	-2.1706	0.0006	-0.9125	0.3509	-0.0978	0.9468	-0.4283	0.5980	-1.9221	0.0044	-1.5955	0.0088
AT4G01870	-1.3725	0.0415	0.6353	0.4465	0.6831	0.4770	0.7321	0.4752	-1.2135	0.1530	1.1271	0.1374	-1.4714	0.0221	0.4704	0.7551	1.4195	0.0385	1.0571	0.2696	-2.0586	0.0049	-1.4965	0.0267	-1.2697	0.1184	0.5942	0.3732
AT4G01883	-0.0547	0.9252	-0.6457	0.1571	-0.4535	0.4217	-0.5554	0.3413	-0.4276	0.5038	-1.1442	0.0090	-0.0138	0.9804	-0.5710	0.3472	-1.3660	0.0005	-1.2797	0.0038	-0.6834	0.2010	-0.0994	0.8670	-0.8535	0.0686	-2.7521	0.0000
AT4G01895	-2.5375	0.0001	-1.3148	0.0848	-0.7291	0.4628	-0.6274	0.5681	-0.5853	0.6348	-0.3223	0.7287	-1.8056	0.0060	-0.7230	0.5642	-1.0270	0.1671	-1.0911	0.2757	-1.3839	0.1186	-1.6641	0.0173	-1.3651	0.1057	-1.4006	0.0349
AT4G01950	-0.4315	0.4413	0.0023	0.9986	-0.1823	0.8258	-0.0850	0.9349	-0.7861	0.2275	-0.6777	0.2325	-0.7318	0.1368	-0.6545	0.3905	-0.4995	0.3613	0.0380	0.9800	2.5611	0.0000	-0.2830	0.6504	-0.0311	0.9849	-0.1995	0.7060
AT4G02280	0.1337	0.8787	1.0946	0.1106	0.3120	0.7565	0.2480	0.8288	0.3893	0.7484	0.0649	0.9468	-0.2895	0.7013	-0.0555	0.9803	1.0522	0.1059	0.9819	0.2683	0.8833	0.3039	2.1200	0.0003	1.1344	0.1373	1.1873	0.0423
AT4G02320	-1.7265	0.0225	-1.8327	0.0198	-0.5588	0.6282	-1.1366	0.2954	-1.1104	0.2941	-1.0460	NA	-1.6166	0.0276	-0.4079	0.8324	-1.5545	0.0469	-1.5425	0.1147	-1.4872	0.1239	-2.0541	0.0059	-1.3337	0.1633	-0.8862	0.2324
AT4G02330	-0.7194	0.2735	-2.2275	0.0001	-0.9602	0.2185	-1.2901	0.1021	-0.4907	0.6523	-1.2623	0.0598	-1.6418	0.0039	-0.8966	0.3145	-2.4107	0.0000	-1.9018	0.0043	-0.5918	0.5222	-1.7589	0.0028	-0.6747	0.4778	-1.2157	0.0332
AT4G02360	0.3350	0.6861	0.8838	0.2505	0.3284	0.7599	0.6377	0.5349	0.8763	0.3555	-0.0300	0.9790	0.1849	0.8276	0.2054	0.9156	0.8021	0.2632	0.7134	0.5274	0.9058	0.3242	2.0919	0.0007	0.9143	0.3231	1.4904	0.0154
AT4G02380	-2.3269	0.0000	-0.0470	0.9489	-0.2595	0.7130	-0.1746	0.8328	-0.7752	0.1822	0.5398	0.3056	-2.5111	0.0000	-0.9886	0.0630	0.6279	0.1899	0.0485	0.9729	-1.0112	0.0611	-2.0950	0.0000	-1.6720	0.0002	0.2956	0.5218
AT4G02410	-1.8522	0.0001	-2.7704	0.0000	-2.5680	0.0000	-0.7423	0.3093	-2.7469	0.0000	-1.5082	0.0062	-3.3511	0.0000	-1.3735	0.0142	-1.2506	0.0154	-0.4177	0.6696	0.2441	0.7980	-1.3472	0.0073	-0.8876	0.1684	-0.8673	0.0739
AT4G02520	-1.6794	0.0204	-1.2282	0.1329	-0.8455	0.4056	-1.0430	0.3206	-0.4526	0.7517	-0.6162	0.4953	-2.0644	0.0025	-1.6276	0.0592	-0.6866	0.4048	-0.6237	0.6519	-1.3313	0.1609	-1.7669	0.0152	-1.0764	0.2780	0.1892	0.8146
AT4G02530	-0.0656	0.9180	-0.6831	0.1792	-0.6666	0.2513	-0.5790	0.3763	-0.2330	0.8019	-0.5594	0.2831	0.0106	0.9865	-0.1434	0.9117	-0.8492	0.0651	-0.8325	0.1575	-0.6623	0.2764	-0.1105	0.8665	-0.7268	0.2071	-2.3684	0.0000
AT4G02540	0.6169	0.0168	-6.3572	0.0000	-6.0407	0.0000	-0.4905	0.1684	-6.2216	0.0000	-0.3552	0.2479	-6.6828	0.0000	-0.2057	0.7141	-0.1731	0.5805	-0.3063	0.4842	0.3055	0.4246	0.1007	0.7789	-0.4947	0.1267	-0.2688	0.3036
AT4G02800	0.8038	0.0954	0.1239	0.8620	-0.3368	0.6404	0.3539	0.6444	0.7261	0.2529	-0.0557	0.9385	0.6042	0.2112	0.7623	0.2413	-0.0430	0.9487	0.0228	0.9860	-0.0807	0.9401	0.8527	0.0792	-0.2852	0.7646	-2.0713	0.0000
AT4G02970	-0.1720	0.8261	-1.4091	0.0182	-2.4637	0.0000	0.6547	0.4554	-2.6849	0.0000	1.1127	0.0839	-2.1010	0.0001	0.4695	0.6969	1.2512	0.0339	0.9505	0.2406	-0.1533	0.9026	-0.1325	0.8773	0.2561	0.8397	1.1268	0.0381
AT4G03210	2.4581	0.0000	0.1950	0.7684	0.4621	0.4852	0.8399	0.1782	1.7275	0.0001	0.3372	0.5595	1.9319	0.0000	1.2747	0.0107	0.1904	0.7434	0.9410	0.1088	0.7160	0.2492	2.1014	0.0000	0.5766	0.3951	0.0917	0.8600
AT4G03450	-2.5669	0.0004	-3.3347	0.0000	-1.6328	0.0672	-1.3536	0.1912	-1.0060	0.3608	-2.1926	0.0073	-2.6119	0.0002	-2.5284	0.0012	-3.3370	0.0000	-1.7766	0.0528	-1.1242	0.2804	-2.9171	0.0000	-2.0916	0.0092	-2.7224	0.0001
AT4G03460	-1.9482	0.0001	-1.9010	0.0003	-0.9743	0.1404	-1.6236	0.0111	-0.9236	0.1787	-1.2558	0.0346	-2.1530	0.0000	-1.0149	0.1311	-1.9591	0.0003	-1.7752	0.0028	-0.7321	0.3228	-1.7748	0.0005	-1.4733	0.0115	-1.6738	0.0014
AT4G04330	2.0541	0.0008	1.9518	0.0020	1.9683	0.0037	1.6370	0.0402	2.5167	0.0000	1.6449	0.0182	2.1783	0.0002	1.7209	0.0146	1.9285	0.0023	2.4385	0.0003	-0.9159	0.3088	2.6537	0.0000	3.0471	0.0000	2.4828	0.0000
AT4G04490	-1.6110	0.0226	-2.6173	0.0001	-1.5021	0.0741	-0.1559	0.9135	-0.2041	0.9028	-1.8328	0.0175	-1.7228	0.0108	-1.9537	0.0117	-2.5857	0.0002	-1.2154	0.2162	-1.3491	0.1392	-1.9785	0.0043	-1.6857	0.0332	-1.6061	0.0162
AT4G04500	-1.6977	0.0230	-2.6741	0.0002	-1.4114	0.1272	-1.1166	0.2975	0.2568	0.8811	-1.7516	0.0332	-1.9239	0.0067	-1.9383	0.0204	-2.9476	0.0000	-0.9153	0.4519	-1.0853	0.2959	-2.0195	0.0061	-1.8775	0.0222	-1.2172	0.0889
AT4G04510	-1.8571	0.0130	-2.4381	0.0010	-1.2531	0.1964	-1.0182	0.3566	-0.2684	0.8764	-1.2373	0.1520	-1.8956	0.0083	-1.8397	0.0335	-2.2772	0.0023	-1.3919	0.1717	-1.1624	0.2591	-2.1273	0.0041	-2.1167	0.0083	-0.7664	0.3053
AT4G04540	-3.3355	0.0000	-3.8334	0.0000	-1.9972	0.0132	-1.0356	0.3299	-1.1216	0.2621	-2.2933	0.0035	-2.4315	0.0003	-2.9422	0.0001	-3.4616	0.0000	-2.1011	0.0115	-1.6703	0.0592	-3.5479	0.0000	-2.7267	0.0002	-2.3498	0.0006
AT4G04570	-0.5409	0.4892	-2.2700	0.0004	-0.6476	0.5050	-0.9074	0.3535	-0.0343	0.9888	-1.3433	0.0718	-0.8466	0.2244	-0.2859	0.8750	-2.2237	0.0007	-0.3187	0.8389	0.5646	0.5977	-0.6931	0.3699	-0.9444	0.3145	-1.6195	0.0103
AT4G04745	-1.0923	0.1390	-2.0586	0.0037	-0.1623	0.9009	-1.4083	0.1321	-0.0157	0.9969	-0.1092	0.9186	-2.2562	0.0006	-0.3452	0.8509	-1.3038	0.0774	-1.2602	0.1921	-1.6981	0.0439	-1.4696	0.0417	-0.1087	0.9510	-0.5630	0.4306
AT4G04835	-0.0957	0.9302	0.0025	0.9995	1.6819	0.0542	0.5061	0.6892	0.0653	0.9790	0.0901	0.9381	0.5449	0.5247	-0.2526	0.9079	0.1630	0.8735	-0.3791	0.8282	0.3934	0.7755	-0.5666	0.5427	0.2901	0.8666	2.0195	0.0042
AT4G04840	0.4626	0.4749	-1.2607	0.0320	-0.8392	0.2511	-0.3642	0.6975	-1.4911	0.0137	-1.2447	0.0435	-0.1450	0.8427	-1.6183	0.0072	-2.0317	0.0002	-1.1089	0.1254	-0.3638	0.7060	0.9367	0.1101	-0.2887	0.8066	-1.5601	0.0030
AT4G05010	-2.0482	0.0000	-0.4386	0.4056	-0.5487	0.3450	-0.4125	0.5367	-0.3243	0.6796	-0.2499	0.6508	-1.8299	0.0000	-0.5917	0.3656	-0.1899	0.7221	-0.3050	0.7249	-0.3585	0.5985	-1.8178	0.0000	-1.2337	0.0071	-0.4890	0.2455
AT4G05100	-1.1151	0.1026	0.7614	0.3351	0.2193	0.8455	0.0771	0.9574	-0.6997	0.5058	0.5382	0.5084	-2.1954	0.0003	-0.6832	0.5671	0.8542	0.2271	0.6655	0.5659	-0.2875	0.8203	-1.3923	0.0393	0.2499	0.8673	0.0372	0.9619
AT4G05110	0.0106	0.9932	-1.8944	0.0075	-1.3851	0.0999	-0.5695	0.6065	-0.3435	0.8142	-1.9144	0.0121	0.0198	0.9836	-1.7012	0.0321	-2.7149	0.0001	-0.3637	0.8169	0.5530	0.6172	0.1784	0.8598	-1.6339	0.0375	-1.3369	0.0430
AT4G05150	0.6757	0.1979	1.1879	0.0198	1.1518	0.0412	1.3164	0.0292	1.0400	0.0761	1.5615	0.0028	0.6106	0.2337	1.2479	0.0238	1.6322	0.0007	1.1921	0.0424	-0.4942	0.5095	0.8077	0.1212	1.3897	0.0074	2.6817	0.0000
AT4G05170	-0.6724	0.4347	-0.7464	0.4235	-1.3259	0.1631	-0.9828	0.3747	-0.8229	0.4865	-1.8010	NA	-2.0282	0.0043	-0.4078	0.8293	-1.5318	0.0493	-1.3865	0.1718	1.6076	0.0763	-1.0980	0.1759	-0.8285	0.4868	-0.9679	0.1861
AT4G06265	-1.1997	0.0846	-0.3973	0.6620	-0.8758	0.3427	-1.5124	0.0869	-1.9086	0.0117	-1.0804	NA	-1.2322	0.0598	-1.4802	0.0715	-1.2830	0.0749	-1.3488	0.1327	-0.7375	0.4640	-2.0096	0.0030	-1.0576	0.2516	-1.6738	0.0121
AT4G06534	-0.3119	0.7115	0.5748	0.4923	-0.3342	0.7558	1.5525	0.0638	-1.6178	0.0323	1.7654	0.0134	-1.7779	0.0045	0.8694	0.4042	2.0841	0.0012	1.1329	0.2140	-0.4360	0.7032	-0.0944	0.9273	1.5274	0.0393	1.7406	0.0049
AT4G06955	1.7394	0.0173	0.9418	0.2958	1.2200	0.2062	1.6864	0.0758	1.3641	0.1506	0.7799	NA	2.0693	0.0024	1.2722	0.2084	1.2007	0.1306	1.0818	0.3379	1.2593	0.2081	1.8764	0.0101	0.8878	0.4435	0.6848	0.3660
AT4G07190	-1.1865	0.1339	-0.8677	0.3434	-1.0829	0.2791	-1.4978	0.1348	-1.0786	0.3093	-0.9265	NA	-1.8978	0.0081	-1.1338	0.3064	-1.4217	NA	-0.6685	0.6420	-1.7567	0.0481	-2.0513	0.0052	-1.0050	0.3528	-0.7744	NA
AT4G07395	0.0282	0.9794	0.7394	0.4116	0.1738	0.8948	0.0468	0.9780	1.8505	0.0227	0.2361	NA	0.0000	1.0000	0.8592	0.4871	1.1568	NA	0.1372	0.9511	0.1996	0.8966	0.0614	0.9595	2.7330	0.0002	1.4556	NA
AT4G08040	-0.2539	0.7575	-2.1445	0.0005	-0.0969	0.9374	-1.4134	0.0837	-1.2499	0.1073	-1.3655	0.0521	-1.0655	0.0888	-1.3261	0.0856	-2.1072	0.0008	-1.5411	0.0419	0.8346	0.3482	-0.9574	0.1561	-1.5470	0.0282	-0.6482	0.2989
AT4G08400	0.2481	NA	0.2545	0.7829	1.0270	NA	0.0000	NA	2.1260	0.0016	0.0000	NA	0.1242	NA	0.6986	NA	0.0000	NA	0.0000	NA	0.8492	0.3552	0.0000	NA	0.0000	NA	0.4367	NA
AT4G08555	-2.9871	0.0000	-2.8996	0.0000	-2.1598	0.0065	-0.2817	0.8377	-1.7810	0.0329	-2.1432	0.0072	-2.8783	0.0000	-2.0754	0.0097	-2.8162	0.0001	-2.1336	0.0104	-1.8753	0.0292	-2.6819	0.0001	-3.0448	0.0000	-2.4317	0.0004
AT4G08770	-2.1663	0.0017	-2.4356	0.0004	-1.1528	0.2107	-0.7827	0.4757	-0.1534	0.9323	-1.6931	0.0310	-1.9344	0.0041	-1.1852	0.2303	-2.2726	0.0012	-0.4992	0.7335	-0.8407	0.4211	-1.8835	0.0076	-2.1026	0.0050	-0.9652	0.1622
AT4G08870	-0.2773	0.7049	-1.4875	0.0115	-0.9679	0.1881	-0.6547	0.4554	-1.0123	0.1729	-1.7585	0.0043	-0.5037	0.4278	-1.6317	0.0089	-1.7330	0.0023	-0.9821	0.2187	-0.8600	0.2764	-0.4614	0.5082	-0.9286	0.2172	-2.3429	0.0000
AT4G09020	2.2418	0.0000	1.5829	0.0004	2.0494	0.0000	1.4447	0.0083	3.0481	0.0000	1.2600	0.0122	2.6279	0.0000	2.2611	0.0000	1.7610	0.0001	1.7905	0.0002	-0.0476	0.9662	2.7792	0.0000	3.0044	0.0000	1.7239	0.0001
AT4G09200	-0.1398	0.8965	-0.0186	0.9887	0.5179	0.6562	0.2345	0.8753	4.4422	0.0000	-0.2249	0.8360	-0.1650	0.8687	0.0125	0.9963	0.4682	0.6083	0.8594	0.5010	0.9759	0.3678	0.5856	0.5271	0.3184	0.8490	0.7678	0.3056
AT4G09350	-0.9807	0.1723	-1.4278	0.0490	-1.2577	0.1360	-1.0521	0.2746	-1.0143	0.2782	-2.6727	0.0002	-0.7295	0.3139	-1.2492	0.1589	-2.8808	0.0000	-0.7074	0.5536	-0.0891	0.9550	-0.5065	0.5436	-1.0200	0.2666	-2.8942	0.0000
AT4G09890	-1.5479	0.0010	-1.0427	0.0450	-0.6704	0.3062	-0.9092	0.1701	-0.1293	0.9129	-0.3092	0.6186	-2.0711	0.0000	-0.1063	0.9465	-0.8925	0.0802	-0.5248	0.5356	-0.0562	0.9615	-1.0292	0.0389	0.0087	0.9984	-0.5291	0.2707
AT4G09990	-0.5026	0.4695	-0.9107	0.1953	-0.5697	0.5179	-0.5687	0.5472	0.0585	0.9751	-0.9656	0.1720	-0.2031	0.7866	-0.4539	0.7267	-1.2907	0.0434	-0.7420	0.4499	0.1187	0.9290	-0.6430	0.3480	-0.4472	0.6946	-2.2137	0.0003
AT4G10120	-1.5546	0.0143	-2.7188	0.0000	-1.3685	0.0714	-1.5126	0.0642	-0.9034	0.3202	-2.7619	0.0000	-1.4736	0.0166	-1.5401	0.0366	-2.9679	0.0000	-0.9163	0.3452	-0.0294	0.9863	-1.0624	0.1173	-1.3541	0.0698	-2.5919	0.0000
AT4G10150	-2.3674	0.0000	-0.1444	0.8613	0.4259	0.5989	-0.1014	0.9299	-0.5162	0.5788	-0.2566	0.7271	-1.9898	0.0001	-0.3306	0.8014	-0.4921	0.4323	-0.9285	0.2273	0.4228	0.6327	-1.4781	0.0068	0.8227	0.2574	0.4377	0.4263
AT4G10160	-2.3567	0.0002	-0.2759	0.7572	0.5603	0.5318	-0.0880	0.9484	-0.8425	0.3599	0.2560	0.7603	-1.6887	0.0045	-0.5419	0.6654	0.1867	0.8175	-0.1919	0.9105	-0.4788	0.6436	-2.3746	0.0001	0.5797	0.5763	1.0863	0.0606
AT4G10310	-2.3746	0.0000	-1.0405	0.0600	-0.5909	0.4073	-0.9831	0.1502	-0.4300	0.6344	-1.9608	0.0005	-2.5523	0.0000	-0.8539	0.2248	-1.3926	0.0074	-1.4199	0.0165	-0.0472	0.9699	-2.2718	0.0000	-0.5116	0.5456	-0.7777	0.1163
AT4G10500	-1.6321	0.0281	-2.6904	0.0002	-1.8357	0.0285	-1.9748	0.0309	-0.1385	0.9427	-2.0025	0.0131	-2.2752	0.0010	-2.3833	0.0022	-2.3730	0.0012	-1.0020	0.3834	-0.6634	0.5678	-2.0366	0.0052	-2.2006	0.0046	-0.4844	0.5283
AT4G10695	1.4853	0.0472	0.4369	0.6725	1.4544	0.1099	0.7765	0.5018	0.7005	0.5865	0.0200	NA	0.2795	0.7669	1.3894	0.1473	0.1500	0.8841	1.0160	0.3812	1.3333	0.1750	2.0271	0.0047	0.5660	0.6900	1.7025	0.0158
AT4G11000	-1.0569	0.0731	-2.7444	0.0000	-1.1635	0.0956	-1.3070	0.0794	-0.4095	0.7057	-1.1069	0.0880	-2.0021	0.0002	-2.2572	0.0001	-1.6986	0.0033	-1.5582	0.0196	-0.5355	0.5488	-1.9017	0.0006	-1.7095	0.0051	-1.1513	0.0360
AT4G11070	-1.7491	0.0146	-1.9931	0.0056	-1.1781	0.2072	-2.0371	0.0197	-1.3804	0.1256	-1.7316	NA	-2.0212	0.0031	-2.0867	0.0069	-1.1484	NA	-1.8203	0.0316	-1.1040	0.2652	-1.8176	0.0116	-1.6115	0.0480	-1.2550	NA
AT4G11100	-0.4167	0.4826	-0.3341	0.6257	-0.4973	0.5011	-0.6215	0.4166	-0.2440	0.8234	-0.4005	0.5290	-0.5106	0.3531	-0.3290	0.7819	-0.7282	0.1824	-0.5736	0.5051	-0.7678	0.2716	-0.4235	0.4893	-0.6098	0.4311	-2.0543	0.0000
AT4G11170	-2.7027	0.0000	-3.0141	0.0000	-2.3830	0.0007	-1.6298	0.0633	-2.1308	0.0028	-2.1880	0.0030	-2.9162	0.0000	-2.8636	0.0000	-2.6091	0.0001	-2.5014	0.0006	-2.2718	0.0022	-2.9395	0.0000	-3.3017	0.0000	-2.9450	0.0000
AT4G11280	-1.6450	0.0024	-0.7957	0.2227	-1.0696	0.1199	0.3106	0.7506	-1.2447	0.0574	-0.9255	0.1475	-1.7509	0.0007	-1.3233	0.0407	-1.1253	0.0519	-0.7774	0.3630	2.2746	0.0001	-1.7066	0.0016	-1.6053	0.0066	-1.0835	0.0408
AT4G11320	2.1411	0.0001	1.6496	0.0052	0.9610	0.2033	1.3269	0.0805	0.4881	0.6435	1.5674	0.0140	1.3265	0.0187	2.0892	0.0005	2.1229	0.0002	2.9509	0.0000	1.8519	0.0038	1.9468	0.0005	2.2643	0.0001	2.7148	0.0000
AT4G11470	-2.4685	0.0000	-2.9725	0.0000	-1.6582	0.0004	-1.7482	0.0005	-1.8204	0.0001	-1.2818	0.0101	-1.1713	0.0041	-2.2790	0.0000	-2.6678	0.0000	-2.1318	0.0000	-0.7998	0.1686	-2.7843	0.0000	-2.4598	0.0000	-1.8728	0.0000
AT4G11521	-2.1293	0.0002	-1.8662	0.0019	-0.5138	0.5669	-1.1068	0.1787	-0.4544	0.6838	-1.4418	0.0310	-1.8807	0.0008	-1.0806	0.1785	-2.0280	0.0007	-1.8113	0.0077	-0.8642	0.3035	-2.4824	0.0000	-1.3792	0.0456	-1.0420	0.0716
AT4G11850	-1.1380	0.0170	-2.1225	0.0000	-0.8313	0.1726	-1.1811	0.0512	-0.4541	0.5771	-0.8957	0.0976	-1.2482	0.0061	-1.1068	0.0488	-1.5853	0.0008	-0.8636	0.1878	-0.6603	0.3290	-1.2980	0.0061	-1.4035	0.0054	-1.2717	0.0050
AT4G11890	-2.7471	0.0000	-0.8306	0.0993	-0.3470	0.6152	-1.2272	0.0289	0.0788	0.9485	0.0357	0.9611	-2.8593	0.0000	-1.5133	0.0014	-0.3383	0.5249	-0.8280	0.1815	-2.3414	0.0000	-3.2860	0.0000	-1.1390	0.0233	-0.6521	0.1384
AT4G12030	0.6626	0.4030	-2.1514	0.0018	-1.6940	0.0330	0.0771	0.9605	-0.4424	0.7464	-2.0411	0.0069	0.3819	0.6461	-0.0464	0.9880	-1.5063	0.0361	-0.7931	0.4996	0.4817	0.6864	1.1938	0.1039	-0.6452	0.5892	-1.7228	0.0090
AT4G12390	-0.7838	0.2142	-1.6936	0.0041	-1.6285	0.0117	-1.1800	0.1305	-0.8882	0.2716	-1.9828	0.0016	-0.5397	0.4025	-0.5723	0.6067	-2.3089	0.0001	-0.5060	0.6510	0.0947	0.9441	-0.7097	0.2813	-1.2949	0.0554	-2.4564	0.0000
AT4G12400	-1.7549	0.0099	-1.8390	0.0089	-1.7562	0.0230	0.2850	0.8230	-1.4427	0.0811	-1.9234	0.0104	-1.8870	0.0036	-1.5255	0.0632	-1.7540	0.0118	-0.6323	0.6225	-0.6649	0.5302	-1.4579	0.0382	-1.7253	0.0230	-2.5101	0.0001
AT4G12480	1.0917	0.0249	-0.4230	0.4959	-0.3325	0.6574	-0.0909	0.9303	0.3481	0.7055	0.0197	0.9809	0.3709	0.5018	-0.8604	0.1899	-1.2552	0.0111	-0.2992	0.7775	-2.0487	0.0000	0.2066	0.7569	0.0290	0.9863	1.0256	0.0257
AT4G12500	0.8865	0.2239	-0.5851	0.5009	-0.8667	0.3527	-0.4409	0.7013	-1.1377	0.2082	-0.9009	0.2603	0.2043	0.8161	-1.9067	0.0114	-1.6958	0.0152	-1.3065	0.1489	-2.1567	0.0046	0.5950	0.4622	-0.1130	0.9472	0.0183	0.9824
AT4G12520	-1.4931	0.0512	0.4086	0.6927	0.7460	0.4901	-0.7218	0.5435	0.5833	0.6704	0.3668	0.7135	-1.4594	0.0477	-0.3468	0.8616	-0.1967	0.8442	-0.1437	0.9511	0.3024	0.8366	-1.2463	0.1186	1.2033	0.2185	4.0527	0.0000
AT4G12720	-1.2739	0.0055	-1.8569	0.0000	-1.0324	0.0604	-1.1119	0.0631	-0.7828	0.2050	-0.9430	0.0708	-1.8878	0.0000	-1.9131	0.0000	-1.7925	0.0001	-1.4827	0.0040	-0.5396	0.4336	-2.0070	0.0000	-1.6234	0.0006	-1.7967	0.0000
AT4G12735	-0.1259	0.9073	-0.1812	0.8769	-0.3761	0.7607	3.1380	0.0001	0.7748	0.5292	-0.0816	0.9448	-0.6246	0.4620	0.0700	0.9798	-0.1848	0.8560	1.8623	0.0393	0.0984	0.9557	-0.5295	0.5761	-0.4699	0.7555	0.9174	0.2156
AT4G12740	-0.5248	0.4869	-0.8058	0.3052	-0.8775	0.3237	-0.2374	0.8457	0.1382	0.9325	-1.2793	0.0882	-0.5040	0.4858	-0.1317	0.9510	-1.0898	0.1195	-0.8282	0.4287	-0.3589	0.7581	-0.0792	0.9386	-0.6803	0.5271	-2.1675	0.0008
AT4G12830	0.1783	0.8430	-1.0752	0.1435	-0.3345	0.7516	-0.4317	0.6932	-0.2951	0.8347	-1.6373	0.0218	0.4117	0.5871	-0.3567	0.8282	-2.2357	0.0005	-0.7223	0.5156	0.5864	0.5635	0.5100	0.5194	-0.4034	0.7558	-2.4798	0.0000
AT4G13040	-2.4281	0.0000	-1.5162	0.0000	-1.0262	0.0132	-1.6987	0.0000	-0.7371	0.1126	-1.0949	0.0073	-2.6106	0.0000	-1.2813	0.0010	-1.6537	0.0000	-1.4100	0.0004	-1.6874	0.0000	-2.5389	0.0000	-1.0273	0.0109	-1.6418	0.0000
AT4G13280	1.7852	0.0040	0.9403	0.2100	0.6804	0.4653	1.1567	0.1876	0.5226	0.6581	1.6893	0.0154	0.4831	0.5073	1.9888	0.0031	2.0081	0.0014	3.3151	0.0000	1.9756	0.0047	1.7083	0.0065	2.2241	0.0005	2.0883	0.0005
AT4G13395	-2.6029	0.0000	-1.7780	0.0100	-1.6753	0.0283	-0.1922	0.8851	-1.2164	0.1579	-0.8783	0.2650	-2.6003	0.0000	-1.6979	0.0259	-1.8729	0.0056	-2.0169	0.0076	1.2146	0.1655	-1.8412	0.0055	-1.7057	0.0214	-0.0402	0.9595
AT4G13410	-0.5569	0.5265	-2.3842	0.0010	-1.4840	0.0988	-0.5831	0.6279	-0.9210	0.4056	-2.2312	0.0053	-0.6148	0.4581	-0.5314	0.7498	-2.4880	0.0006	-0.1653	0.9403	0.2504	0.8684	-0.5901	0.5122	-1.1839	0.2260	-1.8112	0.0098
AT4G13540	-2.1259	0.0002	-0.9529	0.1631	-0.6389	0.4556	-0.8318	0.3394	-0.4131	0.7171	-0.2343	0.7749	-2.1669	0.0001	-0.3500	0.8112	-0.5685	0.4027	-0.0135	0.9945	-1.1247	0.1479	-1.9097	0.0010	-0.6918	0.4565	-0.1691	0.7983
AT4G13560	-0.4780	0.4892	-0.6081	0.4073	-0.3821	0.6780	-0.3374	0.7432	-0.5778	0.5580	0.1976	0.8110	-1.1873	0.0397	0.0558	0.9795	-0.0876	0.9138	-0.3267	0.8057	0.6242	0.4818	-0.5184	0.4628	0.6074	0.5303	2.0517	0.0002
AT4G13577	0.5060	0.5394	-1.2446	0.1108	-0.5472	0.6010	-0.2454	0.8547	-0.7192	0.5246	0.0927	0.9312	-0.3556	0.6700	0.5702	0.6924	-0.9602	0.2040	0.0036	0.9997	1.5615	0.0620	0.4619	0.5970	2.0909	0.0035	0.5507	0.4344
AT4G13680	-0.2460	0.8049	-0.8175	0.3744	-0.2962	0.8152	-0.6975	0.5545	-0.0443	0.9861	0.0510	NA	-0.0238	0.9815	-0.4691	0.7903	-0.2347	0.8100	0.5461	0.7142	2.1968	0.0058	-0.3125	0.7585	-0.2666	0.8771	-0.2617	0.7491
AT4G14060	2.5185	0.0004	0.5347	0.5916	0.1539	0.9130	0.5667	0.6452	1.3702	0.1484	0.6242	0.5050	1.4918	0.0394	1.2305	0.2353	0.9540	0.2429	3.4797	0.0000	1.5709	0.0898	2.0727	0.0043	1.1855	0.2316	1.4201	0.0453
AT4G14103	1.6745	0.0092	1.7545	0.0087	1.0541	0.2267	1.3438	0.1231	1.8028	0.0106	1.2450	0.1008	2.6450	0.0000	1.6773	0.0223	1.2185	0.0825	1.6883	0.0286	1.0406	0.2572	2.3280	0.0001	1.6746	0.0210	0.9770	0.1393
AT4G14270	1.5896	0.0028	1.9057	0.0003	1.6243	0.0057	1.4714	0.0291	2.2547	0.0000	1.9899	0.0005	1.9294	0.0001	2.0215	0.0003	2.1941	0.0000	1.9571	0.0008	-0.9259	0.2100	2.0154	0.0001	2.4370	0.0000	2.7577	0.0000
AT4G14365	-1.7305	0.0062	-2.7065	0.0000	-1.3070	0.0930	-1.3650	0.1061	-0.7143	0.4803	-1.4189	0.0470	-2.2243	0.0002	-2.2160	0.0008	-2.7790	0.0000	-1.7613	0.0175	-0.9783	0.2679	-2.6814	0.0000	-1.9171	0.0044	-1.5299	0.0119
AT4G14370	-2.0615	0.0000	-2.3717	0.0000	-1.3181	0.0125	-0.9367	0.1402	-1.7451	0.0003	-1.0713	0.0439	-2.7362	0.0000	-2.7582	0.0000	-1.9872	0.0000	-1.7799	0.0005	-1.1507	0.0437	-3.2428	0.0000	-2.7296	0.0000	-2.4621	0.0000
AT4G14390	-1.8141	0.0153	-2.4397	0.0009	-1.7783	0.0395	-1.3369	0.1986	-0.3098	0.8544	-1.8818	0.0219	-1.5314	0.0378	-1.9324	0.0221	-2.4154	0.0010	-0.6618	0.6452	-0.6267	0.6064	-1.7686	0.0198	-2.0769	0.0093	-1.4216	0.0461
AT4G14400	-1.6977	0.0244	-3.0144	0.0000	-1.9593	0.0196	-1.6635	0.0899	-0.9273	0.4132	-2.5042	0.0018	-2.1261	0.0027	-1.5503	0.0967	-2.9361	0.0001	-0.2929	0.8808	-0.0327	0.9877	-1.8089	0.0169	-1.8872	0.0227	-1.9502	0.0061
AT4G14450	-1.7459	0.0099	-2.4909	0.0002	-1.4584	0.0742	-1.0884	0.2557	-1.0793	0.2404	-2.0046	0.0082	-1.8107	0.0048	-1.4095	0.0943	-2.1771	0.0016	-1.8041	0.0249	-0.0671	0.9670	-2.0820	0.0018	-2.2734	0.0016	-0.6407	0.3463
AT4G14465	0.1352	0.8708	-0.5176	0.4923	-0.1232	0.9108	-0.3742	0.7094	0.4060	0.7180	-0.4549	0.5423	0.2001	0.7903	0.4272	0.7451	-0.3079	0.6736	1.1920	0.1160	2.0882	0.0007	0.0910	0.9208	0.5930	0.5439	1.5421	0.0053
AT4G14630	-1.1047	0.1247	-1.8710	0.0086	-2.1414	0.0042	-1.5514	0.0837	-1.5419	0.0589	-2.1363	0.0050	-1.6569	0.0118	-2.0024	0.0081	-2.1525	0.0020	-1.4071	0.1164	-1.1114	0.2344	-1.6017	0.0219	-1.5699	0.0490	-0.3066	0.6795
AT4G14640	-1.8088	0.0098	-1.5163	0.0453	-0.2722	0.8199	-1.7366	0.0529	-0.2170	0.8948	-0.6156	0.4832	-2.4396	0.0002	-0.8931	0.4348	-1.6025	0.0281	-0.7124	0.5696	-1.2721	0.1718	-2.0288	0.0036	-0.8397	0.4367	-0.4999	0.4906
AT4G14690	-1.3828	0.0672	-2.6648	0.0002	-1.5541	0.0772	-0.6566	0.5775	-1.7159	0.0433	-3.0155	0.0001	-0.4722	0.5833	-1.8667	0.0249	-3.3809	0.0000	-1.8233	0.0381	-0.5599	0.6467	-0.7999	0.3436	-2.0479	0.0092	-3.5088	0.0000
AT4G15210	-0.7796	0.3584	-2.1402	0.0050	-1.3840	0.1443	1.1651	0.2798	-0.3643	0.8231	-1.9894	0.0160	-1.0000	0.2033	-1.3379	0.1881	-2.0643	0.0065	-1.2310	0.2540	-1.5919	0.0912	-0.8704	0.3108	-0.7433	0.5580	-1.8119	0.0113
AT4G15233	-0.8732	0.1301	-2.4140	0.0000	-0.7892	0.2820	-1.4555	0.0323	-0.6013	0.4898	-1.4448	0.0161	-1.5187	0.0034	-1.1601	0.0847	-2.3208	0.0000	-1.0364	0.1577	-0.4937	0.5672	-1.1521	0.0412	-1.8858	0.0007	-1.8520	0.0003
AT4G15248	-2.5343	0.0005	-1.2525	0.1431	-1.6015	0.0753	-1.8263	0.0578	-2.1640	0.0070	-2.4526	0.0023	-2.4801	0.0004	-2.4533	0.0019	-1.9941	0.0087	0.5366	0.7304	-0.2338	0.8835	-1.6310	0.0347	-2.0840	0.0097	-1.9744	0.0056
AT4G15280	-1.5029	0.0314	-1.4036	0.0632	-2.1927	0.0037	0.0390	0.9810	-0.9254	0.3582	-0.7053	0.4015	-1.5061	0.0232	-1.1486	0.2314	-0.8303	0.2752	-0.1802	0.9265	-1.1665	0.2114	-1.4972	0.0344	-1.7526	0.0251	0.1835	0.8091
AT4G15340	0.1836	0.8577	0.4729	0.6299	0.5782	0.5964	0.6295	0.5920	-0.1738	0.9232	-0.1033	NA	0.3976	0.6504	0.8259	0.5183	0.3855	0.6667	0.6101	0.6643	0.7535	0.4948	-0.2549	0.8024	0.4313	0.7689	2.2714	0.0010
AT4G15390	1.1261	0.1483	1.2445	0.1354	0.8797	0.3957	-0.4612	0.7195	1.1484	0.2551	-0.4522	0.6429	0.1652	0.8657	1.2194	0.2357	-0.5308	0.5512	1.0150	0.3749	2.2011	0.0068	0.2843	0.7820	0.6883	0.5906	1.4030	0.0461
AT4G15430	-2.3637	0.0000	-1.9241	0.0000	-1.5647	0.0026	-1.5410	0.0085	-2.4794	0.0000	-1.7546	0.0008	-2.2103	0.0000	-1.7574	0.0005	-1.6398	0.0007	-1.2686	0.0289	0.6351	0.3673	-2.2435	0.0000	-2.1277	0.0000	-1.6910	0.0003
AT4G15550	-3.4103	0.0000	-1.4429	0.0094	-0.9993	0.1412	-1.1086	0.1242	-0.7408	0.3449	-1.3737	0.0209	-3.4021	0.0000	-0.7969	0.3258	-1.8152	0.0007	-1.1654	0.0893	-1.0670	0.1247	-2.6894	0.0000	-1.2174	0.0503	-1.8634	0.0002
AT4G15610	-0.1155	0.8927	-0.2998	0.7268	0.2227	0.8298	0.1521	0.9002	-0.5372	0.6106	0.7843	0.2682	-0.4366	0.5284	0.0421	0.9874	0.7000	0.2948	0.8896	0.3252	-0.8162	0.3430	-0.2203	0.7937	-0.2609	0.8448	2.7571	0.0000
AT4G15680	-0.7759	0.3497	1.5234	0.0563	0.6964	0.5207	1.5823	0.1031	-1.5817	0.0753	1.7515	0.0314	-1.2012	0.1080	0.4833	0.7797	2.1678	0.0032	1.3860	0.1620	-0.3938	0.7695	-0.9866	0.2278	-1.1542	0.2462	-0.7310	0.3221
AT4G15975	-0.6023	0.4026	-2.5015	0.0001	-1.0334	0.2102	-0.9272	0.3101	-1.2944	0.0962	-1.3754	0.0543	-0.9272	0.1502	-1.1802	0.1561	-2.2099	0.0005	-0.8718	0.3742	-0.0627	0.9674	-1.2179	0.0651	-1.6685	0.0176	-0.8094	0.1943
AT4G15990	-0.6058	0.3184	0.2609	0.7355	-0.0162	0.9893	0.6706	0.4019	-0.1594	0.9026	0.1323	0.8693	-2.1347	0.0000	0.7012	0.4134	0.4529	0.4682	-0.0984	0.9546	-0.6912	0.3928	-0.4752	0.4671	1.2071	0.0482	1.5780	0.0017
AT4G16140	-0.4341	0.5112	-1.3375	0.0224	-1.3919	0.0283	-0.7878	0.3333	-0.8463	0.2721	-1.7391	0.0039	-0.5349	0.3808	-0.7113	0.4343	-2.0324	0.0002	-0.3925	0.7332	0.9781	0.1862	-0.3137	0.6665	-1.1952	0.0672	-1.3073	0.0138
AT4G16146	0.7416	0.3027	2.0183	0.0017	1.7978	0.0118	1.4448	0.0880	2.7582	0.0000	1.7555	0.0133	1.1313	0.0809	1.8220	0.0104	2.1789	0.0006	1.9698	0.0066	-1.1715	0.1847	2.2477	0.0003	3.3889	0.0000	2.2708	0.0002
AT4G16240	-2.0770	0.0008	-1.4180	0.0362	-1.1568	0.1476	-1.5190	0.0618	-0.9831	0.2568	-1.3150	0.0656	-2.0882	0.0004	-1.0815	0.2132	-1.9757	0.0020	-1.2324	0.1415	-1.3363	0.0937	-1.2255	0.0625	-1.1217	0.1650	-1.1257	0.0653
AT4G16260	-1.6693	0.0082	-1.9675	0.0019	-1.6863	0.0179	-0.5835	0.5672	-1.1918	0.1413	-0.6230	0.4270	-2.2477	0.0001	-0.6855	0.5498	-1.0805	0.1102	-0.0690	0.9734	-1.9830	0.0049	-1.8675	0.0028	-0.9285	0.2950	0.7855	0.2088
AT4G16563	2.1430	0.0000	-0.0496	0.9537	0.8661	0.1858	-0.1301	0.8997	0.1179	0.9274	-0.0429	0.9588	1.1538	0.0182	0.4698	0.6413	0.6397	0.2540	0.3976	0.6972	-0.0009	1.0000	1.3804	0.0059	-0.1753	0.8850	0.7663	0.1215
AT4G16590	-1.0030	0.2103	-2.0088	0.0071	-1.4906	0.0963	0.0301	0.9886	-1.4141	0.1268	-1.6789	NA	-0.4975	0.5675	-1.5442	0.0903	-1.9853	0.0070	-1.7781	0.0458	-0.1459	0.9306	-0.9663	0.2416	-1.7059	0.0405	-1.9311	0.0051
AT4G16670	-2.4218	0.0000	-0.2854	0.6451	0.4062	0.5369	-0.7101	0.2757	0.0353	0.9811	-0.3362	0.5680	-2.2363	0.0000	-0.7702	0.2294	0.0186	0.9789	-0.3646	0.6853	-0.7898	0.2123	-2.1484	0.0000	-0.3017	0.7397	0.3732	0.4168
AT4G16690	-2.2749	0.0001	0.5461	0.4886	0.1428	0.9012	0.1534	0.9022	-1.4544	0.0413	0.6855	0.3556	-1.8089	0.0019	-0.6296	0.5840	0.8212	0.2221	0.4529	0.7198	-1.2124	0.1270	-1.9945	0.0008	-1.2122	0.1059	0.4654	0.4628
AT4G16740	-0.7308	0.3895	0.7085	0.4545	0.6781	0.5423	-0.0961	0.9545	0.9386	0.4050	-0.3436	0.7332	-0.7395	0.3664	0.0251	0.9937	-0.2919	0.7614	-0.0972	0.9681	-0.6447	0.5876	1.8658	0.0134	3.0131	0.0000	0.8889	0.2277
AT4G16780	-2.7663	0.0000	-0.6310	0.3746	-0.0182	0.9882	-0.5615	0.5388	0.1700	0.9017	0.6182	0.3748	-2.4697	0.0000	-0.3122	0.8324	-1.1244	0.0618	-0.4822	0.6652	0.5422	0.5467	-2.9700	0.0000	0.0052	0.9999	-0.2449	0.6960
AT4G16820	-1.4390	0.0537	-2.3095	0.0016	-1.1446	0.2304	0.5185	0.6637	-1.6052	0.0659	-2.1765	NA	-1.9330	0.0054	-1.7112	0.0469	-1.9843	0.0077	-0.9565	0.4081	-0.6372	0.5819	-1.4183	0.0619	-2.3410	0.0022	-1.5455	0.0274
AT4G16880	-1.0797	0.0490	-0.2908	0.6975	-0.5962	0.4364	-0.3842	0.6664	-0.3248	0.7628	-1.8661	0.0013	-0.8229	0.1317	-1.1959	0.0643	-2.2704	0.0000	-0.8748	0.2565	-1.5656	0.0105	-0.1761	0.8186	0.3203	0.7684	-1.4165	0.0059
AT4G16920	-0.3314	0.6477	0.2426	0.7743	0.3454	0.7071	0.1518	0.8958	2.0067	0.0005	0.5592	0.4332	-0.1235	0.8716	0.2688	0.8617	0.4654	0.4894	0.2405	0.8702	0.1439	0.9114	-0.5071	0.4674	0.1241	0.9321	0.0247	0.9721
AT4G16983	-1.0919	0.1736	-1.3479	0.1076	-1.4744	0.1089	-2.1024	0.0221	-1.5199	0.0970	-1.3511	NA	-1.4603	0.0493	-1.4643	0.1272	-1.8521	0.0148	-1.9025	0.0320	-1.1510	0.2645	-1.6710	0.0291	-1.4954	0.0963	-1.8749	0.0076
AT4G17070	-1.1762	0.0185	-2.1197	0.0000	-1.0757	0.0707	-1.0468	0.1140	-0.5198	0.5206	-0.9648	0.0869	-1.0606	0.0292	-0.9291	0.1559	-1.7514	0.0004	-0.8957	0.1925	-0.6538	0.3620	-1.2011	0.0172	-1.6426	0.0014	-0.8857	0.0641
AT4G17090	-2.3157	0.0000	-2.2275	0.0000	-1.8033	0.0029	-2.0531	0.0016	-1.1123	0.1191	-2.3245	0.0001	-1.9434	0.0003	-1.6697	0.0072	-2.3255	0.0000	-0.2341	0.8736	0.2846	0.7894	-1.9112	0.0005	-1.5979	0.0089	-1.6688	0.0020
AT4G17098	-2.5155	0.0001	-2.4882	0.0002	-2.3245	0.0012	-2.4258	0.0018	-2.2203	0.0016	-2.2395	NA	-2.4511	0.0001	-1.8790	0.0114	-2.5726	0.0002	-2.1574	0.0044	-0.3831	0.7443	-2.4824	0.0001	-2.3846	0.0007	-1.7744	0.0064
AT4G17470	1.0400	0.0829	-2.1415	0.0002	-0.8893	0.2501	0.0456	0.9728	-0.4870	0.6444	-1.5083	0.0202	0.2245	0.7592	-0.8184	0.3648	-1.1326	0.0654	-0.6484	0.5198	0.5872	0.5093	0.7218	0.2668	-0.5987	0.5371	-0.6239	0.2838
AT4G17600	0.4653	0.3250	-0.2738	0.6347	-0.3854	0.5408	-0.2212	0.7705	-0.2861	0.7289	-0.7723	0.1096	0.4140	0.3733	0.0546	0.9709	-0.9580	0.0286	-0.7492	0.2024	-0.2749	0.7105	0.3998	0.4271	-0.3346	0.6702	-2.2074	0.0000
AT4G18170	-0.9361	0.0271	-0.7539	0.1095	-0.4302	0.4809	-0.4694	0.4670	-0.9439	0.0582	-0.0493	0.9390	-1.2391	0.0017	-1.1040	0.0200	-0.0097	0.9889	-0.3927	0.6165	-2.1079	0.0000	-1.1411	0.0063	-1.4027	0.0015	-0.8375	0.0388
AT4G18197	-0.1925	0.7512	-2.3052	0.0000	-0.7955	0.1824	-0.6625	0.3273	-0.6503	0.3290	-0.6627	0.2223	-0.5977	0.2158	-0.4441	0.6186	-1.4597	0.0017	-0.9490	0.1163	0.3254	0.6811	-0.4612	0.3945	-1.2151	0.0174	-0.5681	0.2147
AT4G18205	0.0606	0.9486	-2.4844	0.0000	-0.6057	0.5074	-0.5502	0.5845	-0.3591	0.7784	-0.7665	0.3018	-0.2319	0.7700	-0.5266	0.6834	-1.9086	0.0021	-0.2536	0.8744	0.6726	0.4748	-0.1436	0.8785	-1.2703	0.0890	-0.1413	0.8418
AT4G18250	-2.0020	0.0065	-3.1517	0.0000	-1.7050	0.0504	-1.5760	0.1101	-1.1718	0.2494	-1.9346	0.0179	-2.3036	0.0010	-1.6396	0.0683	-2.9009	0.0001	-1.1070	0.3231	-1.2578	0.2100	-2.0505	0.0055	-2.2361	0.0043	-2.3841	0.0007
AT4G18253	-1.5769	0.0199	-2.9474	0.0000	-1.3280	0.1098	-1.6233	0.0610	-0.5653	0.6433	-1.7595	0.0176	-1.7460	0.0068	-1.6836	0.0295	-3.0895	0.0000	-1.8442	0.0186	-1.0222	0.2736	-1.9109	0.0041	-1.7851	0.0152	-1.4142	0.0278
AT4G18270	1.3078	0.0043	1.7128	0.0001	1.2827	0.0132	1.3648	0.0158	1.3682	0.0058	0.7599	0.1652	1.5689	0.0003	1.4320	0.0041	1.2859	0.0064	1.3655	0.0100	0.1379	0.8944	2.3082	0.0000	2.6145	0.0000	1.5246	0.0006
AT4G18340	-1.6566	0.0028	-0.4077	0.5888	-0.6462	0.4205	-0.2467	0.8159	-1.4527	0.0218	-0.0013	0.9988	-2.4001	0.0000	-0.6193	0.5399	0.0633	0.9370	-0.4754	0.6643	-1.1673	0.1042	-1.7966	0.0011	-0.8358	0.2865	-0.8866	0.1055
AT4G18430	-2.2523	0.0018	-2.4143	0.0010	-1.9036	0.0228	-1.3729	0.1738	-1.3947	0.1362	-1.6022	NA	-0.9125	0.2362	-1.8506	0.0286	-1.7814	0.0191	-1.0367	0.3596	-1.2640	0.2012	-2.2081	0.0023	-1.9757	0.0143	-0.8257	0.2583
AT4G18440	0.8043	0.1998	-0.8949	0.1861	-0.2418	0.8068	0.2813	0.7895	0.1387	0.9248	-1.5532	0.0151	0.8334	0.1656	0.2175	0.8965	-1.5007	0.0113	-0.7826	0.3942	0.1413	0.9131	0.6634	0.3198	-0.1873	0.8922	-2.2015	0.0001
AT4G18480	0.4050	0.4314	-0.1514	0.8198	-0.3281	0.6308	-0.2540	0.7447	-0.1941	0.8443	-0.5648	0.2838	0.2936	0.5725	0.1077	0.9397	-0.8011	0.0876	-0.5695	0.4254	-0.3821	0.5921	0.2417	0.6806	-0.4035	0.6029	-2.2757	0.0000
AT4G18810	-0.5144	0.2976	-0.6856	0.1858	-0.5003	0.4312	-0.8797	0.1416	-0.5011	0.4754	-1.5752	0.0010	-0.2453	0.6471	-0.8836	0.1205	-1.4683	0.0009	-0.7984	0.1949	-0.3301	0.6603	-0.1115	0.8673	-0.7415	0.2045	-2.6481	0.0000
AT4G19030	1.5483	0.0321	1.4586	0.0617	1.4306	0.1045	0.2967	0.8266	1.2121	0.2060	-0.0925	0.9356	1.1961	0.0955	1.3522	0.1484	0.3010	0.7499	1.1733	0.2571	2.3581	0.0022	1.8490	0.0093	0.8015	0.4938	0.7938	0.2754
AT4G19170	-1.0736	0.0350	-1.0753	0.0463	-0.0450	0.9654	-1.2297	0.0552	0.3846	0.6810	-1.6541	0.0021	-0.2942	0.6229	-0.7963	0.2709	-2.2621	0.0000	-1.0999	0.0820	-0.4583	0.5622	-0.5950	0.2969	0.1325	0.9154	-0.3140	0.5516
AT4G19370	-1.9450	0.0041	-2.0684	0.0028	-1.1308	0.2061	-1.0020	0.3155	-0.2679	0.8629	-1.0023	0.2126	-1.7590	0.0075	-0.8962	0.4211	-1.8948	0.0066	-0.5108	0.7166	-0.7586	0.4627	-1.9140	0.0050	-1.8308	0.0149	-1.0840	0.1035
AT4G19460	-2.2030	0.0001	-1.6674	0.0048	-1.5560	0.0169	-1.0311	0.1943	-0.8450	0.2982	-2.4222	0.0001	-1.7412	0.0013	-0.8419	0.3304	-1.5843	0.0073	-0.4949	0.6523	-0.0669	0.9612	-1.6997	0.0029	-1.3034	0.0527	-1.3923	0.0127
AT4G19820	-1.3586	0.0049	-1.2263	0.0000	-2.1249	0.0000	-1.5544	0.0081	-2.2109	0.0000	-1.6304	0.0021	-1.8955	0.0000	-1.6728	0.0011	-2.1921	0.0000	-1.6003	0.0032	0.1518	0.8829	-1.4915	0.0018	-2.5422	0.0000	-2.1779	0.0000
AT4G20110	-2.0623	0.0000	-1.4011	0.0057	-0.7113	0.2879	-1.4826	0.0139	-0.4124	0.6479	-0.6168	0.2978	-2.0519	0.0000	-2.1367	0.0000	-1.1517	0.0250	-1.1558	0.0603	-1.8795	0.0003	-1.6963	0.0004	-1.2285	0.0274	-0.4683	0.3496
AT4G20240	0.7360	0.3014	0.9186	0.2274	0.9857	0.2482	1.1147	0.2097	0.3095	0.8297	0.1343	0.8948	0.0573	0.9493	0.9154	0.3511	1.3107	0.0497	1.0168	0.2762	2.3933	0.0002	0.3767	0.6558	0.3451	0.8081	-0.4236	0.5626
AT4G20362	-1.1931	0.1171	-1.2511	0.1272	-1.8140	0.0285	-2.3803	0.0055	-1.6068	0.0623	-1.5430	0.0582	-1.4754	0.0382	-0.9307	0.4313	-1.9742	0.0073	-1.9484	0.0215	-2.1895	0.0071	-1.3894	0.0670	-2.1690	0.0048	-1.7909	0.0098
AT4G20780	-0.5820	0.4868	-1.9459	0.0076	-0.4786	0.6650	-0.0967	0.9509	0.0023	1.0000	-0.7494	0.3857	-0.4000	0.6385	-0.0618	0.9813	-1.4033	0.0597	0.0546	0.9801	0.1983	0.8980	-0.6031	0.4838	-2.9260	0.0000	-0.4849	0.5120
AT4G20835	-1.5666	0.0366	-1.2555	0.1333	-1.0856	0.2705	-1.7947	0.0567	-1.2467	0.2031	-0.9599	NA	-1.8718	0.0083	-1.1838	0.2640	-1.4401	0.0613	-1.3136	0.1978	-1.6804	0.0596	-2.0613	0.0045	-1.9080	0.0162	-1.1862	0.0936
AT4G20860	-1.2611	0.0044	-1.3589	0.0025	-0.2782	0.6967	-0.7474	0.2403	-1.8168	0.0000	-0.5560	0.2989	-2.5583	0.0000	-1.7745	0.0001	-1.3031	0.0039	-0.6774	0.3123	-0.8975	0.1192	-1.8828	0.0000	-2.1715	0.0000	-1.1976	0.0051
AT4G21215	-0.2812	0.6757	0.8399	0.1671	1.6629	0.0029	0.3038	0.7435	2.1472	0.0000	0.7561	0.2211	0.7278	0.1837	2.0256	0.0001	0.8527	0.1303	0.9177	0.2132	-0.1240	0.9163	0.5051	0.4193	2.6413	0.0000	1.6113	0.0012

AT4G21380	-1.4851	0.0313	-2.5373	0.0001	-0.7892	0.4126	-1.6205	0.0647	-0.0252	0.9924	-1.8006	0.0161	-1.4544	0.0289	-1.1796	0.2063	-2.3561	0.0004	-1.5442	0.0689	-1.4807	0.0807	-1.3523	0.0567	-1.1445	0.1974	-1.7784	0.0061
AT4G21390	-1.8841	0.0005	-2.5922	0.0000	-0.8790	0.2280	0.0663	0.9574	-1.6130	0.0071	-0.6153	0.3604	-2.3266	0.0000	-1.7768	0.0028	-1.3444	0.0193	-1.1661	0.1056	-1.0870	0.1334	-2.4692	0.0000	-2.2688	0.0000	-1.5660	0.0033
AT4G21400	-1.7351	0.0000	-2.2430	0.0000	-1.0764	0.0061	-1.0441	0.0179	-1.2988	0.0003	-1.0497	0.0082	-1.8738	0.0000	-0.8257	0.0540	-1.5845	0.0000	-0.8744	0.0458	0.0440	0.9589	-1.6041	0.0000	-1.1534	0.0022	-1.4379	0.0000
AT4G21500	-2.5339	0.0000	-0.5730	0.2140	-0.2488	0.6857	-1.1403	0.0184	-0.6519	0.2166	-0.1389	0.7994	-1.9165	0.0000	-1.0053	0.0255	-0.3404	0.4529	-0.8468	0.0929	-1.0940	0.0180	-2.2763	0.0000	-1.4398	0.0005	-0.4274	0.2791
AT4G21590	1.7920	0.0065	0.5815	0.5242	0.1554	0.9083	0.7343	0.4916	-0.2736	0.8692	-0.0982	NA	2.2253	0.0002	0.2278	0.9125	-0.0500	0.9630	0.8038	0.4964	1.1613	0.2139	1.9366	0.0030	0.9170	0.3769	-0.7800	0.2905
AT4G21680	1.2998	0.0416	-0.2797	0.7594	-0.9498	0.2568	0.6847	0.4812	0.2465	0.8619	-0.3735	0.6496	-0.1924	0.8143	-0.8060	0.4343	0.4248	0.5733	0.7943	0.4336	-0.9981	0.2482	-0.0144	0.9904	-2.3337	0.0003	-0.2890	0.6719
AT4G21840	-1.7633	0.0195	-2.6036	0.0004	-1.6268	0.0698	-1.3391	0.1998	-0.4682	0.7548	-1.6622	0.0478	-0.9689	0.2191	-2.0587	0.0135	-2.5018	0.0008	-1.4027	0.1687	-0.9312	0.3960	-1.8092	0.0175	-1.3714	0.1462	-0.4175	0.6017
AT4G21850	-0.9189	0.1339	-1.8364	0.0015	-1.1270	0.1170	-1.2154	0.1156	-0.6373	0.4904	-1.2603	0.0526	-1.1229	0.0502	-1.2593	0.0747	-2.1711	0.0001	-1.0370	0.1957	0.1153	0.9296	-0.9794	0.1129	-0.5242	0.6066	-0.6827	0.2338
AT4G21903	-0.7125	0.3818	-2.3539	0.0010	-1.1134	0.2384	-0.7090	0.5318	-0.5425	0.6857	-1.4599	0.0716	-1.1357	0.1187	-0.9071	0.4405	-2.1428	0.0031	0.1685	0.9366	0.5143	0.6725	-0.9395	0.2390	-1.4406	0.0978	-0.5769	0.4318
AT4G21920	-1.2539	0.0742	-2.0310	0.0034	-1.3948	0.0934	-0.6755	0.5264	-0.6445	0.5788	-0.5416	0.5266	-1.8424	0.0044	-1.0298	0.3057	-1.3957	0.0505	-0.1097	0.9602	-0.3136	0.8081	-1.1488	0.1118	-0.8796	0.3843	-0.2152	0.7742
AT4G22190	-2.1206	0.0000	-0.6281	0.1424	-0.4065	0.4531	-0.9641	0.0420	-0.5946	0.2469	-0.5897	0.1760	-2.1321	0.0000	-0.8787	0.0475	-1.0823	0.0041	-0.7305	0.1433	-0.0712	0.9339	-2.0047	0.0000	-0.4217	0.4811	0.0689	0.8730
AT4G22200	-2.0517	0.0000	-0.8939	0.0772	-1.0058	0.0663	-1.0288	0.0875	-1.2857	0.0097	-1.0717	0.0359	-2.0451	0.0000	-1.3639	0.0064	-1.2914	0.0053	-0.4486	0.5958	-0.0644	0.9519	-1.5682	0.0004	-0.6771	0.2889	-1.6704	0.0001
AT4G22212	1.3783	0.0469	1.1524	0.1409	0.5407	0.6137	0.6276	0.5709	1.3561	0.1112	0.4458	0.6261	0.8423	0.2393	2.1342	0.0028	1.4227	0.0462	2.4919	0.0004	2.1305	0.0042	1.3139	0.0644	1.2923	0.1318	1.7487	0.0073
AT4G22230	0.8501	0.3013	1.1293	0.1913	0.4731	0.6896	0.1451	0.9274	1.0179	0.3475	0.9032	NA	1.3363	0.0649	0.9889	0.4026	0.8474	NA	0.5710	0.7059	-0.0705	0.9699	0.8258	0.3333	2.0840	0.0076	1.0551	NA
AT4G22410	0.3455	0.7165	0.7140	0.4417	-0.0042	0.9985	0.3024	0.8259	3.1726	0.0000	0.1319	0.9071	0.2819	0.7615	0.6745	0.6448	0.4821	0.5889	0.3657	0.8326	-0.1001	0.9541	-0.2614	0.8028	1.0946	0.2802	0.7374	0.3189
AT4G22690	-0.4013	0.5371	-2.3105	0.0000	-0.9002	0.1994	-0.4878	0.5753	-0.5267	0.5649	-1.0108	0.1001	-0.5317	0.3705	-0.7728	0.3517	-1.8750	0.0004	-0.5035	0.6212	0.5321	0.5256	-0.5275	0.4098	-0.9552	0.1667	-1.0451	0.0431
AT4G22770	1.6717	0.0005	1.2123	0.0233	1.7919	0.0004	1.2016	0.0631	2.4403	0.0000	1.8408	0.0005	1.9168	0.0000	2.1402	0.0000	1.1847	0.0245	1.7163	0.0015	1.0202	0.1225	1.7819	0.0002	1.0431	0.0935	1.6261	0.0007
AT4G22840	-0.4667	0.4919	-1.7260	0.0031	-0.4649	0.5929	-0.7902	0.3527	0.5025	0.6220	-1.6152	0.0114	-0.0840	0.9142	-0.3611	0.7934	-2.1553	0.0002	-1.3508	0.0583	-0.4556	0.6317	-0.3381	0.6510	-0.2679	0.8327	-0.7732	0.1717
AT4G23030	-2.1411	0.0000	-0.8698	0.0339	-0.3630	0.5137	-0.7973	0.1130	-0.9632	0.0281	-0.6314	0.1571	-1.7393	0.0000	-0.8287	0.0734	-0.9465	0.0178	-1.0527	0.0195	-0.8924	0.0588	-2.1046	0.0000	-0.4582	0.4367	0.1005	0.8124
AT4G23130	-0.6867	0.4183	-2.4013	0.0009	-0.9232	0.3643	-1.0978	0.3026	0.0883	0.9677	-2.0039	0.0131	-1.0443	0.1701	-1.1533	0.2796	-2.6066	0.0003	-0.5103	0.7404	-0.2565	0.8654	-1.0413	0.1973	-1.2672	0.1816	-1.6250	0.0204
AT4G23140	-1.6318	0.0314	-2.4047	0.0011	-1.0641	0.2913	-1.4838	0.1394	0.5021	0.7309	-1.5013	0.0747	-1.4760	0.0459	-1.3035	0.2031	-2.3470	0.0016	-1.0525	0.3617	-0.6992	0.5516	-2.0325	0.0063	-1.4790	0.1038	-1.2362	0.0865
AT4G23150	-1.8943	0.0105	-2.5784	0.0004	-1.6480	0.0616	-1.6368	0.0938	-0.4923	0.7349	-1.7447	0.0344	-1.9000	0.0077	-1.9460	0.0203	-2.6989	0.0002	-1.3464	0.1896	-1.3532	0.1689	-1.9783	0.0077	-1.7515	0.0381	-1.6419	0.0205
AT4G23170	-1.1836	0.0561	-2.1598	0.0002	-0.9959	0.2038	-1.0432	0.2170	-0.4051	0.7308	-1.4455	0.0310	-1.2021	0.0434	-1.3410	0.0654	-2.2147	0.0002	-1.3771	0.0671	-0.3538	0.7431	-1.3024	0.0360	-1.2646	0.0776	-0.0613	0.9310
AT4G23190	-1.5356	0.0000	-1.6933	0.0000	-0.6960	0.0534	-0.7710	0.0464	-1.3144	0.0000	-0.5909	0.0888	-2.2819	0.0000	-2.1303	0.0000	-1.3969	0.0000	-1.4489	0.0000	-1.2990	0.0000	-2.1523	0.0000	-1.5267	0.0000	-1.4984	0.0000
AT4G23200	-0.4599	0.5956	-2.6562	0.0001	-0.9057	0.3519	-1.2955	0.1834	-0.7979	0.4726	-1.7588	0.0246	-1.0599	0.1439	-1.4016	0.1205	-2.8504	0.0000	-1.0660	0.3122	-0.0280	0.9894	-0.8118	0.3153	-0.9514	0.3549	-1.7197	0.0107
AT4G23210	-3.3804	0.0000	-2.6110	0.0000	-0.9172	0.0342	-2.2044	0.0000	-0.8523	0.0553	-1.3194	0.0011	-3.5080	0.0000	-2.6457	0.0000	-2.7882	0.0000	-2.3854	0.0000	-2.9109	0.0000	-3.4210	0.0000	-2.1199	0.0000	-2.0649	0.0000
AT4G23230	-1.4215	0.0458	-2.2502	0.0011	-0.9527	0.3148	-1.5129	0.1000	-0.0722	0.9732	-1.3280	0.0933	-1.7751	0.0078	-1.2727	0.1702	-2.3524	0.0007	-1.1041	0.2757	-0.8072	0.4351	-1.6546	0.0193	-1.2616	0.1518	-1.2501	0.0619
AT4G23260	-0.5655	0.3394	-2.1224	0.0000	-0.9184	0.1750	-0.5341	0.5195	-0.5717	0.4987	-1.1093	0.0611	-0.6394	0.2530	-1.4677	0.0119	-1.9170	0.0002	-0.7832	0.3248	-0.4345	0.6136	-0.6022	0.3193	-1.4825	0.0089	-1.5698	0.0018
AT4G23280	-1.8995	0.0009	-2.6127	0.0000	-0.7194	0.3695	-1.6087	0.0262	-1.4265	0.0342	-1.2808	0.0522	-2.0279	0.0002	-2.0469	0.0009	-2.0165	0.0007	-1.7570	0.0087	-1.6489	0.0155	-1.8725	0.0011	-1.6811	0.0085	-1.3021	0.0214
AT4G23310	-1.7408	0.0172	-3.3919	0.0000	-2.2963	0.0031	-2.1005	0.0186	-1.1707	0.2361	-2.0612	0.0100	-2.6620	0.0001	-2.4046	0.0018	-2.8695	0.0001	-1.7125	0.0560	-1.0517	0.3033	-2.5877	0.0002	-2.4331	0.0012	-2.0072	0.0038
AT4G23700	-2.0603	0.0006	-2.5323	0.0000	-1.4622	0.0415	-1.1413	0.1792	-1.1056	0.1690	-1.5564	0.0228	-2.0779	0.0003	-2.4828	0.0001	-2.3033	0.0002	-2.0128	0.0032	-2.0908	0.0018	-2.5905	0.0000	-1.5815	0.0206	-1.1109	0.0613
AT4G23810	-2.4474	0.0000	-2.5521	0.0000	-1.2182	0.0165	-1.3711	0.0124	-0.7597	0.2097	-1.1863	0.0178	-2.1350	0.0000	-1.6267	0.0005	-2.0657	0.0000	-1.5822	0.0014	-0.4481	0.5237	-2.7490	0.0000	-1.5190	0.0012	-0.7152	0.1040
AT4G23870	-0.9286	0.1637	1.0796	0.1250	1.1714	0.1336	0.5851	0.5564	-0.3066	0.8202	1.9191	0.0043	-1.0247	0.1050	0.6116	0.6071	1.5792	0.0128	1.1182	0.1937	-0.0169	0.9944	-0.6356	0.3841	1.4375	0.0434	2.5454	0.0000
AT4G24110	-1.1142	0.1230	-1.9745	0.0047	-1.1623	0.1906	0.0155	0.9935	-1.0009	0.3032	-0.9245	0.2535	-2.2546	0.0004	-0.9653	0.3636	-1.4404	0.0445	-0.8352	0.4624	0.1394	0.9277	-1.7092	0.0137	-2.0849	0.0043	-2.1591	0.0010
AT4G24150	-1.7660	0.0027	0.2863	0.7312	0.3676	0.6887	0.1906	0.8676	0.3836	0.7361	0.4701	0.5215	-2.0242	0.0003	0.0864	0.9681	0.8842	0.1548	0.8115	0.3630	0.6041	0.4947	-1.8371	0.0018	0.3597	0.7598	0.8837	0.1163
AT4G24350	-0.6023	0.4045	-1.7831	0.0054	-1.1042	0.1708	0.2450	0.8360	-0.9165	0.3044	-1.7540	0.0112	-0.7695	0.2488	-0.6975	0.5342	-2.0922	0.0008	-0.3650	0.7959	-0.2084	0.8739	-0.3803	0.6408	-0.8940	0.3175	-2.1175	0.0004
AT4G24700	-2.2781	0.0001	-1.3913	0.0374	-1.8618	0.0063	-1.5619	0.0489	-1.6852	0.0141	-2.1228	0.0017	-2.0238	0.0005	-1.8201	0.0078	-1.9251	0.0021	-0.4901	0.6941	-0.2060	0.8739	-1.6086	0.0103	-1.6467	0.0163	-2.6025	0.0000
AT4G24930	-0.5074	0.2049	-0.3570	0.4455	-0.4978	0.3254	-0.6472	0.2117	-0.9529	0.0214	-0.6842	0.1049	-0.2419	0.5782	-0.5973	0.2588	-0.9387	0.0131	-1.0679	0.0119	-0.6885	0.1585	-0.6304	0.1101	-1.0865	0.0057	-2.1707	0.0000
AT4G24970	-0.6215	0.2766	-1.7563	0.0006	-1.2903	0.0311	-0.9208	0.2040	-0.3663	0.7120	-1.4409	0.0124	-0.9556	0.0643	-0.5514	0.5597	-1.3393	0.0120	-1.1050	0.0988	-0.4346	0.6066	-0.7478	0.1884	-1.4178	0.0127	-2.1647	0.0000
AT4G25070	-2.8478	0.0000	-2.7083	0.0000	-1.4294	0.0216	-1.8630	0.0038	-0.9784	0.1735	-1.2122	0.0496	-2.5109	0.0000	-2.3315	0.0000	-2.4800	0.0000	-1.8285	0.0027	-1.7051	0.0055	-2.9409	0.0000	-2.5145	0.0000	-2.3094	0.0000
AT4G25100	1.9933	0.0000	0.6201	0.3226	-0.4736	0.5358	0.3134	0.7254	-1.3226	0.0217	0.1722	0.8128	2.2979	0.0000	0.1602	0.9172	0.2712	0.6721	0.2679	0.8229	0.6112	0.4224	1.5727	0.0018	-0.2262	0.8438	-0.1030	0.8612
AT4G25110	-1.0237	0.1289	-3.0480	0.0000	-1.8386	0.0087	-2.2547	0.0022	-0.7608	0.4383	-1.5053	0.0338	-1.2816	0.0418	-1.4210	0.0649	-2.8266	0.0000	-1.7638	0.0173	-1.1992	0.1521	-1.2400	0.0634	-2.3068	0.0004	-1.8744	0.0020
AT4G25260	-2.4694	0.0000	-0.4291	0.4337	0.1506	0.8416	-0.8139	0.1735	0.3923	0.6061	-0.4740	0.3685	-2.0585	0.0000	-0.2158	0.8516	-0.9645	0.0327	-1.1558	0.0238	-1.0259	0.0520	-2.3629	0.0000	0.2376	0.7969	0.2838	0.5338
AT4G25290	-0.4869	0.3303	-1.1088	0.0178	-1.1294	0.0265	-0.6181	0.3472	-1.3816	0.0032	-1.7774	0.0002	-0.4983	0.2987	-1.3502	0.0052	-1.9451	0.0000	-1.1834	0.0244	-0.3152	0.6821	-0.3705	0.4964	-1.0032	0.0527	-2.4523	0.0000
AT4G25350	-0.3280	0.6392	-1.8371	0.0014	-0.1729	0.8608	-0.9417	0.2377	-0.1210	0.9325	-0.8527	0.1987	-0.6692	0.2582	-1.0077	0.1876	-2.1213	0.0002	-0.6558	0.4911	-0.4922	0.5848	-0.3165	0.6657	-0.8650	0.2658	-1.4522	0.0087
AT4G25390	-2.0840	0.0001	-1.8478	0.0010	-1.1299	0.1049	-0.9564	0.2323	-1.0751	0.1357	-1.2773	0.0438	-2.0854	0.0001	-1.3257	0.0478	-1.7155	0.0025	-0.9547	0.2351	-0.7071	0.3933	-2.0642	0.0001	-1.4858	0.0169	-1.4195	0.0087
AT4G25420	0.0239	0.9741	0.1616	0.8227	0.8613	0.1496	0.2277	0.7923	0.7139	0.2792	0.0167	0.9842	0.3626	0.4976	0.8030	0.2177	0.1907	0.7595	0.2589	0.8132	2.2089	0.0000	0.6127	0.2431	1.3396	0.0075	0.3023	0.5550
AT4G25470	0.7807	0.2821	2.5420	0.0001	2.4696	0.0002	2.2507	0.0031	2.1078	0.0018	2.3381	0.0008	1.4152	0.0271	1.9489	0.0062	2.6313	0.0000	1.0804	0.2520	4.3253	0.0000	1.3740	0.0430	2.1306	0.0017	3.2592	0.0000
AT4G25480	-0.0360	0.9741	2.5172	0.0002	2.7393	0.0001	2.0070	0.0209	2.7734	0.0001	1.9815	0.0105	0.4485	0.5912	2.2443	0.0028	3.0217	0.0000	0.9893	0.3668	0.8870	0.3913	1.5745	0.0298	3.0564	0.0000	2.3604	0.0004
AT4G25490	0.8712	0.2604	3.1059	0.0000	2.9349	0.0000	2.8084	0.0003	3.1032	0.0000	3.3978	0.0000	1.0309	0.1553	3.1415	0.0000	3.5362	0.0000	2.3476	0.0024	2.5203	0.0007	0.8504	0.2879	3.5337	0.0000	3.9566	0.0000
AT4G25810	-0.2616	0.5903	-2.0905	0.0000	-1.2012	0.0055	-1.0993	0.0259	-1.9714	0.0000	-0.4778	0.3157	-1.9421	0.0000	-2.1561	0.0000	-1.5179	0.0001	-1.6145	0.0001	-1.3655	0.0016	-1.4789	0.0001	-1.8001	0.0000	-1.1727	0.0021
AT4G25940	-1.0022	0.0381	-2.2426	0.0000	-0.8431	0.1637	-1.4237	0.0130	-0.7150	0.2802	-0.5213	0.3638	-1.3537	0.0026	-0.6244	0.4155	-1.2907	0.0072	-1.2287	0.0290	-0.6248	0.3615	-1.1962	0.0124	-1.0004	0.0751	-1.0924	0.0158
AT4G26120	-3.1847	0.0000	-1.6523	0.0000	-1.0636	0.0115	-1.4069	0.0011	-0.7597	0.1035	-1.0328	0.0138	-3.2513	0.0000	-1.5793	0.0000	-1.8460	0.0000	-1.3595	0.0009	-1.1403	0.0076	-3.1587	0.0000	-1.4390	0.0002	-0.6934	0.0577
AT4G26150	-0.8332	0.1230	-0.2879	0.6865	-0.7076	0.3099	-0.6706	0.3813	-2.0226	0.0001	-1.0418	0.0723	-0.9848	0.0524	-1.2563	0.0349	-0.2673	0.6752	0.8958	0.2085	0.9330	0.1665	-0.4564	0.4594	-1.6568	0.0020	-0.7379	0.1407
AT4G26200	-1.1810	0.0543	-2.0354	0.0007	-1.4743	0.0340	-1.9707	0.0060	-1.9133	0.0026	-2.6421	0.0001	-1.7449	0.0020	-3.1435	0.0000	-2.1037	0.0005	-1.7626	0.0111	-0.7203	0.4142	-1.7591	0.0031	-2.7324	0.0000	-2.1251	0.0003
AT4G26530	0.8935	0.0360	-0.8718	0.0557	-0.4719	0.4305	-0.4580	0.4817	0.1407	0.8856	-2.0502	0.0000	1.1051	0.0058	-0.6420	0.3003	-2.2662	0.0000	-1.3617	0.0033	-0.3560	0.5994	1.2167	0.0032	1.4828	0.0005	-0.3795	0.3708
AT4G26660	0.6380	0.2303	0.1337	0.8589	-0.4273	0.5637	0.0672	0.9507	0.4235	0.6283	-0.2953	0.6465	0.3761	0.5012	0.5170	0.5641	-0.4399	0.4403	-0.3237	0.7603	-0.2075	0.8310	0.5643	0.3133	-0.2311	0.8318	-2.2830	0.0000
AT4G26670	2.4739	0.0000	2.2218	0.0000	1.7939	0.0003	2.0703	0.0001	2.5130	0.0000	1.9877	0.0001	2.9164	0.0000	2.4628	0.0000	2.4869	0.0000	2.5400	0.0000	0.4418	0.5689	3.0429	0.0000	2.1652	0.0000	2.1553	0.0000
AT4G26850	-1.7862	0.0028	-1.8510	0.0024	-1.7373	0.0095	-1.2496	0.1253	-1.5162	0.0279	-2.4602	0.0001	-1.7426	0.0026	-1.8715	0.0042	-2.5470	0.0000	-0.1201	0.9504	0.7857	0.3722	-1.2880	0.0408	-1.2406	0.0898	-2.0928	0.0003
AT4G27030	-0.7695	0.2154	-1.7221	0.0028	-1.6629	0.0081	-1.4596	0.0442	-2.2268	0.0001	-2.5206	0.0000	-0.8321	0.1593	-1.9359	0.0013	-2.9378	0.0000	-1.3865	0.0469	0.3037	0.7728	-0.8431	0.1784	-2.2411	0.0001	-2.9112	0.0000
AT4G27260	-2.2659	0.0000	0.3959	0.6114	0.4617	0.5965	0.3508	0.7258	-0.2608	0.8381	0.2921	0.7058	-2.6382	0.0000	-0.1572	0.9283	0.3666	0.6011	-0.2646	0.8482	-0.8329	0.3042	-2.1321	0.0001	0.6870	0.4415	1.0623	0.0547
AT4G27280	-2.0183	0.0000	-1.1491	0.0283	-1.2511	0.0258	-0.8739	0.2049	-1.4946	0.0041	-1.0415	0.0603	-2.4506	0.0000	-1.6996	0.0010	-1.2272	0.0153	-1.5328	0.0057	1.0696	0.0816	-1.9007	0.0000	-0.9978	0.0940	-0.5658	0.2478
AT4G27290	-1.9891	0.0051	-0.3526	0.7273	0.1184	0.9314	-0.7179	0.5186	-1.3762	0.1281	-0.0345	0.9773	-2.7720	0.0000	-0.8272	0.5003	0.3305	0.7097	-0.3234	0.8483	-1.6228	0.0668	-1.9617	0.0060	-0.3979	0.7888	0.3792	0.6145
AT4G27300	-1.4350	0.0206	-2.4579	0.0000	-0.9901	0.2186	-0.8622	0.3413	-1.0880	0.1777	-0.8357	0.2492	-1.8300	0.0017	-0.9789	0.2710	-1.6794	0.0071	0.1611	0.9275	-0.0141	0.9966	-1.4090	0.0252	-0.7803	0.3985	-0.9956	0.0944
AT4G27360	-1.8153	0.0138	-1.4822	0.0661	-1.6140	0.0656	-1.0621	0.3273	-1.7138	0.0455	-1.9225	0.0168	-1.5527	0.0326	-2.0768	0.0103	-2.0739	0.0048	0.7563	0.5710	1.0293	0.3301	-1.4540	0.0595	-1.9420	0.0149	-1.7840	0.0104
AT4G27410	-2.3404	0.0000	0.3212	0.6670	0.2840	0.7487	0.3542	0.7013	-0.5621	0.5252	1.0635	0.0821	-2.1556	0.0000	-0.5030	0.6384	0.9093	0.1151	0.0823	0.9633	-1.7147	0.0042	-1.8894	0.0003	0.5102	0.5838	0.1847	0.7569
AT4G27440	3.2622	0.0000	2.0255	0.0000	1.7913	0.0000	2.0395	0.0000	2.4659	0.0000	1.5723	0.0004	3.3665	0.0000	2.5248	0.0000	2.0239	0.0000	1.5947	0.0003	0.2304	0.7626	3.2132	0.0000	2.6473	0.0000	1.4951	0.0001
AT4G27590	-2.5399	0.0001	-0.2912	0.7547	-0.6532	0.4910	-0.6969	0.4866	-0.5663	0.6224	-0.4430	0.5943	-2.8048	0.0000	-0.1067	0.9628	0.0568	0.9513	-0.1064	0.9593	-1.8624	0.0159	-2.6047	0.0000	0.2694	0.8505	-0.8156	0.2089
AT4G27652	0.1769	0.8509	0.1764	0.8635	-0.3705	0.7344	0.7596	0.4604	-0.7089	0.5162	-0.6599	0.4281	0.1654	0.8519	-0.5734	0.6786	-0.5628	0.4727	-0.0799	0.9710	3.3645	0.0000	0.3754	0.6681	-0.5069	0.6906	1.6098	0.0111
AT4G27654	0.7610	0.3581	0.2546	0.8156	-0.2530	0.8396	1.3682	0.1750	-0.1116	0.9562	-0.2084	NA	0.6290	0.4459	-0.3092	0.8759	0.0463	0.9661	0.2146	0.9170	3.4771	0.0000	0.5562	0.5404	0.2128	0.9047	1.7400	0.0127
AT4G27657	-0.3383	0.6861	0.0393	0.9715	-0.5129	0.6079	0.5543	0.6013	-1.3546	0.0961	-0.5707	0.4909	-0.7181	0.3088	-1.1325	0.2146	-0.3687	0.6497	0.0754	0.9724	3.1336	0.0000	0.1917	0.8423	-0.7544	0.4704	0.9898	0.1184
AT4G27820	-0.5827	0.2617	-0.2339	0.7305	-0.6728	0.2931	-1.0633	0.0872	-0.2521	0.8013	-2.1835	0.0000	-0.4482	0.3899	-0.5361	0.5214	-1.2941	0.0073	0.2111	0.8532	-0.2870	0.7338	0.4938	0.3693	0.3970	0.6428	-0.6123	0.1897
AT4G28350	-1.3324	0.0343	-2.2185	0.0002	-0.7359	0.3989	-1.1877	0.1598	-0.7580	0.4220	-1.2955	0.0632	-1.4177	0.0184	-0.9387	0.3077	-2.2087	0.0003	-0.9714	0.2840	-0.9699	0.2548	-1.2710	0.0481	-0.7936	0.3919	-1.2265	0.0388
AT4G28460	-1.5941	0.0366	-1.7165	0.0306	-1.4889	0.1051	-0.4777	0.7113	-1.5147	0.0997	-1.6316	NA	-2.1882	0.0020	-1.5185	0.1089	-1.5737	0.0430	-0.9986	0.4023	-1.2240	0.2298	-2.3327	0.0013	-1.6772	0.0507	-0.4034	0.6148
AT4G28490	-1.7762	0.0000	-1.3564	0.0003	-0.8076	0.0821	-1.1753	0.0114	-0.6522	0.2021	-1.2903	0.0018	-2.0395	0.0000	-1.2249	0.0028	-1.3754	0.0003	-1.4390	0.0004	-0.9005	0.0536	-2.1792	0.0000	-1.0674	0.0100	-0.9223	0.0119
AT4G28660	0.5122	0.3339	-0.5940	0.2998	-0.7215	0.2499	-0.4335	0.5687	-0.7234	0.2701	-1.1463	0.0290	0.7355	0.1281	-0.1143	0.9401	-1.4130	0.0029	-0.9608	0.1202	-0.0877	0.9355	0.5320	0.3279	-0.8655	0.1456	-2.4145	0.0000
AT4G28700	-0.8399	0.2699	-1.5170	0.0444	-0.7020	0.4889	-0.5661	0.6156	-0.0625	0.9780	-1.9456	0.0126	-0.4634	0.5645	-0.7072	0.5852	-2.2095	0.0019	-1.5233	0.0865	-0.6776	0.5305	-1.2309	0.0945	-0.0344	0.9888	-1.4270	0.0343
AT4G28720	-2.0654	0.0000	-0.0788	0.9189	0.7080	0.2764	-0.3522	0.6637	0.3272	0.7297	-0.7335	0.1971	-2.5414	0.0000	0.0047	0.9979	-0.7309	0.1640	-1.1125	0.0674	-0.1812	0.8529	-2.4951	0.0000	0.6329	0.3789	-0.0413	0.9429
AT4G28740	-0.2970	0.6458	-0.9198	0.1123	-0.8616	0.1975	-0.7892	0.2876	-0.1985	0.8649	-1.6384	0.0033	-0.0302	0.9671	-0.6617	0.4323	-1.8168	0.0004	-0.4317	0.6660	0.1512	0.8916	0.1337	0.8620	-0.4966	0.5720	-2.1836	0.0000
AT4G28780	1.7395	0.0000	-0.0415	0.9540	0.4925	0.4218	0.6520	0.2960	0.3159	0.6997	0.3377	0.5376	1.4201	0.0004	0.2221	0.8439	-0.3346	0.5112	0.1328	0.9132	1.4766	0.0014	2.0584	0.0000	0.3720	0.6321	-0.1143	0.8146
AT4G29060	0.0047	0.9959	-0.6125	0.2765	-0.7220	0.2429	-0.6121	0.3823	-0.4668	0.5522	-0.9579	0.0690	-0.0722	0.9097	-0.0748	0.9648	-1.1529	0.0163	-0.7676	0.2540	-0.4451	0.5438	-0.0203	0.9809	-0.6666	0.3155	-2.1131	0.0000
AT4G29190	0.4165	0.3896	0.6122	0.2202	1.0322	0.0340	0.7377	0.2142	0.9856	0.0460	1.4515	0.0014	0.4867	0.2835	1.0980	0.0220	1.0552	0.0149	0.7566	0.1973	-0.7661	0.1746	0.3869	0.4469	1.7431	0.0000	2.3821	0.0000
AT4G29610	1.8365	0.0043	1.0417	0.1732	1.1632	0.1689	0.9615	0.3166	1.3971	0.0794	0.4032	0.6465	1.8890	0.0022	1.5582	0.0424	0.8581	0.2405	0.2023	0.9152	1.0463	0.2493	2.2479	0.0003	1.4658	0.0549	0.6672	0.3220
AT4G29780	-0.5161	0.4181	-0.1402	0.8690	-0.4747	0.5676	0.3078	0.7522	-0.7828	0.3246	-0.8902	0.1642	-1.3082	0.0143	-0.8999	0.2518	-0.9153	0.1213	-1.0521	0.1559	3.1434	0.0000	-0.8698	0.1449	-0.9275	0.1994	0.0499	0.9390
AT4G30180	-2.2110	0.0000	-0.6577	0.2703	-0.6385	0.3527	-0.4955	0.5222	0.4214	0.6271	-0.2440	0.7151	-2.1587	0.0000	0.1414	0.9219	-0.6968	0.2010	-0.4949	0.5812	-0.4333	0.5856	-2.0193	0.0000	-0.4293	0.6319	0.7245	0.1261
AT4G30230	-1.1019	0.1247	-1.5633	0.0328	-0.8698	0.3548	-0.8132	0.4335	-0.4882	0.7059	-1.8176	0.0172	-0.6717	0.3640	-0.3519	0.8417	-1.8977	0.0068	-1.3399	0.1402	-0.6316	0.5548	-0.5696	0.4891	-1.2529	0.1472	-2.1404	0.0013
AT4G30280	-0.4541	0.4468	-1.1625	0.0338	-0.2795	0.7378	-0.7420	0.3206	-0.9294	0.1613	-0.5937	0.3356	-2.1717	0.0000	-1.2648	0.0330	-1.8918	0.0002	-1.4774	0.0123	-0.7472	0.2993	-1.8311	0.0002	-0.6194	0.4322	-0.8616	0.0822
AT4G30290	-2.0751	0.0004	1.2322	0.0572	0.9498	0.2233	-0.2219	0.8465	0.4198	0.7136	0.7367	0.3009	-3.0225	0.0000	0.0552	0.9799	0.4764	0.4950	-0.2440	0.8744	0.1635	0.9015	-2.1393	0.0002	1.8859	0.0023	1.7031	0.0026
AT4G30430	-1.5421	0.0444	-1.4865	0.0710	-1.6079	0.0736	-1.5601	0.1197	-2.3842	0.0018	-1.9093	NA	-2.0608	0.0040	-2.0063	0.0165	-2.1487	NA	-1.7615	0.0560	-0.6969	0.5549	-2.4713	0.0006	-2.5161	0.0008	-1.8212	NA
AT4G30640	-0.8706	0.2267	-2.0964	0.0027	-0.9428	0.3012	-1.8884	0.0253	0.2046	0.8942	-1.3344	NA	-0.9298	0.1696	-1.1755	0.1991	-1.7707	0.0123	-1.5698	0.0656	-1.9291	0.0160	-1.1843	0.0944	-0.9382	0.3355	-0.8742	0.1918
AT4G30650	1.9327	0.0014	1.9402	0.0017	1.7061	0.0132	1.3622	0.0957	3.2754	0.0000	1.4131	0.0412	2.2934	0.0001	2.0494	0.0017	1.9404	0.0017	1.9835	0.0040	0.4405	0.6810	3.0976	0.0000	3.8944	0.0000	2.8246	0.0000
AT4G30660	1.2758	0.0990	1.3676	0.0974	0.6187	0.5813	1.0833	0.3144	1.5656	0.0804	0.4459	0.6479	0.6505	0.4317	1.2247	0.2400	1.0856	0.1764	0.8110	0.5328	-0.1740	0.9170	1.6258	0.0319	1.7237	0.0412	3.3360	0.0000
AT4G31230	-1.0158	0.1678	-2.0427	0.0044	-1.1288	0.2130	-1.4763	0.1087	-0.5041	0.6989	-1.7623	NA	-1.0660	0.1263	-1.4243	0.1030	-1.2746	0.0846	-1.1111	0.2696	-0.4090	0.7388	-1.1060	0.1364	-0.8507	0.4228	-1.0703	0.1156
AT4G31370	-2.2277	0.0021	-1.4697	0.0699	0.5248	0.6384	-1.3933	0.1677	1.0887	0.2784	-1.7501	NA	-1.7549	0.0135	-0.8107	0.5346	-1.3875	0.0754	-1.5056	0.1188	-0.4923	0.7004	-2.3825	0.0009	-0.1179	0.9485	-0.2597	0.7481
AT4G31380	-4.1776	0.0000	-1.5227	0.0386	0.1021	0.9410	-1.1789	0.2220	0.3893	0.7842	-1.3550	0.0812	-3.7456	0.0000	-0.3431	0.8503	-2.5006	0.0002	-2.0641	0.0078	-0.9879	0.3065	-3.9269	0.0000	-0.1913	0.9109	0.5894	0.3983
AT4G31500	-0.8013	0.3111	-2.6247	0.0001	-0.8292	0.4089	-1.0334	0.3183	0.1912	0.9126	-1.9713	0.0114	-1.0208	0.1649	-0.7374	0.5747	-2.3755	0.0007	-0.7478	0.5541	0.1738	0.9125	-0.9051	0.2559	-1.1437	0.2264	-0.4835	0.5135
AT4G31870	-0.6411	0.3695	-2.4115	0.0001	-0.8973	0.2915	-0.8256	0.3806	-0.2367	0.8692	-2.0959	0.0022	0.2546	0.7471	-1.1602	0.1665	-2.1977	0.0005	-1.7330	0.0186	-0.6844	0.4721	-0.1559	0.8687	-2.0433	0.0020	-2.9610	0.0000
AT4G31877	-0.1059	0.9226	0.0962	0.9364	0.2344	0.8576	-0.3709	0.7823	-0.3097	0.8544	-0.2937	NA	-0.5918	0.4851	0.0727	0.9794	-0.3876	0.6784	-0.6180	0.6724	1.1474	0.2572	0.2653	0.7991	0.2674	0.8777	2.3544	0.0006
AT4G32280	-2.3948	0.0001	-0.1251	0.9039	0.3763	0.7219	-0.4016	0.7252	0.1931	0.9026	0.4410	0.6023	-2.4044	0.0001	0.4172	0.7912	-0.3410	0.6780	-1.1238	0.2255	-0.2231	0.8697	-2.0462	0.0015	0.7974	0.4303	-0.0842	0.9123
AT4G32340	0.6937	0.2698	1.5936	0.0062	1.5661	0.0134	1.1516	0.1325	1.4628	0.0220	1.4599	0.0203	1.2660	0.0228	1.1288	0.1227	1.8551	0.0010	1.6386	0.0123	-1.0610	0.1616	1.3077	0.0250	2.2745	0.0001	1.9047	0.0004
AT4G32510	0.7500	0.3758	2.2706	0.0020	1.8891	0.0240	1.6254	0.0938	0.0000	1.0000	1.4684	NA	1.0169	0.1862	1.0844	0.3349	0.8138	0.3336	1.1355	0.3039	0.1649	0.9208	0.4943	0.6045	1.2561	0.1964	1.8994	0.0072
AT4G32770	0.0186	0.9745	-0.7113	0.0884	-0.4312	0.4196	-0.7804	0.1205	-0.5765	0.2688	-1.2465	0.0021	0.2659	0.5410	-1.2208	0.0022	-1.2397	0.0009	-1.1690	0.0057	-0.7427	0.1269	0.0765	0.8934	-0.6577	0.1739	-2.2228	0.0000
AT4G32870	-2.0449	0.0000	-1.4687	0.0032	-1.4649	0.0065	-1.1092	0.0846	-1.9672	0.0001	-0.7555	0.1871	-2.1282	0.0000	-1.1213	0.0567	-0.7442	0.1598	-0.8688	0.2041	-1.3038	0.0239	-1.9723	0.0000	-2.5672	0.0000	-0.7682	0.1082
AT4G32980	0.1874	0.8095	-0.9355	0.1549	-0.6093	0.4623	-0.4172	0.6613	-0.1443	0.9184	-1.4158	0.0261	0.1526	0.8405	-0.1544	0.9294	-1.3755	0.0200	-0.3982	0.7398	0.4571	0.6269	0.8800	0.1549	-0.0809	0.9567	-2.1631	0.0001
AT4G33010	-0.0513	0.9477	-1.2959	0.0162	-0.3972	0.6155	-0.8898	0.2208	-0.4214	0.6557	-1.8478	0.0008	0.1868	0.7777	-0.7887	0.3029	-2.0428	0.0000	-1.4164	0.0184	0.1121	0.9217	0.0091	0.9926	-0.6956	0.3558	-2.4483	0.0000
AT4G33050	-2.1455	0.0001	-2.9748	0.0000	-1.5319	0.0188	-1.2476	0.1047	-1.0254	0.1787	-1.3792	0.0325	-2.3310	0.0000	-1.6203	0.0117	-2.2758	0.0001	-1.1006	0.1600	-0.3832	0.7064	-2.3317	0.0000	-1.8259	0.0026	-1.7150	0.0019
AT4G33150	-0.5262	0.5465	1.8792	0.0120	1.6462	0.0530	1.5901	0.0947	-0.0867	0.9678	2.1818	0.0055	-0.5444	0.5129	1.4317	0.1217	2.7707	0.0001	2.3272	0.0036	-0.2689	0.8531	0.1784	0.8696	0.6015	0.6486	2.5340	0.0002
AT4G33467	0.1250	0.9074	1.5849	0.0493	1.0310	0.3091	1.2098	0.2557	0.2861	0.8660	1.9438	0.0179	-0.0439	0.9670	0.9988	0.4028	2.1312	0.0045	1.7046	0.0668	-0.1475	0.9304	0.3393	0.7400	1.6020	0.0675	1.4334	0.0445
AT4G33490	0.2068	0.6610	1.3698	0.0001	1.4583	0.0001	0.9157	0.0529	2.0049	0.0000	1.2946	0.0011	0.6254	0.0942	1.7378	0.0000	1.4323	0.0001	1.1209	0.0073	-0.3858	0.5062	0.5343	0.1898	2.1898	0.0000	1.5511	0.0000
AT4G33550	1.5730	0.0378	0.5425	0.5857	0.0613	0.9692	-0.2382	0.8719	1.6534	0.0609	-0.7729	0.3987	1.5882	0.0290	0.1553	0.9505	-0.5054	0.5747	0.0938	0.9696	1.3437	0.1738	3.4455	0.0000	-0.2296	0.8996	-0.5671	0.4627
AT4G33610	1.4770	0.0173	-0.1127	0.9109	0.1547	0.8958	-0.0576	0.9675	0.0744	0.9690	0.4239	0.6026	0.1969	0.8088	1.1397	0.1686	0.2496	0.7604	1.4814	0.0509	2.0184	0.0028	1.0448	0.1152	0.6148	0.5661	2.2599	0.0001
AT4G33666	-2.3773	0.0000	-0.6210	0.3890	-1.1000	0.1360	-0.3993	0.6798	-1.8334	0.0037	-0.3752	0.6151	-2.0094	0.0002	-1.5293	0.0217	-0.2582	0.7247	-1.2443	0.0991	-1.3781	0.0556	-2.2381	0.0001	-0.7709	0.3709	-0.4637	0.4385
AT4G33980	0.7207	0.3912	1.3420	0.1026	1.6568	0.0559	1.0851	0.3094	2.4811	0.0009	1.8968	0.0190	1.8172	0.0102	2.1702	0.0066	2.4201	0.0009	3.2151	0.0000	0.0400	0.9847	1.7283	0.0207	2.1892	0.0047	2.8597	0.0000
AT4G34135	-1.8669	0.0000	-1.0699	0.0354	-0.4060	0.5708	-0.2042	0.8192	-0.3273	0.7222	-0.3991	0.5042	-2.1882	0.0000	-0.6386	0.4001	-0.7501	0.1421	-0.2892	0.7846	0.3455	0.6696	-1.8016	0.0001	-1.7635	0.0002	-1.3676	0.0026
AT4G34150	-0.7847	0.0519	-1.8939	0.0000	-1.2458	0.0029	-1.1317	0.0190	-1.5290	0.0001	-1.1821	0.0059	-1.8000	0.0000	-2.2086	0.0000	-2.0556	0.0000	-1.6613	0.0000	0.5091	0.3709	-1.7207	0.0000	-1.7966	0.0000	-1.3992	0.0002
AT4G34380	-2.7253	0.0000	-1.4800	0.0184	-1.0502	0.1636	-1.6768	0.0226	-0.8813	0.2825	-1.5319	0.0232	-3.2532	0.0000	-2.4796	0.0001	-1.9273	0.0018	-2.5587	0.0001	-1.8750	0.0062	-3.2581	0.0000	-1.1856	0.1042	-0.9223	0.1121
AT4G34390	-1.5605	0.0000	-1.6163	0.0000	-0.8924	0.0079	-1.1815	0.0006	-0.9511	0.0031	-1.0831	0.0010	-2.1312	0.0000	-1.4738	0.0000	-1.3380	0.0000	-1.4905	0.0000	-0.6143	0.1187	-2.0202	0.0000	-1.3981	0.0000	-1.3549	0.0000
AT4G34410	0.3356	0.7308	1.2904	0.1275	0.3034	0.8113	1.5104	0.1316	-0.5139	0.7200	0.5879	0.5334	-0.4219	0.6383	-0.0304	0.9924	0.6807	0.4293	-0.0922	0.9700	3.7968	0.0000	-0.4027	0.6843	0.1208	0.9484	0.5270	0.4957
AT4G34550	-1.8148	0.0158	-0.4778	0.6403	-1.2967	0.1788	-1.4848	0.1404	-1.7949	0.0380	-2.5279	0.0016	-1.9501	0.0066	-2.1208	0.0103	-2.4183	0.0011	-0.9603	0.4278	-0.9446	0.3871	-0.0571	0.9649	-1.1632	0.2531	-1.9614	0.0059
AT4G34610	-0.7257	0.1945	-0.8192	0.1723	-0.8714	0.1953	-1.1439	0.0938	-0.5654	0.4965	-2.3597	0.0000	-0.6954	0.1994	-0.9077	0.2012	-1.7466	0.0007	-0.5062	0.5963	0.3748	0.6716	-0.3236	0.6291	-0.7498	0.3045	-2.2356	0.0000
AT4G34630	-1.9010	0.0011	-1.1352	0.0856	-1.3365	0.0585	-1.4099	0.0681	-0.9101	0.2716	-1.8619	0.0039	-1.5555	0.0065	-1.3326	0.0638	-1.8242	0.0023	0.2167	0.8923	-0.7818	0.3638	-1.4347	0.0178	-1.0072	0.1958	-2.0560	0.0003
AT4G34881	-1.0566	0.1692	0.1162	0.9189	-0.0182	0.9905	-0.0732	0.9643	2.3214	0.0016	0.0983	0.9299	-0.6644	0.4034	0.2951	0.8810	-0.1179	0.9047	0.2891	0.8763	0.3699	0.7799	-0.3754	0.6957	0.1472	0.9352	2.4636	0.0003
AT4G34950	2.8643	0.0000	2.0256	0.0000	1.2494	0.0069	1.4485	0.0034	0.9433	0.0618	0.8742	0.0678	3.0422	0.0000	1.5581	0.0003	2.2207	0.0000	1.6714	0.0002	-0.5505	0.3743	2.6845	0.0000	-0.0953	0.9293	0.7471	0.0683
AT4G35110	-1.5932	0.0002	-1.3140	0.0040	-0.9977	0.0628	-0.9357	0.1198	-1.3908	0.0034	-0.9026	0.0769	-2.1907	0.0000	-1.6712	0.0003	-1.2658	0.0055	-0.5838	0.4190	-1.2691	0.0144	-1.7325	0.0001	-1.0609	0.0394	-1.3405	0.0019
AT4G35180	-0.8420	0.2958	-3.2530	0.0000	-2.4598	0.0012	-0.5025	0.6822	-1.3757	0.1362	-2.4048	0.0021	-1.4507	0.0430	-2.3543	0.0022	-3.2593	0.0000	-1.5682	0.0897	-1.1895	0.2288	-1.8505	0.0114	-2.7162	0.0002	-2.3469	0.0007
AT4G35720	-1.5376	0.0376	1.5369	0.0451	2.1084	0.0064	0.1312	0.9322	1.9348	0.0128	0.3662	0.7032	-1.3396	0.0622	1.4657	0.1013	0.0129	0.9917	-0.4482	0.7757	-0.3377	0.8057	-1.4662	0.0521	1.9714	0.0101	1.0259	0.1410
AT4G36010	0.5811	0.3260	0.7388	0.2395	0.8753	0.2031	0.9327	0.2057	1.3013	0.0302	0.3708	0.5871	0.7287	0.1837	0.6570	0.4573	0.4999	0.4130	0.4849	0.6303	0.9967	0.1505	0.8729	0.1234	1.3804	0.0170	2.0294	0.0000
AT4G36110	-1.9869	0.0001	-0.8258	0.1673	0.1948	0.8258	-0.9381	0.1899	0.0404	0.9811	-0.3211	0.6316	-2.0163	0.0000	-0.8444	0.2504	-1.4536	0.0061	-0.7426	0.3488	0.4332	0.6025	-1.9712	0.0001	-0.0448	0.9780	0.2923	0.5936
AT4G36600	0.1522	0.8859	3.3023	0.0000	0.6187	0.5797	0.0712	0.9665	0.5161	0.7174	-0.0491	NA	0.0215	0.9839	0.2516	0.9073	0.5482	0.5311	0.5474	0.7191	1.7475	0.0523	0.8732	0.3030	0.3694	0.8179	0.6462	0.3882
AT4G36610	2.2513	0.0011	0.3694	0.7227	0.9722	0.3259	0.8214	0.4609	1.2279	0.1993	1.1594	0.1669	1.0246	0.1656	1.1862	0.2413	0.6781	0.4181	1.9455	0.0191	1.5836	0.0752	1.9019	0.0074	1.1696	0.2243	1.4797	0.0319
AT4G36640	-1.5833	0.0105	-1.8078	0.0040	-1.2827	0.0891	-0.9244	0.3047	-0.7445	0.4344	-1.3644	0.0497	-1.8853	0.0013	-1.0843	0.2020	-2.1540	0.0004	0.1004	0.9605	0.0951	0.9468	-1.3903	0.0289	-1.4096	0.0488	-1.6737	0.0047
AT4G36700	1.2019	0.1280	1.4772	0.0718	0.0518	0.9741	0.0428	0.9815	-0.0495	0.9841	-0.2301	NA	2.0231	0.0044	-0.5997	0.7101	-0.0080	0.9952	-0.5550	0.7179	0.3396	0.8142	2.6819	0.0002	-0.6411	0.6314	-0.5759	0.4493
AT4G36830	1.2236	0.0609	0.1512	0.8832	-0.2309	0.8426	0.6888	0.4904	0.1525	0.9274	0.0226	0.9843	1.1451	0.0680	0.0085	0.9972	0.1010	0.9121	0.7808	0.4665	1.2672	0.1239	2.0723	0.0006	0.2158	0.8910	0.8766	0.1705
AT4G36880	-0.9797	0.1853	1.4518	0.0452	-0.4683	0.6607	0.0940	0.9503	0.5393	0.6667	-1.1408	0.1576	-0.1123	0.9021	0.2186	0.9134	-1.1450	0.1238	-0.2867	0.8689	2.2280	0.0023	-0.3431	0.7084	-0.3755	0.7971	-1.1864	0.0795
AT4G36990	-1.9487	0.0010	-2.1744	0.0002	-1.6934	0.0118	-1.1913	0.1463	-0.7781	0.3921	-1.4956	0.0261	-2.0735	0.0002	-1.6753	0.0129	-2.2074	0.0002	-0.7275	0.4730	-0.8714	0.3074	-1.8956	0.0014	-1.6502	0.0122	-1.5147	0.0089
AT4G37290	-1.8163	0.0156	-1.8595	0.0174	-1.2521	0.1982	0.6488	0.5900	-2.2295	0.0047	-0.8248	NA	-2.0000	0.0053	-1.6318	0.0739	-1.0058	0.2201	-1.4982	0.1288	-0.1593	0.9236	-1.4131	0.0727	-1.3789	0.1422	-1.3824	0.0539
AT4G37370	-1.7196	0.0024	-1.2361	0.0470	-0.6596	0.4209	0.7995	0.3454	-1.2823	0.0615	-0.1310	0.8791	-2.2639	0.0000	-1.4146	0.0344	-0.3403	0.6298	-0.8907	0.2944	-2.1526	0.0004	-2.2554	0.0000	-1.8926	0.0015	-0.6317	0.2710
AT4G37530	-1.1822	0.0002	-2.1377	0.0000	-1.4098	0.0000	-1.6330	0.0000	-1.5526	0.0000	-1.4937	0.0000	-1.7378	0.0000	-1.9435	0.0000	-1.5428	0.0000	-0.9623	0.0123	-1.3347	0.0001	-1.7417	0.0000	-2.2288	0.0000	-0.9502	0.0029
AT4G37560	-1.9900	0.0006	-1.7127	0.0053	-1.4899	0.0301	-1.8847	0.0085	-1.3406	0.0574	-1.2423	0.0659	-2.2930	0.0000	-1.4383	0.0366	-1.7625	0.0040	-0.9277	0.2772	-1.1389	0.1409	-1.7934	0.0022	-1.4306	0.0370	-2.0017	0.0008
AT4G37610	-1.4986	0.0083	-1.0688	0.0921	-1.0958	0.1237	-0.7041	0.4172	-2.2913	0.0000	-0.1809	0.8249	-2.5767	0.0000	-1.4194	0.0308	-0.5318	0.4181	-1.0585	0.1744	-0.5010	0.5831	-1.9061	0.0005	-1.1543	0.0935	-1.0551	0.0540
AT4G37670	-1.2891	0.0060	-1.6108	0.0006	-1.1177	0.0427	-0.5592	0.4383	-0.9601	0.0997	-0.5713	0.3124	-1.1327	0.0128	-0.4424	0.6330	-1.3767	0.0041	-0.4753	0.5765	-0.3130	0.7042	-0.9849	0.0441	-0.9106	0.1199	-2.0013	0.0000
AT4G37770	-1.8886	0.0024	-0.9218	0.2171	0.3890	0.7000	-1.2031	0.1671	0.1008	0.9541	-0.6134	0.4348	-2.3272	0.0001	0.0266	0.9924	-1.5276	0.0193	-1.9215	0.0073	-0.5710	0.5699	-2.1128	0.0006	0.2554	0.8586	-0.6638	0.2944
AT4G37925	-0.6068	0.4028	-1.6838	0.0098	-1.5122	0.0387	-1.5099	0.0637	-0.9821	0.2595	-2.1896	0.0012	-0.4538	0.5355	-1.1379	0.1814	-2.6655	0.0000	-0.5439	0.6551	-0.0999	0.9460	-0.5746	0.4489	-1.3412	0.0722	-3.3848	0.0000
AT4G37990	-2.1288	0.0033	-0.3199	0.7665	-0.2628	0.8369	0.3536	0.7924	-1.0531	0.3186	-0.2690	0.7940	-2.2095	0.0015	-1.3411	0.1744	0.6002	0.4861	0.1133	0.9629	-2.5456	0.0013	-1.6553	0.0285	-1.5831	0.0677	-0.4639	0.5501
AT4G38080	1.4828	0.0038	0.0238	0.9787	0.1521	0.8717	-0.2054	0.8360	0.3507	0.7333	0.7141	0.2485	0.1003	0.8854	0.8139	0.2866	0.1657	0.8122	1.5238	0.0104	1.9965	0.0002	0.9835	0.0728	0.9458	0.1543	2.2296	0.0000
AT4G38340	-0.3777	0.6947	-0.3833	0.7194	-0.2197	0.8698	0.6997	0.5592	0.7347	0.5610	-0.4928	0.6092	-0.3284	0.7245	0.6607	0.6648	-0.4797	0.5985	0.5758	0.7027	2.5671	0.0014	-0.1123	0.9240	0.7233	0.5714	1.4099	0.0490
AT4G38410	-1.8006	0.0092	0.3570	0.7060	0.4711	0.6502	-0.1996	0.8819	0.2858	0.8498	0.3621	0.6865	-2.0150	0.0020	0.0264	0.9925	0.4915	0.5372	-0.3333	0.8346	-1.8429	0.0251	-2.3731	0.0005	0.6462	0.5730	0.0416	0.9590
AT4G38552	-1.2854	0.0875	-2.1431	0.0036	-1.7492	0.0375	-2.3110	0.0082	-1.8019	0.0295	-1.6128	NA	-1.9246	0.0053	-1.7326	0.0412	-1.7181	0.0221	-2.4399	0.0025	-1.9847	0.0187	-1.7663	0.0161	-1.8830	0.0193	-1.9878	0.0044
AT4G38560	-2.3886	0.0003	-2.7961	0.0000	-1.9008	0.0126	-1.4769	0.1031	-0.5582	0.6557	-2.0812	0.0055	-2.6683	0.0000	-2.4349	0.0006	-2.8077	0.0000	-1.8562	0.0205	-1.1498	0.2154	-2.7892	0.0000	-2.0638	0.0045	-1.6095	0.0141
AT4G38580	-0.1932	0.7446	1.1777	0.0113	1.1451	0.0247	0.6961	0.2820	1.2206	0.0129	0.9572	0.0581	0.2981	0.5725	1.2063	0.0163	1.4399	0.0013	1.1450	0.0329	-0.9551	0.0953	0.2450	0.6819	1.9466	0.0000	2.2939	0.0000
AT4G38960	-2.0938	0.0000	-1.4871	0.0018	-1.5261	0.0026	-1.5064	0.0083	-1.7859	0.0002	-1.5763	0.0022	-2.2701	0.0000	-1.8321	0.0002	-1.6588	0.0004	-0.0858	0.9547	0.6616	0.3241	-1.9740	0.0000	-1.8216	0.0001	-1.7284	0.0001
AT4G39030	-1.8444	0.0037	-2.7077	0.0000	-1.5984	0.0304	-0.9661	0.3010	-1.1050	0.1958	-2.1825	0.0016	-1.6753	0.0066	-2.1906	0.0012	-2.8691	0.0000	-1.6155	0.0369	-1.1840	0.1642	-2.0393	0.0012	-2.3577	0.0003	-2.1452	0.0004
AT4G39090	1.0755	0.0589	1.5518	0.0059	1.5645	0.0102	1.5463	0.0239	1.5761	0.0080	1.6607	0.0059	1.3776	0.0097	1.2684	0.0543	1.9966	0.0002	1.5606	0.0144	-0.6763	0.4047	1.0473	0.0713	1.5672	0.0081	2.3359	0.0000

AT4G39610	-2.4322	0.0006	-2.0290	0.0068	-1.3980	0.1240	-1.0094	0.3409	-1.9580	0.0153	-0.2823	NA	-2.9683	0.0000	-1.7588	0.0385	-1.1216	0.1513	-1.5025	0.1133	-2.5537	0.0012	-2.3113	0.0012	-1.8444	0.0238	0.2701	0.7313
AT4G39670	-2.6442	0.0000	-2.2848	0.0001	-1.6831	0.0130	-0.5008	0.6135	-1.9852	0.0016	-1.2779	0.0622	-2.7330	0.0000	-2.7725	0.0000	-1.8810	0.0021	-1.9130	0.0051	-1.2070	0.1244	-2.9530	0.0000	-2.9569	0.0000	-1.5647	0.0072
AT4G39770	-1.3981	0.0001	-1.1269	0.0037	-1.1057	0.0090	-0.6156	0.2589	-0.2481	0.7416	-1.1541	0.0063	-2.2843	0.0000	-0.3283	0.6760	-1.2710	0.0009	-0.9206	0.0496	-0.3317	0.5885	-1.6124	0.0000	-0.6080	0.2380	-1.5142	0.0000
AT4G39795	0.5592	0.5198	0.0206	0.9875	0.2647	0.8342	-0.4623	0.7152	0.6235	0.6344	-0.6864	0.4590	0.3074	0.7341	0.3787	0.8420	-0.1672	0.8676	0.4764	0.7631	1.5366	0.0896	1.2799	0.0936	1.1123	0.2612	2.0652	0.0026
AT4G40020	-1.8071	0.0107	-1.5299	0.0456	-0.9692	0.3147	-1.1946	0.2314	-1.2691	0.1697	-0.7414	0.3895	-1.9623	0.0036	-1.2238	0.2085	-1.8582	0.0105	-1.4877	0.1050	-2.0906	0.0100	-1.5940	0.0282	-1.4087	0.1039	-1.8069	0.0082
AT4G40065	1.4406	0.0068	0.5296	0.4433	0.7125	0.3422	0.4008	0.6590	0.4277	0.6746	0.3839	0.5871	1.5312	0.0025	0.7081	0.4222	0.4962	0.4333	0.4074	0.7169	-0.8931	0.2574	1.5242	0.0041	0.1516	0.9132	2.1559	0.0000
AT5G01040	1.2024	0.0166	-0.0340	0.9685	1.3187	0.0188	-1.2767	0.0502	0.3780	0.6900	-0.9130	0.1217	1.4751	0.0018	-0.5817	0.5147	-2.1884	0.0000	-1.3866	0.0213	0.9780	0.1335	0.6122	0.2809	-1.5660	0.0045	-1.5990	0.0014
AT5G01100	-1.0528	0.0441	-2.3640	0.0000	-1.4199	0.0126	-1.0387	0.1299	-1.7628	0.0007	-1.1374	0.0478	-2.2490	0.0000	-2.1700	0.0000	-1.9762	0.0001	-1.5935	0.0057	-0.1708	0.8722	-2.2831	0.0000	-2.3836	0.0000	-1.9214	0.0001
AT5G01120	-0.1547	0.8689	-2.3312	0.0009	-2.0792	0.0074	0.2290	0.8568	-2.1058	0.0053	-0.2093	NA	-2.7650	0.0000	0.0357	0.9901	0.0992	0.9140	0.0321	0.9860	-0.4634	0.6940	0.0082	0.9951	0.0194	0.9955	-1.0643	0.1182
AT5G01250	-1.5130	0.0372	-1.3494	0.0887	-1.7994	0.0285	-1.0120	0.3311	-1.7358	0.0357	-0.5584	0.5348	-2.0686	0.0022	-1.9111	0.0174	-1.3406	0.0759	-0.4352	0.7804	-0.5591	0.6338	-1.4848	0.0443	-0.8369	0.4518	-0.5076	0.4917
AT5G01260	-1.5948	0.0011	-1.2752	0.0144	-1.4385	0.0090	-1.3153	0.0354	-1.1944	0.0389	-1.7240	0.0014	-1.8083	0.0001	-1.2304	0.0337	-1.4924	0.0030	-0.2905	0.7955	-0.5731	0.4425	-1.2138	0.0166	-0.9527	0.1245	-2.0144	0.0000
AT5G01330	-0.9407	0.2110	-1.1003	0.1786	0.1083	0.9359	0.0809	0.9574	-0.6783	0.5656	0.2583	NA	-2.0210	0.0025	-0.1635	0.9402	-0.3663	0.6706	-0.6700	0.6035	-0.2087	0.8869	-1.1104	0.1398	0.3982	0.7761	1.4721	0.0204
AT5G01380	-2.6827	0.0000	-1.4228	0.0072	-1.1301	0.0653	-1.3846	0.0301	-2.3095	0.0000	-1.0436	0.0731	-3.3508	0.0000	-2.1527	0.0000	-1.5677	0.0027	-1.7280	0.0027	-2.8389	0.0000	-3.2021	0.0000	-2.3797	0.0000	-1.7410	0.0005
AT5G01550	-1.9637	0.0007	-1.3550	0.0334	-0.9115	0.2462	-0.6627	0.4682	-1.5859	0.0169	-0.9633	0.1628	-2.5940	0.0000	-2.1604	0.0005	-1.1685	0.0621	-1.0137	0.2253	-2.2122	0.0006	-1.4996	0.0124	-1.6099	0.0143	-0.6900	0.2421
AT5G01590	0.4287	0.3258	-0.0464	0.9415	-0.3486	0.5504	-0.1100	0.8883	-0.2174	0.7896	-0.6287	0.1642	0.3423	0.4329	0.1744	0.8687	-0.6788	0.1023	-0.7474	0.1531	-0.2876	0.6636	0.2992	0.5360	-0.2695	0.7221	-2.0698	0.0000
AT5G01600	-2.1670	0.0001	0.2985	0.7168	0.5753	0.4979	0.3582	0.7212	0.2146	0.8717	0.7270	0.2921	-2.4610	0.0000	0.4573	0.7170	0.6450	0.3211	-0.2612	0.8514	-0.3600	0.7255	-2.2282	0.0000	0.4307	0.6962	0.8066	0.1536
AT5G01610	-1.1527	0.0027	-1.7295	0.0000	-1.8358	0.0000	-0.8833	0.0828	-1.0318	0.0185	-0.2493	0.6187	-2.4205	0.0000	-1.0532	0.0162	-0.6556	0.1166	-0.7129	0.1838	-1.0945	0.0163	-1.0900	0.0049	-0.8925	0.0503	-0.6847	0.0723
AT5G01740	-1.6665	0.0133	-1.5498	0.0306	-0.9985	0.2660	-1.6327	0.0592	-0.6453	0.5738	-2.3220	0.0014	-2.2478	0.0003	-1.1806	0.1987	-2.0874	0.0020	-0.4755	0.7331	-0.0749	0.9630	-1.5607	0.0229	-0.9022	0.3583	-0.4835	0.4879
AT5G01900	-1.9223	0.0102	-2.6122	0.0003	-1.4234	0.1298	-1.8896	0.0472	-1.0750	0.3170	-2.5477	0.0015	-1.9394	0.0071	-1.9509	0.0216	-2.4738	0.0009	-1.1658	0.2912	-0.0266	0.9907	-1.9962	0.0078	-2.4359	0.0016	-0.8360	0.2623
AT5G02020	-3.2868	0.0000	0.5771	0.4902	0.1413	0.9083	0.4820	0.6573	-1.0490	0.2361	1.2216	0.0995	-2.9706	0.0000	-0.3439	0.8390	1.2031	0.0804	0.4049	0.7763	-1.6369	0.0343	-2.9781	0.0000	0.3920	0.7682	0.6366	0.3304
AT5G02170	-0.9537	0.1663	-1.6738	0.0138	-1.2563	0.1200	0.3512	0.7569	-2.6589	0.0000	0.4621	0.5711	-3.0491	0.0000	0.1697	0.9312	0.9996	0.1437	0.4204	0.7603	-0.7358	0.4462	-1.0497	0.1299	0.4583	0.7099	0.9390	0.1300
AT5G02200	-1.0720	0.0685	-1.8712	0.0010	-1.1613	0.0961	-1.0497	0.1807	-0.8202	0.3123	0.0298	0.9751	-2.7495	0.0000	0.4630	0.7006	-1.0264	0.0896	-1.0876	0.1548	-0.0846	0.9485	-1.5460	0.0065	0.3055	0.8003	0.7027	0.2080
AT5G02230	-0.3213	0.6938	-0.2478	0.7931	-0.5292	0.5811	-0.5704	0.5784	-0.4897	0.6801	-2.2794	0.0009	-0.4771	0.5137	-1.4039	0.0694	-1.8616	0.0038	-0.2649	0.8702	0.5259	0.6118	-0.1029	0.9178	-0.5402	0.6389	-0.1246	0.8642
AT5G02270	-1.2129	0.0293	-1.5200	0.0069	-1.2312	0.0582	-1.0908	0.1393	-1.1000	0.1061	-1.5371	0.0110	-1.6120	0.0019	-1.0221	0.1588	-1.8510	0.0007	-0.7588	0.3746	-0.7977	0.3006	-1.0405	0.0711	-1.1233	0.0885	-2.1352	0.0000
AT5G02430	-0.3454	0.6468	-1.3137	0.0441	-0.6340	0.4691	-0.6323	0.5008	-0.3596	0.7676	-1.7870	0.0078	-0.5747	0.3846	-0.3373	0.8260	-2.0699	0.0007	-1.3465	0.0776	-0.7273	0.4142	-0.4062	0.5949	-0.6084	0.5482	-1.7570	0.0026
AT5G02440	-1.0825	0.0131	-1.8933	0.0000	-1.7909	0.0001	-0.1083	0.9013	-1.5015	0.0009	-0.1871	0.7536	-2.6890	0.0000	-0.4659	0.5525	0.1061	0.8563	-0.2598	0.7870	0.1188	0.9004	-1.1482	0.0086	-0.6512	0.2822	-0.4015	0.3641
AT5G02490	-2.4368	0.0002	-2.7932	0.0000	-1.8886	0.0133	-0.8194	0.4362	-1.0623	0.2632	-2.5581	0.0005	-2.4295	0.0001	-2.0706	0.0053	-3.6363	0.0000	-1.2460	0.1896	-0.8547	0.3950	-2.6793	0.0000	-2.0824	0.0040	-2.4090	0.0002
AT5G02540	-3.3525	0.0000	-1.1411	0.0729	0.4894	0.5744	-1.2929	0.0888	0.1879	0.8910	-1.3414	0.0372	-2.9159	0.0000	-0.3477	0.8077	-2.2892	0.0001	-2.7170	0.0000	-2.7673	0.0000	-3.3237	0.0000	0.1628	0.9099	-1.1833	0.0323
AT5G02580	-3.0448	0.0000	0.2903	0.7645	0.9956	0.2598	-0.1579	0.9076	1.7320	0.0189	0.8473	0.2832	-2.8966	0.0000	1.1109	0.2317	0.0957	0.9170	0.0119	0.9960	-0.8964	0.3492	-2.8983	0.0000	0.9472	0.3141	1.2579	0.0476
AT5G02760	-1.8390	0.0007	-0.6415	0.3531	0.2307	0.8090	-0.6224	0.4759	0.4324	0.6798	-0.6991	0.2961	-2.0658	0.0001	0.6129	0.5423	-1.1715	0.0453	-2.0393	0.0007	-0.4872	0.5885	-1.7020	0.0020	0.5611	0.5534	-1.2308	0.0215
AT5G02780	-2.9040	0.0000	-1.9924	0.0025	-1.7314	0.0187	-0.2960	0.8042	-1.7920	0.0122	-1.1199	0.1354	-2.3461	0.0001	-2.3387	0.0006	-2.0563	0.0018	-1.4951	0.0658	-2.7629	0.0001	-2.7979	0.0000	-2.5087	0.0002	-1.3516	0.0310
AT5G02830	-1.4704	0.0055	-1.2309	0.0305	-1.0470	0.1140	-1.1163	0.1176	-1.2338	0.0491	-1.5040	0.0104	-1.2053	0.0205	-1.1631	0.0745	-1.5778	0.0033	-0.3162	0.7909	0.2189	0.8360	-0.9252	0.1039	-1.1116	0.0813	-2.3395	0.0000
AT5G02865	-2.0333	0.0012	-0.6038	0.4503	-0.0194	0.9887	-0.7288	0.4531	0.1280	0.9367	-0.6941	0.3676	-1.6748	0.0057	-0.4162	0.7780	-1.4262	0.0330	-1.3765	0.0922	0.2586	0.8347	-1.3016	0.0484	-0.0784	0.9635	-1.2372	0.0459
AT5G03240	0.0516	0.9374	0.8978	0.0620	0.9037	0.0927	0.8674	0.1452	0.6497	0.2972	0.8073	0.1084	0.2748	0.5989	0.6965	0.2756	1.2390	0.0053	1.1704	0.0238	0.4224	0.5398	0.3785	0.4794	1.3510	0.0035	2.0285	0.0000
AT5G03350	-0.2766	0.7765	-2.2931	0.0015	-1.2329	0.1888	-1.0476	0.3241	-0.1931	0.9142	-2.3401	0.0027	-0.4729	0.5802	-1.0771	0.3226	-2.2828	0.0016	-1.3726	0.1605	0.2341	0.8780	-0.3121	0.7556	-0.8156	0.4807	-0.2944	0.7121
AT5G03355	0.1687	0.8102	-2.0110	0.0001	-0.3675	0.6470	-1.1652	0.0878	-1.0775	0.0908	-0.8888	0.1388	-0.8541	0.1039	-1.0269	0.1237	-1.6977	0.0011	-1.4176	0.0199	0.3788	0.6662	-0.4652	0.4559	-1.2900	0.0277	-0.5607	0.2799
AT5G03545	1.6997	0.0047	0.4013	0.6363	1.3931	0.0537	0.6411	0.5065	2.5814	0.0000	0.3363	0.6876	0.7805	0.2247	1.2207	0.1187	0.1347	0.8757	1.1094	0.1921	1.8902	0.0052	1.5618	0.0109	0.6352	0.5424	0.7219	0.2438
AT5G03860	-0.0538	0.9618	2.8154	0.0001	1.1090	0.2501	0.8806	0.4283	0.0285	0.9921	0.3733	0.7023	-0.1676	0.8631	0.4086	0.8262	1.2689	0.1009	0.8217	0.5108	2.1468	0.0086	-0.1155	0.9198	0.4518	0.7587	0.6839	0.3520
AT5G04130	0.0839	0.8953	-0.5576	0.3031	-0.7439	0.2000	-0.3797	0.6006	-0.5343	0.4368	-0.8532	0.0936	0.1080	0.8552	-0.3557	0.7118	-1.0361	0.0252	-0.7069	0.2777	0.0504	0.9627	0.4316	0.4135	-0.4864	0.5020	-2.0102	0.0000
AT5G04140	-0.6066	0.1476	-0.7553	0.0826	-0.2554	0.6801	-0.5670	0.3255	-0.5291	0.3610	-0.9990	0.0215	-0.4701	0.2620	-0.6598	0.2315	-0.9846	0.0145	-1.0418	0.0244	-0.4659	0.4242	-0.7802	0.0578	-1.1684	0.0050	-2.3464	0.0000
AT5G04150	2.8729	0.0000	-1.0406	0.2028	-0.4418	0.6823	0.5503	0.6067	-1.5189	0.0760	-0.4616	0.6036	2.4480	0.0000	-0.1222	0.9584	0.1487	0.8715	-0.0624	0.9778	-0.7999	0.4402	3.9989	0.0000	-0.2426	0.8796	-0.9535	0.1683
AT5G04190	-2.6189	0.0000	-1.3870	0.0047	-0.3194	0.6743	-0.6153	0.3989	-0.3699	0.6826	-0.4674	0.4354	-2.0101	0.0000	-0.5060	0.5694	-1.5066	0.0018	-2.1634	0.0000	-0.7265	0.2809	-2.2637	0.0000	-0.0205	0.9907	-0.6954	0.1404
AT5G04200	-0.0065	0.9949	-1.2364	0.0451	-0.4782	0.5758	-0.1567	0.8905	0.5159	0.5977	-1.7466	0.0061	0.4689	0.4590	-0.2560	0.8669	-0.9006	0.1421	-0.4032	0.7316	-2.1394	0.0006	0.2733	0.7189	-0.5431	0.5778	-0.6096	0.2849
AT5G04250	-0.0538	0.9389	1.3145	0.0052	0.9812	0.0806	0.8970	0.1549	0.6561	0.3305	1.2517	0.0142	-0.0697	0.9122	0.9960	0.0803	2.3448	0.0000	1.5686	0.0019	-0.1969	0.8348	0.2549	0.6807	1.1480	0.0275	1.1543	0.0095
AT5G04390	-1.3620	0.0335	-1.2797	0.0661	-1.0071	0.2233	-1.0050	0.2591	-1.0010	0.2459	-0.6654	0.3857	-1.4524	0.0159	-0.7893	0.4405	-1.2280	0.0677	-0.8159	0.4157	-0.5636	0.5686	-2.0316	0.0012	-1.1347	0.1642	-1.0127	0.1032
AT5G04970	-1.3475	0.0694	-1.4713	0.0627	-0.5818	0.5879	-0.9120	0.3930	-0.6853	0.5733	-1.1523	NA	-2.0995	0.0021	-0.3477	0.8524	-2.1724	0.0030	-1.0688	0.3235	-0.9573	0.3554	-2.1061	0.0033	-1.0323	0.3127	-1.4933	0.0321
AT5G05250	2.3160	0.0004	-0.5590	0.5282	-0.9052	0.3337	0.9894	0.3150	-0.3293	0.8237	-0.8824	0.2764	2.4889	0.0001	0.2292	0.9078	-0.4580	0.5765	0.2531	0.8888	0.3635	0.7693	3.3035	0.0000	-1.0535	0.2574	-1.5085	0.0217
AT5G05290	-0.0359	0.9741	2.1392	0.0031	0.3161	0.7901	0.5725	0.6251	0.3464	0.8219	0.7355	NA	-0.1308	0.8907	0.4722	0.7783	0.5054	0.5503	0.5820	0.6764	0.9999	0.3240	-0.1274	0.9080	0.9252	0.3823	0.5882	0.4153
AT5G05300	-0.9578	0.1872	-2.0053	0.0037	-1.0907	0.2185	-0.4673	0.6807	-0.5595	0.6489	-0.4599	0.5958	-1.3052	0.0508	-1.5968	0.0466	-1.3688	0.0550	-1.2484	0.1794	-0.6455	0.5413	-1.6350	0.0176	-1.7893	0.0176	-0.6731	0.3222
AT5G05580	-0.0833	0.9191	-1.5399	0.0075	-1.2519	0.0603	-0.9251	0.2465	-1.1740	0.0862	-2.0984	0.0004	0.1179	0.8743	-1.0800	0.1388	-2.3232	0.0000	-1.7288	0.0062	-0.2606	0.8081	-0.0611	0.9479	-1.1215	0.0998	-3.3953	0.0000
AT5G06520	-0.3642	0.7058	-0.0404	0.9748	0.1548	0.9125	-0.3388	0.8075	0.1373	0.9453	-0.7423	NA	0.1764	0.8595	0.4927	0.7797	0.4293	0.6420	0.6873	0.6283	2.8125	0.0003	-0.7792	0.3718	-0.5408	0.7041	-0.0742	0.9327
AT5G06570	-2.2893	0.0000	0.1909	0.7855	0.8836	0.1366	-0.0476	0.9651	0.7159	0.2770	1.0779	0.0406	-1.5231	0.0007	0.0263	0.9901	0.3042	0.5984	-0.0399	0.9788	-1.8697	0.0004	-2.8518	0.0000	-0.6837	0.3231	0.7009	0.1292
AT5G06690	0.8498	0.2051	1.5876	0.0136	1.5025	0.0351	1.1679	0.1700	1.4797	0.0385	2.0939	0.0016	0.8488	0.1891	1.3700	0.0675	1.9759	0.0013	1.5590	0.0359	-1.7860	0.0116	0.9072	0.1810	1.8070	0.0060	1.6542	0.0050
AT5G06730	-0.1642	0.8627	-1.4294	0.0601	-2.2349	0.0033	-1.8228	0.0366	-0.7309	0.5104	-1.1634	NA	-1.0465	0.1298	-1.5570	0.0622	-2.1780	0.0022	-0.7513	0.5296	-1.1333	0.2307	-1.1645	0.1115	-2.2863	0.0021	-1.3048	0.0535
AT5G06760	0.3221	0.7439	2.3630	0.0015	0.5471	0.6360	0.8802	0.4457	0.2386	0.8931	0.6062	0.5217	-0.2931	0.7581	0.3460	0.8634	1.8023	0.0195	1.2910	0.2209	1.2970	0.1974	2.6001	0.0003	1.2154	0.2227	0.6943	0.3594
AT5G06845	-0.2475	0.8033	-2.1002	0.0049	-2.4404	0.0016	-0.5739	0.6345	-1.9160	0.0192	0.0567	0.9621	-2.0925	0.0027	-0.8361	0.5176	-0.0344	0.9763	0.0730	0.9761	0.2277	0.8838	0.4980	0.5928	-0.1171	0.9490	-0.2233	0.7842
AT5G07000	-2.0616	0.0000	-0.9921	0.0731	0.2574	0.7555	-0.7828	0.2790	-0.1769	0.8798	-0.2824	0.6701	-2.3627	0.0000	-0.2023	0.8848	-1.5369	0.0023	-1.0573	0.1040	0.0725	0.9506	-2.0624	0.0000	0.2699	0.8018	-0.7004	0.1546
AT5G07010	-5.5131	0.0000	-0.5549	0.4207	0.6209	0.4273	-1.1921	0.1002	0.7867	0.3157	-0.4784	0.4876	-4.6368	0.0000	0.0131	0.9954	-0.8498	0.1498	-1.3796	0.0361	-2.2323	0.0001	-4.6704	0.0000	0.9713	0.1654	1.1235	0.0318
AT5G07100	-1.8870	0.0001	-0.9976	0.0725	-0.1409	0.8772	-0.7779	0.2854	-0.4585	0.6032	0.6124	0.3095	-2.3261	0.0000	-0.7348	0.3384	-0.2821	0.6518	-0.6938	0.3792	-1.8034	0.0008	-2.0939	0.0000	-0.4777	0.5828	0.0025	0.9972
AT5G07200	-1.1769	0.1096	-0.3824	0.6952	0.4224	0.7020	-1.5321	0.0991	0.3980	0.7806	-0.7424	0.3854	-0.5895	0.4509	0.8003	0.5076	-1.2420	0.0972	-1.7687	0.0377	0.0723	0.9661	-1.4021	0.0555	0.2165	0.8980	-2.0050	0.0032
AT5G07330	0.1634	0.8508	2.3534	0.0001	0.0840	0.9453	0.0000	1.0000	0.2560	0.8524	0.1012	NA	0.0000	1.0000	0.0000	1.0000	0.2584	0.7416	0.1507	0.9334	1.0042	0.2334	1.1869	0.0695	0.1002	0.9478	0.0000	1.0000
AT5G07640	-0.5967	0.3485	-1.4532	0.0204	-0.3739	0.6756	-0.6422	0.4622	-0.4745	0.6475	-1.5129	0.0254	-0.4100	0.5190	-0.8836	0.2934	-2.0869	0.0008	-0.9673	0.2436	-0.2269	0.8402	-0.5441	0.4150	-0.3393	0.7732	-1.7487	0.0033
AT5G07700	0.7954	0.3254	-2.1130	0.0048	-1.8700	0.0256	-0.2582	0.8554	-1.2873	0.1824	-2.1142	0.0092	0.0792	0.9369	-0.9467	0.4265	-1.4492	0.0605	-1.1473	0.2850	0.1619	0.9207	1.1453	0.1418	-1.1253	0.2610	-1.4896	0.0345
AT5G07760	-2.1463	0.0001	-2.8991	0.0000	-1.9801	0.0011	-1.9717	0.0032	-0.5911	0.5153	-1.9628	0.0019	-1.4923	0.0052	-0.9839	0.1997	-2.5777	0.0000	-1.1288	0.1292	-0.5864	0.4933	-1.6581	0.0031	-1.1242	0.1057	-1.2442	0.0239
AT5G07780	-0.3268	0.7036	-1.7548	0.0157	-1.0557	0.2440	-1.6436	0.0635	-0.4957	0.6989	-1.4235	NA	-0.6465	0.3767	-1.4216	0.0970	-2.0020	0.0048	-1.2394	0.1921	-0.6308	0.5546	-0.5734	0.4806	-0.8991	0.3758	-0.2449	0.7430
AT5G08240	-2.0161	0.0001	-1.9502	0.0003	-1.2311	0.0570	-1.2290	0.0864	-0.5719	0.5200	-0.6478	0.3228	-2.3278	0.0000	-1.6871	0.0042	-1.7822	0.0011	-1.2659	0.0623	-1.1543	0.0937	-2.5042	0.0000	-1.2453	0.0484	0.2380	0.6890
AT5G08650	-0.4293	0.2940	-0.6698	0.1052	-0.3688	0.4954	-0.6431	0.2138	-0.2880	0.6746	-0.9398	0.0211	-0.3351	0.4144	-0.4187	0.5206	-1.1069	0.0027	-0.8324	0.0715	-0.3724	0.5129	-0.3374	0.4460	-0.6856	0.1370	-2.0735	0.0000
AT5G08720	-0.5785	0.0719	-0.5642	0.1052	-0.6680	0.0735	-0.6502	0.1168	-1.0364	0.0012	-0.9017	0.0083	-0.7553	0.0116	-1.1636	0.0003	-0.9561	0.0021	-0.7906	0.0335	-0.6442	0.1026	-0.5924	0.0689	-0.7229	0.0424	-2.0726	0.0000
AT5G08760	-2.4900	0.0001	-3.3907	0.0000	-2.4436	0.0004	-2.4636	0.0014	-0.5369	0.6672	-2.6567	0.0002	-2.7931	0.0000	-1.7114	0.0266	-3.7720	0.0000	-2.2169	0.0030	-0.4792	0.6804	-2.5819	0.0001	-2.1444	0.0023	-2.1240	0.0009
AT5G08790	-2.3389	0.0000	-2.1111	0.0000	-1.5348	0.0101	-0.1629	0.8798	-1.1752	0.0704	-1.0747	0.0792	-2.3842	0.0000	-1.2444	0.0537	-1.3823	0.0123	-1.1875	0.0827	-0.5879	0.4723	-2.2864	0.0000	-1.8232	0.0011	-1.6917	0.0010
AT5G09215	-0.2571	0.7984	2.1291	0.0037	1.9988	0.0134	-0.2174	0.8831	3.0436	0.0000	-0.3014	0.7701	2.5462	0.0001	-0.2397	0.9134	-0.0166	0.9895	-0.1201	0.9608	0.0414	0.9842	-0.2229	0.8380	-0.0678	0.9766	-0.5654	0.4643
AT5G09290	-1.6496	0.0217	-2.0082	0.0061	-1.8143	0.0259	-1.5337	0.1042	-0.0901	0.9653	-1.8746	0.0178	-1.2918	0.0684	-1.7780	0.0307	-2.7673	0.0001	-1.4818	0.1098	-0.9519	0.3527	-1.6474	0.0236	-1.9840	0.0106	-0.9421	0.1784
AT5G09440	-0.5579	0.3542	0.0184	0.9845	0.7399	0.3060	0.0320	0.9804	-0.1610	0.8989	0.2350	0.7472	-0.8548	0.1151	0.3730	0.7610	0.9902	0.0780	0.8958	0.2355	0.1719	0.8780	-0.7834	0.1792	0.0188	0.9933	2.1042	0.0000
AT5G09805	-2.0385	0.0023	-2.2003	0.0013	-1.4566	0.0744	-1.4537	0.1043	-0.7347	0.4958	-1.9929	0.0083	-2.5342	0.0001	-1.5832	0.0482	-1.9746	0.0043	-1.5910	0.0579	-0.6830	0.5098	-1.6746	0.0145	-1.1195	0.2105	-0.8640	0.1955
AT5G09815	0.0000	1.0000	2.8447	0.0001	3.0411	0.0000	0.0000	1.0000	3.7451	0.0000	0.0000	NA	2.6420	0.0001	0.0000	1.0000	0.0000	1.0000	0.0000	1.0000	0.0000	1.0000	0.0000	1.0000	0.2053	0.9078	0.0000	1.0000
AT5G09876	-1.8011	0.0168	-1.6620	0.0388	-1.2009	0.2211	-1.6704	0.0902	-0.6516	0.6266	-0.7143	NA	-1.9995	0.0054	-2.0697	0.0129	-1.9106	0.0125	-1.9619	0.0268	-1.5702	0.0972	-1.8688	0.0137	-1.6192	0.0655	-1.9452	0.0063
AT5G09990	-1.2798	0.0608	-1.3346	0.0674	-1.5173	0.0542	-0.9060	0.3526	-0.0636	0.9756	-1.5639	0.0363	-1.3930	0.0309	-0.5508	0.6895	-2.1218	0.0017	-0.2320	0.8964	-0.4562	0.6896	-0.9423	0.1931	-0.9666	0.2997	-1.8815	0.0036
AT5G10100	-0.9277	0.2290	-0.7907	0.3679	-0.2176	0.8613	-1.3965	0.1459	0.2859	0.8578	-2.0564	0.0090	-1.2135	0.0891	0.1304	0.9577	-1.7527	0.0170	-0.7390	0.5597	1.2052	0.2039	-1.0495	0.1758	0.0431	0.9860	-0.8379	0.2342
AT5G10250	-0.3991	0.5420	-1.1552	0.0528	-0.7397	0.3230	-1.1507	0.1150	-1.2649	0.0507	-0.7389	0.2541	-0.5160	0.3881	-1.3154	0.0405	-2.1519	0.0001	-1.4195	0.0306	0.2282	0.8315	-0.5547	0.3841	-0.8728	0.2365	-2.4115	0.0000
AT5G10380	-1.7034	0.0028	-2.2270	0.0001	-1.3985	0.0372	-1.7579	0.0123	-0.1039	0.9470	-1.3864	0.0315	-1.6012	0.0038	-2.1802	0.0002	-2.6169	0.0000	-1.8390	0.0045	-0.6982	0.4159	-1.9086	0.0007	-1.7011	0.0059	-1.1758	0.0342
AT5G10470	-0.6553	0.1877	-1.2066	0.0118	-0.8420	0.1508	-0.8599	0.1771	-0.9242	0.1073	-1.7088	0.0005	-0.7147	0.1308	-0.9718	0.0903	-1.9045	0.0000	-0.5866	0.4340	0.0834	0.9379	-0.4800	0.3744	-0.6353	0.3407	-2.1725	0.0000
AT5G10760	-0.7150	0.3997	-1.9946	0.0088	-0.7328	0.4997	-1.3809	0.1750	0.8650	0.4517	-1.2712	0.1352	-0.8751	0.2674	-1.0098	0.3890	-2.0728	0.0055	-0.3277	0.8561	-0.1555	0.9253	-0.8640	0.3068	-0.6984	0.5838	-0.2126	0.7969
AT5G10946	-2.2075	0.0000	-0.4904	0.4144	-0.6383	0.3314	-0.7703	0.2596	-0.8874	0.1504	-0.0050	0.9959	-2.2220	0.0000	-0.9029	0.1518	-0.1755	0.7780	0.2078	0.8595	-0.2390	0.7894	-1.8031	0.0001	-0.3256	0.7286	0.7615	0.0983
AT5G11210	-2.4747	0.0001	-2.5653	0.0001	-1.7184	0.0203	-2.3286	0.0022	-1.8366	0.0100	-1.8174	0.0122	-2.7498	0.0000	-3.1303	0.0000	-2.0408	0.0020	-1.6313	0.0389	-2.0905	0.0043	-2.6814	0.0000	-2.1133	0.0022	-0.9799	0.1240
AT5G11260	-2.0907	0.0001	-1.4428	0.0122	-1.5682	0.0107	-1.4098	0.0448	-1.6768	0.0044	-1.0893	0.0834	-2.3574	0.0000	-1.8483	0.0016	-1.6626	0.0029	0.2143	0.8826	-0.7332	0.3588	-1.6156	0.0031	-1.8698	0.0011	-1.5979	0.0026
AT5G11590	1.5072	0.0230	1.5534	0.0262	1.2777	0.1220	0.4335	0.7051	-0.0339	0.9895	0.1884	0.8513	1.6053	0.0108	1.2893	0.1302	0.4402	0.5915	1.5130	0.0656	1.9906	0.0066	2.0224	0.0015	0.8253	0.4222	0.8247	0.2171
AT5G11920	-0.9074	0.2462	-2.1688	0.0026	-0.3621	0.7584	-1.4511	0.1306	0.7401	0.5276	-2.2131	0.0046	-0.5912	0.4657	-1.2938	0.1796	-2.6162	0.0002	-0.4070	0.8004	0.0380	0.9848	-1.3006	0.0857	-1.4880	0.0823	-1.6212	0.0180
AT5G12030	-2.3165	0.0015	-0.8260	0.3707	-1.5911	0.0765	1.0934	0.3098	-0.9674	0.3855	-1.3395	0.1169	-1.9768	0.0056	-1.9527	0.0206	-0.3191	0.7374	0.1470	0.9503	-1.1405	0.2695	-1.5711	0.0419	-1.4621	0.1096	-1.2794	0.0755
AT5G12340	-1.7317	0.0073	-2.5366	0.0001	-1.7393	0.0181	-0.3787	0.7341	-1.4157	0.0698	-0.8204	0.2875	-2.4889	0.0000	-1.8557	0.0100	-0.8950	0.2041	-1.1449	0.2037	-1.1924	0.1647	-2.4513	0.0001	-2.5556	0.0001	-0.6806	0.2929
AT5G12960	-0.5411	0.5417	0.0009	1.0000	-0.2412	0.8513	-0.7425	0.5208	2.1144	0.0081	-0.3980	NA	-0.3744	0.6789	-0.2323	0.9159	-0.6199	0.4640	-0.6846	0.6190	-0.7816	0.4778	-0.4454	0.6409	-0.4215	0.7802	-0.5449	0.4655
AT5G13080	-2.6824	0.0001	-1.8788	0.0107	-2.0014	0.0111	-0.3151	0.8121	-1.2877	0.1618	-0.2886	0.7677	-2.6491	0.0001	-1.7984	0.0271	-1.0306	0.1804	-1.1641	0.2547	-2.5602	0.0008	-2.0217	0.0041	-1.9311	0.0126	-0.9220	0.1869
AT5G13320	-2.8948	0.0000	-2.9951	0.0000	-1.9729	0.0100	-1.2613	0.1897	-1.4493	0.0868	-2.3590	0.0016	-2.0718	0.0016	-2.7140	0.0001	-3.2115	0.0000	-2.0003	0.0122	-1.7211	0.0374	-2.5710	0.0001	-2.5543	0.0003	-2.4555	0.0002
AT5G13770	-1.1534	0.0105	-0.2757	0.6582	-0.2978	0.6748	-0.8631	0.1629	-1.1997	0.0164	-0.7428	0.1553	-1.1389	0.0088	-1.2796	0.0103	-0.9859	0.0359	-0.9274	0.1161	-0.4424	0.5296	-1.3336	0.0027	-0.6818	0.2727	-2.0404	0.0000
AT5G14180	-1.9150	0.0031	0.0151	0.9898	0.0573	0.9666	1.0807	0.2408	-0.9412	0.3154	0.6379	0.4277	-2.1444	0.0005	-0.4913	0.7334	1.0105	0.1483	1.1266	0.2162	-1.0637	0.2350	-2.2084	0.0005	-0.4648	0.7122	1.1532	0.0651
AT5G14200	1.5535	0.0319	-2.0303	0.0051	-1.1657	0.2105	0.4812	0.6914	0.0887	0.9664	-1.5779	0.0481	1.2249	0.0886	0.2118	0.9215	-1.3573	0.0709	-0.5755	0.6829	0.0793	0.9634	1.6309	0.0253	-0.4978	0.7178	-1.7294	0.0110
AT5G14400	-2.3150	0.0006	-0.8238	0.3152	-0.2521	0.8257	-0.6287	0.5528	-0.4783	0.7079	0.1303	0.8962	-2.2289	0.0005	-0.1936	0.9218	-0.4052	0.6204	-0.6161	0.6261	-0.5398	0.6243	-1.8133	0.0085	-0.0623	0.9755	0.3917	0.5751
AT5G14760	-2.1073	0.0012	-2.2143	0.0007	-1.7888	0.0163	-0.6451	0.5441	-1.1032	0.2162	-2.2124	0.0020	-1.9925	0.0015	-1.1072	0.2363	-2.2601	0.0006	-0.2504	0.8874	0.4774	0.6745	-2.0245	0.0019	-2.2339	0.0011	-2.4365	0.0001
AT5G14820	0.2674	0.7911	0.4289	0.6806	-0.3070	0.8099	0.2207	0.8827	2.5969	0.0006	-0.1933	NA	0.0927	0.9279	0.3229	0.8750	0.3514	0.7114	0.2972	0.8787	-0.0050	1.0000	0.0490	0.9699	0.0079	0.9995	-0.2914	0.7231
AT5G15100	-0.1530	0.8697	-0.3312	0.7194	-0.2161	0.8485	0.1269	0.9278	0.0228	0.9930	-0.4469	NA	-0.2426	0.7731	-0.0338	0.9909	-0.0142	0.9897	-0.3523	0.8126	-0.2085	0.8782	0.0114	0.9927	-0.4513	0.7143	2.0637	0.0010
AT5G15265	-2.6090	0.0000	0.5179	0.5397	0.2095	0.8537	-0.2420	0.8454	0.2321	0.8765	0.1353	0.8900	-1.5492	0.0141	-0.0401	0.9891	0.4152	0.5972	0.3138	0.8372	-1.3928	0.0940	-1.9905	0.0022	-0.0026	1.0000	1.1189	0.0717
AT5G15500	-0.0155	0.9903	0.2573	0.8159	-0.1847	0.8927	-0.2825	0.8394	0.5736	0.6754	-0.9771	0.2631	0.0862	0.9317	-0.4846	0.7798	-0.6135	0.4761	1.3140	0.1970	1.5118	0.1060	2.0315	0.0054	-0.3540	0.8270	-0.3232	0.6879
AT5G15600	-1.6711	0.0012	-0.9109	0.1263	-1.5233	0.0094	-1.0541	0.1350	-2.0020	0.0002	-1.4432	0.0141	-1.6625	0.0007	-1.7897	0.0014	-1.6236	0.0024	-1.5373	0.0116	-0.8437	0.2413	-1.9964	0.0001	-1.6137	0.0044	-2.8302	0.0000
AT5G15800	-0.9902	0.2092	-1.0145	0.2430	-0.2203	0.8632	-0.4613	0.7099	-0.2823	0.8638	-0.6229	NA	0.2764	0.7597	-0.7944	0.5419	-1.3299	0.0879	-0.5695	0.6960	-1.9723	0.0217	-1.0971	0.1668	-1.9113	0.0187	-2.0296	0.0038
AT5G15830	-1.6172	0.0021	-2.0027	0.0001	-0.6332	0.3998	-1.3312	0.0512	-0.5851	0.4930	-1.1209	0.0636	-1.8150	0.0003	-0.7975	0.3183	-1.9155	0.0003	-1.3925	0.0288	-0.4649	0.5868	-1.7657	0.0007	-1.4394	0.0143	-0.4213	0.4428
AT5G15950	-2.8026	0.0000	-1.6158	0.0098	-1.8691	0.0041	-1.5906	0.0360	-1.8818	0.0030	-2.4963	0.0001	-2.4322	0.0000	-2.1300	0.0007	-2.5570	0.0000	-0.3083	0.8271	0.6774	0.4572	-2.3851	0.0000	-1.7956	0.0052	-3.2329	0.0000
AT5G15960	-0.3168	0.7486	1.8636	0.0174	0.9761	0.3439	0.4838	0.7062	0.8326	0.4847	0.8877	0.3232	-0.2829	0.7671	0.9022	0.4800	2.1167	0.0050	1.6080	0.0929	0.3484	0.8081	3.2636	0.0000	2.7375	0.0003	1.6879	0.0182
AT5G15970	-1.1395	0.0454	1.2964	0.0280	0.5548	0.4964	-0.0366	0.9779	0.9994	0.1674	1.1639	0.0625	-0.5360	0.3829	1.0641	0.1420	2.0879	0.0001	1.7929	0.0036	-0.0541	0.9685	0.0156	0.9881	2.9651	0.0000	1.7596	0.0008
AT5G16023	-2.8930	0.0000	-0.9139	0.3090	-0.3194	0.7983	-1.4275	0.1562	0.3028	0.8558	-1.3172	0.1202	-3.0747	0.0000	-0.0431	0.9897	-2.0046	0.0075	-1.2464	0.2351	-0.1237	0.9427	-2.7601	0.0001	-0.3513	0.8292	-1.0883	0.1306
AT5G16170	-1.4149	0.0235	-2.0415	0.0009	-1.0751	0.1765	-1.0324	0.2390	-0.5648	0.5997	-1.1137	0.1163	-1.7023	0.0039	-0.9606	0.2877	-1.8860	0.0025	-0.9809	0.2762	-0.3736	0.7342	-1.7196	0.0052	-1.6516	0.0149	-0.4961	0.4342
AT5G16190	0.2797	0.7681	-1.7969	0.0155	-1.3872	0.1161	-0.4960	0.6787	-0.3421	0.8256	-1.8862	0.0162	0.3535	0.6844	-0.2679	0.8955	-2.1475	0.0027	0.0180	0.9941	0.5797	0.6182	0.3082	0.7536	-1.1065	0.2517	-2.0293	0.0028
AT5G16380	1.3389	0.0001	1.0443	0.0043	1.4624	0.0000	1.3580	0.0007	1.7238	0.0000	1.4793	0.0001	1.5902	0.0000	1.6266	0.0000	1.6754	0.0000	1.5945	0.0000	0.6819	0.1604	1.6117	0.0000	1.6192	0.0000	2.3447	0.0000
AT5G17170	-0.9718	0.0196	-0.9127	0.0397	-0.9196	0.0611	-0.6723	0.2543	-0.7055	0.1993	-1.1670	0.0102	-0.6411	0.1298	-0.8530	0.1006	-1.4727	0.0003	-0.6058	0.3355	0.2350	0.7531	-1.3204	0.0010	-1.1370	0.0111	-2.4306	0.0000
AT5G17230	-0.1461	0.8594	-0.8573	0.2108	-0.4807	0.5845	-0.4323	0.6569	-0.1970	0.8850	-1.1736	0.0743	0.0307	0.9703	-0.3374	0.8176	-1.6458	0.0051	-0.3056	0.8220	0.3719	0.7168	-0.2701	0.7342	-0.5211	0.6131	-2.4094	0.0000
AT5G17300	-2.7066	0.0000	-0.9290	0.1452	-1.1261	0.1003	-1.0924	0.1486	-3.0530	0.0000	-0.4117	0.5635	-2.9788	0.0000	-1.8919	0.0012	-0.6397	0.3062	-0.3936	0.7356	0.1226	0.9210	-2.5674	0.0000	-2.2741	0.0000	-0.8060	0.1387
AT5G17350	-0.6069	0.4298	-0.8343	0.3002	-1.5268	0.0531	-0.2204	0.8656	-1.9203	0.0081	-1.5684	0.0360	-0.1565	0.8581	-1.5160	0.0580	-1.7830	0.0092	-0.6809	0.5684	2.9410	0.0000	-0.7917	0.2934	-2.0772	0.0036	-0.3332	0.6413
AT5G17390	-1.9070	0.0106	-2.1097	0.0057	-1.6090	0.0731	0.4696	0.7145	-1.2363	0.2183	-1.4749	0.0814	-2.6336	0.0002	-1.4946	0.1169	-0.4367	0.6336	-1.0610	0.3570	-1.5784	0.0937	-2.0915	0.0049	-2.3512	0.0026	-1.4352	0.0451
AT5G17460	0.1448	0.8889	2.2380	0.0019	1.8118	0.0278	1.5243	0.1128	1.4513	0.1039	2.0232	0.0105	0.1994	0.8342	2.0905	0.0081	2.8660	0.0000	2.6425	0.0006	-0.0003	1.0000	1.4939	0.0461	3.4218	0.0000	4.0129	0.0000
AT5G17523	0.9009	0.2724	0.9109	0.3163	0.9929	0.3327	0.5001	0.6940	3.9280	0.0000	-0.5180	0.5901	0.7845	0.3328	0.6345	0.6821	0.3069	0.7507	0.6224	0.6694	0.6470	0.5908	0.6506	0.4708	1.3730	0.1424	0.5744	0.4567
AT5G17760	-2.7672	0.0000	-0.8473	0.0246	-0.8341	0.0450	-1.2668	0.0020	-0.0854	0.9243	-1.0276	0.0089	-2.0388	0.0000	-0.7923	0.0655	-0.9803	0.0068	-0.8067	0.0705	-1.5924	0.0000	-2.1546	0.0000	-0.7065	0.1043	-1.3108	0.0001
AT5G17780	-1.7866	0.0031	-1.2272	0.0678	-0.5526	0.5423	-1.0366	0.2273	0.1124	0.9440	-2.2980	0.0006	-1.1187	0.0643	-0.4386	0.7506	-2.1912	0.0004	-1.9381	0.0052	-1.4366	0.0550	-1.6217	0.0082	-0.3030	0.8192	-2.1756	0.0003
AT5G17860	-1.3182	0.0000	-0.6387	0.0752	-0.3091	0.5201	-0.4244	0.3842	-0.3103	0.5788	-0.5893	0.1153	-2.3748	0.0000	-0.9796	0.0057	-0.7737	0.0218	-0.7145	0.0832	-1.8619	0.0000	-1.6457	0.0000	-0.5517	0.1958	-0.1471	0.6787
AT5G18020	-1.7464	0.0002	-0.0133	0.9875	-0.1076	0.9046	-0.6760	0.3393	0.0546	0.9697	-0.2754	0.6636	-2.0700	0.0000	-0.0881	0.9581	-0.9491	0.0614	-0.7377	0.3012	0.8771	0.1653	-1.1242	0.0221	0.2817	0.7758	-0.0616	0.9123
AT5G18050	-2.3927	0.0000	-0.3230	0.6611	0.0140	0.9903	-0.5223	0.5393	0.4074	0.6828	0.0916	0.9101	-2.3945	0.0000	0.2102	0.8874	-0.7695	0.1849	-1.0810	0.1230	0.4451	0.6082	-2.0269	0.0001	0.6704	0.4041	0.1889	0.7481
AT5G18060	-1.8755	0.0000	-0.1362	0.8454	-0.0478	0.9601	-0.5434	0.4444	0.4270	0.5914	-0.0859	0.9029	-2.1142	0.0000	0.1719	0.8968	-0.9262	0.0582	-0.9191	0.1349	0.6321	0.3343	-1.6339	0.0003	0.4627	0.5495	0.7392	0.0963
AT5G18570	0.3240	0.5871	-0.6379	0.2771	-0.8462	0.1777	-0.4128	0.6030	-0.3413	0.7159	-0.9661	0.0804	0.2486	0.6759	0.3274	0.7741	-1.0461	0.0396	-0.7835	0.2691	-0.3357	0.6914	0.3549	0.5614	-0.6805	0.3313	-2.2355	0.0000
AT5G18660	0.7742	0.1878	-0.4050	0.5774	-0.3312	0.7032	-0.3723	0.6880	-0.0384	0.9836	-1.0129	0.1038	0.7996	0.1559	0.2365	0.8749	-1.2915	0.0219	-0.6541	0.4742	-0.1350	0.9120	0.5618	0.3797	-0.5398	0.5596	-2.7962	0.0000
AT5G18670	-1.1183	0.0291	-1.2547	0.0176	-1.1064	0.0664	-0.8077	0.2630	-2.1884	0.0000	-0.9041	0.1181	-2.3180	0.0000	-1.5655	0.0039	-0.8992	0.0936	-0.8629	0.2256	-0.2352	0.8077	-1.7248	0.0004	-1.6564	0.0015	-1.2892	0.0074
AT5G19230	-1.8474	0.0007	-0.5841	0.4055	0.1927	0.8430	-0.3753	0.6938	-1.7799	0.0023	0.1517	0.8537	-2.6413	0.0000	-1.2742	0.0574	-0.3385	0.6215	-0.4896	0.6502	-1.4595	0.0266	-2.3475	0.0000	-1.0238	0.1471	0.2739	0.6501
AT5G19500	-0.3953	0.4677	-0.9617	0.0574	-0.7921	0.1873	-0.5164	0.4731	-0.7826	0.2063	-1.5690	0.0018	-0.3445	0.5174	-0.5977	0.4303	-1.7577	0.0001	-0.7626	0.2528	0.2519	0.7650	-0.5890	0.2571	-0.7237	0.2510	-2.2669	0.0000
AT5G19600	-2.8780	0.0000	-0.2182	0.8144	-0.5049	0.5907	0.0941	0.9458	-0.2113	0.8833	-0.7799	0.2953	-3.2687	0.0000	-0.2382	0.8921	-0.3530	0.6452	-0.8360	0.3965	-0.3167	0.7833	-2.2740	0.0002	-0.8839	0.3216	-1.2987	0.0314
AT5G19730	-1.5005	0.0156	-0.9277	0.1999	-1.6901	0.0144	-0.7991	0.3887	-1.1067	0.1703	-1.7992	0.0082	-1.5781	0.0078	-0.9010	0.3363	-1.7549	0.0050	-0.2556	0.8717	0.6349	0.5016	-0.4800	0.5288	-0.6226	0.5490	-2.0483	0.0005
AT5G19850	-1.0558	0.0027	-0.8002	0.0370	-1.1835	0.0018	-0.6711	0.1716	-1.6304	0.0000	-1.4729	0.0001	-0.8881	0.0097	-1.7497	0.0000	-1.4815	0.0000	-0.9404	0.0264	-0.5064	0.3179	-0.8896	0.0138	-1.2907	0.0004	-2.2887	0.0000
AT5G19880	-1.7911	0.0169	-2.7586	0.0001	-1.8944	0.0258	-1.1705	0.2745	-1.4423	0.1256	-1.5932	NA	-1.3444	0.0713	-2.2108	0.0066	-2.3592	0.0015	-2.0544	0.0181	-1.8054	0.0443	-0.8937	0.2910	-1.9255	0.0194	-1.3539	0.0594
AT5G19970	-2.1187	0.0003	-0.2447	0.7760	-0.6827	0.4158	-1.0392	0.2095	-1.4599	0.0345	-1.0203	0.1388	-2.3218	0.0000	-1.2609	0.0863	-0.8458	0.1895	-0.0145	0.9942	-0.4803	0.6194	-1.8221	0.0019	-1.0517	0.1702	-1.1328	0.0501
AT5G20110	-2.0735	0.0002	-0.9854	0.1342	-0.3199	0.7327	-1.2370	0.1062	0.1725	0.9002	-0.5466	0.4466	-2.3766	0.0000	0.0413	0.9870	-1.1121	0.0683	-0.4913	0.6611	-0.4213	0.6681	-1.5942	0.0057	0.4584	0.6670	-0.5805	0.3168
AT5G20140	0.0645	0.9117	-0.4793	0.3351	-0.2775	0.6601	-0.4745	0.4474	-0.1084	0.9134	-0.8274	0.0711	0.1096	0.8383	-0.2960	0.7470	-1.0378	0.0125	-0.7950	0.1411	-0.2032	0.7892	-0.1133	0.8509	-0.5722	0.3248	-2.3997	0.0000
AT5G20260	-0.3536	0.7142	1.3546	0.0955	0.8129	0.4433	0.8924	0.4225	0.5491	0.6945	0.6619	NA	0.3678	0.6803	0.9734	0.4064	2.0481	0.0049	2.2054	0.0065	1.8067	0.0361	-0.1649	0.8840	-0.3272	0.8446	1.6214	0.0203
AT5G20630	1.4249	0.0022	0.6478	0.2524	0.3736	0.6044	0.4220	0.5828	1.3193	0.0104	-0.4973	0.3910	1.5410	0.0005	1.1575	0.0359	0.5245	0.3268	1.1193	0.0558	1.3456	0.0132	2.0099	0.0000	0.9292	0.1099	0.5685	0.2261
AT5G20790	-0.1219	0.9070	-1.7305	0.0262	-1.0598	0.2809	-0.9189	0.4023	-0.6626	0.6026	-1.2846	0.1268	0.3801	0.6644	-0.8530	0.4987	-1.2889	0.0980	0.4188	0.7963	0.7403	0.5048	0.1663	0.8791	-1.8424	0.0249	-2.1620	0.0021
AT5G21100	-0.5539	0.5068	-1.7871	0.0143	-1.4851	0.0791	-0.6080	0.5932	-0.8014	0.4655	-2.1975	0.0038	-0.5623	0.4818	-0.9381	0.4031	-2.2298	0.0014	0.0024	1.0000	0.4378	0.7240	-0.3187	0.7403	-0.6197	0.6185	-1.9979	0.0027
AT5G21430	-0.6270	0.1740	-0.7223	0.1412	-0.5056	0.4061	-0.8638	0.1350	-0.4226	0.5610	-0.7414	0.1343	-0.3536	0.4686	-0.7473	0.2062	-1.0459	0.0181	-0.8445	0.1393	-0.1803	0.8322	-0.6421	0.1717	-0.9886	0.0456	-2.1799	0.0000
AT5G22140	0.2528	0.7824	-0.1096	0.9198	-0.2564	0.8285	2.9506	0.0001	0.7821	0.4623	-0.3714	0.6836	0.3146	0.7084	0.5195	0.7281	-0.1976	0.8284	0.0080	0.9974	-0.1322	0.9325	0.3565	0.6959	-0.0112	0.9984	-0.7022	0.3092
AT5G22270	-0.6938	0.1598	-0.6048	0.2771	0.2404	0.7510	-0.1498	0.8695	0.1403	0.8983	0.9371	0.0706	-0.5565	0.2565	-0.2206	0.8583	0.0448	0.9466	-0.5032	0.5356	-2.1542	0.0000	-0.5576	0.2874	0.2874	0.7568	0.8951	0.0423
AT5G22290	-2.1623	0.0000	0.0133	0.9848	-0.2863	0.6409	-0.4238	0.4926	0.0776	0.9408	0.2095	0.6923	-1.9378	0.0000	-0.1800	0.8668	0.1732	0.7323	0.1507	0.8859	-0.6090	0.2692	-1.3756	0.0003	0.4857	0.4229	-0.0297	0.9494
AT5G22300	-1.4255	0.0268	-2.0321	0.0013	-1.4731	0.0477	0.3905	0.7236	-0.1073	0.9502	-1.4536	0.0405	-1.1711	0.0651	-0.8574	0.3974	-1.6445	0.0112	-0.6172	0.5997	-0.3449	0.7661	-1.1403	0.0903	-1.3423	0.0750	-1.3234	0.0294
AT5G22310	-2.0121	0.0001	-0.2834	0.7101	0.2490	0.7819	-0.8471	0.2743	0.2856	0.8021	-0.1449	0.8545	-1.9697	0.0001	0.0499	0.9799	-0.5641	0.3569	-0.9927	0.1795	-1.2199	0.0663	-1.9718	0.0001	0.3805	0.7137	-0.2511	0.6673
AT5G22380	-2.6921	0.0001	-3.0752	0.0000	-1.8576	0.0218	-1.8162	0.0448	-0.5816	0.6545	-2.1169	0.0072	-2.5392	0.0001	-1.9870	0.0123	-2.6622	0.0002	-2.2593	0.0050	0.0588	0.9748	-2.6810	0.0001	-2.3009	0.0022	-0.8701	0.2171
AT5G22390	-2.0108	0.0000	-0.7763	0.0646	-1.4667	0.0002	-0.7439	0.1538	-2.0439	0.0000	-0.1593	0.7627	-2.4387	0.0000	-1.7844	0.0000	-0.8073	0.0439	-1.2105	0.0048	-0.4116	0.4799	-2.3147	0.0000	-1.6212	0.0000	-2.1890	0.0000
AT5G22500	-2.3812	0.0000	-1.2584	0.0539	0.4446	0.6310	-2.3051	0.0006	0.5079	0.6406	-2.3006	0.0003	-2.4523	0.0000	-0.6659	0.5346	-2.4199	0.0000	-2.2099	0.0006	-2.7686	0.0000	-2.3377	0.0000	0.1102	0.9422	-1.5865	0.0056
AT5G22520	-3.4541	0.0000	-3.1332	0.0000	-1.6273	0.0147	-2.0429	0.0035	-1.2872	0.0737	-1.6012	0.0152	-3.0667	0.0000	-3.0794	0.0000	-3.3541	0.0000	-2.7603	0.0000	-1.8346	0.0061	-4.2959	0.0000	-2.8596	0.0000	-2.4909	0.0000
AT5G22530	-3.6845	0.0000	-3.2217	0.0000	-1.8099	0.0143	-1.6854	0.0431	-0.9381	0.3184	-1.9753	0.0068	-3.1156	0.0000	-2.8524	0.0000	-2.9750	0.0000	-2.3864	0.0009	-1.9800	0.0082	-4.1867	0.0000	-2.1160	0.0024	-1.8174	0.0043
AT5G22540	-1.4608	0.0504	-1.5526	0.0490	-1.2210	0.1964	-0.3157	0.8167	-0.4135	0.7829	-1.0572	0.2157	-2.0106	0.0038	-1.8726	0.0243	-1.7026	0.0243	-0.9547	0.4115	-0.4481	0.7275	-2.1555	0.0027	-1.5598	0.0705	-0.0074	0.9937
AT5G22570	-1.8739	0.0125	-2.5058	0.0007	-0.9720	0.3471	-1.3683	0.1876	-0.2915	0.8641	-1.8382	0.0270	-1.5840	0.0314	-1.7210	0.0543	-2.5912	0.0005	-1.4509	0.1464	-0.9864	0.3625	-2.1113	0.0045	-1.3280	0.1658	0.1928	0.8190

AT5G22800	0.1036	0.8713	-0.7354	0.1538	-0.4334	0.5130	-0.5669	0.4027	-0.3399	0.6869	-0.9781	0.0522	0.1335	0.8210	-0.2086	0.8623	-1.0557	0.0229	-0.6484	0.3466	-0.3102	0.6907	0.1319	0.8424	-0.7793	0.1788	-2.1058	0.0000
AT5G23010	1.1558	0.0638	-2.2541	0.0001	-1.2547	0.0871	-0.1633	0.8933	0.2451	0.8577	-1.4394	0.0318	0.9018	0.1471	0.3438	0.8212	-0.9252	0.1546	-0.6830	0.5106	-0.3878	0.7149	1.3055	0.0359	-0.2399	0.8618	-1.2374	0.0319
AT5G23060	0.7921	0.0510	-0.1619	0.7812	-0.4036	0.4855	0.0118	0.9917	-0.6446	0.2286	-0.5130	0.2748	1.0321	0.0065	-0.2937	0.7392	-0.7765	0.0618	-0.6090	0.2981	0.5649	0.3113	0.5717	0.1877	-0.8144	0.0861	-2.0960	0.0000
AT5G23210	2.0808	0.0000	-0.4067	0.3332	0.5192	0.2530	-0.4552	0.3741	0.1340	0.8629	-0.1224	0.7990	1.2110	0.0001	-0.0428	0.9725	-0.6090	0.0957	-0.1579	0.8478	0.1472	0.8271	1.3889	0.0000	-0.0352	0.9728	0.1856	0.6129
AT5G23240	1.7840	0.0029	0.2081	0.8217	2.2244	0.0003	0.1993	0.8693	3.5196	0.0000	0.3982	0.6146	2.7694	0.0000	1.5480	0.0264	0.3854	0.6064	1.3024	0.0967	0.1966	0.8794	2.7826	0.0000	3.1735	0.0000	1.9780	0.0006
AT5G23350	-1.3261	0.0170	-0.3996	0.5886	-0.3838	0.6539	-0.3586	0.7039	-1.1230	0.0997	0.0425	0.9624	-2.2844	0.0000	-0.5899	0.5570	-0.1271	0.8651	-0.8153	0.3304	-1.0185	0.1639	-1.8327	0.0006	-0.6146	0.4879	-0.8398	0.1190
AT5G23575	1.2689	0.0007	1.6750	0.0000	1.7769	0.0000	1.3005	0.0040	1.8058	0.0000	1.5245	0.0002	1.6118	0.0000	1.6889	0.0000	1.8403	0.0000	1.7583	0.0000	0.0428	0.9641	1.6441	0.0000	2.1432	0.0000	1.8311	0.0000
AT5G23700	-0.4253	0.6531	1.6213	0.0449	2.0499	0.0140	1.4119	0.1712	-0.0873	0.9691	2.1490	0.0090	-0.0788	0.9396	0.8826	0.4968	1.7732	0.0219	1.3755	0.1823	-0.4049	0.7677	-0.1648	0.8854	0.5127	0.7266	1.9726	0.0057
AT5G23730	-1.8834	0.0004	-1.5869	0.0045	-1.4822	0.0161	-1.3314	0.0597	-2.0582	0.0002	-1.8803	0.0015	-1.8667	0.0003	-2.0428	0.0003	-2.1644	0.0001	-0.7828	0.3543	0.2012	0.8560	-1.7810	0.0009	-2.1063	0.0001	-2.2046	0.0000
AT5G23830	1.8523	0.0099	0.8173	0.3699	0.1615	0.9083	0.0070	0.9977	1.1101	0.2768	-0.0062	NA	1.4921	0.0347	0.8301	0.5214	-0.0792	0.9408	1.1101	0.3094	2.6268	0.0005	1.0436	0.1922	0.5185	0.7179	-0.0549	0.9503
AT5G23980	-0.3410	0.6319	0.6274	0.3694	1.5007	0.0167	0.0744	0.9530	1.6844	0.0044	0.5993	0.3849	-1.0940	0.0532	1.2312	0.0708	-0.0330	0.9696	-0.0386	0.9826	-0.7724	0.3527	-0.2073	0.7950	2.2616	0.0000	0.6617	0.2388
AT5G24080	-1.8142	0.0126	0.5211	0.5894	-0.1112	0.9385	-0.2762	0.8400	-0.7323	0.5433	-0.6309	0.4889	-2.1422	0.0018	-1.6972	0.0484	0.2871	0.7583	-0.2156	0.9160	-1.7754	0.0422	-1.4129	0.0630	-0.5563	0.6840	-1.1660	0.0979
AT5G24110	-3.4069	0.0000	-2.0998	0.0000	-0.2938	0.7147	-1.7058	0.0042	-1.0275	0.0997	-0.7606	0.1982	-2.9778	0.0000	-1.8689	0.0004	-1.5042	0.0034	-1.6711	0.0031	-0.8949	0.1864	-3.3465	0.0000	-1.7983	0.0006	-1.3358	0.0063
AT5G24120	-2.8798	0.0000	-1.5368	0.0021	-1.7980	0.0004	-1.8408	0.0012	-2.6298	0.0000	-2.1720	0.0000	-3.1657	0.0000	-2.8523	0.0000	-2.3280	0.0000	-1.0375	0.1088	-1.3386	0.0210	-2.5731	0.0000	-1.5638	0.0027	-3.1370	0.0000
AT5G24150	-2.0443	0.0011	-2.3822	0.0001	-2.8074	0.0000	-1.2587	0.1475	-2.4467	0.0001	-3.1495	0.0000	-2.2890	0.0001	-3.4454	0.0000	-2.7615	0.0000	-1.4450	0.0730	-1.2926	0.1167	-1.3532	0.0420	-2.2000	0.0008	-3.6885	0.0000
AT5G24155	-1.4164	0.0491	-1.5124	0.0466	-1.7717	0.0283	-1.0324	0.3101	-2.4164	0.0009	-2.6654	0.0005	-1.6547	0.0147	-2.5673	0.0005	-2.0147	0.0048	-1.0507	0.3197	-0.7027	0.5167	-0.0181	0.9887	-0.6461	0.5965	-2.1961	0.0012
AT5G24200	-2.2864	0.0007	-2.3550	0.0006	-0.7665	0.4435	-1.9074	0.0279	-0.0307	0.9913	-1.8936	0.0137	-1.9324	0.0036	-2.1153	0.0050	-2.9003	0.0000	-1.5457	0.0784	-1.7123	0.0400	-2.2922	0.0007	-1.4536	0.0802	-0.9853	0.1472
AT5G24205	-1.2256	0.0271	-1.1958	0.0447	-0.5844	0.4584	-0.9649	0.2048	-0.1210	0.9297	-1.7528	0.0048	-0.6778	0.2301	-1.0280	0.1533	-1.8799	0.0009	-1.2283	0.0776	-1.3959	0.0355	-1.1848	0.0355	-1.2160	0.0623	-2.1229	0.0001
AT5G24210	-1.3070	0.0566	-2.4600	0.0001	-1.2226	0.1473	-1.5513	0.0737	-0.6030	0.6051	-1.9052	0.0092	-1.5269	0.0185	-2.0634	0.0039	-2.7216	0.0000	-1.3539	0.1203	-0.8054	0.4130	-1.6012	0.0180	-1.7927	0.0134	-2.2413	0.0004
AT5G24215	-0.5789	0.1252	-1.3838	0.0001	-0.9634	0.0187	-0.9567	0.0334	-2.0966	0.0000	-1.3211	0.0010	-0.3583	0.3533	-1.7816	0.0000	-1.5973	0.0000	-1.0969	0.0078	-0.4163	0.4379	-0.1931	0.6820	-1.7737	0.0000	-0.9120	0.0098
AT5G24230	-0.5664	0.4678	-2.1030	0.0021	-0.8840	0.3360	-1.3364	0.1377	-0.7128	0.5089	-1.4776	0.0519	-0.9427	0.1693	-1.2955	0.1354	-1.7111	0.0138	-0.6983	0.5571	-0.8819	0.3615	-1.0470	0.1463	-1.5571	0.0457	-1.4588	0.0249
AT5G24314	0.0235	0.9729	-0.2862	0.6366	-0.3355	0.6231	-0.4620	0.5065	0.1358	0.8977	-0.4538	0.4064	0.0332	0.9577	0.0521	0.9745	-0.5933	0.2229	-0.5139	0.4965	-0.6480	0.2995	-0.0295	0.9693	-0.4126	0.5927	-2.0152	0.0000
AT5G24470	2.5473	0.0001	3.0184	0.0000	3.0542	0.0000	2.6974	0.0004	2.8875	0.0000	3.0106	0.0000	2.7582	0.0000	2.9742	0.0000	3.7335	0.0000	3.4517	0.0000	-0.5845	0.5965	3.0705	0.0000	2.9334	0.0000	3.3473	0.0000
AT5G24530	-2.0359	0.0000	-1.2480	0.0080	-1.0323	0.0565	-1.2662	0.0258	-0.9135	0.1089	-0.2284	0.7106	-2.5550	0.0000	-1.5093	0.0018	-0.2464	0.6656	-0.4994	0.5344	-0.7819	0.2058	-2.1302	0.0000	-1.8346	0.0001	-0.0806	0.8787
AT5G24580	-3.2219	0.0000	-1.0583	0.0207	-0.7859	0.1543	-1.1438	0.0354	-1.1358	0.0191	-0.5743	0.2640	-3.3981	0.0000	-0.9386	0.0778	-0.8478	0.0635	-1.5103	0.0014	-2.1465	0.0000	-3.1153	0.0000	-0.4765	0.4975	-0.4160	0.3420
AT5G24640	0.2012	0.8486	-0.2626	0.8152	0.0090	0.9960	2.8683	0.0006	0.3732	0.8179	0.6231	NA	-0.1642	0.8693	0.4300	0.8199	0.6653	0.4431	1.6608	0.0783	-0.4279	0.7508	-0.3148	0.7621	0.3721	0.8201	1.3631	0.0582
AT5G24770	2.2651	0.0001	-0.9366	0.1805	-0.6084	0.4889	0.9595	0.2616	-0.1158	0.9417	0.6803	0.3466	1.4042	0.0154	0.7737	0.4294	-0.1097	0.8940	1.6236	0.0199	1.1242	0.1529	2.0778	0.0003	-0.1314	0.9307	-0.2438	0.7124
AT5G24810	-0.4664	0.4692	-2.0545	0.0001	-0.8676	0.2264	-0.8260	0.2975	-0.3217	0.7733	-1.4154	0.0190	-0.6837	0.2372	-1.0283	0.1534	-2.0800	0.0001	-0.6124	0.5179	0.1231	0.9199	-0.7004	0.2539	-1.0538	0.1185	-1.9617	0.0001
AT5G24910	-2.1496	0.0028	-0.6642	0.4702	-0.8329	0.4173	-1.4466	0.1378	-0.9150	0.4017	-0.9106	NA	-0.9523	0.2034	-0.2643	0.8985	-0.5652	0.5041	-0.3302	0.8477	-1.2379	0.2077	-1.6098	0.0310	-0.7447	0.5370	0.5040	0.4968
AT5G25110	-0.3466	0.7092	0.0092	0.9951	1.2908	0.1566	-0.3201	0.8121	2.0395	0.0081	-0.6707	0.4578	-0.5454	0.5097	1.5025	0.0918	-0.5706	0.5010	0.3135	0.8598	1.2078	0.2140	1.0772	0.1684	2.0589	0.0071	1.5874	0.0204
AT5G25130	-1.4764	0.0008	-0.5680	0.2995	-1.0221	0.0567	-0.6744	0.3028	-1.1301	0.0283	-0.9882	0.0526	-1.7089	0.0000	-1.7037	0.0003	-0.7810	0.1057	-1.1395	0.0365	-0.6066	0.3514	-1.3230	0.0031	-1.2855	0.0087	-2.4723	0.0000
AT5G25190	-2.6533	0.0000	-1.2559	0.0225	-0.5778	0.4399	-0.6643	0.3981	-0.0970	0.9440	-0.3980	0.5489	-3.6265	0.0000	-0.0930	0.9592	-1.5368	0.0034	-1.5971	0.0067	-0.5643	0.4748	-2.4125	0.0000	0.9596	0.1408	-0.7964	0.1164
AT5G25250	-1.8728	0.0010	-2.2207	0.0001	-0.9283	0.2257	-1.2685	0.1012	-0.7652	0.3794	-1.2491	0.0570	-1.5807	0.0047	-2.3503	0.0001	-2.4766	0.0000	-1.6595	0.0137	-1.5600	0.0219	-2.6402	0.0000	-2.0006	0.0008	-1.8300	0.0010
AT5G25260	-3.7977	0.0000	-3.2244	0.0000	-1.4819	0.0082	-2.4687	0.0000	-1.2542	0.0318	-1.8452	0.0008	-3.2484	0.0000	-3.5930	0.0000	-3.7165	0.0000	-3.3016	0.0000	-3.0532	0.0000	-4.0611	0.0000	-2.8522	0.0000	-2.4727	0.0000
AT5G25450	-1.6415	0.0092	-2.2519	0.0004	-1.7800	0.0124	-0.7911	0.4005	-1.1966	0.1367	-1.7189	0.0154	-1.9409	0.0011	-2.1547	0.0014	-2.3703	0.0002	-1.7687	0.0171	-0.9887	0.2538	-1.8523	0.0031	-2.1544	0.0014	-1.9062	0.0021
AT5G25810	1.9309	0.0011	0.6736	0.4078	0.2778	0.8065	0.5563	0.5911	0.6924	0.5111	1.3234	0.0658	1.8262	0.0013	1.0555	0.2314	1.5078	0.0200	2.2124	0.0007	2.6573	0.0000	2.0754	0.0004	1.7581	0.0089	1.7354	0.0039
AT5G25910	-1.5039	0.0000	-2.0674	0.0000	-0.7569	0.0824	-1.5043	0.0002	-0.8483	0.0432	-0.4443	0.3011	-2.1532	0.0000	-1.6236	0.0000	-1.4749	0.0000	-1.4128	0.0003	-0.9996	0.0171	-1.7799	0.0000	-1.4577	0.0001	-0.7954	0.0227
AT5G25930	-1.2146	0.0305	-2.1401	0.0001	-0.9680	0.1728	-0.3066	0.7545	-1.2496	0.0564	-0.9278	0.1467	-1.9140	0.0002	-1.5489	0.0115	-1.7793	0.0013	-1.3266	0.0502	-0.5741	0.4980	-1.8643	0.0005	-1.7799	0.0020	-1.4728	0.0053
AT5G26146	0.0369	0.9745	2.0791	0.0062	1.7629	0.0415	0.2381	0.8719	0.0902	0.9674	-0.3640	NA	-0.5596	0.5147	0.2032	0.9283	0.6576	0.4461	-0.2785	0.8878	2.1476	0.0111	-0.3573	0.7232	0.8041	0.5084	1.4496	0.0421
AT5G26200	-2.2265	0.0000	1.2583	0.0071	0.7320	0.2237	0.6692	0.3159	-0.5471	0.4553	0.7752	0.1448	-1.7566	0.0001	-0.0697	0.9676	1.0844	0.0217	0.0514	0.9726	-0.4773	0.5106	-2.7099	0.0000	-0.4565	0.5715	-0.9441	0.0418
AT5G26220	0.3838	0.6629	-2.3420	0.0008	-0.6292	0.5469	-0.2028	0.8842	1.0998	0.2475	-1.4886	0.0592	-0.0856	0.9281	1.0673	0.2978	-2.0352	0.0041	-1.2103	0.2183	-1.1352	0.2344	0.0875	0.9381	-0.1804	0.9174	-1.8122	0.0071
AT5G26270	0.0936	0.9111	-0.1420	0.8775	2.0103	0.0006	0.2484	0.8196	-0.1027	0.9487	-0.9019	0.2118	-0.0524	0.9486	-0.4095	0.7652	-0.6267	0.3623	-0.7499	0.4494	1.9340	0.0016	0.1595	0.8536	-0.0423	0.9827	1.9009	0.0004
AT5G26340	-0.8135	0.1169	-0.1006	0.8968	0.2834	0.7197	0.7018	0.3312	0.0700	0.9608	0.7156	0.2144	-0.8192	0.1008	-0.1944	0.8874	0.4582	0.4185	0.2103	0.8648	-2.0344	0.0001	-0.8079	0.1273	-0.0218	0.9905	0.9857	0.0361
AT5G26690	-2.3130	0.0015	-2.8106	0.0001	-1.6334	0.0665	-2.0604	0.0259	-0.2696	0.8756	-2.5023	0.0018	-1.9415	0.0066	-2.1641	0.0080	-2.8924	0.0001	-1.8095	0.0463	-1.2196	0.2303	-2.1132	0.0043	-1.7128	0.0456	-0.8768	0.2351
AT5G26920	-2.7535	0.0000	-2.6379	0.0000	-1.2811	0.0323	-1.6296	0.0094	-1.0238	0.1163	-1.0021	0.0903	-2.6150	0.0000	-2.1411	0.0000	-2.2206	0.0000	-1.2998	0.0381	-0.1702	0.8762	-2.8054	0.0000	-1.7452	0.0011	-1.1649	0.0192
AT5G27060	-1.9438	0.0057	-3.0041	0.0000	-2.5813	0.0004	-0.7054	0.5266	-1.3867	0.1186	-1.6962	0.0320	-2.8786	0.0000	-1.5648	0.0685	-2.2208	0.0019	-0.6007	0.6611	-1.1001	0.2596	-2.3504	0.0006	-1.5913	0.0532	-1.4791	0.0296
AT5G27220	-2.1877	0.0000	-0.7631	0.2442	0.0545	0.9617	-1.0037	0.1879	-0.0018	1.0000	-1.0108	0.1123	-1.7662	0.0006	-0.0959	0.9599	-1.0052	0.0874	-0.1572	0.9191	-0.5423	0.5258	-2.2167	0.0000	-0.0367	0.9842	0.2363	0.6926
AT5G27765	-2.2652	NA	-1.7587	0.0209	-1.9561	0.0144	-1.2127	NA	-0.9900	0.3598	-0.8559	NA	-2.0558	0.0032	-1.9436	NA	-1.9384	NA	-1.8185	NA	-1.9496	0.0186	-2.0438	0.0045	-1.9015	0.0146	-0.9895	NA
AT5G27930	0.7997	0.0205	0.7478	0.0434	0.7026	0.0951	0.6958	0.1305	1.9445	0.0000	0.6542	0.0966	0.8556	0.0091	1.1888	0.0008	0.9479	0.0064	1.3860	0.0001	0.4432	0.3768	1.1175	0.0007	2.0531	0.0000	1.7305	0.0000
AT5G28237	-1.8827	0.0059	0.0926	0.9326	0.0178	0.9903	0.9269	0.3453	-1.4590	0.0812	0.3708	0.6766	-2.2731	0.0004	-0.6605	0.6124	0.5391	0.4920	0.3391	0.8284	-1.3456	0.1334	-2.1166	0.0018	-0.7709	0.4791	0.1995	0.7909
AT5G28320	-1.9180	0.0012	-1.1331	0.0927	-0.6065	0.4949	-1.0808	0.1999	-0.1899	0.8945	-0.1919	0.8249	-2.1573	0.0001	-0.9604	0.2725	-0.4378	0.5429	-0.5768	0.6099	-1.3104	0.0835	-1.9058	0.0013	-0.7576	0.4086	-0.8986	0.1278
AT5G28340	-1.5515	0.0416	-1.7035	0.0324	-1.4303	0.1245	-1.4459	0.1537	0.6680	0.6088	-1.6031	0.0557	-2.3386	0.0009	-1.6920	0.0583	-0.9946	0.2232	-1.9717	0.0249	-2.0684	0.0161	-1.5706	0.0419	-1.3816	0.1393	-1.1774	0.1034
AT5G28400	-1.5909	0.0037	-0.7019	0.2968	-0.2827	0.7561	-0.2913	0.7691	-0.0926	0.9511	0.1395	0.8647	-2.0595	0.0001	-0.2569	0.8623	-0.0194	0.9825	-0.1255	0.9399	-0.6953	0.3914	-1.6241	0.0031	-0.4425	0.6668	-0.3941	0.4945
AT5G28500	0.1785	0.8153	-0.8640	0.1852	-0.6016	0.4577	-0.6543	0.4479	-0.1484	0.9147	-1.4011	0.0237	0.1236	0.8684	0.0342	0.9893	-1.7163	0.0022	-0.7841	0.3659	-0.4292	0.6467	0.1672	0.8388	-0.5562	0.5570	-2.3150	0.0000
AT5G28630	0.1072	0.9171	-2.2021	0.0020	-0.1753	0.8945	-0.9672	0.3571	-0.6167	0.6266	-0.6753	0.4442	-0.4953	0.5483	0.1170	0.9635	-1.9518	0.0068	-0.3075	0.8611	0.7716	0.4713	0.0181	0.9889	-1.1456	0.2269	-0.7281	0.3058
AT5G34780	-0.0346	0.9758	3.9576	0.0000	3.8322	0.0000	0.5965	0.6224	1.3081	0.1774	0.6163	NA	0.6235	0.4552	0.4846	0.7813	0.3638	0.6935	0.2808	0.8857	0.0000	1.0000	0.1683	0.8799	0.4079	0.7922	0.5325	0.4836
AT5G35525	-3.1480	0.0000	-1.0631	0.0958	0.0946	0.9318	-0.8458	0.3057	-1.1927	0.0894	-0.2657	0.7304	-2.2443	0.0000	-1.1691	0.1049	-0.8482	0.1701	-1.2570	0.0836	-0.4243	0.6581	-2.2036	0.0001	-0.0609	0.9709	0.3649	0.5389
AT5G35735	-0.4972	0.4812	-2.1216	0.0003	-0.7800	0.3437	-0.0937	0.9441	-0.5227	0.6241	-1.1773	0.0823	-0.5778	0.3812	-0.9007	0.3132	-1.9536	0.0010	-1.1593	0.1462	0.2317	0.8457	-0.5548	0.4385	-1.3003	0.0635	-0.4068	0.5145
AT5G36220	-2.6637	0.0000	-2.2584	0.0000	-1.3581	0.0070	-1.0907	0.0681	-1.1353	0.0314	-1.4109	0.0051	-2.5876	0.0000	-1.2029	0.0214	-1.7185	0.0002	-1.0140	0.0823	-1.0313	0.0708	-2.6125	0.0000	-1.4157	0.0036	-1.5195	0.0005
AT5G36260	-1.2491	0.1015	-1.5845	0.0450	-0.6290	0.5648	-1.4711	0.1335	-0.9015	0.4150	-1.5942	NA	-1.6051	0.0235	-1.3496	0.1640	-2.5533	0.0005	-1.6834	0.0657	-0.8216	0.4523	-1.3451	0.0801	-0.5506	0.6898	-1.4457	0.0401
AT5G36670	0.0552	0.9524	-0.0550	0.9565	-0.1532	0.8910	0.3001	0.7862	2.2786	0.0002	-0.2476	NA	-0.1917	0.8115	0.0591	0.9794	-0.1396	0.8640	-0.0737	0.9705	-0.1630	0.9023	-0.1130	0.9045	-0.1405	0.9258	0.1111	0.8722
AT5G36740	0.7286	0.3814	-0.0091	0.9953	0.1842	0.8907	0.3999	0.7579	2.3168	0.0023	-0.3067	NA	0.5191	0.5411	0.4935	0.7702	-0.1652	0.8650	0.1831	0.9310	0.3022	0.8327	0.7028	0.4173	0.3937	0.7977	0.3821	0.6201
AT5G36790	0.9028	0.2732	0.5797	0.5557	-0.0430	0.9787	-0.2535	0.8633	3.2239	0.0000	-0.4990	0.6039	-0.2182	0.8242	-0.1512	0.9525	-0.3216	0.7367	-0.4737	0.7714	-0.2745	0.8562	0.0485	0.9701	-0.6296	0.6427	-0.2375	0.7747
AT5G36925	-0.8882	0.1729	-2.3441	0.0001	-1.3238	0.0716	-0.6671	0.4800	-1.2727	0.0890	-1.5212	0.0261	-1.6473	0.0046	-1.8035	0.0073	-2.3144	0.0002	-1.3228	0.0897	0.0842	0.9526	-1.3709	0.0285	-1.4522	0.0385	-1.3526	0.0219
AT5G37260	-3.2714	0.0000	-1.2180	0.0091	-1.2570	0.0126	-1.1959	0.0338	-2.0572	0.0000	-1.1044	0.0285	-2.9205	0.0000	-1.2090	0.0171	-0.6476	0.1864	-0.5010	0.5219	1.0329	0.0584	-3.0771	0.0000	-3.0791	0.0000	-0.6435	0.1455
AT5G37450	-2.0153	0.0062	-1.4836	0.0696	-0.4655	0.6969	-1.5964	0.1074	-0.9397	0.4065	-1.6057	NA	-1.4831	0.0460	-1.5853	0.0860	-0.9797	NA	-1.2005	0.2695	-0.2604	0.8661	-1.3640	0.0850	0.1958	0.9167	-1.1887	NA
AT5G37550	-0.9990	0.2049	-1.6533	0.0345	-1.4267	0.1156	-1.0750	0.3098	-1.0899	0.2872	-2.2450	0.0051	-1.5672	0.0278	-1.8841	0.0235	-2.1486	0.0036	0.2132	0.9175	0.6176	0.5981	-0.5749	0.5210	-1.3870	0.1266	-2.1407	0.0022
AT5G37600	-0.4837	0.5278	-1.5915	0.0170	-0.4921	0.6145	-0.3878	0.7262	1.1903	0.1452	-1.1191	0.1261	-0.3387	0.6620	-0.3583	0.8262	-2.2091	0.0005	-0.5922	0.6232	-0.5208	0.6187	-0.5292	0.4976	0.1153	0.9435	-0.3120	0.6502
AT5G37990	2.0351	0.0009	-0.4036	0.6545	0.0785	0.9523	-0.1489	0.9101	0.4991	0.6772	0.5535	0.4953	1.1006	0.0825	1.5079	0.0414	-0.2459	0.7729	1.9723	0.0051	2.9578	0.0000	1.3802	0.0337	1.0777	0.1963	1.4858	0.0144
AT5G38000	2.0795	0.0032	-0.5577	0.5711	-0.8893	0.3966	0.1297	0.9349	0.8385	0.4618	-0.7927	0.3825	1.2761	0.0767	0.7661	0.5657	-0.6857	0.4244	0.6422	0.6468	2.0486	0.0127	1.8365	0.0111	0.3329	0.8394	-0.4145	0.6018
AT5G38005	-0.0825	0.9371	0.5737	0.5265	0.9532	0.3099	-0.2161	0.8773	0.8324	0.4332	0.9474	0.2472	0.4716	0.5540	0.5799	0.6886	0.4528	0.5943	0.5338	0.7040	0.7739	0.4591	0.0859	0.9396	0.1314	0.9414	2.1054	0.0011
AT5G38020	2.4773	0.0002	-0.0103	0.9941	0.3288	0.7784	0.4085	0.7391	0.8964	0.3920	0.8735	0.2956	1.0332	0.1482	1.0749	0.2934	-0.2126	0.8213	1.9825	0.0128	2.2540	0.0030	1.8216	0.0086	1.1792	0.1955	0.0897	0.9133
AT5G38120	-1.3555	0.0587	-1.9289	0.0078	-1.1547	0.2037	-0.2719	0.8329	-1.5264	0.0698	-2.0626	0.0082	-1.2348	0.0743	-1.5148	0.0763	-1.7541	0.0154	-0.5526	0.6882	0.4445	0.7118	-1.1936	0.1066	-1.5248	0.0650	-2.0337	0.0027
AT5G38200	-2.6760	0.0000	0.9604	0.1575	0.9024	0.2501	0.0550	0.9669	-0.6104	0.5361	0.1053	0.9072	-2.4611	0.0000	0.0400	0.9880	1.0168	0.1066	0.1617	0.9245	-1.2700	0.0937	-2.9810	0.0000	-0.4262	0.7110	0.1664	0.8030
AT5G38250	-1.8532	0.0102	-2.0769	0.0048	-0.9984	0.3089	-1.5813	0.0973	-0.1105	0.9563	-1.6000	0.0489	-2.0463	0.0029	-2.0733	0.0093	-2.8474	0.0001	-0.9483	0.4107	-1.2397	0.2024	-2.0315	0.0047	-1.6657	0.0455	-0.8692	0.2237
AT5G38420	-0.5192	0.2635	-0.6311	0.2025	-0.3379	0.5971	-0.4667	0.4777	-0.1357	0.8925	-0.8679	0.0680	-0.0958	0.8644	-0.2235	0.8384	-1.0299	0.0176	-0.9868	0.0588	-0.0474	0.9630	-0.7693	0.0850	-1.5675	0.0002	-2.3128	0.0000
AT5G38430	-0.0058	0.9968	-1.1281	0.1448	-0.8730	0.3507	-0.2554	0.8429	1.1620	0.1961	-1.4635	0.0541	0.5363	0.4888	-0.3599	0.8376	-1.7639	0.0105	-0.7563	0.5198	0.9222	0.3429	0.1052	0.9213	-1.3975	0.0847	-2.2163	0.0005
AT5G38700	0.5604	0.5097	0.2638	0.8025	0.0814	0.9541	0.5002	0.6769	0.2334	0.8878	-0.0325	NA	0.1251	0.8963	0.0458	0.9885	0.3751	NA	0.5607	0.6939	2.7804	0.0002	-0.1794	0.8652	-0.2620	0.8694	0.0211	NA
AT5G38710	-1.5752	0.0011	-0.4272	0.4996	-0.3270	0.6710	-0.0646	0.9526	-0.4972	0.5425	0.2385	0.7189	-1.2597	0.0075	-0.7324	0.3202	0.1982	0.7542	-0.2617	0.8183	-2.5770	0.0000	-1.8393	0.0001	-0.7376	0.2784	0.0396	0.9451
AT5G38900	-1.8608	0.0028	-2.5390	0.0000	-1.6545	0.0206	-0.8829	0.3431	-1.1886	0.1413	-0.8612	0.2482	-1.9906	0.0008	-2.7442	0.0000	-1.7261	0.0073	-1.2877	0.1198	-2.3614	0.0004	-1.7408	0.0057	-1.9015	0.0047	-0.9001	0.1451
AT5G38980	-0.8071	0.1819	-2.0286	0.0002	-1.5019	0.0172	-1.2738	0.0837	-0.6596	0.4501	-1.8895	0.0018	-0.7413	0.2092	-1.2750	0.0585	-2.2734	0.0000	-0.7893	0.3649	0.3591	0.7165	-0.5883	0.3707	-1.0118	0.1569	-0.8169	0.1360
AT5G39120	1.8770	0.0109	-0.0833	0.9457	-0.0615	0.9688	-0.1133	0.9458	0.5928	0.6661	-0.2124	NA	0.2236	0.8189	0.5306	0.7551	0.8855	0.2843	2.3324	0.0046	0.1266	0.9410	1.2986	0.0991	0.5466	0.6999	1.8612	0.0085
AT5G39240	0.2943	0.7411	1.6155	0.0204	1.0814	0.2183	0.5873	0.5900	2.0722	0.0025	0.9669	0.2244	0.6371	0.3846	1.4992	0.0586	1.4285	0.0398	1.7118	0.0290	0.2687	0.8447	1.2791	0.0655	2.2922	0.0006	0.6564	0.3441
AT5G39410	1.8255	0.0000	1.8999	0.0000	1.6736	0.0004	1.5074	0.0054	2.2740	0.0000	1.5894	0.0013	2.3417	0.0000	2.1909	0.0000	2.1238	0.0000	2.1601	0.0000	0.0569	0.9598	2.1626	0.0000	2.5131	0.0000	2.4899	0.0000
AT5G39520	-0.2206	0.8020	0.9788	0.1961	0.6493	0.4949	0.4121	0.7113	1.1878	0.1543	1.0867	0.1459	-0.2548	0.7564	0.7357	0.5203	1.7772	0.0066	1.8160	0.0150	-0.5074	0.6399	0.7825	0.2866	1.2817	0.1038	3.4889	0.0000
AT5G39580	-1.3777	0.0520	-0.6254	0.4786	0.3164	0.7831	-0.3339	0.7898	-1.5655	0.0555	0.7762	0.3505	-2.3324	0.0003	-0.7752	0.5258	0.1725	0.8533	-0.5573	0.6827	-1.4692	0.0910	-2.0882	0.0021	-1.6668	0.0332	-0.5316	0.4548
AT5G39610	-2.5563	0.0000	-0.2349	0.7368	0.5757	0.3993	-0.0093	0.9942	0.6066	0.4077	0.8684	0.1173	-1.3531	0.0034	0.0727	0.9678	-0.1373	0.8327	-0.4404	0.6337	-2.2728	0.0000	-2.1658	0.0000	0.7932	0.2162	1.1238	0.0150
AT5G39650	-1.8261	0.0147	-1.1407	0.1911	-0.6484	0.5634	-1.3077	0.2118	-1.2557	0.2081	0.2323	NA	-2.2052	0.0018	-0.5993	0.7084	-0.1862	NA	-1.2929	0.2183	-0.5589	0.6572	-1.8229	0.0160	-0.6769	0.6066	0.1816	NA
AT5G39660	-2.7015	0.0000	-1.9637	0.0000	-1.3140	0.0112	-1.3371	0.0200	-2.0101	0.0000	-0.8343	0.1197	-3.0358	0.0000	-1.6191	0.0009	-1.4602	0.0018	-0.9737	0.1088	-0.2481	0.7729	-2.9509	0.0000	-2.5989	0.0000	-1.0448	0.0194
AT5G39820	-2.0239	0.0062	-0.6424	0.5046	-1.1446	0.2484	-0.4806	0.7056	-1.4764	0.1134	-0.9090	NA	-1.6459	0.0240	-1.4796	0.1227	-1.7959	NA	-0.7694	0.5665	-0.8411	0.4540	-1.3096	0.0991	-0.5758	0.6821	-1.0896	NA
AT5G40060	-1.4661	0.0010	-1.0238	0.0368	-0.7908	0.1799	-1.1923	0.0383	-0.7133	0.2576	-1.6500	0.0008	-1.2314	0.0048	-0.9694	0.0867	-2.0011	0.0000	-0.3226	0.7343	0.0884	0.9322	-1.0428	0.0259	-0.6174	0.3550	-1.7416	0.0001
AT5G40230	-0.7128	0.2428	-1.7850	0.0023	-1.1498	0.1012	-1.4347	0.0469	-0.4037	0.7057	-1.5532	0.0161	-1.1158	0.0424	-1.1113	0.1266	-2.3746	0.0001	-1.0109	0.1988	-0.4273	0.6497	-1.3144	0.0230	-0.6792	0.4374	-0.9313	0.0945
AT5G40990	-1.9446	0.0092	-2.3549	0.0014	-2.2942	0.0040	-0.4872	0.7044	-1.6361	0.0678	-1.3155	NA	-2.0633	0.0040	-1.9102	0.0252	-2.2847	0.0021	-1.4958	0.1301	-1.6276	0.0810	-1.9061	0.0116	-2.0819	0.0091	-0.4330	0.5873
AT5G41180	-0.8733	0.0207	-1.3412	0.0003	-0.7514	0.1040	-0.9606	0.0429	-0.4821	0.3908	-0.7910	0.0620	-1.2555	0.0003	-0.9263	0.0331	-1.3892	0.0002	-0.8108	0.0899	-0.4848	0.3720	-1.0513	0.0047	-1.0848	0.0073	-2.0608	0.0000
AT5G41680	-1.8269	0.0142	-1.7896	0.0225	-0.3986	0.7408	-0.2675	0.8502	-1.2266	0.2196	0.2498	NA	-1.5906	0.0279	-1.1039	0.3197	-0.4840	0.5880	-0.9203	0.4498	-0.7594	0.5046	-2.1153	0.0041	-1.4810	0.1012	-1.3972	0.0499
AT5G41690	-0.4688	0.2622	-1.4540	0.0003	-1.1094	0.0137	0.0656	0.9356	-1.5143	0.0003	0.0036	0.9965	-2.2468	0.0000	0.0083	0.9957	-0.2809	0.5409	0.1770	0.8435	0.4312	0.4402	0.3019	0.5059	0.6620	0.1650	-0.3466	0.3886
AT5G41730	-3.2383	0.0000	-3.4096	0.0000	-3.3972	0.0000	-2.1916	0.0158	-3.3699	0.0000	-2.4976	NA	-4.0256	0.0000	-3.0845	0.0000	-3.8709	0.0000	-2.5993	0.0013	-2.5381	0.0016	-3.5485	0.0000	-2.6255	0.0005	-3.3902	0.0000
AT5G41740	-1.2839	0.0483	-3.8945	0.0000	-3.1682	0.0000	-1.4832	0.0720	-2.7831	0.0000	-1.7598	0.0117	-3.3672	0.0000	-2.1336	0.0013	-2.8465	0.0000	-1.1401	0.1942	0.6781	0.4815	-1.9582	0.0016	-2.5269	0.0001	-2.4535	0.0000
AT5G41750	-2.3048	0.0000	-4.7319	0.0000	-4.1412	0.0000	-1.3428	0.0811	-3.5819	0.0000	-1.4550	0.0258	-4.5789	0.0000	-2.7568	0.0000	-2.7333	0.0000	-2.1926	0.0005	-0.1800	0.8853	-3.2546	0.0000	-2.3948	0.0000	-2.1549	0.0001
AT5G41820	-0.9760	0.2004	-2.4587	0.0006	-2.3982	0.0018	-0.7054	0.5236	-2.6168	0.0003	-1.2351	NA	-2.5383	0.0001	-0.6407	0.6500	-1.5596	0.0378	-0.8787	0.4502	0.0265	0.9899	-0.8843	0.2641	-0.7909	0.4880	-1.6598	0.0166
AT5G41910	-0.1135	0.7852	-1.7888	0.0000	-1.7164	0.0000	0.3139	0.5146	-1.9327	0.0000	1.0734	0.0010	-2.4449	0.0000	0.2603	0.6847	0.7185	0.0233	0.6029	0.1370	0.1827	0.7450	-0.1038	0.8160	0.1975	0.7558	0.4532	0.1394
AT5G42010	-2.0185	0.0000	-1.9602	0.0001	-0.6474	0.3475	-0.7763	0.2790	-0.7206	0.3128	-0.7734	0.1857	-1.8252	0.0001	-1.3435	0.0171	-1.0239	0.0518	-0.6881	0.3775	-0.8551	0.2064	-1.8145	0.0002	-1.1085	0.0594	-1.2681	0.0089
AT5G42070	-0.0312	0.9536	-0.0625	0.9109	-0.4927	0.3059	-0.4218	0.4359	-0.3584	0.5419	-0.8783	0.0259	0.3342	0.3901	-0.4935	0.3718	-1.0962	0.0020	-0.6691	0.1621	0.2321	0.7070	0.1649	0.7315	-0.4382	0.4172	-2.0895	0.0000
AT5G42180	-0.5269	0.5537	0.7556	0.4046	1.6329	0.0526	-0.1628	0.9134	-0.5576	0.6880	-0.4054	NA	-1.4097	0.0545	0.4402	0.8040	-1.2716	0.1087	0.2561	0.8970	2.2606	0.0040	-0.5846	0.5179	0.5318	0.7037	1.2490	0.0730
AT5G42250	-0.0357	0.9646	-4.5027	0.0000	-3.0569	0.0000	-1.3173	0.0464	-1.8742	0.0004	-1.7007	0.0026	-3.6023	0.0000	-1.1778	0.0601	-1.8645	0.0003	-1.3560	0.0286	-1.4575	0.0154	-0.1935	0.7908	0.2121	0.8578	-0.8902	0.0773
AT5G42380	-3.0426	0.0000	-2.4238	0.0002	-2.0644	0.0040	-1.2528	0.1702	-2.2803	0.0008	-2.3562	0.0010	-3.4309	0.0000	-3.1298	0.0000	-2.8311	0.0000	-1.9509	0.0104	-0.7800	0.4290	-3.4845	0.0000	-2.5906	0.0001	-2.2491	0.0004
AT5G42530	-1.4396	0.0445	-2.8964	0.0000	-2.3075	0.0017	-1.7712	0.0460	-1.0285	0.2998	-1.9213	0.0124	-1.4047	0.0425	-1.4929	0.0838	-2.6396	0.0001	-1.6522	0.0556	-0.3413	0.7976	-1.4507	0.0460	-1.5124	0.0655	0.0454	0.9563
AT5G42680	-0.9994	0.0942	-2.5607	0.0000	-2.0433	0.0005	-0.9924	0.2159	-0.7981	0.3344	-1.6268	0.0099	-2.6390	0.0000	-0.2024	0.9021	-2.0717	0.0003	-0.8365	0.3358	-0.7552	0.3603	-1.0975	0.0670	-1.3349	0.0415	-1.5632	0.0043
AT5G42760	-1.8725	0.0068	-2.1949	0.0016	-2.4968	0.0005	-1.5648	0.0865	-2.4283	0.0006	-2.6823	0.0003	-1.7354	0.0095	-2.2973	0.0018	-2.4905	0.0003	0.0027	1.0000	0.2215	0.8799	-1.3051	0.0744	-2.5102	0.0004	-3.0139	0.0000
AT5G42830	-2.9089	0.0000	-2.4298	0.0000	-1.5411	0.0154	-1.4561	0.0429	-0.7644	0.3610	-1.5901	0.0112	-3.1362	0.0000	-2.5191	0.0000	-2.5522	0.0000	-2.3472	0.0001	-1.9237	0.0018	-3.4116	0.0000	-2.2925	0.0001	-1.8677	0.0006
AT5G42900	0.3654	0.6941	1.6223	0.0353	1.7757	0.0318	1.5655	0.1005	1.7911	0.0285	1.9748	0.0126	1.0206	0.1725	2.6218	0.0004	3.1381	0.0000	3.8091	0.0000	0.8017	0.4614	1.0810	0.1696	2.2936	0.0022	3.2409	0.0000
AT5G43040	0.6881	0.3029	-2.6400	0.0001	-2.5832	0.0002	-0.2459	0.8309	-2.6060	0.0001	0.3023	0.7162	-3.1193	0.0000	0.0135	0.9957	-0.4573	0.5412	0.0187	0.9925	1.1616	0.1375	0.5280	0.4674	0.6261	0.5381	-0.1253	0.8620
AT5G43050	0.1168	0.7329	-3.1317	0.0000	-3.7088	0.0000	-0.0336	0.9552	-3.2792	0.0000	-0.2287	0.5010	-2.6873	0.0000	-0.1075	0.8829	-0.3292	0.2710	-0.2847	0.5483	-0.1239	0.8085	0.2512	0.4181	-0.2589	0.5756	-0.5385	0.0420
AT5G43070	1.1722	0.0479	-2.0078	0.0008	-1.6336	0.0149	0.3244	0.7527	-1.2301	0.0915	-0.7493	0.2891	-1.1952	0.0385	0.4414	0.7296	-1.1222	0.0743	-0.2210	0.8871	0.9142	0.2512	1.3612	0.0206	-0.0311	0.9878	-0.7707	0.1881
AT5G43170	0.7121	0.2382	-2.0600	0.0005	-1.4267	0.0366	-0.5004	0.5847	-0.8754	0.2771	-0.8285	0.2256	-0.3157	0.6372	-0.0800	0.9688	-1.3395	0.0286	-0.3598	0.7712	0.6154	0.4639	0.3502	0.6259	-0.2818	0.8217	-0.2655	0.6704
AT5G43240	-1.4021	0.0712	-1.8288	0.0197	-1.8144	0.0347	-0.3021	0.8295	-0.6869	0.5990	-1.5709	NA	-2.2812	0.0013	-0.0486	0.9885	-1.0939	0.1777	-0.4698	0.7737	-0.3980	0.7729	-1.3369	0.0927	-0.2151	0.9075	-1.6632	0.0190
AT5G43270	-0.3173	0.4866	-2.7152	0.0000	-2.6267	0.0000	-0.8788	0.0828	-1.7522	0.0000	-0.9269	0.0320	-2.7221	0.0000	-0.1303	0.9080	-0.6228	0.1340	-0.2537	0.7616	0.2632	0.6949	-0.5047	0.2425	-0.6329	0.2189	-0.8076	0.0300
AT5G43290	-2.0405	0.0006	-0.4409	0.5807	-0.3180	0.7451	-0.5687	0.5519	-0.2530	0.8504	-1.4353	0.0356	-2.0511	0.0003	-0.8291	0.3802	-0.7063	0.2931	-1.7141	0.0157	-1.9984	0.0035	-1.6797	0.0054	-0.0420	0.9828	-1.0817	0.0662
AT5G43350	-0.0250	0.9814	-0.1717	0.8755	-0.9410	0.3404	0.1792	0.8990	-0.3265	0.8332	-1.0629	0.2087	-0.9480	0.1972	0.2222	0.9141	0.2576	0.7780	0.2384	0.9021	2.1580	0.0044	0.5769	0.4951	0.2126	0.9016	0.2754	0.7221
AT5G43403	-0.2470	0.5623	-4.4040	0.0000	-4.3203	0.0000	0.3067	0.5814	-4.2165	0.0000	0.6467	0.1019	-5.0236	0.0000	0.3835	0.5317	0.7818	0.0279	0.6776	0.1349	0.1026	0.8932	0.1405	0.7702	0.4664	0.3618	1.1211	0.0006
AT5G43420	-0.0386	0.9666	-3.4263	0.0000	-2.7085	0.0000	-0.5810	0.5451	-3.2345	0.0000	-0.8325	0.2442	-2.3008	0.0000	-0.9384	0.2876	-1.4471	0.0216	0.0297	0.9860	-0.0295	0.9858	-0.6187	0.3786	-1.9057	0.0033	-1.0054	0.0879
AT5G43450	-1.7588	0.0000	-2.1370	0.0000	-1.8760	0.0000	0.0924	0.9115	-1.8896	0.0000	-0.1387	0.8093	-2.6925	0.0000	-0.9429	0.0510	-0.5803	0.1894	-0.4776	0.4877	-1.5567	0.0003	-1.6586	0.0000	-0.2872	0.7142	-0.5530	0.1656
AT5G43470	-0.5665	0.0723	-3.5769	0.0000	-2.8528	0.0000	-0.5828	0.1609	-2.7927	0.0000	-0.0367	0.9381	-3.2796	0.0000	-0.6971	0.0548	-0.3919	0.2419	-0.2208	0.7250	-0.1750	0.7496	-0.7693	0.0122	-0.3984	0.3766	-0.6483	0.0257
AT5G43530	1.2719	0.0803	1.3451	0.0831	1.0202	0.2841	0.8255	0.4463	1.7653	0.0248	-0.0352	0.9775	2.1236	0.0011	1.0949	0.2861	-0.3653	0.6900	0.5934	0.6686	0.8035	0.4510	1.2410	0.0941	0.4994	0.7190	-0.6442	0.3898
AT5G43540	-0.6041	0.4377	-0.0259	0.9824	-0.0805	0.9524	-0.6025	0.5756	-0.2933	0.8435	-0.3764	NA	-0.6530	0.3797	-0.1819	0.9291	-0.0849	0.9276	-0.2141	0.9086	0.7451	0.4645	-0.3384	0.7083	-0.3963	0.7689	2.1783	0.0008
AT5G43580	-0.3864	0.6292	-2.7978	0.0000	-3.3754	0.0000	-0.6728	0.5019	-2.6150	0.0000	-0.9232	0.2226	-3.4113	0.0000	-0.3392	0.8377	0.6213	0.3944	0.4005	0.7733	-0.9926	0.2652	0.0025	0.9995	-0.8035	0.4106	0.0759	0.9192
AT5G43620	-0.9027	0.0972	-1.2018	0.0334	-1.5438	0.0089	-0.0249	0.9856	-1.1289	0.0774	-0.3348	0.6181	-1.8946	0.0001	-0.4855	0.6337	-0.1854	0.7841	0.1149	0.9410	2.3998	0.0000	-1.2136	0.0232	-0.6828	0.3821	-0.4189	0.4367
AT5G43630	-2.4961	0.0000	-0.9000	0.1389	-1.0721	0.1020	-1.5523	0.0176	-2.1613	0.0000	-2.1966	0.0001	-2.7198	0.0000	-1.7387	0.0020	-2.0978	0.0001	-0.1880	0.8966	0.0432	0.9752	-1.4671	0.0057	-1.1873	0.0551	-1.8473	0.0002
AT5G43650	-1.6311	0.0315	-2.0562	0.0071	-1.7148	0.0506	0.3287	0.8121	-1.8665	0.0273	-1.6036	NA	-2.1666	0.0022	-1.8042	0.0385	-1.6707	0.0310	-0.3418	0.8479	-0.7609	0.5070	-1.8386	0.0150	-1.7601	0.0381	-1.5176	0.0333
AT5G43740	-1.5138	0.0014	-5.8458	0.0000	-6.0192	0.0000	-1.0047	0.1223	-5.7105	0.0000	-0.8647	0.1209	-6.2528	0.0000	-0.3179	0.7806	-0.9056	0.0777	0.2855	0.7920	0.0532	0.9641	-0.9365	0.0656	-0.1028	0.9332	-0.7321	0.1215
AT5G43750	-0.0292	0.9694	-1.0535	0.0502	-0.8592	0.1796	-0.7384	0.3068	-0.1703	0.8827	-1.0521	0.0596	-0.4773	0.3844	-0.1688	0.9073	-1.2927	0.0106	-1.0683	0.0923	-0.4186	0.6040	-0.0942	0.9018	-0.8913	0.1586	-3.1162	0.0000
AT5G43890	0.4087	0.6303	-1.8295	0.0099	-0.2184	0.8546	-0.4374	0.7072	-0.2858	0.8515	-0.7835	0.3411	-0.7400	0.3135	0.4127	0.8038	-2.1505	0.0018	-0.6643	0.5970	1.6729	0.0380	0.0936	0.9309	-0.5290	0.6790	-0.6144	0.3746
AT5G44060	-0.9158	0.0539	-3.0356	0.0000	-2.6206	0.0000	-0.9468	0.1265	-2.0491	0.0000	-1.7032	0.0006	-2.5997	0.0000	-0.9586	0.0985	-2.0463	0.0000	-1.3148	0.0142	0.2793	0.7339	-1.2843	0.0053	-0.9129	0.1077	-1.2637	0.0045
AT5G44063	-1.1233	0.1079	-3.8650	0.0000	-3.8247	0.0000	-1.2245	0.1833	-3.8407	0.0000	-2.3592	0.0013	-4.2597	0.0000	-0.6431	0.6165	-1.8204	0.0079	-0.8815	0.4065	0.3624	0.7636	-1.0349	0.1504	-1.2230	0.1481	-1.3138	0.0417
AT5G44120	0.0862	0.9324	2.2131	0.0014	1.4405	0.0878	-0.0338	0.9855	-0.1057	0.9563	0.0602	0.9564	0.0156	0.9873	0.0319	0.9924	-0.0451	0.9654	-0.0217	0.9922	3.7784	0.0000	0.2686	0.7809	0.1591	0.9268	0.0673	0.9342
AT5G44130	-0.2480	0.6750	-2.0019	0.0000	-0.4950	0.4623	-1.3690	0.0157	-1.1888	0.0225	-1.2141	0.0176	-1.0362	0.0221	-0.7779	0.2322	-1.8150	0.0001	-1.6137	0.0014	-0.8138	0.1908	-0.8597	0.0792	-1.2230	0.0162	-1.0502	0.0179
AT5G44210	-2.1269	0.0000	0.3111	0.5807	0.5908	0.3006	-0.1845	0.8171	0.6690	0.2440	0.2621	0.6347	-1.4045	0.0005	0.2865	0.7697	0.2815	0.5819	-0.1358	0.9088	-1.6379	0.0006	-1.8017	0.0000	0.7405	0.1702	0.6816	0.0956
AT5G44260	-1.4850	0.0141	-0.5150	0.5086	0.1096	0.9253	-0.0356	0.9808	-0.1942	0.8923	0.1803	0.8373	-2.1910	0.0001	0.0508	0.9828	-0.4452	0.5336	-0.6605	0.5318	-1.8054	0.0077	-1.7062	0.0044	0.9264	0.2603	0.7539	0.2053
AT5G44310	-0.0772	0.9412	2.5126	0.0002	0.9422	0.3266	-0.1419	0.9228	0.6915	0.5580	0.0100	0.9940	0.4928	0.5475	0.0627	0.9805	-0.0094	0.9936	-0.1227	0.9561	2.0575	0.0100	1.7072	0.0170	0.0002	1.0000	0.0563	0.9447
AT5G44390	-1.7634	0.0119	-2.7533	0.0000	-1.6746	0.0398	-1.1192	0.2632	-0.8897	0.3993	-2.6604	0.0004	-2.7089	0.0000	-1.4424	0.1019	-3.1200	0.0000	-0.7684	0.5305	-0.6925	0.5237	-1.6225	0.0234	-1.4784	0.0759	-2.4905	0.0002
AT5G44420	-2.8391	0.0001	-2.9549	0.0000	-2.5049	0.0014	-0.6139	0.6167	-1.6539	0.0645	-2.5138	0.0018	-1.9575	0.0065	-2.4071	0.0025	-2.5739	0.0005	-0.8383	0.5191	-2.0201	0.0203	-1.6452	0.0331	-1.5621	0.0807	-0.5590	0.4712
AT5G44430	-2.2358	0.0022	-2.1373	0.0047	-2.1285	0.0094	0.4341	0.7406	-1.4130	0.1362	-1.8468	0.0253	-1.6608	0.0225	-1.7079	0.0548	-1.6782	0.0297	-0.9083	0.4637	-1.3482	0.1736	-1.1993	0.1363	-1.1226	0.2738	0.1229	0.8863
AT5G44440	-0.9715	0.2321	2.1123	0.0055	1.1811	0.2280	-0.3063	0.8264	-0.0482	0.9845	-0.6894	0.4584	-1.0350	0.1833	0.7066	0.6294	-0.5654	0.5197	-0.9861	0.4072	0.6042	0.6224	-0.7348	0.4051	0.6564	0.6214	0.1798	0.8316
AT5G44500	0.7462	0.0644	-2.2182	0.0000	-2.5532	0.0000	0.3806	0.5448	-2.0817	0.0000	-0.0621	0.9200	-1.5983	0.0000	0.4368	0.5338	-0.0793	0.8850	-0.0348	0.9786	0.8286	0.0937	0.6283	0.1365	0.0844	0.9333	-0.1418	0.7514
AT5G44563	0.0000	NA	1.1347	0.1765	2.3975	0.0017	0.5718	NA	2.1786	0.0046	0.2335	NA	0.8901	0.2487	0.3957	NA	0.2003	NA	0.3272	NA	0.3847	0.7674	0.0108	0.9937	0.6146	0.6328	0.5487	NA
AT5G44565	-0.8381	0.2691	-2.5203	0.0002	-1.8236	0.0197	-1.1598	0.2351	-1.1301	0.2258	-1.4398	0.0653	-2.0988	0.0013	-0.3813	0.8283	-1.8801	0.0077	-0.0007	1.0000	-0.1622	0.9157	-0.7808	0.3242	-0.4593	0.7357	-0.2539	0.7375
AT5G44567	-1.2729	0.0454	-1.6284	0.0133	-0.9932	0.2242	-1.9929	0.0086	-0.2869	0.8314	-0.6379	0.4034	-0.8098	0.2056	-1.6997	0.0158	-2.0578	0.0014	-1.0515	0.2339	-2.0849	0.0034	-1.2831	0.0467	-1.2079	0.1212	0.1113	0.8749
AT5G44568	-1.2010	0.0576	-2.8330	0.0000	-1.8308	0.0062	-1.7599	0.0197	-2.6997	0.0000	-1.8359	0.0063	-1.9993	0.0005	-2.1147	0.0010	-2.6296	0.0000	-2.3159	0.0004	-0.6410	0.4946	-1.5173	0.0146	-2.0337	0.0014	-1.4797	0.0117
AT5G44569	0.0425	0.9665	-2.3806	0.0006	-2.3297	0.0019	0.0175	0.9926	-2.3501	0.0013	0.1352	0.8931	-2.8174	0.0000	-0.3179	0.8582	0.0079	0.9944	-0.3929	0.7928	0.1563	0.9154	0.4128	0.6219	-0.2546	0.8697	0.5167	0.4466
AT5G44570	-0.1074	0.9223	-2.0493	0.0069	-2.0258	0.0139	-0.6335	0.6043	-2.0352	0.0118	-0.6954	NA	-2.2783	0.0013	-1.4020	0.1559	-1.2148	0.1294	-0.9152	0.4625	-1.3748	0.1638	-0.1798	0.8740	-1.2823	0.1852	-0.7825	0.2951
AT5G44572	0.1331	0.8581	-4.8531	0.0000	-4.5606	0.0000	0.1444	0.8914	-4.6895	0.0000	0.5238	0.4191	-5.1205	0.0000	-0.6935	0.4175	0.4362	0.4770	0.6517	0.4497	-1.1805	0.0737	0.5694	0.3482	0.2175	0.8546	0.5778	0.2695
AT5G44574	-1.4609	0.0200	-3.0896	0.0000	-2.8565	0.0000	-1.1676	0.1671	-3.2602	0.0000	-1.1203	NA	-3.4342	0.0000	-1.1656	0.1511	-0.9711	0.1485	-0.3928	0.7650	-1.2075	0.1424	-0.7578	0.2679	-1.4616	0.0484	-0.4568	0.4771
AT5G44575	-1.3907	0.0216	-1.5222	0.0162	-1.2598	0.0852	-1.0269	0.2217	-0.7302	0.4244	-1.6645	0.0135	-1.4182	0.0139	-2.7054	0.0000	-1.2291	0.0520	-1.3120	0.0865	-2.2689	0.0006	-0.8009	0.2256	-1.0528	0.1749	-1.1805	0.0427

AT5G44578	0.8160	0.0494	-2.8506	0.0000	-2.9389	0.0000	0.1348	0.8705	-2.8047	0.0000	0.6513	0.1913	-3.4529	0.0000	0.6932	0.2238	0.7130	0.1123	1.3003	0.0034	0.9469	0.0609	1.1174	0.0047	0.4943	0.4691	0.4517	0.3067
AT5G44580	-1.9487	0.0006	-4.0758	0.0000	-3.5495	0.0000	-1.8381	0.0094	-2.5254	0.0000	-1.9766	0.0018	-4.3271	0.0000	-1.1704	0.1202	-2.9222	0.0000	-1.1696	0.1327	-0.4658	0.6321	-1.8052	0.0017	-0.4968	0.6418	-0.6347	0.2793
AT5G44582	-1.1951	0.0091	-1.7656	0.0001	-1.7894	0.0002	-1.0826	0.0688	-0.7707	0.2091	-0.8175	0.1241	-2.1085	0.0000	-0.8177	0.1871	-1.0406	0.0306	-0.6759	0.3289	-0.1735	0.8489	-1.0157	0.0307	-0.6100	0.3677	0.4031	0.3801
AT5G44720	0.3446	0.5992	-2.0183	0.0001	-1.3244	0.0301	-0.0935	0.9356	0.0461	0.9790	-0.9820	0.1062	-0.2030	0.7643	-0.1178	0.9465	-0.9634	0.0880	-0.3402	0.7689	0.0522	0.9684	0.4198	0.5225	-0.5577	0.5272	-0.8619	0.0947
AT5G44850	0.0000	1.0000	1.3717	0.0489	1.9016	0.0069	0.0000	1.0000	1.0934	0.2009	0.0000	NA	2.0448	0.0008	0.0000	1.0000	0.0000	1.0000	0.0000	1.0000	0.0676	0.9649	0.0000	1.0000	0.0000	1.0000	0.1068	0.8832
AT5G44910	-0.9429	0.0450	-1.3764	0.0036	0.0374	0.9693	-0.8456	0.1820	-0.8748	0.1376	-0.3600	0.5375	-2.1361	0.0000	-0.2970	0.7831	-1.1053	0.0217	-0.6032	0.4117	-0.6337	0.3360	-0.8021	0.0994	-0.5200	0.4792	-0.3830	0.4178
AT5G44973	-1.5713	0.0245	-2.8255	0.0000	-2.5697	0.0004	-0.0751	0.9606	-2.3704	0.0010	1.3905	0.0695	-3.4298	0.0000	-0.4562	0.7773	0.5221	0.5151	0.1828	0.9262	-1.5768	0.0652	-1.1490	0.1194	-0.2878	0.8517	0.0097	0.9915
AT5G44980	-0.6812	0.3903	-2.7725	0.0001	-2.5768	0.0005	-0.1962	0.8877	-2.7499	0.0001	-0.5570	0.5276	-3.0202	0.0000	-0.2819	0.8825	-0.7505	0.3418	-0.0503	0.9813	0.1885	0.9016	-0.3053	0.7483	-0.2803	0.8586	0.3624	0.6250
AT5G44990	-2.4051	0.0006	-2.1713	0.0030	-1.6511	0.0530	-0.4379	0.7241	-1.3218	0.1555	-1.0395	0.2189	-1.6205	0.0203	-2.0366	0.0111	-1.8084	0.0151	-2.0165	0.0166	-2.1347	0.0097	-2.4051	0.0006	-1.8719	0.0197	-1.0379	0.1409
AT5G45000	-1.9350	0.0056	-2.8916	0.0000	-1.5460	0.0661	-1.7911	0.0450	-0.7936	0.4735	-1.4545	0.0673	-1.9756	0.0032	-1.9839	0.0111	-2.4641	0.0004	-1.7412	0.0410	-1.8075	0.0304	-1.9027	0.0068	-1.8843	0.0146	-1.9044	0.0048
AT5G45060	-1.1920	0.0017	-1.9673	0.0000	-1.6732	0.0000	-0.9117	0.0701	-1.8994	0.0000	-0.6139	0.1730	-2.2496	0.0000	-1.1178	0.0086	-1.0985	0.0050	-0.3536	0.6318	-0.3761	0.5342	-1.3477	0.0003	-1.3875	0.0004	-1.7072	0.0000
AT5G45080	-1.3830	0.0498	-3.1499	0.0000	-3.1049	0.0000	-1.1705	0.2222	-3.1229	0.0000	-2.3405	NA	-3.5262	0.0000	-1.5146	0.0714	-2.2516	0.0016	-2.0591	0.0111	-0.3653	0.7674	-1.7398	0.0131	-1.7349	0.0290	-1.8758	0.0058
AT5G45090	-1.7117	0.0195	-2.0917	0.0041	-2.0801	0.0083	-1.2332	0.2328	-2.0846	0.0068	-0.7604	NA	-2.2136	0.0013	-1.2668	0.2067	-1.3106	0.0889	-1.0355	0.3578	-1.2702	0.1924	-1.8530	0.0114	-1.3588	0.1330	-0.3844	0.6236
AT5G45113	0.2103	0.8379	3.2106	0.0000	2.6629	0.0003	-0.1180	0.9422	1.7981	0.0323	0.2560	NA	2.2154	0.0012	0.3963	0.8349	0.0000	1.0000	0.0000	1.0000	0.4236	0.7482	0.4151	0.6680	0.4657	0.7503	0.0000	1.0000
AT5G45115	-0.1252	0.8862	-2.9685	0.0000	-2.8922	0.0000	-0.5166	0.6070	-3.0927	0.0000	-0.6834	0.3731	-3.1786	0.0000	-0.5503	0.6619	-0.6779	0.3393	-0.3414	0.8090	-0.2870	0.8086	0.0798	0.9348	-0.8588	0.3519	-0.2666	0.6975
AT5G45220	-0.0644	0.9507	-2.7735	0.0001	-2.7352	0.0002	-0.1689	0.9043	-2.7504	0.0001	0.0772	0.9430	-3.1478	0.0000	0.0725	0.9773	-0.6038	0.4534	0.3727	0.8126	0.0411	0.9831	-0.5686	0.5007	-0.6123	0.6203	-0.6738	0.3380
AT5G45240	-0.3310	0.6981	-3.0417	0.0000	-2.9962	0.0000	-0.9550	0.3326	-3.0144	0.0000	-0.7081	0.3909	-3.4508	0.0000	-0.8692	0.4278	-1.3193	0.0672	-1.0229	0.3118	-1.2069	0.1862	-0.4739	0.5718	-0.8238	0.4293	0.3567	0.6159
AT5G45310	-0.0768	0.9299	2.5265	0.0000	2.1113	0.0005	1.2193	0.1228	0.4554	0.6812	1.1994	0.0719	0.5969	0.3554	0.6709	0.5214	1.8506	0.0016	1.3927	0.0553	-0.6141	0.5051	0.5517	0.4351	1.2975	0.0594	2.2706	0.0000
AT5G45340	0.2942	0.6977	-0.7799	0.2719	-0.6440	0.4499	-0.2329	0.8329	-0.4823	0.6552	-0.9248	0.1793	-1.0394	0.0793	-0.2139	0.9001	-0.5887	0.3828	-0.1255	0.9459	3.7350	0.0000	-0.2026	0.8098	-0.7881	0.3641	0.0253	0.9719
AT5G45380	-0.9607	0.2326	-1.9540	0.0104	-0.9630	0.3436	-0.9097	0.4180	0.0762	0.9733	-1.8207	0.0260	-0.8776	0.2650	-0.8511	0.5109	-2.4177	0.0010	-0.1500	0.9489	0.2797	0.8504	-0.9363	0.2595	-1.2927	0.1728	-0.6082	0.4207
AT5G45520	-1.2958	0.0286	-1.1486	0.0741	-1.0362	0.1651	-1.1844	0.1304	-0.2777	0.8275	-1.1687	0.0775	-1.0816	0.0627	-0.2711	0.8621	-0.9266	0.1396	-1.0121	0.2171	-1.3224	0.0665	-1.0924	0.0752	-0.9971	0.1899	-2.0110	0.0003
AT5G45750	2.0646	0.0000	0.8228	0.0092	1.0494	0.0010	1.0951	0.0017	1.4459	0.0000	1.3236	0.0000	1.6848	0.0000	1.0828	0.0006	0.8741	0.0046	0.6042	0.1379	0.1268	0.8364	1.5903	0.0000	1.2125	0.0001	0.6833	0.0205
AT5G45830	-0.5108	0.4966	0.9972	0.1735	0.8753	0.3090	0.3480	0.7579	-0.0622	0.9755	0.9618	0.1920	-0.5256	0.4638	-0.0280	0.9924	0.8119	0.2443	0.1940	0.9137	-2.6712	0.0001	-0.4250	0.5995	1.1083	0.1723	0.4642	0.4797
AT5G46115	-0.6455	0.4639	-1.6547	0.0391	-1.9635	0.0188	-0.9383	0.4084	-1.9738	0.0166	-1.0720	NA	-2.0995	0.0033	-0.2228	0.9214	-1.0149	0.2161	-0.2523	0.9032	0.0571	0.9775	-0.4406	0.6517	-0.8894	0.4448	-1.3158	0.0670
AT5G46220	0.0908	0.9148	-2.1811	0.0003	-1.1066	0.1492	-0.5778	0.5426	-0.8000	0.3671	-1.7114	0.0122	0.2342	0.7506	-0.3729	0.7934	-2.2117	0.0004	-0.7483	0.4456	0.2245	0.8496	0.6153	0.3676	-0.2276	0.8685	-1.5448	0.0092
AT5G46240	-1.3678	0.0176	-1.1738	0.0608	-0.1616	0.8756	-1.2502	0.0991	-0.3914	0.7259	-0.9205	0.1665	-1.4919	0.0067	-0.1633	0.9238	-2.0626	0.0003	-0.9531	0.2455	0.4123	0.6743	-1.3575	0.0201	0.4074	0.7123	-1.5698	0.0041
AT5G46250	-1.7551	0.0000	-1.9775	0.0000	-1.7955	0.0000	-1.0583	0.0172	-1.5995	0.0000	-0.4321	0.3203	-2.4857	0.0000	-0.9055	0.0302	-0.7604	0.0446	-0.4203	0.4957	-0.4429	0.4060	-1.6774	0.0000	-0.7034	0.1151	-0.7487	0.0308
AT5G46295	-1.7779	0.0183	-2.3054	0.0020	-2.2803	0.0044	-0.1331	0.9346	-2.2903	0.0034	-0.2981	NA	-2.5539	0.0003	-1.1683	0.2851	-1.3269	0.0954	-0.8462	0.5107	-0.0428	0.9840	-1.6500	0.0323	-1.4740	0.1066	0.0375	0.9670
AT5G46350	-1.8430	0.0005	-2.0510	0.0001	-1.5467	0.0104	-1.0143	0.1749	-1.8522	0.0009	-0.2348	0.7526	-2.5713	0.0000	-1.8386	0.0014	-1.0864	0.0574	-0.7880	0.3431	-1.9296	0.0010	-1.8445	0.0005	-1.5763	0.0071	-0.7566	0.1556
AT5G46440	0.2010	0.7851	-2.4758	0.0000	-1.3432	0.0436	-0.9779	0.2130	-0.4089	0.6992	-1.1757	0.0687	-0.1172	0.8721	-0.1475	0.9305	-1.1134	0.0623	-0.8576	0.3042	0.3170	0.7497	0.1043	0.9015	-1.2796	0.0546	-1.5004	0.0071
AT5G46490	-1.0182	0.0675	-5.2642	0.0000	-5.1405	0.0000	-0.9412	0.2124	-4.6999	0.0000	-0.8127	0.1958	-5.6477	0.0000	-1.6884	0.0034	-1.6501	0.0022	-0.4894	0.6331	-0.1307	0.9132	-1.3436	0.0135	-2.1555	0.0001	-1.4750	0.0041
AT5G46500	-1.7832	0.0087	-3.9428	0.0000	-3.5056	0.0000	-1.6193	0.0672	-3.5037	0.0000	-1.5570	0.0412	-3.8775	0.0000	-2.1309	0.0036	-2.4792	0.0002	-0.6113	0.6411	-0.4404	0.7130	-2.5098	0.0001	-2.5361	0.0002	-2.7498	0.0000
AT5G46510	-0.1760	0.7850	-6.2877	0.0000	-6.3160	0.0000	-0.7389	0.2868	-6.2355	0.0000	-0.7065	0.2108	-6.8776	0.0000	-0.1681	0.9038	-0.9041	0.0756	-0.4721	0.5932	0.2518	0.7768	-0.5527	0.3161	-0.3228	0.7339	-1.1889	0.0100
AT5G46520	-1.4248	0.0012	-5.2112	0.0000	-4.5932	0.0000	-1.4942	0.0051	-4.3919	0.0000	-0.4146	0.4585	-4.3891	0.0000	-0.6979	0.2846	-1.1339	0.0145	-0.6446	0.3474	-1.2235	0.0202	-1.2059	0.0072	-0.2051	0.8359	-0.8524	0.0501
AT5G46580	0.1955	0.7947	-0.7547	0.2562	-0.6767	0.3862	-0.4989	0.5796	-0.1961	0.8797	-1.1609	0.0639	0.1565	0.8310	0.0752	0.9693	-1.3448	0.0191	-0.3767	0.7505	-0.0009	1.0000	0.3798	0.5918	-0.4140	0.6966	-2.0101	0.0001
AT5G46600	1.6978	0.0134	0.4178	0.6664	1.2706	0.1473	0.8854	0.3968	1.4505	0.0840	0.7371	0.3883	2.0798	0.0012	2.0596	0.0055	-0.3526	0.6947	1.1701	0.2336	0.5807	0.6124	2.3124	0.0004	2.7690	0.0000	0.2143	0.7866
AT5G46871	-0.5721	0.4587	-1.1218	0.1412	-2.2456	0.0020	0.0775	0.9575	-2.3015	0.0011	0.3021	0.7350	-2.3724	0.0001	-0.2713	0.8810	0.5459	0.4734	0.1031	0.9616	-1.5767	0.0557	-0.4875	0.5526	-1.8457	0.0133	-0.7226	0.2791
AT5G47220	-1.6422	0.0004	-2.2634	0.0000	-1.3801	0.0093	-0.3227	0.6934	-0.5677	0.4464	-0.7206	0.2002	-1.1226	0.0162	-0.8661	0.1828	-1.5654	0.0011	-1.7380	0.0008	-0.4194	0.5876	-1.6554	0.0004	-0.8786	0.1482	-1.2986	0.0048
AT5G47260	0.0000	1.0000	4.5656	0.0000	5.0355	0.0000	-0.1732	0.9101	4.8237	0.0000	0.0000	1.0000	4.0836	0.0000	0.0000	1.0000	0.0000	1.0000	0.0000	1.0000	0.0000	1.0000	0.0713	0.9550	1.0817	0.2982	0.3340	0.6823
AT5G47450	-0.1058	0.9235	3.0053	0.0000	2.6921	0.0004	-0.0300	0.9888	0.7364	0.5605	0.2297	0.8328	1.4415	0.0507	1.7701	0.0426	1.3139	0.0979	0.9820	0.4117	-0.2195	0.8925	0.0209	0.9882	0.6668	0.6151	1.0379	0.1563
AT5G47560	-2.0383	0.0000	-0.3288	0.4911	-0.2588	0.6532	-0.5423	0.3169	-1.4826	0.0000	-0.3355	0.4705	-2.5808	0.0000	-1.2183	0.0019	-0.1437	0.7653	-0.5255	0.3595	-0.5541	0.2860	-1.6970	0.0000	-1.1423	0.0034	0.0346	0.9372
AT5G47590	-1.3781	0.0208	0.3138	0.7037	0.0430	0.9723	0.5727	0.5393	-0.5768	0.5629	1.8707	0.0030	-2.2117	0.0001	0.9312	0.2702	1.6247	0.0059	1.1660	0.1301	-0.3601	0.7314	-1.5287	0.0103	0.1243	0.9332	2.0710	0.0002
AT5G47610	-2.2118	0.0000	-1.6521	0.0010	-1.6687	0.0019	-0.8980	0.2043	-2.3629	0.0000	-1.2056	0.0326	-2.2243	0.0000	-2.0690	0.0000	-1.1574	0.0276	-0.6861	0.3906	0.7545	0.2848	-1.6038	0.0012	-1.1253	0.0563	0.1912	0.7309
AT5G47850	-1.6055	0.0280	-2.5053	0.0004	-1.4874	0.0911	-0.8495	0.4405	-0.8658	0.4331	-1.2582	0.1294	-1.7728	0.0106	-2.2258	0.0044	-2.6391	0.0002	-1.4347	0.1325	0.0930	0.9563	-2.4000	0.0006	-1.6661	0.0454	-0.3532	0.6488
AT5G47910	-1.0630	0.1054	-2.0264	0.0011	-1.2562	0.1023	-0.5562	0.5825	-0.8462	0.3573	-1.6219	0.0187	-0.8575	0.1893	-0.9906	0.2741	-2.2394	0.0003	-0.4483	0.7300	0.8370	0.3526	-1.0451	0.1193	-1.3986	0.0535	-1.5817	0.0080
AT5G47920	-1.9193	0.0013	-1.3089	0.0453	-1.8830	0.0052	-0.7488	0.4006	-1.1915	0.1153	-1.7044	0.0131	-2.4515	0.0000	-0.9493	0.2692	-1.9689	0.0017	-0.9254	0.2938	-0.8247	0.3351	-1.1613	0.0630	-0.6164	0.5361	-1.9780	0.0011
AT5G47960	-1.2094	0.0999	-2.0636	0.0040	-0.5825	0.5813	-1.1252	0.2615	-0.3206	0.8347	-0.9096	0.2761	-1.3550	0.0511	-1.0038	0.3517	-1.8782	0.0092	-0.5967	0.6611	0.3457	0.7922	-1.7044	0.0170	-1.5543	0.0594	-0.4243	0.5673
AT5G48000	1.0477	0.1766	-0.1216	0.9180	0.0148	0.9926	0.7123	0.5384	0.1337	0.9459	0.7730	0.3868	0.0570	0.9556	1.8562	0.0225	0.0781	0.9410	1.5832	0.0820	2.4323	0.0016	0.3278	0.7440	1.4308	0.1055	1.4549	0.0364
AT5G48010	1.9635	0.0081	1.0702	0.2244	1.2567	0.1931	0.5428	0.6624	1.2483	0.2100	0.1317	NA	0.6801	0.4130	0.8298	0.5325	0.3199	0.7367	0.9244	0.4503	2.5413	0.0016	0.6784	0.4489	1.1799	0.2386	2.1308	0.0026
AT5G48090	0.3786	0.6376	2.0749	0.0007	2.0214	0.0021	0.3325	0.7732	2.0097	0.0018	-0.7661	0.3557	2.1841	0.0001	0.1362	0.9504	0.0047	0.9972	-0.2077	0.9105	0.0827	0.9586	0.3883	0.6401	-0.1444	0.9316	-0.5660	0.4197
AT5G48110	-0.4883	0.5791	1.8604	0.0118	1.8802	0.0188	0.8791	0.4185	0.5417	0.6880	1.0619	0.2049	-0.2461	0.7912	1.6221	0.0574	1.5017	0.0438	2.1029	0.0094	0.5016	0.6863	-0.3413	0.7274	0.9861	0.3402	1.5147	0.0266
AT5G48250	0.7323	0.1714	1.1100	0.0379	1.4697	0.0068	0.4925	0.5371	2.2553	0.0000	1.1350	0.0420	1.5706	0.0007	1.7347	0.0008	1.8574	0.0001	2.3300	0.0000	0.2771	0.7633	1.3803	0.0052	1.2149	0.0307	1.5438	0.0011
AT5G48400	-2.1587	0.0006	-2.2529	0.0004	-1.8582	0.0098	-1.6513	0.0452	-1.1716	0.1649	-1.6271	0.0246	-2.3776	0.0001	-2.3166	0.0006	-1.9624	0.0028	-1.1627	0.1957	-2.1811	0.0022	-1.8834	0.0034	-1.8346	0.0091	-1.4715	0.0183
AT5G48430	-1.1800	0.0392	-1.9481	0.0005	-1.0546	0.1360	-1.5177	0.0306	-0.1026	0.9450	-2.0572	0.0008	-0.6770	0.2530	-1.2259	0.0738	-2.2502	0.0001	-1.7704	0.0053	0.1917	0.8676	-0.9739	0.1019	-1.5254	0.0135	-1.8502	0.0007
AT5G48470	0.2332	0.6985	-0.6098	0.2822	-0.8340	0.1663	-0.2748	0.7377	-0.5468	0.4555	-0.9024	0.0930	0.1095	0.8607	0.1546	0.9105	-0.8807	0.0772	-0.6852	0.3400	-0.4687	0.5195	0.4726	0.3901	-0.6353	0.3556	-2.1186	0.0000
AT5G48530	-2.0002	0.0000	-1.8816	0.0001	-2.1186	0.0000	-1.0622	0.1045	-1.6309	0.0014	-1.2352	0.0246	-2.2189	0.0000	-1.8276	0.0003	-1.4788	0.0030	-0.8811	0.1989	-0.4932	0.5186	-2.1298	0.0000	-2.2810	0.0000	-2.4512	0.0000
AT5G48540	-1.8041	0.0007	-2.0860	0.0001	-1.5991	0.0074	-1.0750	0.1452	-1.8013	0.0014	-1.6082	0.0072	-2.2404	0.0000	-2.2079	0.0001	-2.4827	0.0000	-1.2820	0.0573	-0.2598	0.8029	-1.9919	0.0001	-1.8277	0.0012	-1.8939	0.0003
AT5G48610	-1.1399	0.0263	-1.3989	0.0072	-0.9515	0.1360	-0.7297	0.3256	-1.0527	0.0895	-0.3353	0.6072	-2.1920	0.0000	-0.2151	0.8765	-0.4398	0.4542	-0.4175	0.6733	-0.3870	0.6483	-1.0184	0.0540	-0.3223	0.7512	-0.8737	0.0745
AT5G48620	-0.7818	0.0104	-2.0933	0.0000	-1.5532	0.0000	-0.5564	0.1886	-1.8429	0.0000	-0.0542	0.9072	-2.1513	0.0000	-0.6280	0.1028	-0.2343	0.5146	-0.0765	0.9321	-0.3676	0.4161	-0.8815	0.0035	-0.5795	0.1269	-0.5408	0.0664
AT5G48657	-2.3417	0.0000	-2.8767	0.0000	-1.7855	0.0020	-2.1614	0.0004	-1.4810	0.0135	-1.1896	0.0523	-3.2988	0.0000	-2.2371	0.0000	-2.5368	0.0000	-2.1114	0.0003	-2.0198	0.0005	-3.1314	0.0000	-2.3499	0.0000	-1.7267	0.0009
AT5G48660	0.1738	0.7778	-2.2023	0.0000	-2.1029	0.0000	0.0240	0.9844	-1.0953	0.0487	-0.2120	0.7385	-1.6955	0.0001	0.2011	0.8729	-0.1844	0.7591	0.2086	0.8508	0.6113	0.3560	0.1940	0.7606	-0.0153	0.9944	-0.4751	0.3151
AT5G48690	-0.7730	0.2045	-1.2174	0.0502	-2.1014	0.0006	-0.6068	0.4875	-0.9565	0.2095	-0.9889	0.1355	-1.7289	0.0012	0.0120	0.9957	-0.5247	0.4209	-0.3577	0.7694	-0.5317	0.5497	-0.7130	0.2584	-0.4640	0.6559	-1.7221	0.0024
AT5G48775	-1.5939	0.0345	-2.5114	0.0006	-2.4804	0.0014	-1.4583	0.1448	-2.4927	0.0010	-0.8409	NA	-2.8078	0.0000	-0.9442	0.4352	-0.5040	0.5703	-0.3166	0.8632	-1.3574	0.1672	-0.6781	0.4424	-0.5180	0.7161	-1.1789	0.1013
AT5G48780	-0.4812	0.4252	-3.2169	0.0000	-3.1490	0.0000	-0.1152	0.9124	-3.1762	0.0000	-0.1663	0.8276	-3.7457	0.0000	-0.0817	0.9650	0.5135	0.3768	0.4655	0.6275	0.5569	0.4672	-0.9537	0.0917	-0.3189	0.7712	-0.0998	0.8710
AT5G48900	-2.2125	0.0000	-0.1162	0.8436	0.4992	0.3519	-0.4070	0.5035	0.2244	0.7791	0.3441	0.4812	-1.9508	0.0000	0.0294	0.9860	-0.2906	0.5272	-0.7613	0.1412	-0.4606	0.4245	-2.0143	0.0000	0.2856	0.7010	0.0137	0.9770
AT5G48980	-0.9720	0.2253	-1.7083	0.0305	-1.6411	0.0629	-0.1731	0.9073	-1.4471	0.1204	-0.8140	NA	-2.0760	0.0033	-0.4902	0.7752	0.0289	0.9800	-0.2502	0.9017	0.3831	0.7745	-1.5143	0.0496	-0.1263	0.9467	-0.5796	0.4468
AT5G49015	0.5918	0.2958	0.8783	0.1289	1.5852	0.0034	0.8530	0.2364	2.3164	0.0000	1.5581	0.0047	0.9901	0.0483	1.5653	0.0040	1.6552	0.0010	1.2906	0.0335	-1.1536	0.0836	1.1081	0.0338	1.9950	0.0001	1.9131	0.0001
AT5G49030	0.0541	0.9309	-0.6338	0.2059	-0.4866	0.4235	-0.4158	0.5419	-0.5424	0.3993	-1.0204	0.0314	-0.0056	0.9931	-0.1460	0.9064	-1.0076	0.0222	-0.6299	0.3304	-0.1674	0.8439	0.1637	0.7833	-0.6565	0.2595	-2.1136	0.0000
AT5G49290	-0.0396	0.9584	-1.6802	0.0023	-1.8575	0.0017	-0.1644	0.8684	-1.1665	0.0687	-0.4421	0.5007	-2.0868	0.0000	0.2946	0.8112	-0.5744	0.3279	0.0973	0.9511	0.5806	0.4331	-0.0612	0.9400	-0.2864	0.7931	-0.2486	0.6592
AT5G49480	-2.0988	0.0002	-1.0402	0.1158	-1.2234	0.0857	-0.4520	0.6419	-1.6046	0.0122	-1.4042	0.0313	-2.0146	0.0002	-1.4287	0.0362	-0.8031	0.2104	-0.0651	0.9726	0.4798	0.6174	-1.8032	0.0017	-3.1149	0.0000	-2.2996	0.0000
AT5G49520	-1.7972	0.0000	-2.1156	0.0000	-1.2612	0.0070	-1.3993	0.0057	-1.6995	0.0001	-1.1041	0.0197	-2.7020	0.0000	-2.0603	0.0000	-1.2354	0.0042	-1.1702	0.0189	-0.9515	0.0722	-2.1564	0.0000	-1.7837	0.0000	-1.4110	0.0006
AT5G49525	1.7004	0.0195	0.1636	0.8888	0.7002	0.5193	1.3741	0.1679	1.5966	0.0657	1.7051	0.0349	1.8841	0.0062	1.1690	0.2640	1.4533	0.0573	1.4154	0.1435	0.0575	0.9771	2.3240	0.0009	2.5486	0.0005	2.1584	0.0017
AT5G49620	-2.0518	0.0055	-1.4368	0.0820	-1.1915	0.2233	-0.8183	0.4797	-0.6402	0.6327	-1.3876	NA	-1.8176	0.0117	-0.9371	0.4472	-0.8389	NA	-0.6249	0.6660	-1.2879	0.1995	-1.4942	0.0553	-1.2443	0.2047	-0.1937	NA
AT5G49680	-2.0452	0.0000	-2.2386	0.0000	-1.0850	0.0921	-1.6766	0.0082	-0.8564	0.2307	-1.2941	0.0277	-2.0451	0.0000	-1.4246	0.0159	-1.7139	0.0012	-1.4124	0.0227	-1.0316	0.1332	-2.0607	0.0000	-1.7819	0.0011	-2.0025	0.0001
AT5G49690	-2.0794	0.0000	-0.5964	0.2922	-1.4953	0.0045	-0.2937	0.7095	-0.6262	0.3662	-0.0954	0.8921	-2.9865	0.0000	-0.6094	0.4181	0.0489	0.9407	-0.5019	0.5418	-1.6994	0.0015	-1.6085	0.0005	-1.0921	0.0507	0.3308	0.4895
AT5G49700	-1.7053	0.0000	-1.7140	0.0000	-1.0113	0.0477	-0.9444	0.1012	-1.2542	0.0076	-0.8951	0.0690	-2.7145	0.0000	-0.7282	0.2335	-1.6543	0.0001	-1.8069	0.0001	-0.9799	0.0688	-1.8531	0.0000	-0.8239	0.1289	-0.3888	0.3795
AT5G49740	-2.1448	0.0000	-2.5183	0.0000	-1.8906	0.0000	-1.4634	0.0038	-1.3521	0.0028	-1.5008	0.0012	-1.9780	0.0000	-1.7070	0.0001	-2.2502	0.0000	-0.9967	0.0644	-0.8722	0.1160	-1.8305	0.0000	-1.6171	0.0002	-1.8878	0.0000
AT5G49850	2.0729	0.0029	0.5333	0.5842	0.7220	0.4989	0.2397	0.8676	2.2406	0.0025	0.4890	0.6048	1.1126	0.1270	1.8204	0.0249	0.0693	0.9483	2.7436	0.0002	2.2771	0.0037	1.6781	0.0207	1.1458	0.2400	1.3654	0.0493
AT5G49920	0.0000	NA	0.1503	0.8847	0.3416	NA	0.5625	NA	0.8747	0.3871	0.0000	NA	0.0753	NA	0.4779	NA	0.3605	NA	1.0841	NA	2.4209	0.0011	0.2363	NA	0.0000	NA	0.0000	NA
AT5G50200	-1.9628	0.0000	-1.3870	0.0000	-0.5903	0.1519	-0.6774	0.1180	-0.2094	0.7444	-1.0702	0.0023	-1.6188	0.0000	-1.3056	0.0001	-1.5278	0.0000	-1.2067	0.0005	-0.8496	0.0260	-2.0311	0.0000	-1.7326	0.0000	-0.7227	0.0203
AT5G50335	-3.9056	0.0000	-0.0247	0.9788	0.0595	0.9577	-0.8983	0.2535	0.9136	0.2150	-1.1722	0.0596	-2.4516	0.0000	-0.0961	0.9606	-1.1012	0.0578	-1.1832	0.0946	-0.9284	0.2152	-3.4058	0.0000	0.2736	0.8201	0.5316	0.3370
AT5G50360	0.2393	0.8133	2.1384	0.0047	0.7843	0.4687	0.8744	0.4457	0.6019	0.6594	0.6831	0.4619	0.4311	0.6294	0.3464	0.8622	1.2448	0.1177	1.4544	0.1420	0.7610	0.5058	2.5188	0.0005	0.9139	0.4217	0.9508	0.1941
AT5G50450	1.2313	0.0361	0.8442	0.2140	0.9351	0.2152	1.3646	0.0679	0.5887	0.5386	1.9182	0.0019	1.1183	0.0507	1.5551	0.0158	1.9079	0.0008	1.9254	0.0024	-0.9692	0.2195	1.3326	0.0237	1.4836	0.0197	2.1374	0.0001
AT5G50550	-0.4300	0.6477	-0.5855	0.5511	-0.9622	0.3496	0.3701	0.7850	2.2556	0.0044	0.1032	0.9295	-1.1140	0.1488	-0.3106	0.8803	0.7280	0.3962	-0.1153	0.9629	-0.6917	0.5561	-0.3041	0.7707	0.4810	0.7481	-0.2544	0.7578
AT5G50565	-0.6977	0.4221	-0.5895	0.5498	-0.6266	0.5802	-0.1197	0.9426	2.6668	0.0004	-0.2201	0.8404	-0.6455	0.4447	0.2095	0.9266	-0.0227	0.9853	0.0499	0.9826	-0.3828	0.7835	-0.1565	0.8912	-0.1977	0.9158	1.5810	0.0271
AT5G50570	0.0047	0.9979	-0.6381	0.5092	-0.8804	0.4064	-0.3429	0.8048	2.5665	0.0007	-0.4195	0.6712	-1.2519	0.0989	-0.1336	0.9593	-0.4936	0.5854	-0.4830	0.7668	0.2823	0.8509	-1.1434	0.1604	-0.4426	0.7743	1.1600	0.1092
AT5G50580	0.0341	0.9767	0.9695	0.2840	0.6767	0.5469	-0.3048	0.8284	2.2607	0.0043	0.6457	0.4940	0.2263	0.8185	-0.2376	0.9152	0.7246	0.3994	-0.1617	0.9450	-0.4569	0.7329	0.8734	0.3091	1.4505	0.1163	0.4646	0.5571
AT5G50790	-0.5101	0.4628	-0.4987	0.5220	-0.4194	0.6518	-0.6758	0.4622	-0.2404	0.8578	-1.0437	0.1502	0.2426	0.7324	-1.7785	0.0103	-2.0960	0.0014	-0.5967	0.5871	-1.0682	0.2012	-0.8170	0.2179	-1.2393	0.1095	-1.6079	0.0099
AT5G50915	-0.1619	0.7847	0.1531	0.8170	-1.0246	0.0509	-0.7558	0.2266	-1.5026	0.0011	-1.0482	0.0356	-1.2111	0.0041	-1.3208	0.0067	-0.4627	0.3593	-0.4349	0.5932	0.1436	0.8766	-0.5654	0.2545	-1.6737	0.0002	-2.3770	0.0000
AT5G51060	-1.7480	0.0164	-1.9267	0.0103	-1.1179	0.2437	1.3302	0.1811	-1.6499	0.0543	-1.2657	0.1285	-2.0798	0.0025	-1.7400	0.0403	-1.6097	0.0327	-1.3102	0.1921	-1.7209	0.0507	-1.9374	0.0076	-2.1871	0.0045	-2.0098	0.0038
AT5G51190	-0.3012	NA	0.0613	0.9538	-0.5059	0.5897	-0.1897	NA	-0.3268	0.8067	-0.2795	NA	-0.6068	0.3915	-0.3302	NA	-0.1273	NA	-0.5189	NA	2.0086	0.0060	-0.1270	0.8966	-0.2870	0.8348	-0.2639	NA
AT5G51465	-2.9483	0.0000	-4.3832	0.0000	-3.9590	0.0000	-2.2855	0.0096	-3.2922	0.0000	-1.8989	0.0190	-4.0067	0.0000	-2.9060	0.0001	-3.5636	0.0000	-2.6630	0.0007	-2.3877	0.0031	-3.4279	0.0000	-2.8891	0.0001	-1.4643	0.0371
AT5G51530	-1.0175	0.1062	-0.0598	0.9520	0.0127	0.9919	-1.1241	0.1750	1.0697	0.1530	-1.5235	0.0284	0.0638	0.9368	-1.1608	0.1402	-0.8803	0.1810	-0.2971	0.8308	-0.8529	0.3210	-1.1359	0.0727	-0.9147	0.2770	-2.0105	0.0010
AT5G51550	2.6478	0.0000	0.2779	0.5162	0.3837	0.4126	0.7456	0.0841	0.1541	0.8316	0.6568	0.0785	1.9373	0.0000	0.7132	0.0772	0.6187	0.0759	0.5255	0.2747	0.3058	0.5563	2.0728	0.0000	0.2461	0.6960	0.8425	0.0074
AT5G51620	-0.4108	0.1665	-6.2916	0.0000	-6.4217	0.0000	-0.4059	0.3150	-6.3451	0.0000	0.2986	0.3750	-6.8015	0.0000	-0.2990	0.5516	-0.0002	1.0000	-0.0339	0.9703	-0.3931	0.3210	-0.3413	0.2757	-0.4671	0.2053	-0.4988	0.0665
AT5G51630	-0.9367	0.0029	-5.8559	0.0000	-5.7708	0.0000	-0.6512	0.1263	-5.6699	0.0000	-0.8220	0.0225	-6.3716	0.0000	-0.7926	0.0322	-1.0708	0.0010	-0.6192	0.1462	-0.0701	0.9208	-0.8724	0.0061	-0.4442	0.3371	-0.7480	0.0167
AT5G51720	-1.9205	0.0001	-1.1347	0.0400	-1.3902	0.0157	-1.1438	0.0907	-1.6686	0.0017	-1.2094	0.0346	-1.9605	0.0000	-1.0753	0.0934	-1.4986	0.0038	-1.0587	0.1147	-0.3260	0.7187	-2.2226	0.0000	-0.6898	0.3591	-2.7360	0.0000
AT5G51780	1.1340	0.1140	1.4020	0.0596	2.5030	0.0002	1.0329	0.2971	0.3567	0.8119	1.0931	0.1729	-0.3704	0.6599	1.7476	0.0231	1.1021	0.1353	1.6873	0.0392	1.4068	0.1062	0.4787	0.5839	1.7304	0.0220	2.9261	0.0000
AT5G51990	0.2399	NA	0.8636	0.2551	0.0000	1.0000	0.2302	NA	0.2175	0.8810	0.1542	NA	-0.0064	0.9953	0.4647	NA	0.1458	NA	0.6109	NA	2.2070	0.0015	0.0504	0.9632	0.0000	1.0000	0.6656	NA
AT5G52050	-1.2771	0.0553	1.1931	0.0960	0.8796	0.3166	1.3546	0.1140	-0.6245	0.5716	1.2498	0.0867	-1.9129	0.0018	0.5221	0.7059	2.1413	0.0008	1.7107	0.0232	1.9502	0.0065	-1.8156	0.0050	-0.5910	0.6026	0.7787	0.2214
AT5G52250	-1.1077	0.0105	-1.6518	0.0001	-1.1013	0.0260	-0.4993	0.4549	-1.9999	0.0000	0.2730	0.6249	-2.0178	0.0000	-0.9074	0.0930	-0.3876	0.4395	-0.8047	0.1744	-1.1856	0.0186	-1.2331	0.0041	-0.8793	0.0941	0.0949	0.8475
AT5G52300	0.0709	0.9506	2.7140	0.0002	1.6318	0.0690	0.5000	0.6958	0.4011	0.8013	0.8521	0.3466	-0.1156	0.9099	0.4873	0.7845	1.3273	0.0960	0.8350	0.5219	0.7355	0.5288	2.7601	0.0001	1.5373	0.0877	1.5994	0.0255
AT5G52310	-0.4554	0.5785	2.1779	0.0010	1.9284	0.0088	1.1322	0.2328	1.6803	0.0268	2.1614	0.0028	-0.6023	0.4212	1.8510	0.0129	2.9766	0.0000	2.4535	0.0006	-0.6028	0.5711	0.9088	0.2222	3.6871	0.0000	2.7910	0.0000
AT5G52570	-1.6447	0.0000	-0.3530	0.4294	-0.5991	0.1930	-0.3033	0.5986	-1.8687	0.0000	-0.4762	0.2561	-1.9035	0.0000	-1.1835	0.0016	-0.4011	0.3132	-0.8155	0.0656	-0.6798	0.1430	-2.0336	0.0000	-0.7614	0.0694	-1.3462	0.0001
AT5G52640	-2.3432	0.0006	-2.3866	0.0006	-2.1090	0.0059	0.2107	0.8812	-1.4305	0.0999	-2.3889	0.0016	-2.8436	0.0000	-2.0824	0.0068	-2.4402	0.0005	0.0687	0.9765	-1.0805	0.2686	-2.1953	0.0015	-1.8380	0.0178	-2.3145	0.0005
AT5G52720	-2.2789	0.0018	-2.3107	0.0019	-1.7387	0.0463	-1.8161	0.0592	-0.1979	0.9151	-1.8066	NA	-2.1497	0.0025	-1.7325	0.0510	-2.4116	0.0011	-1.3961	0.1709	-1.4950	0.1198	-2.1437	0.0037	-1.6923	0.0493	-1.3324	0.0637
AT5G52730	-1.9636	0.0085	-1.7091	0.0325	-0.6895	0.5368	-1.6439	0.0967	-0.2184	0.9028	-1.0556	NA	-1.9545	0.0066	-1.6205	0.0774	-1.9597	0.0099	-1.1071	0.3303	-0.8422	0.4557	-2.1466	0.0038	-0.9621	0.3917	-0.8914	0.2295
AT5G52740	-1.5637	0.0414	-2.2200	0.0033	-1.5095	0.1013	-0.9543	0.3995	0.3719	0.8188	-1.3919	0.1036	-1.7664	0.0152	-1.1239	0.3173	-2.2447	0.0028	0.2874	0.8846	0.0354	0.9861	-1.7505	0.0222	-1.2364	0.2116	-0.9604	0.1936
AT5G52760	-2.4490	0.0005	-2.7275	0.0001	-1.8282	0.0278	-1.2960	0.2015	-0.9344	0.3916	-1.4743	0.0724	-2.2465	0.0011	-2.0991	0.0085	-2.6655	0.0002	-2.1372	0.0101	-1.1120	0.2700	-2.9243	0.0000	-1.9561	0.0135	-0.6788	0.3554
AT5G52810	-1.7728	0.0008	-2.1981	0.0000	-1.6792	0.0042	-1.3999	0.0425	-1.1553	0.0811	-2.3318	0.0000	-1.9855	0.0001	-2.0542	0.0002	-2.3934	0.0000	-1.9136	0.0013	-1.2381	0.0652	-2.0207	0.0001	-2.1077	0.0001	-2.5381	0.0000
AT5G52870	-2.0035	0.0003	-2.3907	0.0000	-2.1341	0.0003	-1.4389	0.0486	-1.5118	0.0177	-1.1325	0.0813	-2.7651	0.0000	-0.6747	0.4966	-1.5264	0.0089	-0.7839	0.3832	-0.8489	0.2904	-1.9611	0.0004	-1.3713	0.0353	-1.9845	0.0003
AT5G52890	-2.0708	0.0000	-0.1430	0.7229	-0.1577	0.7311	-0.5912	0.1178	-0.2255	0.6662	-0.1325	0.7308	-2.0108	0.0000	-0.7857	0.0129	-0.3531	0.2612	-1.0197	0.0010	-1.1334	0.0002	-1.8627	0.0000	-0.0828	0.9061	-1.0792	0.0001
AT5G52900	-2.1392	0.0000	-0.2416	0.6855	-0.2053	0.7721	-0.5891	0.3517	-0.2483	0.7772	0.1970	0.7350	-2.6584	0.0000	-0.0487	0.9748	-0.0730	0.9009	-0.4671	0.5333	-0.0888	0.9268	-2.1233	0.0000	0.3748	0.6231	-0.2617	0.5625
AT5G52940	0.2760	0.7420	-1.5600	0.0360	-1.6369	0.0448	0.6011	0.5527	-1.6602	0.0403	0.7782	0.3103	-2.2120	0.0006	0.2700	0.8777	0.2694	0.7477	0.1010	0.9623	-0.6248	0.5557	1.1110	0.0934	1.0486	0.2111	0.7683	0.2297
AT5G53030	-2.2146	0.0000	-1.5514	0.0035	-1.2531	0.0395	-1.5177	0.0181	-0.2418	0.8319	-1.9211	0.0007	-1.4542	0.0034	-1.1986	0.0548	-1.7263	0.0010	-0.8896	0.2272	-0.8700	0.2206	-1.4911	0.0040	-1.2525	0.0360	-1.2201	0.0154
AT5G53050	-0.9325	0.0143	-1.0780	0.0056	-1.0929	0.0094	-0.0781	0.9213	-1.6703	0.0000	0.1620	0.7552	-2.3607	0.0000	-0.5441	0.3510	0.4051	0.3434	0.1296	0.9016	-0.5628	0.2908	-1.0994	0.0034	-0.4663	0.4232	-0.5582	0.1331
AT5G53200	-3.3727	0.0000	-1.8662	0.0045	-2.5163	0.0001	-1.8695	0.0181	-2.3867	0.0002	-2.2248	0.0015	-3.1781	0.0000	-2.1556	0.0016	-3.0021	0.0000	-1.1451	0.1989	-0.7523	0.4296	-2.3959	0.0001	-2.8247	0.0000	-2.6804	0.0000
AT5G53250	-2.1296	0.0004	0.1677	0.8549	0.6690	0.4340	-0.9456	0.2804	0.2820	0.8297	-0.0454	0.9636	-0.8797	0.1568	0.0364	0.9893	-0.6517	0.3455	-0.4187	0.7425	-1.2130	0.1315	-2.3902	0.0001	0.7561	0.4006	0.6273	0.2959
AT5G53370	-0.7046	0.2495	-2.0576	0.0001	-1.3625	0.0343	-1.0974	0.1469	-0.9107	0.2287	-1.4597	0.0178	-1.0758	0.0528	-1.3474	0.0392	-1.7813	0.0014	-0.6968	0.4488	-0.4031	0.6733	-1.0030	0.0903	-1.6138	0.0069	-1.4018	0.0086
AT5G53390	1.2096	0.1174	0.4750	0.6316	0.5167	0.6458	-0.0866	0.9574	0.7758	0.5121	0.0000	NA	2.2537	0.0011	0.4601	0.7925	0.5487	NA	0.8801	0.4706	1.7774	0.0432	1.7612	0.0180	0.7720	0.5199	0.0000	NA
AT5G53950	1.6257	0.0243	1.2942	0.1129	-0.2752	0.8319	0.6790	0.5610	1.0229	0.3334	0.6333	NA	1.7196	0.0117	0.9933	0.3865	0.8884	0.2766	1.0311	0.3596	1.2756	0.1908	2.0315	0.0037	1.1689	0.2334	0.1188	0.8910
AT5G53970	-2.1830	0.0001	0.4558	0.5642	-0.1389	0.9012	-0.0717	0.9574	-1.6075	0.0144	0.6449	0.3744	-2.4190	0.0000	-0.3810	0.7899	0.8303	0.2022	0.5825	0.5972	-1.2732	0.0898	-1.8027	0.0021	-0.7776	0.3816	0.5624	0.3507
AT5G53980	-3.0063	0.0000	-0.2869	0.7590	0.5190	0.5910	-1.5333	0.0681	0.4840	0.6865	-1.0517	0.1621	-3.2719	0.0000	-0.5043	0.7198	-1.0598	0.1266	-0.9191	0.3556	-0.1646	0.9083	-2.5557	0.0000	-0.2906	0.8396	1.1170	0.0694
AT5G54060	1.0143	0.2118	-0.5282	0.5995	-0.5756	0.6147	0.9593	0.3954	-0.1341	0.9469	-0.7685	0.4007	0.7286	0.3773	0.2543	0.9073	-0.7339	0.3902	1.7381	0.0610	1.3551	0.1725	2.1226	0.0042	-0.4417	0.7741	-0.4197	0.5981
AT5G54130	-2.0521	0.0003	-1.5833	0.0091	-1.9523	0.0016	-1.3974	0.0669	-1.7063	0.0069	-1.8476	0.0038	-2.2186	0.0000	-1.8242	0.0040	-1.8806	0.0014	0.4646	0.6939	0.3341	0.7537	-2.1284	0.0002	-2.3509	0.0001	-2.6274	0.0000
AT5G54145	1.4654	0.0109	0.1420	0.8754	-0.0514	0.9666	0.6000	0.5089	-0.2893	0.8197	0.3680	0.6256	0.7989	0.1836	0.3092	0.8370	0.2073	0.7853	0.2031	0.8978	1.0230	0.1866	1.6523	0.0037	0.4733	0.6587	2.6005	0.0000
AT5G54148	2.0122	0.0012	-0.1387	0.8965	-0.0578	0.9680	0.6292	0.5465	-0.8801	0.4008	0.5541	0.5090	1.4943	0.0154	-0.0833	0.9726	-0.2393	0.7881	0.4617	0.7388	1.0093	0.2639	1.9825	0.0014	-0.3165	0.8353	0.5110	0.4606
AT5G54190	0.8233	0.2360	-1.6386	0.0144	1.8721	0.0069	0.7455	0.4474	2.9705	0.0000	0.5448	0.4972	-3.2486	0.0000	1.9138	0.0055	0.6847	0.3420	-0.1935	0.9145	-0.4420	0.6904	0.8837	0.2092	2.8476	0.0000	0.8102	0.1972
AT5G54240	-1.5240	0.0043	-1.1884	0.0409	-0.6616	0.3713	-0.9255	0.2130	-0.2213	0.8510	-1.6615	0.0069	-1.6035	0.0014	-0.3447	0.7797	-0.9740	0.0887	-0.3680	0.7382	0.5952	0.4400	-2.1382	0.0000	-0.0806	0.9523	-0.7839	0.1391
AT5G54300	-1.7501	0.0004	-1.0394	0.0618	-1.1799	0.0482	-0.7909	0.2803	-1.7116	0.0011	-0.2415	0.7268	-2.8458	0.0000	-1.3718	0.0162	-0.2468	0.6997	-0.2269	0.8541	-1.3693	0.0212	-1.9536	0.0001	-1.3580	0.0149	-0.8604	0.0807
AT5G54470	-3.3310	0.0000	-0.8477	0.2762	-0.9730	0.2589	-1.0410	0.2598	-0.2474	0.8664	-0.4247	0.6097	-3.7037	0.0000	-0.4268	0.7792	-0.7654	0.2883	-0.5075	0.6959	-0.5417	0.6070	-2.3265	0.0002	0.2203	0.8849	-0.5070	0.4458
AT5G54585	-2.7307	0.0000	-0.2920	0.6867	-0.4780	0.5363	-0.6659	0.3954	-0.6702	0.3897	0.1163	0.8816	-2.3798	0.0000	-0.6118	0.4965	-0.4578	0.4470	-0.4928	0.6127	-0.3760	0.6719	-1.2728	0.0156	0.9200	0.1646	-0.5100	0.3291
AT5G54610	-2.3770	0.0011	-2.6287	0.0003	-1.9835	0.0179	-1.6165	0.1019	-1.0199	0.3506	-2.7827	0.0004	-2.3186	0.0010	-2.3952	0.0025	-2.8052	0.0001	-1.3051	0.2120	-0.8749	0.4305	-2.0217	0.0066	-1.7814	0.0355	-2.1152	0.0029
AT5G54710	-0.8384	0.2453	-3.4598	0.0000	-1.6903	0.0238	-1.6463	0.0502	-1.1912	0.1645	-1.7516	0.0159	-2.1125	0.0006	-1.7802	0.0158	-2.9262	0.0000	-1.4480	0.0827	-0.2344	0.8626	-1.6682	0.0122	-2.1143	0.0020	-2.5458	0.0000
AT5G54720	-1.1608	0.1057	-4.4100	0.0000	-2.8904	0.0000	-1.8616	0.0288	-1.2471	0.1581	-1.7824	0.0180	-3.6888	0.0000	-1.8486	0.0155	-2.9397	0.0000	-1.5393	0.0729	-0.5687	0.6104	-1.9977	0.0031	-2.2571	0.0014	-2.8830	0.0000
AT5G55050	-1.0641	0.0620	-2.1124	0.0001	-1.5696	0.0102	-1.3424	0.0591	-0.8053	0.3044	-1.9554	0.0010	-1.5371	0.0035	-1.4850	0.0164	-2.3241	0.0000	-0.9690	0.2096	-0.4174	0.6528	-1.2614	0.0257	-1.6457	0.0051	-1.6888	0.0014
AT5G55150	0.0501	0.9580	-0.0992	0.9204	0.0644	0.9601	2.1594	0.0037	0.0750	0.9678	-0.0828	NA	-0.0456	0.9590	0.0838	0.9698	0.1465	0.8627	1.3737	0.0882	0.2635	0.8314	0.0997	0.9198	-0.0854	0.9587	0.7579	0.2205
AT5G55220	0.5400	0.2871	-0.1184	0.8667	-0.3494	0.6193	-0.1357	0.8819	-0.1617	0.8796	-0.4681	0.4064	0.5069	0.3054	0.3773	0.6980	-0.6997	0.1567	-0.4454	0.5995	-0.0860	0.9348	0.5185	0.3256	-0.4824	0.5254	-2.2814	0.0000
AT5G55340	2.1742	0.0021	0.7651	0.4042	1.1154	0.2490	1.1138	0.2887	1.7875	0.0306	0.2472	0.8128	2.3398	0.0005	1.6389	0.0595	0.0447	0.9685	0.6376	0.6510	1.7221	0.0503	2.4478	0.0004	1.0978	0.2757	-0.3597	0.6539
AT5G55380	-0.7218	0.1829	-1.3954	0.0084	-1.0349	0.0981	-1.0013	0.1424	-1.3406	0.0195	-1.7682	0.0018	-0.7827	0.1264	-1.4704	0.0091	-2.2145	0.0000	-0.5139	0.5715	0.4256	0.5981	-0.7158	0.1965	-1.0500	0.0889	-1.1390	0.0215
AT5G55460	-1.6253	0.0060	-2.0995	0.0004	-1.2390	0.0878	-2.1320	0.0022	0.5144	0.6195	-1.2447	0.0655	-1.7996	0.0013	-1.6907	0.0107	-2.5043	0.0000	-1.8291	0.0072	-0.9802	0.2254	-1.8006	0.0022	-0.9621	0.2263	-0.9075	0.1188
AT5G55570	0.1923	0.8082	-1.0300	0.1296	-0.3575	0.7040	-0.5556	0.5556	-0.1947	0.8881	-1.5795	0.0200	0.7490	0.2172	-0.9821	0.2424	-1.8917	0.0022	-1.5657	0.0301	-0.4175	0.6827	0.4351	0.5480	-0.9610	0.2331	-3.0195	0.0000
AT5G55620	-1.7972	0.0023	-1.3980	0.0285	-0.9277	0.2428	-1.4584	0.0592	-0.6944	0.4584	-1.6615	0.0119	-1.5800	0.0060	-0.7152	0.4875	-2.1328	0.0003	-0.4035	0.7535	0.1083	0.9379	-1.2304	0.0488	-0.7313	0.4279	-1.2537	0.0290
AT5G55700	-0.1004	0.9065	1.4882	0.0159	1.2201	0.0905	1.3831	0.0722	0.0136	0.9968	2.1003	0.0009	-0.0016	0.9997	1.1725	0.1222	2.0373	0.0005	1.7273	0.0107	-0.6796	0.4425	-0.0814	0.9310	0.6945	0.4532	1.4906	0.0082
AT5G55930	0.4541	0.4966	-2.3367	0.0000	-1.0184	0.1551	-1.0551	0.1740	-0.1664	0.9021	-1.3100	0.0369	0.0931	0.9026	-0.8551	0.3087	-2.2195	0.0001	-0.5229	0.6235	-0.1402	0.9115	0.0218	0.9833	-1.7766	0.0026	-1.3405	0.0128
AT5G55970	-2.2078	0.0000	-0.0300	0.9674	0.3808	0.5578	0.2089	0.7907	-0.5792	0.3666	0.7771	0.1163	-2.0112	0.0000	0.1167	0.9296	0.1858	0.7374	0.1318	0.9141	-1.4224	0.0029	-1.7989	0.0000	0.5052	0.4565	1.2964	0.0016
AT5G56040	-1.1148	0.0685	-1.7498	0.0034	-0.6845	0.4144	-1.4938	0.0463	-0.1436	0.9232	-2.0581	0.0012	-0.5625	0.3889	-0.2770	0.8608	-2.3076	0.0001	-0.5601	0.6131	0.6276	0.4847	-0.7987	0.2234	-1.2632	0.0702	-1.1339	0.0453
AT5G56490	-0.0116	0.9902	-0.7185	0.2692	-1.0487	0.1262	-0.4506	0.6052	-0.0673	0.9658	-1.2071	0.0533	-0.1985	0.7681	-0.3877	0.7505	-1.4017	0.0141	-0.5560	0.5661	0.4059	0.6479	-0.4570	0.4814	-0.4566	0.6427	-2.0636	0.0002
AT5G56555	-2.2036	0.0026	-1.7433	0.0276	-0.8838	0.4001	-1.1416	0.2875	-0.6770	0.6024	0.0108	NA	-2.2170	0.0017	-0.9506	0.4354	-0.7266	0.3938	-0.3787	0.8263	-0.1531	0.9270	-1.8320	0.0153	-1.4044	0.1299	-0.3482	0.6658
AT5G56850	-1.3167	0.0512	-1.7321	0.0109	-1.3634	0.0881	-1.5676	0.0651	-1.1768	0.1706	-1.9793	0.0059	-1.2312	0.0606	-1.0424	0.2734	-2.6293	0.0000	-1.0196	0.2929	-0.5395	0.6165	-1.0986	0.1194	-1.4141	0.0675	-3.1051	0.0000
AT5G56960	-3.0560	0.0000	-3.4484	0.0000	-2.0065	0.0033	-1.1724	0.1789	-2.6491	0.0000	-1.5533	0.0282	-3.1823	0.0000	-2.6053	0.0001	-2.6853	0.0000	-2.0711	0.0034	-1.7926	0.0145	-3.6540	0.0000	-2.9484	0.0000	-2.8037	0.0000
AT5G56970	-1.8437	0.0087	0.8695	0.2862	0.4642	0.6604	-0.4929	0.6675	-0.7231	0.5244	0.4452	0.6121	-1.2897	0.0603	-0.2301	0.9086	-0.1447	0.8778	0.0507	0.9810	-1.3128	0.1585	-2.3902	0.0005	-1.6131	0.0498	-0.3487	0.6416
AT5G57010	-2.6031	0.0000	-2.4893	0.0001	-1.4395	0.0622	-0.8161	0.4017	-1.3991	0.0737	-1.3274	0.0700	-2.8422	0.0000	-2.0198	0.0039	-2.0517	0.0017	-1.7199	0.0246	-0.8948	0.3319	-2.5744	0.0000	-2.0251	0.0032	-1.4830	0.0173

AT5G57030	0.1260	0.8130	-0.5865	0.2017	-0.4895	0.3686	-0.2303	0.7363	-0.3479	0.6148	-1.1705	0.0063	0.0603	0.9098	-0.3424	0.6688	-1.0445	0.0088	-0.7643	0.1426	0.1020	0.9064	0.1077	0.8533	0.0928	0.9254	-2.1463	0.0000
AT5G57090	-0.5627	0.4980	1.6356	0.0225	1.5239	0.0591	1.0556	0.2826	-0.2319	0.8882	1.2398	0.1140	-0.7170	0.3442	1.0158	0.3221	1.1692	0.1093	1.0149	0.3270	1.4222	0.1001	0.2231	0.8232	1.1308	0.2117	2.2503	0.0005
AT5G57110	0.9011	0.0394	1.0204	0.0244	0.8567	0.1048	0.8085	0.1704	0.9555	0.0609	0.7221	0.1460	0.8262	0.0522	1.1960	0.0117	1.4856	0.0004	1.6037	0.0004	0.5995	0.3271	1.5610	0.0001	2.0614	0.0000	1.5315	0.0001
AT5G57220	-1.7884	0.0000	-2.7271	0.0000	-0.7490	0.1916	-0.8158	0.1811	-1.5126	0.0008	-0.6132	0.2408	-2.2517	0.0000	-1.3326	0.0055	-1.5518	0.0004	-1.7907	0.0001	-0.9804	0.0746	-2.3812	0.0000	-1.2852	0.0066	-0.6157	0.1553
AT5G57240	-2.0957	0.0001	-0.1460	0.8618	0.0353	0.9759	-0.5105	0.5594	-0.5727	0.5211	0.0478	0.9563	-2.7157	0.0000	-1.0659	0.1334	-0.2018	0.7768	-0.4261	0.6974	-1.3064	0.0514	-2.3631	0.0000	0.4516	0.6502	0.3405	0.5538
AT5G57510	-2.5987	0.0001	-1.5427	0.0412	-1.9196	0.0152	-0.0290	0.9885	-1.9629	0.0109	-0.7824	0.3540	-2.2984	0.0005	-1.8793	0.0173	-1.0841	0.1502	-0.4271	0.7798	-1.7752	0.0347	-2.6818	0.0001	-2.0430	0.0073	-0.8176	0.2392
AT5G57520	-2.1654	0.0004	-1.3127	0.0468	-1.5930	0.0252	-0.5965	0.5092	-1.3580	0.0645	-0.8066	NA	-1.1966	0.0356	-0.9846	0.2348	-1.7519	0.0064	-1.5078	0.0448	-0.8676	0.3050	-1.4053	0.0220	-1.2720	0.0883	-0.2545	0.6950
AT5G57760	-1.6934	0.0122	-1.0465	0.1792	-0.4371	0.6746	-1.3795	0.1242	0.3275	0.8164	-1.5704	0.0406	-2.0846	0.0010	-0.2927	0.8694	-1.5597	0.0276	-1.9887	0.0118	-1.8793	0.0185	-1.5104	0.0285	-0.1877	0.9085	-1.0667	0.1062
AT5G57910	-2.0607	0.0000	-0.9444	0.0617	-1.2511	0.0158	-0.7001	0.2960	-1.1814	0.0233	0.2807	0.6412	-2.4629	0.0000	-0.9004	0.1318	-0.1207	0.8467	-0.1003	0.9440	-1.1123	0.0465	-2.0530	0.0000	-1.0046	0.0646	-0.5084	0.2701
AT5G58120	-1.3276	0.0152	-1.7420	0.0013	-1.2951	0.0412	-1.4386	0.0350	-1.1762	0.0737	-1.5691	0.0089	-1.5615	0.0026	-2.3204	0.0000	-2.3752	0.0000	-1.1950	0.0833	0.3158	0.7477	-2.1586	0.0000	-2.5748	0.0000	-2.3867	0.0000
AT5G58260	-0.0119	0.9868	-0.9163	0.0572	-0.7750	0.1718	-0.5457	0.4166	-0.3667	0.6486	-1.3073	0.0070	0.2142	0.6932	-0.4662	0.5653	-1.5682	0.0003	-0.9965	0.0725	0.3307	0.6570	0.1065	0.8730	-0.7088	0.2308	-2.2217	0.0000
AT5G58310	0.5586	0.4830	-1.0093	0.2058	-0.1003	0.9414	-0.4127	0.7254	-0.0437	0.9845	-1.7997	0.0173	1.0447	0.1299	0.0758	0.9752	-1.7694	0.0113	-0.6919	0.5710	0.8988	0.3555	0.3485	0.7006	-1.2639	0.1393	-2.3898	0.0003
AT5G58350	-2.1751	0.0000	-0.9374	0.0047	-0.8140	0.0293	-0.8049	0.0536	-1.1661	0.0004	-0.7185	0.0496	-2.3487	0.0000	-1.7601	0.0000	-0.8541	0.0102	-0.7100	0.0865	-1.3257	0.0001	-1.9004	0.0000	-1.2820	0.0001	-0.7247	0.0203
AT5G58600	1.7575	0.0000	0.4911	0.3518	0.5069	0.4021	1.3405	0.0082	1.3904	0.0014	1.3490	0.0033	1.1262	0.0052	2.1283	0.0000	1.6816	0.0000	1.6167	0.0003	0.1205	0.8988	1.8928	0.0000	2.1515	0.0000	1.6499	0.0000
AT5G58620	-1.8084	0.0000	-1.0488	0.0003	-0.8208	0.0122	-0.7515	0.0431	-0.4605	0.2549	-0.7333	0.0256	-2.1758	0.0000	-0.7438	0.0271	-0.6973	0.0221	-0.5871	0.1300	-0.2008	0.6943	-1.9258	0.0000	-0.6809	0.0449	-0.4855	0.0898
AT5G58760	-1.7359	0.0017	-1.6709	0.0035	-1.4827	0.0204	-1.3083	0.0762	-1.1919	0.0833	-1.7185	0.0057	-2.1221	0.0000	-1.1508	0.1073	-1.7487	0.0021	-0.1421	0.9311	-0.0647	0.9622	-1.0847	0.0672	-1.2280	0.0648	-1.9557	0.0003
AT5G58770	-2.0826	0.0007	-2.6743	0.0000	-3.0750	0.0000	-1.2174	0.1576	-3.1995	0.0000	-2.5061	0.0002	-2.0252	0.0006	-3.0468	0.0000	-2.4270	0.0001	-0.8115	0.4267	-1.0531	0.2201	-2.0568	0.0008	-2.3445	0.0002	-3.5598	0.0000
AT5G58860	2.1035	0.0006	0.3155	0.7316	0.0998	0.9377	0.3800	0.7328	0.3510	0.7948	1.1037	0.1315	0.8365	0.2079	1.5188	0.0401	0.8441	0.2275	1.9893	0.0048	1.6059	0.0325	1.4934	0.0204	1.1659	0.1455	2.2481	0.0002
AT5G58980	0.1829	0.6480	-2.4735	0.0000	-2.4189	0.0000	-0.0365	0.9594	-1.7117	0.0000	-0.2775	0.4974	-2.1108	0.0000	0.3006	0.6313	0.0705	0.8727	0.1593	0.8318	0.1852	0.7489	0.3237	0.3858	-0.0911	0.9098	-0.5033	0.1156
AT5G59130	0.3622	0.6915	-1.3527	0.0862	-0.5495	0.6133	-0.2098	0.8822	-0.0666	0.9772	-2.1045	0.0069	0.1112	0.9084	-0.4280	0.8071	-2.0597	0.0041	-0.2343	0.9059	0.7606	0.4809	1.1106	0.1484	0.8323	0.4533	-0.9972	0.1513
AT5G59320	1.2535	0.1055	1.2469	0.1381	1.3506	0.1487	0.0231	0.9912	1.9259	0.0191	-0.0057	0.9971	1.3021	0.0792	-0.1434	0.9551	0.5033	0.5714	0.3408	0.8477	1.2275	0.2208	4.5138	0.0000	0.2969	0.8595	0.5302	0.4891
AT5G59340	-2.0916	0.0008	-0.3157	0.7065	-0.3894	0.6797	-0.8260	0.3483	-2.0287	0.0031	-1.5606	NA	-1.7360	0.0027	-2.0389	0.0026	-1.2858	0.0519	-1.3587	0.0889	0.6152	0.4819	-2.5365	0.0000	-1.5996	0.0275	-1.5747	0.0117
AT5G59390	-2.1847	0.0016	0.0565	0.9596	-0.5340	0.6071	-0.8771	0.3910	0.2715	0.8573	-0.5281	NA	-1.0134	0.1427	-1.2053	0.2014	-0.0876	0.9259	0.3976	0.7875	-1.4288	0.1119	-1.4977	0.0364	-0.5584	0.6594	-0.6059	0.3901
AT5G59510	-2.4545	0.0004	-1.0271	0.2126	-1.0225	0.2781	-0.2966	0.8195	-1.6746	0.0418	-1.0279	0.2127	-2.6908	0.0000	-1.2898	0.1672	-0.8873	0.2512	-0.8698	0.4488	-0.6772	0.5342	-2.2445	0.0012	-1.4312	0.0946	-0.0444	0.9570
AT5G59530	-0.7938	0.2712	-1.6598	0.0193	-2.3936	0.0009	-0.6542	0.5264	-2.4375	0.0005	-0.7139	0.3765	-2.5546	0.0000	-1.0531	0.2693	-1.2609	0.0753	-0.0368	0.9841	-0.2511	0.8479	-0.5340	0.5031	-0.5646	0.6363	0.5919	0.3666
AT5G59570	0.1523	0.8679	2.0659	0.0009	2.0216	0.0024	1.4776	0.0717	1.5164	0.0379	1.8615	0.0070	0.5828	0.4071	2.0230	0.0024	2.9637	0.0000	2.8387	0.0000	0.7115	0.4614	0.3082	0.7226	1.2010	0.1297	2.6520	0.0000
AT5G59590	-2.1518	0.0007	0.4802	0.5583	0.4010	0.6835	0.2017	0.8698	-1.5797	0.0342	-0.1810	0.8445	-2.3221	0.0001	-0.4494	0.7547	0.4262	0.5702	-0.1953	0.9116	-0.0165	0.9948	-1.7777	0.0056	-0.3985	0.7550	0.8459	0.1656
AT5G59670	-1.9885	0.0058	-2.3015	0.0015	-1.1743	0.2171	-1.8242	0.0486	-0.2661	0.8737	-2.2713	0.0039	-0.9951	0.1904	-1.4081	0.1352	-2.8622	0.0001	-0.4527	0.7759	0.0256	0.9907	-1.7973	0.0146	-1.5693	0.0662	-2.0600	0.0029
AT5G59680	-1.7740	0.0053	-2.3898	0.0001	-1.4402	0.0588	-1.4064	0.0973	-0.7795	0.4278	-1.1317	0.1249	-1.9214	0.0016	-1.2349	0.1390	-2.5518	0.0001	-0.8707	0.3894	0.1762	0.9005	-1.8085	0.0046	-2.0626	0.0021	-1.8032	0.0033
AT5G59732	-1.8578	0.0088	-2.0643	0.0037	-2.0653	0.0071	-1.8428	0.0402	-2.2179	0.0024	-1.1796	NA	-1.9229	0.0050	-1.5712	0.0693	-1.8027	0.0124	-0.5966	0.6704	-1.8200	0.0288	-1.8593	0.0093	-1.4140	0.1012	-2.0059	0.0025
AT5G59750	-1.0354	0.0005	-0.9308	0.0026	-1.2373	0.0001	-0.9275	0.0124	-1.2740	0.0000	-1.5275	0.0000	-0.8572	0.0032	-1.0392	0.0013	-1.4462	0.0000	-0.7967	0.0289	-0.0551	0.9379	-1.0099	0.0007	-1.1758	0.0001	-2.0233	0.0000
AT5G59920	-0.8852	0.0849	-0.9375	0.0867	-0.8975	0.1515	-1.2640	0.0429	-1.4693	0.0052	-1.7502	0.0010	-1.8004	0.0001	-1.1715	0.0435	-1.6232	0.0010	-0.6462	0.4129	0.5883	0.4190	-0.8705	0.0961	-1.6249	0.0015	-2.1831	0.0000
AT5G59930	-0.0147	0.9910	1.2921	0.1216	0.8989	0.3835	0.8479	0.4567	0.3942	0.8003	1.4851	NA	0.0000	1.0000	1.4037	0.1459	1.8135	0.0168	1.6208	0.0827	0.2715	0.8537	0.0000	1.0000	1.2969	0.1702	2.0105	0.0042
AT5G59990	-2.2109	0.0000	-0.6714	0.2564	-1.4741	0.0071	-1.0074	0.1299	-0.1502	0.8983	-1.0818	0.0544	-2.0613	0.0000	-0.7393	0.3192	-1.0108	0.0531	-0.5437	0.5296	-1.2792	0.0310	-1.9305	0.0001	-1.1809	0.0389	-1.2923	0.0073
AT5G60100	1.0545	0.1474	3.2289	0.0000	3.4900	0.0000	2.0071	0.0158	3.8352	0.0000	2.5869	0.0003	2.9437	0.0000	2.7777	0.0000	3.2588	0.0000	3.4942	0.0000	0.5418	0.6338	2.3290	0.0004	3.3105	0.0000	3.3758	0.0000
AT5G60250	0.8569	0.1247	-1.9423	0.0015	-2.0033	0.0023	0.0374	0.9764	-0.4001	0.7095	-0.0477	0.9577	-0.5784	0.3253	-0.0374	0.9878	-0.0571	0.9427	0.4177	0.7001	0.6633	0.4002	0.5525	0.3747	-0.7257	0.4069	1.0673	0.0380
AT5G60440	-1.0440	0.0828	-1.9802	0.0010	-1.4494	0.0347	-1.0616	0.1845	-0.5682	0.5597	-0.1648	0.8444	-2.2860	0.0000	-0.3608	0.7916	-0.9313	0.1386	-0.1714	0.9154	-0.1364	0.9145	-1.0208	0.0957	-0.3569	0.7598	-0.4186	0.4886
AT5G60470	-1.8228	0.0119	-2.7198	0.0001	-2.2543	0.0041	-1.6489	0.0816	-1.1043	0.2712	-1.6870	NA	-2.0883	0.0023	-1.1981	0.2376	-1.9706	0.0078	-0.7059	0.5932	-0.5589	0.6411	-1.4133	0.0613	-0.8521	0.4491	-1.2950	0.0646
AT5G60780	1.7494	0.0016	1.2554	0.0405	0.0069	0.9961	0.9895	0.2166	1.7854	0.0026	0.1274	0.8855	1.9273	0.0003	0.6409	0.5305	0.2114	0.7810	0.4573	0.6926	2.0098	0.0009	1.6824	0.0025	0.0625	0.9712	-0.2582	0.6915
AT5G60800	-1.9171	0.0014	-2.0611	0.0007	-0.8756	0.2919	-1.4989	0.0578	-0.4587	0.6926	-1.0433	0.1411	-1.6509	0.0050	-1.3837	0.0613	-2.0961	0.0006	-1.6572	0.0212	-0.7971	0.3706	-1.8716	0.0019	-2.2819	0.0002	-2.2065	0.0001
AT5G60890	-0.0937	0.9063	-0.0545	0.9531	-0.8621	0.2388	0.3466	0.7144	-1.0636	0.1310	-0.4558	0.5135	-0.2044	0.7708	-0.0188	0.9936	0.3014	0.6593	1.1591	0.1010	2.4619	0.0000	0.6618	0.2882	0.0106	0.9984	-0.5130	0.3608
AT5G60900	-2.2623	0.0010	-2.5009	0.0003	-1.5186	0.0721	-1.7290	0.0555	-0.4996	0.7111	-1.7415	0.0258	-2.3513	0.0004	-1.9381	0.0132	-2.2198	0.0016	-0.8118	0.4977	-0.7697	0.4689	-2.2939	0.0008	-1.6495	0.0402	-1.5903	0.0180
AT5G60950	-2.1485	0.0000	-2.5834	0.0000	-1.4441	0.0127	-1.6241	0.0096	-0.6518	0.4068	-1.3432	0.0198	-2.0069	0.0000	-1.6646	0.0028	-2.1320	0.0000	-1.3671	0.0264	-0.8212	0.2503	-2.3669	0.0000	-1.5828	0.0042	-1.1052	0.0268
AT5G61070	-0.8414	0.1678	-0.7599	0.2681	-0.9760	0.1906	-1.0740	0.1720	0.2333	0.8545	-1.3871	0.0334	-0.6519	0.2795	-0.5711	0.5961	-2.1469	0.0003	-0.8260	0.3465	-0.8454	0.2942	-0.9382	0.1261	-0.8345	0.3036	-1.9425	0.0007
AT5G61160	-3.0984	0.0000	-3.2735	0.0000	-1.2422	0.1250	-0.2131	0.8676	-2.0722	0.0021	-1.3649	0.0611	-3.7521	0.0000	-2.1555	0.0016	-2.5781	0.0000	-1.1888	0.1824	-1.8906	0.0103	-2.9329	0.0000	-2.0030	0.0033	-1.3035	0.0357
AT5G61260	-1.4821	0.0014	-1.8758	0.0001	-0.7397	0.2283	-0.8571	0.1782	-0.9134	0.1243	-0.2405	0.6977	-2.0046	0.0000	-0.6215	0.3947	-0.5437	0.2957	-0.1632	0.8939	-1.0978	0.0609	-1.2810	0.0062	-0.7075	0.2775	-0.2727	0.5828
AT5G61290	3.2274	0.0000	0.6064	0.4223	0.3005	0.7649	0.8137	0.3526	1.1592	0.1183	0.3012	0.7169	2.0111	0.0002	1.1595	0.1251	0.2582	0.7425	0.9979	0.2364	1.3723	0.0559	3.2669	0.0000	1.5118	0.0204	1.1238	0.0510
AT5G61350	-2.3320	0.0007	-1.5263	0.0449	-1.6495	0.0459	-1.6878	0.0633	-1.9951	0.0095	-2.4690	0.0013	-0.7124	0.3487	-1.8257	0.0229	-2.1789	0.0023	-1.4680	0.1087	-0.5635	0.6243	-0.4768	0.5906	-1.6595	0.0407	-1.2262	0.0722
AT5G61380	0.3113	0.6286	0.8590	0.1431	0.7852	0.2496	0.6447	0.4068	0.8434	0.2232	1.0782	0.0620	0.1794	0.7867	1.1562	0.0612	1.7200	0.0007	2.0151	0.0002	-0.2491	0.7995	0.4289	0.4945	1.1807	0.0453	1.6214	0.0009
AT5G61610	-0.7197	0.2545	-2.8311	0.0000	-3.1961	0.0000	-0.7086	0.4185	-1.8430	0.0033	-0.1723	0.8374	-2.1767	0.0001	-0.5996	0.5743	-1.3399	0.0284	-0.7665	0.4072	-0.4532	0.6370	-0.9097	0.1457	-1.9093	0.0025	-1.2383	0.0294
AT5G61890	-1.9036	0.0005	-1.0954	0.0708	-0.6618	0.3864	-0.7281	0.3637	-0.8161	0.2889	-1.1008	0.0820	-2.7570	0.0000	-2.0184	0.0006	-0.5504	0.3753	-0.2019	0.8877	-1.5060	0.0219	-2.0515	0.0001	-1.2322	0.0594	-2.1113	0.0002
AT5G62020	-1.8840	0.0000	-0.5493	0.3575	-0.7836	0.2160	-0.2929	0.7268	-1.1877	0.0326	-0.1066	0.8840	-2.1256	0.0000	-1.0906	0.0613	-0.5759	0.2850	-0.3311	0.7454	0.5120	0.4852	-1.4741	0.0019	-0.5479	0.4778	0.1111	0.8398
AT5G62170	-2.1048	0.0000	-0.8022	0.0977	-0.9194	0.0797	-0.6880	0.2664	-0.9001	0.0902	-0.1751	0.7694	-2.1540	0.0000	-0.3820	0.6649	-0.4049	0.4171	-0.8957	0.1134	-0.8256	0.1465	-2.0857	0.0000	-0.3161	0.7041	-1.1242	0.0070
AT5G62210	2.0609	0.0015	-0.6263	0.4640	-0.8263	0.3792	0.7257	0.4832	0.0626	0.9772	-0.5960	0.4812	1.7628	0.0055	0.4326	0.7846	-0.8748	0.2381	-0.0938	0.9664	0.8418	0.3860	2.2562	0.0004	0.2813	0.8512	-1.6072	0.0138
AT5G62350	1.8519	0.0000	0.4649	0.1719	0.5066	0.1816	0.7786	0.0334	0.9022	0.0034	0.1327	0.7411	2.0664	0.0000	1.1010	0.0002	0.2199	0.5272	0.6403	0.0859	0.7501	0.0308	2.0523	0.0000	1.5191	0.0000	1.3161	0.0000
AT5G62360	2.6195	0.0000	-0.8075	0.2791	1.0810	0.1673	0.2560	0.8237	1.4285	0.0448	0.6903	0.3512	3.2906	0.0000	1.6861	0.0128	0.2365	0.7662	1.8936	0.0057	1.5985	0.0254	3.2290	0.0000	1.8280	0.0046	1.1687	0.0463
AT5G62430	-2.2017	0.0001	-0.4720	0.5300	-0.5288	0.5382	-0.8985	0.2784	-0.9558	0.2170	-0.9026	0.1786	-1.6534	0.0025	-0.7044	0.4699	-0.7745	0.2204	-0.3081	0.8179	0.1823	0.8811	-1.3422	0.0226	-1.0871	0.1299	-1.1597	0.0358
AT5G62520	-0.0933	0.9302	0.1930	0.8634	-0.5567	0.6226	1.1520	0.2639	-0.1269	0.9490	-0.1089	0.9232	-0.5964	0.4713	-0.3413	0.8621	0.1824	0.8539	0.6197	0.6600	2.3249	0.0033	-0.5223	0.5718	-0.5279	0.7103	-0.0161	0.9861
AT5G62720	1.1369	0.0521	1.3419	0.0263	0.9897	0.1774	1.6770	0.0158	0.7260	0.3979	1.4463	0.0217	1.3827	0.0121	1.6251	0.0095	2.1141	0.0002	1.4579	0.0322	-0.2251	0.8431	1.2991	0.0260	1.2678	0.0552	0.8488	0.1264
AT5G62730	2.2635	0.0020	0.0203	0.9876	0.2466	0.8513	0.8636	0.4555	0.0330	0.9913	-0.7192	0.4388	2.2239	0.0017	0.1076	0.9688	-0.7535	0.3779	0.1101	0.9646	0.8959	0.4199	2.0559	0.0059	-0.4948	0.7380	0.0256	0.9777
AT5G62920	1.4000	0.0373	2.1449	0.0010	1.7077	0.0220	1.6330	0.0526	-0.9510	0.3192	1.0671	0.1631	1.5521	0.0152	0.5549	0.6879	2.7033	0.0000	0.8971	0.3887	1.5802	0.0470	1.4387	0.0342	-0.7510	0.4791	-0.1499	0.8430
AT5G62980	0.5605	0.1437	-1.9076	0.0000	-2.5967	0.0000	0.6593	0.1856	-1.6424	0.0000	0.3964	0.3727	-1.7433	0.0000	0.6865	0.1448	0.5922	0.1276	0.6213	0.2223	0.4174	0.4429	0.9406	0.0088	0.1853	0.8164	0.3047	0.4192
AT5G63020	-0.6726	0.0794	-3.7655	0.0000	-3.2420	0.0000	-0.6654	0.1934	-3.0400	0.0000	-0.0734	0.8952	-4.1370	0.0000	-0.7612	0.1007	-0.3945	0.3443	-0.6478	0.2090	-0.7656	0.1026	-0.9893	0.0073	-0.6200	0.1996	-0.7154	0.0434
AT5G63060	0.1370	0.8040	-0.5289	0.2864	-0.1438	0.8395	-0.3843	0.5567	-0.1321	0.8917	-0.8925	0.0565	0.1961	0.6935	-0.5000	0.4710	-1.0420	0.0146	-1.1212	0.0209	-0.5739	0.3282	-0.2019	0.7136	-0.4987	0.4331	-2.0665	0.0000
AT5G63560	2.0487	0.0014	0.3897	0.6729	-0.3800	0.7293	0.3015	0.8074	0.4112	0.7593	1.0856	0.1577	0.3003	0.7145	1.5031	0.0566	1.0755	0.1299	2.5450	0.0002	1.6507	0.0352	1.3088	0.0574	1.0831	0.2186	2.1776	0.0005
AT5G63580	-0.3333	0.7049	-2.6719	0.0001	-2.4573	0.0009	-0.5390	0.6329	-2.3453	0.0013	-0.6603	0.4423	-2.4262	0.0002	-0.8331	0.4771	-2.1563	0.0024	0.4738	0.7394	-0.6855	0.5215	-0.8475	0.2754	-1.1114	0.2338	-0.4830	0.5025
AT5G63600	2.4050	0.0000	1.5529	0.0120	0.7770	0.3536	0.3112	0.7766	1.0971	0.1585	0.7166	0.3299	1.1696	0.0463	1.9121	0.0023	1.6786	0.0053	2.9158	0.0000	2.6674	0.0000	1.9771	0.0005	1.7121	0.0073	1.6423	0.0041
AT5G63625	-0.2090	0.7986	-1.8375	0.0068	-1.7339	0.0204	0.3920	0.6988	-2.2083	0.0015	0.9343	0.1773	-2.9082	0.0000	0.7551	0.4352	1.6821	0.0041	1.5285	0.0262	-0.6335	0.5148	0.0847	0.9292	0.5415	0.6099	0.6559	0.2766
AT5G63650	-2.2830	0.0000	-0.5972	0.2698	0.4801	0.4587	-0.9256	0.1254	0.4163	0.5914	-1.1989	0.0170	-2.8366	0.0000	-0.5131	0.5183	-1.0633	0.0234	-1.2736	0.0154	-1.8989	0.0001	-2.5677	0.0000	0.5976	0.3627	-0.8035	0.0666
AT5G63790	-1.7362	0.0000	-0.9469	0.0350	-0.7627	0.1519	0.0390	0.9669	-0.7973	0.1354	-0.2139	0.7037	-2.0449	0.0000	-0.5216	0.4569	-0.3055	0.5399	-0.3541	0.6643	-0.5258	0.3914	-1.4752	0.0003	-1.1855	0.0086	-1.0165	0.0113
AT5G63810	0.4010	0.5615	0.5569	0.4367	0.8636	0.2513	1.1464	0.1317	0.9216	0.2300	1.8420	0.0025	0.5090	0.4214	1.4040	0.0321	1.9037	0.0007	1.6659	0.0100	-1.0342	0.1699	0.6884	0.2846	2.4317	0.0000	2.2826	0.0000
AT5G63820	-0.5940	0.3870	0.0623	0.9500	0.6632	0.4334	1.4760	0.0467	0.6595	0.4829	2.1695	0.0005	-0.6875	0.2807	1.2060	0.1023	1.8946	0.0011	1.0915	0.1741	-0.7822	0.3773	-0.1053	0.9098	2.6433	0.0000	1.8357	0.0010
AT5G64170	-1.6348	0.0000	-0.7268	0.0970	-0.9577	0.0341	-0.8991	0.0800	-1.3115	0.0011	-1.0669	0.0135	-2.0932	0.0000	-1.1816	0.0055	-1.0152	0.0112	-0.1787	0.8499	-0.1843	0.8056	-1.2973	0.0006	-0.3622	0.5960	-1.3761	0.0002
AT5G64510	-1.9386	0.0030	-0.1130	0.9134	0.1678	0.8901	-0.3935	0.7307	0.7306	0.4823	-2.0923	0.0039	-0.6830	0.3393	-1.3400	0.1093	-1.0719	0.1300	0.1234	0.9532	-0.0419	0.9816	-1.4958	0.0272	-1.0836	0.2162	-0.7828	0.2319
AT5G64580	0.5357	0.4135	-0.4905	0.5036	-0.2543	0.7894	-0.3140	0.7562	-0.0038	1.0000	-1.2791	0.0444	0.6423	0.2929	0.3344	0.8127	-1.5152	0.0088	-0.5517	0.6001	0.0708	0.9587	0.6500	0.3193	-0.6737	0.4458	-2.2072	0.0000
AT5G64640	1.0688	0.0029	1.0521	0.0046	1.2331	0.0012	-0.1356	0.8502	1.8049	0.0000	-0.5894	0.1846	2.1900	0.0000	0.5247	0.3556	-0.6959	0.0845	-0.4727	0.4415	0.2910	0.6368	0.9173	0.0130	0.6745	0.1475	-0.3637	0.3439
AT5G64810	-2.9115	0.0000	-2.8705	0.0001	-1.8260	0.0330	-1.6917	0.0828	-0.7846	0.5171	-1.9333	0.0185	-2.3627	0.0007	-2.4044	0.0023	-2.8470	0.0001	-1.1677	0.2850	-1.3768	0.1615	-2.7287	0.0001	-1.9720	0.0155	-1.4855	0.0371
AT5G64840	-1.2086	0.0037	-0.5400	0.2906	-0.4123	0.5067	-1.1261	0.0325	-0.9276	0.0659	-0.9173	0.0526	-0.8746	0.0355	-1.5107	0.0005	-1.1118	0.0096	-0.7741	0.1826	-0.5745	0.3433	-1.2840	0.0019	-0.8469	0.0978	-2.1262	0.0000
AT5G64860	2.1728	0.0000	1.5944	0.0000	1.3376	0.0009	1.4587	0.0008	2.0824	0.0000	1.1855	0.0049	2.2083	0.0000	1.8878	0.0000	1.7388	0.0000	1.6281	0.0000	-0.0900	0.9182	2.3867	0.0000	2.2267	0.0000	1.3243	0.0003
AT5G64870	-0.5244	0.2445	-1.7652	0.0000	-1.6755	0.0002	0.0572	0.9498	-2.0306	0.0000	-0.4637	0.3645	-2.8512	0.0000	-0.9275	0.0688	-0.9282	0.0374	-0.1927	0.8451	1.0080	0.0343	-0.5913	0.1923	-0.7529	0.1690	-0.2371	0.5979
AT5G64890	-2.2581	0.0019	-2.5340	0.0004	-2.5100	0.0010	-1.1741	0.2756	-2.5196	0.0007	-1.2723	NA	-2.2883	0.0012	-2.0012	0.0164	-1.8901	NA	-1.6655	0.0763	-1.2804	0.2036	-2.2742	0.0018	-2.0205	0.0112	-0.9110	NA
AT5G64905	-2.5224	0.0004	-3.0486	0.0000	-2.6307	0.0006	-1.8789	0.0458	-2.4181	0.0016	-1.8918	NA	-2.6839	0.0001	-2.2670	0.0047	-1.1935	0.1327	-1.8632	0.0367	-2.2143	0.0081	-3.3182	0.0000	-2.4442	0.0015	-1.8519	0.0090
AT5G64940	-0.7344	0.1329	-1.1393	0.0183	-1.1349	0.0320	-0.7441	0.2584	-1.3253	0.0074	-0.8150	0.1230	-1.0283	0.0221	-0.7908	0.2154	-1.0446	0.0286	-0.5565	0.4683	0.1233	0.9037	-0.7868	0.1098	-1.1718	0.0217	-2.1036	0.0000
AT5G65040	-0.4942	0.5243	-1.9159	0.0040	-2.0849	0.0031	-0.7293	0.4682	-0.2384	0.8728	-0.9102	0.2363	-0.7389	0.2862	0.1294	0.9529	-1.2073	0.0806	1.3102	0.1194	1.4338	0.0712	-0.4840	0.5486	-1.0860	0.2062	-1.1479	0.0692
AT5G65070	-1.5404	0.0006	-2.9430	0.0000	-3.1693	0.0000	-0.3938	0.5957	-3.7328	0.0000	-2.2458	0.0000	-4.1066	0.0000	-0.7574	0.2427	-0.4146	0.4347	-0.8805	0.1543	-0.2053	0.8169	-1.6354	0.0002	-1.9484	0.0000	-2.0316	0.0000
AT5G65080	-2.8376	0.0000	-1.1219	0.0695	-1.9078	0.0012	-0.7940	0.3363	-2.6474	0.0000	-3.3138	0.0000	-3.4297	0.0000	-0.5301	0.6310	-0.8274	0.1743	-1.8370	0.0033	-0.6670	0.4238	-2.9245	0.0000	-3.1202	0.0000	-3.5869	0.0000
AT5G65090	0.2666	0.7859	0.1560	0.8929	1.2409	0.1879	0.5701	0.6359	2.0822	0.0074	-0.0028	0.9983	1.8350	0.0083	0.4104	0.8262	-0.7106	0.4036	0.9318	0.4300	0.6731	0.5598	0.4553	0.6301	-0.0954	0.9620	-0.2998	0.7129
AT5G65140	-1.0891	0.0228	0.4989	0.3963	0.1256	0.8840	0.1952	0.8248	0.0058	1.0000	-0.5852	0.3018	-0.9596	0.0392	-0.3620	0.7271	-0.5705	0.2784	0.2799	0.7897	0.6238	0.3584	-0.8312	0.0981	-0.4023	0.6389	-2.1972	0.0000
AT5G65350	0.1437	0.8759	-0.2085	0.8361	-1.3694	0.1156	0.3068	0.7920	-0.5872	0.6277	-0.7671	0.3596	0.8540	0.1837	-0.0933	0.9688	-0.1294	0.8875	0.4364	0.7501	2.0745	0.0018	1.1631	0.0750	0.2579	0.8649	-0.6582	0.3469
AT5G65790	1.0931	0.1318	-0.1819	0.8665	0.0947	0.9460	-0.1207	0.9369	0.5379	0.6783	0.2776	0.7750	-0.0426	0.9653	0.7357	0.5576	0.2306	0.8027	2.0505	0.0075	1.2500	0.1703	0.8576	0.2681	0.5072	0.7047	0.4256	0.5662
AT5G65850	-0.6190	0.3108	-3.3785	0.0000	-2.9886	0.0000	-0.4669	0.5984	-3.0136	0.0000	-0.6750	0.3184	-3.7534	0.0000	0.0755	0.9688	-0.6797	0.2750	0.0339	0.9833	0.6445	0.4151	-0.1444	0.8575	0.1220	0.9291	-0.5300	0.3539
AT5G65980	-0.2256	0.8226	0.7652	0.4081	-0.2712	0.8285	0.3022	0.8264	0.2714	0.8720	0.5436	NA	0.3414	0.7090	1.5040	0.1069	0.3305	NA	0.7503	0.5720	2.3482	0.0039	0.1525	0.8920	0.5221	0.7118	1.2118	NA
AT5G66052	0.9151	0.1714	1.3522	0.0442	1.2121	0.1179	1.3900	0.0899	0.1977	0.8935	1.4204	0.0413	1.3755	0.0239	1.1882	0.1447	1.8430	0.0032	2.2476	0.0008	2.0876	0.0020	1.1394	0.0839	1.0788	0.1810	3.2938	0.0000
AT5G66400	-0.4121	0.6560	0.8973	0.3141	0.1001	0.9457	0.5877	0.6251	-0.4128	0.7867	0.6558	0.4752	-0.6495	0.4280	0.2184	0.9213	1.6861	0.0261	0.9192	0.4432	0.4154	0.7538	3.6991	0.0000	1.5520	0.0732	1.3110	0.0627
AT5G66480	-0.9624	0.0048	-1.1082	0.0014	-0.9089	0.0207	-0.8591	0.0507	-1.6684	0.0000	-0.8486	0.0292	-2.0098	0.0000	-1.8787	0.0000	-1.0606	0.0025	-0.8808	0.0339	-0.2954	0.5945	-1.5145	0.0000	-1.2609	0.0005	-1.2546	0.0002
AT5G66580	-1.4025	0.0001	-0.3397	0.4717	-0.6753	0.1508	0.2129	0.7426	-0.9654	0.0199	0.8451	0.0391	-2.5884	0.0000	0.3492	0.6279	0.2140	0.6370	-0.0678	0.9542	1.2565	0.0015	-1.4306	0.0000	0.9332	0.0220	0.3153	0.4017
AT5G66620	-2.3372	0.0000	-1.9377	0.0003	-1.0083	0.1406	-1.4614	0.0310	-1.0047	0.1499	-1.1583	0.0583	-2.4939	0.0000	-1.9264	0.0007	-2.0116	0.0002	-0.8832	0.2595	-0.2501	0.8090	-3.3724	0.0000	-1.7717	0.0018	-1.7476	0.0008
AT5G66630	-1.2989	0.0029	-1.7649	0.0000	-0.7042	0.2252	-1.1308	0.0442	-0.5255	0.4412	-0.4285	0.4351	-1.9645	0.0000	-1.0548	0.0431	-1.4956	0.0008	-0.1704	0.8830	-0.2138	0.7988	-2.0138	0.0000	-1.1824	0.0159	-0.4020	0.3712
AT5G66640	-2.2457	0.0006	-2.1366	0.0015	-0.4656	0.6571	-1.6996	0.0479	0.1125	0.9513	-1.1336	0.1466	-1.8854	0.0034	-1.0268	0.3067	-2.6791	0.0001	-1.1010	0.2596	-0.5140	0.6504	-2.7944	0.0000	-1.7005	0.0239	-1.7320	0.0073
AT5G66780	0.3679	0.6938	2.4759	0.0005	0.4278	0.7114	0.1746	0.9067	0.0839	0.9691	0.0000	NA	0.5435	0.5186	0.1593	0.9468	0.9850	0.2153	0.7516	0.5643	1.9566	0.0212	2.2569	0.0016	0.7482	0.5350	0.7047	0.3313
AT5G66840	0.7110	0.2614	1.9320	0.0005	2.2429	0.0001	-0.1344	0.9104	2.2555	0.0000	-0.6521	0.3765	1.9912	0.0002	0.7641	0.4109	-0.4741	0.4997	-0.2668	0.8515	-0.2128	0.8606	0.8312	0.1877	0.2696	0.8364	-0.9275	0.1229
AT5G66970	0.0000	1.0000	3.2295	0.0000	3.5407	0.0000	-0.1652	0.9135	4.1378	0.0000	0.0000	NA	3.2161	0.0000	0.0000	1.0000	0.0000	1.0000	0.0000	1.0000	0.0000	1.0000	0.0000	1.0000	0.4923	0.7361	0.0000	1.0000
AT5G66980	-0.0065	0.9966	2.7696	0.0001	3.2103	0.0000	0.0324	0.9874	2.5833	0.0004	0.0000	NA	2.4221	0.0004	0.0000	1.0000	0.0000	1.0000	0.0000	1.0000	0.1757	0.9128	0.0000	1.0000	0.2320	0.8921	0.0000	1.0000
AT5G67080	-2.1944	0.0003	-1.5830	0.0156	-1.4208	0.0547	0.5849	0.5521	-2.2177	0.0006	0.0595	0.9522	-1.6591	0.0052	-1.0855	0.2034	-0.3199	0.6791	-0.8765	0.3613	-1.4002	0.0709	-2.0218	0.0009	-1.9458	0.0036	-0.3013	0.6530
AT5G67140	-2.0503	0.0000	-1.0620	0.0466	-0.9430	0.1272	-1.2531	0.0457	-0.7190	0.3044	-1.2893	0.0188	-2.1058	0.0000	-1.4640	0.0068	-1.4181	0.0047	-0.6271	0.4324	-0.2348	0.8019	-1.9947	0.0000	-1.0170	0.0873	-0.8839	0.0643
AT5G67310	-2.0427	0.0046	-0.9123	0.2979	-0.4754	0.6745	0.8175	0.4567	-0.5182	0.7061	-0.1114	0.9197	-1.6465	0.0189	-0.9815	0.3911	-0.3075	0.7384	-0.0448	0.9834	-1.1459	0.2500	-1.2052	0.1196	-1.4140	0.1147	0.3507	0.6502
AT5G67340	-2.1290	0.0000	-2.3662	0.0000	-1.1792	0.0319	-1.5280	0.0076	-0.8241	0.1925	-1.3386	0.0112	-2.3693	0.0000	-2.1549	0.0000	-2.4373	0.0000	-1.6818	0.0012	-1.3850	0.0111	-2.4437	0.0000	-2.0113	0.0000	-1.7931	0.0001
AT5G67370	-0.7281	0.2573	-1.3743	0.0270	-1.2001	0.0934	-0.9941	0.2296	-0.6174	0.5171	-1.6591	0.0100	-0.5260	0.4212	-1.3363	0.0567	-2.0263	0.0005	-0.6163	0.5555	0.1985	0.8697	0.0952	0.9176	-0.9379	0.2316	-2.0084	0.0003
AT5G67450	-1.2406	0.0478	-1.4162	0.0307	-1.0780	0.1705	-1.1508	0.1712	-0.8746	0.3183	-1.6654	0.0149	-1.6821	0.0039	-2.2198	0.0006	-1.8054	0.0039	-1.3568	0.0804	0.4394	0.6708	-1.7421	0.0043	-1.7007	0.0115	-1.2149	0.0402
AT5G67480	0.7907	0.1499	1.0277	0.0691	1.0337	0.1024	1.2496	0.0594	0.8035	0.2576	2.0654	0.0001	0.3642	0.5434	1.1528	0.0633	2.1793	0.0000	1.6770	0.0032	-1.6524	0.0038	0.4755	0.4417	0.4725	0.5998	0.9737	0.0486
ATCG00010	0.0928	0.9325	1.9655	0.0110	1.4538	0.1170	2.1519	0.0189	0.5048	0.7291	1.2099	0.1616	0.2270	0.8169	0.8244	0.5359	0.5492	0.5347	1.2293	0.2525	0.6037	0.6224	0.1077	0.9275	0.6891	0.5956	1.9278	0.0068
ATCG00030	-0.0830	0.9388	0.2499	0.8178	0.0848	0.9538	1.5419	0.1074	-0.0596	0.9805	0.3924	0.6830	-0.2779	0.7625	1.3770	0.1443	0.0475	0.9654	0.7884	0.5344	0.3284	0.8142	-0.1502	0.8918	-0.0067	0.9999	3.1417	0.0000
ATCG00050	-0.4900	0.5935	0.4144	0.6918	-0.2138	0.8733	0.3129	0.8234	-0.3596	0.8256	0.0729	0.9515	-0.8269	0.3054	0.5055	0.7718	-0.0014	1.0000	-0.1298	0.9567	0.0415	0.9842	-0.4961	0.6042	-0.1121	0.9534	2.0465	0.0040
ATCG00090	-0.3097	0.7557	0.5366	0.5940	0.4264	0.7244	1.8002	0.0631	0.0721	0.9762	0.5143	0.5932	-0.3081	0.7447	1.5095	0.1135	0.1528	0.8820	0.7074	0.6160	0.3336	0.8194	0.1688	0.8819	0.3952	0.8061	3.9655	0.0000
ATCG00100	-0.1758	0.8570	0.0691	0.9525	-0.0714	0.9601	0.5554	0.6212	-0.0952	0.9617	0.1451	0.8889	-0.6325	0.4079	0.9170	0.4039	0.2447	0.7852	0.2093	0.9141	0.4609	0.7010	0.0205	0.9870	-0.2393	0.8824	2.2374	0.0006
ATCG00160	-2.3360	0.0000	-0.7318	0.2100	-0.8721	0.1736	-0.8411	0.2286	-1.4359	0.0085	-0.6159	0.3014	-3.1035	0.0000	-0.2186	0.8700	-0.0605	0.9303	-0.6979	0.3628	-1.7399	0.0017	-2.4044	0.0000	-1.1935	0.0382	-0.6483	0.1886
ATCG00170	-2.0160	0.0002	-0.4250	0.5696	-0.3975	0.6486	-0.3001	0.7665	-1.5666	0.0110	-0.4236	0.5557	-2.5470	0.0000	-0.4223	0.7352	0.2712	0.7025	-0.2259	0.8771	-1.6759	0.0087	-1.8139	0.0010	-0.7801	0.3376	0.2030	0.7437
ATCG00240	-0.3824	0.6762	-0.1090	0.9241	0.5205	0.6390	1.8774	0.0378	-0.1719	0.9246	0.0931	0.9334	-0.3825	0.6613	0.9391	0.4223	-0.3016	0.7436	-0.2621	0.8921	-0.2628	0.8570	-0.4970	0.5857	-0.2008	0.9106	3.2117	0.0000
ATCG00280	-2.0487	0.0016	-0.9997	0.1976	-0.9918	0.2619	-0.7924	0.4347	-1.0375	0.2550	-0.9702	0.2113	-2.3338	0.0002	-0.5621	0.6830	-0.2014	0.8186	-1.1137	0.2372	-0.9791	0.2912	-1.8217	0.0057	-0.9967	0.2748	-0.7626	0.2458
ATCG00290	-2.1936	0.0028	-1.4445	0.0805	-1.4468	0.1196	-0.6302	0.6057	-1.6463	0.0648	-1.0702	0.2225	-2.3287	0.0009	-0.7167	0.6233	-0.0752	0.9445	-0.9271	0.4517	-1.5358	0.1065	-1.6837	0.0283	-1.6037	0.0677	0.0983	0.9103
ATCG00330	-2.6859	0.0000	-1.0354	0.1839	-0.9471	0.2953	-1.2671	0.1681	-1.0469	0.2557	-0.4122	0.6352	-2.7015	0.0000	-0.6227	0.6377	-0.3327	0.6916	-0.9211	0.3776	-1.2737	0.1462	-2.5230	0.0001	-1.3769	0.0866	-0.3292	0.6483
ATCG00340	-2.9525	0.0000	-1.4425	0.0392	-1.3298	0.0954	-1.9938	0.0116	-1.7276	0.0167	-0.9501	0.2138	-2.9928	0.0000	-1.7690	0.0151	-0.4179	0.5983	-1.1519	0.2054	-1.6159	0.0374	-2.9243	0.0000	-1.7014	0.0176	-1.7984	0.0038
ATCG00350	-2.8519	0.0000	-1.3777	0.0551	-1.3581	0.0916	-1.9231	0.0175	-1.7046	0.0211	-1.1487	0.1300	-2.9232	0.0000	-1.7651	0.0170	-0.4099	0.6110	-1.2290	0.1726	-1.4716	0.0715	-2.8201	0.0000	-1.6802	0.0217	-1.7190	0.0063
ATCG00360	-0.9277	0.2493	-0.3946	0.7031	-0.4004	0.7389	0.6159	0.6070	-0.8932	0.4287	-0.3240	0.7477	-1.3511	0.0662	0.2637	0.9020	-0.0227	0.9849	-0.4579	0.7758	-0.9769	0.3581	-0.8876	0.2879	-0.7698	0.5279	2.3081	0.0009
ATCG00380	-0.8141	0.2925	0.3260	0.7455	-0.1574	0.9038	0.9882	0.3322	-0.4831	0.7200	0.1847	0.8565	-0.8785	0.2317	0.7954	0.5149	-0.0105	0.9931	0.1054	0.9634	-0.3566	0.7830	-0.9973	0.1939	-0.4760	0.7277	2.3675	0.0003
ATCG00400	0.0148	0.9911	0.4436	0.6683	0.2438	0.8531	2.4692	0.0052	0.2204	0.9026	0.2885	0.7812	-0.2105	0.8314	2.6101	0.0008	0.2856	0.7690	0.3265	0.8603	0.5524	0.6641	0.0504	0.9693	0.1089	0.9551	2.6998	0.0001
ATCG00430	-0.4011	0.6700	-0.0620	0.9599	0.2454	0.8496	1.7413	0.0699	-0.3241	0.8448	-0.0460	0.9702	-0.6632	0.4227	2.2051	0.0062	0.0241	0.9836	-0.2276	0.9132	0.1026	0.9527	-0.3901	0.6939	-0.1078	0.9550	2.1671	0.0021
ATCG00440	-0.3537	0.7016	0.7486	0.4037	0.7335	0.4832	1.3769	0.1573	-0.2781	0.8643	0.7179	0.4156	-1.0726	0.1459	1.7101	0.0405	0.1162	0.9060	0.6547	0.6299	0.5356	0.6572	-0.5494	0.5365	0.1665	0.9264	2.3217	0.0006
ATCG00530	0.2372	0.7684	0.4678	0.5551	0.4457	0.6314	0.3988	0.6975	-0.0248	0.9917	0.5598	0.4567	0.0149	0.9863	0.8363	0.3750	0.4790	0.4978	0.5134	0.6611	0.4748	0.6346	0.2195	0.7959	0.2409	0.8611	2.0588	0.0003
ATCG00570	0.6031	0.4945	0.4971	0.6257	0.5143	0.6583	0.2231	0.8813	0.7862	0.5176	0.2178	NA	0.1737	0.8616	0.8686	0.5009	1.0514	0.1947	1.2476	0.2378	1.0014	0.3512	0.1183	0.9200	0.6113	0.6573	2.0687	0.0033
ATCG00600	-0.2334	0.8124	0.2261	0.8365	0.0869	0.9525	0.5088	0.6734	-0.0931	0.9650	0.5323	0.5630	-0.7443	0.3448	-0.0098	0.9968	0.4335	0.6235	0.0173	0.9945	-0.3478	0.8013	-0.6944	0.4208	0.2015	0.9110	2.0253	0.0028
ATCG00610	0.0364	0.9699	0.0808	0.9368	-0.0011	0.9999	1.4581	0.0787	-0.0531	0.9798	-0.1019	0.9153	-0.1048	0.9024	0.7606	0.4804	-0.0432	0.9630	0.0522	0.9791	-0.0471	0.9776	0.2004	0.8270	-0.0293	0.9893	2.4220	0.0000
ATCG00620	-0.2430	0.8113	0.2104	0.8541	-0.2783	0.8292	1.5976	0.1074	-0.4816	0.7444	-0.2455	0.8170	0.0026	0.9993	1.7368	0.0503	-0.5418	0.5409	-0.3737	0.8308	-0.2034	0.9018	0.0604	0.9628	-0.4398	0.7744	2.1795	0.0022
ATCG00630	-2.0314	0.0011	-0.6035	0.4582	-0.3921	0.7006	-1.3928	0.0997	0.0281	0.9913	0.3606	0.6686	-2.2097	0.0002	0.0324	0.9912	-0.3141	0.6961	-0.7858	0.4570	-0.5881	0.5603	-1.7548	0.0059	-0.7316	0.4703	-0.9284	0.1364
ATCG00650	-0.5132	0.5651	0.2159	0.8456	0.1922	0.8868	1.5256	0.1168	-0.3020	0.8553	0.2320	0.8250	-0.6623	0.4164	2.0270	0.0119	0.0720	0.9460	0.4085	0.8055	0.4864	0.7035	-0.3453	0.7289	-0.2253	0.8996	2.3569	0.0006
ATCG00700	-1.3317	0.0681	-0.7315	0.4056	-0.3672	0.7484	-0.6544	0.5571	-0.9184	0.3832	-0.4442	NA	-2.2105	0.0009	-0.9973	0.3581	-0.2887	0.7479	-0.8827	0.4426	-1.0725	0.2764	-1.4214	0.0536	-0.6901	0.5653	0.0875	0.9139
ATCG00740	-0.6283	0.4626	0.0816	0.9460	0.0101	0.9954	1.6176	0.0904	-0.4618	0.7493	0.2590	0.7992	-1.0029	0.1863	2.4847	0.0011	0.1289	0.8970	0.1222	0.9587	-0.0451	0.9822	-0.7835	0.3539	-0.1770	0.9227	2.0979	0.0024
ATCG00760	-0.8296	0.3164	-0.1412	0.9039	-0.3691	0.7626	1.8328	0.0521	-0.7293	0.5605	0.0775	0.9475	-1.1405	0.1332	2.7690	0.0002	0.0545	0.9603	-0.2657	0.8942	-0.6434	0.5891	-0.5460	0.5562	-0.4295	0.7799	0.5525	0.4705
ATCG00770	-0.8620	0.2897	-0.0366	0.9769	-0.1540	0.9112	1.9060	0.0391	-0.6119	0.6475	0.2243	0.8332	-0.8255	0.2965	2.8696	0.0001	0.2853	0.7642	0.0272	0.9907	-0.5966	0.6213	-0.7818	0.3611	-0.3281	0.8420	0.7899	0.2805
ATCG00790	-0.4097	0.6668	0.1397	0.9057	0.0021	0.9995	2.2951	0.0112	-0.2855	0.8669	0.3180	0.7569	-0.6319	0.4554	3.2374	0.0000	0.0565	0.9593	0.0799	0.9741	-0.0475	0.9817	-0.4167	0.6737	-0.1795	0.9241	2.1438	0.0026
ATCG00820	-0.6518	0.4584	0.1006	0.9340	0.0810	0.9576	2.1712	0.0178	0.3015	0.8583	0.0773	0.9482	-0.7827	0.3373	3.0660	0.0000	0.1790	0.8603	-0.1565	0.9471	-0.5260	0.6839	-0.6050	0.5104	-0.4144	0.7933	0.8426	0.2568
ATCG00830	-0.4886	0.5899	-0.0426	0.9728	0.2937	0.8168	1.5663	0.1110	1.0938	0.2945	0.0407	0.9737	-0.2956	0.7515	2.3521	0.0028	-0.2306	0.8136	-0.1944	0.9274	-0.3207	0.8240	-0.6046	0.5046	-0.1570	0.9332	0.5077	0.5099
ATCG00840	-0.7830	0.3478	-0.2377	0.8321	-0.2796	0.8266	1.5229	0.1254	1.1018	0.2914	-0.0168	0.9901	-0.7295	0.3704	2.4045	0.0022	-0.0558	0.9593	-0.3385	0.8485	-0.2367	0.8794	-0.6216	0.4906	-0.3032	0.8562	0.2011	0.8086
ATCG00920	-1.6747	0.0267	-0.7271	0.4411	-0.7113	0.5186	-1.1727	0.2745	3.1121	0.0000	-0.7043	0.4478	-1.8460	0.0104	-0.8757	0.4985	-0.4082	0.6596	-0.9242	0.4518	-0.8950	0.4178	-1.5882	0.0397	-0.8160	0.4994	-0.3073	0.7078
ATCG00950	-2.1152	0.0038	-0.9368	0.2982	-0.5958	0.5958	-1.5830	0.1079	2.7444	0.0002	-0.7089	0.4420	-2.2566	0.0013	-1.1308	0.3052	-0.6144	0.4789	-1.2233	0.2517	-1.2869	0.1968	-1.9826	0.0076	-0.9690	0.3795	-0.8598	0.2426
ATCG01010	-0.8167	0.3251	0.1588	0.8922	-0.2247	0.8637	0.9364	0.4016	-0.1445	0.9410	0.0524	0.9663	-0.9655	0.2151	1.4904	0.1130	0.1390	0.8913	0.1509	0.9486	0.0379	0.9854	-0.6570	0.4629	-0.1275	0.9467	2.2658	0.0012
ATCG01020	-0.3543	0.7131	-0.1602	0.8921	-0.1471	0.9170	1.9072	0.0428	0.0236	0.9939	0.3036	0.7673	-0.4982	0.5694	2.4555	0.0018	-0.0640	0.9528	-0.1108	0.9639	-0.2101	0.8971	-0.3849	0.7006	-0.0442	0.9863	2.0646	0.0035
ATCG01040	0.0374	0.9741	0.4828	0.6349	0.2394	0.8546	0.5585	0.6525	0.0500	0.9839	0.4264	0.6640	-0.8063	0.3186	0.9722	0.4221	0.3492	0.7116	0.5469	0.7216	0.1522	0.9278	-0.7723	0.3776	-0.0162	0.9976	2.4391	0.0005
ATCG01060	-0.1364	0.8993	-0.0163	0.9906	-0.4060	0.7397	0.7085	0.5526	-0.4751	0.7491	0.0134	0.9924	-0.5789	0.4983	0.4713	0.7920	0.2212	0.8235	0.1974	0.9272	-0.1015	0.9536	-0.2241	0.8382	0.0491	0.9845	2.7357	0.0001
ATCG01100	-0.2216	0.8185	0.4075	0.6767	0.2438	0.8424	1.3770	0.1493	-0.0931	0.9636	0.3134	0.7452	-0.6424	0.4139	1.4923	0.0867	0.5018	0.5511	0.8028	0.5059	0.3415	0.7995	-0.5469	0.5319	0.0126	0.9984	2.3557	0.0004
ATCG01110	-0.2301	0.8223	0.2358	0.8348	-0.0610	0.9694	1.6618	0.0899	-0.6020	0.6611	0.0734	0.9511	-1.0197	0.1912	2.1316	0.0093	0.2328	0.8138	0.3432	0.8479	-0.3076	0.8354	-0.8033	0.3545	-0.3297	0.8436	1.0074	0.1678
ATCG01120	-0.2966	0.7668	0.1860	0.8731	0.0341	0.9830	1.7430	0.0743	-0.3507	0.8308	0.1846	0.8678	-0.6204	0.4652	2.4341	0.0021	0.0005	1.0000	0.0306	0.9893	-0.1242	0.9434	-0.5401	0.5667	-0.2881	0.8676	1.7227	0.0159
ATCG01130	-0.3779	0.6938	0.3687	0.7307	0.7882	0.4669	1.8167	0.0578	-0.2746	0.8728	0.3960	0.6909	-0.4914	0.5755	2.3025	0.0040	-0.0860	0.9352	0.3156	0.8674	0.0995	0.9548	-0.5219	0.5800	-0.0058	1.0000	2.6695	0.0001

ATCG01180	-1.9354	0.0082	-0.4589	0.6488	-0.3327	0.7867	-1.0702	0.3192	2.9634	0.0000	-0.4087	0.6738	-2.0967	0.0027	-0.3556	0.8568	-0.5147	0.5587	-0.7744	0.5553	-0.9612	0.3661	-1.8106	0.0150	-0.5053	0.7249	-0.6798	0.3605
ATCG01210	-1.3651	0.0776	-0.3222	0.7673	-0.4283	0.7200	-0.6477	0.5916	3.4251	0.0000	-0.3808	0.7026	-1.5160	0.0391	0.0738	0.9792	-0.2332	0.8128	-0.3842	0.8244	-0.5566	0.6579	-1.2701	0.1101	-0.3206	0.8474	0.1949	0.8160
ATMG00020	-1.8789	0.0039	-1.0150	0.1878	-0.7625	0.4165	-0.9813	0.3068	-1.0109	0.2703	-0.8147	0.3018	-2.2830	0.0002	-1.1323	0.2162	-0.0268	0.9793	-0.7323	0.5244	-0.6553	0.5224	-1.6554	0.0131	-0.8276	0.4075	0.2062	0.7785
ATMG00070	-1.7010	0.0014	-1.5065	0.0073	-1.3859	0.0258	-1.6062	0.0149	-1.9486	0.0006	-1.0437	0.0919	-2.0357	0.0000	-1.3286	0.0331	-0.6701	0.2555	-0.9408	0.2077	-0.9729	0.1744	-1.5572	0.0036	-1.4275	0.0193	-0.3909	0.4807
ATMG00080	-2.1640	0.0001	-1.4692	0.0144	-1.6513	0.0100	-2.0201	0.0027	-1.5662	0.0137	-1.1646	0.0737	-2.2688	0.0000	-1.5303	0.0175	-0.6305	0.3226	-1.1582	0.1216	-0.7496	0.3612	-1.9456	0.0005	-1.1139	0.1159	-0.7862	0.1617
ATMG00650	-1.7200	0.0086	-1.6848	0.0145	-1.4261	0.0710	-1.1212	0.2215	-1.1935	0.1595	-1.0023	0.1906	-2.2956	0.0002	-1.4925	0.0576	-0.6029	0.4214	-0.9780	0.3193	-0.1492	0.9179	-1.6344	0.0138	-1.2491	0.1271	-0.3420	0.6244
ATMG00980	-1.6371	0.0222	-0.7592	0.3847	-1.2267	0.1802	-0.2108	0.8780	-0.1201	0.9491	0.0187	0.9881	-2.1790	0.0011	-0.0579	0.9830	-0.8842	0.2574	1.3222	0.1446	0.3609	0.7775	-1.7911	0.0127	-0.4399	0.7558	-0.3076	0.6884
ATMG01390	-2.3506	0.0002	-1.2122	0.0969	-1.3070	0.1046	-1.6178	0.0532	-1.0558	0.2354	-1.0861	0.1512	-2.6500	0.0000	-1.3153	0.1143	-0.4143	0.6034	-1.1718	0.1965	-0.8832	0.3486	-2.0776	0.0011	-1.2396	0.1271	-0.9083	0.1557
