# Supplementary material for: Unrelated Fungal Rust Candidate Effectors Act on Overlapping Plant Functions
Source: Microorganisms. 2021 May 5;9(5):996. doi: 10.3390/microorganisms9050996 (PMC8148019; doi:10.3390/microorganisms9050996)
Supplement: Supplementary file 1 [file microorganisms-09-00996-s001.zip › Supplementary Table S2.pdf]

Supplementary Table S2. Percentage of identity and similarity, presented as “ID (SIM)”, calculated with pairwise sequence alignment of CEs using Needle.

	**Mlp37347**	**Mlp72983**	**Mlp102036**	**Mlp106078**	**Mlp123218**	**Mlp123227**	**Mlp123531**	**Mlp124256**	**Mlp124266**	**Mlp124357**	**Mlp124466**	**Mlp124497**	**Mlp124499**	**Mlp124518**
**Mlp37347**	100 (100)	3.1 (4.9)	14.1 (28.9)	8.8 (13.8)	1.6 (2.0)	8.1 (12.4)	8.8 (13.1)	2.9 (5.9)	10.7 (16.1)	9.3 (11.0)	10.2 (18.0)	9.8 (11.2)	2.5 (7.0)	14.0 (16.8)
**Mlp72983**		100 (100)	12.2 (14.1)	13.8 (17.3)	22.2 (31.7)	12.0 (16.8)	9.4 (11.3)	3.9 (5.6)	9.6 (12.5)	7.5 (11.3)	3.6 (4.0)	6.6 (8.0)	6.0 (11.5)	4.4 (9.3)
**Mlp102036**			100 (100)	11 (14.2)	8.1 (12.9)	0.6 (1.1)	9.7 (16.1)	7.6 (13.6)	7.0 (14.9)	7.9 (11.8)	8.7 (15.5)	8.8 (13.3)	6.7 (13.5)	8.1 (9.9)
**Mlp106078**				100 (100)	12.4 (16.1)	6.7 (8.4)	6.8 (9.3)	9.2 (14.8)	10.1 (17.1)	7.1 (8.3)	2.8 (6.2)	7.5 (11.3)	10.3 (13.5)	5.9 (8.1)
**Mlp123218**					100 (100)	3.9 (6.7)	7.1 (14.8)	4.6 (7.8)	11.6 (17.6)	9.3 (12.5)	5.1 (10.2)	8.1 (12.6)	6.3 (12.1)	7.9 (14.2)
**Mlp123227**						100 (100)	1.1 (1.1)	1.3 (1.3)	0.6 (1.2)	1.2 (3.7)	1.4 (2.1)	0.7 (0.7)	2.1 (2.1)	6.5 (13.0)
**Mlp123531**							100 (100)	8.0 (8.8)	8.9 (14.3)	9.8 (9.8)	12.4 (17.5)	10.8 (17.2)	4.5 (5.5)	4.2 (5.9)
**Mlp124256**								100 (100)	38.2 (45.6)	22.7 (27.3)	10.5 (14.0)	15.7 (22.9)	14.8 (17.3)	13.9 (24.1)
**Mlp124266**									100 (100)	19.2 (21.2)	0.9 (3.7)	17.1 (28.9)	13.0 (24.7)	0.9 (2.6)
**Mlp124357**										100 (100)	4.3 (4.3)	19.8 (34.6)	14.1 (17.6)	13.3 (2.0)
**Mlp124466**											100 (100)	12.8 (17.9)	21.5 (33.8)	12.0 (16.0)
**Mlp124497**												100 (100)	41.8 (54.5)	20.0 (38.3)
**Mlp124499**													100 (100)	35.7 (53.6)
**Mlp124518**														100 (100)
