# Supplementary material for: Unrelated Fungal Rust Candidate Effectors Act on Overlapping Plant Functions
Source: Microorganisms. 2021 May 5;9(5):996. doi: 10.3390/microorganisms9050996 (PMC8148019; doi:10.3390/microorganisms9050996)
Supplement: Supplementary file 1 [file microorganisms-09-00996-s001.zip › Supplementary Table S3.pdf]

Supplementary Table S3. Summary of metabolomic analysis in negative mode of the data of extractions with 20% and 80% methanol combined. Assigned, CHO, CHON and Mean mass refer exclusively to the sample in question, while the amount of deregulated formulas considers those identified in the sample or in the Control.

Sample	Assigned	CHO	CHON	Mean mass	Highly unsaturated and phenolic	Aliphatic	Peptides	Polyphenolic	Condensed aromatics	Sugars	Identified	De-regulated (%)	Up-regulated (%)	Down-regulated (%)
Mlp37347	2954	1800	1154	451.517	1509	822	402	127	47	47	3576	101 (2.82)	58 (1.62)	43 (1.20)
Mlp72983	3056	2171	885	461.914	1655	887	285	125	40	64	3575	100 (2.80)	22 (0.62)	78 (2.18)
Mlp102036	3045	1893	1152	442.952	1539	802	448	145	64	47	3719	278 (7.48)	164 (4.41)	114 (3.07)
Mlp106078	2796	1691	1105	445.203	1454	769	351	128	54	40	3522	119 (3.38)	46 (1.31)	73 (2.07)
Mlp123218*	2695	1710	985	431.274	1398	715	379	118	53	32	3515	139 (3.95)	97 (2.76)	42 (1.19)
Mlp123227	2679	1663	1016	439.461	1320	754	378	122	59	46	3645	353 (9.68)	172 (4.72)	181 (4.97)
Mlp123531	3073	1946	1127	450.879	1610	815	422	125	57	44	3599	113 (3.14)	70 (1.94)	43 (1.19)
Mlp124256	2815	1887	928	453.082	1495	829	248	134	57	52	3470	77 (2.22)	25 (0.72)	52 (1.50)
Mlp124266	2754	1679	1075	435.403	1340	798	395	122	58	41	3595	244 (6.79)	90 (2.50)	154 (4.28)
Mlp124357	3151	1983	1168	448.153	1599	892	428	131	53	48	3689	148 (4.01)	64 (1.73)	84 (2.28)
Mlp124466	3026	1969	1057	450.488	1587	863	361	122	50	43	3530	69 (1.95)	24 (0.68)	45 (1.27)
Mlp124497	2937	1801	1136	449.426	1464	844	418	122	52	37	3601	171 (4.75)	82 (2.28)	89 (2.47)
Mlp124499	2946	1878	1068	443.459	1493	827	375	150	63	38	3613	217 (6.01)	118 (3.27)	99 (2.74)
Mlp124518	2735	1651	1084	437.807	1345	767	400	122	61	40	3547	237 (6.68)	117 (3.30)	120 (3.38)
Control	3113	2097	1016	458.505	1712	835	309	143	56	58	3113	-	-	-

* For the transgenic line Mlp123218, only the extraction with 20% methanol was analysed, thus the sample was compared to the Control extraction with 20% methanol.
