# Supplementary material for: Unrelated Fungal Rust Candidate Effectors Act on Overlapping Plant Functions
Source: Microorganisms. 2021 May 5;9(5):996. doi: 10.3390/microorganisms9050996 (PMC8148019; doi:10.3390/microorganisms9050996)
Supplement: Supplementary file 1 [file microorganisms-09-00996-s001.zip › Supplementary Table S4.pdf]

Supplementary Table S4. Metabolites assigned and deregulated in each sample separated by category. Identified metabolites are those present either in the sample or in the control. The percentages were calculated by dividing the number of formulas assigned or deregulated in the sample in each category by the number of formulas identified in that sample and multiplying by 100.

Sample	Compound category	Identified (%)	Assigned	Deregulated (%)	Up (%)	Down (%)
Control	Polyphenolic	143	-	-	-	-
	Highly unsaturated and phenolic	1712	-	-	-	-
	Peptide-like	309	-	-	-	-
	Aliphatic	835	-	-	-	-
	Sugar	58	-	-	-	-
	Condensed aromatic	56	-	-	-	-
Mlp37347	Polyphenolic	1947 (54.45)	1509	57 (1.59)	26 (0.73)	31 (0.87)
	Highly unsaturated and phenolic	437 (12.22)	402	20 (0.56)	20 (0.56)	0 (0.00)
	Peptide-like	61 (1.71)	47	2 (0.06)	1 (0.03)	1 (0.03)
	Aliphatic	166 (4.64)	127	8 (0.22)	5 (0.14)	3 (0.08)
	Sugar	67 (1.87)	47	1 (0.03)	1 (0.03)	0 (0.00)
	Condensed aromatic	898 (25.11)	822	13 (0.36)	5 (0.14)	8 (0.22)
Mlp72983	Polyphenolic	1966 (54.99)	1655	63 (1.76)	13 (0.36)	50 (1.40)
	Highly unsaturated and phenolic	366 (10.24)	285	13 (0.36)	2 (0.06)	11 (0.31)
	Peptide-like	62 (1.73)	40	6 (0.17)	0 (0.00)	6 (0.17)
	Aliphatic	166 (4.64)	125	7 (0.20)	1 (0.03)	6 (0.17)
	Sugar	80 (2.24)	64	2 (0.06)	0 (0.00)	2 (0.06)
	Condensed aromatic	935 (26.15)	887	9 (0.25)	6 (0.17)	3 (0.08)
Mlp102036	Polyphenolic	2004 (53.89)	1539	158 (4.25)	68 (1.83)	90 (2.42)
	Highly unsaturated and phenolic	495 (13.31)	448	75 (2.02)	69 (1.86)	6 (0.16)
	Peptide-like	75 (2.02)	64	3 (0.08)	2 (0.05)	1 (0.03)
	Aliphatic	178 (4.79)	145	14 (0.38)	7 (0.19)	7 (0.19)
	Sugar	70 (1.88)	47	6 (0.16)	3 (0.08)	3 (0.08)
	Condensed aromatic	897 (24.12)	802	22 (0.59)	15 (0.40)	7 (0.19)
Mlp106078	Polyphenolic	1942 (55.14)	1454	78 (2.21)	25 (0.71)	53 (1.50)
	Highly unsaturated and phenolic	400 (11.36)	351	17 (0.48)	14 (0.40)	3 (0.09)
	Peptide-like	66 (1.87)	54	2 (0.06)	0 (0.00)	2 (0.06)
	Aliphatic	172 (4.88)	128	9 (0.26)	1 (0.03)	8 (0.23)
	Sugar	64 (1.82)	40	0 (0.00)	0 (0.00)	0 (0.00)
	Condensed aromatic	878 (24.93)	769	13 (0.37)	6 (0.17)	7 (0.20)
Mlp123218	Polyphenolic	1895 (53.91)	1398	67 (1.91)	36 (1.02)	31 (0.88)
	Highly unsaturated and phenolic	444 (12.63)	379	53 (1.51)	49 (1.39)	4 (0.11)
	Peptide-like	69 (1.96)	53	3 (0.09)	2 (0.06)	1 (0.03)
	Aliphatic	166 (4.72)	118	4 (0.11)	2 (0.06)	2 (0.06)
	Sugar	62 (1.76)	32	2 (0.06)	0 (0.00)	2 (0.06)
	Condensed aromatic	879 (25.01)	715	10 (0.28)	8 (0.23)	2 (0.06)
Mlp123227	Polyphenolic	1974 (54.16)	1320	214 (5.87)	71 (1.95)	143 (3.92)
	Highly unsaturated and phenolic	439 (12.04)	378	59 (1.62)	54 (1.48)	5 (0.14)
	Peptide-like	77 (2.11)	59	12 (0.33)	5 (0.14)	7 (0.19)
	Aliphatic	182 (4.99)	122	20 (0.55)	8 (0.22)	12 (0.33)
	Sugar	76 (2.09)	46	8 (0.22)	4 (0.11)	4 (0.11)
	Condensed aromatic	897 (24.61)	754	40 (1.10)	30 (0.82)	10 (0.27)

Mlp123531	Polyphenolic	1951 (54.21)	1610	59 (1.64)	28 (0.78)	31 (0.86)
	Highly unsaturated and phenolic	449 (12.48)	422	28 (0.78)	27 (0.75)	1 (0.03)
	Peptide-like	68 (1.89)	57	2 (0.06)	1 (0.03)	1 (0.03)
	Aliphatic	162 (4.50)	125	6 (0.17)	2 (0.06)	4 (0.11)
	Sugar	68 (1.89)	44	4 (0.11)	1 (0.03)	3 (0.08)
	Condensed aromatic	901 (25.03)	815	14 (0.39)	11 (0.31)	3 (0.08)
Mlp124256	Polyphenolic	1916 (55.22)	1495	49 (1.41)	14 (0.40)	35 (1.01)
	Highly unsaturated and phenolic	340 (9.80)	248	10 (0.29)	3 (0.09)	7 (0.20)
	Peptide-like	68 (1.96)	57	4 (0.12)	3 (0.09)	1 (0.03)
	Aliphatic	171 (4.93)	134	1 (0.03)	0 (0.00)	1 (0.03)
	Sugar	77 (2.22)	52	4 (0.12)	2 (0.06)	2 (0.06)
	Condensed aromatic	898 (25.88)	829	9 (0.26)	3 (0.09)	6 (0.17)
Mlp124266	Polyphenolic	1936 (53.85)	1340	153 (4.26)	34 (0.95)	119 (3.31)
	Highly unsaturated and phenolic	438 (12.18)	395	32 (0.89)	26 (0.72)	6 (0.17)
	Peptide-like	74 (2.06)	58	9 (0.25)	4 (0.11)	5 (0.14)
	Aliphatic	168 (4.67)	122	14 (0.39)	5 (0.14)	9 (0.25)
	Sugar	71 (1.97)	41	8 (0.22)	5 (0.14)	3 (0.08)
	Condensed aromatic	908 (25.26)	798	28 (0.78)	16 (0.45)	12 (0.33)
Mlp124357	Polyphenolic	1983 (53.75)	1599	94 (2.55)	27 (0.73)	67 (1.82)
	Highly unsaturated and phenolic	459 (12.44)	428	24 (0.65)	23 (0.62)	1 (0.03)
	Peptide-like	67 (1.82)	53	6 (0.16)	2 (0.05)	4 (0.11)
	Aliphatic	169 (4.58)	131	9 (0.24)	4 (0.11)	5 (0.14)
	Sugar	71 (1.92)	48	2 (0.05)	0 (0.00)	2 (0.05)
	Condensed aromatic	940 (25.48)	892	13 (0.35)	8 (0.22)	5 (0.14)
Mlp124466	Polyphenolic	1920 (54.39)	1587	50 (1.42)	14 (0.40)	36 (1.02)
	Highly unsaturated and phenolic	402 (11.39)	361	5 (0.14)	3 (0.08)	2 (0.06)
	Peptide-like	62 (1.76)	50	0 (0.00)	0 (0.00)	0 (0.00)
	Aliphatic	164 (4.65)	122	4 (0.11)	1 (0.03)	3 (0.08)
	Sugar	66 (1.87)	43	0 (0.00)	0 (0.00)	0 (0.00)
	Condensed aromatic	916 (25.95)	863	10 (0.28)	6 (0.17)	4 (0.11)
Mlp124497	Polyphenolic	1951 (54.18)	1464	99 (2.75)	30 (0.83)	69 (1.92)
	Highly unsaturated and phenolic	448 (12.44)	418	44 (1.22)	44 (1.22)	0 (0.00)
	Peptide-like	64 (1.78)	52	3 (0.08)	0 (0.00)	3 (0.08)
	Aliphatic	162 (4.50)	122	6 (0.17)	0 (0.00)	6 (0.17)
	Sugar	67 (1.86)	37	3 (0.08)	0 (0.00)	3 (0.08)
	Condensed aromatic	909 (25.24)	844	16 (0.44)	8 (0.22)	8 (0.22)
Mlp124499	Polyphenolic	1960 (54.25)	1493	134 (3.71)	48 (1.33)	86 (2.38)
	Highly unsaturated and phenolic	423 (11.71)	375	48 (1.33)	47 (1.30)	1 (0.03)
	Peptide-like	71 (1.97)	63	2 (0.06)	1 (0.03)	1 (0.03)
	Aliphatic	185 (5.12)	150	12 (0.33)	5 (0.14)	7 (0.19)
	Sugar	65 (1.80)	38	2 (0.06)	1 (0.03)	1 (0.03)
	Condensed aromatic	909 (25.16)	827	19 (0.53)	16 (0.44)	3 (0.08)
Mlp124518	Polyphenolic	1913 (53.93)	1345	145 (4.09)	47 (1.33)	98 (2.76)
	Highly unsaturated and phenolic	438 (12.35)	400	54 (1.52)	53 (1.49)	1 (0.03)
	Peptide-like	72 (2.03)	61	4 (0.11)	3 (0.08)	1 (0.03)
	Aliphatic	171 (4.82)	122	11 (0.31)	5 (0.14)	6 (0.17)
	Sugar	68 (1.92)	40	5 (0.14)	2 (0.06)	3 (0.08)
	Condensed aromatic	885 (24.95)	767	18 (0.51)	7 (0.20)	11 (0.31)
