# Supplementary material for: Unrelated Fungal Rust Candidate Effectors Act on Overlapping Plant Functions
Source: Microorganisms. 2021 May 5;9(5):996. doi: 10.3390/microorganisms9050996 (PMC8148019; doi:10.3390/microorganisms9050996)
Supplement: Supplementary file 1 [file microorganisms-09-00996-s001.zip › Supplementary Table S5.pdf]

Supplementary Table S5. Parameters used for bioinformatic analyses.

<table>
<tr><th>Analysis step</th><th colspan="2">Software</th><th>Parameters</th></tr>
<tr><td>Read trimming and filtering</td><td colspan="2">Trimmomatic</td><td>\-phred 33 LEADING:3 TRAILING:3 SLIDINGWINDOW:4:15 MINLEN:25</td></tr>
<tr><td>Read alignment</td><td colspan="2">HISAT2</td><td>default parameters and mate inner distance according to the replicate</td></tr>
<tr><td rowspan="2">Read count</td><td rowspan="8">R 4.0.0</td><td>GenomicFeatures</td><td>makeTxDbFromBiomart(biomart = "plants_mart", dataset = "athaliana_eg_gene", id_prefix = "ensembl_", host = "plants.ensembl.org", taxonomyId = 3702) %>% transcriptsBy("gene")</td></tr>
<tr><td>GenomicAlignments</td><td>summarizeOverlaps with mode = Union, singleEnd = F, ignore.strand = FALSE, fragments = T</td></tr>
<tr><td>Filtering out weakly expressed genes</td><td>CustomSeletion</td><td>mean(TPM) < mean(DAFS cutoff)</td></tr>
<tr><td>Variation between replicates and samples</td><td rowspan="2">DESeq2</td><td>plotPCA with ntop = All Arabidopsis genes expressed in samples</td></tr>
<tr><td>Differential expression analysis</td><td>estimateSizeFactors with controlGenes; DESeq(betaPrior = T), |log2(Fold change)| ≥ 2 and adjusted pValue ≤ 0.01</td></tr>
<tr><td>GO enrichment</td><td>clusterProfiler</td><td>enrichGO with universe = All Arabidopsis genes expressed in samples, OrgDb = org.At.tair.db, ont = "BP", keyType = "TAIR", pAdjustMethod = "BH", pvalueCutoff = 0.01, qvalueCutoff = 0.05. readable = TRUE) %>% simplify()</td></tr>
<tr><td>KEGG enrichment</td><td>KEGGprofile</td><td>find_enriched_pathway with species = "ath" and download_latest = TRUE</td></tr>
<tr><td>Co-expression network analysis</td><td>WGCNA</td><td>blockwiseModules with log2-transformed TPM values of deregulated genes, power = 14, TOMType = "unsigned", minModuleSize = 30, reassignThreshold = 0, mergeCutHeight = 0.3, numericLabels = TRUE, pamRespectsDendro = FALSE</td></tr>
</table>
