# Supplementary material for: Unrelated Fungal Rust Candidate Effectors Act on Overlapping Plant Functions
Source: Microorganisms. 2021 May 5;9(5):996. doi: 10.3390/microorganisms9050996 (PMC8148019; doi:10.3390/microorganisms9050996)
Supplement: Supplementary file 1 [file microorganisms-09-00996-s001.zip › File S1.pdf]

microorganisms

MDPI

Supplementary File S1. Heatmaps by KEGG pathway. Pathways with at least 5 genes deregulated across the experiment were selected for display of genes deregulated in each transgenic line.

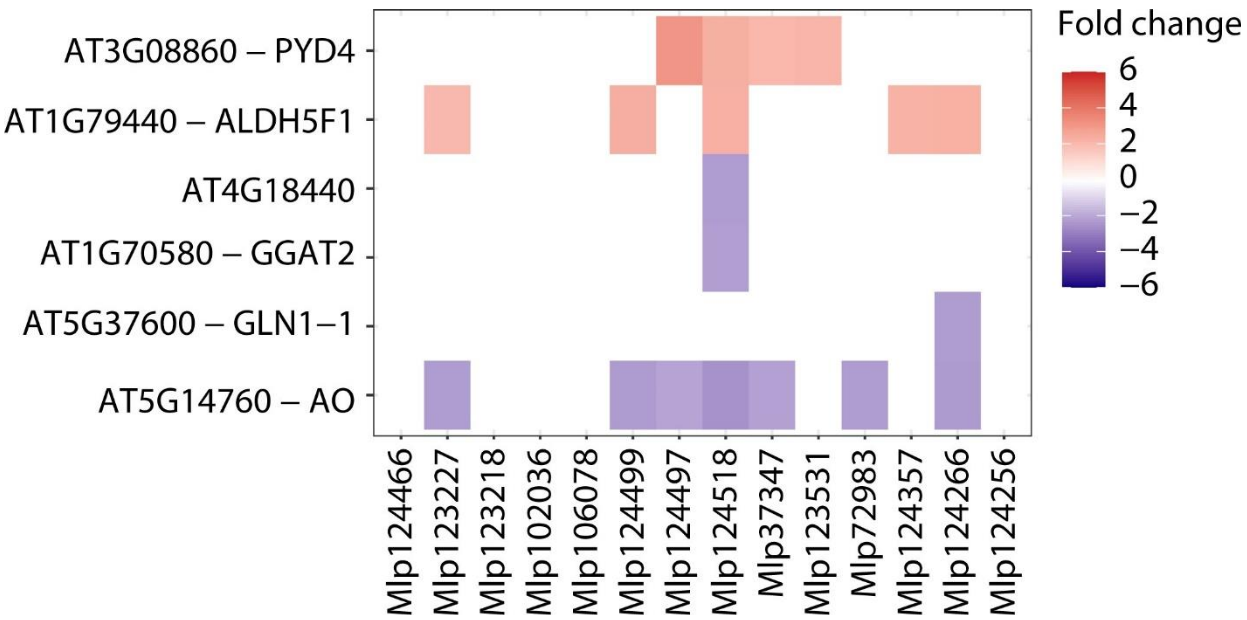

Figure S1. Alanine, aspartate and glutamate metabolism.

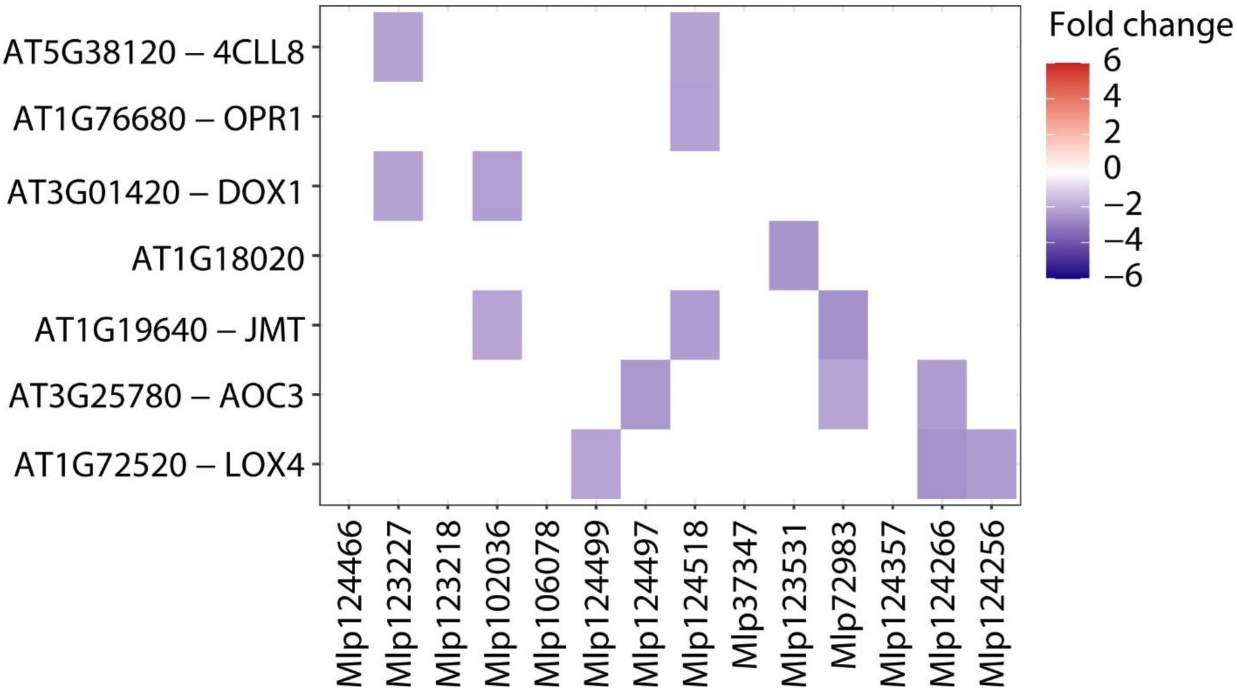

Figure S2. Alpha-Linolenic acid metabolism.

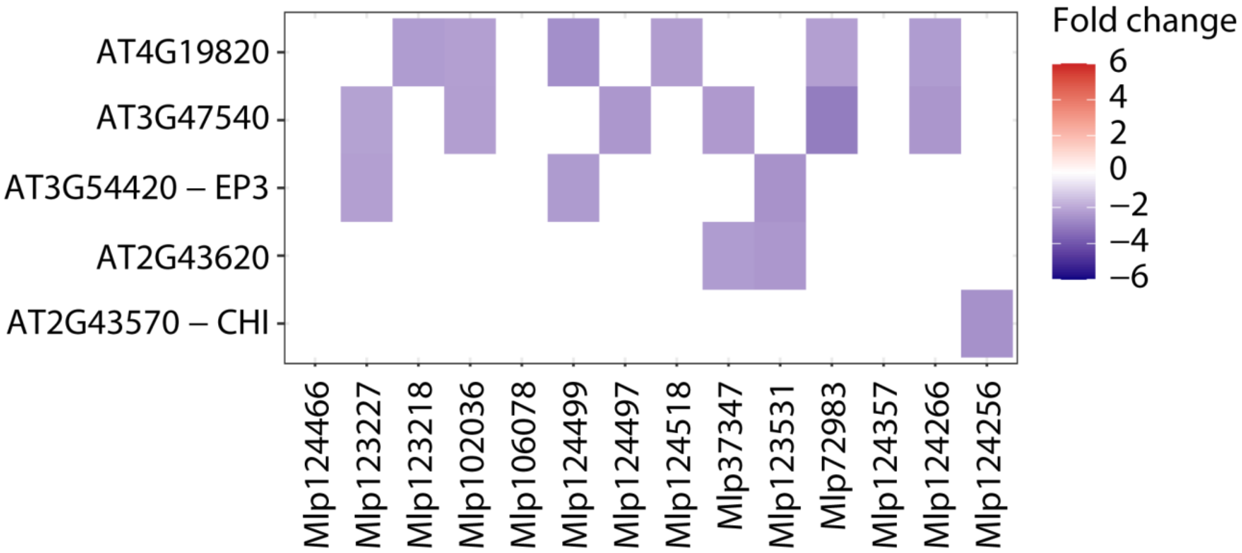

Figure S3. Amino sugar and nucleotide sugar metabolism.

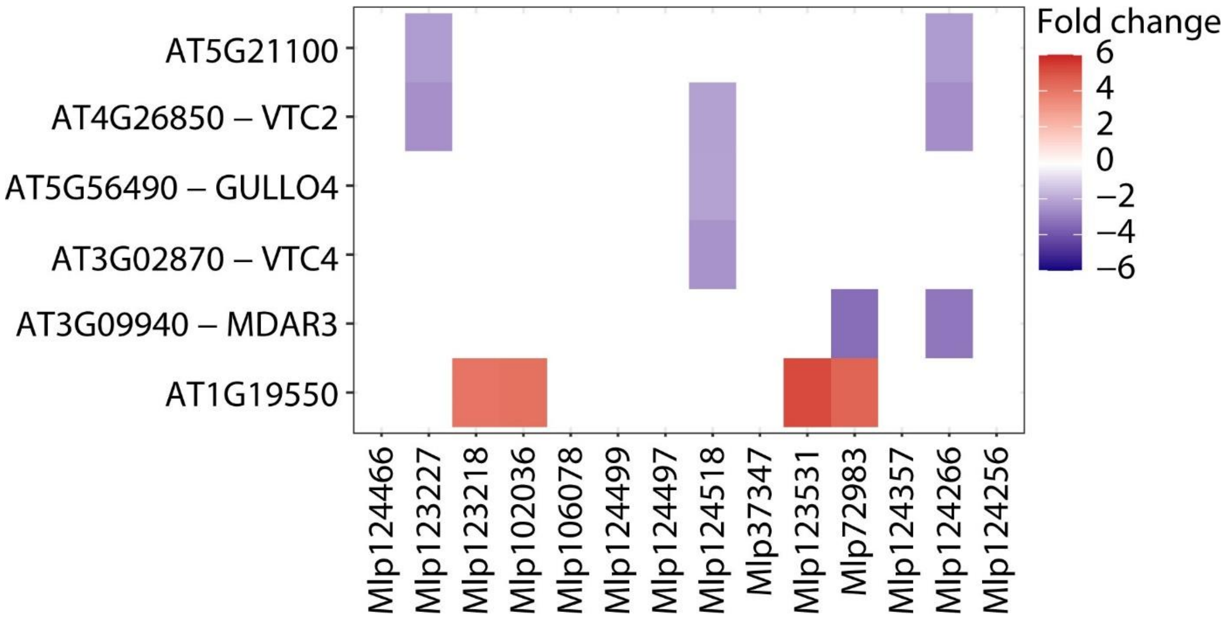

Figure S4. Ascorbate and aldarate metabolism.

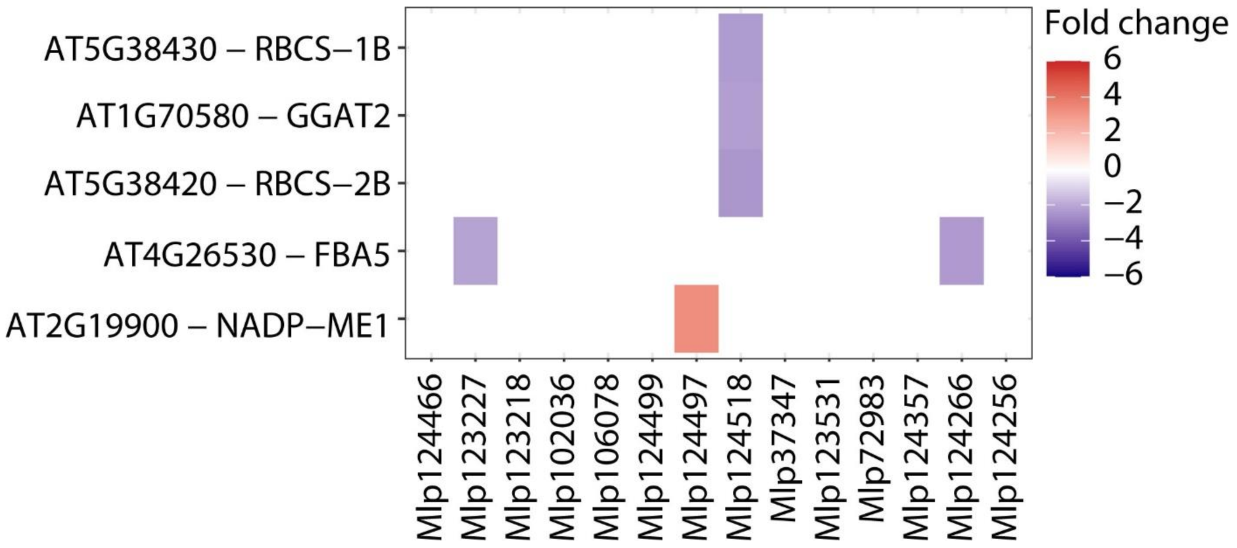

Figure S5. Carbon fixation in photosynthetic organisms.

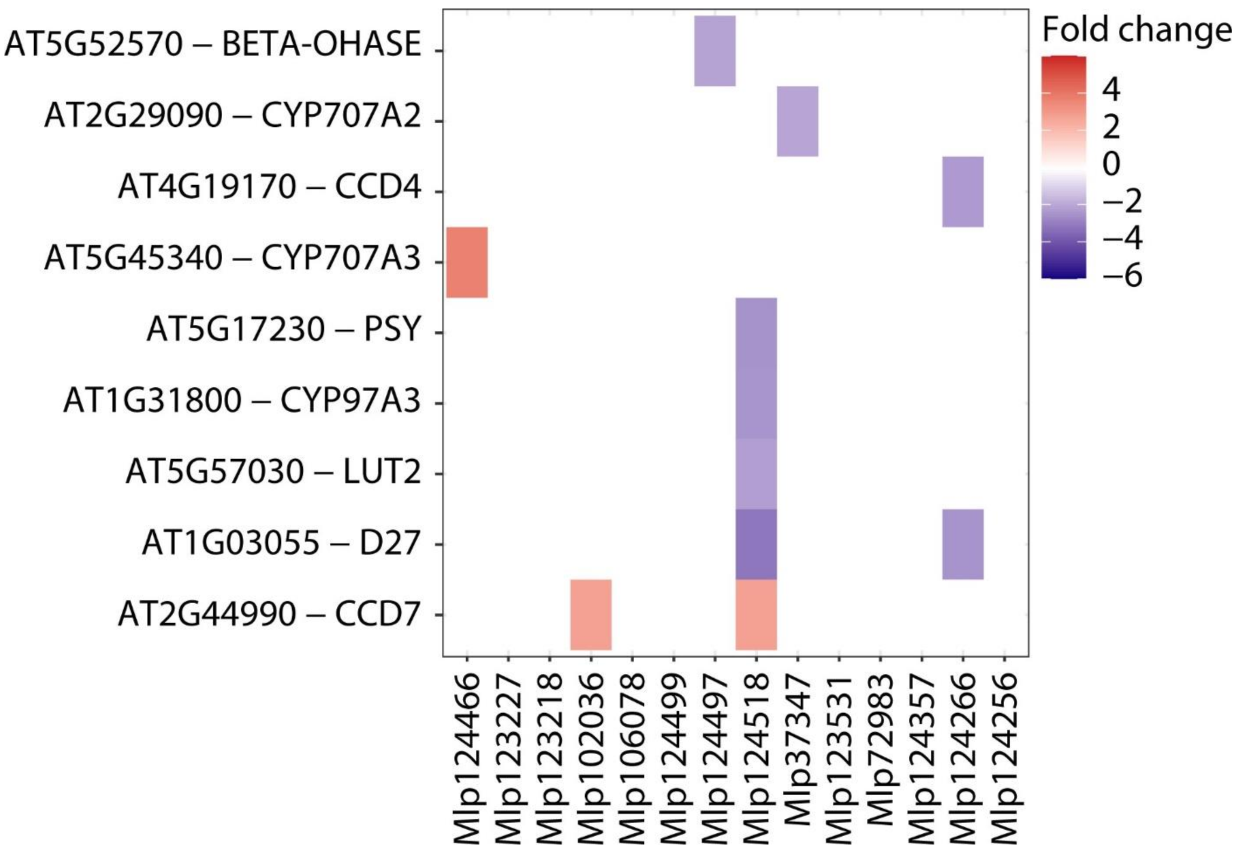

Figure S6. Carotenoid biosynthesis.

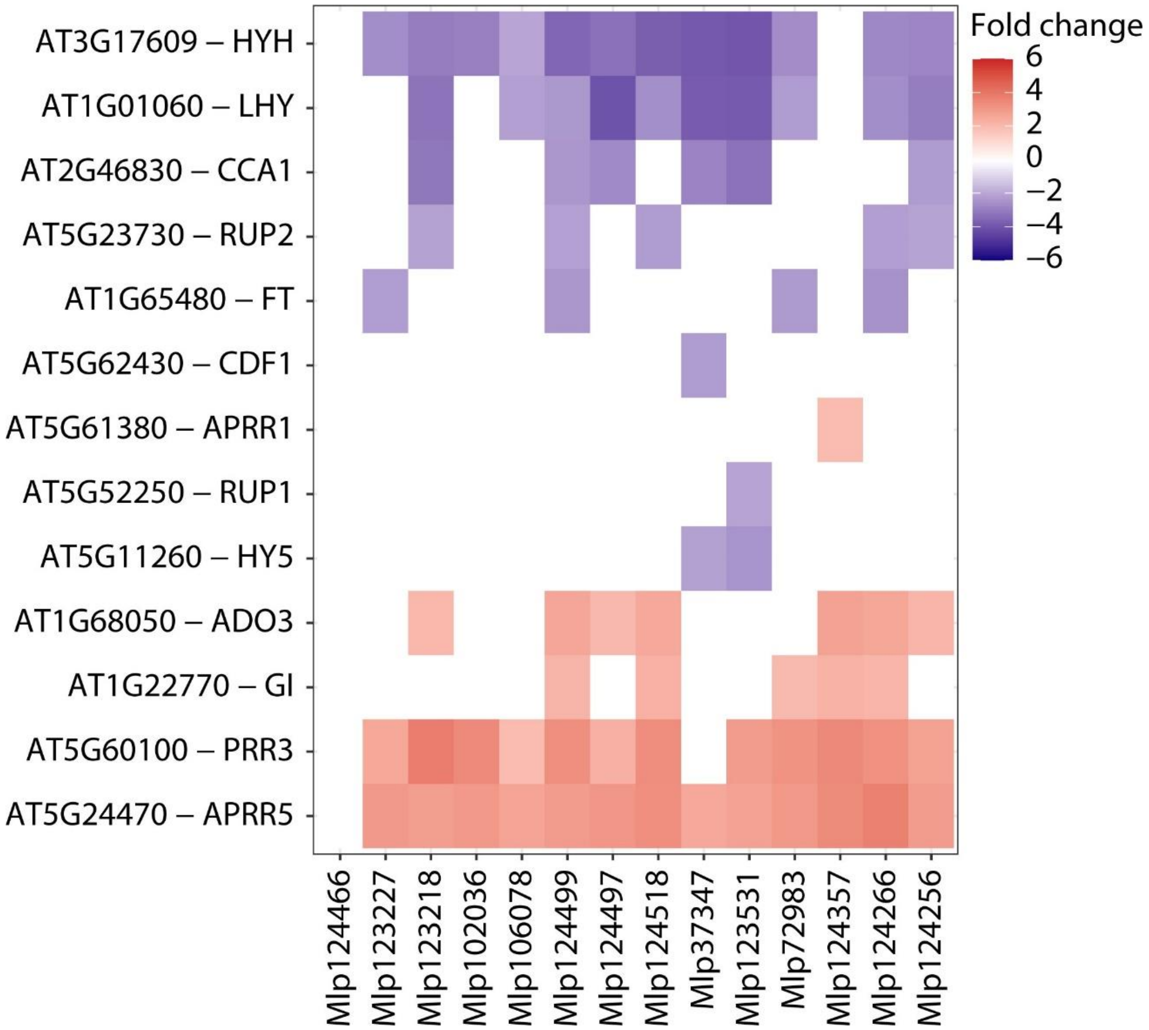

Figure S7. Circadian rhythm—plant.

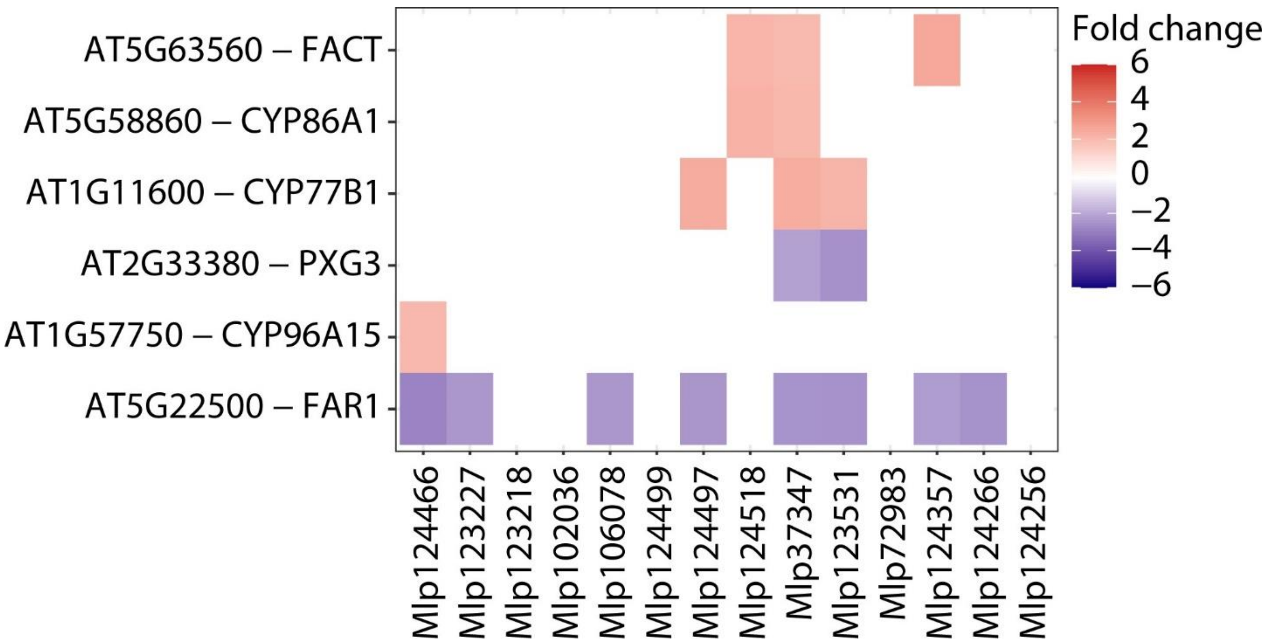

Figure S8. Cutin, suberine and wax biosynthesis.

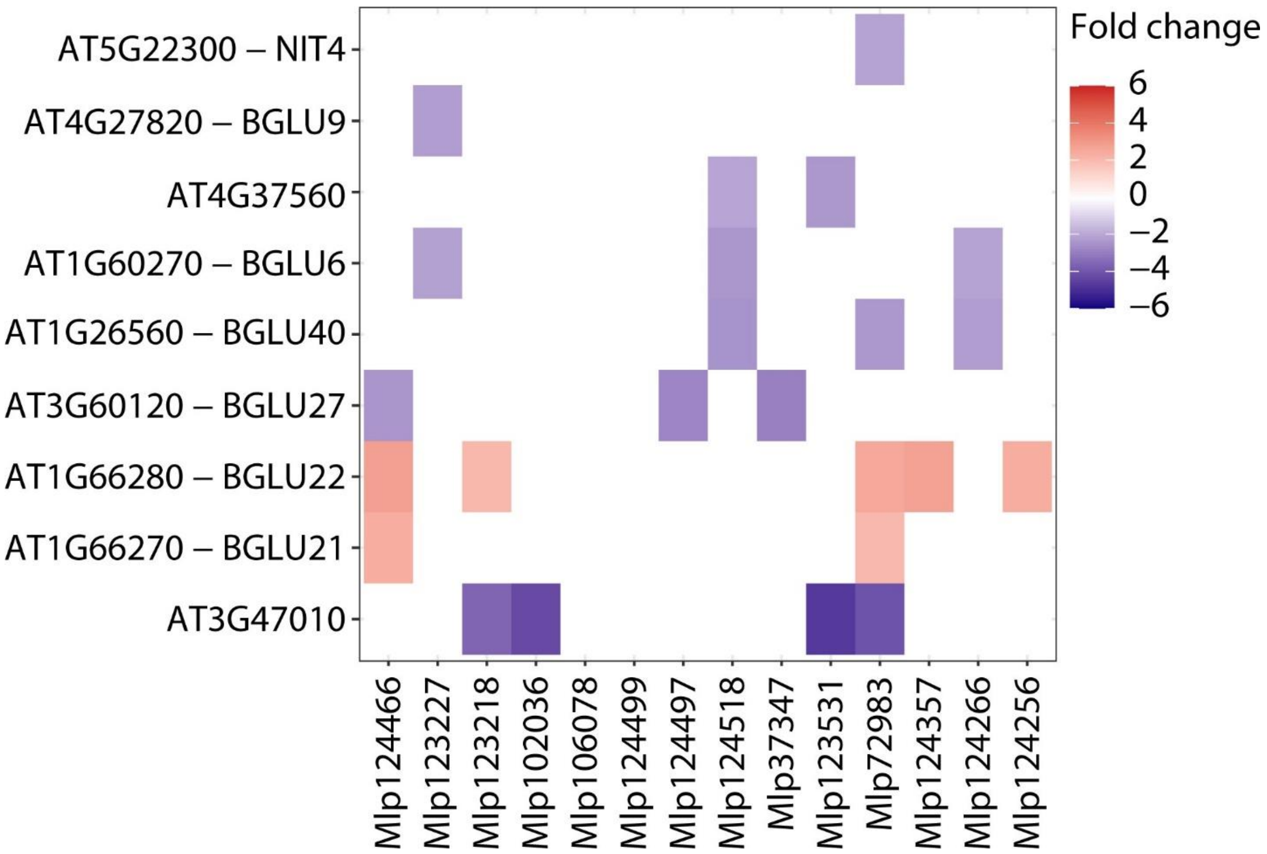

Figure S9. Cyanoamino acid metabolism.

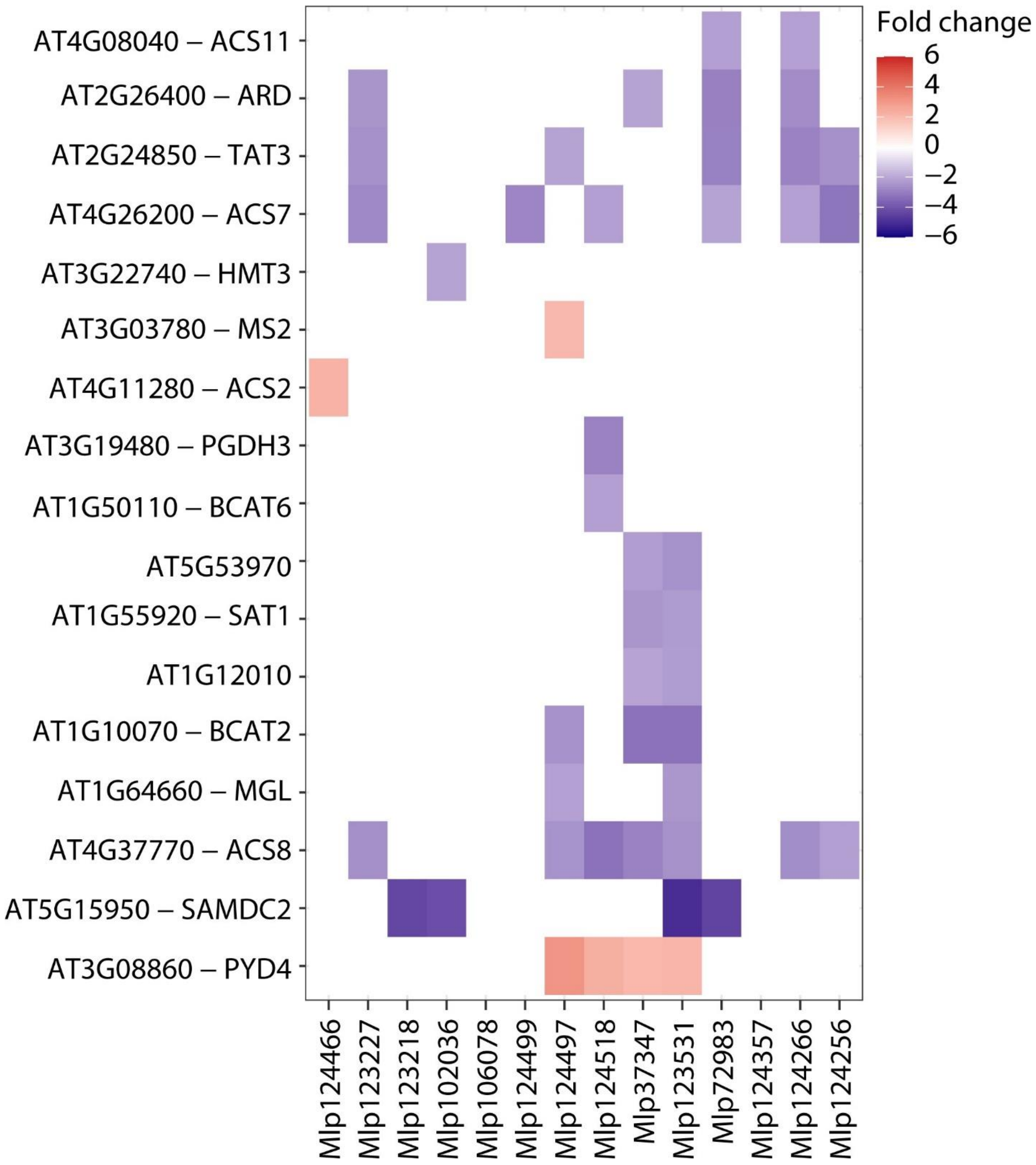

Figure S10. Cysteine and methionine metabolism.

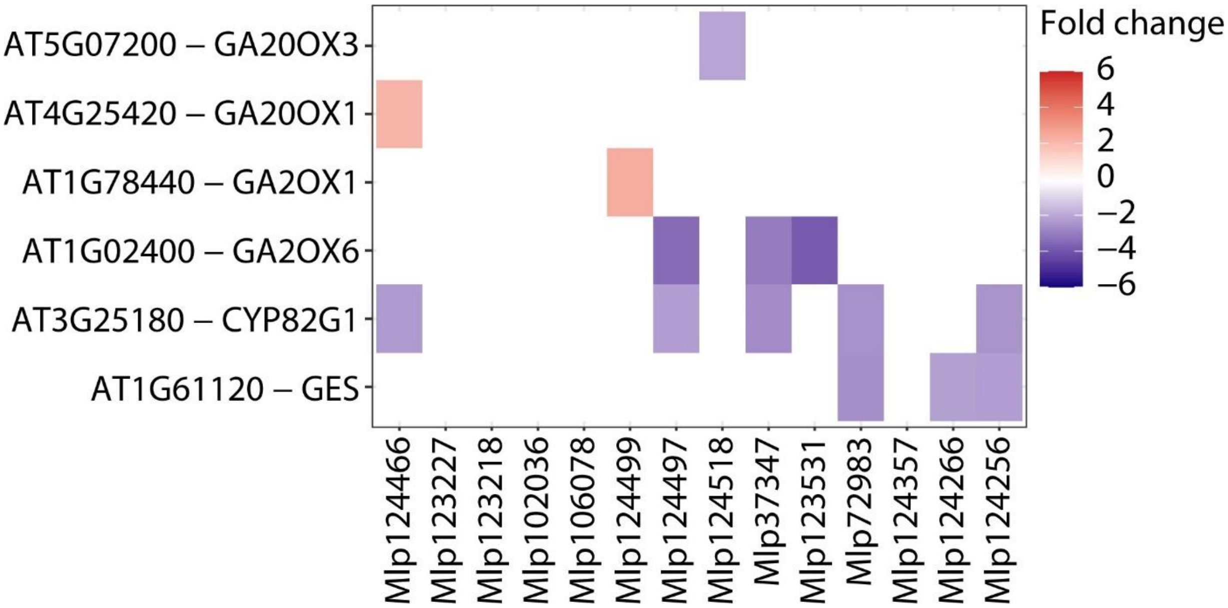

Figure S11. Diterpenoid biosynthesis.

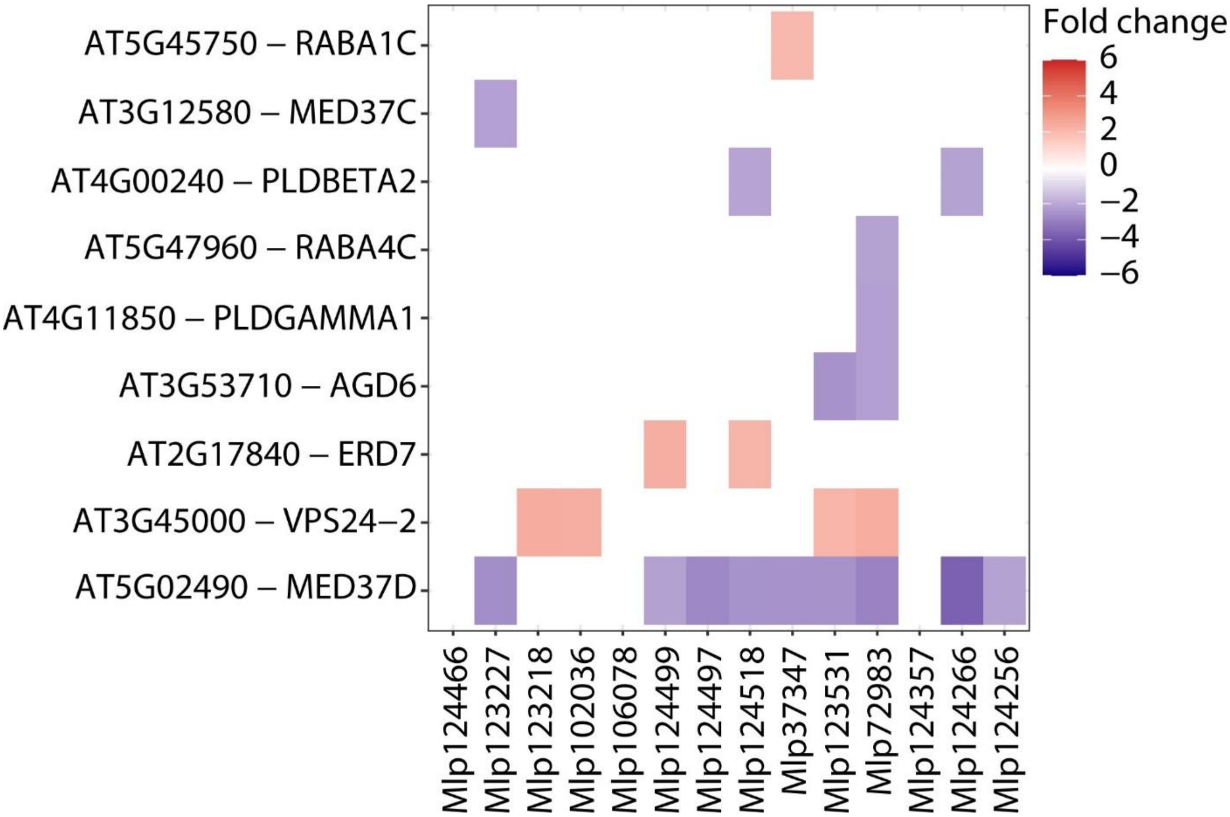

Figure S12. Endocytosis.

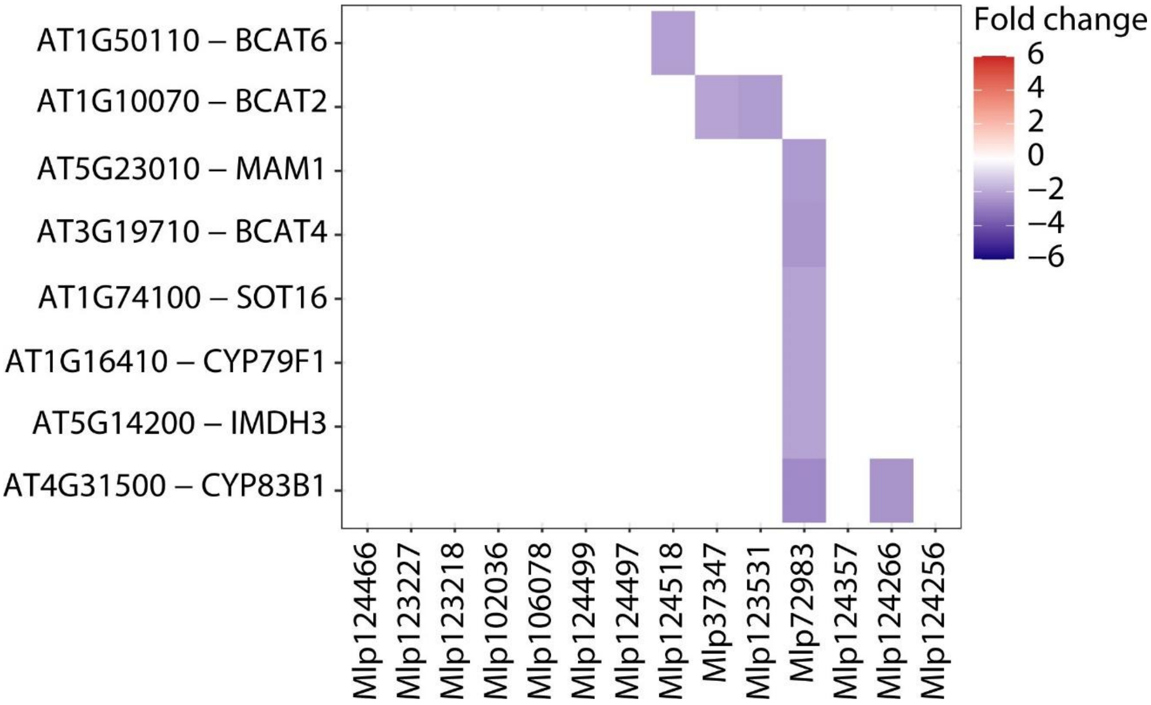

Figure S13. Glucosinolate biosynthesis.

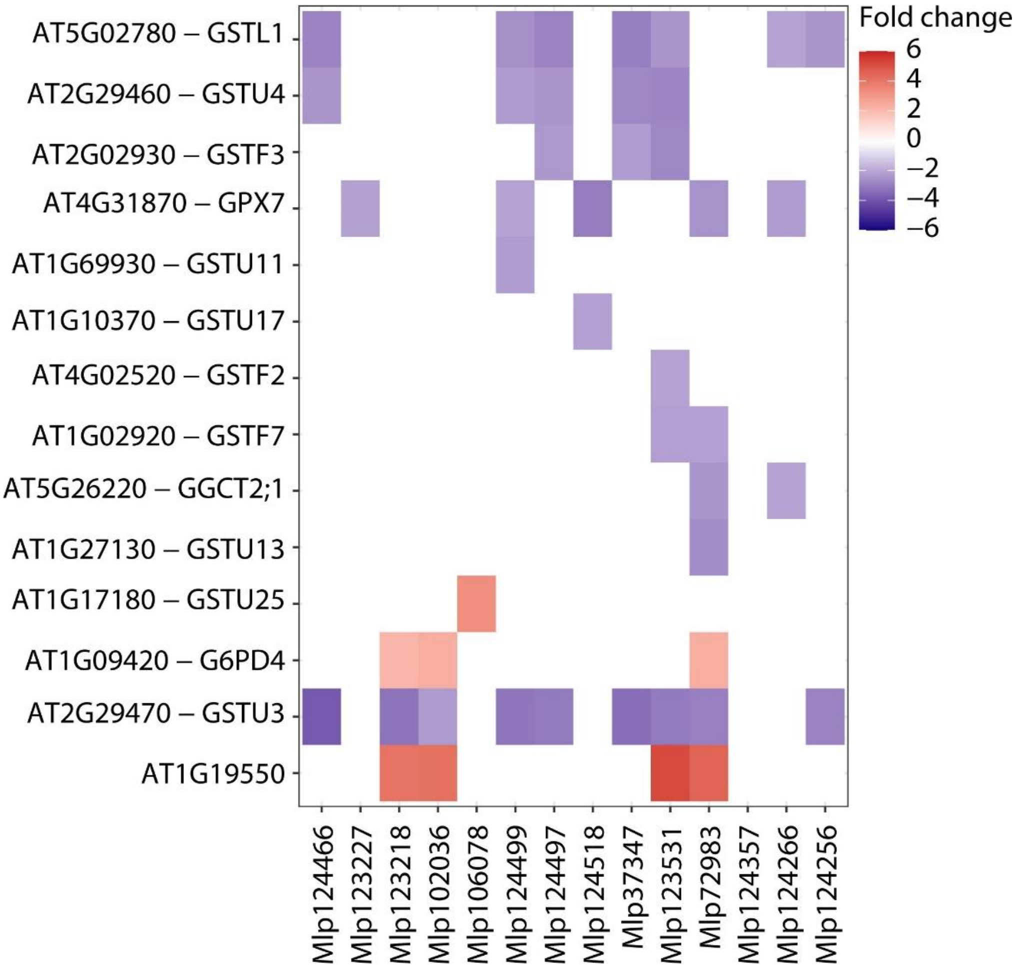

Figure S14. Glutathione metabolism.

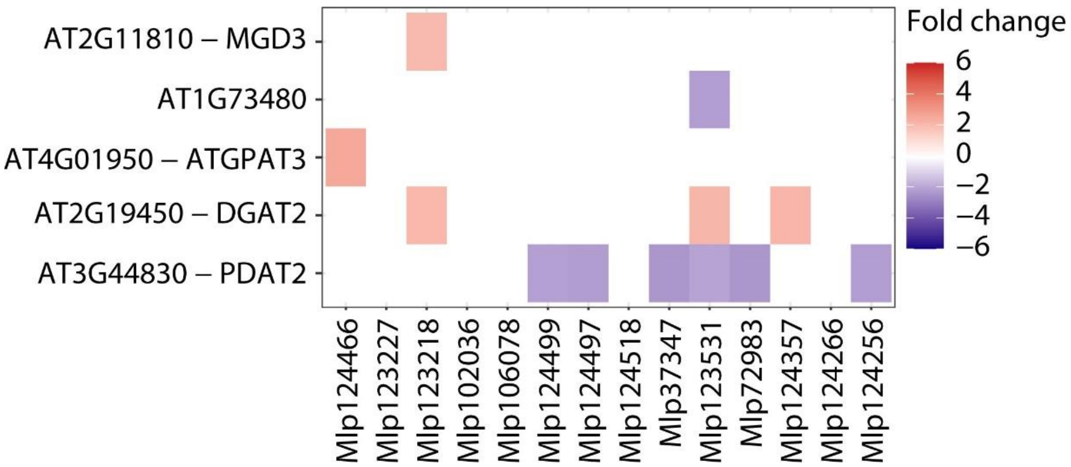

Figure S15. Glycerolipid metabolism.

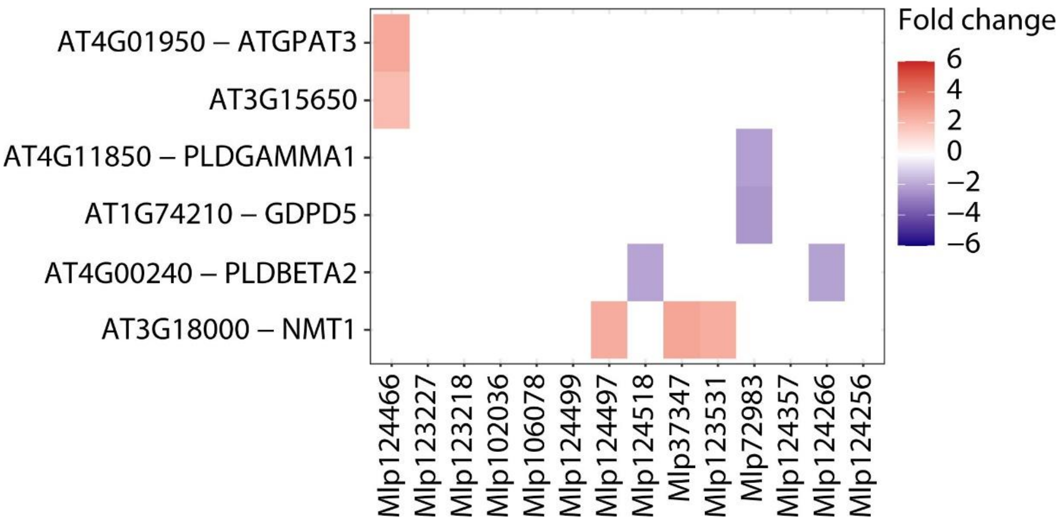

Figure S16. Glycerophospholipid metabolism.

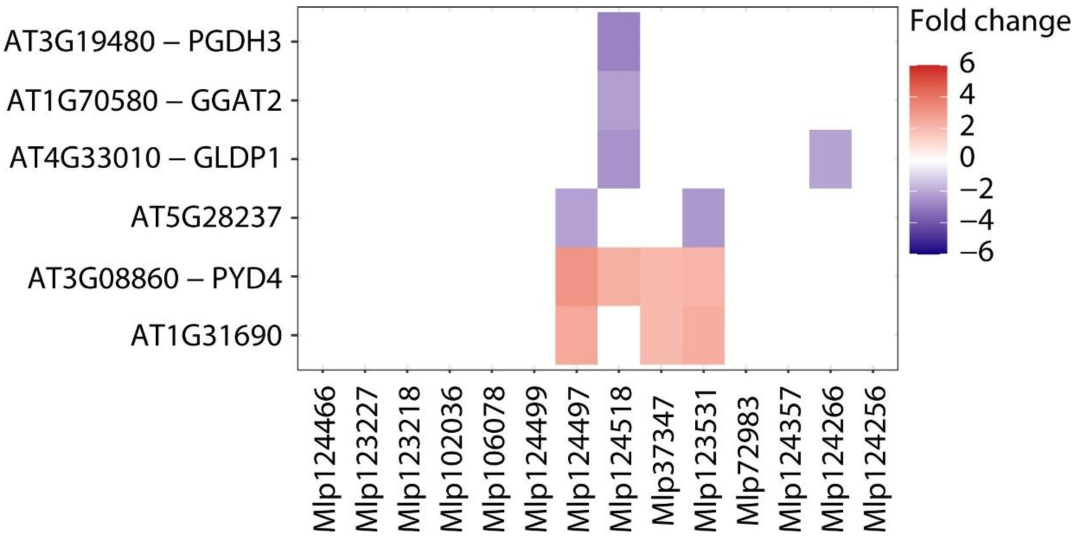

Figure S17. Glycine, serine and threonine metabolism.

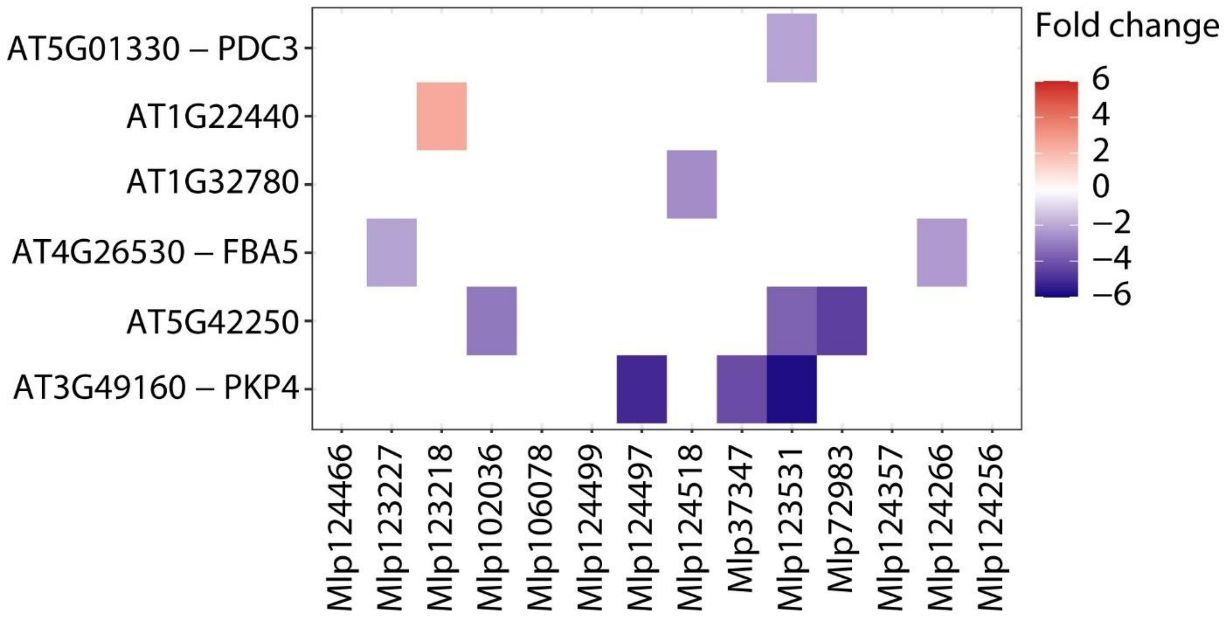

Figure S18. Glycolysis / Gluconeogenesis.

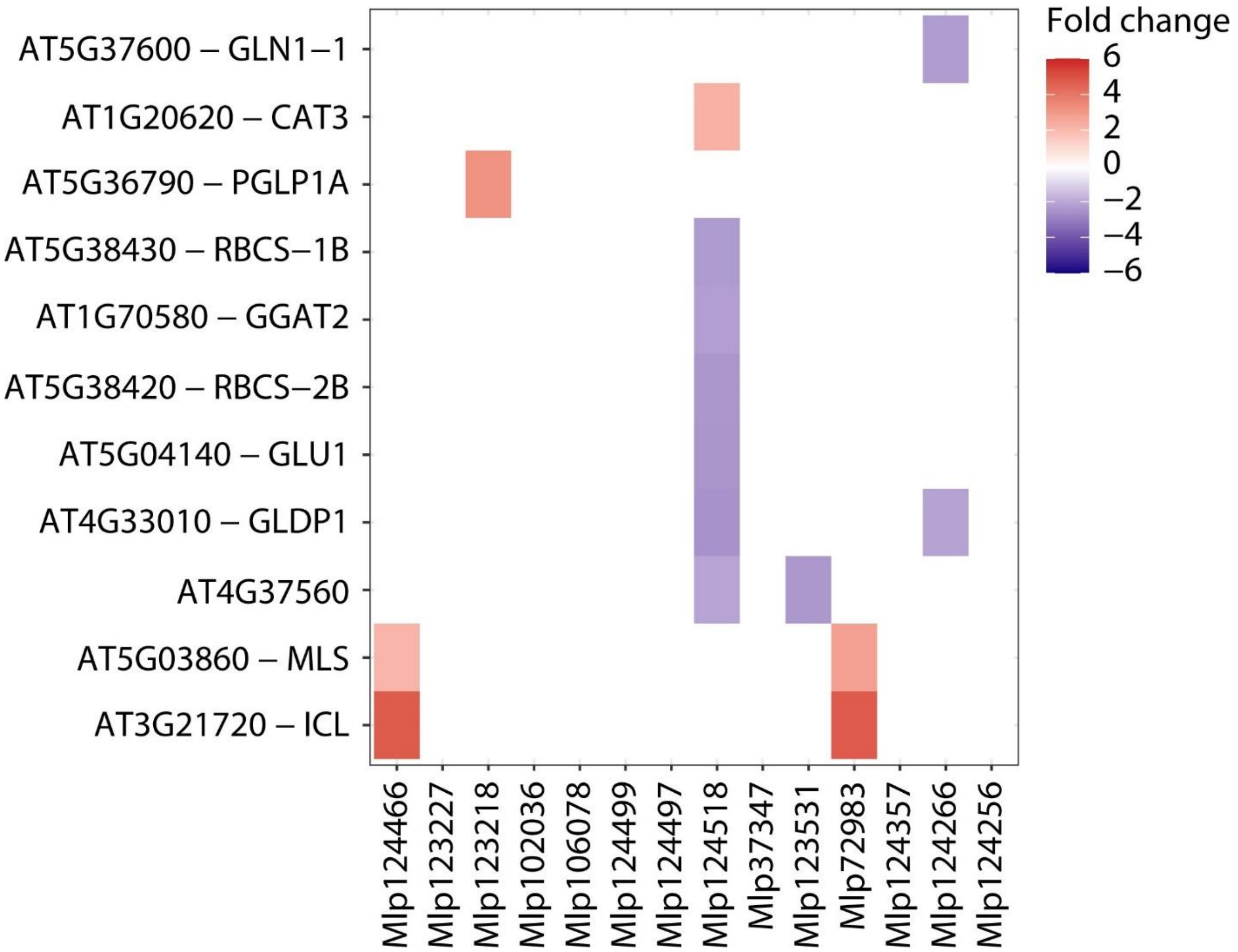

Figure S19. Glyoxylate and dicarboxylate metabolism.

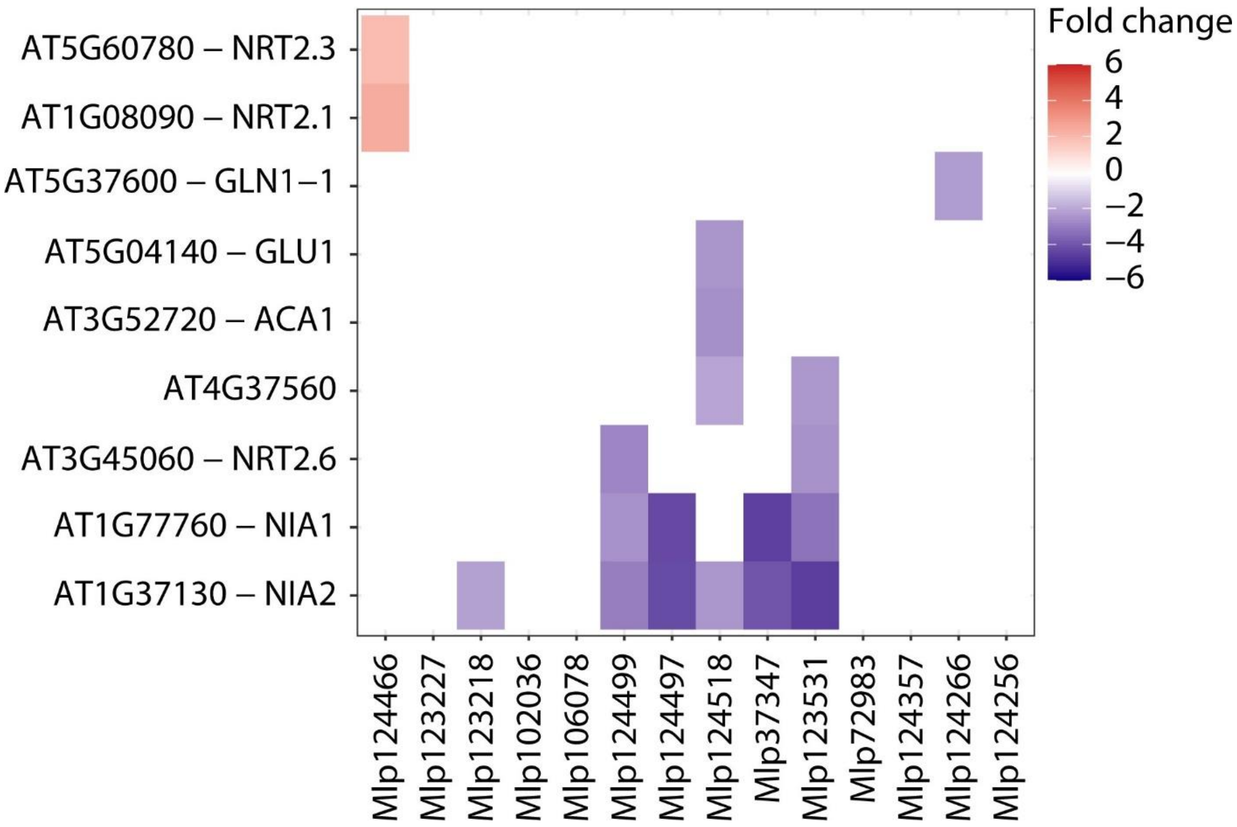

Figure S20. Nitrogen metabolism.

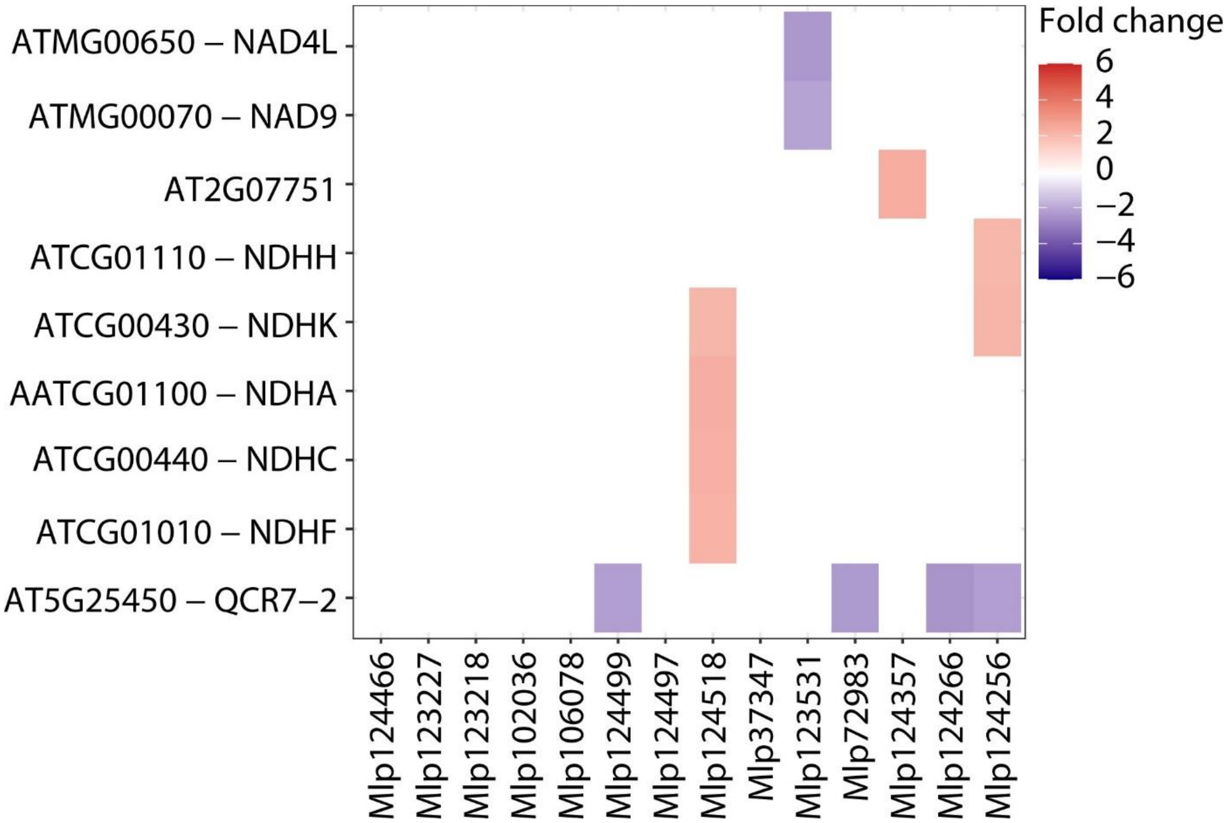

Figure S21. Oxidative phosphorylation.

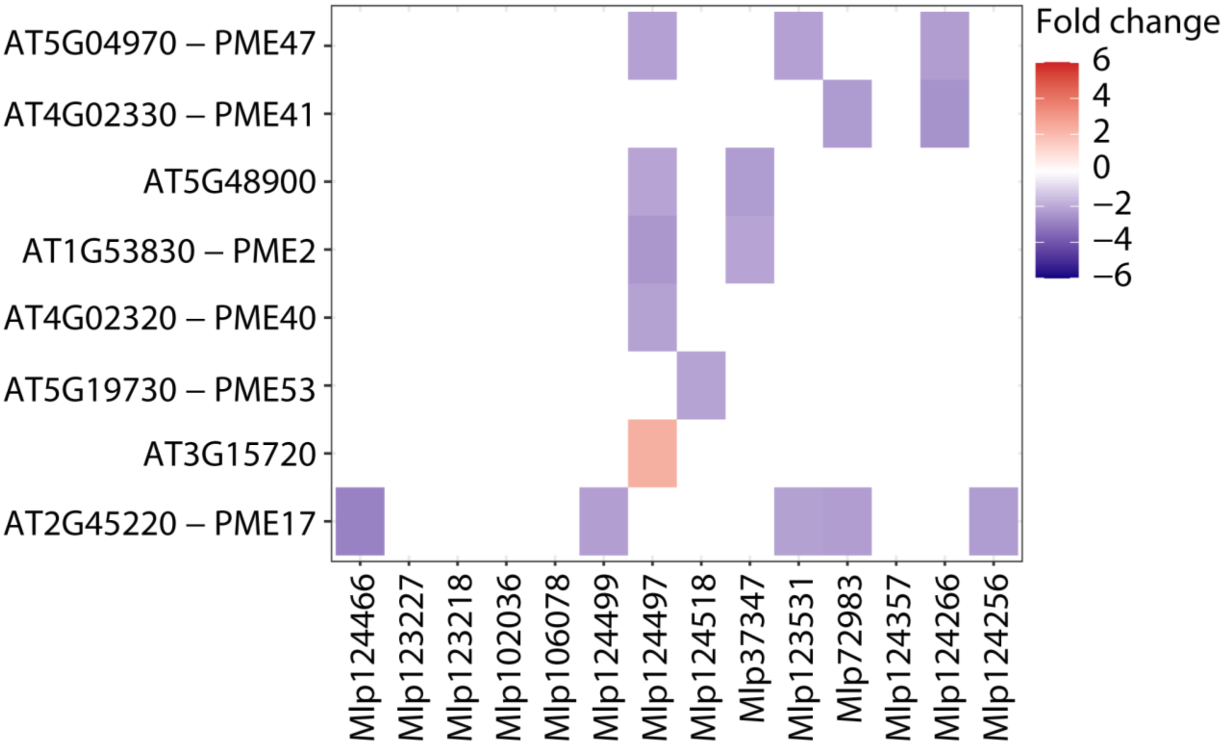

Figure S22. Pentose and glucuronate interconversions.

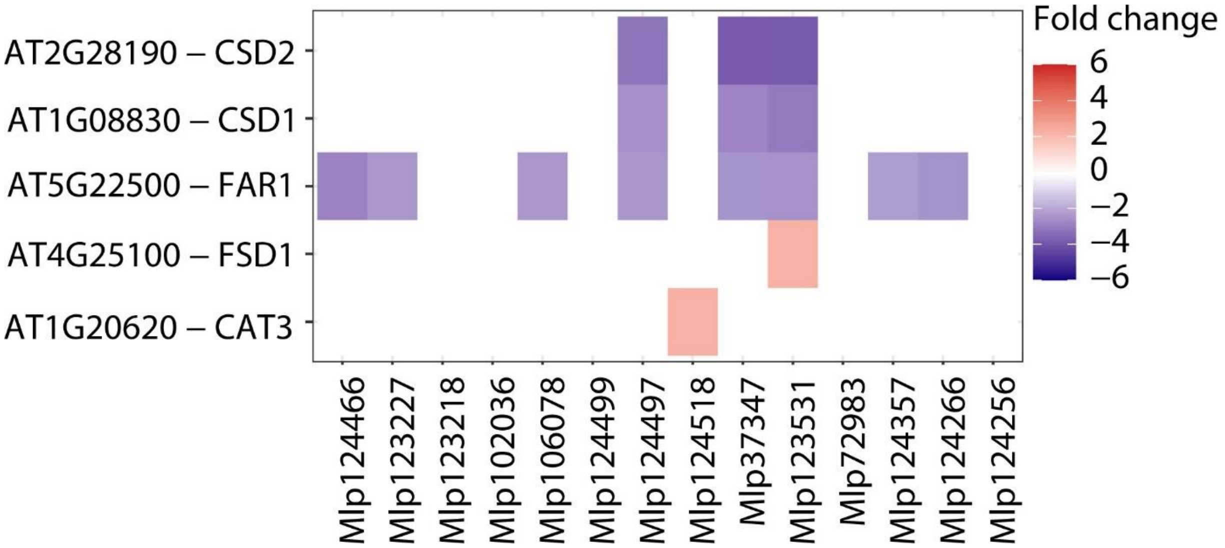

Figure S23. Peroxisome.

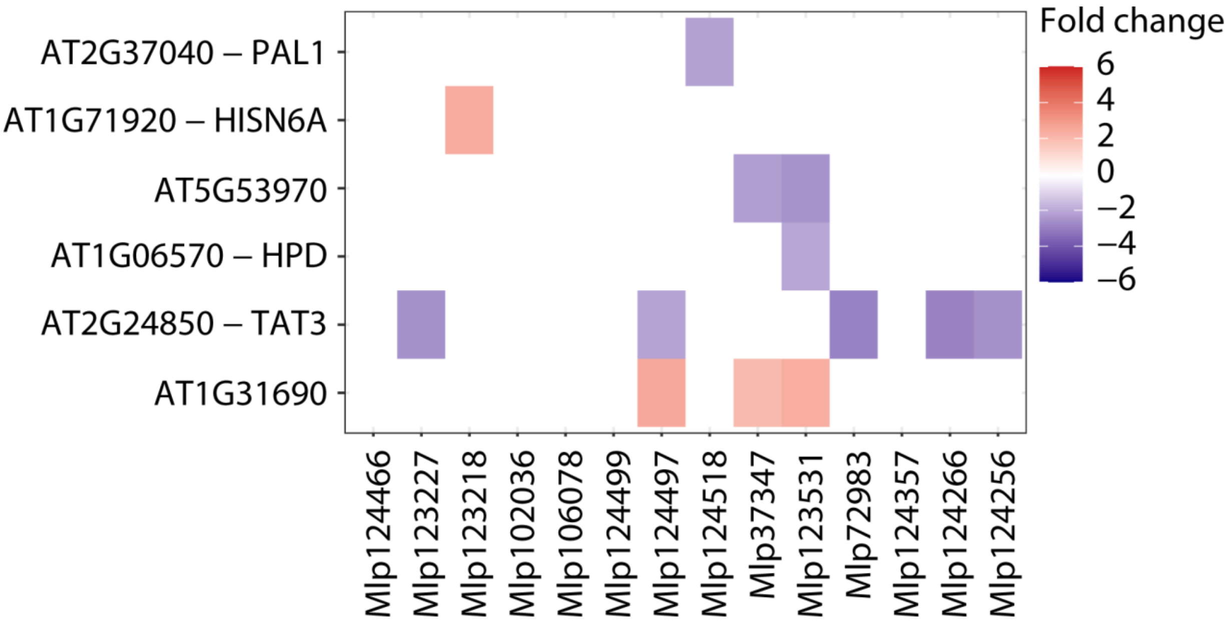

Figure S24. Phenylalanine metabolism.

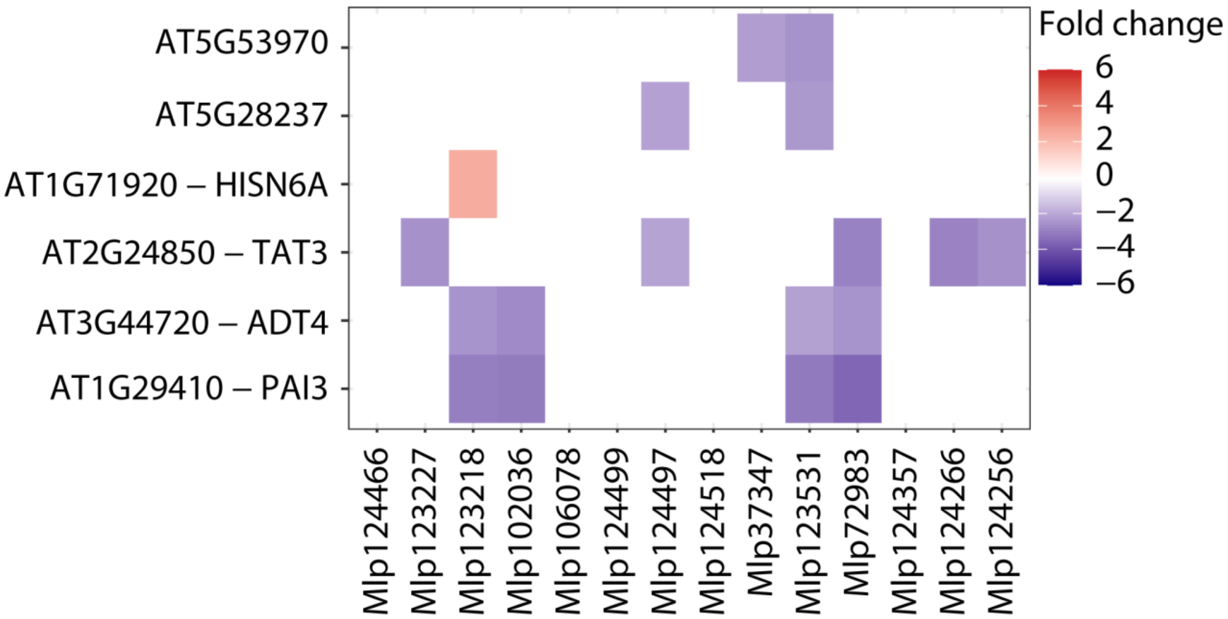

Figure S25. Phenylalanine, tyrosine and tryptophan biosynthesis.

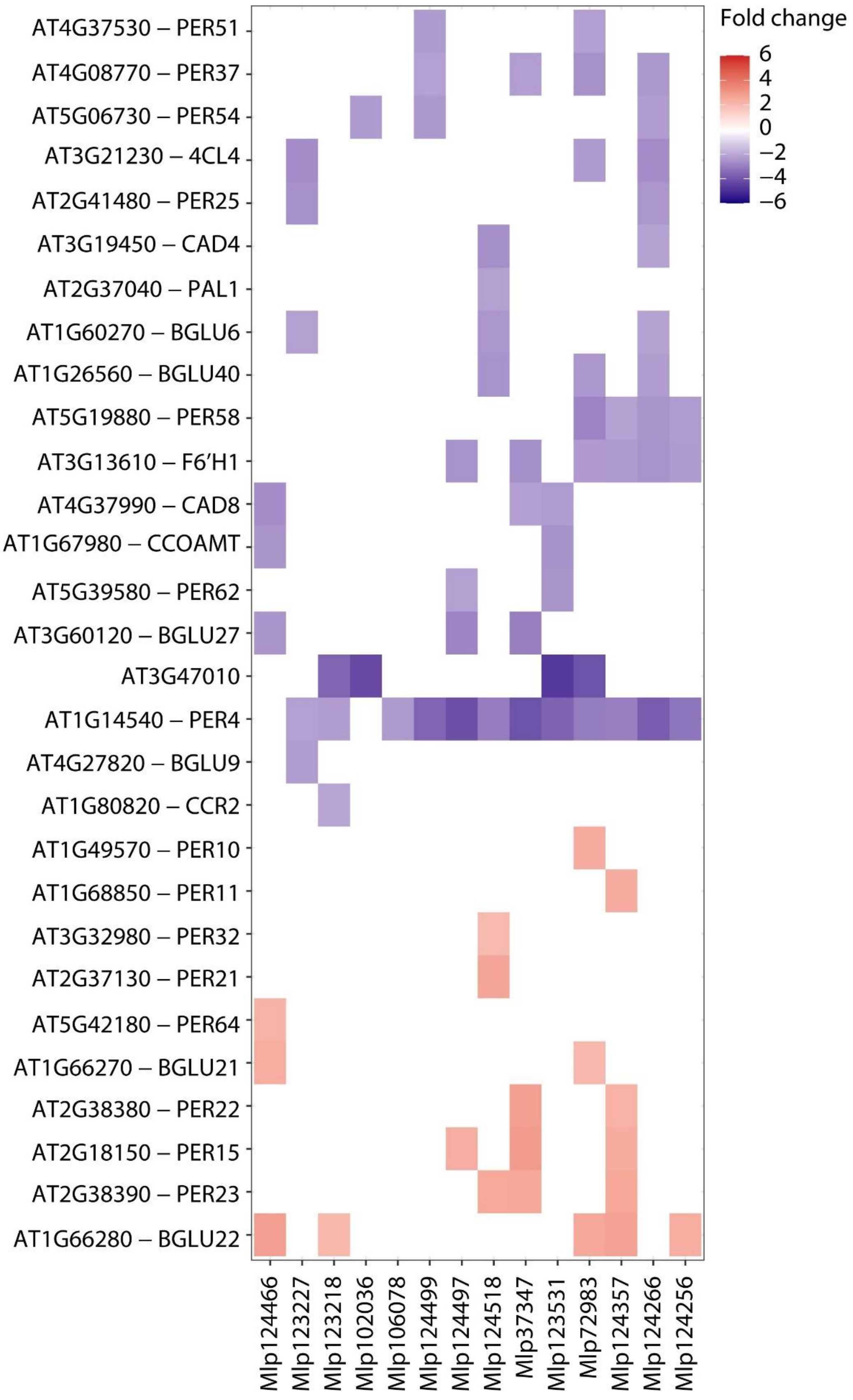

Figure S26. Phenylpropanoid biosynthesis.

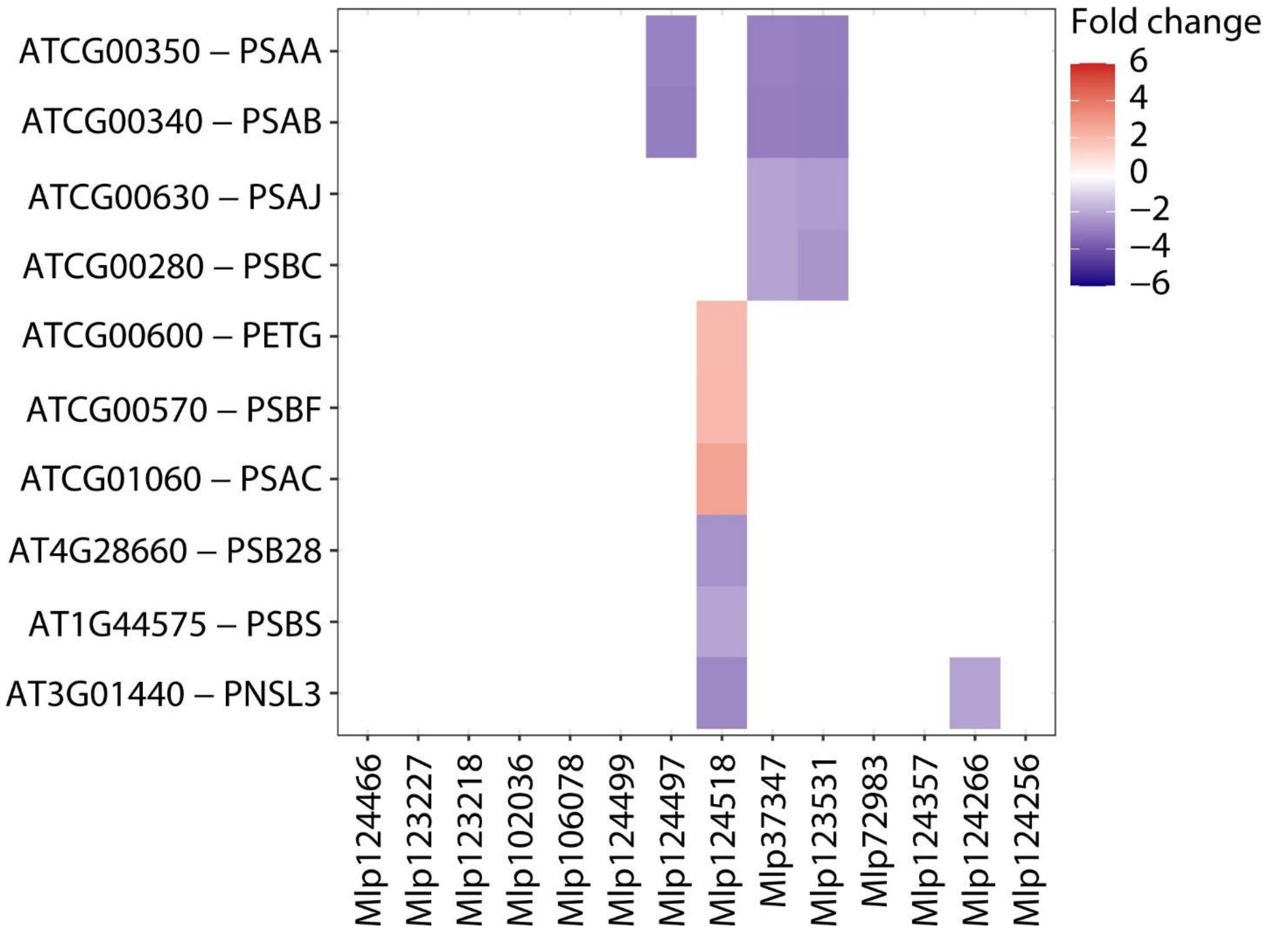

Figure S27. Photosynthesis.

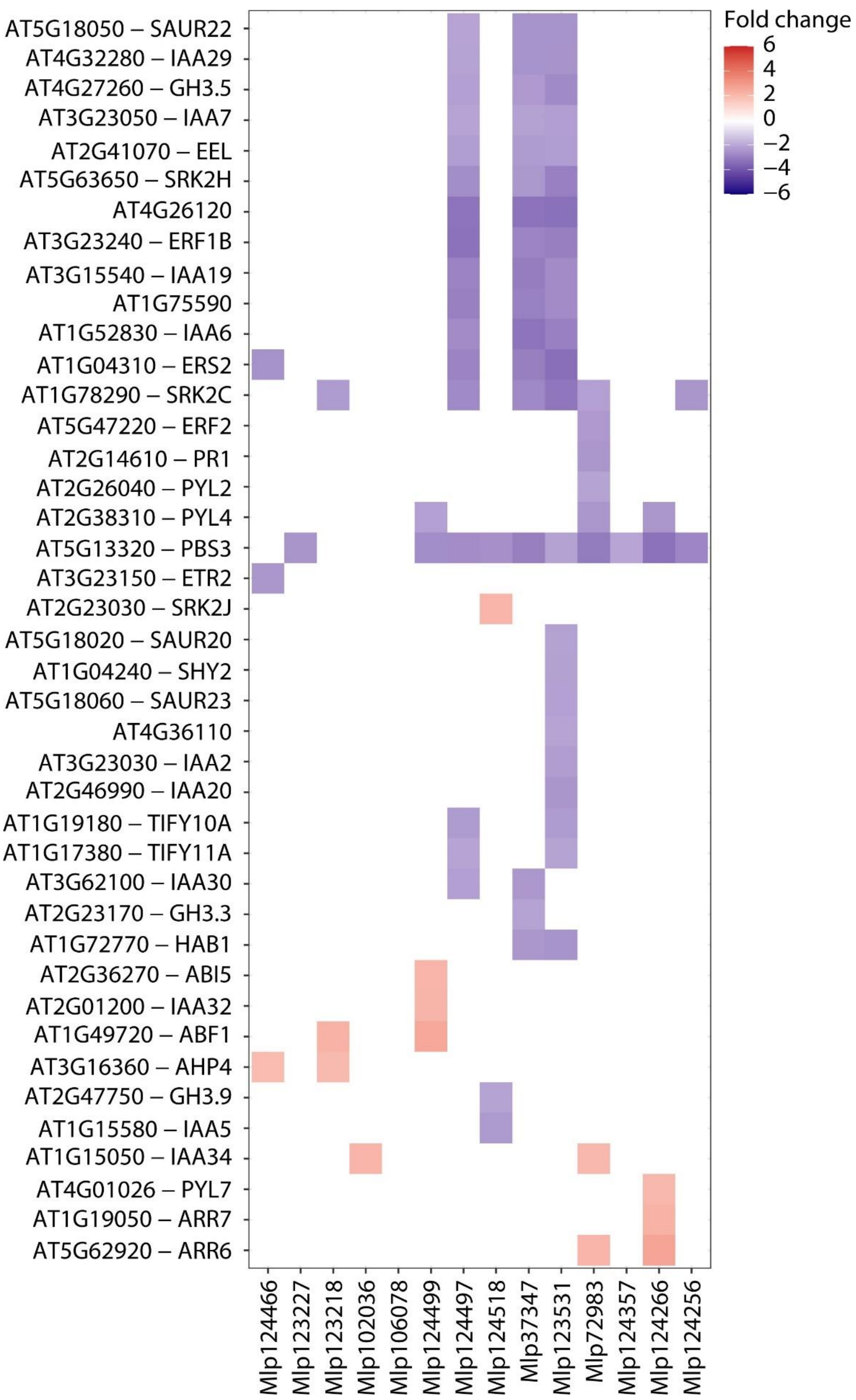

Figure S28. Plant hormone signal transduction.

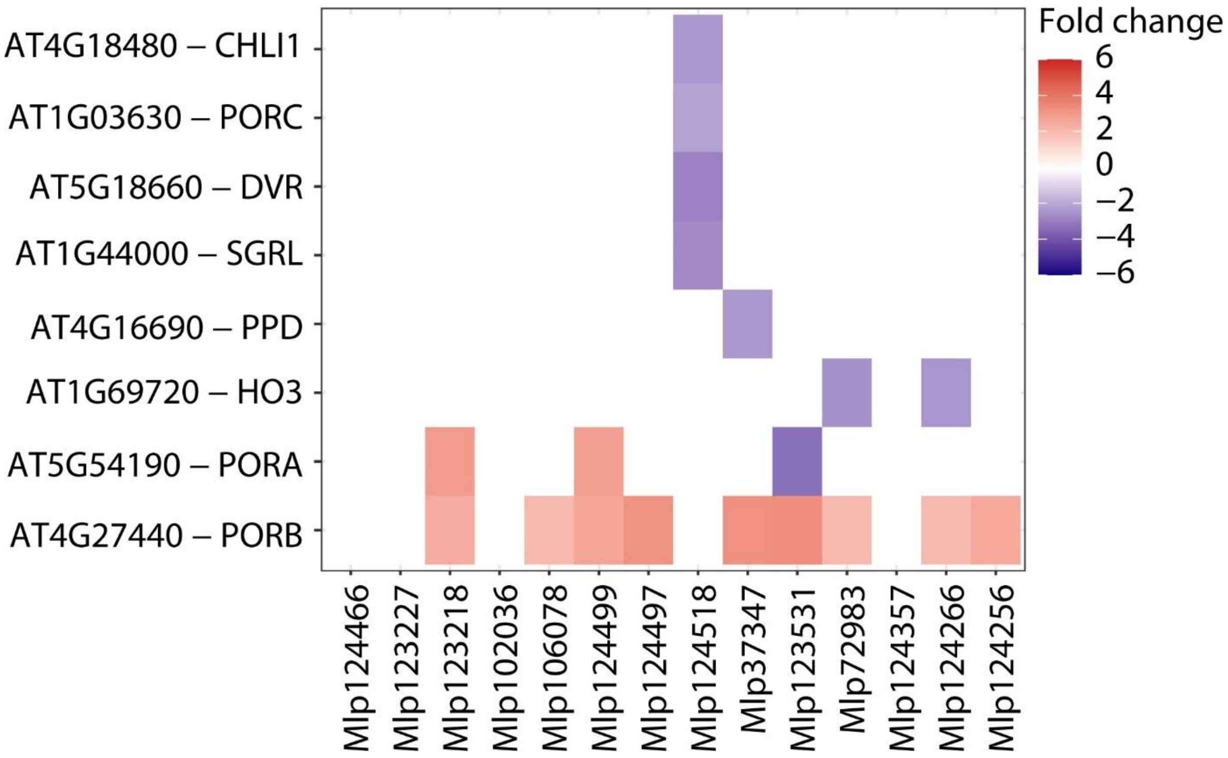

Figure S29. Porphyrin and chlorophyll metabolism.

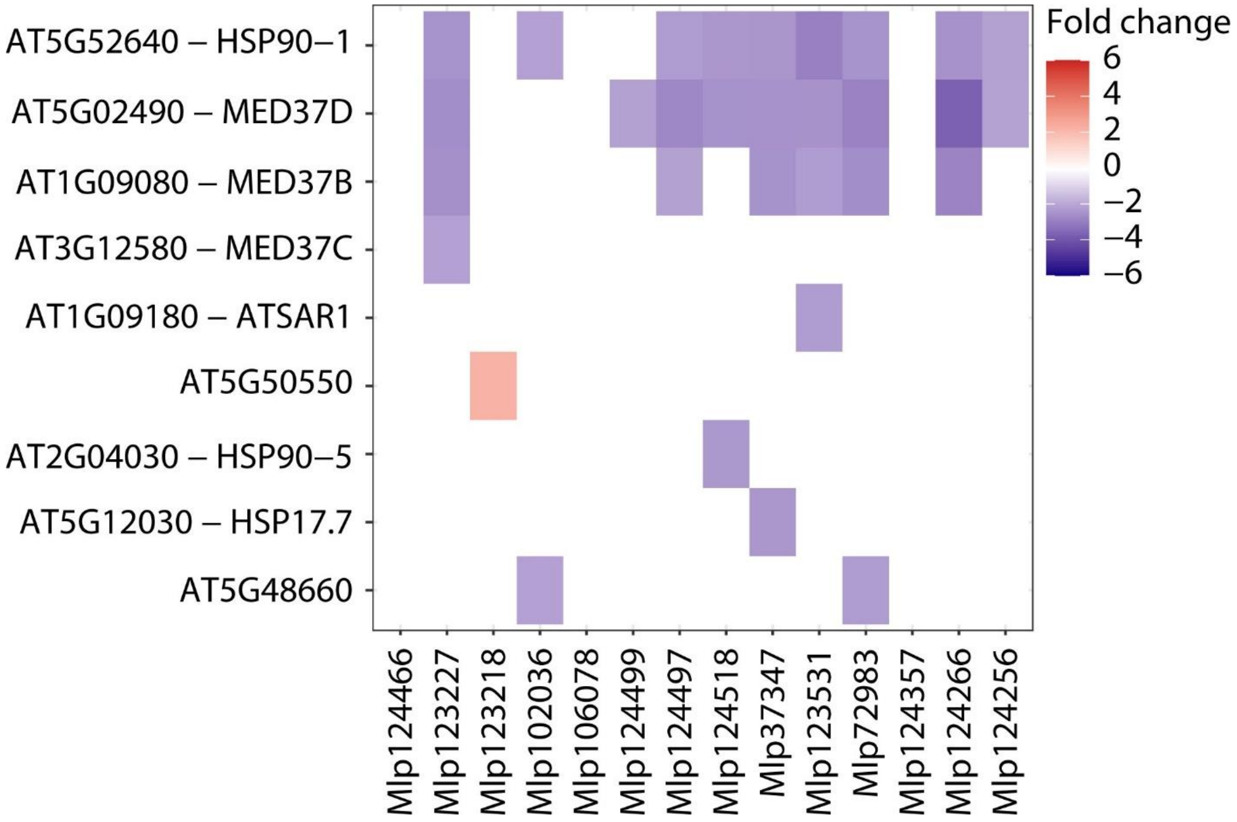

Figure S30. Protein processing in endoplasmic reticulum.

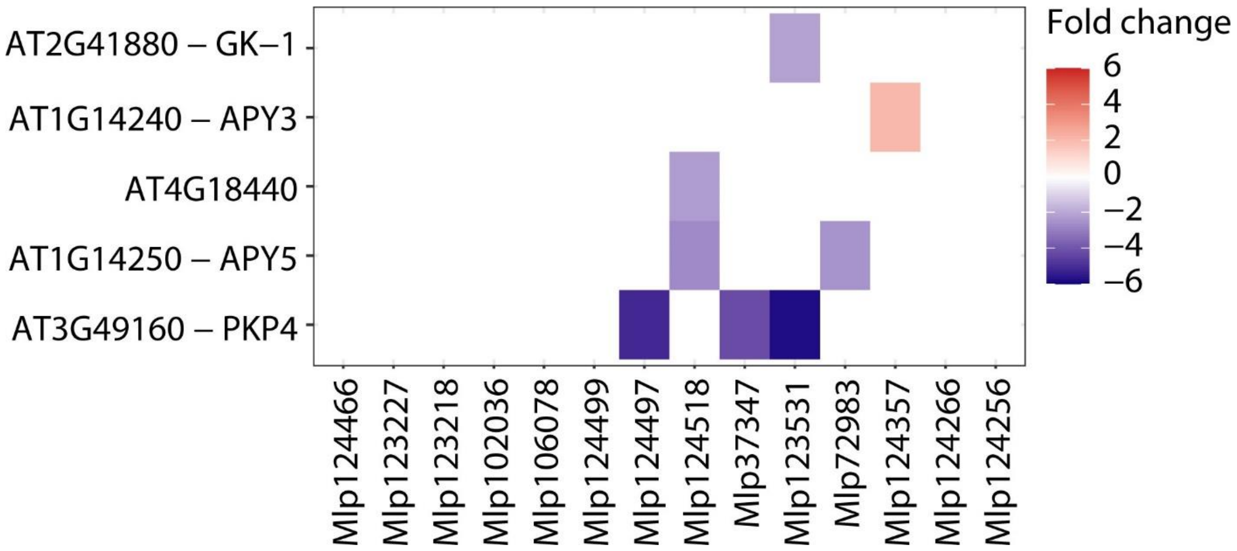

Figure S31. Purine metabolism.

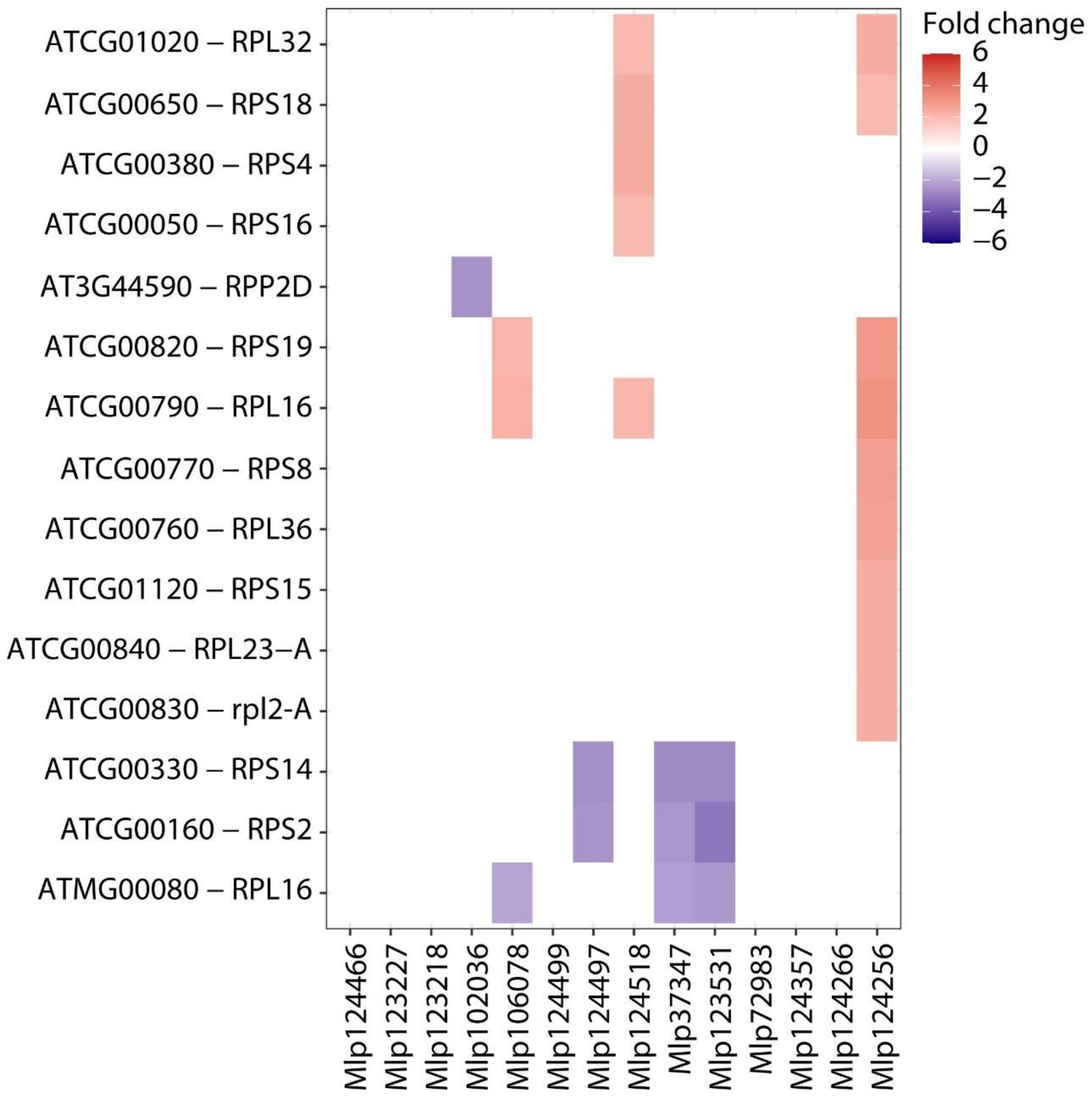

Figure S32. Ribosome.

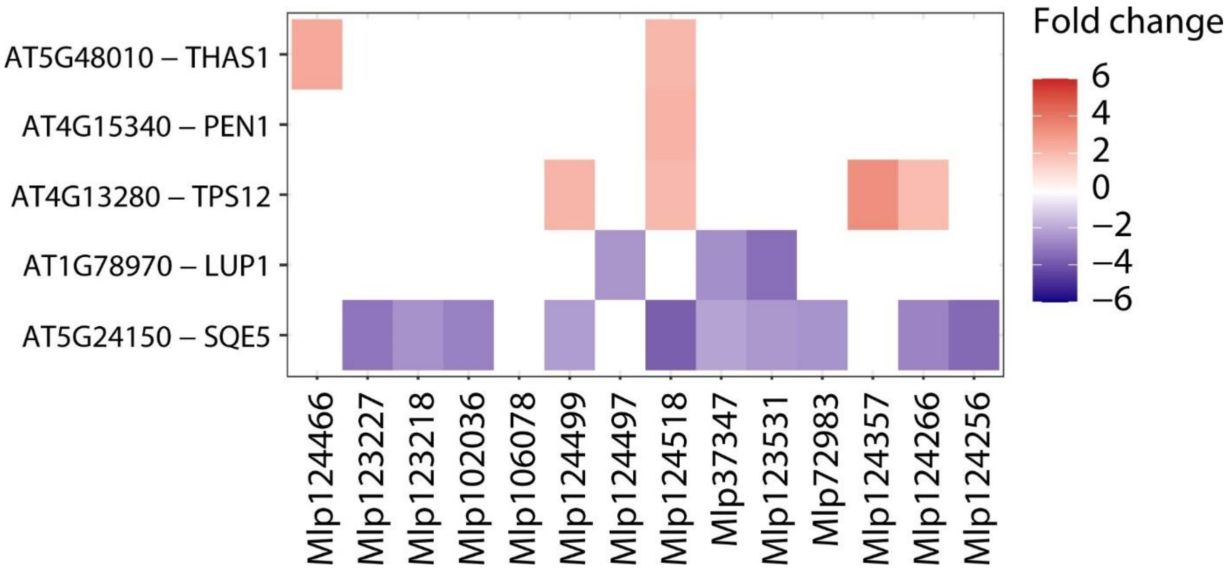

Figure S33. Sesquiterpenoid and triterpenoid biosynthesis.

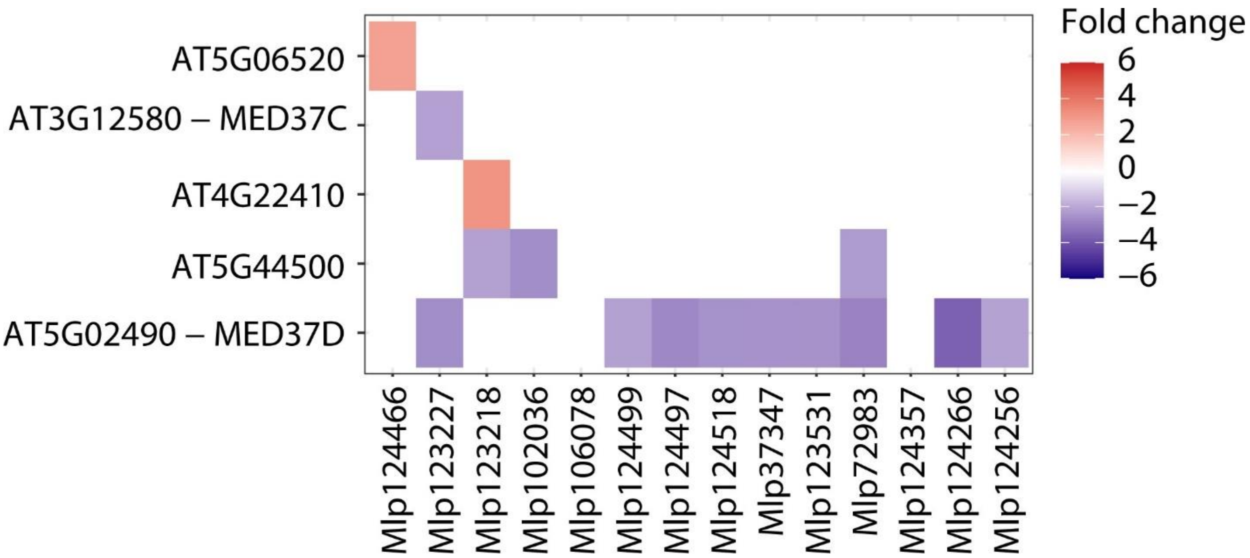

Figure S34. Spliceosome.

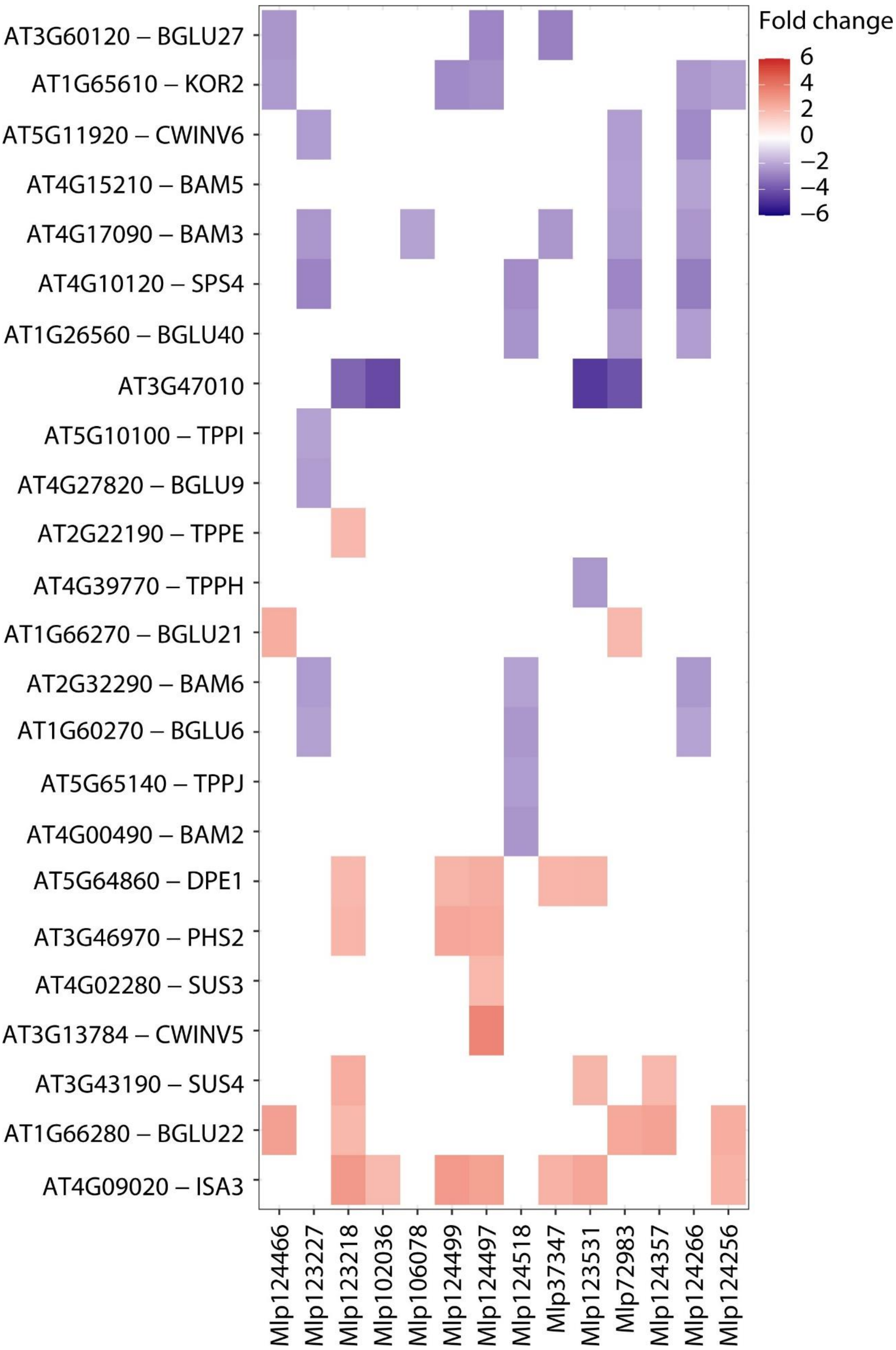

Figure S35. Starch and sucrose metabolism.

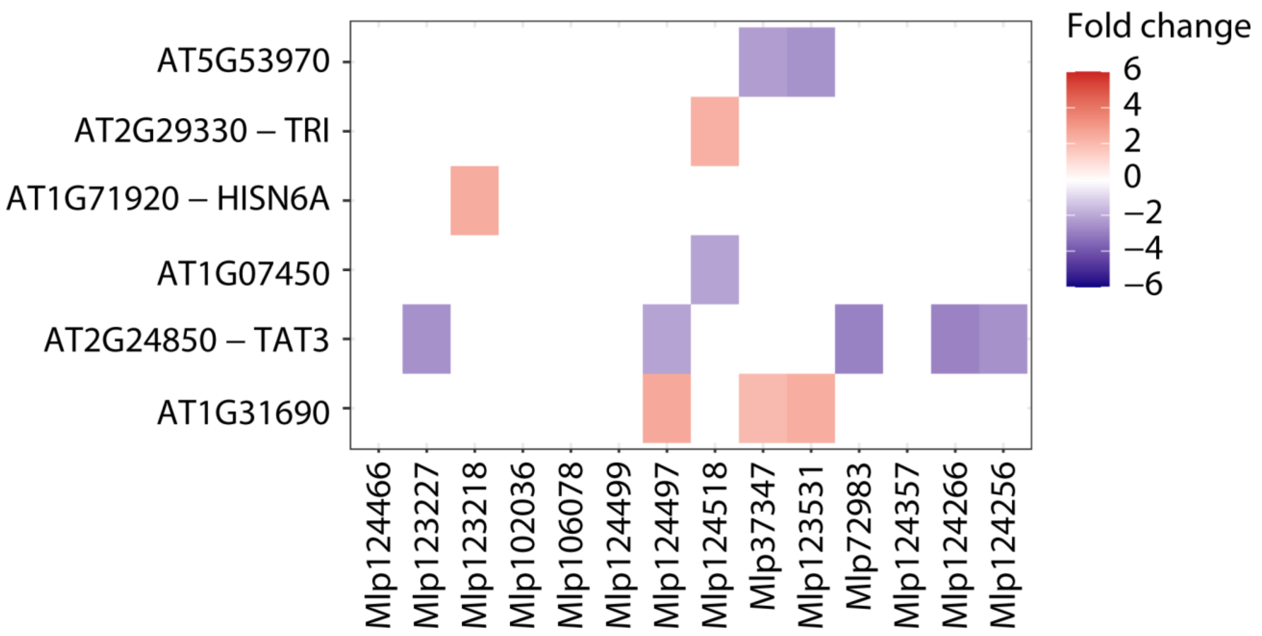

Figure S36. Tropane, piperidine and pyridine alkaloid biosynthesis.

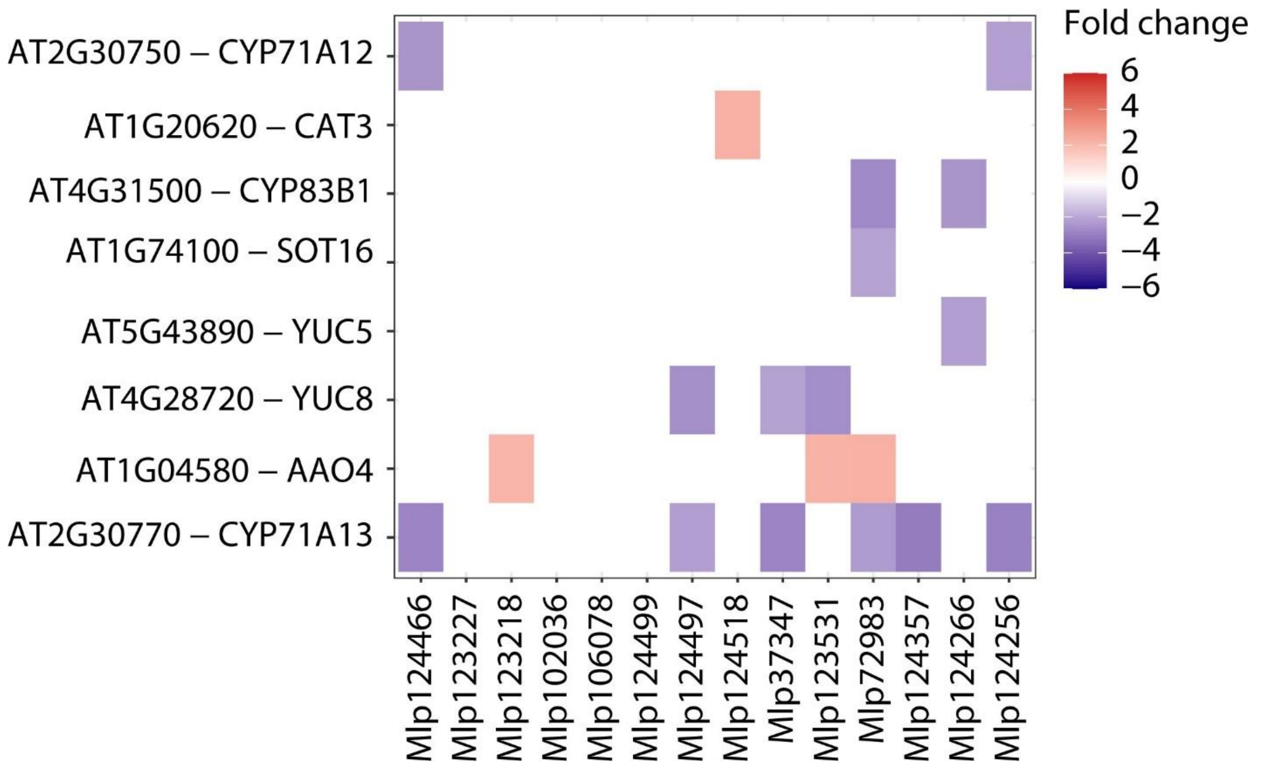

Figure S37. Tryptophan metabolism.

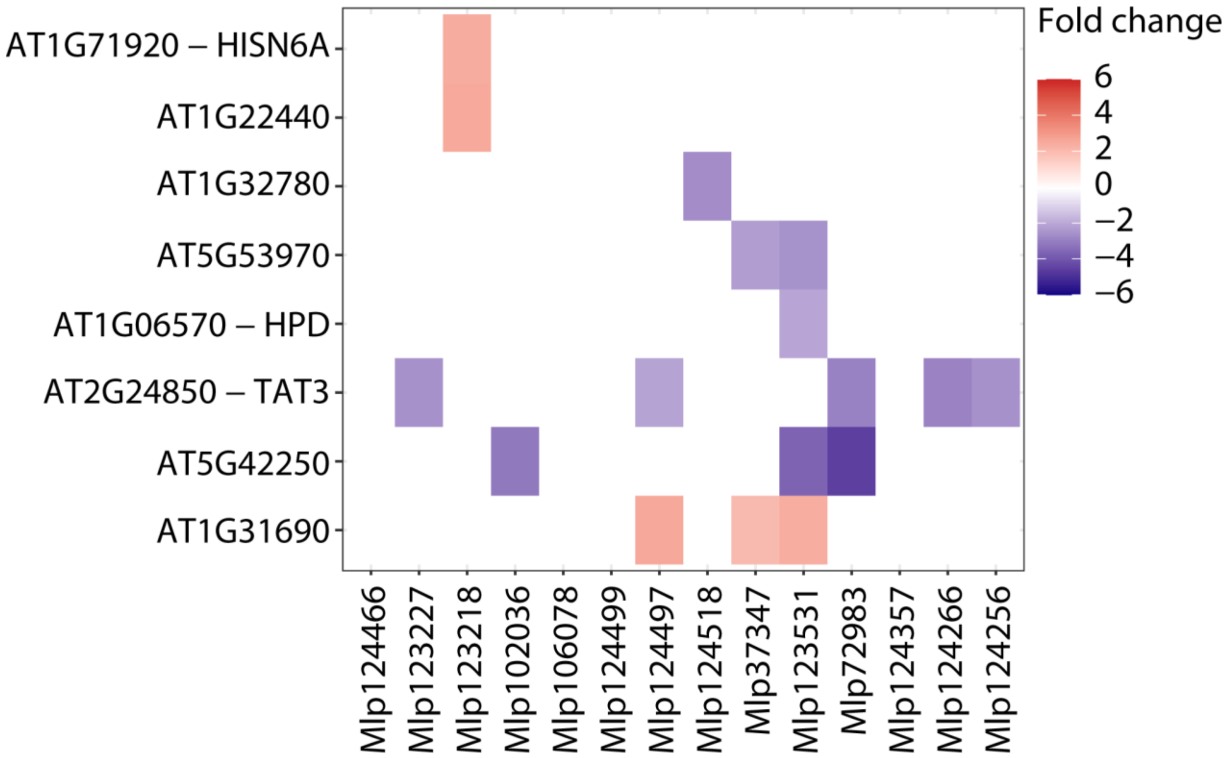

Figure S38. Tyrosine metabolism.

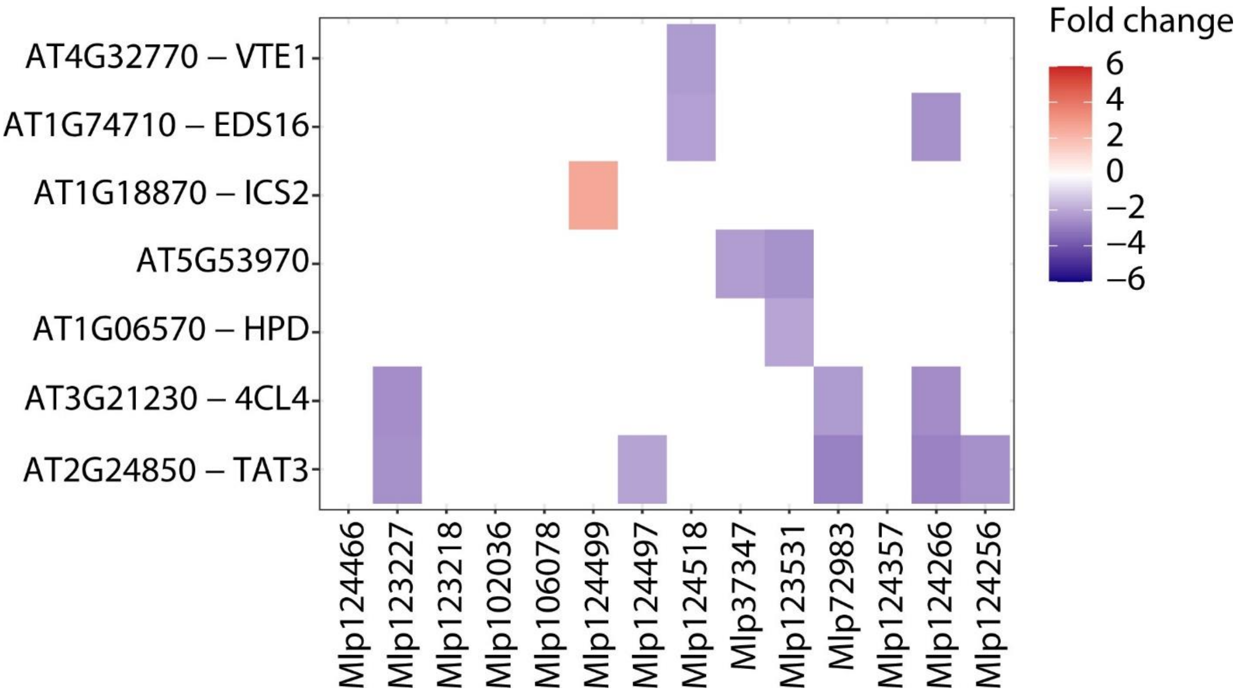

Figure S39. Ubiquinone and other terpenoid-quinone biosynthesis.

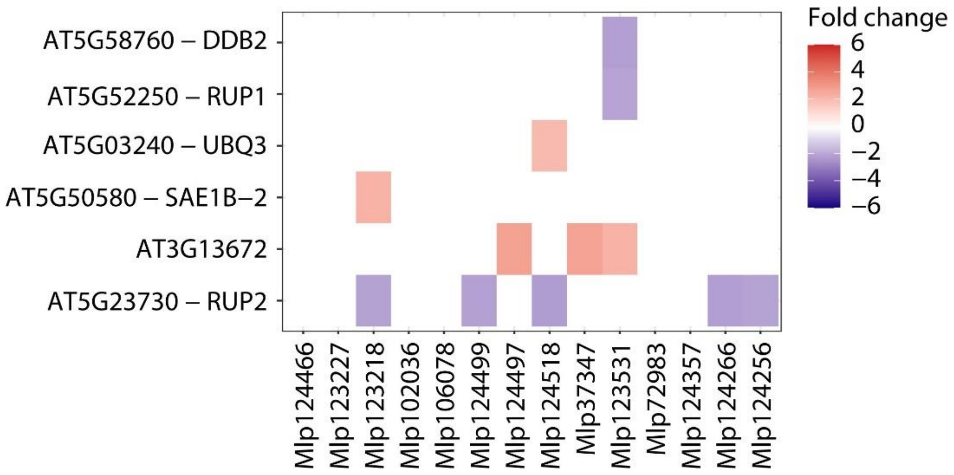

Figure S40. Ubiquitin mediated proteolysis.

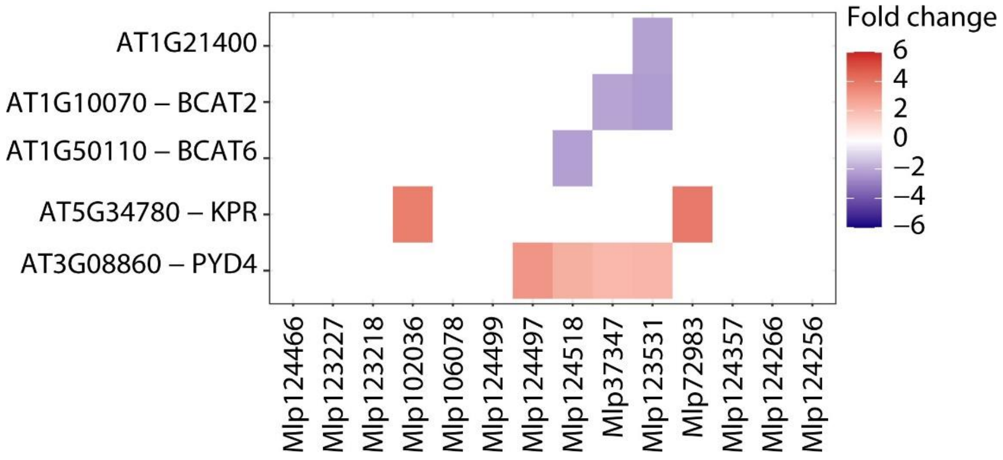

Figure S41. Valine, leucine and isoleucine degradation.

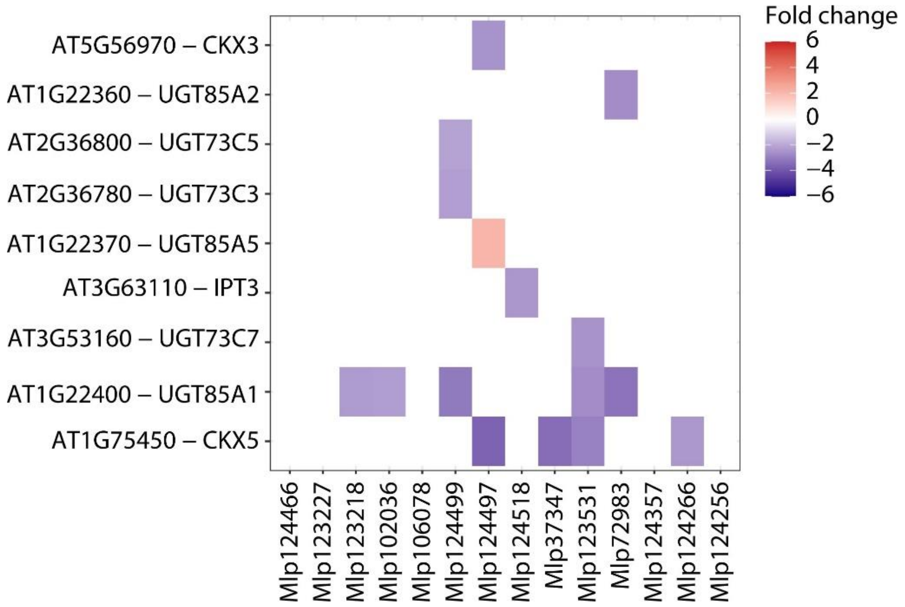

Figure S42. Zeatin biosynthesis.
