# Supplementary material for: Unrelated Fungal Rust Candidate Effectors Act on Overlapping Plant Functions
Source: Microorganisms. 2021 May 5;9(5):996. doi: 10.3390/microorganisms9050996 (PMC8148019; doi:10.3390/microorganisms9050996)
Supplement: Supplementary file 1 [file microorganisms-09-00996-s001.zip › Supplementary Table S6.pdf]

Supplementary Table S6. Sequencing results and alignment summary**.**

Sample name	Number of Reads	Average Quality	Mate inner distance	% Duplicate	Surviving	Aligned	%Aligned
CNT-1	12,959,539	37	50	39.57	11,420,407	6,578,769	58
CNT-2	17,628,091	39	60	24.15	16,441,057	15,326,908	93
CNT-3	44,449,775	39	84	32.81	40,740,349	38,447,039	94
102036-1	14,725,402	38	115	21.91	13,749,600	12,482,030	91
102036-2	16,097,041	38	110	24.53	14,863,654	13,794,120	93
102036-3	18,644,518	38	110	19.00	17,175,540	14,121,101	82
106078-1	14,806,842	38	70	27.15	13,707,505	12,657,991	92
106078-2	16,097,885	38	70	23.93	14,896,296	13,620,408	91
106078-3	57,392,974	39	55	35.94	52,226,039	49,146,796	94
123218-1	14,941,915	38	60	20.13	13,911,559	12,766,811	92
123218-2	19,202,251	38	60	24.98	17,740,615	16,007,510	90
123218-3	14,573,522	38	65	27.47	13,336,684	10,353,032	78
123227-1	14,506,123	38	60	23.68	13,559,794	12,650,378	93
123227-2	13,690,077	38	70	21.88	12,740,323	11,674,516	92
123227-3	14,221,348	38	65	25.24	13,044,995	10,662,871	82
123531-1	30,255,033	39	70	53.11	28,677,354	26,311,924	92
123531-2	29,847,359	39	60	46.49	28,400,730	26,243,184	92
123531-3	33,185,737	39	65	30.72	31,504,015	29,142,533	93
124256-1	13,219,309	38	110	22.30	12,270,185	11,319,954	92
124256-2	15,023,134	38	60	23.12	13,721,513	11,218,900	82
124256-3	53,119,639	39	60	38.35	48,711,081	45,705,676	94
124266-1	14,512,508	37	60	21.17	13,288,961	12,184,913	92
124266-2	14,343,047	37	100	23.84	13,084,917	11,752,213	90
124266-3	16,738,628	37	70	21.41	15,203,780	13,696,801	90
124357-1	17,594,958	38	80	22.41	16,313,595	15,140,354	93
124357-2	15,759,938	38	115	21.54	14,538,561	13,287,744	91
124357-3	17,205,907	38	105	19.78	15,823,808	13,959,229	88
124466-1	15,994,909	38	150	21.84	14,896,010	13,790,242	93
124466-2	17,877,587	38	105	20.43	16,470,499	14,898,728	90
124466-3	12,451,485	39	70	19.33	11,616,591	10,726,259	92
124497-1	15,410,965	39	70	21.36	14,559,104	13,634,149	94
124497-2	25,299,826	39	60	29.76	23,916,582	22,416,808	94
124497-3	14,250,049	39	70	24.61	12,724,994	11,533,107	91
124499-1	12,422,097	38	60	22.33	11,615,353	10,818,003	93
124499-2	14,079,283	38	60	21.04	13,059,459	11,939,187	91
124499-3	16,445,351	38	100	27.87	14,910,023	13,122,045	88
124518-1	14,485,964	36	110	23.32	13,094,223	11,731,759	90
124518-2	12,911,956	36	105	23.39	11,553,534	9,944,892	86
124518-3	11,347,886	34	45	21.35	9,346,542	8,246,506	88
37347-1	27,784,075	39	70	28.79	26,345,883	25,136,138	95
37347-2	23,439,580	38	65	31.35	21,914,868	20,722,973	95
37347-3	14,033,172	39	65	23.16	13,268,922	12,674,770	96
72983-1	23,393,778	38	55	21.00	21,669,105	19,896,426	92
72983-2	15,230,634	38	55	24.76	13,982,126	12,497,170	89
72983-3	14,014,784	38	64	24.18	12,928,472	11,095,184	86
