# Supplementary figures and images for: Unrelated Fungal Rust Candidate Effectors Act on Overlapping Plant Functions
Source: Microorganisms. 2021 May 5;9(5):996. doi: 10.3390/microorganisms9050996 (PMC8148019; doi:10.3390/microorganisms9050996)

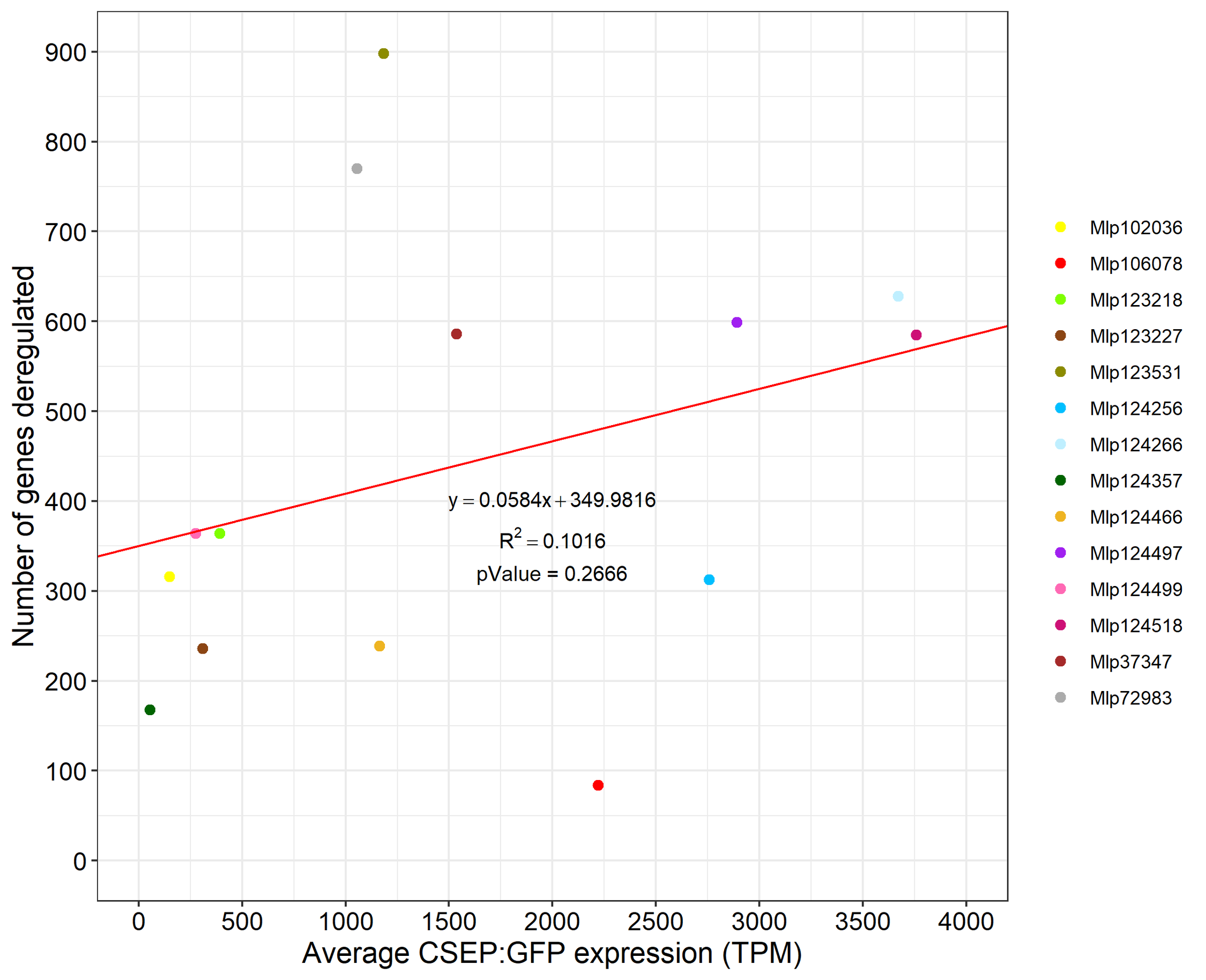

Supplement: Supplementary file 1 [file microorganisms-09-00996-s001.zip › Figure S1.tif]

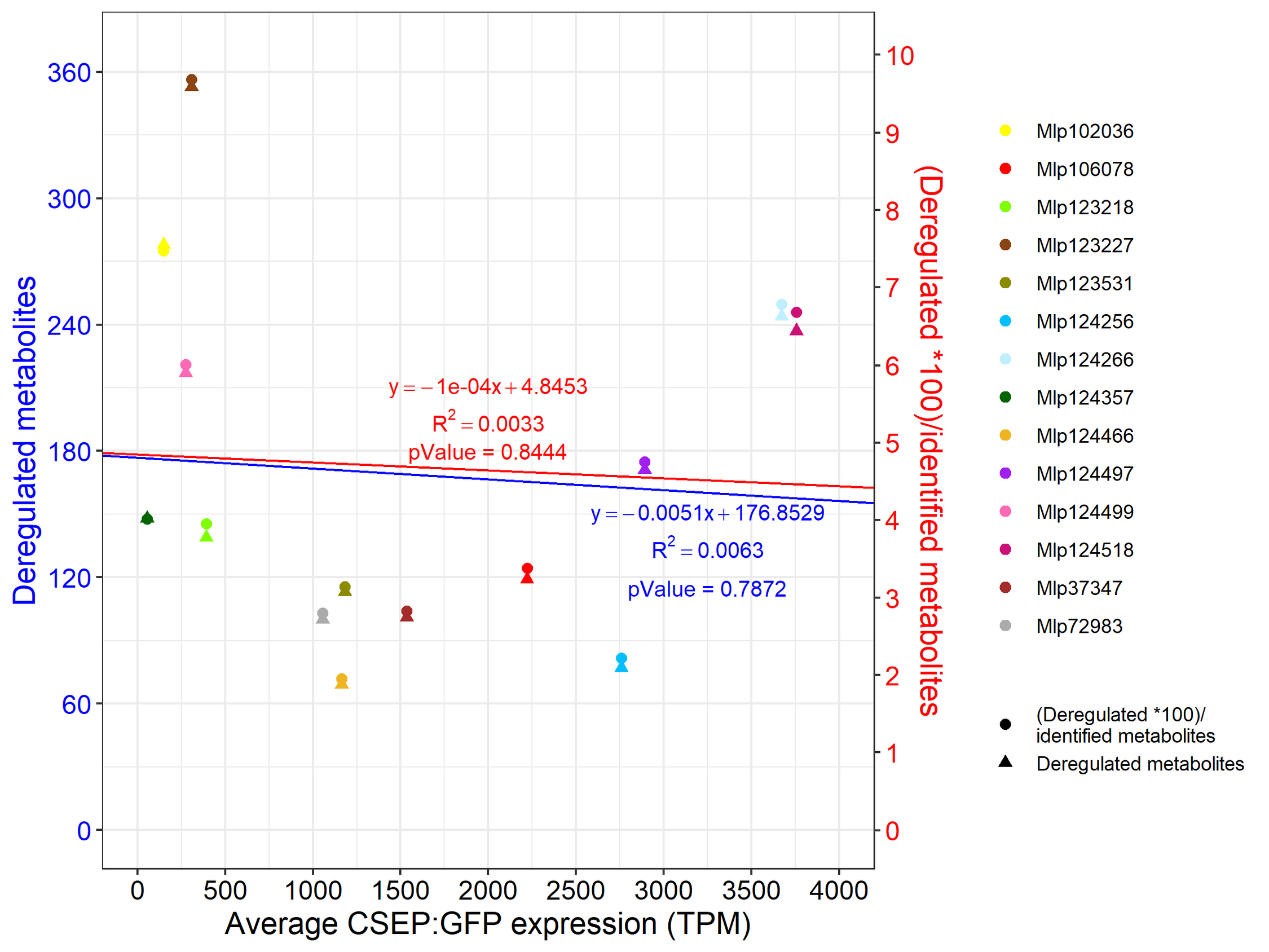

Supplement: Supplementary file 1 [file microorganisms-09-00996-s001.zip › Figure S2.tif]

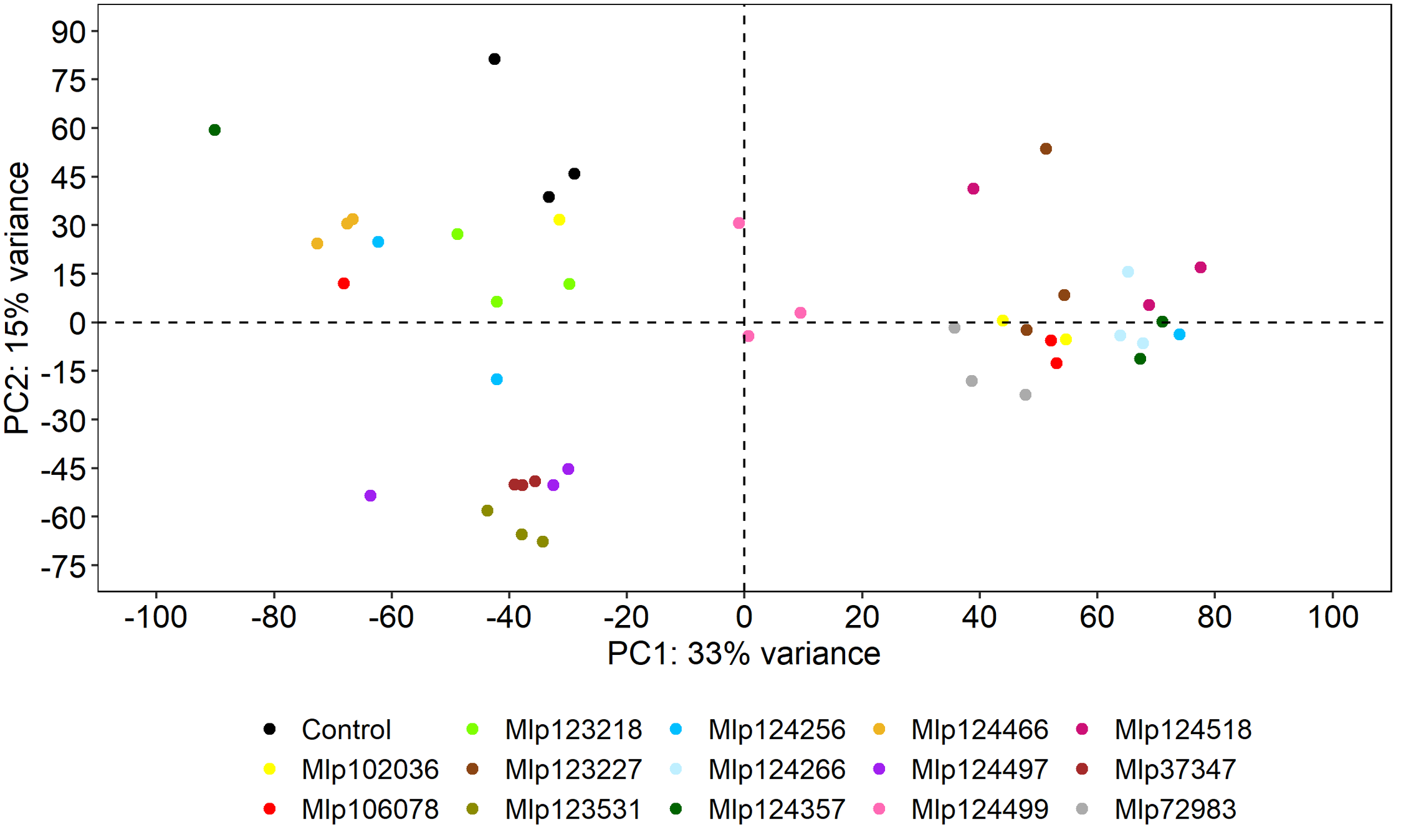

Supplement: Supplementary file 1 [file microorganisms-09-00996-s001.zip › Figure S3.tif]

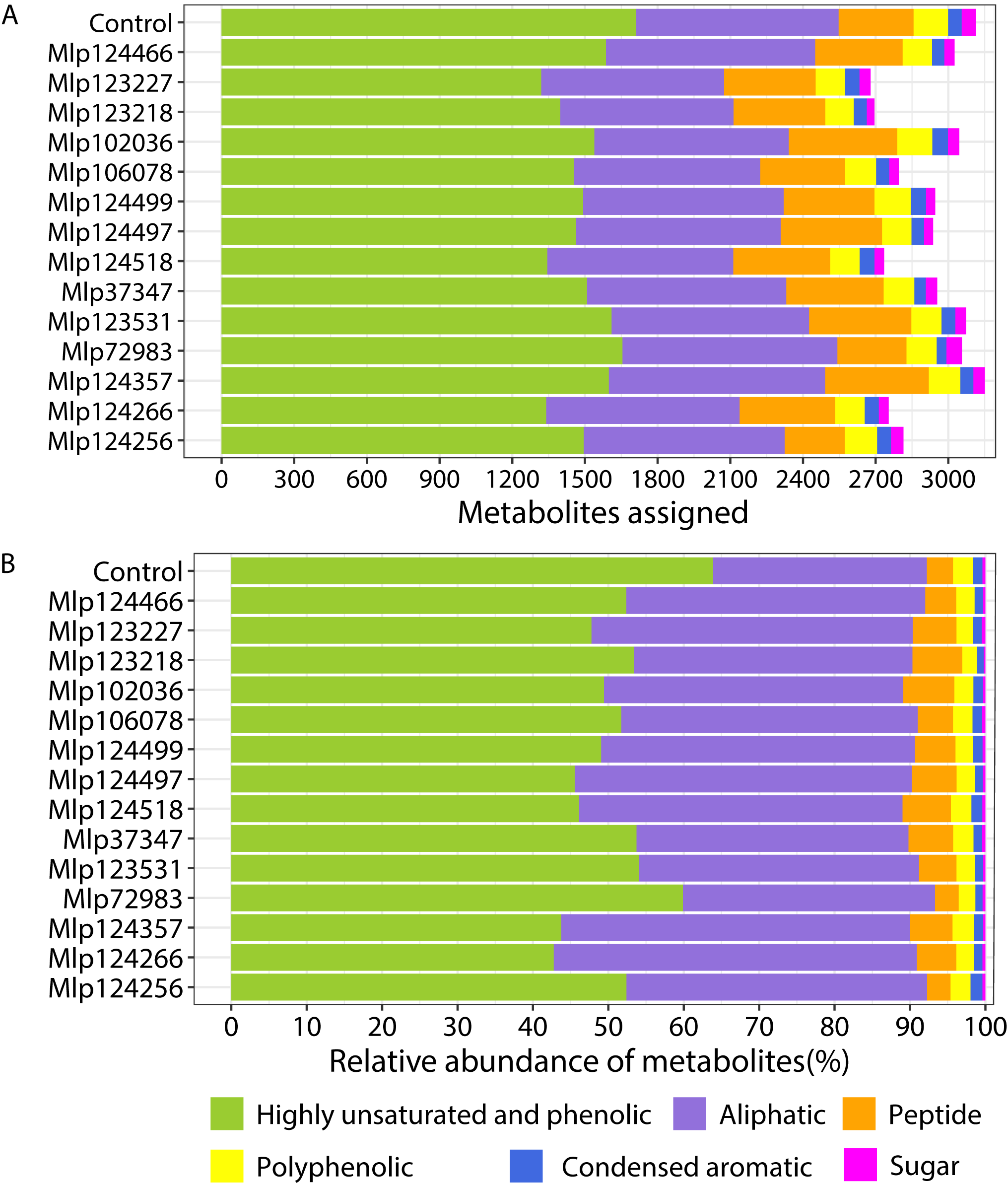

Supplement: Supplementary file 1 [file microorganisms-09-00996-s001.zip › Figure S4.tif]
